# Stability of Liver Radiomics across Different 3D ROI Sizes—An MRI In Vivo Study

**DOI:** 10.3390/tomography7040073

**Published:** 2021-12-03

**Authors:** Laura J. Jensen, Damon Kim, Thomas Elgeti, Ingo G. Steffen, Bernd Hamm, Sebastian N. Nagel

**Affiliations:** 1 Department of Radiology, Corporate Member of Freie Universität and Humboldt-Universität zu Berlin, Charité–Universitätsmedizin Berlin, Hindenburgdamm 30, 12203 Berlin, Germany; damon.kim@charite.de (D.K.); thomas.elgeti@charite.de (T.E.); ingo.steffen@charite.de (I.G.S.); bernd.hamm@charite.de (B.H.); sebastian.nagel@charite.de (S.N.N.); 2 Department of Radiology-Pediatric Radiology, Charité–Universitätsmedizin Berlin, Corporate Member of Freie Universität Berlin and Humboldt-Universität zu Berlin, Augustenburger Platz 1, 13353 Berlin, Germany

**Keywords:** radiomics, texture analysis, magnetic resonance imaging, liver, reproducibility, robustness

## Abstract

We aimed to evaluate the stability of radiomic features in the liver of healthy individuals across different three-dimensional regions of interest (3D ROI) sizes in T1-weighted (T1w) and T2-weighted (T2w) images from different MR scanners. We retrospectively included 66 examinations of patients without known diseases or pathological imaging findings acquired on three MRI scanners (3 Tesla I: 25 patients, 3 Tesla II: 19 patients, 1.5 Tesla: 22 patients). 3D ROIs of different diameters (10, 20, 30 mm) were drawn on T1w GRE and T2w TSE images into the liver parenchyma (segment V–VIII). We extracted 93 radiomic features from the different ROIs and tested features for significant differences with the Mann–Whitney-U (MWU)-test. The MWU-test revealed significant differences for most second- and higher-order features, indicating a systematic difference dependent on the ROI size. The features mean, median, root mean squared (RMS), 10th percentile, and 90th percentile were not significantly different. We also assessed feature robustness to ROI size variation with overall concordance correlation coefficients (OCCCs). OCCCs across the different ROI-sizes for mean, median, and RMS were excellent (>0.90) in both sequences on all three scanners. These features, therefore, seem robust to ROI-size variation and suitable for radiomic studies of liver MRI.

## 1. Introduction

Radiomics analysis translates a medical image into quantitative features that are otherwise not perceptible to the human eye [1,2]. Since these features have quantitative values, they can be statistically linked to various biological and diagnostic endpoints [2,3]. For example, Jajodia et al. built prediction models to evaluate the outcome in cervical cancer based on radiomic features derived from ADC maps [4]. Bobholz et al. revealed parallels between MR-derived radiomic features of brain tumors and their texture in the histopathological specimen to conclude the underlying tissue histology [5]. There are plenty of radiomic studies attempting to characterize liver abnormalities with the aim to predict their outcome [6]. Contrary to the growing body of published data, radiomics analysis is still not applicable in clinical routine, possibly because of the lack of reproducibility [2,7].

Numerous factors influence radiomic features, such as image acquisition parameters, reconstruction algorithms, segmentation, and applied software [3,8,9,10,11,12,13]. Inter-scanner and inter-vendor variability of radiomic features have been observed for CT and MR imaging [14,15]. Ammari et al. found that field strength and the signal-to-noise ratio influences many radiomic features investigating brain phantoms and volunteers’ brains on 1.5 Tesla and 3 Tesla MRI [16].

Furthermore, several studies indicated that the volume of a segmented lesion influences the features’ quantity. Lu et al. found CT-derived radiomics of malign kidney tumors influenced by tumor size and suggested removing confounded features in successive steps to establish a radiomics signature [17]. Traverso et al. detected a strong correlation between feature quantity and tumor volume in 30% of 841 CT-derived radiomic features from lung and head and neck tumors [18]. Roy et al. observed that tumor volume, noise, and image resolution significantly impacts MR-derived radiomic features in breast cancer lesions in both T1-weighted (T1w) and T2-weighted (T2w) images [19]. And as the developers of PyRadiomics software state, features such as energy, total energy, and root mean squared are volume confounded by design [20]. A recent study compared radiomics derived from various ROI sizes in CT and MR images of a water phantom, showing that the majority of first-, second-, and higher-order features differed significantly [21].

This study, therefore, aimed to identify MR-derived radiomic features of healthy liver parenchyma that are stable across different 3D ROI sizes considering T1- and T2-weighted sequences on different MR scanners and field strengths.

## 2. Materials and Methods

### 2.1. Study Population

The institutional review board approved our retrospective study [EA1/104/19]. We retrospectively screened MR examinations that were conducted between April 2012 and August 2020 to rule out chronic inflammatory bowel disease. We included healthy patients without any present or preexisting disease and without structural or parenchymal liver abnormalities. Furthermore, patients with benign liver lesions such as cysts and patients with abdominopelvic metal implants (e.g., dorsal instrumentation of the spine or hip replacement) were excluded to avoid an influence by artifacts. We also excluded patients with apparent signal alterations between in- and opposed phase in the spoiled gradient echo sequences (T1w GRE FLASH in our study, equivalent to T1 FFE or SPGR sequences) to preclude influence by fatty degeneration or iron deposition. We included examinations from three MR scanners: two 3 Tesla MRI scanners of the same model (Magnetom Skyra, Siemens Healthineers, Erlangen, Germany) and one 1.5 Tesla MRI scanner (Magnetom Aera, Siemens Healthineers, Erlangen, Germany). The patient groups on the three scanners were tested for significant differences that could impact the results. Patient gender distribution was tested with a chi-square test (*p* = 0.85) and age distribution with the Kruskal-Wallis test (*p* = 0.12). Thus, significant differences in gender and age between the patient groups were excluded.

Details of the patient population are summarized in Table 1.

### 2.2. Image Acquisition

All 66 patients were examined in clinical routine. The patients had to be fasting for at least 4 h before the examination and were required to fractionally drink 0.75 L of 2.5% mannitol solution one hour before the examination. All patients were examined using the modified Sellink technique with a duration of approximately 40 min scanning time. The T2w Turbo Spin Echo (TSE) Half Fourier Acquisition Single Shot Turbo Spin Echo (HASTE) axial and the T1w Gradient Echo (GRE) Fast Low Angle Shot (FLASH) axial sequences that we analyzed were acquired within the first ten minutes of the scan and before intravenous contrast administration in a fixed examination protocol with a multi-breath-hold regimen for each sequence. Patients were placed into the scanner headfirst and with a phased-array body coil placed on the abdomen. The field of view was adapted to the individual patient’s size. All scanners were used in clinical routine imaging and are calibrated regularly by controlling the homogeneity of the B_0_ field.

Details of the MRI scanning parameters are listed in Table 2.

### 2.3. Image Analysis

In each included examination, three-dimensional sphere-shaped regions of interest (3D ROIs) were drawn in liver segment V, VI, VII or VIII. ROIs were not copied between sequences since the position of the liver is slightly altered according to the multi-breath-hold regimen. We visually correlated the slices and used anatomic landmarks to place the center of the ROI identically between slices. The segments were chosen to exclude large blood vessels and bile ducts while only including hepatic parenchyma. 3D ROIs were drawn using 3D Slicer ([22]; 3D Slicer, Version 4.10.0, http://www.slicer.org, accessed on 21 April 2021) by a radiologist with over four years of experience in MR imaging. ROI diameters were set to 10, 20, and 30 mm. By choosing the 3D ROI diameters of 10, 20, and 30 mm, we aimed to cover typical sizes of liver lesions. With larger ROIs, it also becomes more and more difficult to exclude large blood vessels and bile ducts.

**Figure 1:**
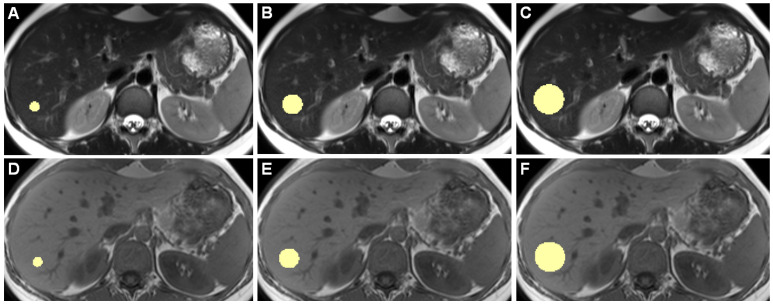
Example slices of 3D ROIs of one healthy individual, acquired on the 3 Tesla I scanner, are shown. Images (**A**–**C**) are from a T2-weighted TSE HASTE sequence, (**D**–**F**) are from a T1-weighted GRE FLASH sequence. (**A**,**D**) show 10-mm diameter ROIs, (**B**,**E**) 20 mm, and (**C**,**F**) 30 mm ROIs. While drawing ROIs manually throughout all included patients, we aimed to only include hepatic parenchyma while excluding any apparent blood vessels or bile ducts.

Figure 1 shows example slices of a 3D ROI.

### 2.4. Radiomic Feature Extraction

Radiomic features were extracted using PyRadiomics (Version 3.0) [20], following the instructions of the Image Biomarker Standardisation Initiative (IBSI) [20,23,24]. The settings used for feature extraction can be found in the Supplemental File SF1a, the IBSI reporting guidelines and the checklist in SF1b.

93 features were extracted: 18 first-order features (energy, total energy, entropy, kurtosis, maximum, minimum, mean, median, interquartile range (IQR), skewness, range, mean absolute deviation (MAD), robust mean absolute deviation (RMAD), root mean squared (RMS), variance, uniformity, 10th percentile, and 90th percentile) as well as 75 second- and higher-order features (24 gray level co-occurrence matrix (GLCM) features, 14 gray level dependence matrix (GLDM) features, 16 gray level run-length matrix (GLRLM) features, 16 gray level size zone matrix (GLSZM) features, and five neighboring gray tone difference matrix (NGTDM) features [20]). Shape features were not considered since ROI sizes intentionally varied.

### 2.5. Statistical Analysis

We used R for the statistical analysis (version 4.0.3, R Foundation for Statistical Computing) [25]. All analyses were done scanner-wise and subsequently compared.

We applied a pairwise Mann–Whitney U (MWU)-test with Bonferroni correction to evaluate differences between the ROI sizes. All possible pairs were tested (10 vs. 20 mm, 10 vs. 30 mm, and 20 vs. 30 mm). A *p*-value < 0.05 was considered to indicate statistical significance.

Overall concordance correlation coefficients (OCCCs) for agreement of continuous measures according to Lin et al. [26] and Barnhart et al. [27] were calculated using the epiR package for R [28]. We chose to use the OCCC as an equivalent to the generalized CCC [27] to measure agreement between more than two variables of interest (i.e., three ROI sizes). Concordance coefficient values range from 1 to −1, 1 indicating complete agreement and −1 indicating reverse agreement [29]. OCCCs ≥ 0.90 were considered to show excellent reproducibility, according to reported studies [8,29].

We calculated OCCCs twice. Once we assessed agreement among the ROI sizes 10, 20, and 30 mm (OCCCs_10–30_) and once for the ROI sizes 20 and 30 mm (OCCCs_20,30_). By excluding the smallest 10 mm ROIs, we intended to analyze if a small ROI size degrades the results.

## 3. Results

### 3.1. MWU-Test

For T1w images, features that concordantly did not show significant differences across all ROI sizes on all scanners were mean, median, RMS, 10th percentile, 90th percentile, maximum, glcm_correlation, glcm_imc2, and glcm_mcc. All other features were significantly different in at least one tested combination of ROI sizes.

For T2w images, features that concordantly did not show significant differences across all ROI sizes on all scanners were mean, median, RMS, 10th percentile, and 90th percentile.

In addition, when we tested the 20 and 30 mm ROIs only while leaving out the 10 mm ROIs, 25 additional features were not significantly different from T1w images and 32 additional features from T2w images. The summary of the MWU results on the different scanners in Supplementary File 2 gives an overview (see Table SF2).

**Figure 2:**
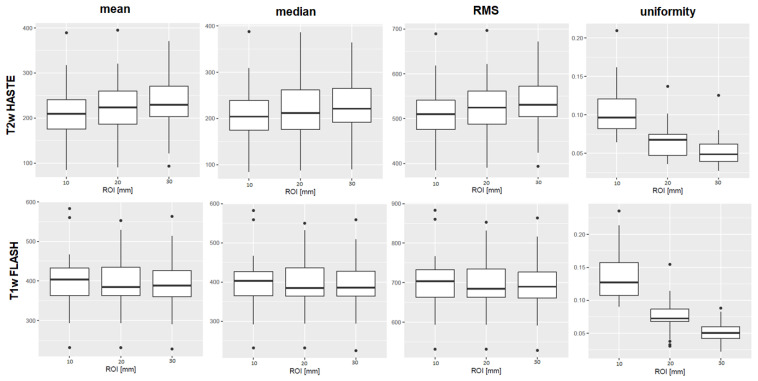
Boxplots of the first-order features mean, median, root mean squared (RMS), and uniformity are shown as examples of the results. The ROI diameters 10, 20, and 30 mm are compiled on the x-axis, the numerical value of the feature on the y-axis. The upper row shows results derived from T2w TSE HASTE images, the lower row from T1w GRE FLASH images, both from 3 Tesla I. For mean, median, and RMS, there was no significant difference between the ROI sizes in the MWU-test. The first-order feature uniformity, as an example, differed significantly in both T1w and T2w sequences. Boxplots of all features, sequences and scanners are provided in the Supplementary File SF4.

Figure 2 shows boxplots of the features mean, median, RMS, and uniformity derived from T1w and T2w sequences from the 3 Tesla I MR scanner.

The results of all MWU tests between 10 vs. 20, 20 vs. 30, and 10 vs. 30 mm 3D ROIs are listed in Supplementary File 3 (see Table SF3). Boxplots of all MWU results are shown in Supplementary File 4 (see Figure SF4).

### 3.2. OCCCs

For T1w and T2w sequences, OCCCs_10–30_ and OCCCs_20,30_ for the first-order features mean, median, and RMS concordantly showed excellent agreement in all possible combinations and on all scanners. OCCCs_10–30_ and OCCCs_20,30_ for 90th percentile and 10th percentile were excellent in almost all combinations, except for OCCC_10–30_ of 10th percentile from T1w images acquired on the 3 Tesla I and for OCCC_10–30_ of 90th percentile from T2w images acquired on the 1.5 Tesla scanner.

Among the OCCCs_10–30_, we observed no excellent agreement considering second- and higher-order features across all scanners. In the OCCCs_20,30_, several second- and higher-order features showed excellent agreement in T1w and T2w sequences: eight features on the 3 Tesla Scanner I and nine on the 3 Tesla Scanner II with an overlap of six features between scanners. For these six features, there was neither excellent agreement in the OCCCs_10–30_ nor in the OCCCs_20,30_ on the 1.5 Tesla Scanner. A summary of the OCCC results on the different scanners is provided in Supplementary File 5 (see Table SF5).

**Figure 3:**
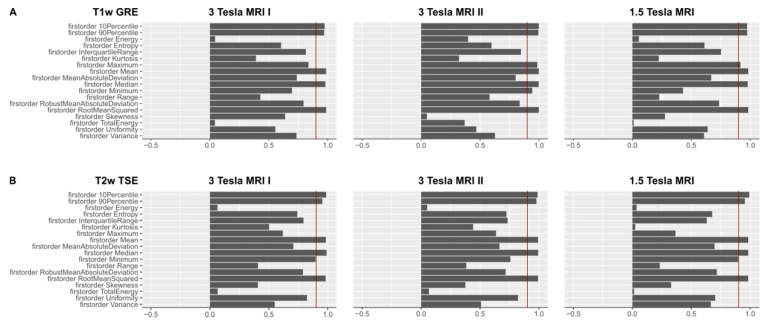
(**A**). OCCCs_20,30_ of the first-order features derived from T1w images are shown for the three different MR scanners. The red benchmark at 0.9 indicates excellent agreement in the OCCCs. Mean, median, RMS, 90th, and 10th percentile showed excellent agreement on all three MR scanners. On the 3 Tesla MR scanner II, also maximum and minimum achieved excellent agreement in the OCCCs. Mean, median, and RMS also showed excellent agreement in the OCCCs_10–30_ (not shown), whereas excellent agreement of the 90th and 10th percentile was inconsistent. A summary of the numerical results of the OCCCs on different scanners and pulse sequences is listed in Supplementary Material 5 (see Table SF5). Complete figures of OCCCs of all features, scanners, and sequences are shown in the Supplementary Files 6–8 (see Figures SF6–SF8). (**B**). OCCCs_20,30_ of the first-order features derived from T2w images are shown for the three different MR scanners. The red benchmark at 0.9 indicates excellent agreement in the OCCCs. Mean, median, RMS, 90th, and 10th percentile showed excellent agreement on all three MR scanners. On the 3 Tesla MRI I and the 1.5 Tesla scanner, also minimum achieved excellent agreement in the OCCCs_20,30_. Mean, median, and RMS also showed excellent agreement in the OCCCs_10–30_ (not shown), whereas excellent agreement of minimum, 90th and 10th percentile was inconsistent. A summary of the numerical results of the OCCCs on different scanners and pulse sequences is listed in Supplementary Material 5 (see Table SF5). Complete figures of OCCCs of all features, scanners, and sequences are shown in the Supplementary Files 6–8 (see Figures SF6–SF8).

Figure 3A shows OCCCs_20,30_ of the first-order features from T1w GRE images, 3B shows OCCCs_20,30_ of the first-order features from T2w TSE images on the different scanners.

For the complete figures of OCCCs_10–30_ and OCCCs_20,30_ on the 3 T scanner I see Supplementary File 6 (see Figure SF6), the 3 T scanner II see Supplementary File 7 (see Figure SF7), and the 1.5 T scanner see Supplementary File 8 (see Figure SF8). Complete numerical data of the OCCCs_10–30_ and OCCCs_20,30_ is listed in Supplementary File 9a,b (see Table SF9a,b).

## 4. Discussion

Our results show that in healthy liver parenchyma, the first-order features mean, median, and RMS were robust when the volume of the 3D ROI was altered. We observed the robustness of these features on different MR scanners, field strengths (3 Tesla and 1.5 Tesla), and MR pulse sequences (T1w and T2w). These three features seem applicable for MR radiomics studies of liver tissue without limitations to the segmented volume.

Across the varying ROI sizes, there was no excellent agreement for any other feature considering all sequences on all scanners. Thus, on the contrary, most of the other radiomic features, especially second- and higher-order features, were -confounded by volume, i.e. when we modified the 3D ROI diameter between 10 and 30 mm, the numeric results of these features differed significantly. This finding indicates a systematic difference in the quantitative values of these features caused by different ROI sizes. The applied 3D ROI diameters were defined with two intentions: (1) we intended to cover typical sizes of liver lesions. For example, a study on 516 metastatic liver lesions showed a mean size of 28 mm of liver metastases [30]. Small hepatocellular carcinomas (HCCs) show diameters of <20 mm [31,32,33]. Furthermore, many benign liver lesions such as bile duct hamartomas with usually 10–15 mm diameter [34] or most hepatic hemangiomas (diameter ≤ 20 mm) [35] would be covered by our ROI spectrum. (2) We aimed to only include liver parenchyma and exclude structures like bile ducts and blood vessels since these could influence radiomics analysis. Considering larger ROIs would have been interesting, but covering only healthy liver parenchyma with the 3D ROIs would have been impaired.

Agreement across the different segmented volumes also improved for numerous features when we excluded the smallest ROI diameter from the OCCCs. When we only considered 20 and 30 mm ROIs, 25 additional second- and higher-order features from T1w images and 32 additional second- and higher-order features from T2w images were no longer significantly different. This finding may imply a required minimum ROI diameter or at least a smaller difference of the ROI size for the stability of several features of at least 20 mm in diameter.

It is also remarkable that even though the two included 3 Tesla MR scanners were of the identical model from the same manufacturer, OCCCs were different, especially considering the T2w TSE HASTE-derived features. Within the T1w GRE FLASH-derived features, there was an overlap of six second- and higher-order features with an excellent agreement in the OCCCs. Since these features did not show excellent agreement on the 1.5 Tesla scanner, they cannot be considered reproducible in general. Ammari et al., who investigated radiomic features from MRI scans of homogenous and heterogenous brain phantoms and healthy volunteers’ brains with different field strengths (1.5 T and 3 T), observed significant differences in most radiomic features with altering field strength [16]. As a plausible explanation for the features’ differences between the different field strengths, they state increasing signal-to-noise ratio with increasing field strength, which is, however, also influenced by the entire signal acquisition system of the scanner and influenced by voxel size [16].

The developers of the PyRadiomics software applied in our study designate the feature root mean squared as volume-confounded. RMS is the square root of the mean of all squared intensity values and a measure of the magnitude of the image values. Volume confounding increases when the intensities are shifted to prevent negative values from being squared [20]. In our study, RMS showed reproducible values in all settings: across different segmentation volumes, on different scanners with different field strength, and when derived from T1w and T2w MRI sequences. In 2015, a radiomics study with non-contrast CT images to differentiate healthy liver parenchyma from diffuse liver diseases by segmenting the whole liver found RMS as a powerful, decisive feature with convincing AUCs [36]. Fusco et al. presented a quantitative imaging decision support tool for predicting RECIST response in lung carcinomas and described RMS as a robust feature correlating with lesion size changes in CT scans [37].

Our findings concerning mean, median, and RMS as robust features align with a recent phantom study investigating radiomic features’ differences in 3D ROI size variations in CT and MRI in a water phantom [21]. As mentioned above, Roy et al. investigated the influence of tumor volume, noise, and resolution on MR-derived radiomic features in breast cancer tumors [19]. They found second- and higher-order features more prone to noise, which might be a possible explanation for our findings. Moreover, Roy et al. detected 16 of 48 features to be volume-confounded by different tumor sizes and tried to correct for that by modeling the data. Mean turned out as a robust parameter in their study, congruent to our results, whereas median and RMS were not extracted. Nevertheless, they highlighted dependency on volume as a significant consideration in the design of imaging studies with radiomic analysis as an endpoint [19]. Rai et al., who investigated the stability of radiomic features on eight different MR scanners in a phantom study, observed that features of first-order statistics are more stable across scanners [15].

Several studies attempted to determine radiomics signatures of focal liver lesions. For example, Ding et al. suggested MR-based radiomics to differentiate hepatocellular carcinoma from focal nodular hyperplasia applying a radiomics model with eight selected features [38]. Yang et al. attempted modeling radiomic features of hepatocellular carcinomas to detect poorly differentiated tumors [39]. Both studies delineated the ROIs around the tumors slice by slice. Since such studies ultimately examine pathologic liver parenchyma, it remains unclear whether the segmented volumes influenced the results. However, a normalization against radiomics of healthy liver parenchyma would be desirable. Van Timmeren et al. suggested selecting only the repeatable and reproducible features and pointed out the demand for standardization for radiomics [40].

Our study has some limitations. One is that, our study group is relatively small. A larger study group would have been desirable to emphasize our results. Also, the field of view of every included examination was adapted to the individual patient’s size resulting in different voxel sizes. Different voxel sizes following variation in matrix and field of view size are considered to influence many radiomic features [16,19]. Since adaption of the FOV to the patient’s size is inevitable in clinical routine imaging, features sensitive to voxel size would only be applicable with a normalization algorithm, as proposed by Shafiq-Ul-Hassan in CT images [41]. Another, more general problem of texture analysis and radiomics is that the impact of different software on the features’ numerical values is not yet fully understood [11]. Ultimately, studies that state robust radiomic features require validation by feature extraction with additional software packages, as proposed by Lu et al. [13]. In addition, subtle, invisible parenchymal changes in the subjects’ livers which could have influenced the radiomic features, cannot be excluded entirely. It was taken into account that there was no known disease and that the liver had no morphological abnormalities (e.g., no apparent signal alterations in the spoiled gradient echo sequences, which excluded fatty degeneration or iron deposition). Nevertheless, in a study focusing on focal liver lesions, one would also expect these parenchymal changes between individuals. When considering diffusely altered liver parenchyma, for example, in liver cirrhosis, parametric maps of appropriate features could allow detection of subtle focal lesions [42].

## 5. Conclusions

In summary, the first-order features mean median and RMS are robust across varying 3D ROI diameters between 10–30 mm in healthy liver parenchyma in T1w and T2w MR images on three different MR scanners. Although further influencing factors such as software and reconstruction algorithm should not be omitted, the features mean, median and RMS seem to be reproducible, reliable, and broadly applicable to radiomic studies of liver MRI. Other features did not obtain consistent excellent agreement among the different 3D ROI sizes in our study. The results of many features, especially for the second- and higher-order, were significantly altered when the segmented volume was varied, indicating a systematic difference of the feature quantity dependent on the segmented volume. Consequently, in radiomics studies, we need to consider differences in segmented volumes and scrutinize correlations with radiomic feature quantity.

## Figures and Tables

**Table 1:** Details of the patient population.

MRI Scanner	3 Tesla I	3 Tesla II	1.5 Tesla
66 patients without pathologic findings	25	19	22
Female patients	15	13	14
Male patients	10	6	8
Age (y)	34.32 (17–62)	28.05 (15–49)	30.86 (15–49)

**Table 2:** Details of the MRI scanning parameters.

MRI Scanner	3 Tesla I		3 Tesla II		1.5 Tesla	
Sequence	T1w GRE FLASH	T2w TSE HASTE	T1w GRE FLASH	T2w TSE HASTE	T1w GRE FLASH	T2w TSE HASTE
TR/TE (ms)	168/2.46	1000/95	168/2.46	1600/95	167/2.39	850/81
Flip angle (deg.)	70	155	70	180	70	180
Slice thickness (mm)	5	5	5	5	6	6
Spacing between slices	5.5	5.5	5.5	5.5	6.6	6.6
Pixel spacing	1.125/1.125	1.125/1.125	1.125/1.125	1.125/1.125	1.09375/1.09375	1.3671875/1.3671875
Acquisition Matrix	320/158	320/194	320/210	320/194	320/203	256/167
Number of phase encoding steps	158	124	210	124	203	111
In plane phase encoding direction	anterior-posterior					
Patient position	Head first (phased-array body coil)					
Fat-saturation	None					
Breathing regimen	Multi-breath-hold					

T1w: T1-weighted. T2w: T2-weighted. GRE: Gradient Echo. TSE: Turbo Spin Echo. FLASH: Fast Low Angle Shot. HASTE: Half Fourier Acquisition single Shot Turbo Spin Echo. TR: repetition time. TE: echo time. Deg.: degree.

## Supplementary Materials

The following are available online at https://www.mdpi.com/article/10.3390/tomography7040073/s1, Textfile SF1a: settings for feature extraction, Textfile SF1b: IBSI reporting guidelines and checklist, Table SF2: summary of MWU results on the different scanners, Table SF3: MWU results, Figure SF4: boxplots of MWU results, Table SF5: summary of the OCCC results on the different scanners, Figure SF6a: OCCCs 3 Tesla I T1 GRE ROIs 10–30 mm, Figure SF6b: OCCCs 3 Tesla I T1 GRE ROIS 20,30 mm, Figure SF6c: OCCCs 3 Tesla I T2 TSE ROIs 10–30 mm, SF6d: OCCCs 3 Tesla I T2 TSE ROIs 20,30 mm, Figure SF7a: OCCCs 3 Tesla II T1 GRE ROIs 10–30 mm, Figure SF7b: OCCCs 3 Tesla II T1 GRE ROIs 20,30, Figure SF7c: OCCCs 3 Tesla II T2 TSE ROIs 10–30 mm, Figure SF7d: OCCCs 3 Tesla II T2 TSE ROIs 20,30 mm, Figure SF8a: OCCCs 1.5 Tesla T1 GRE ROIs 10–30 mm, Figure SF8b: OCCCs 1.5 Tesla T1 GRE ROIs 20,30 mm, Figure SF8c: OCCCs 1.5 Tesla T2 TSE ROIs 10–30 mm, Figure SF8d: OCCCs 1.5 Tesla T2 TSE ROIs 20,30 mm, Table SF9a: numerical OCCC results ROIs 10–30, Table S9Fb: numerical OCCC results ROIs 20,30.

## Abbreviations

3D ROI	Three-dimensional region of interest
AUC	Area under the curve
FLASH	Fast Low Angle Shot
GLCM	Gray level co-occurrence matrix
GLDM	Gray level dependence matrix
GLSZM	Gray level size zone matrix
GRE	Gradient Echo
HASTE	Half Fourier Acquisition single-Shot Turbo Spin Echo
IQR	Interquartile range
MAD	Mean absolute deviation
NGTDM	Neighboring gray tone difference matrix
OCCC	Overall concordance correlation coefficient
OCCCs_10–30_	OCCCs to assess agreement among the 3D ROI diameters 10 mm, 20 mm, and 30 mm
OCCCs_20,30_	OCCCs to assess agreement among the 3D ROI diameters 20 mm and 30 mm
RMAD	Robust mean absolute deviation
RMS	Root mean squared
T2w	T2-weighted
T1w	T1-weighted
TSE	Turbo Spin Echo
