# Supplementary material for: Stability of Liver Radiomics across Different 3D ROI Sizes—An MRI In Vivo Study
Source: Tomography. 2021 Dec 3;7(4):866–76. doi: 10.3390/tomography7040073 (PMC8706942; doi:10.3390/tomography7040073)
Supplement: Supplementary file 1 [file tomography-07-00073-s001.zip › SF4_Boxplots_of_MWU_results.pdf]

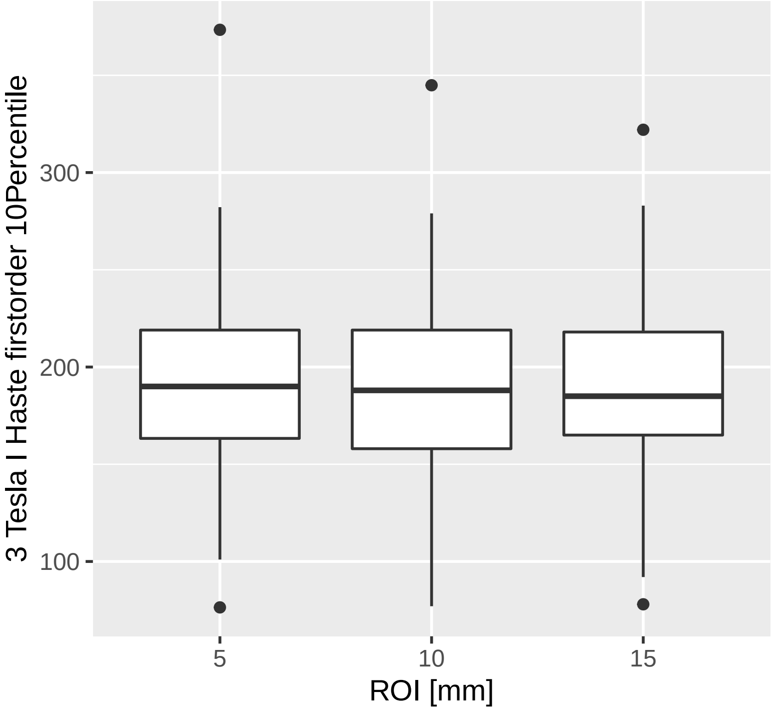
3 Tesla I Haste firstorder 10Percentile
100
200
300
5
10
15
ROI [mm]

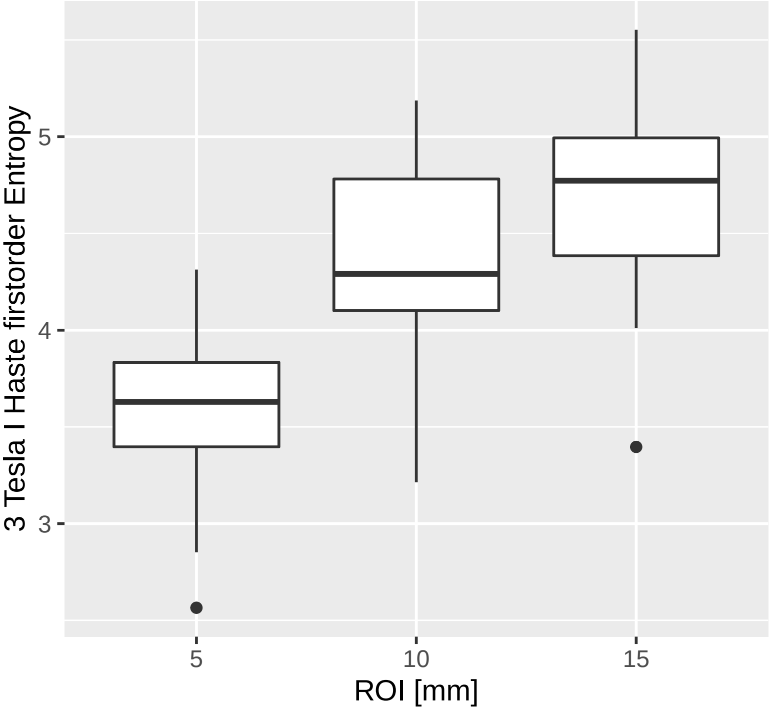
3 Tesla I Haste firstorder Entropy
3
4
5
5
10
15
ROI [mm]

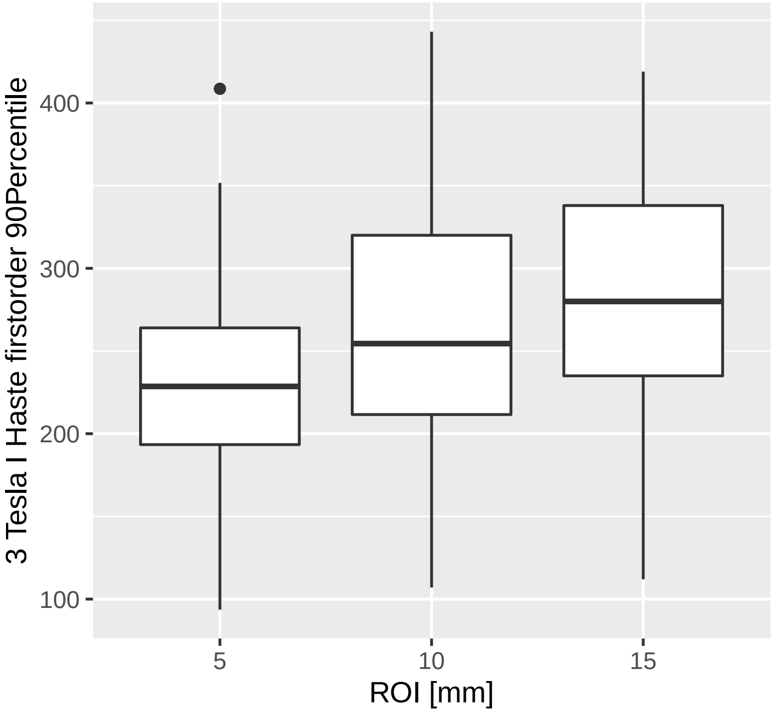
3 Tesla I Haste firstorder 90Percentile
100
200
300
400
5
10
15
ROI [mm]

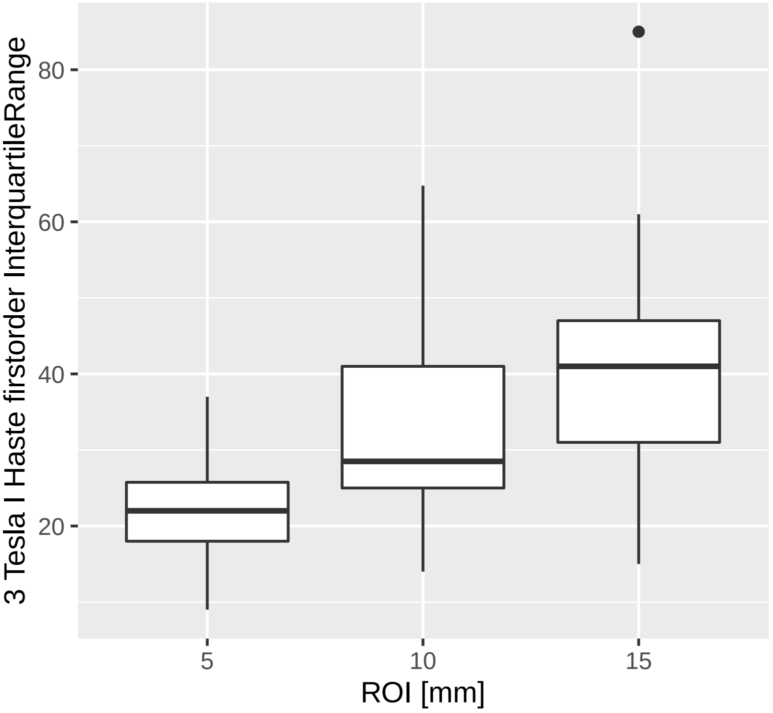
3 Tesla I Haste firstorder InterquartileRange
20
40
60
80
5
10
15
ROI [mm]

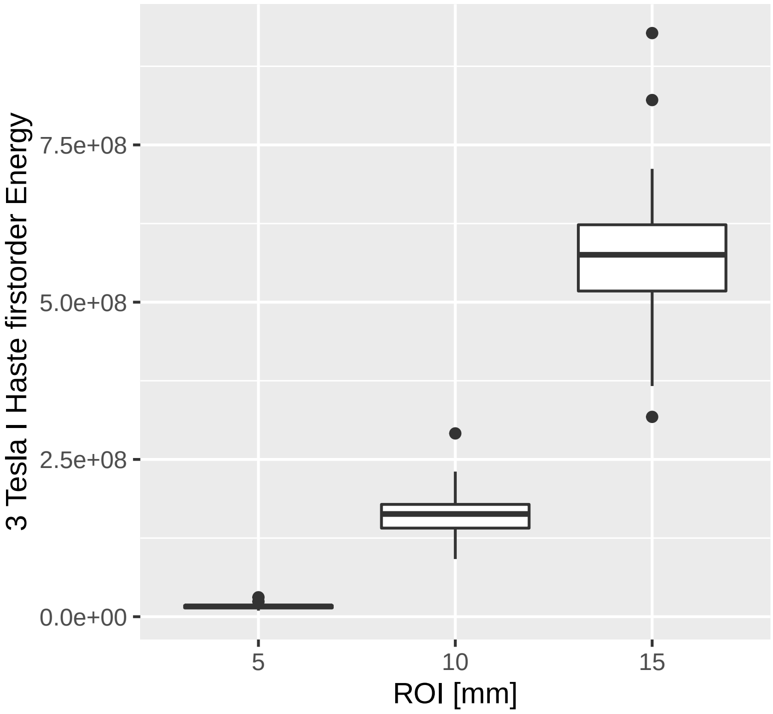
3 Tesla I Haste firstorder Energy
0.0e+00
2.5e+08
5.0e+08
7.5e+08
5
10
15
ROI [mm]

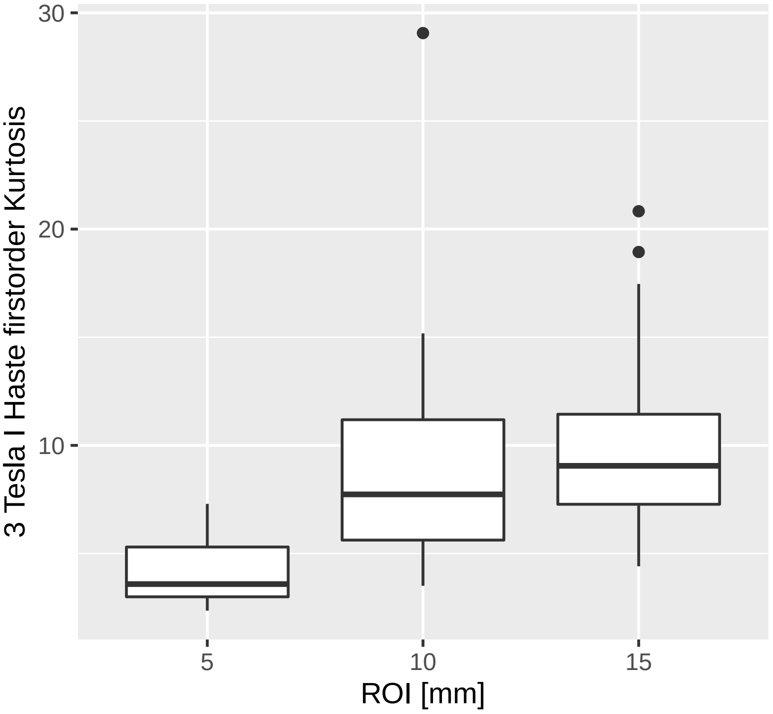
3 Tesla I Haste firstorder Kurtosis
10
20
30
5
10
15
ROI [mm]

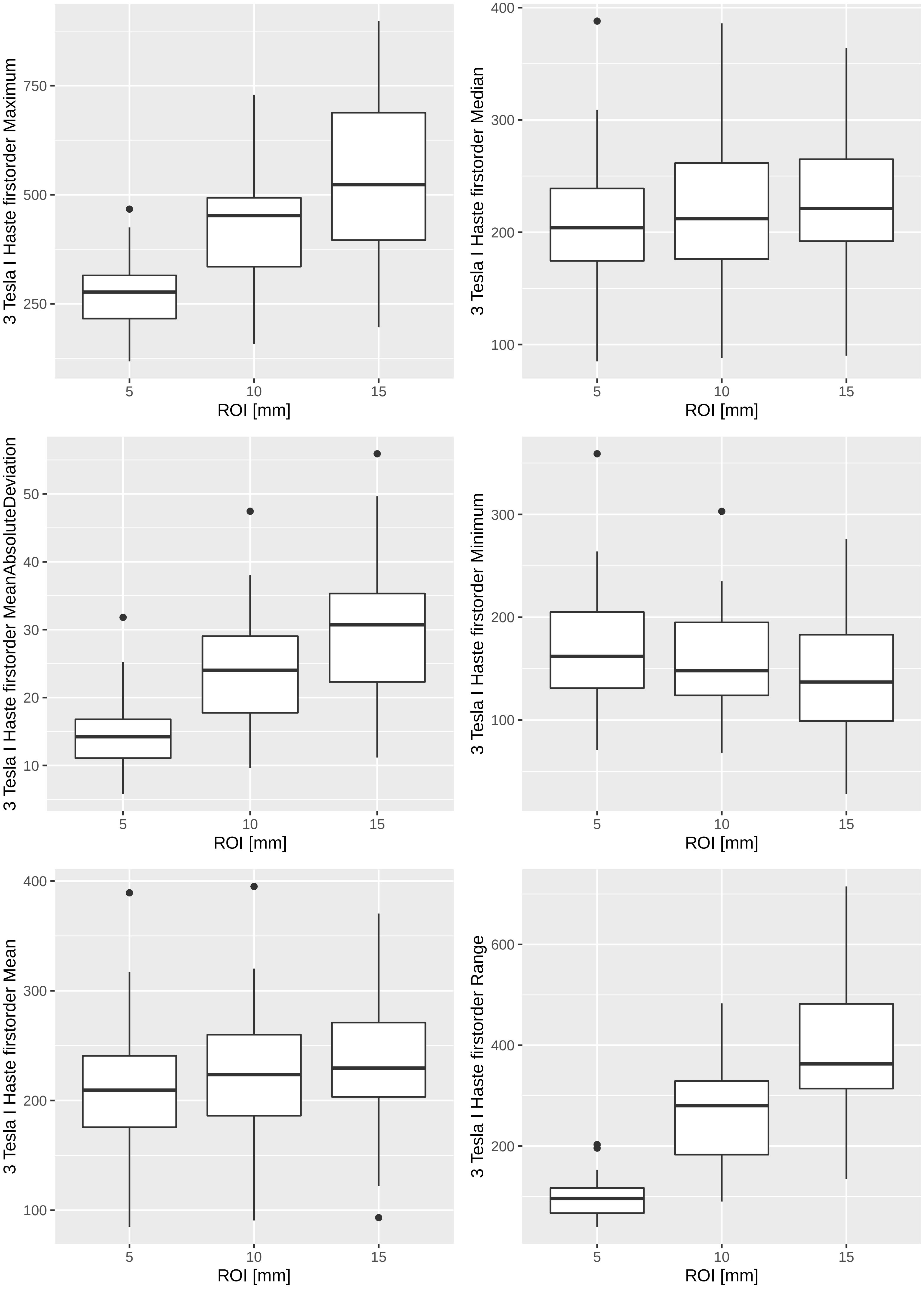
3 Tesla I Haste firstorder Maximum
250
500
750
ROI [mm]
5
10
15
3 Tesla I Haste firstorder Median
100
200
300
400
ROI [mm]
5
10
15
3 Tesla I Haste firstorder MeanAbsoluteDeviation
10
20
30
40
50
ROI [mm]
5
10
15
3 Tesla I Haste firstorder Minimum
100
200
300
ROI [mm]
5
10
15
3 Tesla I Haste firstorder Mean
100
200
300
400
ROI [mm]
5
10
15
3 Tesla I Haste firstorder Range
200
400
600
ROI [mm]
5
10
15

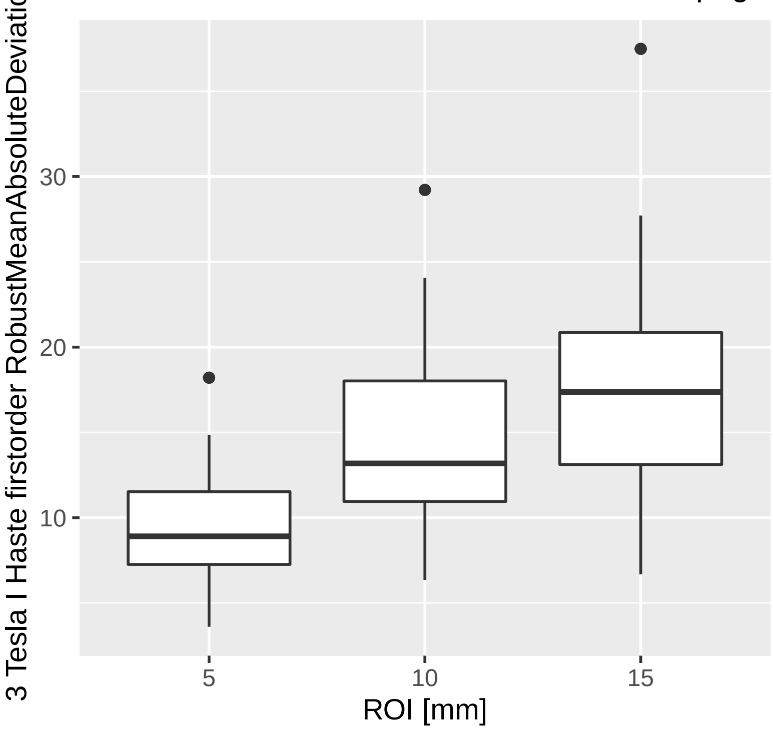
3 Tesla I Haste firstorder RobustMeanAbsoluteDeviation
30
20
10
5
10
15
ROI [mm]

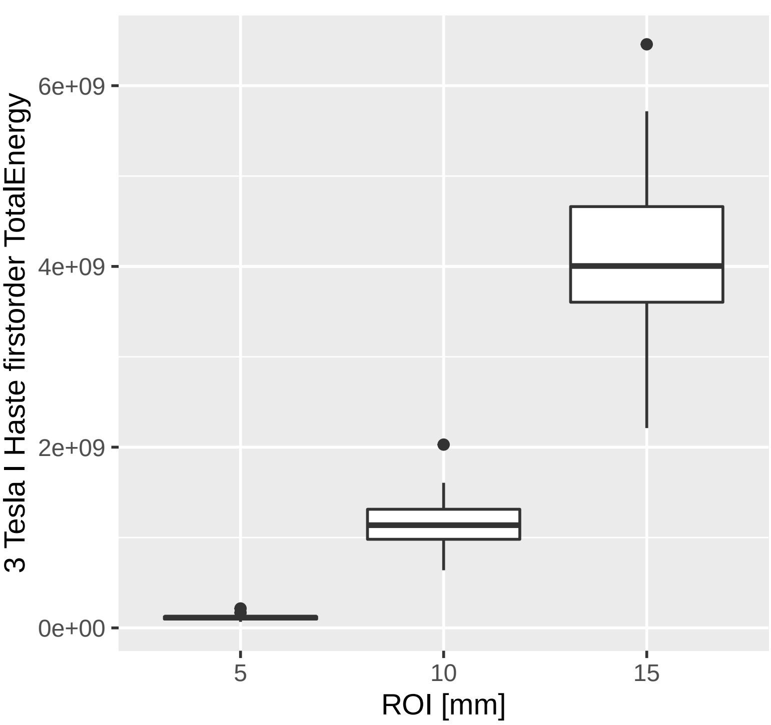
3 Tesla I Haste firstorder TotalEnergy
6e+09
4e+09
2e+09
0e+00
5
10
15
ROI [mm]

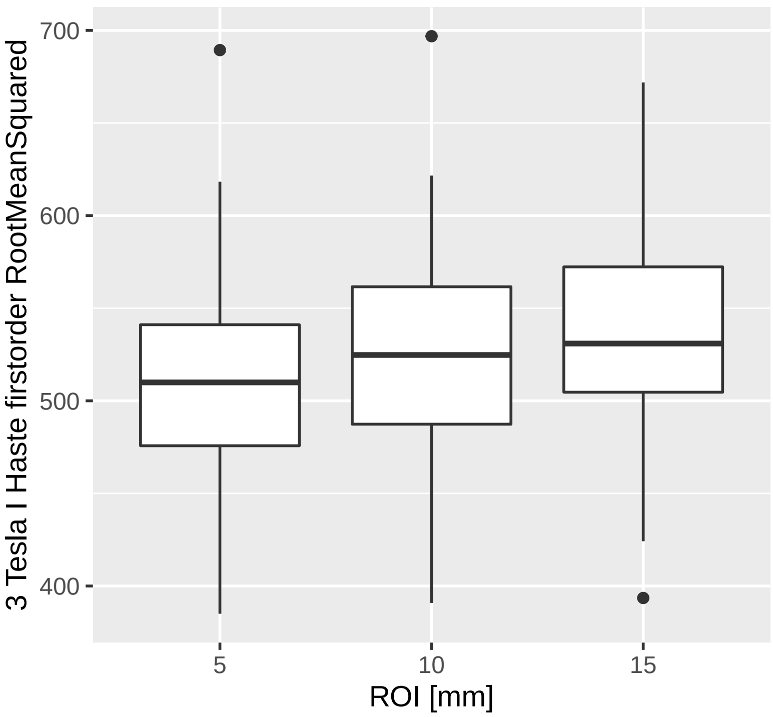
3 Tesla I Haste firstorder RootMeanSquared
700
600
500
400
5
10
15
ROI [mm]

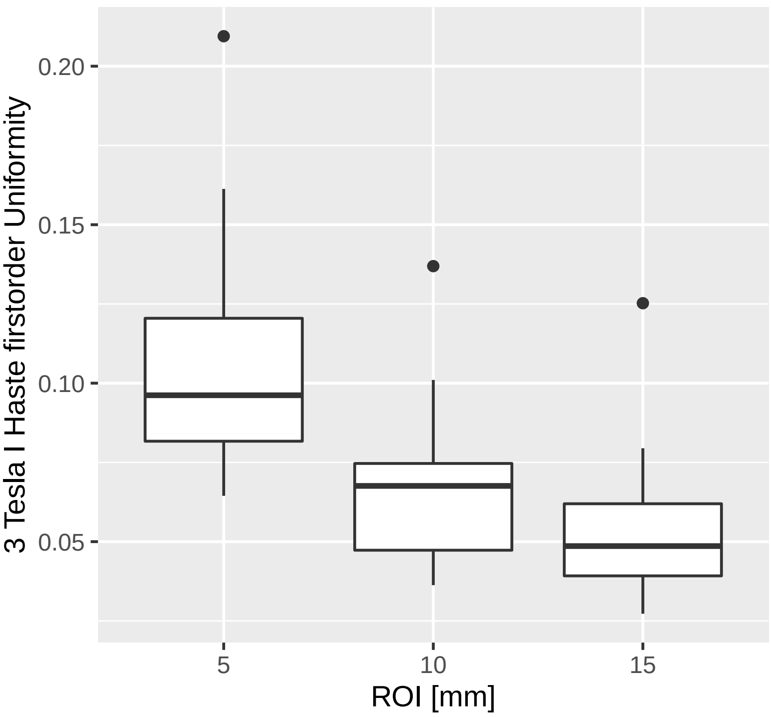
3 Tesla I Haste firstorder Uniformity
0.20
0.15
0.10
0.05
5
10
15
ROI [mm]

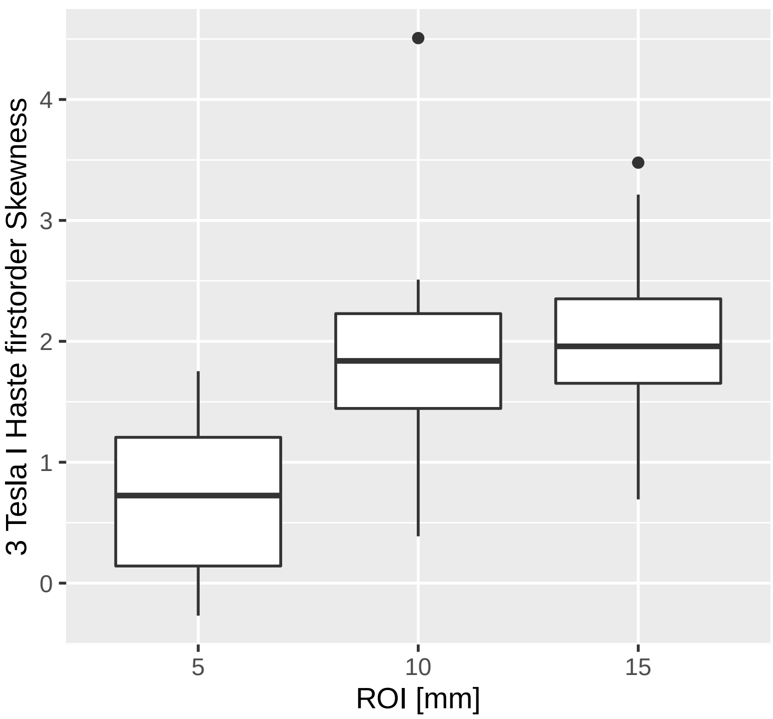
3 Tesla I Haste firstorder Skewness
4
3
2
1
0
5
10
15
ROI [mm]

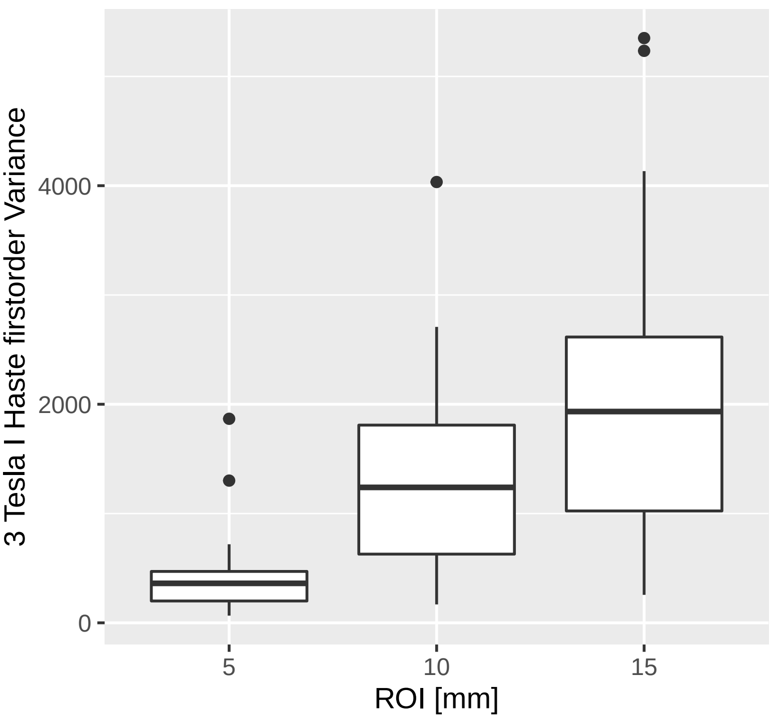
3 Tesla I Haste firstorder Variance
4000
2000
0
5
10
15
ROI [mm]

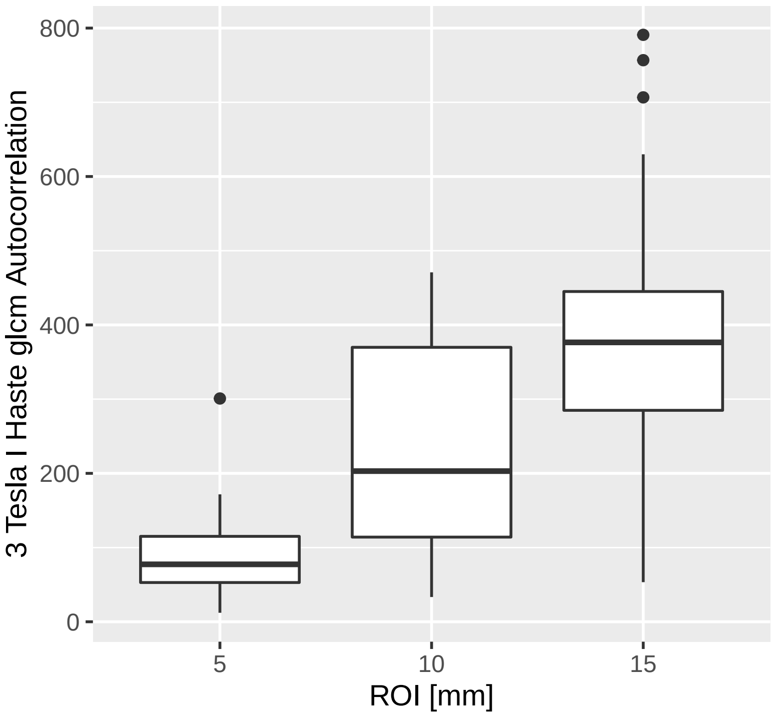
3 Tesla I Haste glcm Autocorrelation
0
200
400
600
800
5
10
15
ROI [mm]

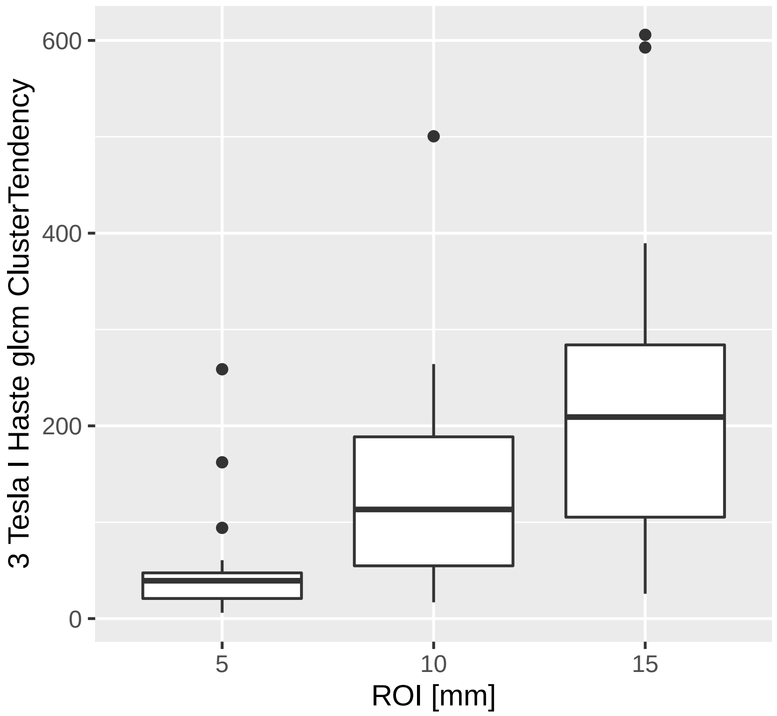
3 Tesla I Haste glcm ClusterTendency
0
200
400
600
5
10
15
ROI [mm]

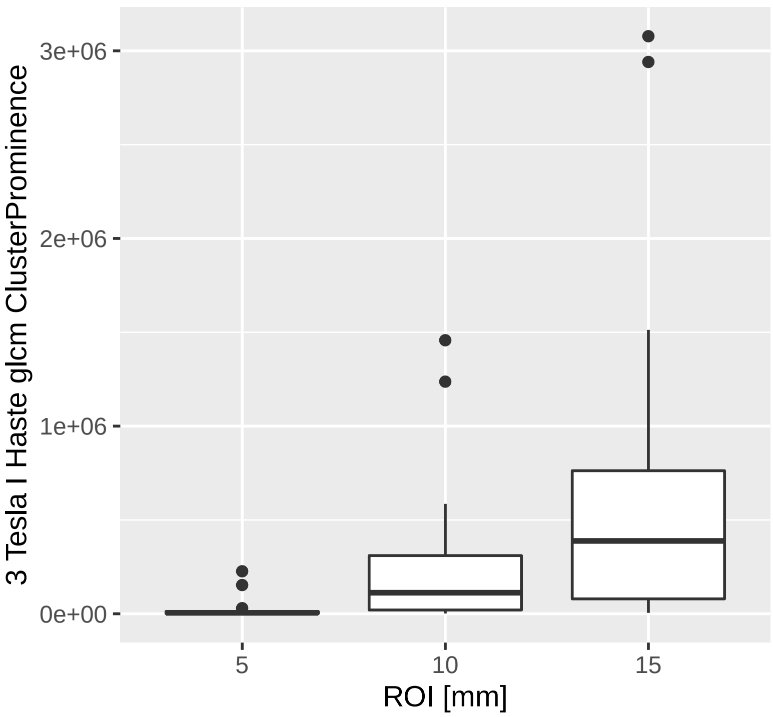
3 Tesla I Haste glcm ClusterProminence
0e+00
1e+06
2e+06
3e+06
5
10
15
ROI [mm]

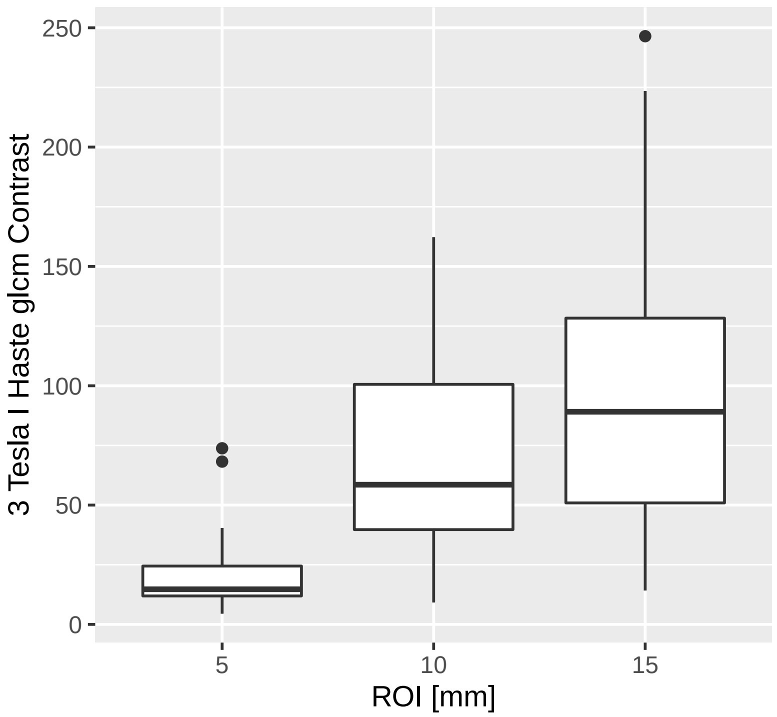
3 Tesla I Haste glcm Contrast
0
50
100
150
200
250
5
10
15
ROI [mm]

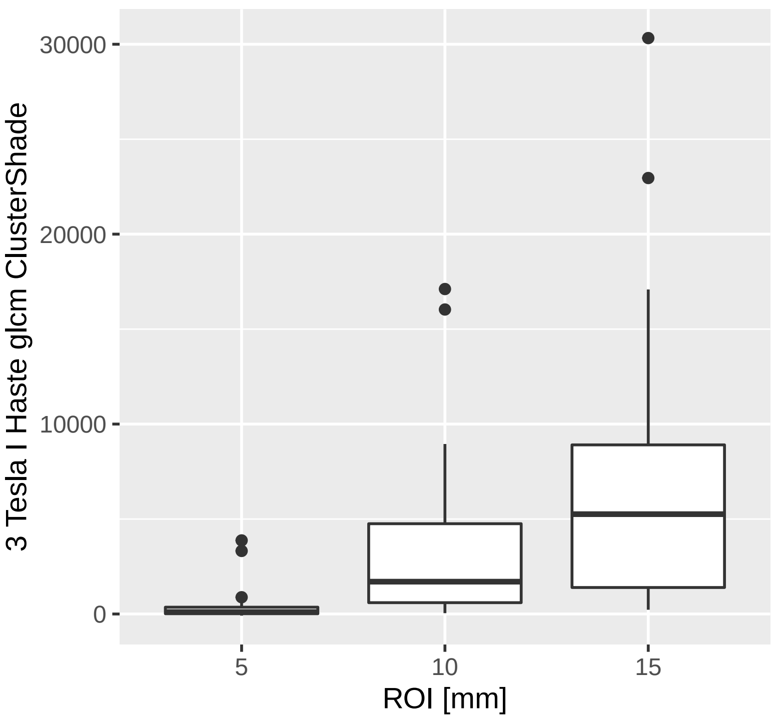
3 Tesla I Haste glcm ClusterShade
0
10000
20000
30000
5
10
15
ROI [mm]

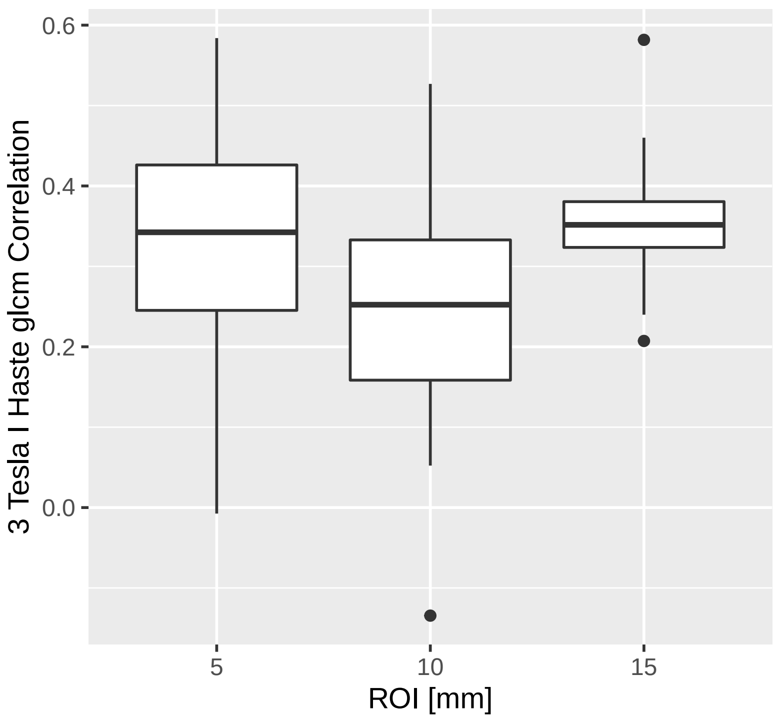
3 Tesla I Haste glcm Correlation
0.0
0.2
0.4
0.6
5
10
15
ROI [mm]

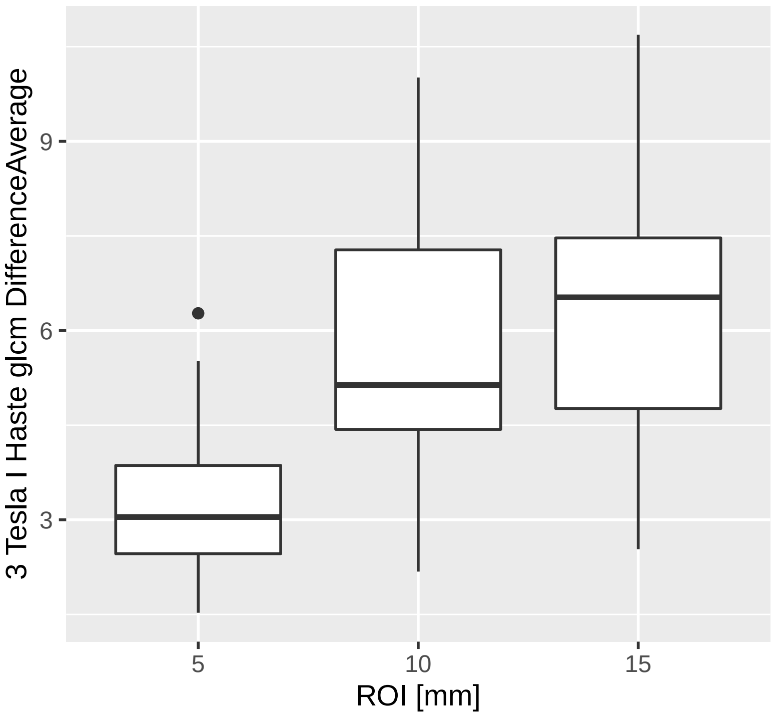
3 Tesla I Haste glcm DifferenceAverage
9
6
3
5
10
15
ROI [mm]

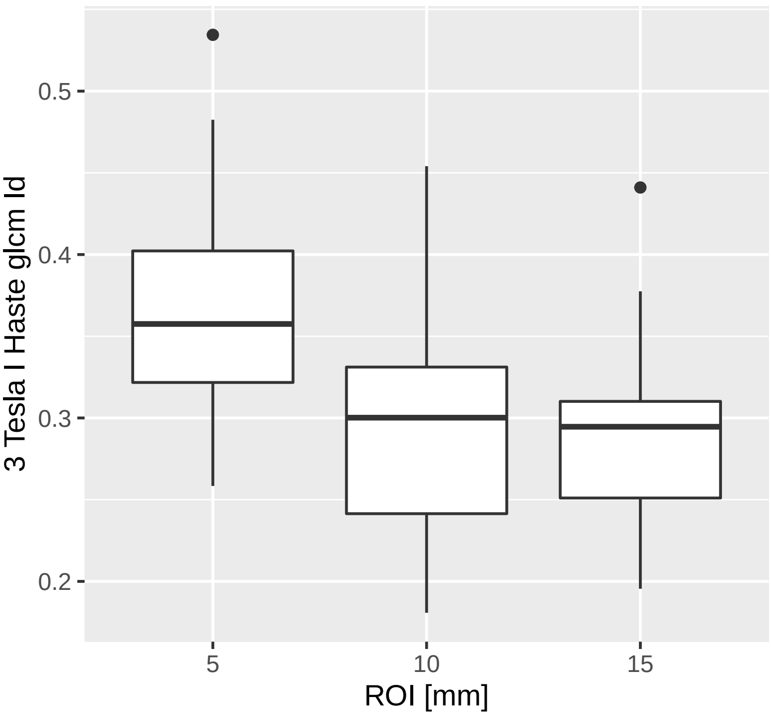
3 Tesla I Haste glcm Id
0.5
0.4
0.3
0.2
5
10
15
ROI [mm]

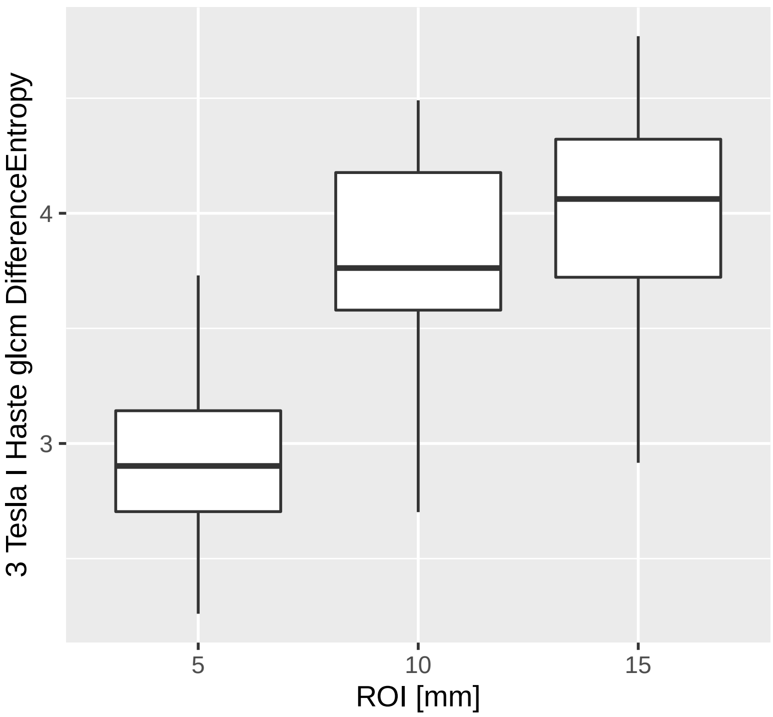
3 Tesla I Haste glcm DifferenceEntropy
4
3
5
10
15
ROI [mm]

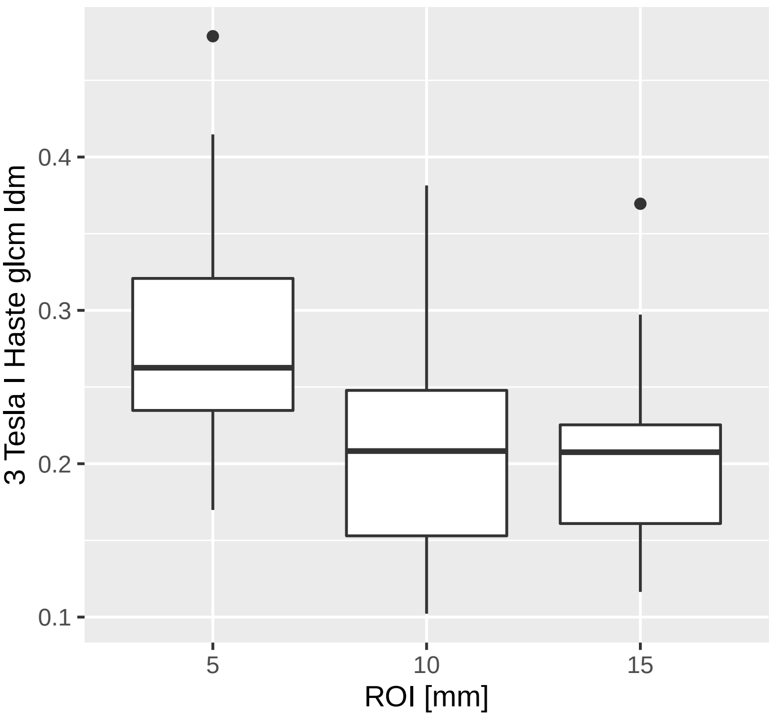
3 Tesla I Haste glcm Idm
0.4
0.3
0.2
0.1
5
10
15
ROI [mm]

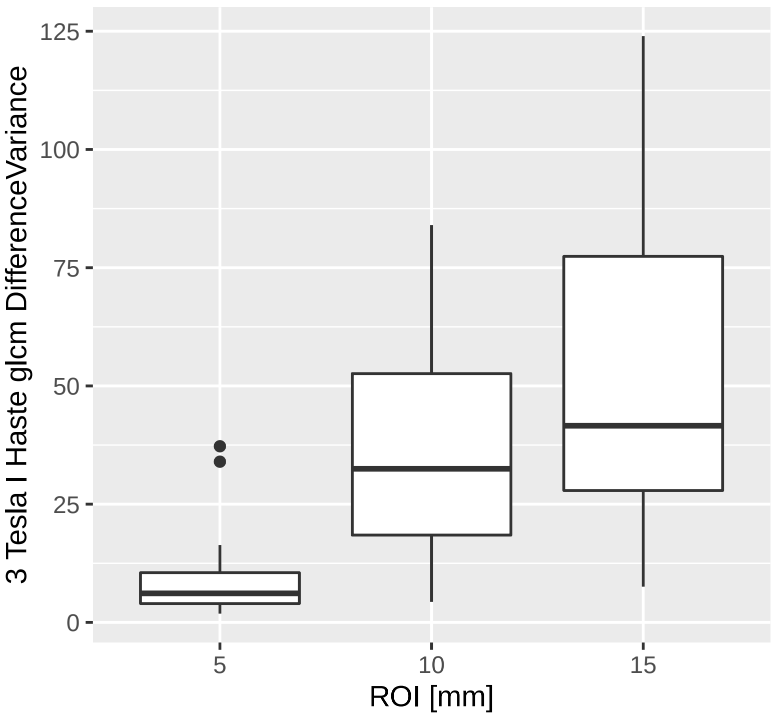
3 Tesla I Haste glcm DifferenceVariance
125
100
75
50
25
0
5
10
15
ROI [mm]

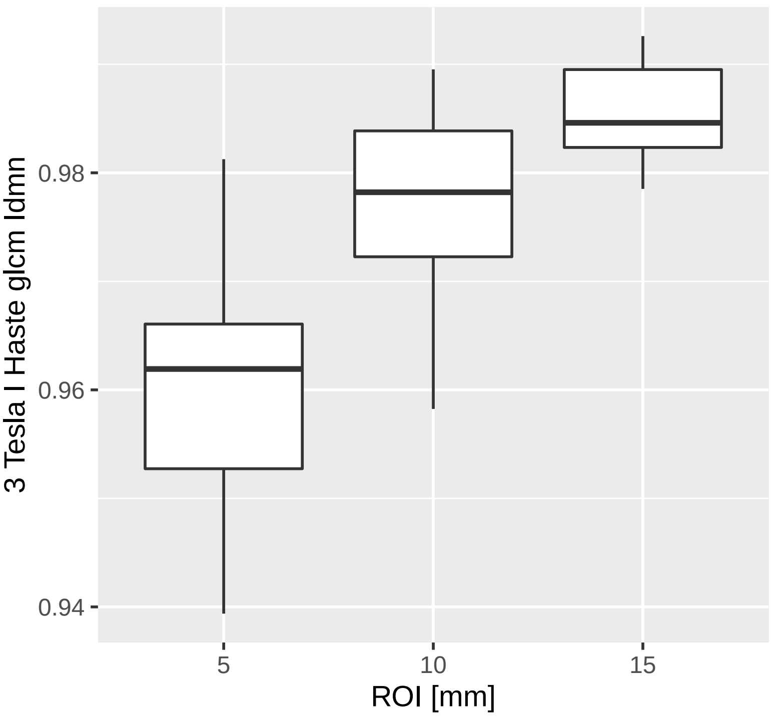
3 Tesla I Haste glcm Idmn
0.98
0.96
0.94
5
10
15
ROI [mm]

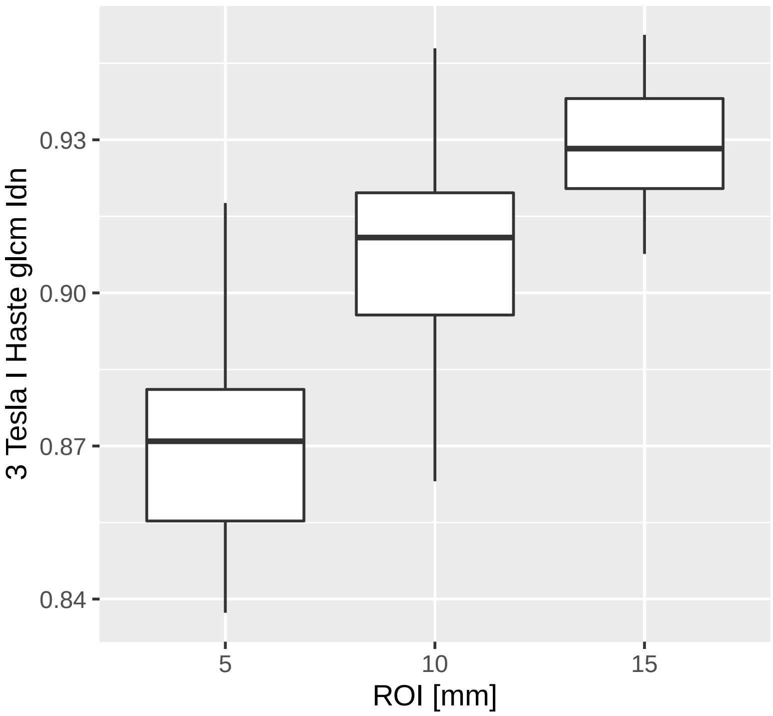

3 Tesla I Haste glcm Idn
0.93
0.90
0.87
0.84
5
10
15
ROI [mm]

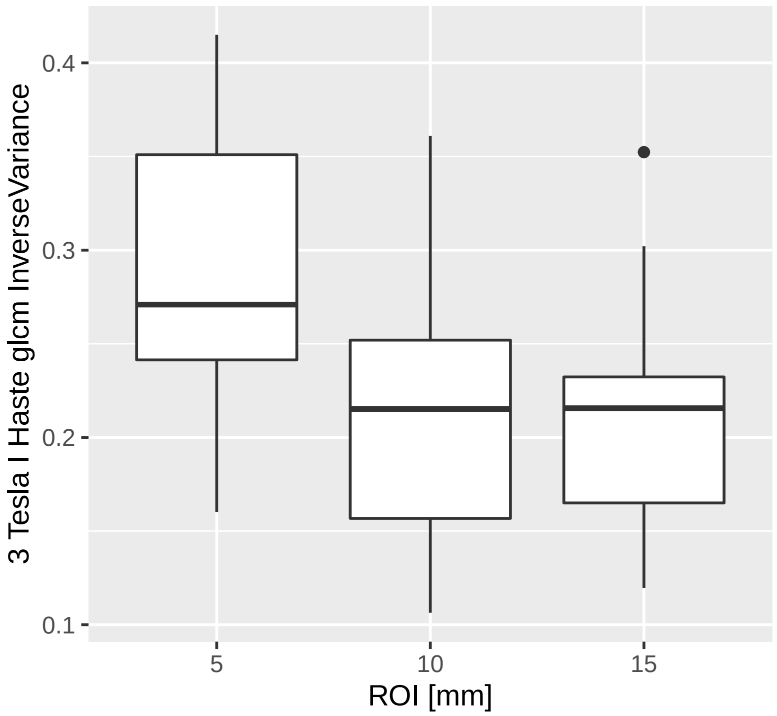

3 Tesla I Haste glcm InverseVariance
0.4
0.3
0.2
0.1
5
10
15
ROI [mm]

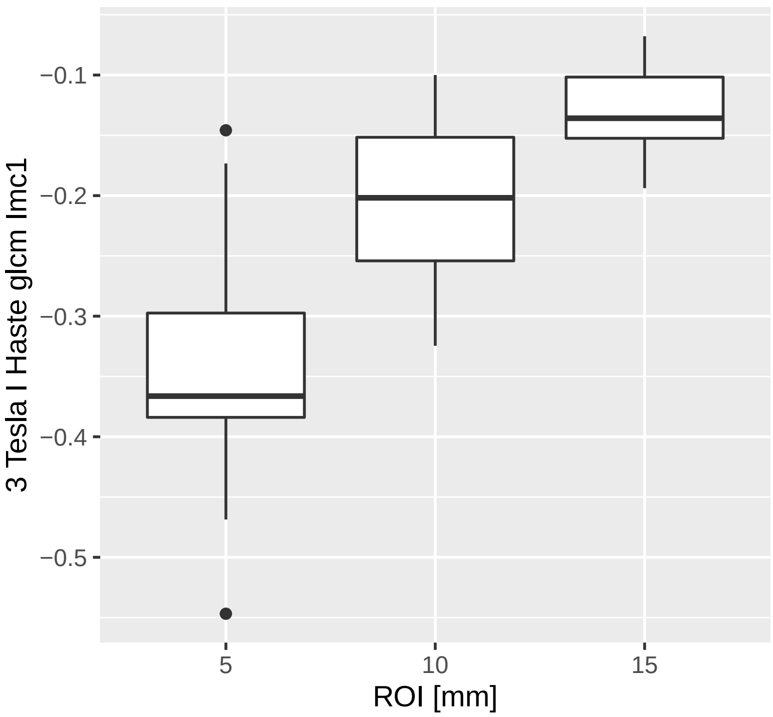

3 Tesla I Haste glcm Imc1
−0.1
−0.2
−0.3
−0.4
−0.5
5
10
15
ROI [mm]

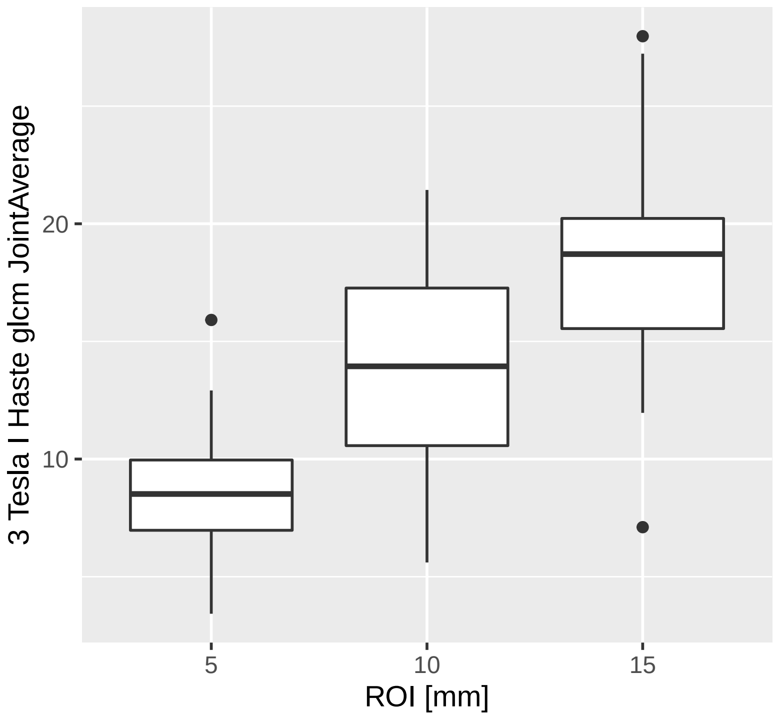

3 Tesla I Haste glcm JointAverage
20
10
5
10
15
ROI [mm]

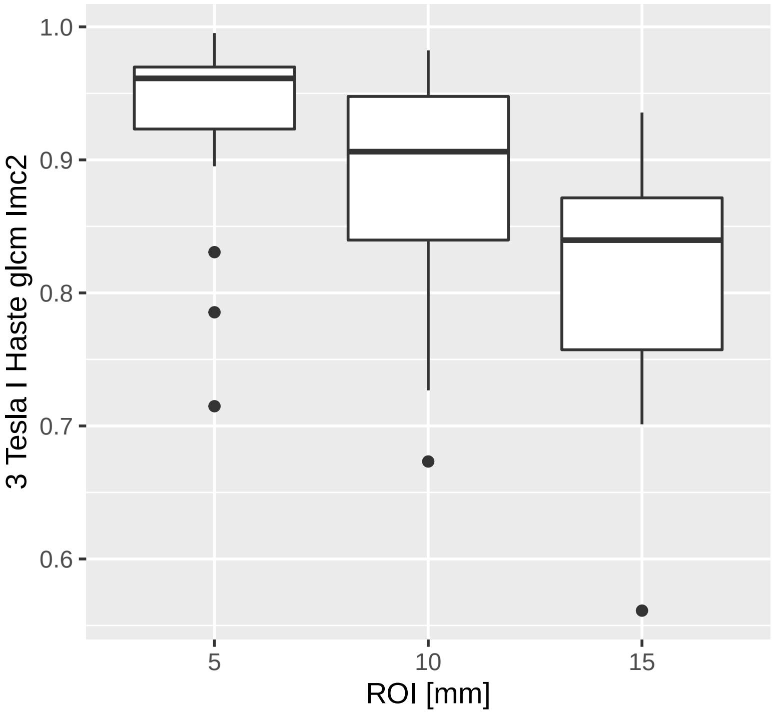

3 Tesla I Haste glcm Imc2
1.0
0.9
0.8
0.7
0.6
5
10
15
ROI [mm]

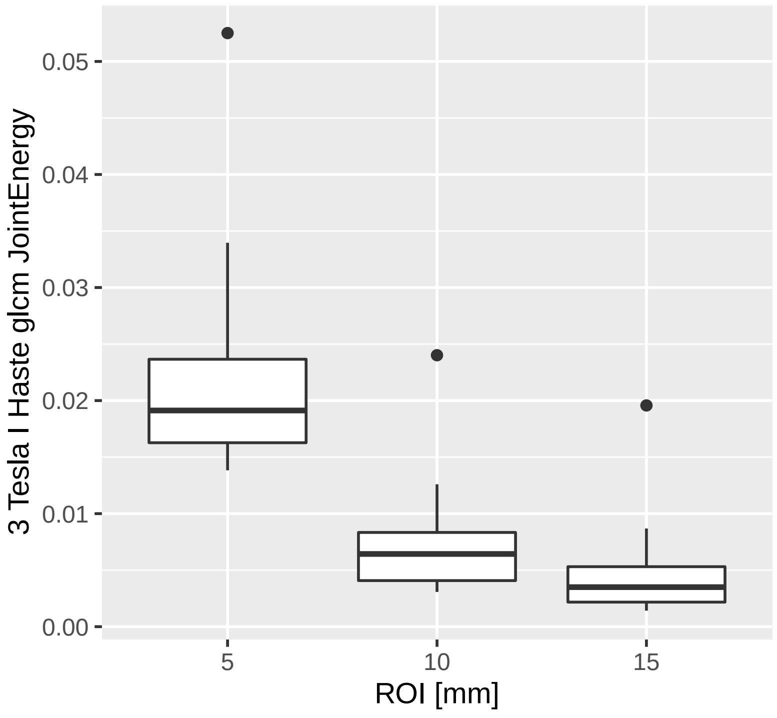

3 Tesla I Haste glcm JointEnergy
0.05
0.04
0.03
0.02
0.01
0.00
5
10
15
ROI [mm]

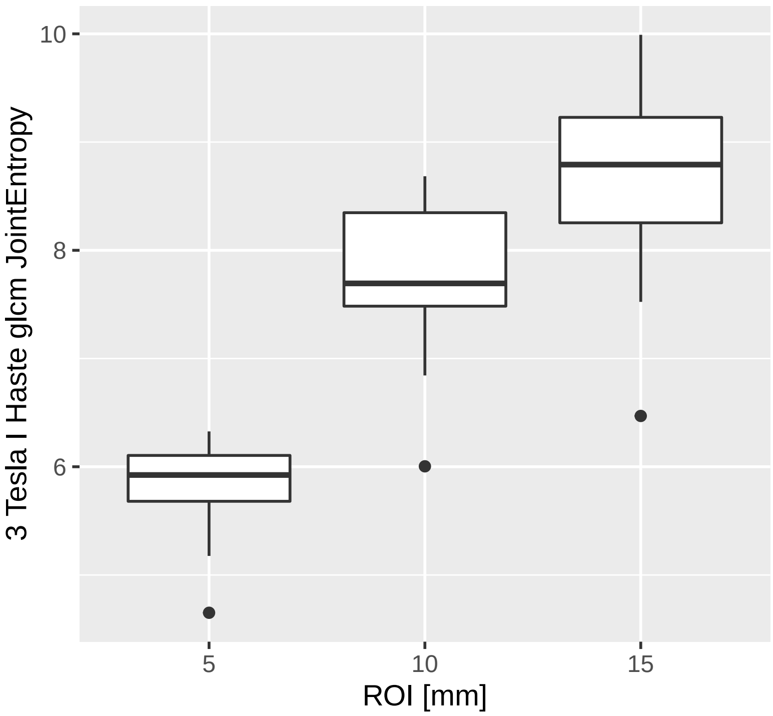
3 Tesla I Haste glcm JointEntropy
10
8
6
5
10
15
ROI [mm]

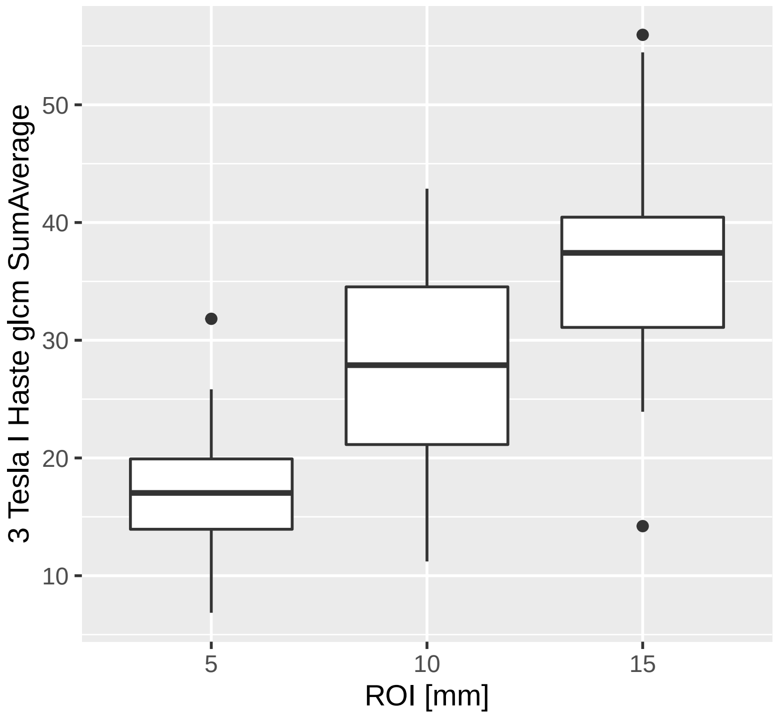
3 Tesla I Haste glcm SumAverage
50
40
30
20
10
5
10
15
ROI [mm]

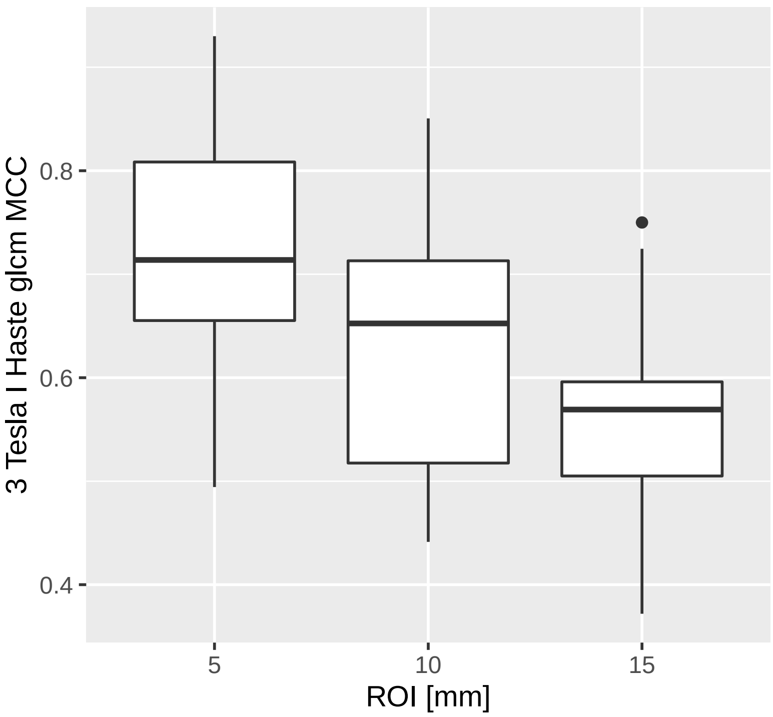
3 Tesla I Haste glcm MCC
0.8
0.6
0.4
5
10
15
ROI [mm]

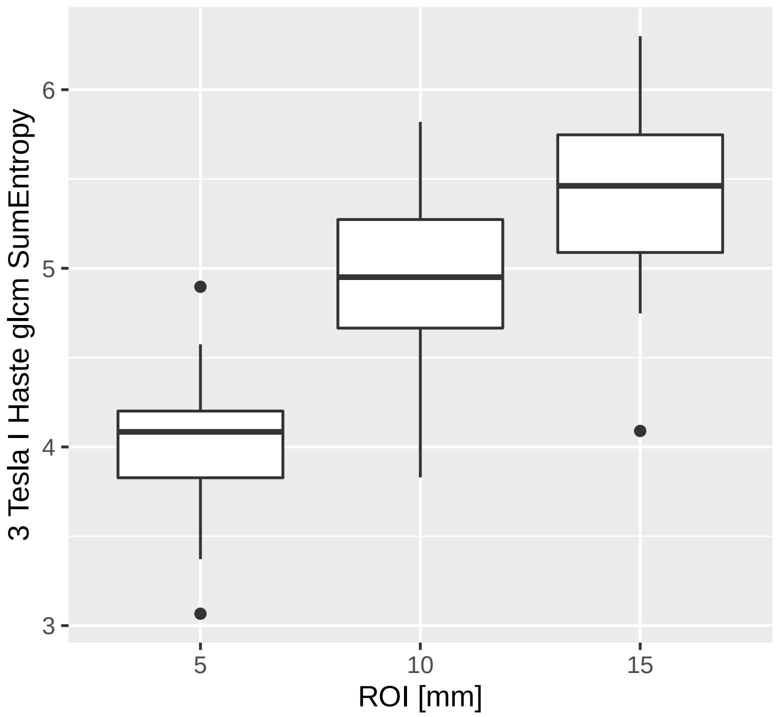
3 Tesla I Haste glcm SumEntropy
6
5
4
3
5
10
15
ROI [mm]

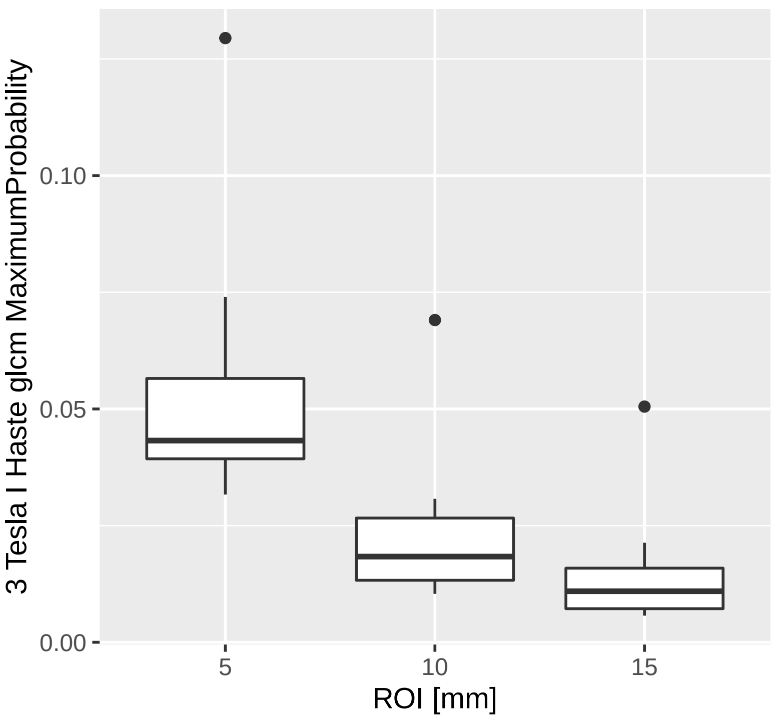
3 Tesla I Haste glcm MaximumProbability
0.10
0.05
0.00
5
10
15
ROI [mm]

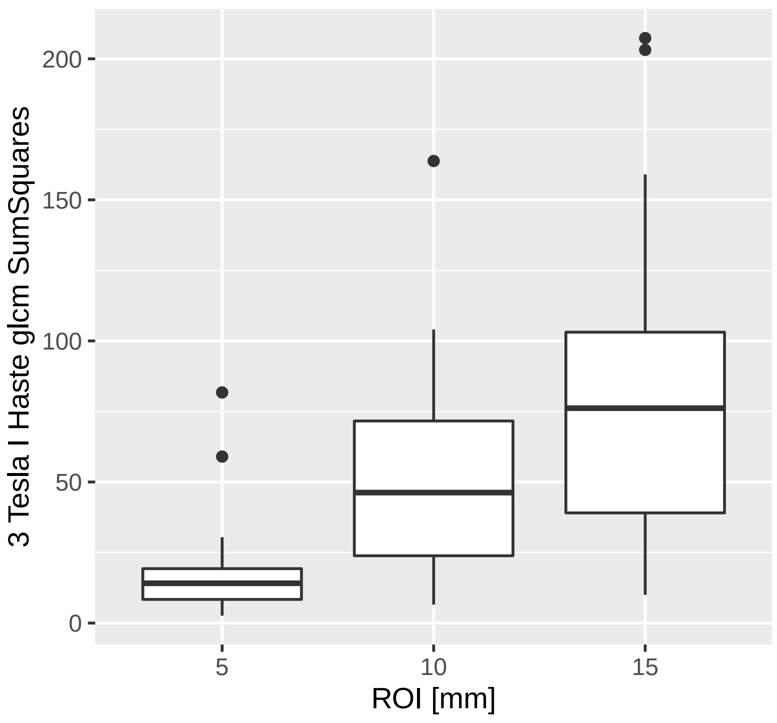
3 Tesla I Haste glcm SumSquares
200
150
100
50
0
5
10
15
ROI [mm]

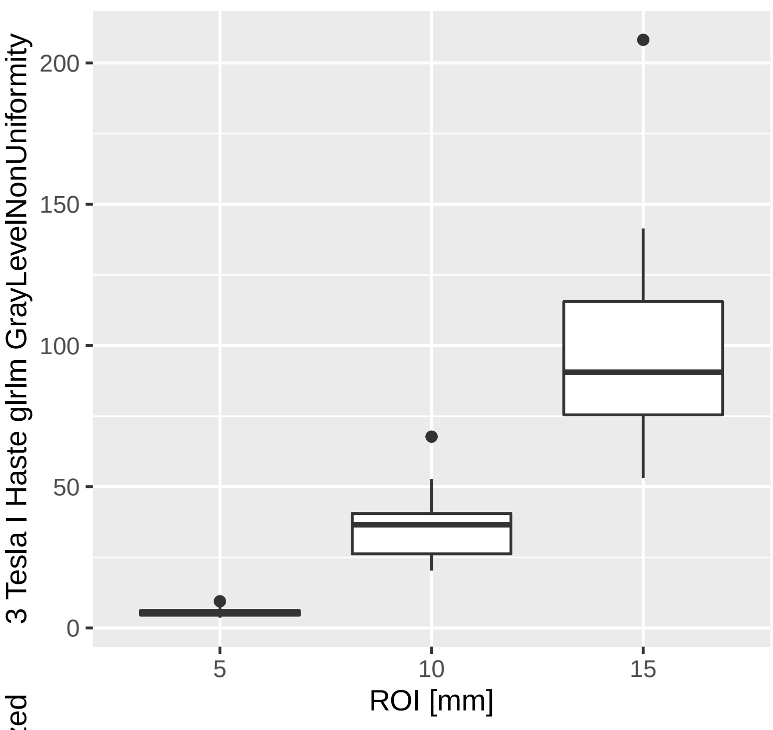
3 Tesla I Haste glrlm GrayLevelNonUniformity
0
50
100
150
200
5
10
15
ROI [mm]

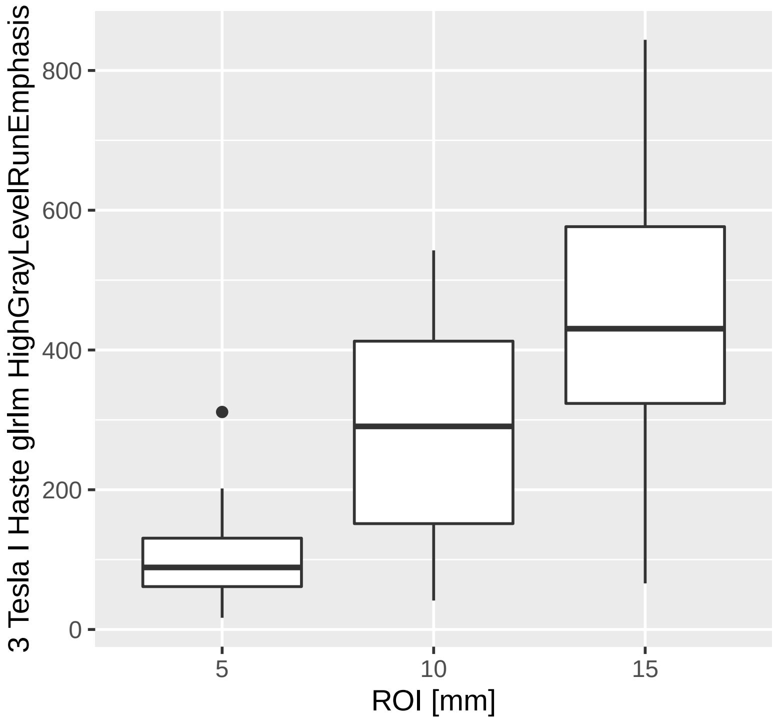
3 Tesla I Haste glrlm HighGrayLevelRunEmphasis
0
200
400
600
800
5
10
15
ROI [mm]

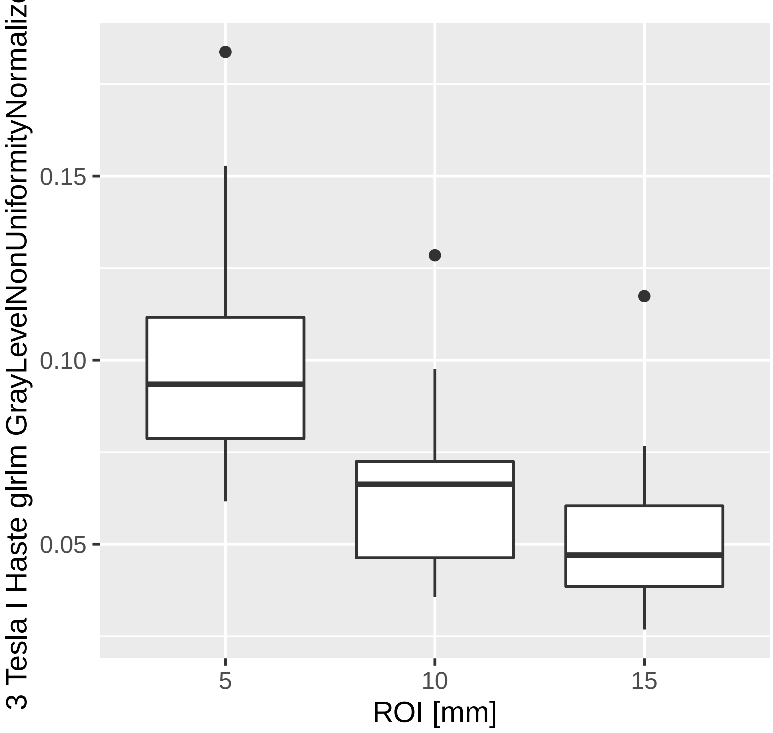
3 Tesla I Haste glrlm GrayLevelNonUniformityNormalized
0.05
0.10
0.15
5
10
15
ROI [mm]

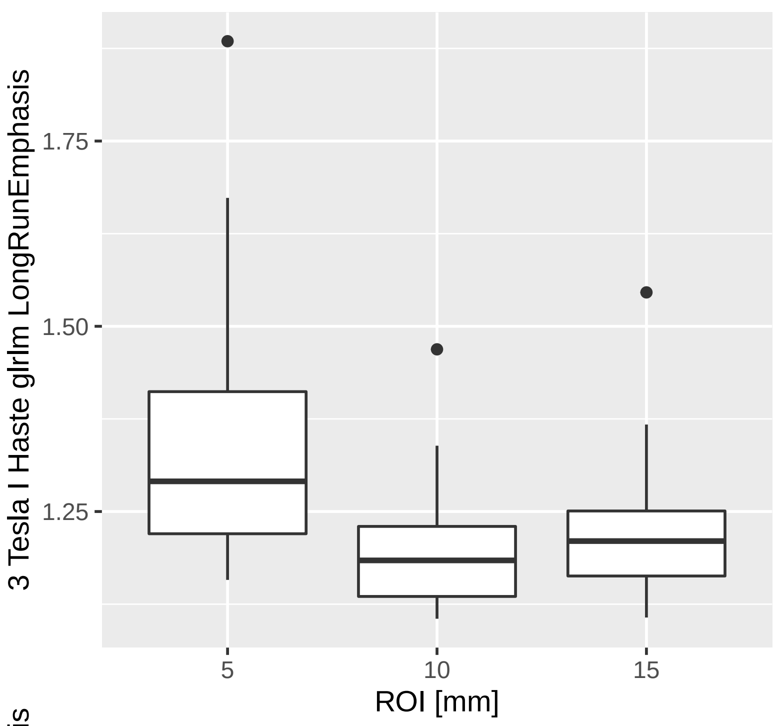
3 Tesla I Haste glrlm LongRunEmphasis
1.25
1.50
1.75
5
10
15
ROI [mm]

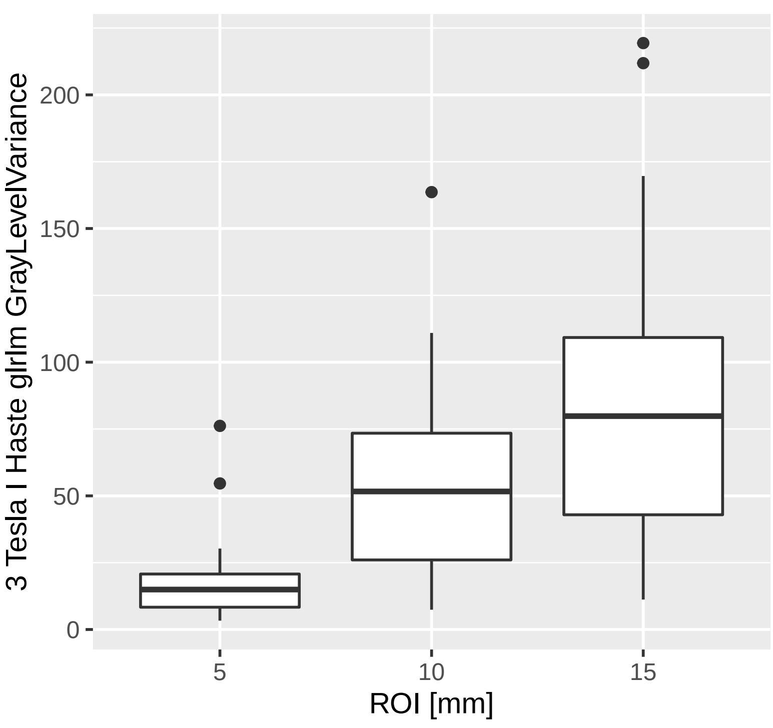
3 Tesla I Haste glrlm GrayLevelVariance
0
50
100
150
200
5
10
15
ROI [mm]

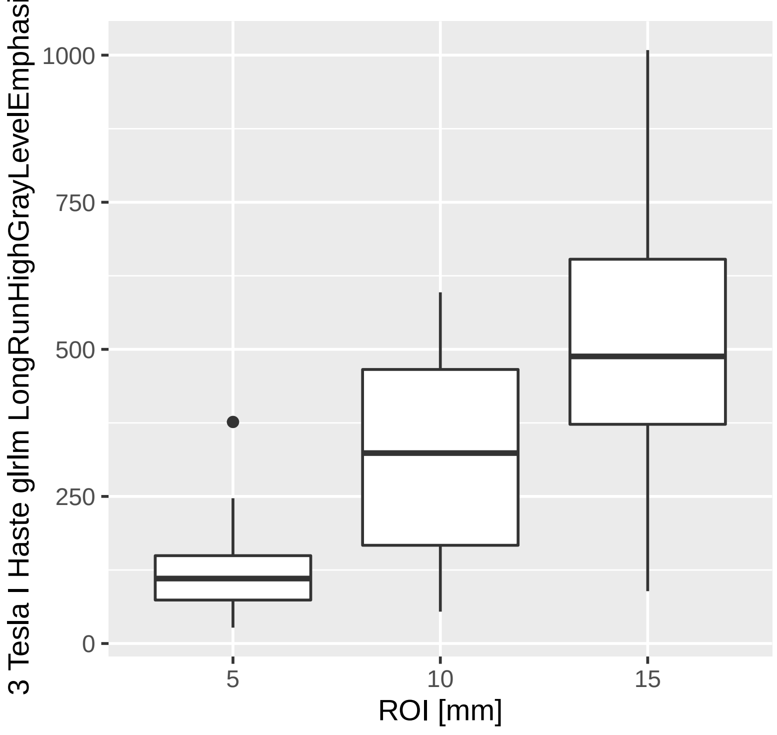
3 Tesla I Haste glrlm LongRunHighGrayLevelEmphasis
0
250
500
750
1000
5
10
15
ROI [mm]

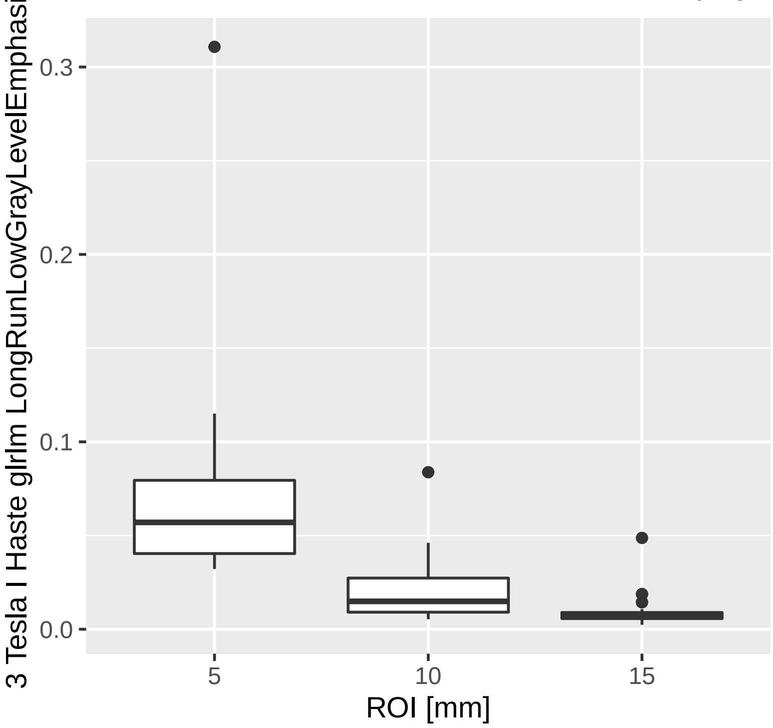
3 Tesla I Haste glrlm LongRunLowGrayLevelEmphasis
0.0
0.1
0.2
0.3
5
10
15
ROI [mm]

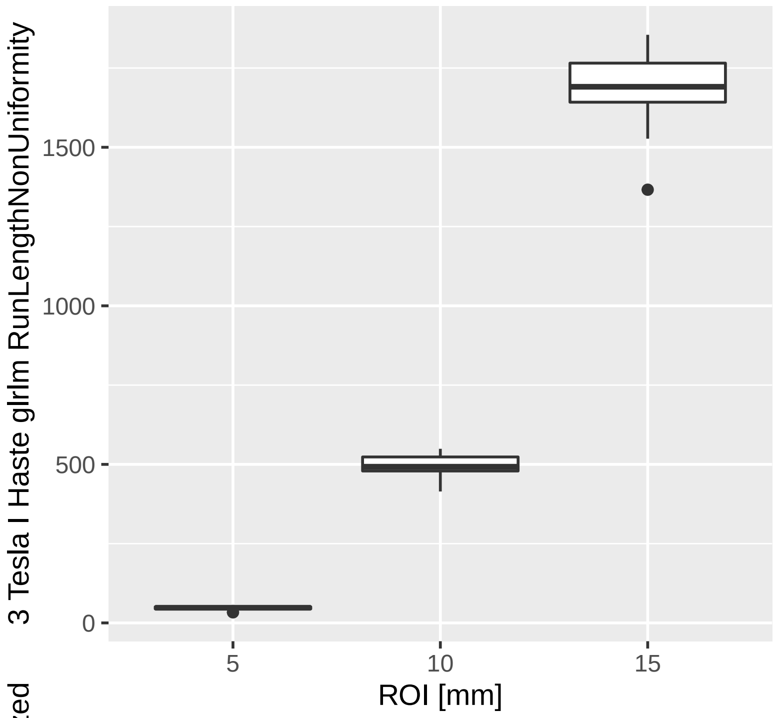
3 Tesla I Haste glrlm RunLengthNonUniformity
0
500
1000
1500
5
10
15
ROI [mm]

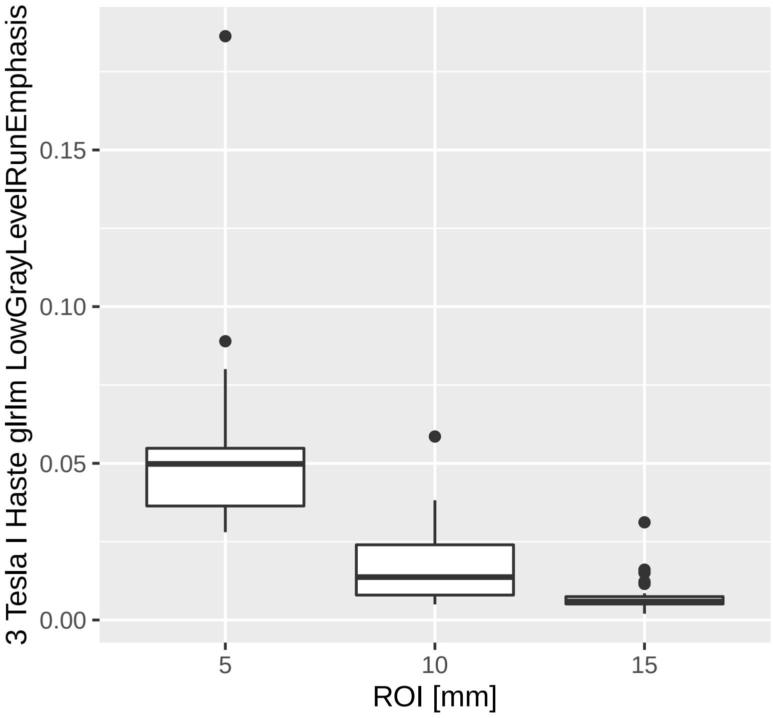
3 Tesla I Haste glrlm LowGrayLevelRunEmphasis
0.00
0.05
0.10
0.15
5
10
15
ROI [mm]

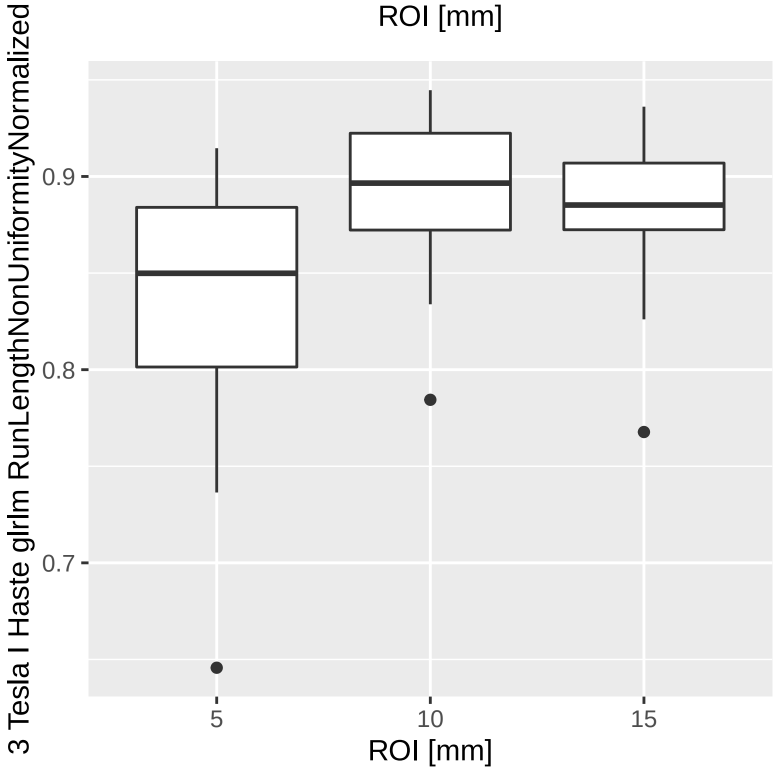
3 Tesla I Haste glrlm RunLengthNonUniformityNormalized
0.7
0.8
0.9
5
10
15
ROI [mm]

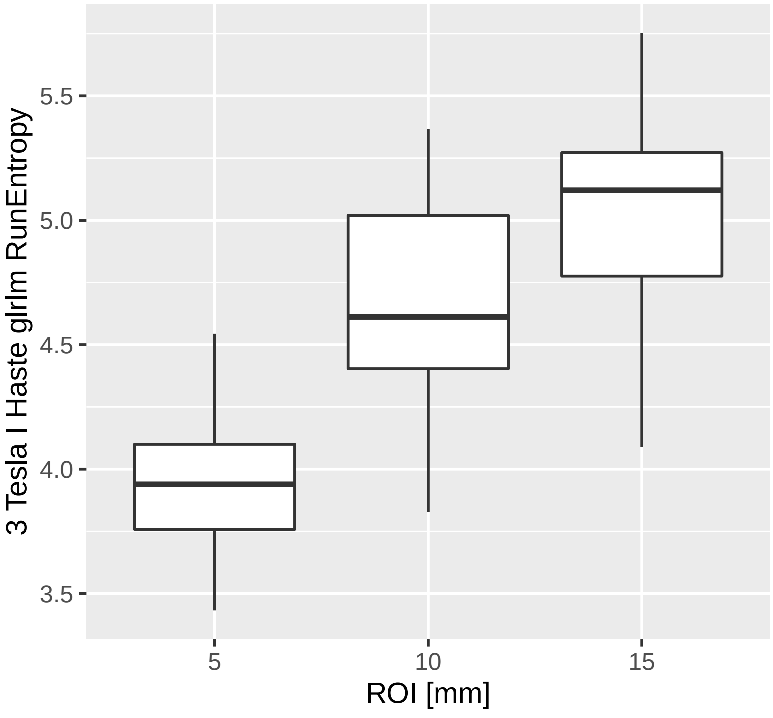
3 Tesla I Haste glrlm RunEntropy
3.5
4.0
4.5
5.0
5.5
5
10
15
ROI [mm]

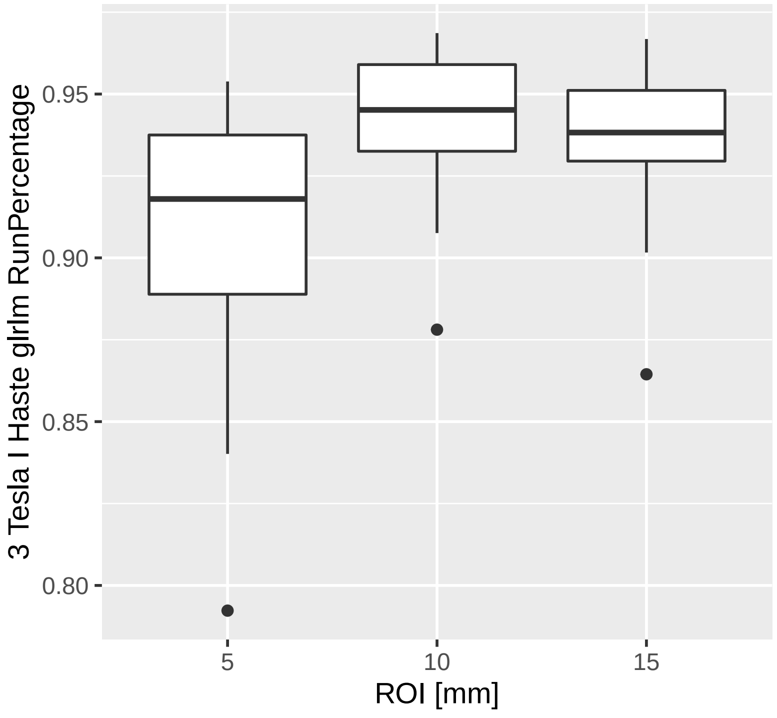
3 Tesla I Haste glrlm RunPercentage
0.80
0.85
0.90
0.95
5
10
15
ROI [mm]

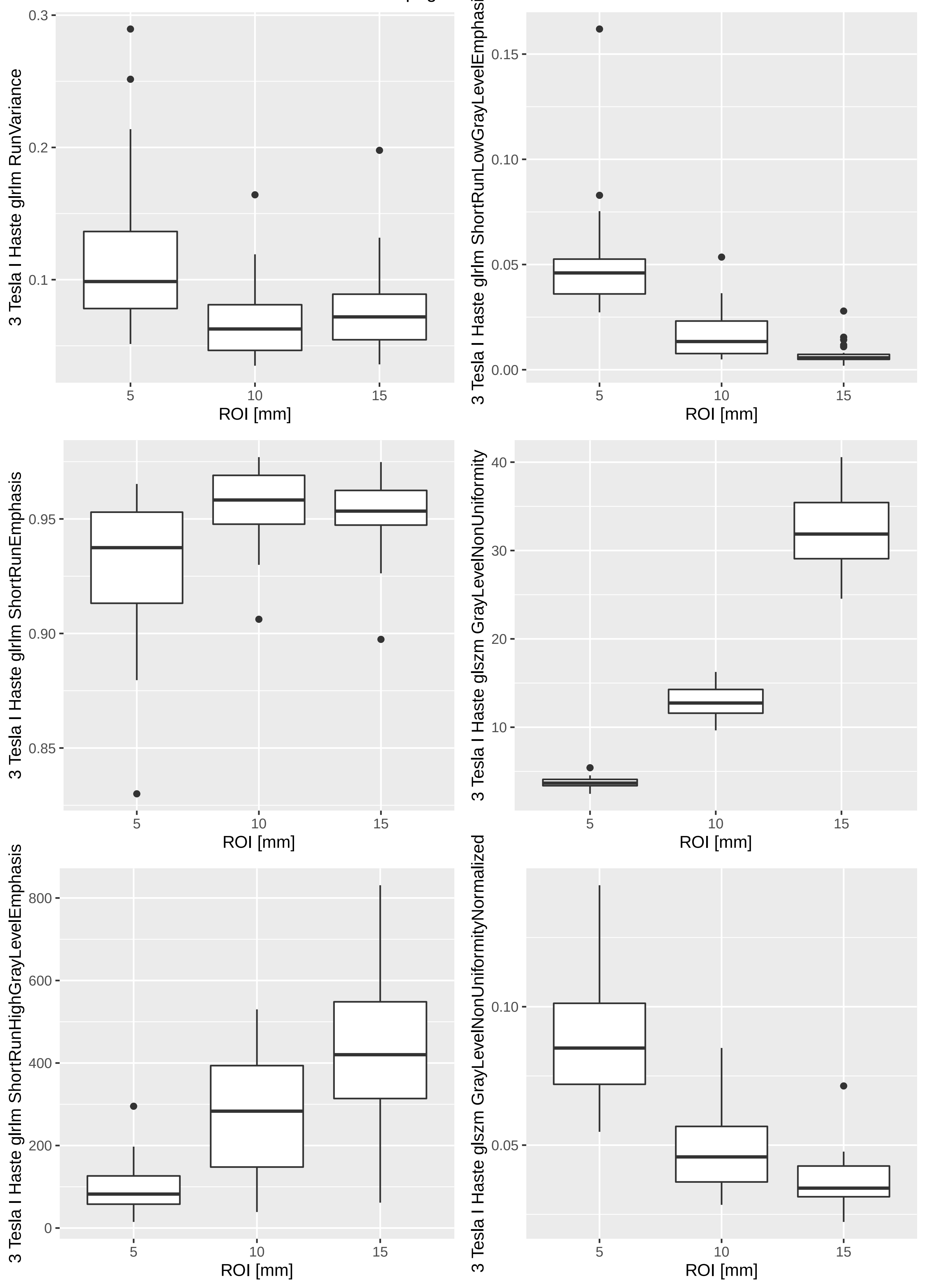

3 Tesla I Haste glrlm RunVariance
0.3
0.2
0.1
5
10
15
ROI [mm]
3 Tesla I Haste glrlm ShortRunLowGrayLevelEmphasis
0.15
0.10
0.05
0.00
5
10
15
ROI [mm]
3 Tesla I Haste glrlm ShortRunEmphasis
0.95
0.90
0.85
5
10
15
ROI [mm]
3 Tesla I Haste glszm GrayLevelNonUniformity
40
30
20
10
5
10
15
ROI [mm]
3 Tesla I Haste glrlm ShortRunHighGrayLevelEmphasis
800
600
400
200
0
5
10
15
ROI [mm]
3 Tesla I Haste glszm GrayLevelNonUniformityNormalized
0.10
0.05
5
10
15
ROI [mm]

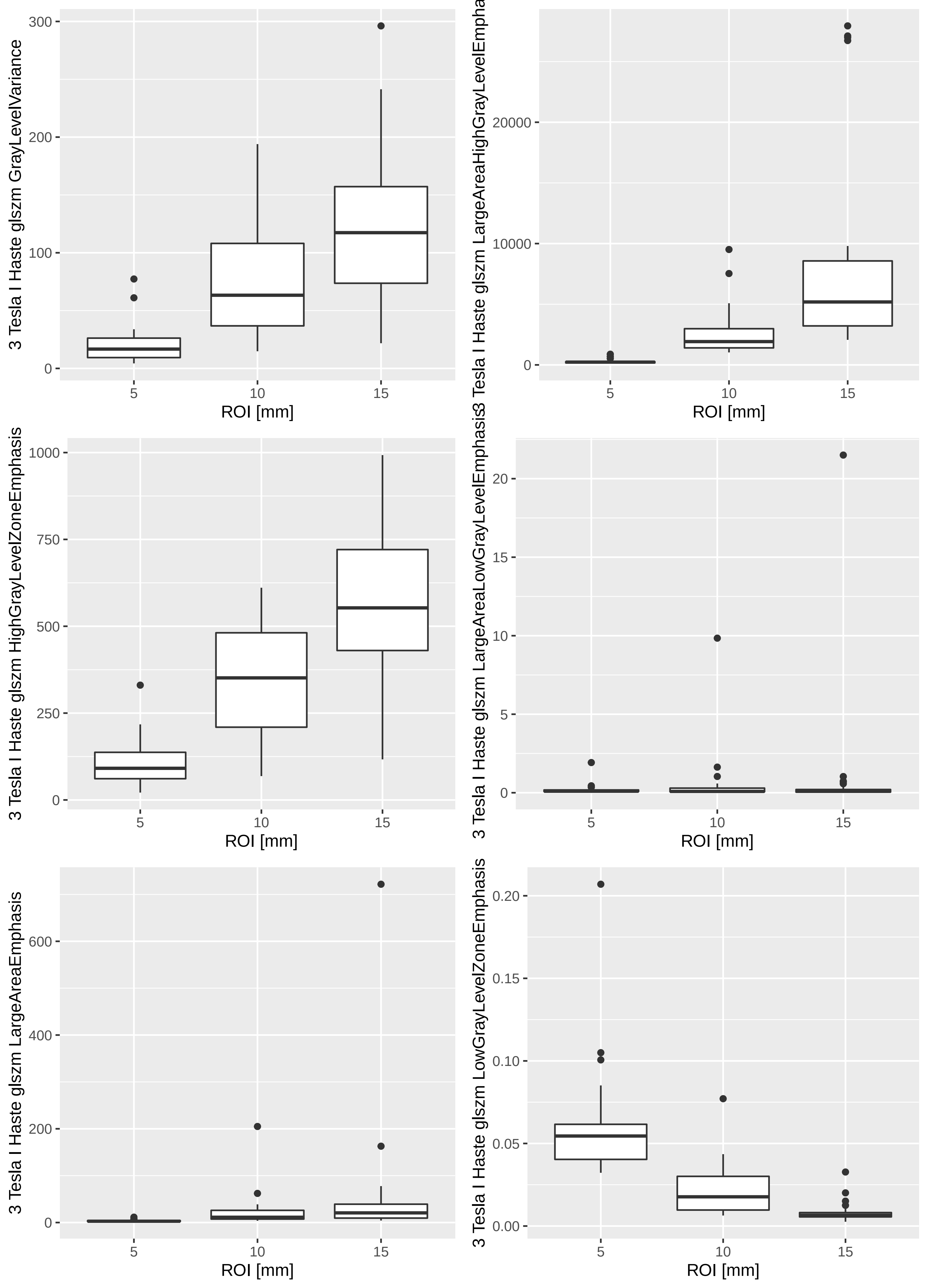

3 Tesla I Haste glszm GrayLevelVariance
0
100
200
300
5
10
15
ROI [mm]
3 Tesla I Haste glszm LargeAreaHighGrayLevelEmphasis
0
10000
20000
5
10
15
ROI [mm]
3 Tesla I Haste glszm HighGrayLevelZoneEmphasis
0
250
500
750
1000
5
10
15
ROI [mm]
3 Tesla I Haste glszm LargeAreaLowGrayLevelEmphasis
0
5
10
15
20
5
10
15
ROI [mm]
3 Tesla I Haste glszm LargeAreaEmphasis
0
200
400
600
5
10
15
ROI [mm]
3 Tesla I Haste glszm LowGrayLevelZoneEmphasis
0.00
0.05
0.10
0.15
0.20
5
10
15
ROI [mm]

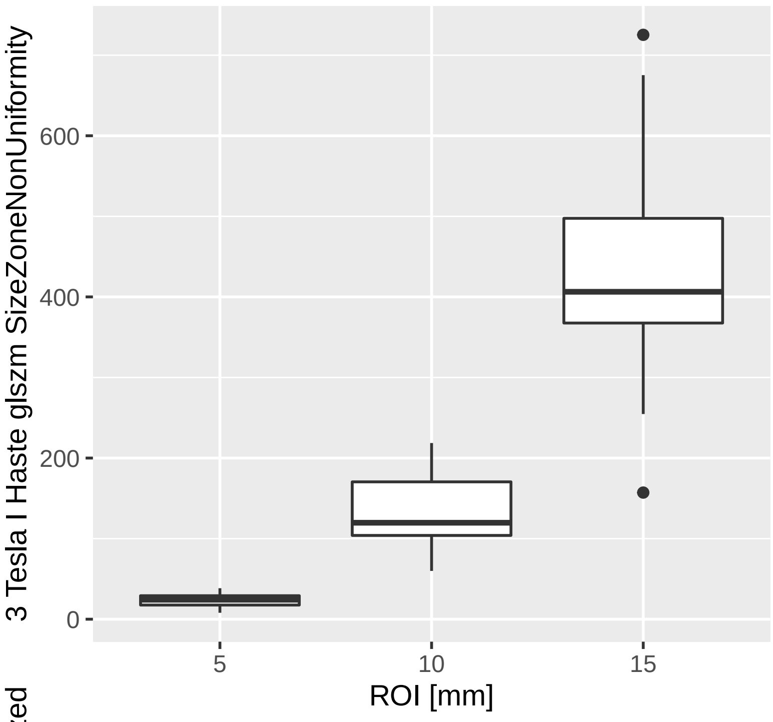
3 Tesla I Haste glszm SizeZoneNonUniformity
0
200
400
600
5
10
15
ROI [mm]

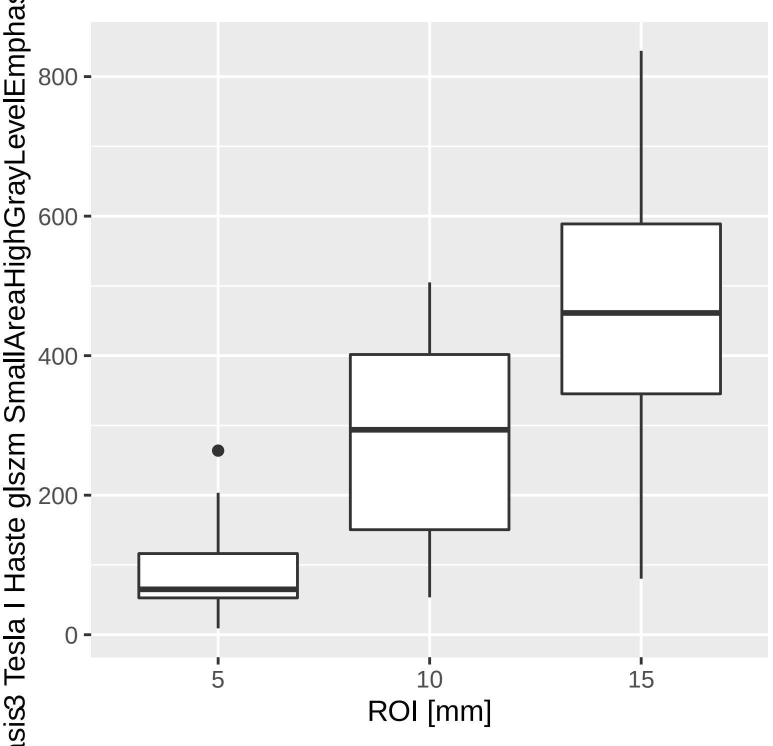
3 Tesla I Haste glszm SmallAreaHighGrayLevelEmphasis
0
200
400
600
800
5
10
15
ROI [mm]

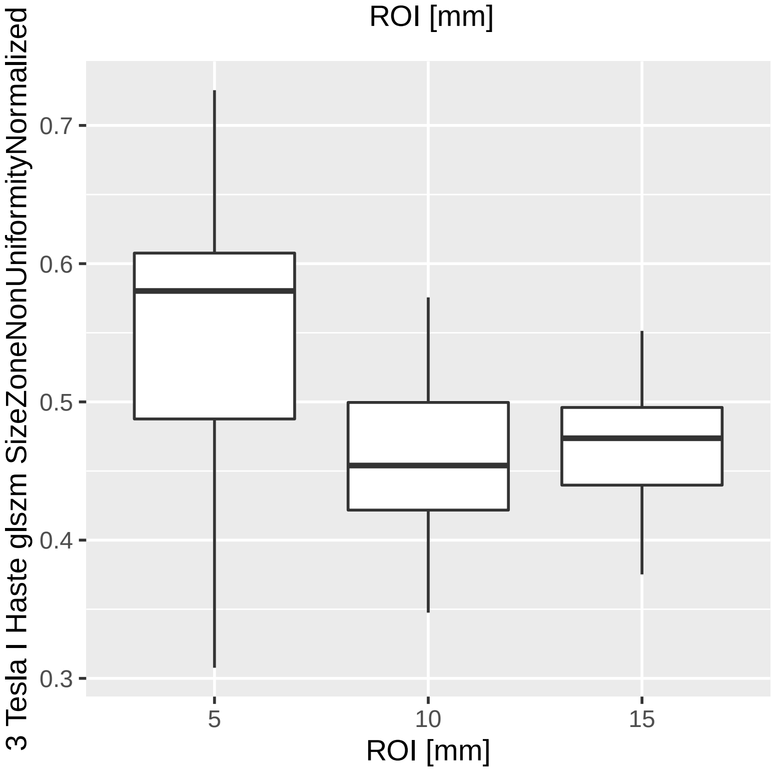
3 Tesla I Haste glszm SizeZoneNonUniformityNormalized
0.3
0.4
0.5
0.6
0.7
5
10
15
ROI [mm]

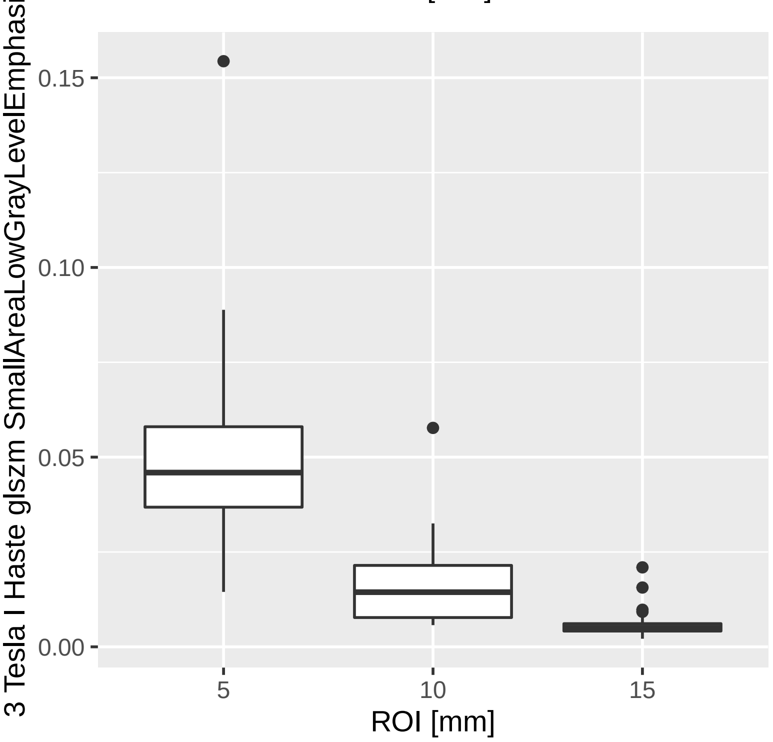
3 Tesla I Haste glszm SmallAreaLowGrayLevelEmphasis
0.00
0.05
0.10
0.15
5
10
15
ROI [mm]

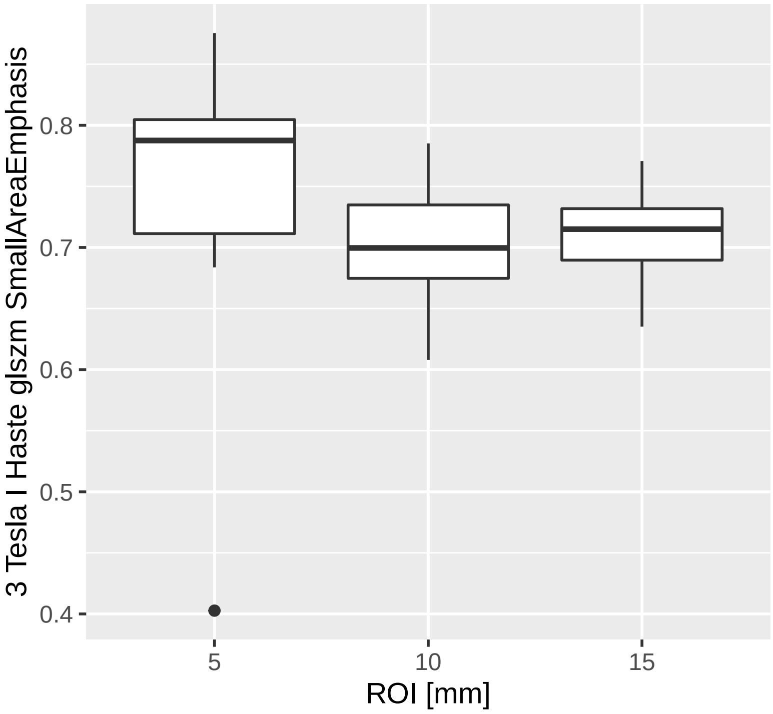
3 Tesla I Haste glszm SmallAreaEmphasis
0.4
0.5
0.6
0.7
0.8
5
10
15
ROI [mm]

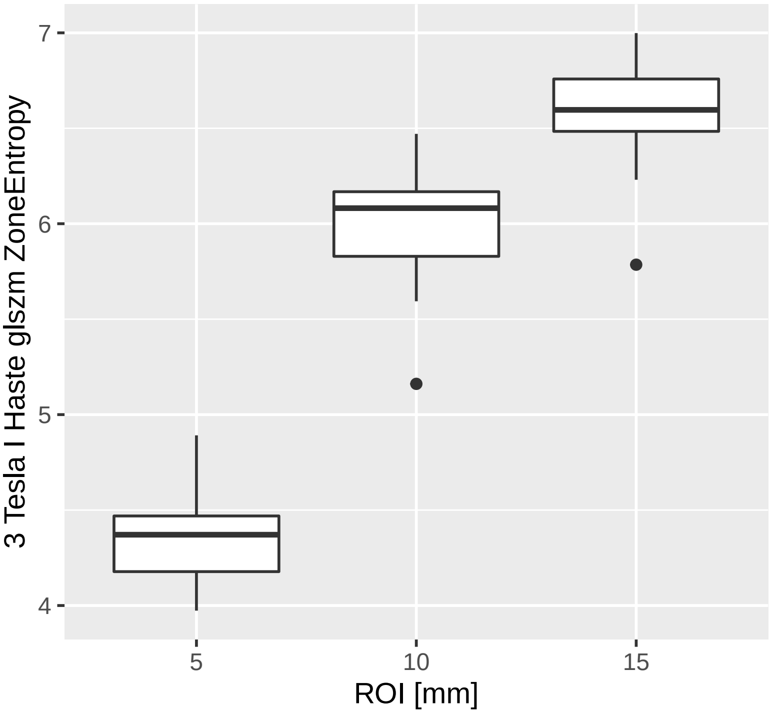
3 Tesla I Haste glszm ZoneEntropy
4
5
6
7
5
10
15
ROI [mm]

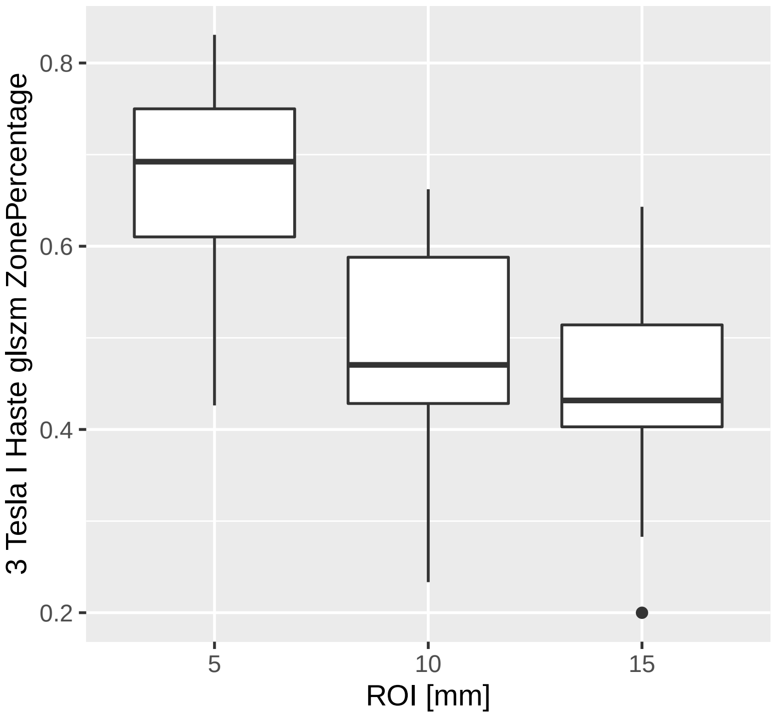
3 Tesla I Haste glszm ZonePercentage
0.8
0.6
0.4
0.2
5
10
15
ROI [mm]

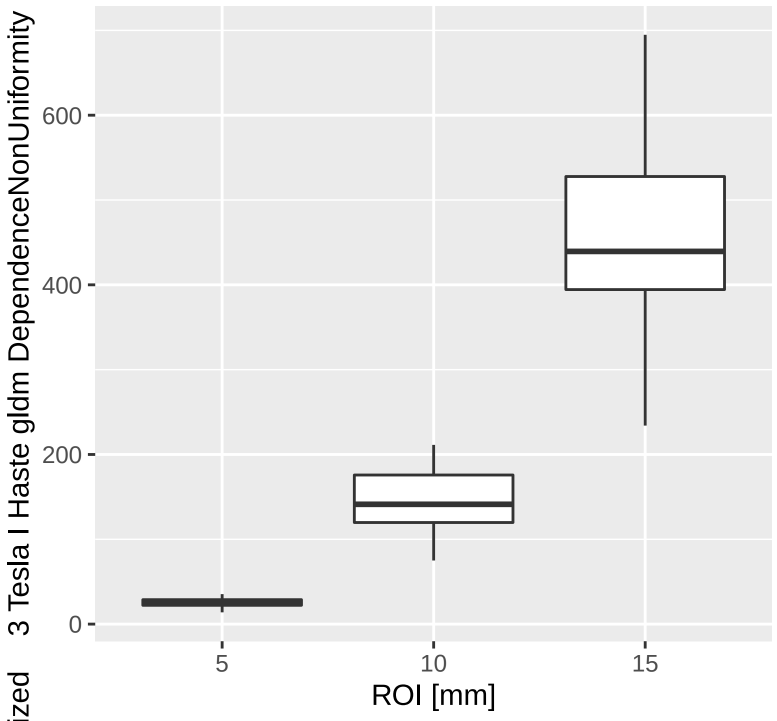
3 Tesla I Haste gldm DependenceNonUniformity
600
400
200
0
5
10
15
ROI [mm]

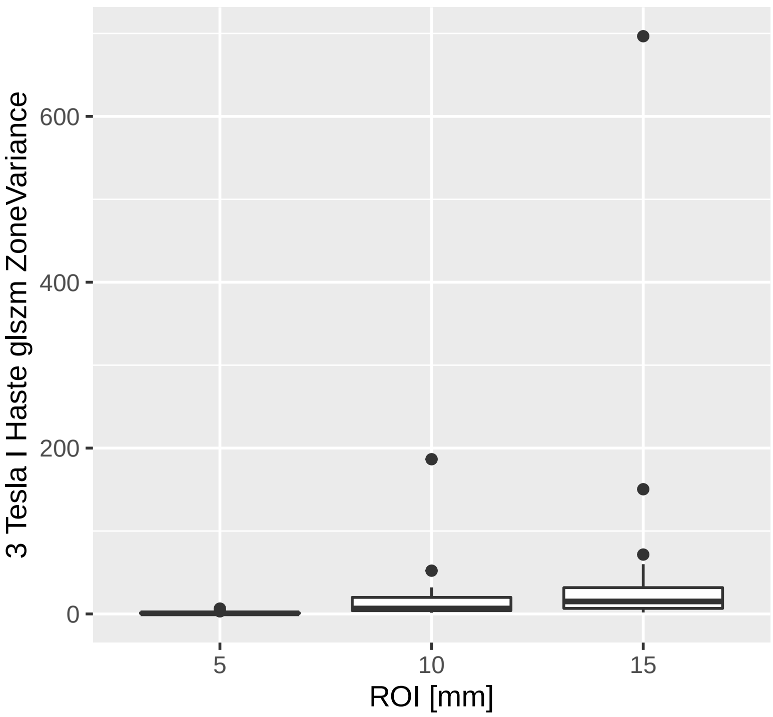
3 Tesla I Haste glszm ZoneVariance
600
400
200
0
5
10
15
ROI [mm]

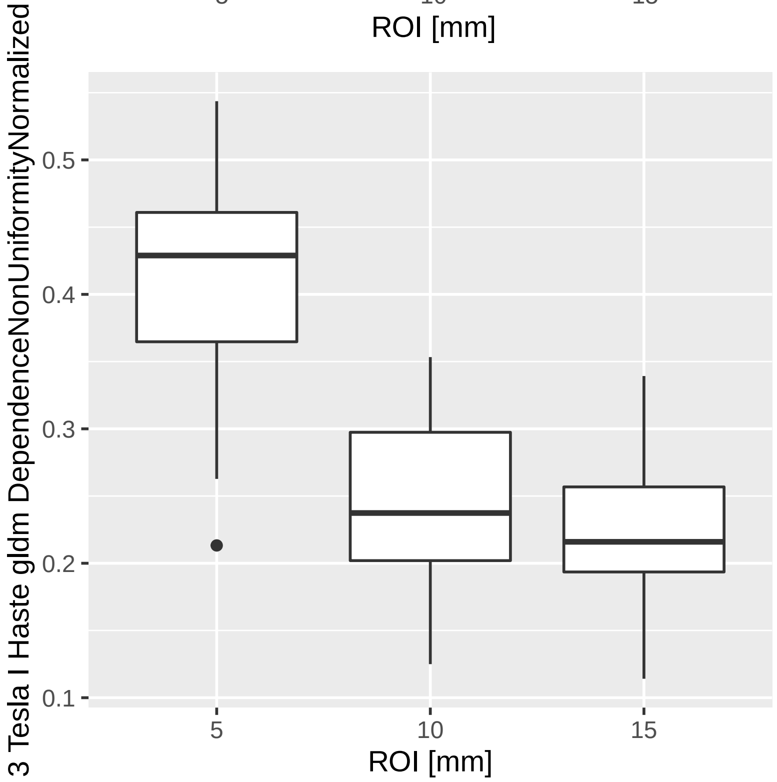
3 Tesla I Haste gldm DependenceNonUniformityNormalized
0.5
0.4
0.3
0.2
0.1
5
10
15
ROI [mm]

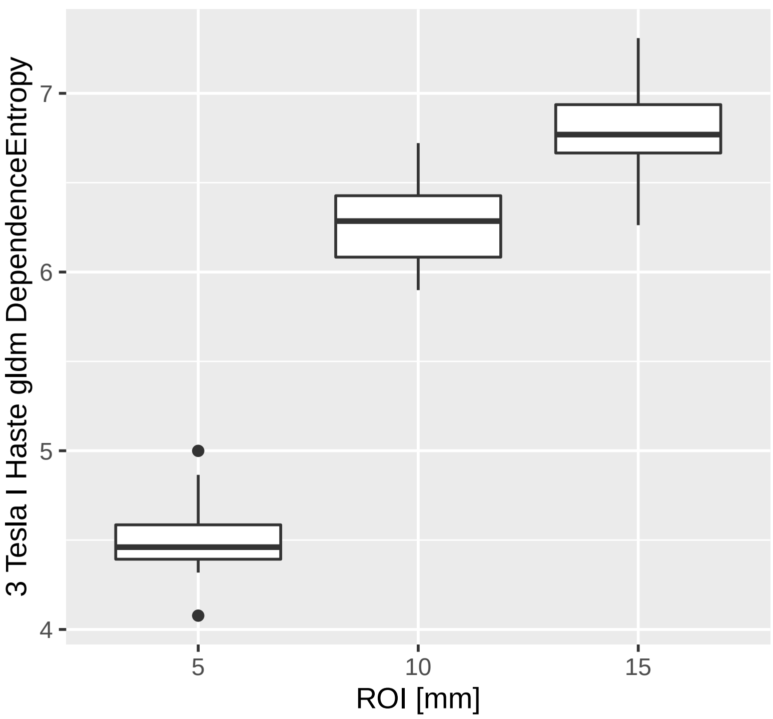
3 Tesla I Haste gldm DependenceEntropy
7
6
5
4
5
10
15
ROI [mm]

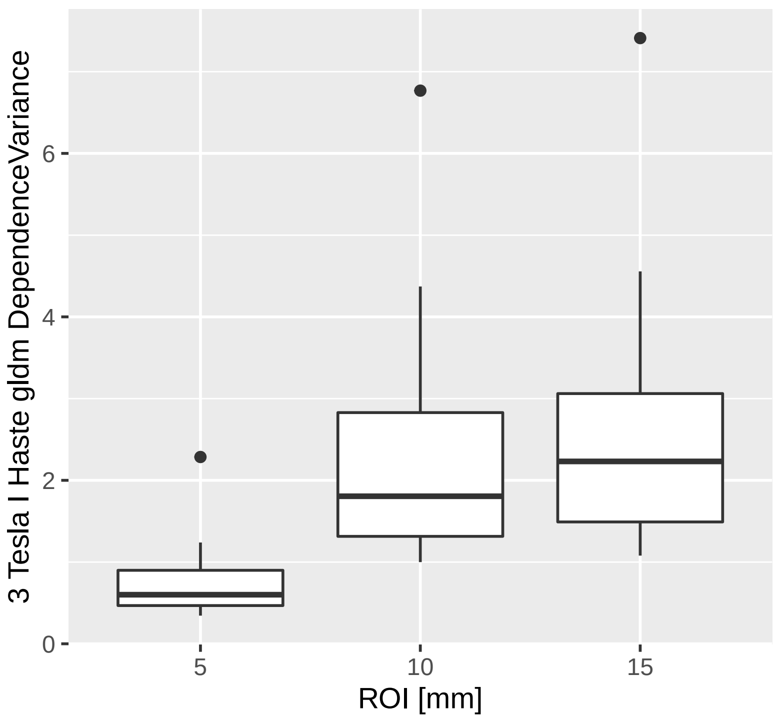
3 Tesla I Haste gldm DependenceVariance
6
4
2
0
5
10
15
ROI [mm]

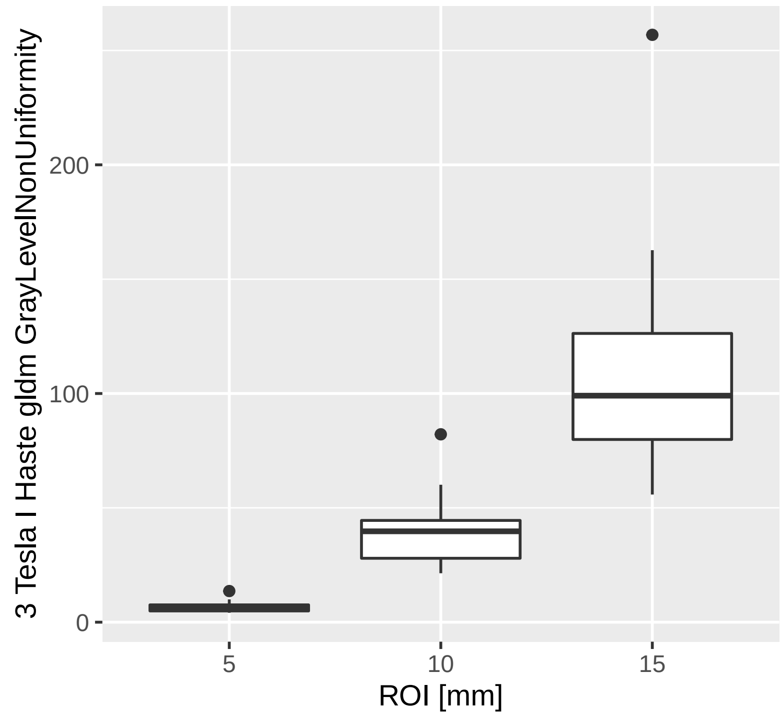
3 Tesla I Haste gldm GrayLevelNonUniformity
0
100
200
5
10
15
ROI [mm]

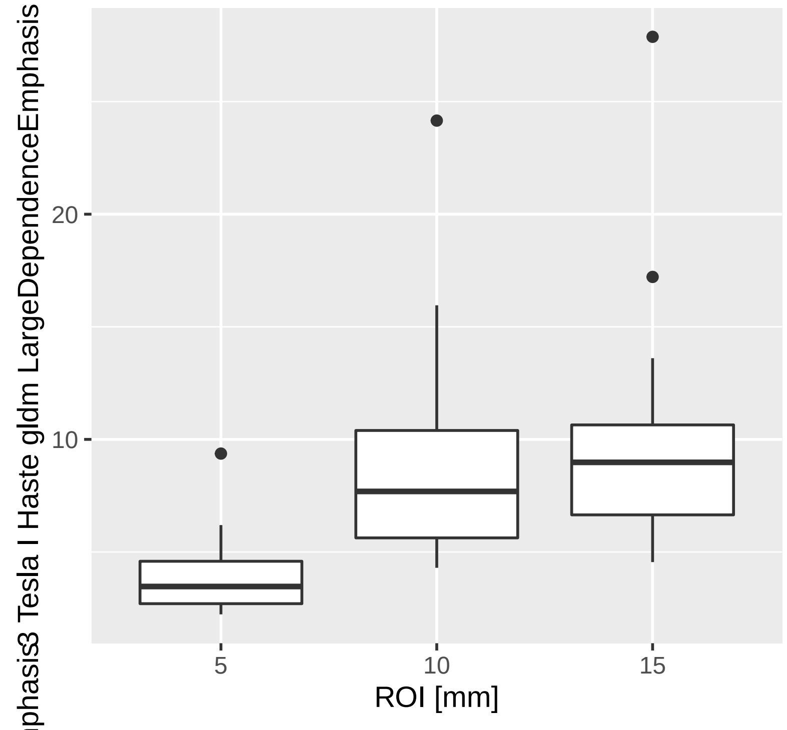
3 Tesla I Haste gldm LargeDependenceEmphasis
10
20
5
10
15
ROI [mm]

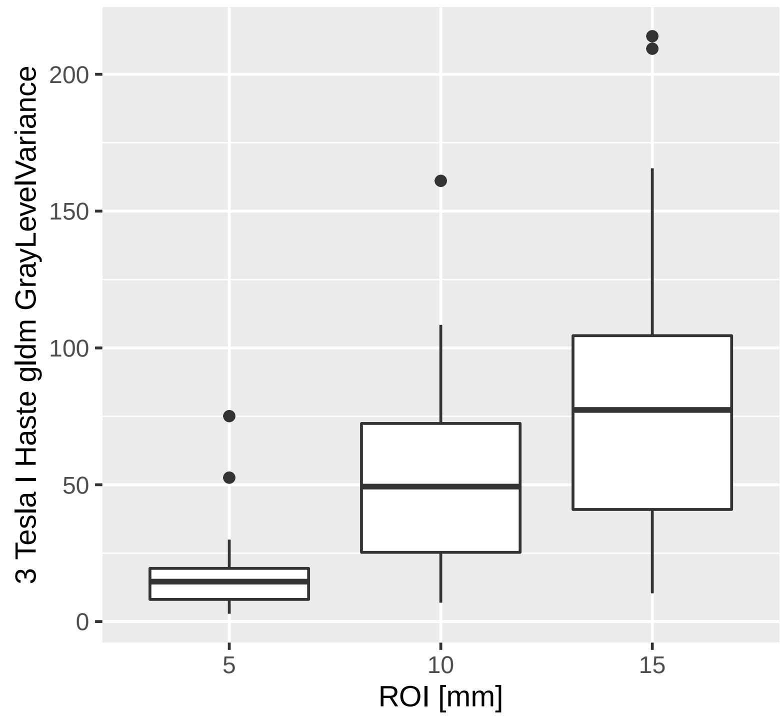
3 Tesla I Haste gldm GrayLevelVariance
0
50
100
150
200
5
10
15
ROI [mm]

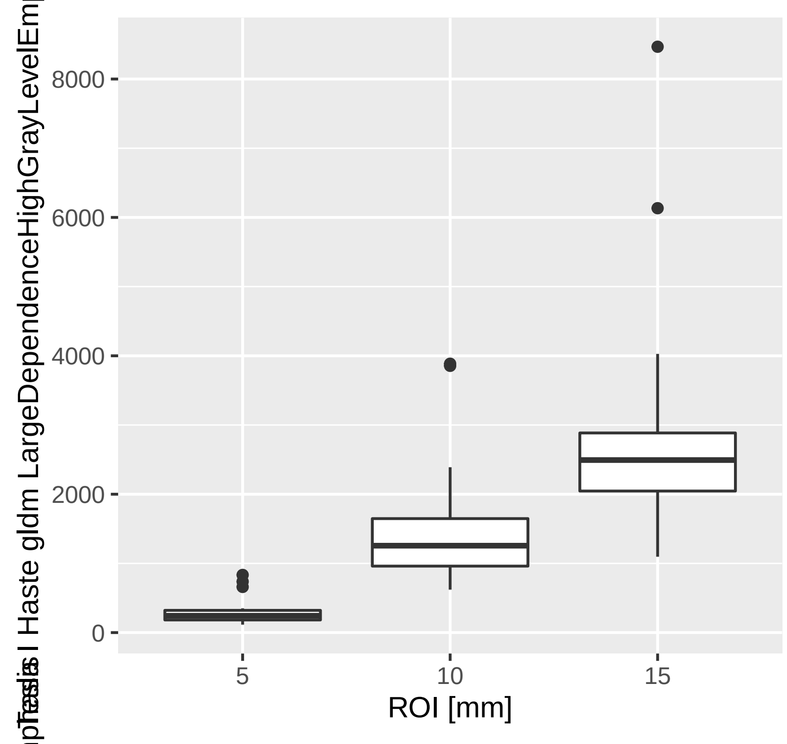
3 Tesla I Haste gldm LargeDependenceHighGrayLevelEmphasis
0
2000
4000
6000
8000
5
10
15
ROI [mm]

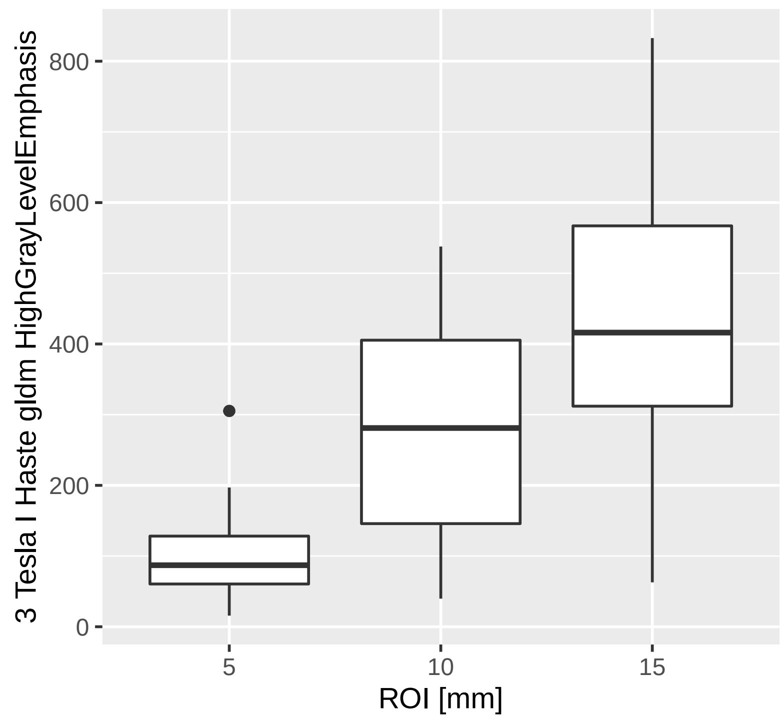
3 Tesla I Haste gldm HighGrayLevelEmphasis
0
200
400
600
800
5
10
15
ROI [mm]

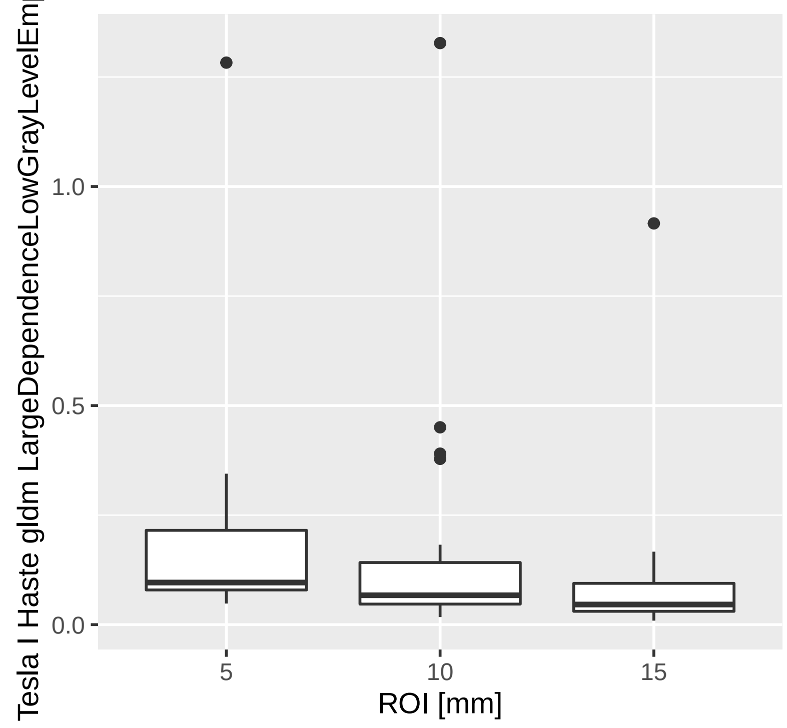
3 Tesla I Haste gldm LargeDependenceLowGrayLevelEmphasis
0.0
0.5
1.0
5
10
15
ROI [mm]

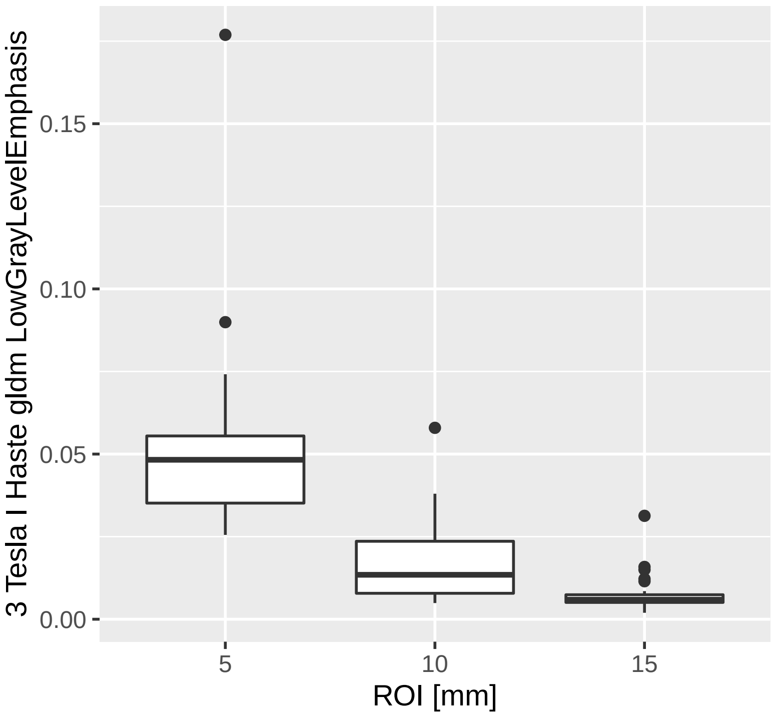
3 Tesla I Haste gldm LowGrayLevelEmphasis
0.00
0.05
0.10
0.15
5
10
15
ROI [mm]

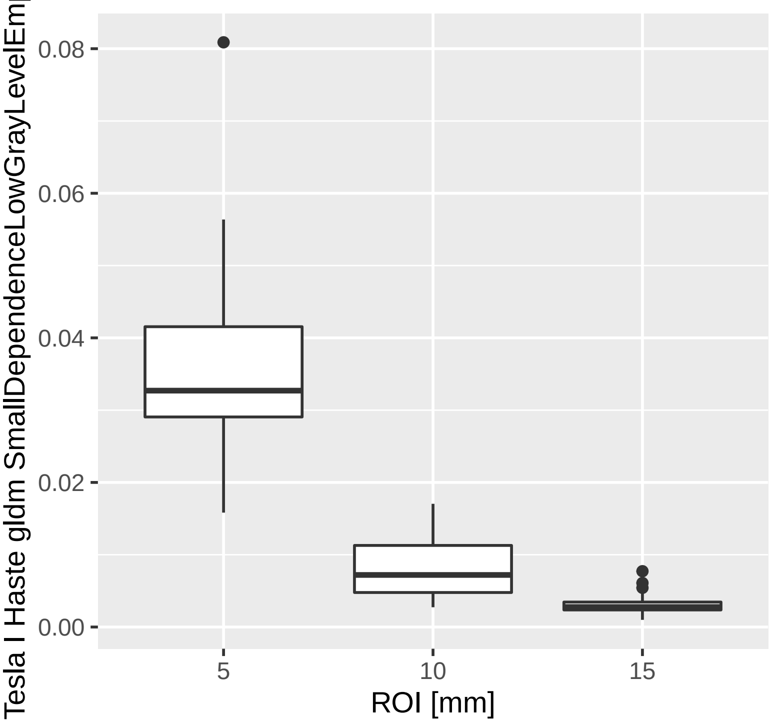
Tesla I Haste gldm SmallDependenceLowGrayLevelEmpha
0.00
0.02
0.04
0.06
0.08
5
10
15
ROI [mm]

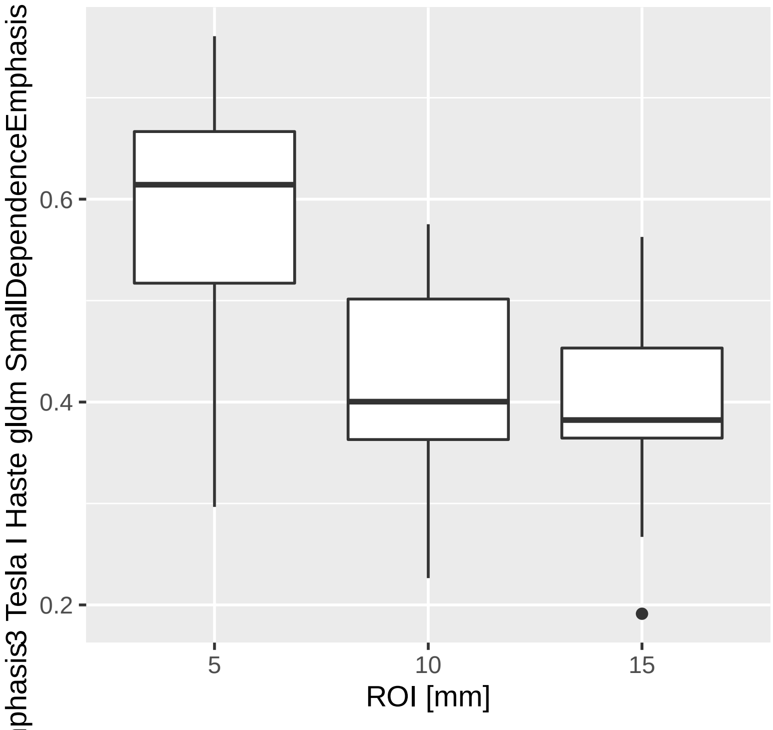
3 Tesla I Haste gldm SmallDependenceEmphasis
0.2
0.4
0.6
5
10
15
ROI [mm]

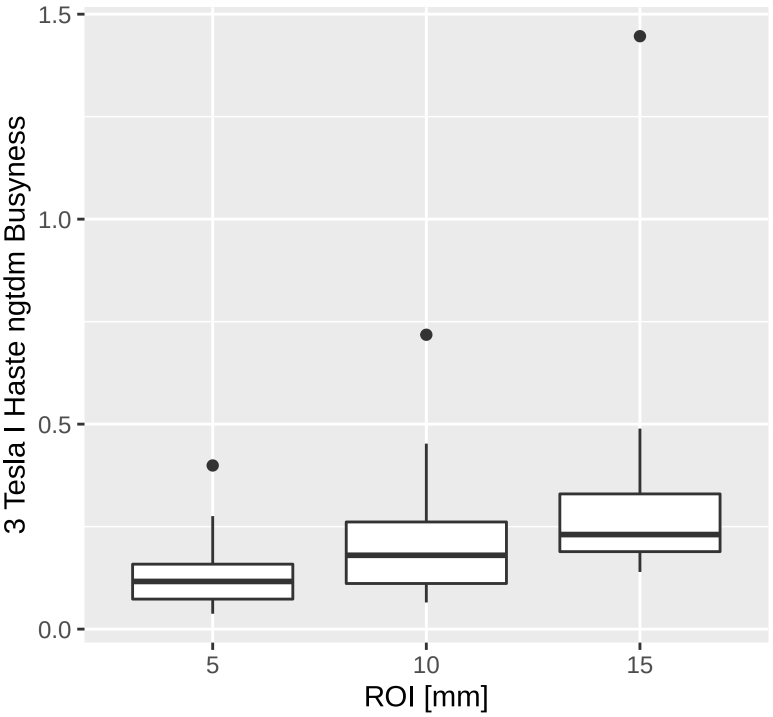
3 Tesla I Haste ngtdm Busyness
0.0
0.5
1.0
1.5
5
10
15
ROI [mm]

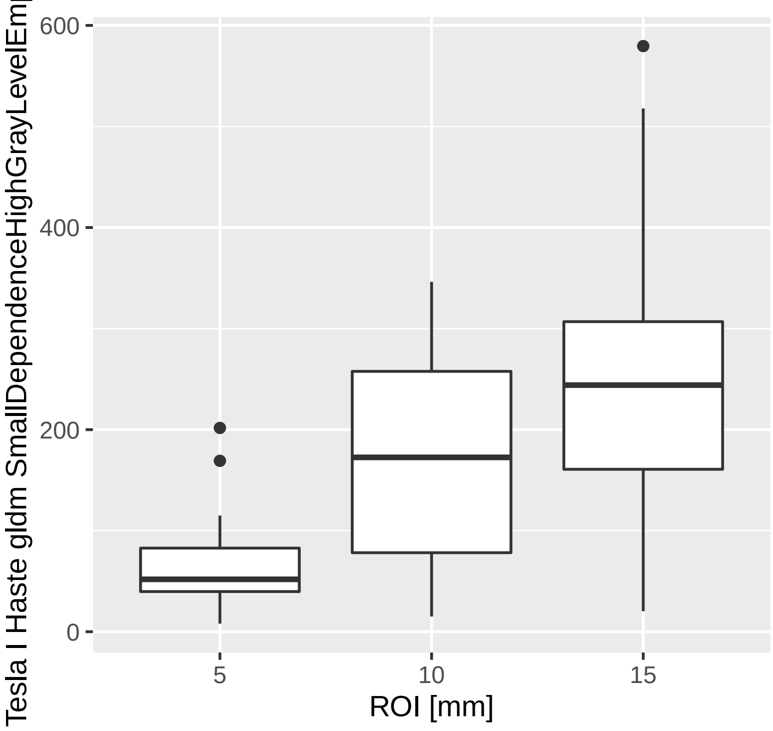
Tesla I Haste gldm SmallDependenceHighGrayLevelEmphasis
0
200
400
600
5
10
15
ROI [mm]

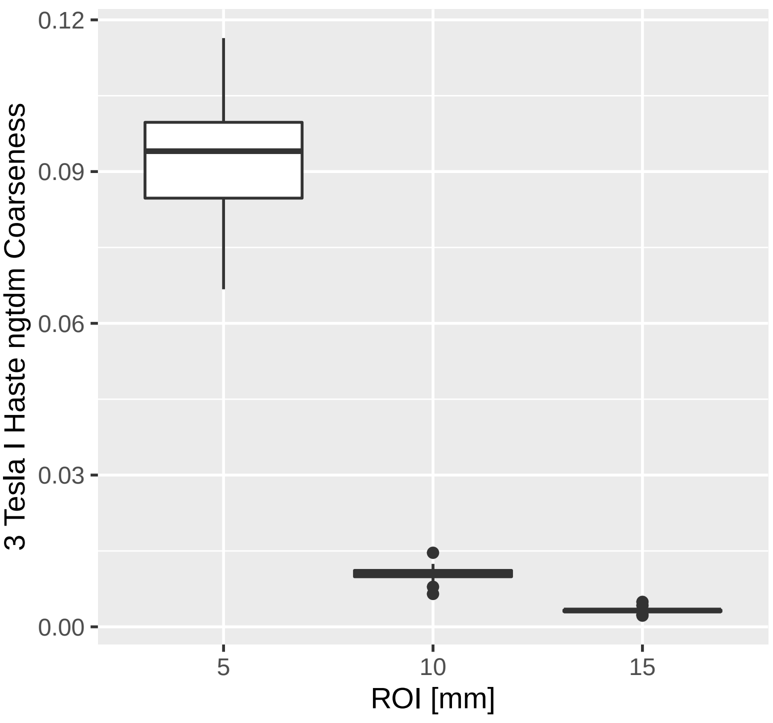
3 Tesla I Haste ngtdm Coarseness
0.00
0.03
0.06
0.09
0.12
5
10
15
ROI [mm]

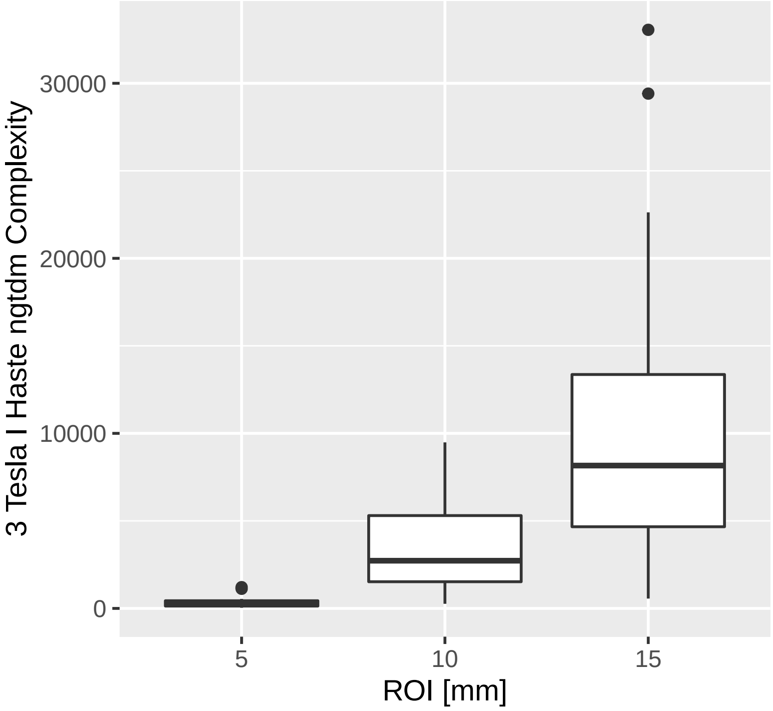
3 Tesla I Haste ngtdm Complexity
30000
20000
10000
0
5
10
15
ROI [mm]

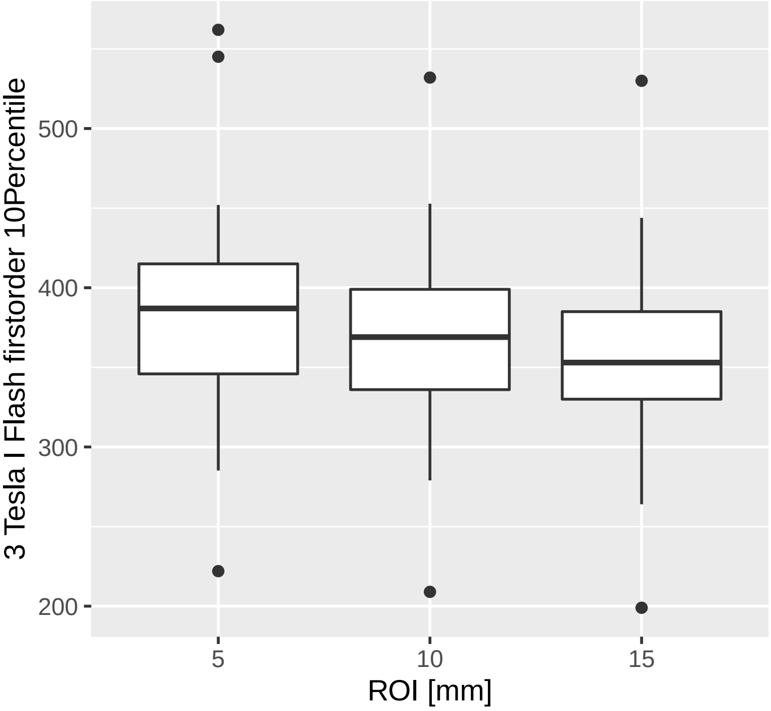
3 Tesla I Flash firstorder 10Percentile
500
400
300
200
5
10
15
ROI [mm]

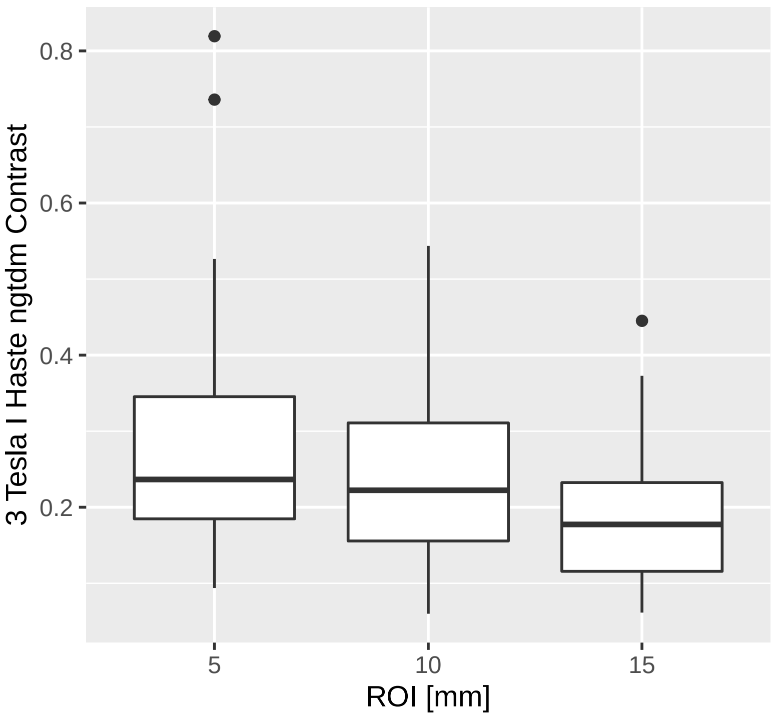
3 Tesla I Haste ngtdm Contrast
0.8
0.6
0.4
0.2
5
10
15
ROI [mm]

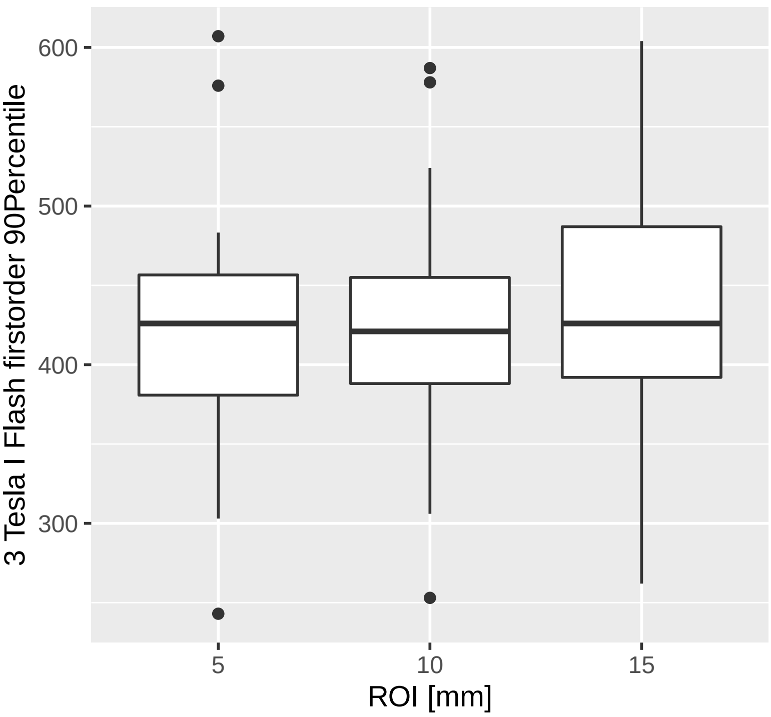
3 Tesla I Flash firstorder 90Percentile
600
500
400
300
5
10
15
ROI [mm]

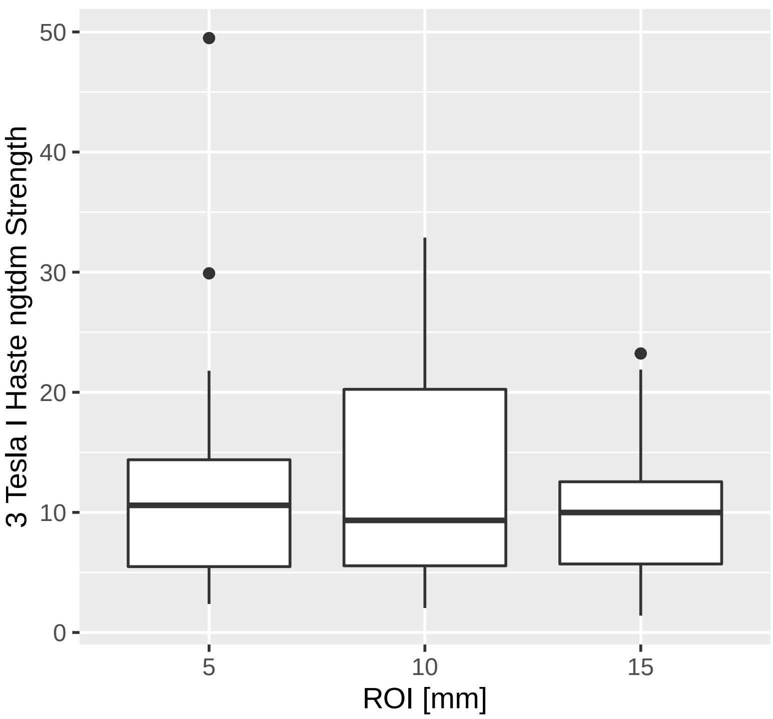
3 Tesla I Haste ngtdm Strength
50
40
30
20
10
0
5
10
15
ROI [mm]

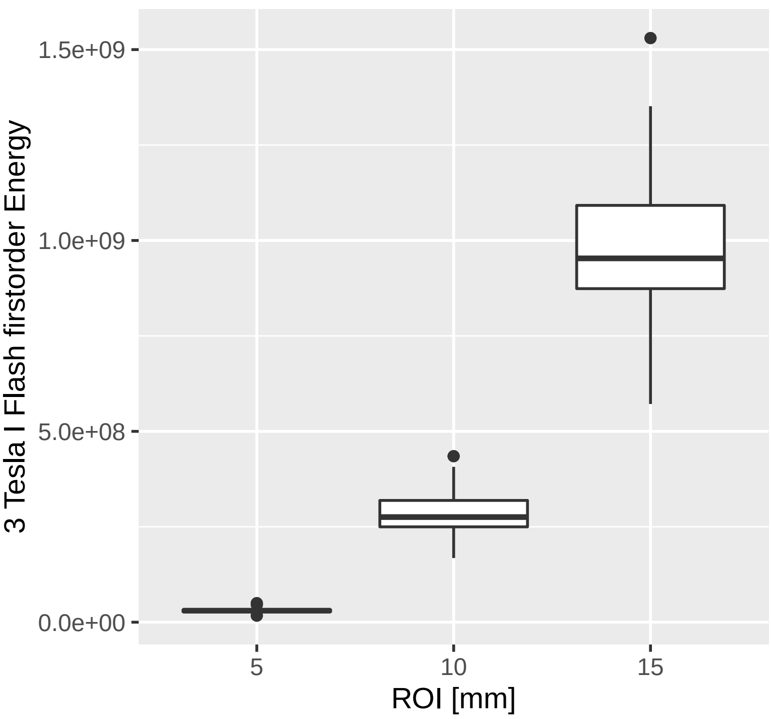
3 Tesla I Flash firstorder Energy
1.5e+09
1.0e+09
5.0e+08
0.0e+00
5
10
15
ROI [mm]

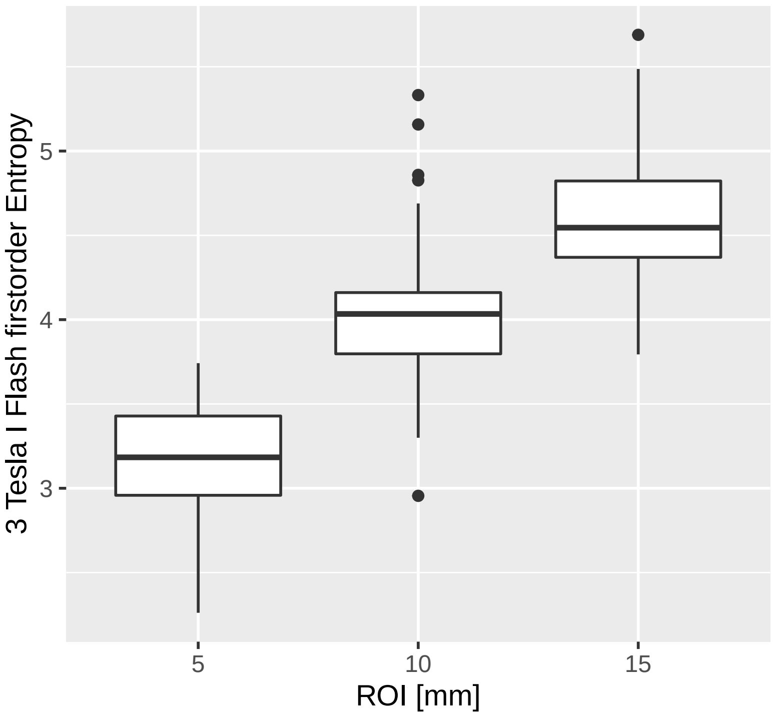
3 Tesla I Flash firstorder Entropy
3
4
5
5
10
15
ROI [mm]

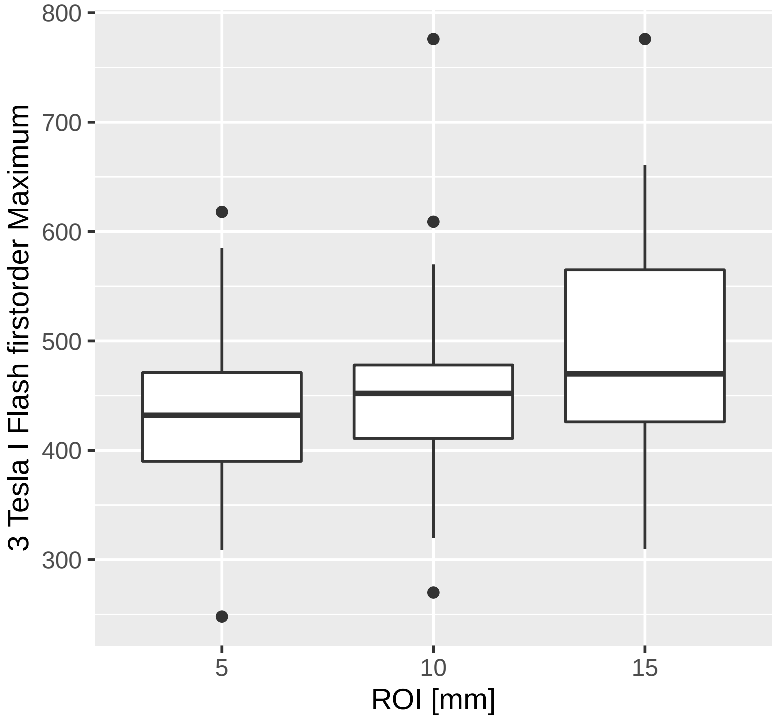
3 Tesla I Flash firstorder Maximum
300
400
500
600
700
800
5
10
15
ROI [mm]

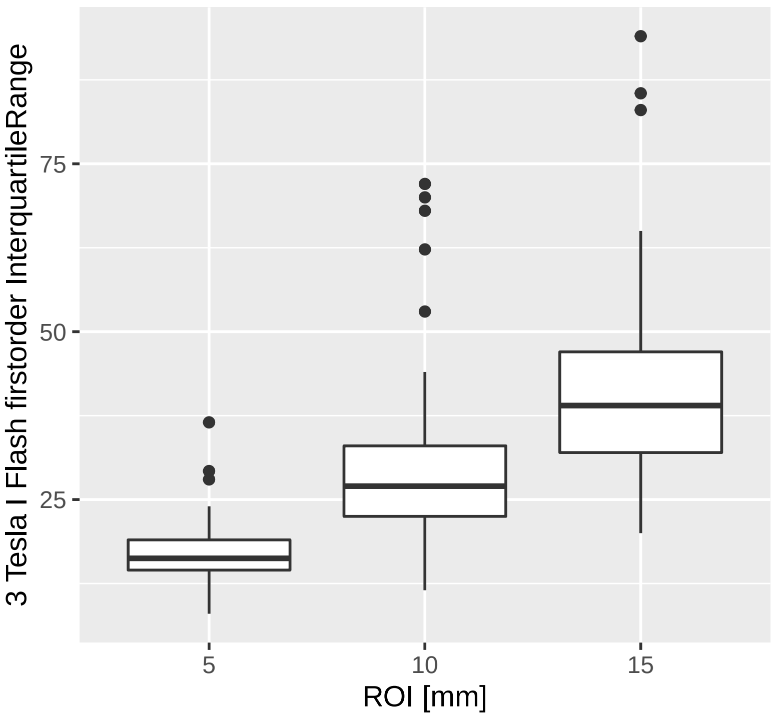
3 Tesla I Flash firstorder InterquartileRange
25
50
75
5
10
15
ROI [mm]

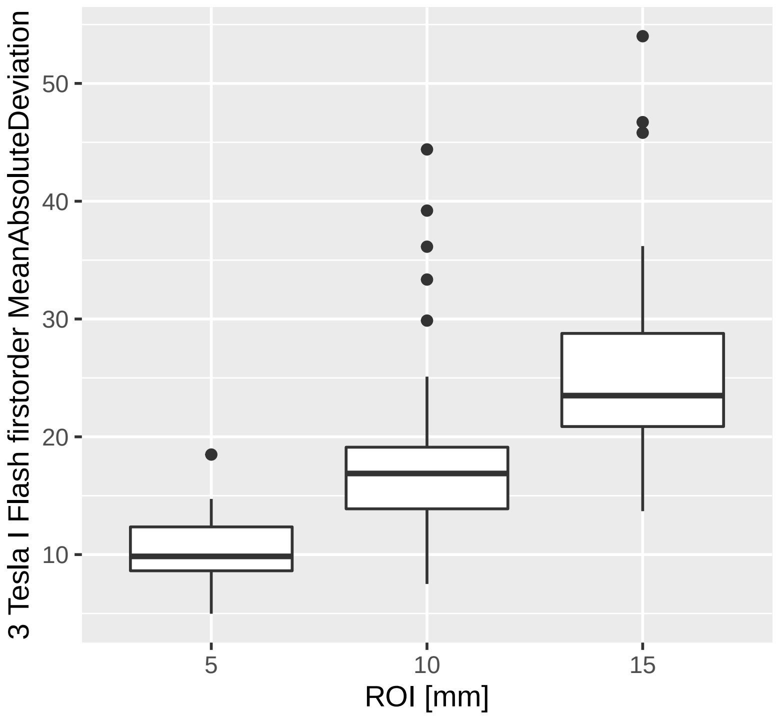
3 Tesla I Flash firstorder MeanAbsoluteDeviation
10
20
30
40
50
5
10
15
ROI [mm]

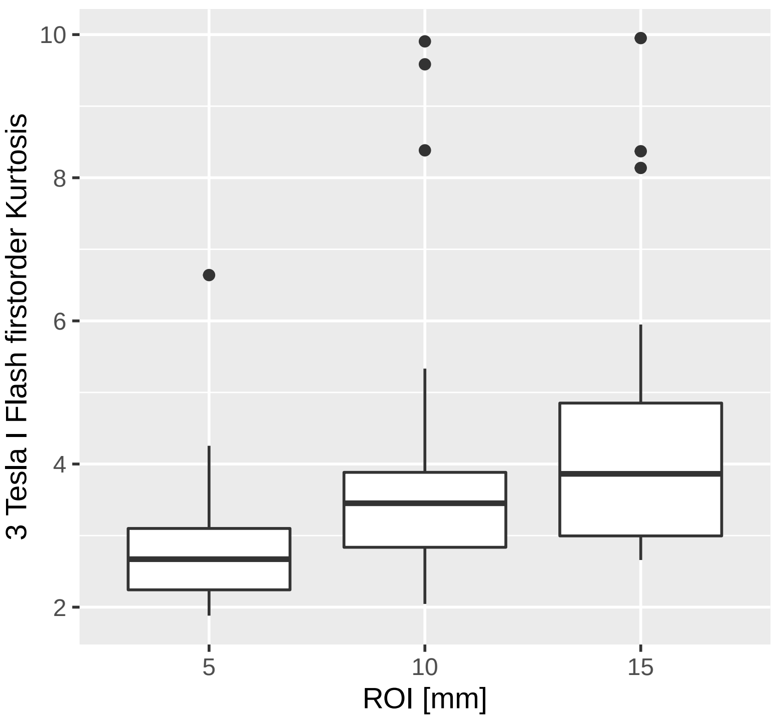
3 Tesla I Flash firstorder Kurtosis
2
4
6
8
10
5
10
15
ROI [mm]

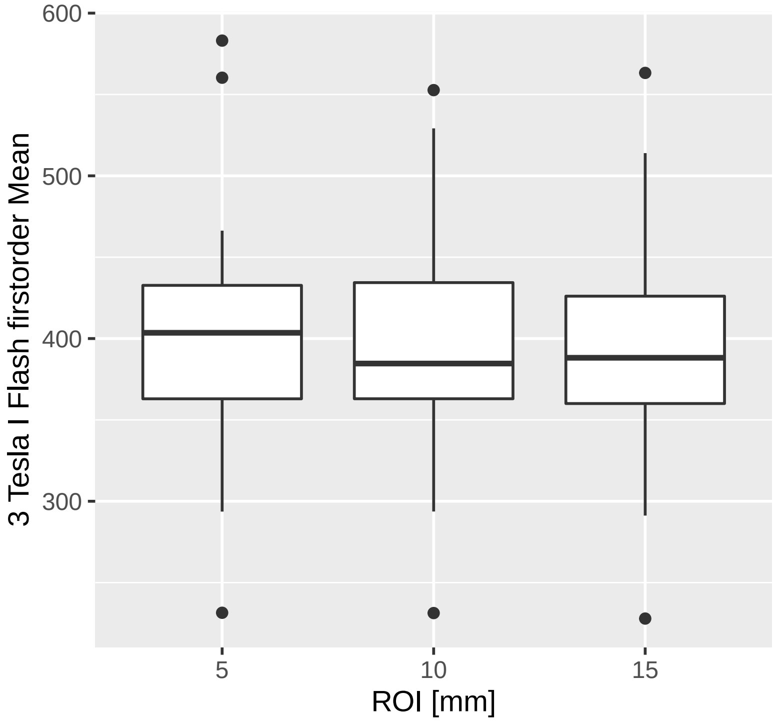
3 Tesla I Flash firstorder Mean
300
400
500
600
5
10
15
ROI [mm]

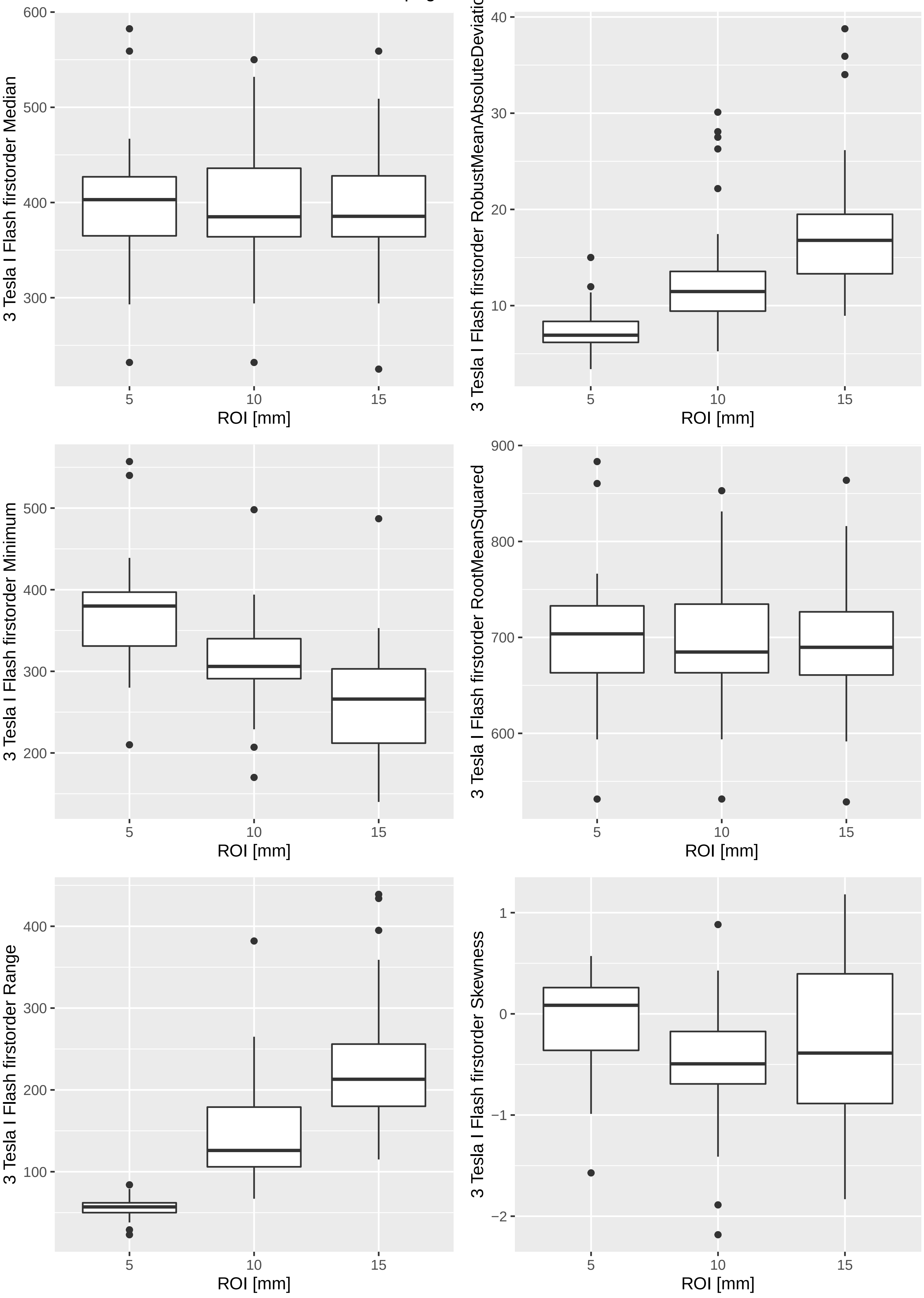
3 Tesla I Flash firstorder Median
3 Tesla I Flash firstorder RobustMeanAbsoluteDeviation
3 Tesla I Flash firstorder Minimum
3 Tesla I Flash firstorder RootMeanSquared
3 Tesla I Flash firstorder Range
3 Tesla I Flash firstorder Skewness
ROI [mm]
5
10
15

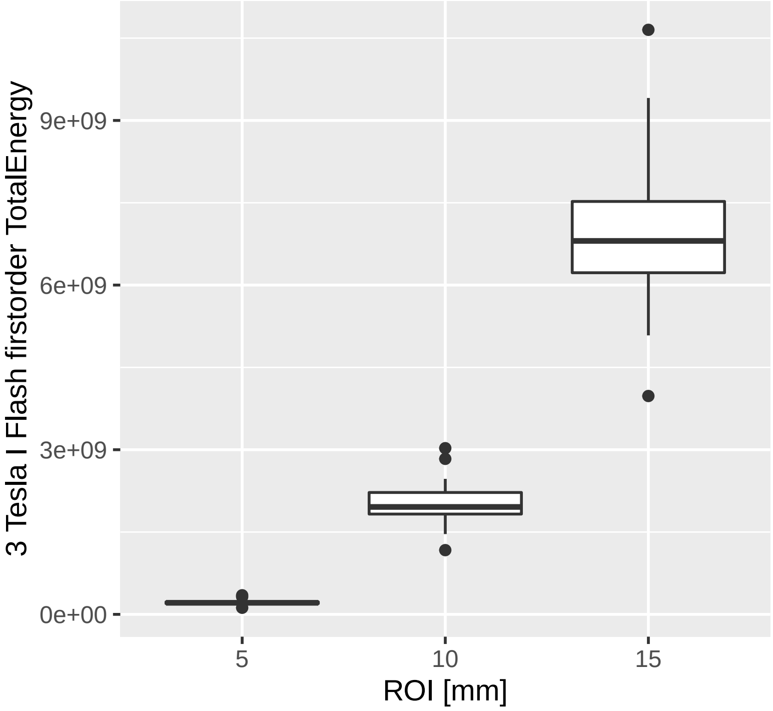
3 Tesla I Flash firstorder TotalEnergy
9e+09
6e+09
3e+09
0e+00
5
10
15
ROI [mm]

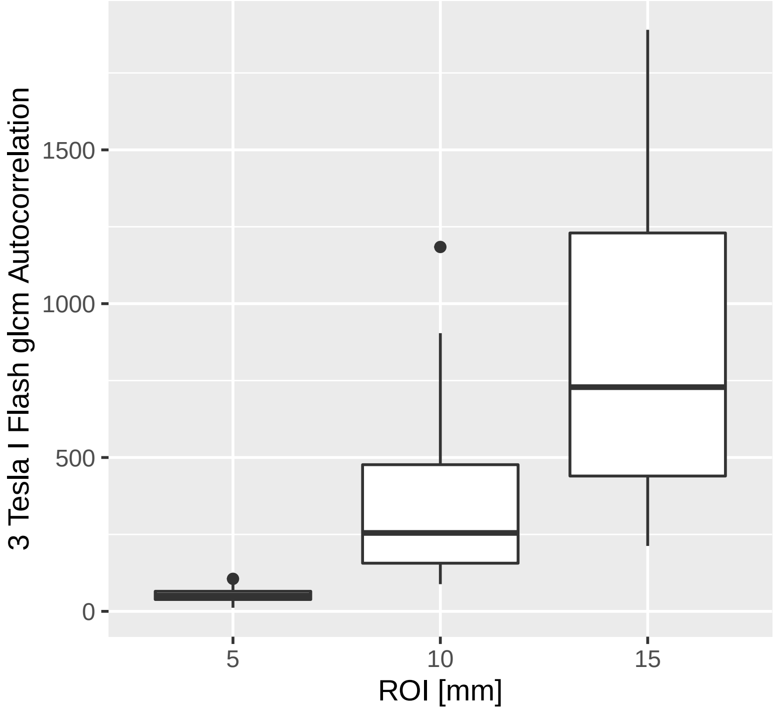
3 Tesla I Flash glcm Autocorrelation
1500
1000
500
0
5
10
15
ROI [mm]

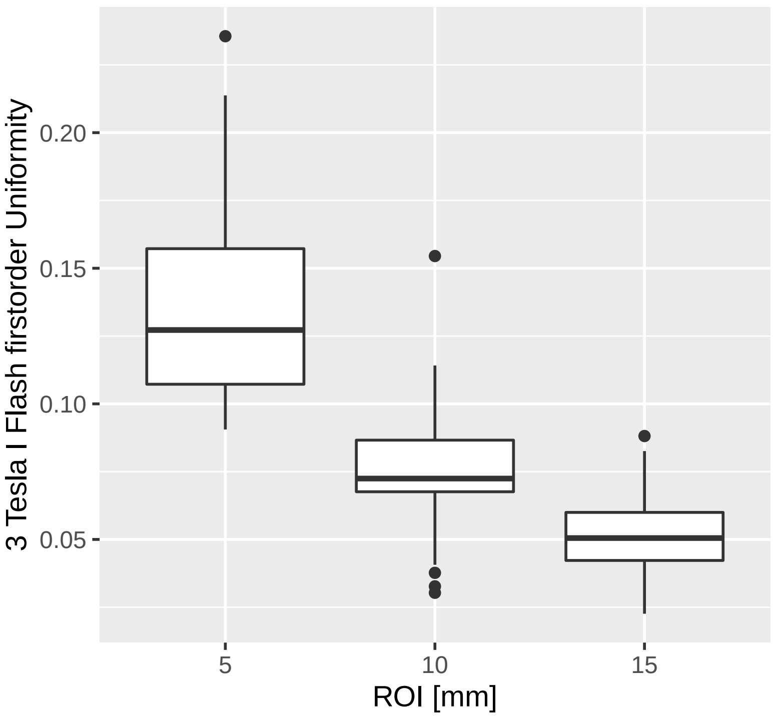
3 Tesla I Flash firstorder Uniformity
0.20
0.15
0.10
0.05
5
10
15
ROI [mm]

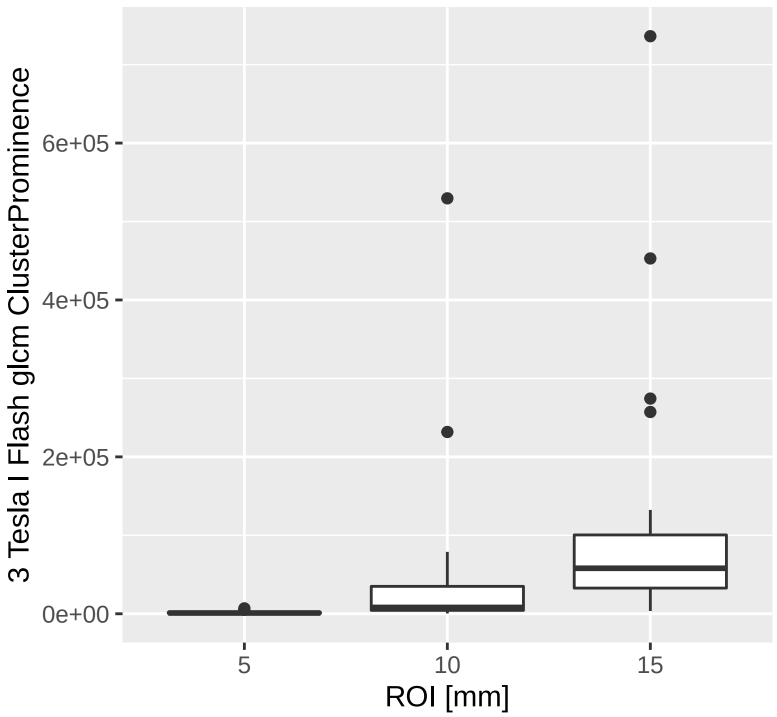
3 Tesla I Flash glcm ClusterProminence
6e+05
4e+05
2e+05
0e+00
5
10
15
ROI [mm]

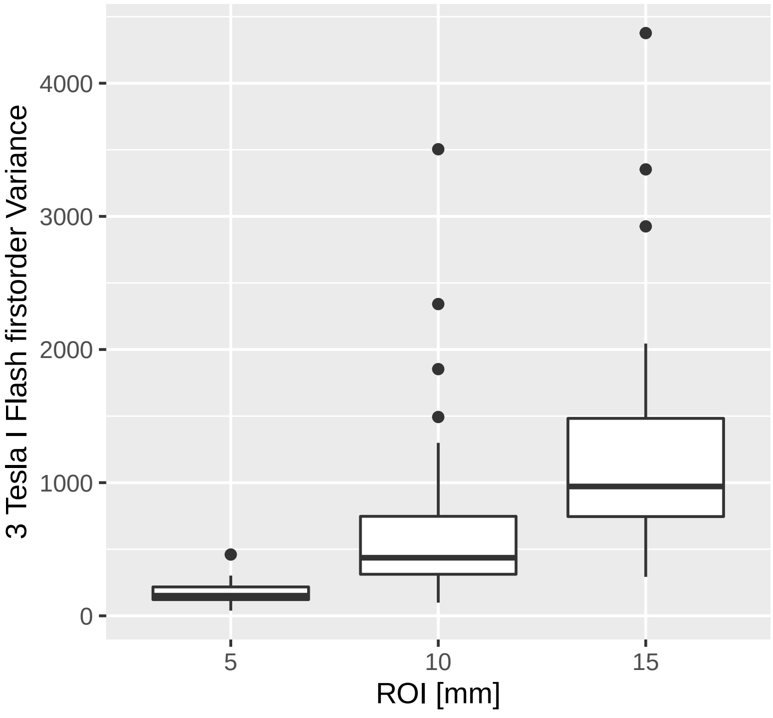
3 Tesla I Flash firstorder Variance
4000
3000
2000
1000
0
5
10
15
ROI [mm]

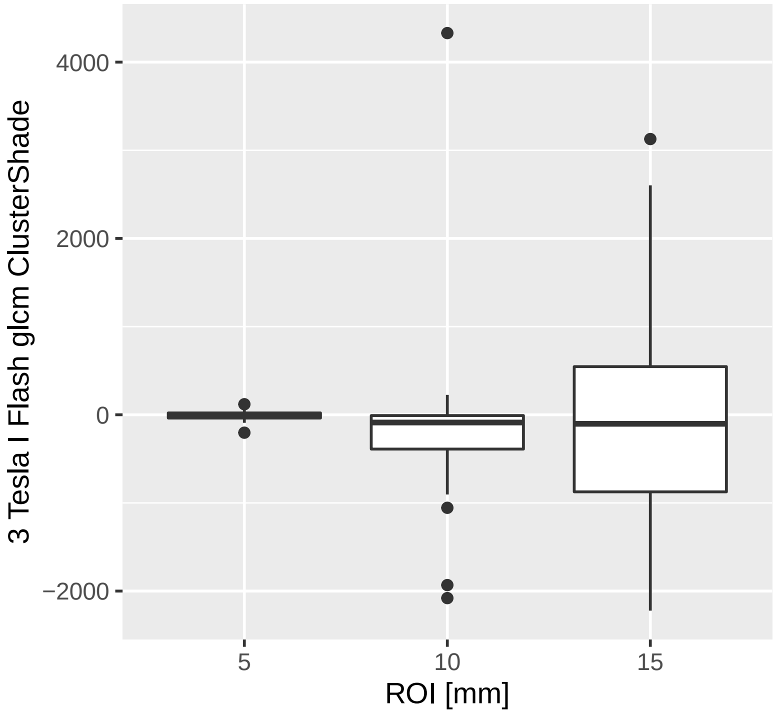
3 Tesla I Flash glcm ClusterShade
4000
2000
0
−2000
5
10
15
ROI [mm]

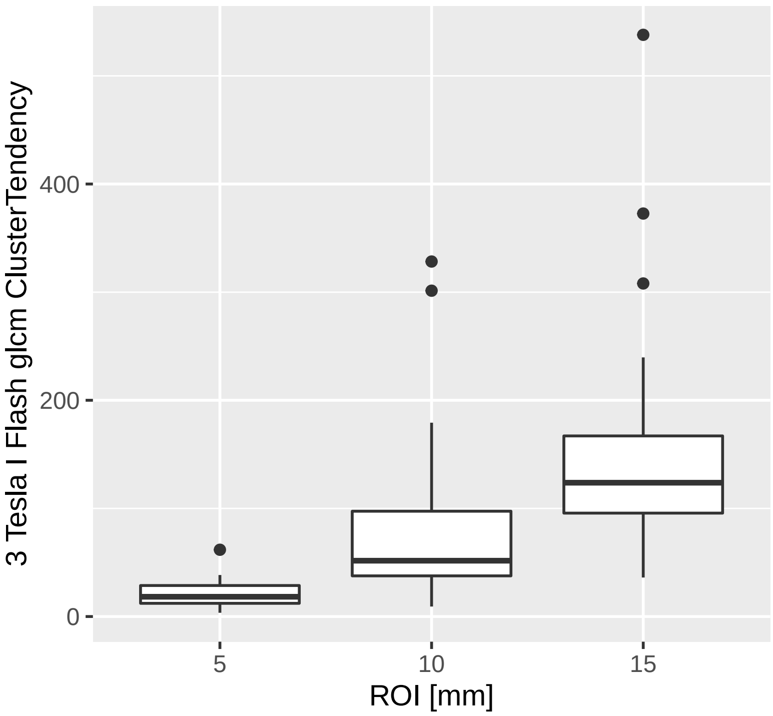
3 Tesla I Flash glcm ClusterTendency
400
200
0
5
10
15
ROI [mm]

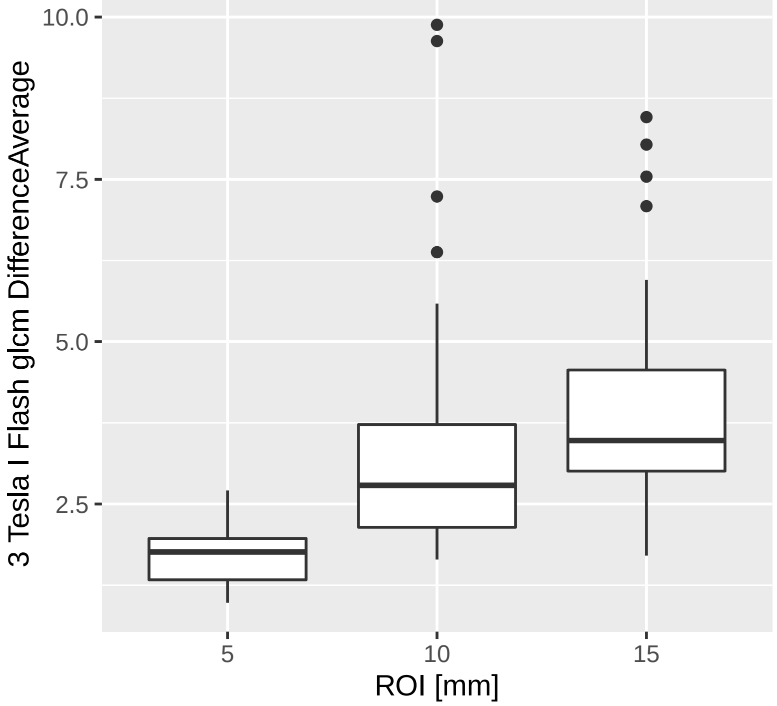
3 Tesla I Flash glcm DifferenceAverage
10.0
7.5
5.0
2.5
5
10
15
ROI [mm]

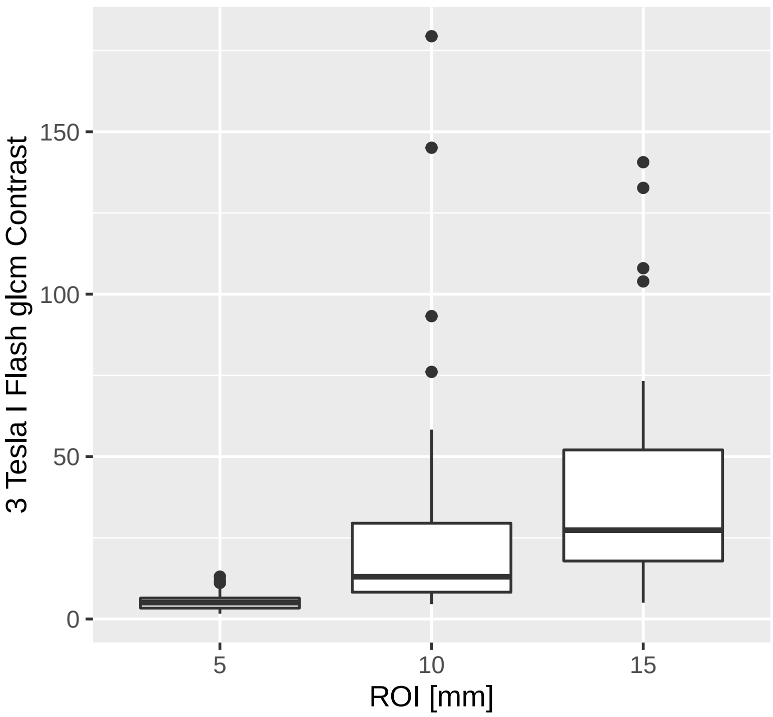
3 Tesla I Flash glcm Contrast
150
100
50
0
5
10
15
ROI [mm]

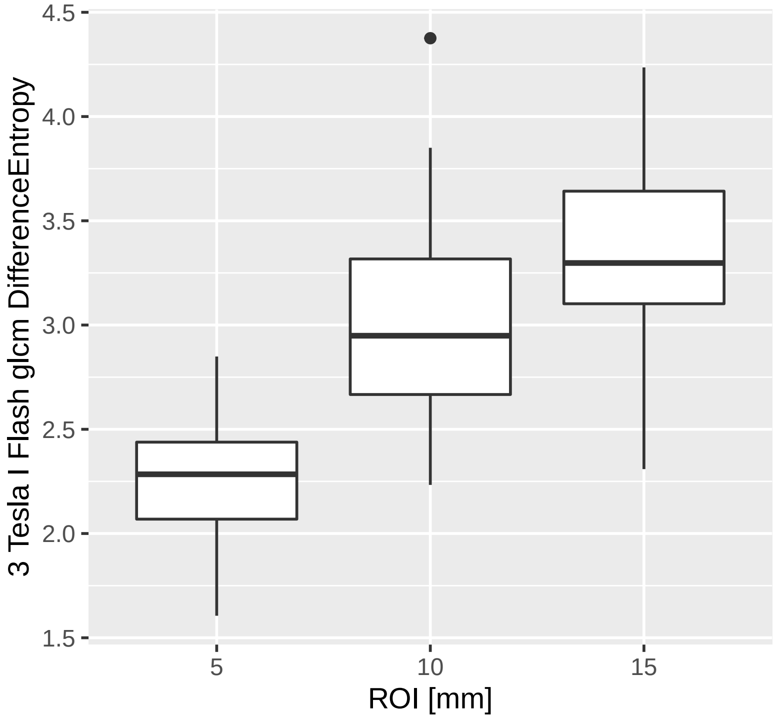
3 Tesla I Flash glcm DifferenceEntropy
4.5
4.0
3.5
3.0
2.5
2.0
1.5
5
10
15
ROI [mm]

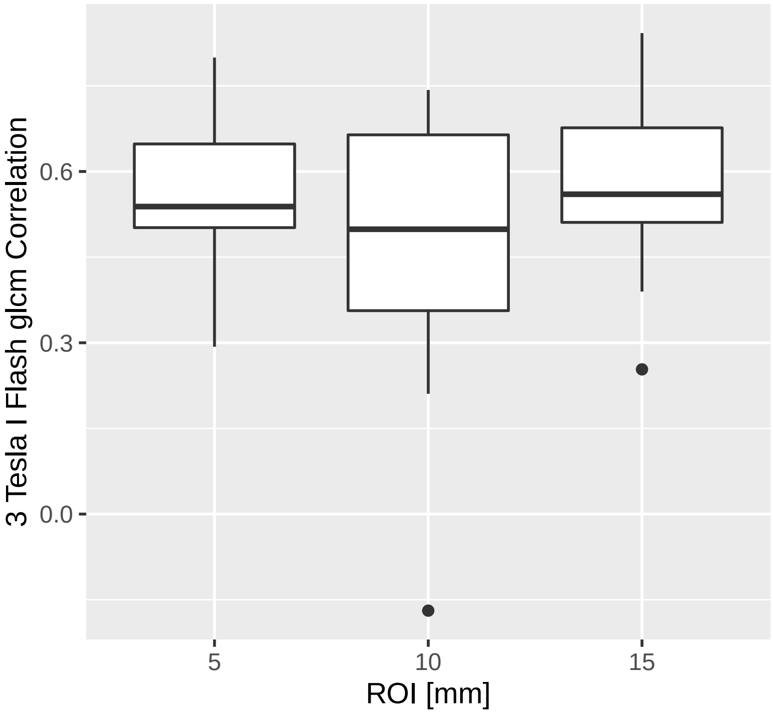
3 Tesla I Flash glcm Correlation
0.6
0.3
0.0
5
10
15
ROI [mm]

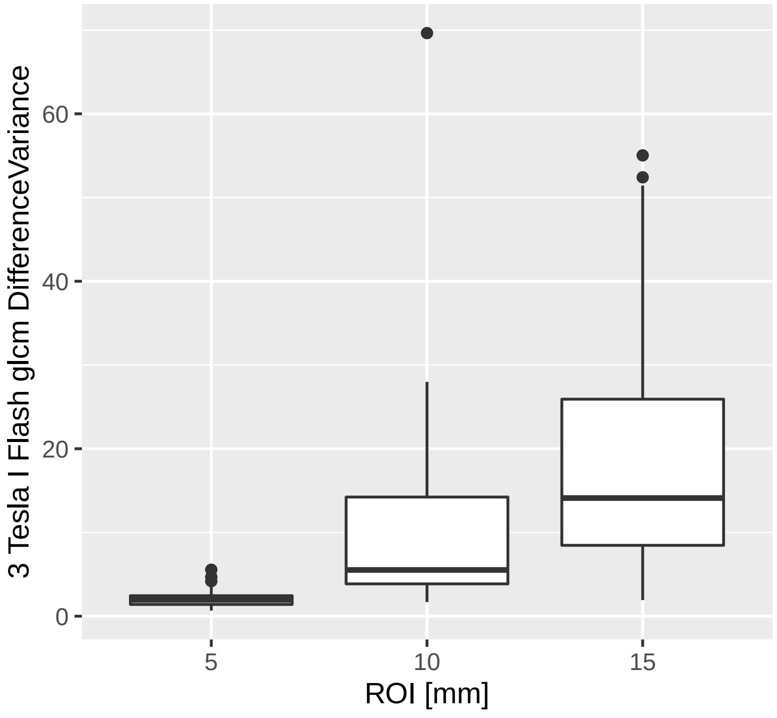
3 Tesla I Flash glcm DifferenceVariance
60
40
20
0
5
10
15
ROI [mm]

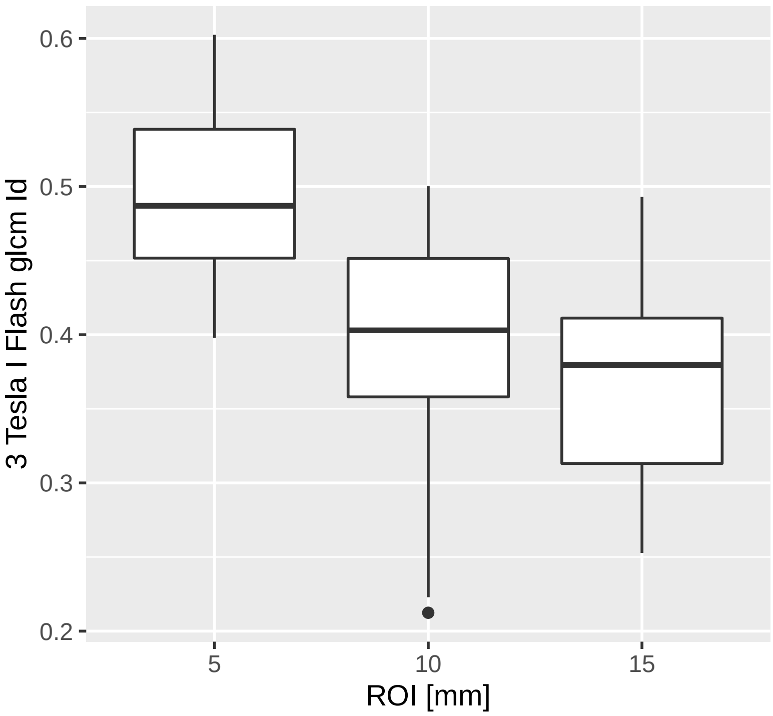
0.6
0.5
0.4
0.3
0.2
3 Tesla I Flash glcm Id
5
10
15
ROI [mm]

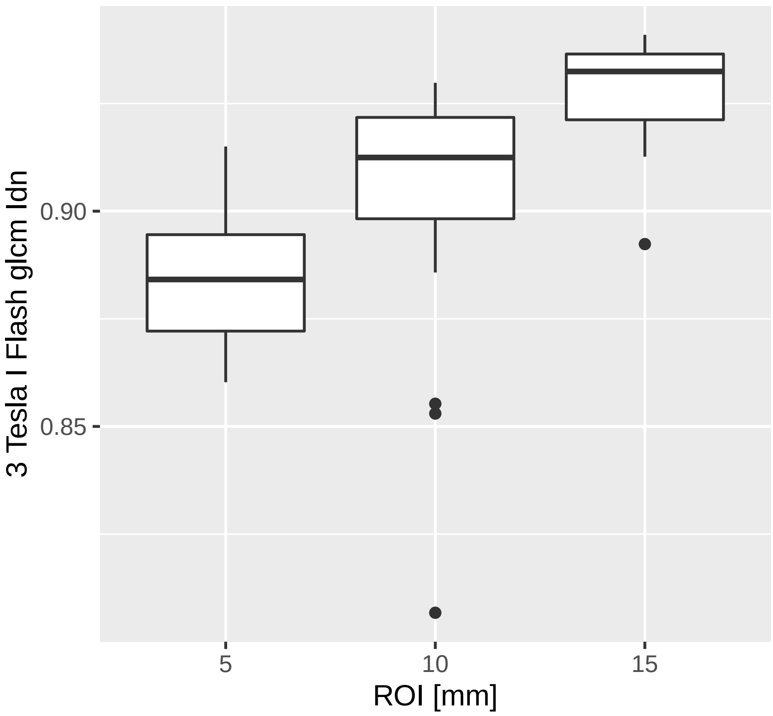
0.90
0.85
3 Tesla I Flash glcm Idn
5
10
15
ROI [mm]

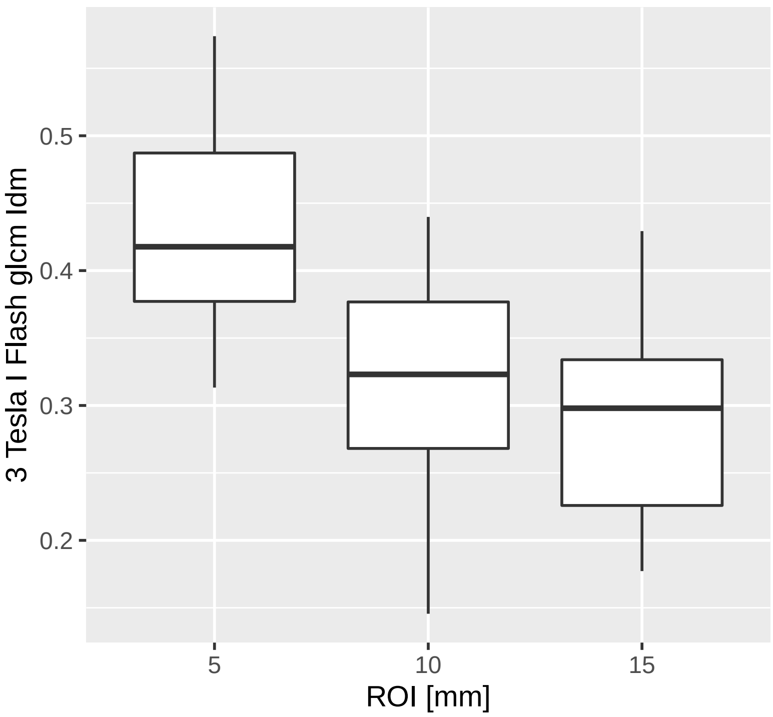
0.5
0.4
0.3
0.2
3 Tesla I Flash glcm Idm
5
10
15
ROI [mm]

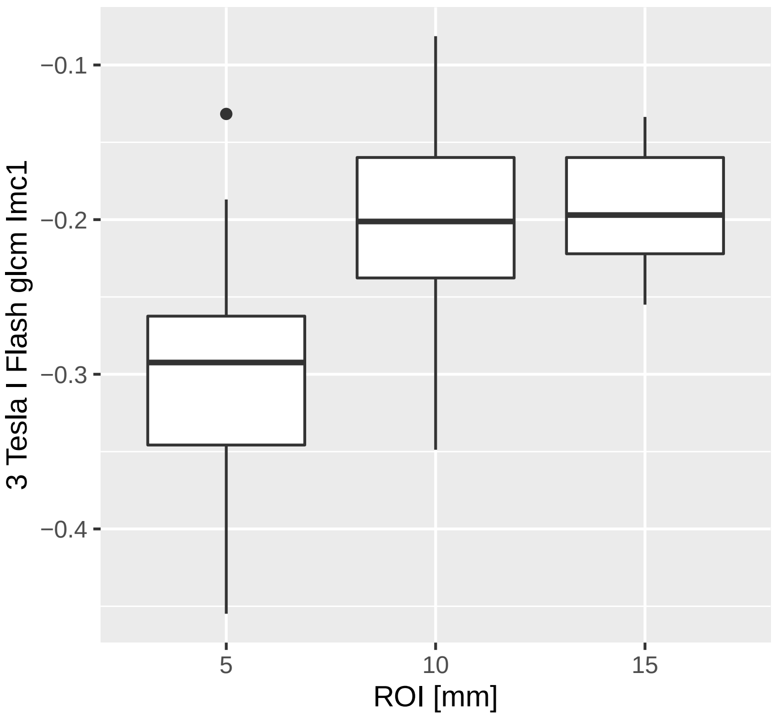
−0.1
−0.2
−0.3
−0.4
3 Tesla I Flash glcm Imc1
5
10
15
ROI [mm]

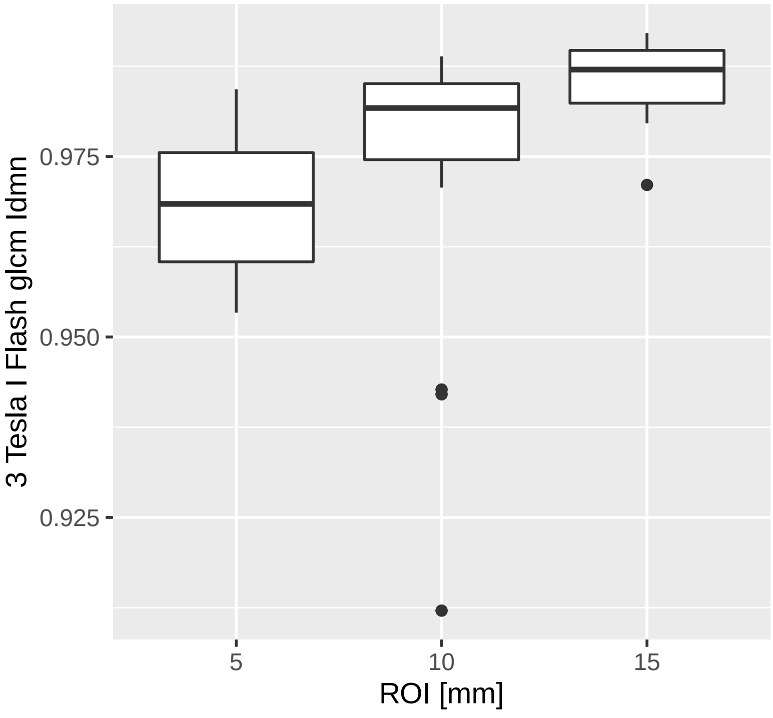
0.975
0.950
0.925
3 Tesla I Flash glcm Idmn
5
10
15
ROI [mm]

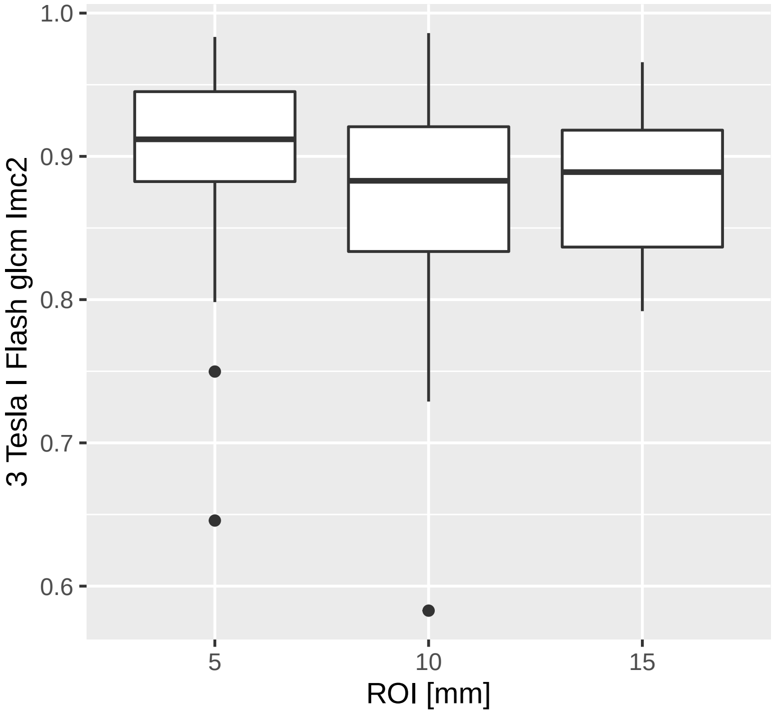
1.0
0.9
0.8
0.7
0.6
3 Tesla I Flash glcm Imc2
5
10
15
ROI [mm]

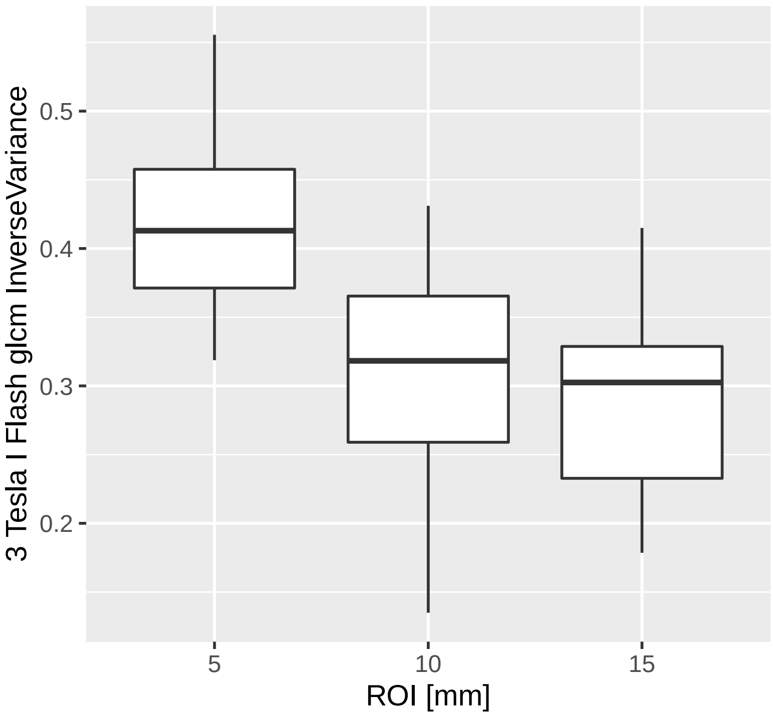
3 Tesla I Flash glcm InverseVariance
0.5
0.4
0.3
0.2
5
10
15
ROI [mm]

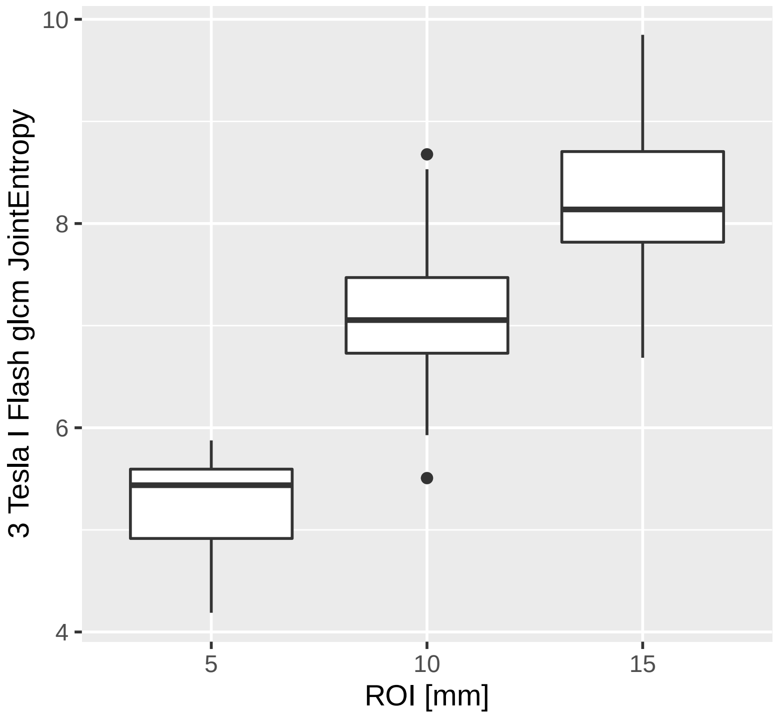
3 Tesla I Flash glcm JointEntropy
10
8
6
4
5
10
15
ROI [mm]

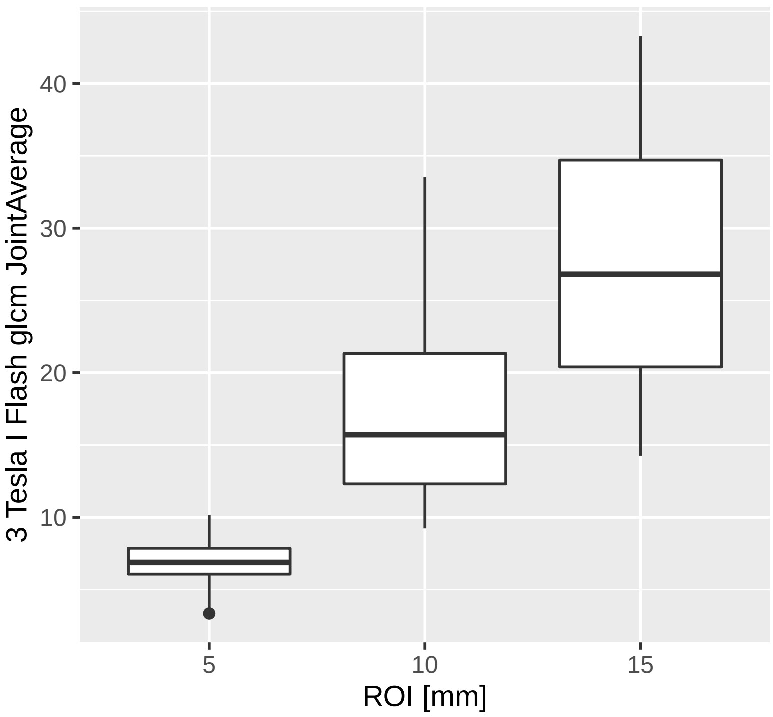
3 Tesla I Flash glcm JointAverage
40
30
20
10
5
10
15
ROI [mm]

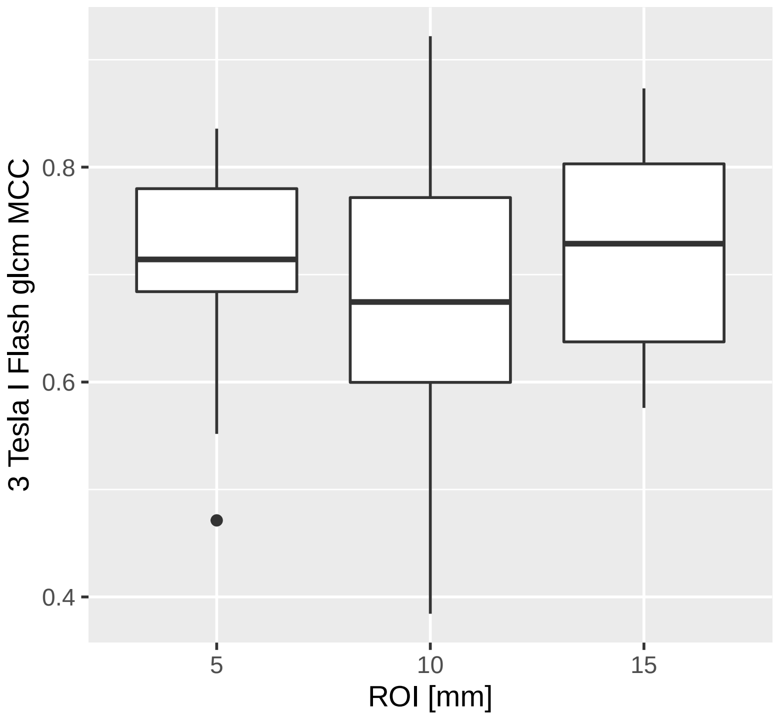
3 Tesla I Flash glcm MCC
0.8
0.6
0.4
5
10
15
ROI [mm]

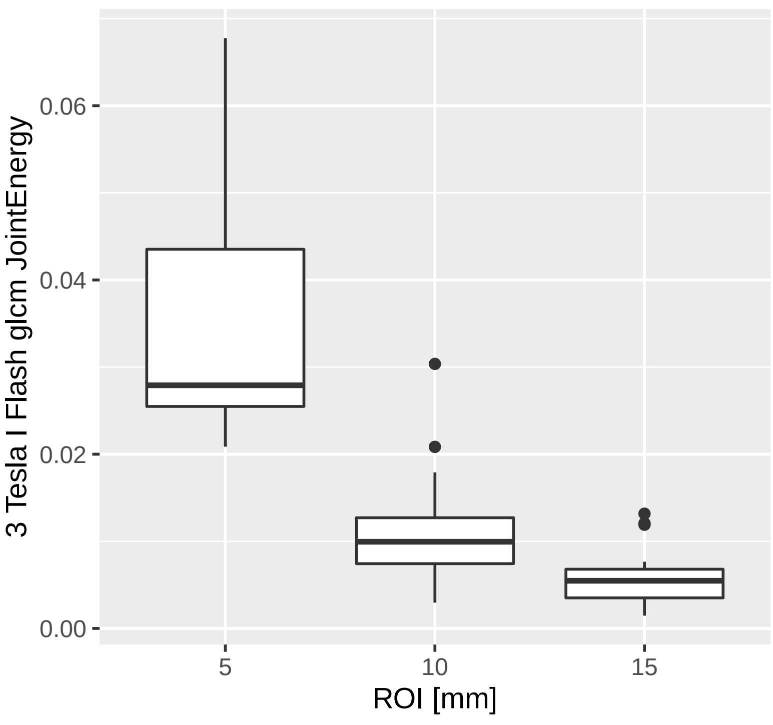
3 Tesla I Flash glcm JointEnergy
0.06
0.04
0.02
0.00
5
10
15
ROI [mm]

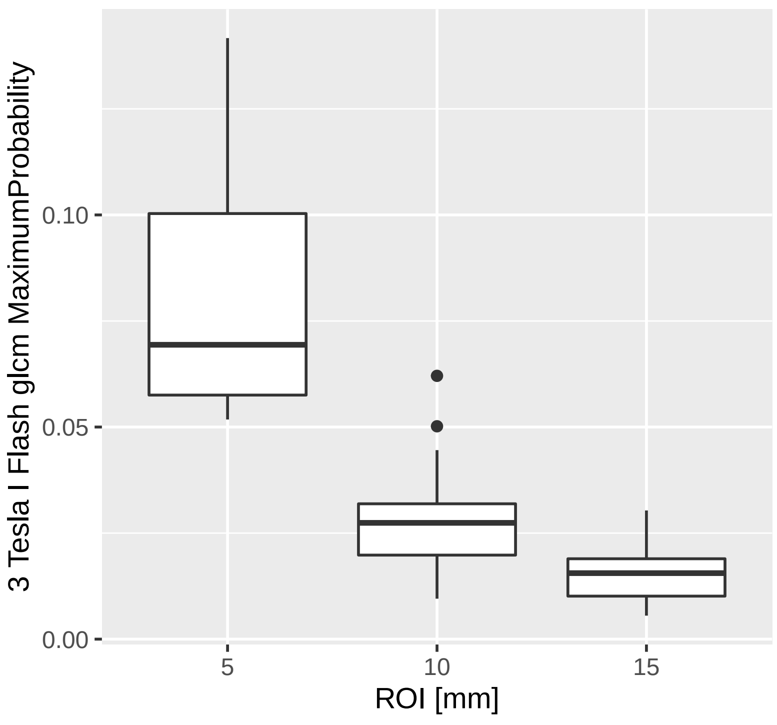
3 Tesla I Flash glcm MaximumProbability
0.10
0.05
0.00
5
10
15
ROI [mm]

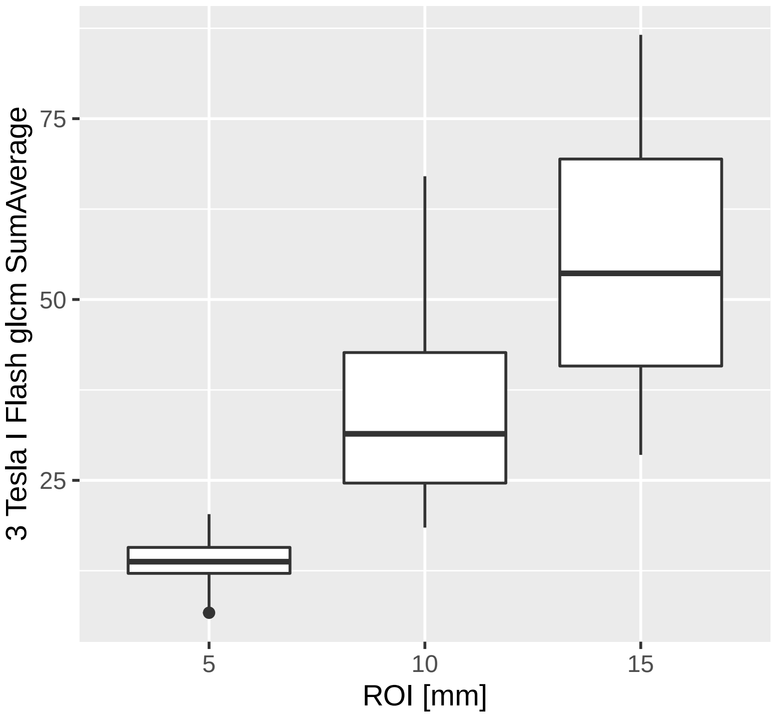
3 Tesla I Flash glcm SumAverage
75
50
25
5
10
15
ROI [mm]

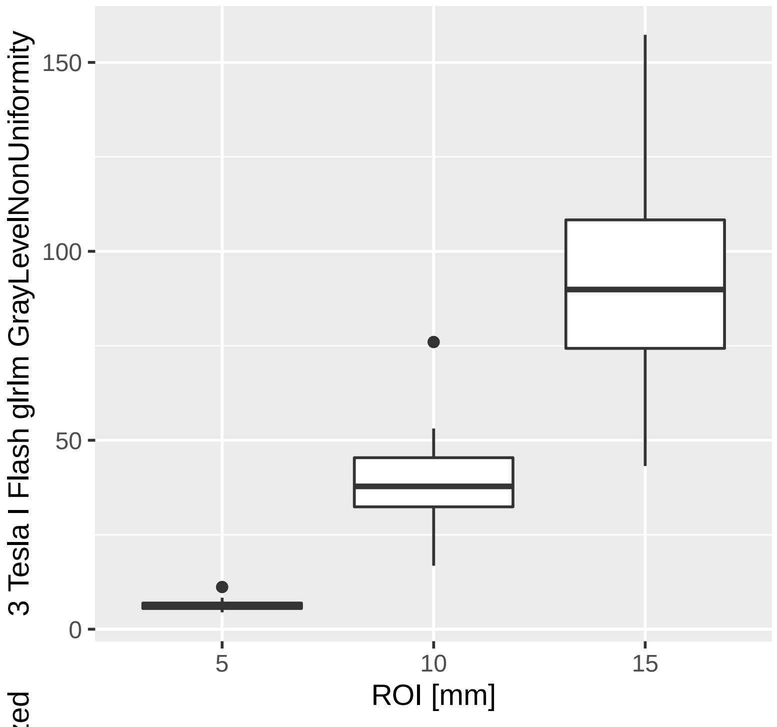
3 Tesla I Flash glrlm GrayLevelNonUniformity
150
100
50
0
5
10
15
ROI [mm]

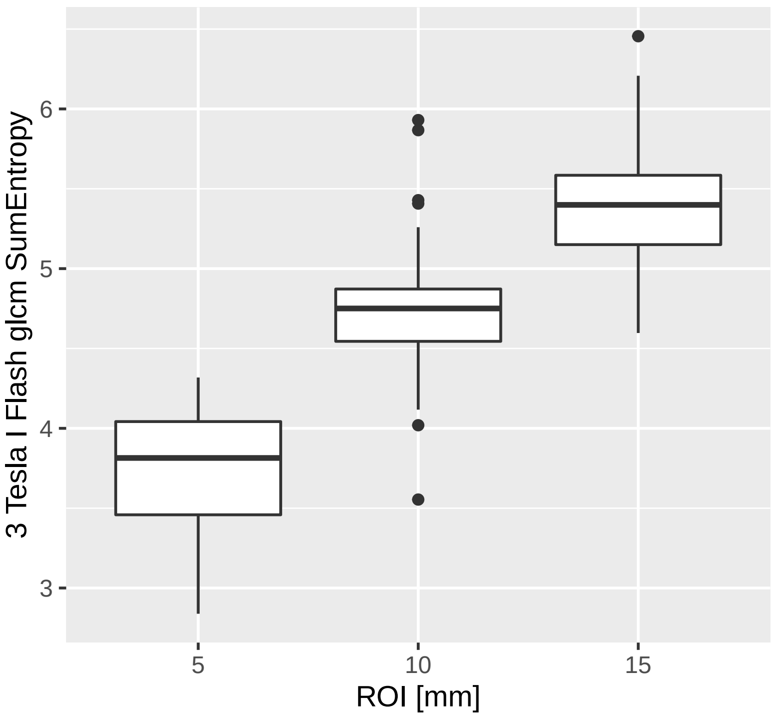
3 Tesla I Flash glcm SumEntropy
6
5
4
3
5
10
15
ROI [mm]

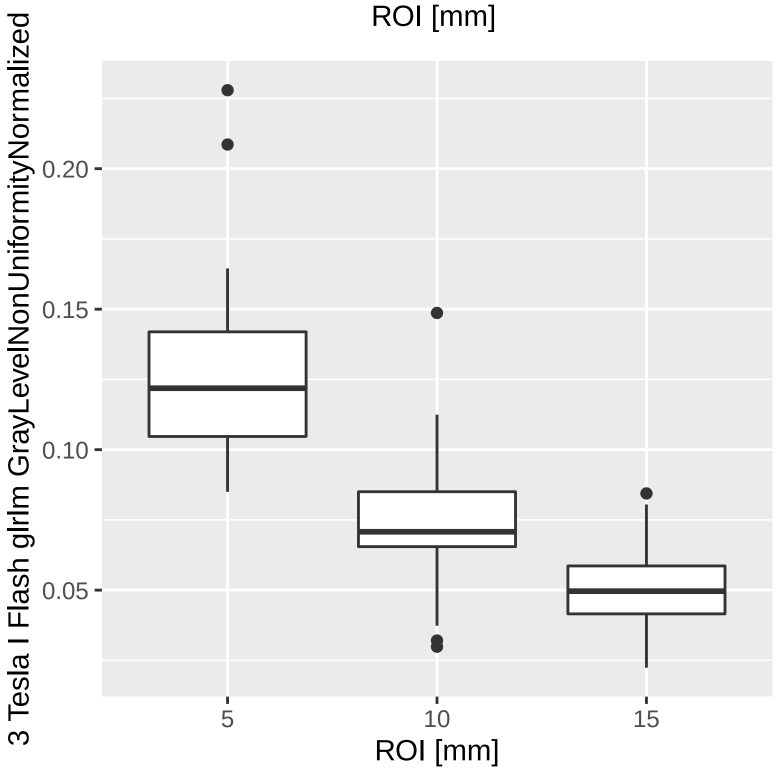
3 Tesla I Flash glrlm GrayLevelNonUniformityNormalized
0.20
0.15
0.10
0.05
5
10
15
ROI [mm]

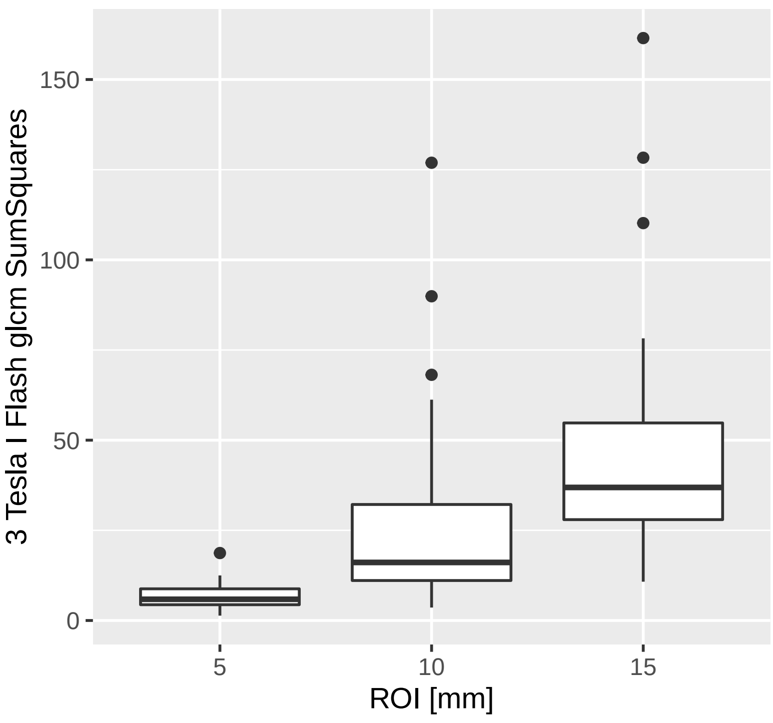
3 Tesla I Flash glcm SumSquares
150
100
50
0
5
10
15
ROI [mm]

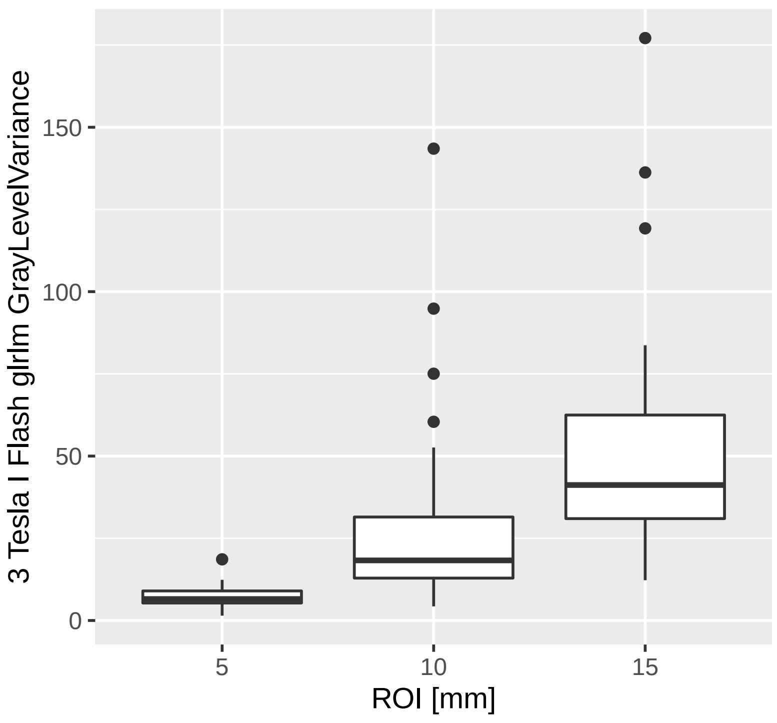
3 Tesla I Flash glrlm GrayLevelVariance
150
100
50
0
5
10
15
ROI [mm]

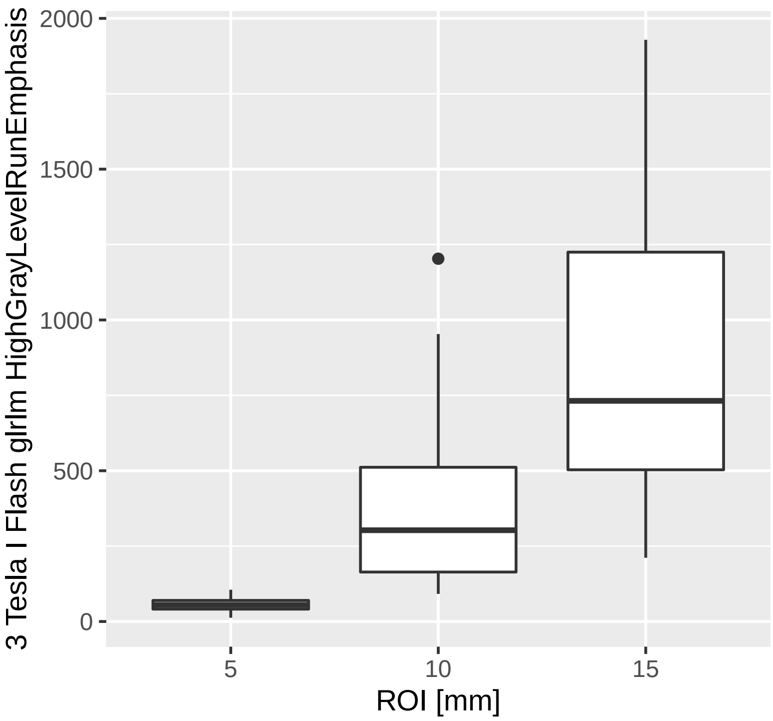
3 Tesla I Flash glrlm HighGrayLevelRunEmphasis
ROI [mm]

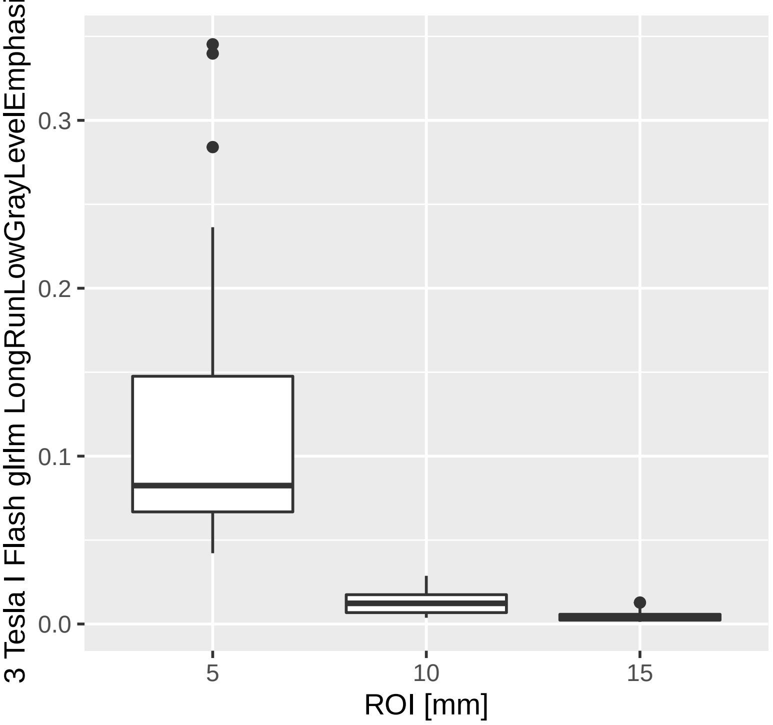
3 Tesla I Flash glrlm LongRunLowGrayLevelEmphasis
ROI [mm]

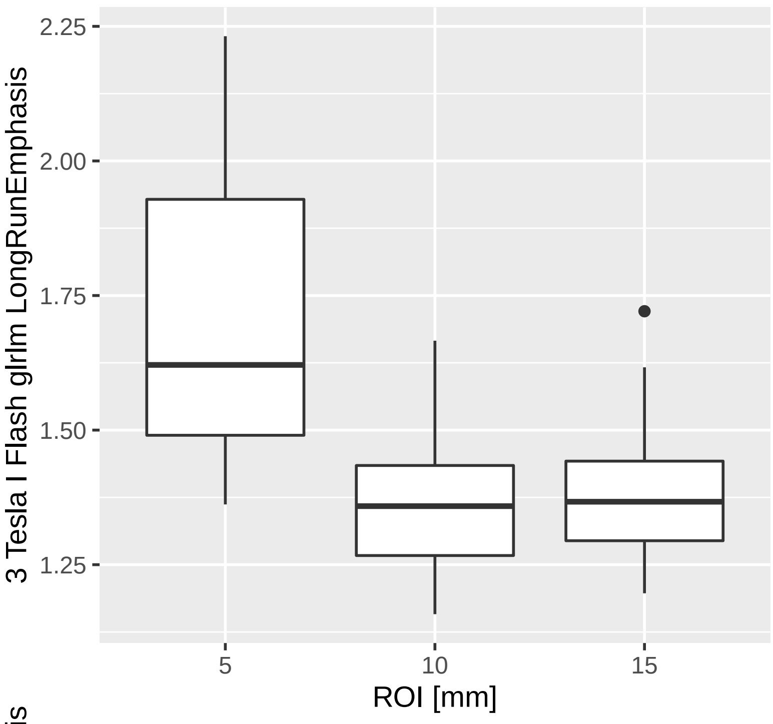
3 Tesla I Flash glrlm LongRunEmphasis
ROI [mm]

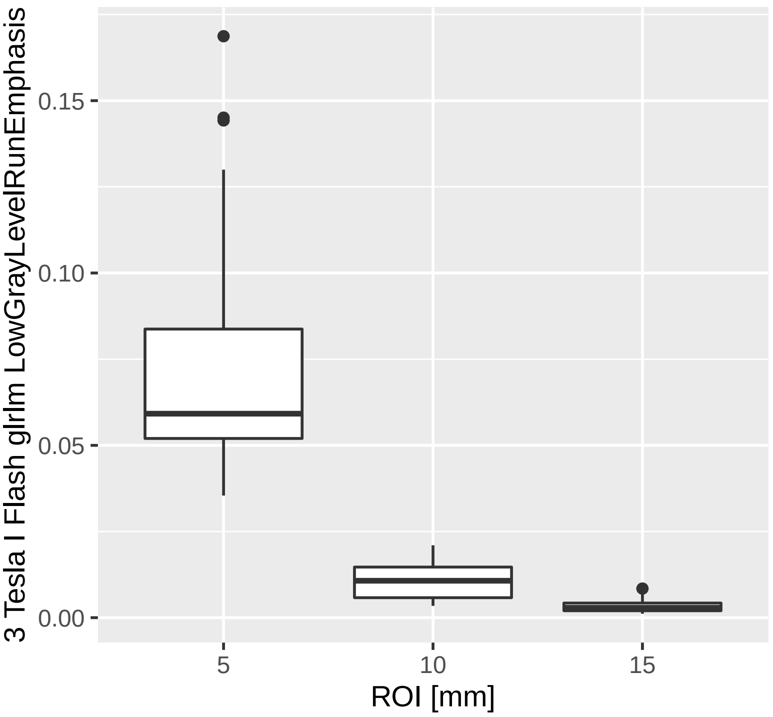
3 Tesla I Flash glrlm LowGrayLevelRunEmphasis
ROI [mm]

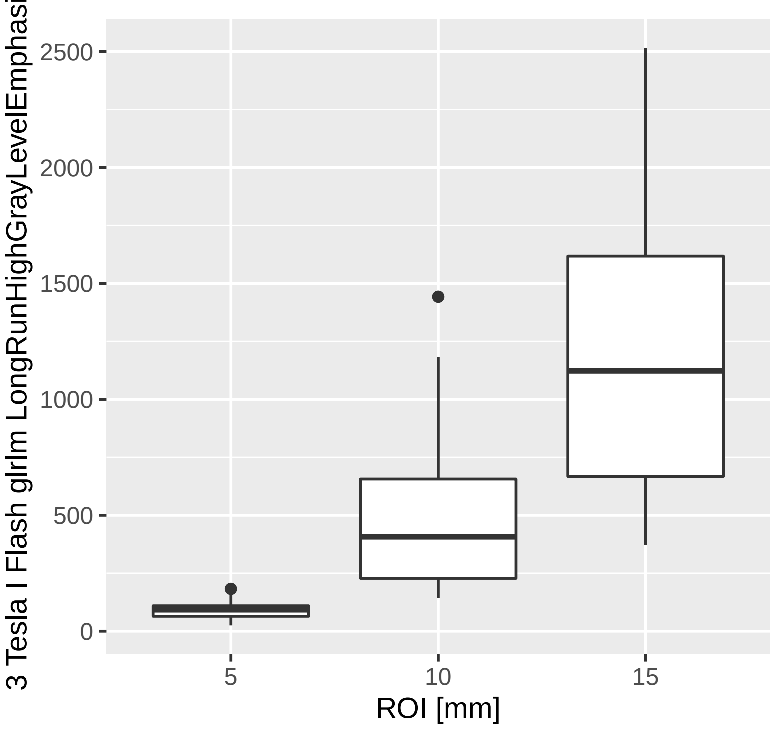
3 Tesla I Flash glrlm LongRunHighGrayLevelEmphasis
ROI [mm]

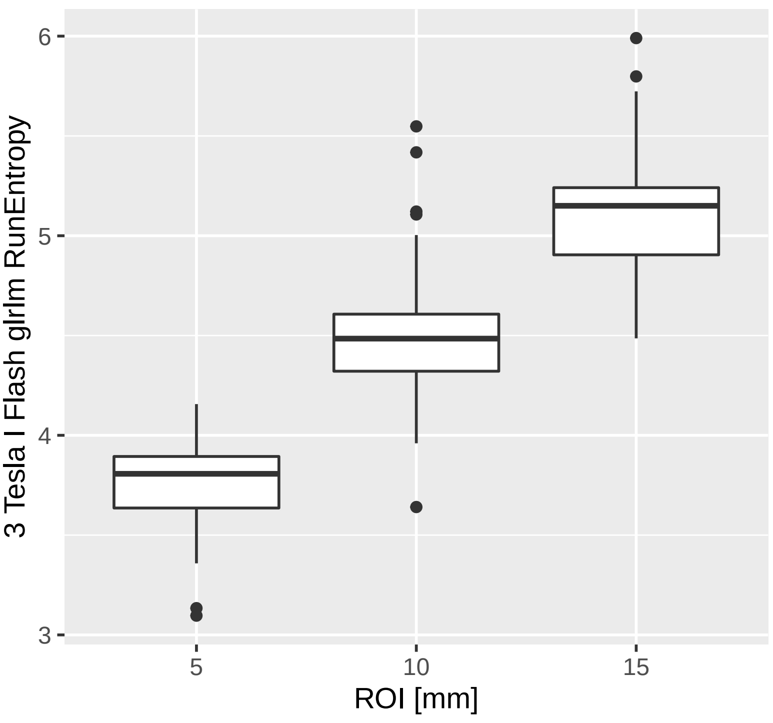
3 Tesla I Flash glrlm RunEntropy
ROI [mm]

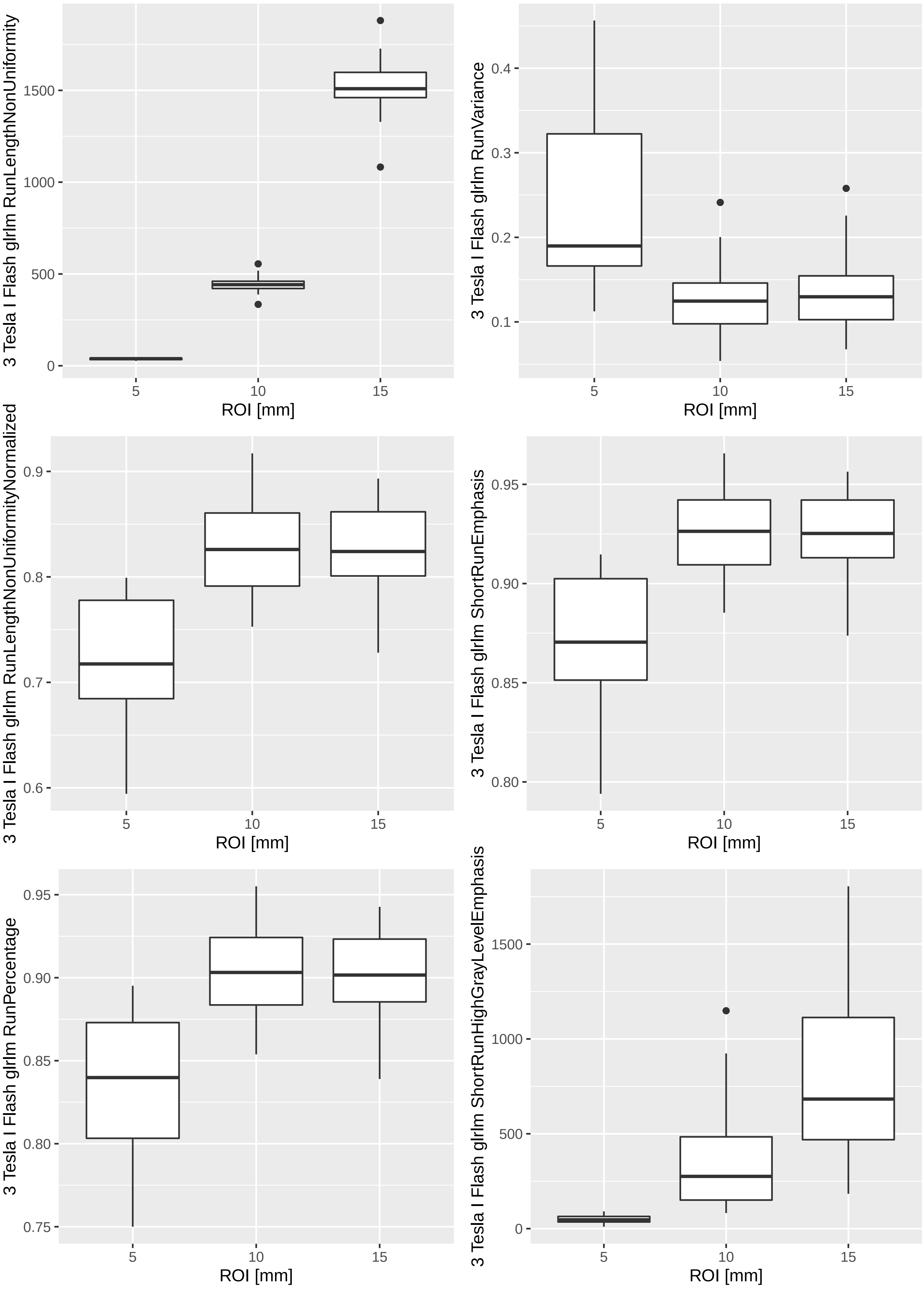

3 Tesla I Flash glrlm RunLengthNonUniformity
ROI [mm]
3 Tesla I Flash glrlm RunVariance
ROI [mm]
3 Tesla I Flash glrlm RunLengthNonUniformityNormalized
ROI [mm]
3 Tesla I Flash glrlm ShortRunEmphasis
ROI [mm]
3 Tesla I Flash glrlm RunPercentage
ROI [mm]
3 Tesla I Flash glrlm ShortRunHighGrayLevelEmphasis
ROI [mm]

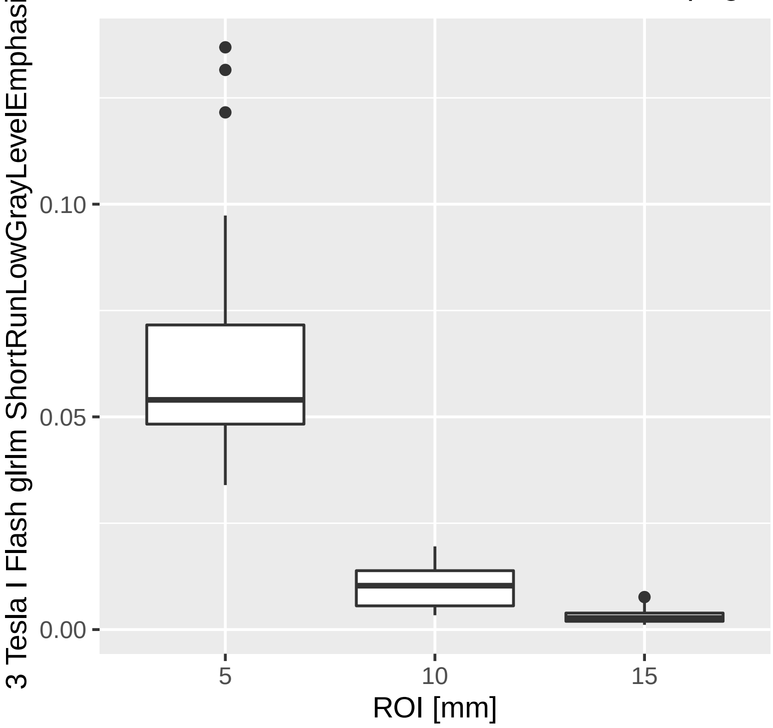
3 Tesla I Flash glrlm ShortRunLowGrayLevelEmphasis
0.10
0.05
0.00
5
10
15
ROI [mm]

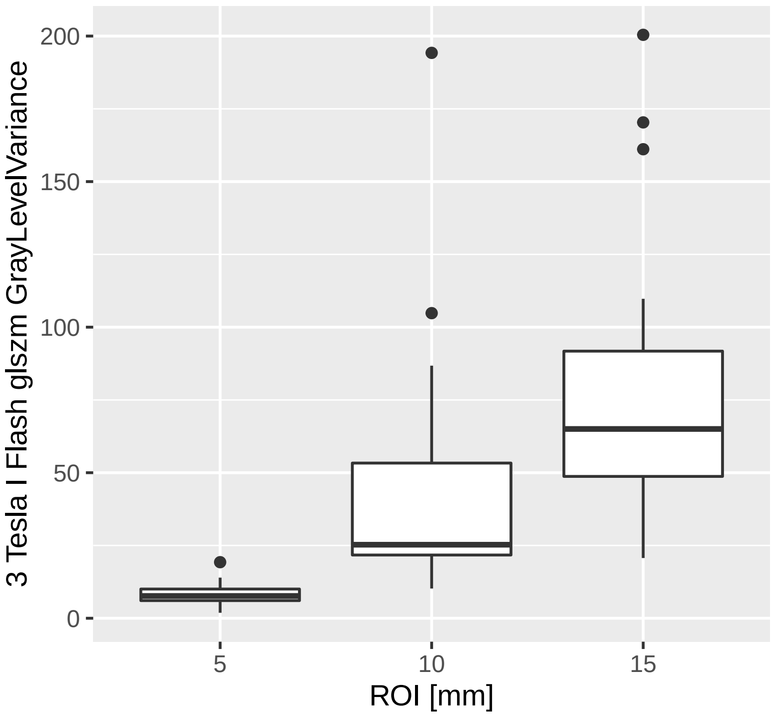
3 Tesla I Flash glszm GrayLevelVariance
200
150
100
50
0
5
10
15
ROI [mm]

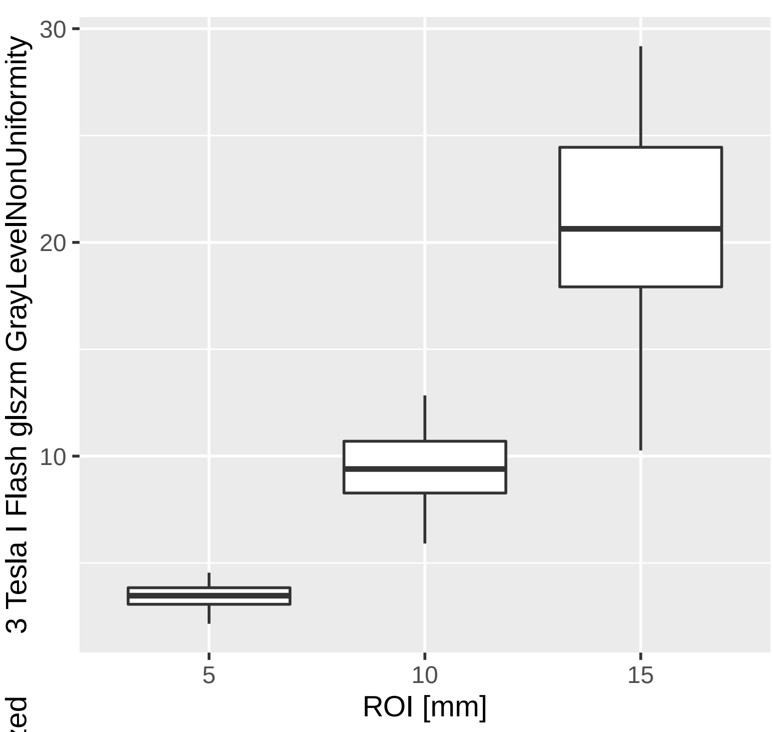
3 Tesla I Flash glszm GrayLevelNonUniformity
30
20
10
5
10
15
ROI [mm]

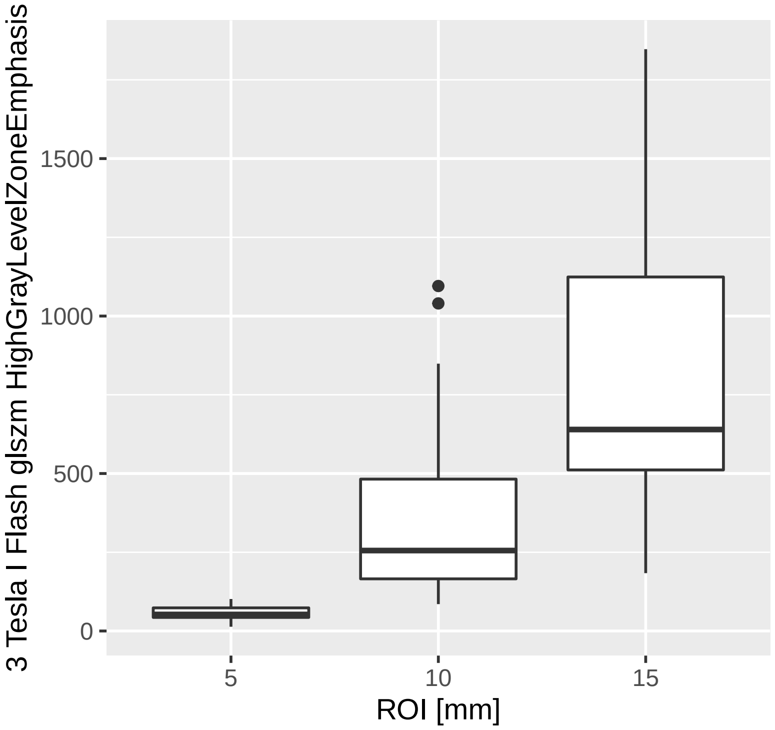
3 Tesla I Flash glszm HighGrayLevelZoneEmphasis
1500
1000
500
0
5
10
15
ROI [mm]

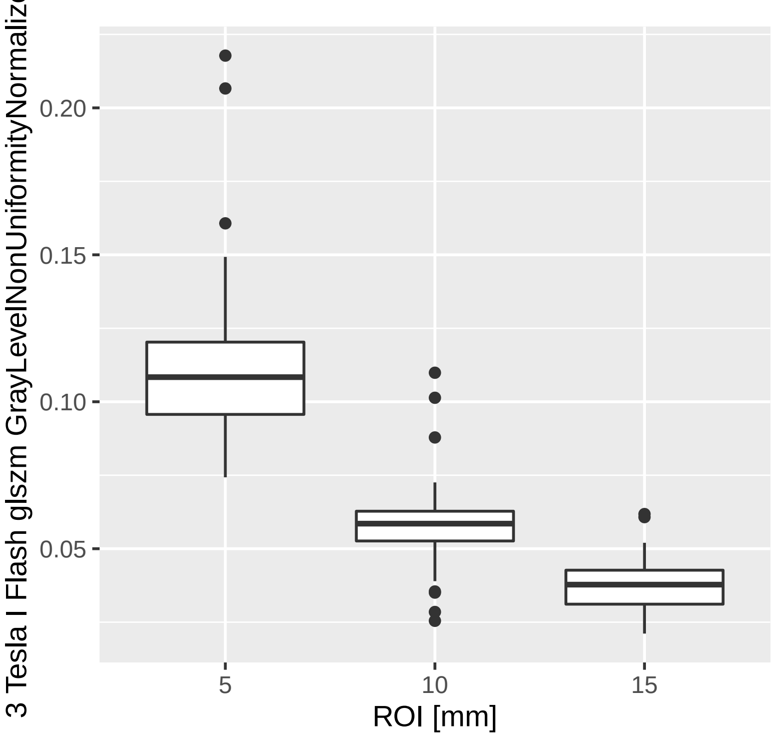
3 Tesla I Flash glszm GrayLevelNonUniformityNormalized
0.20
0.15
0.10
0.05
5
10
15
ROI [mm]

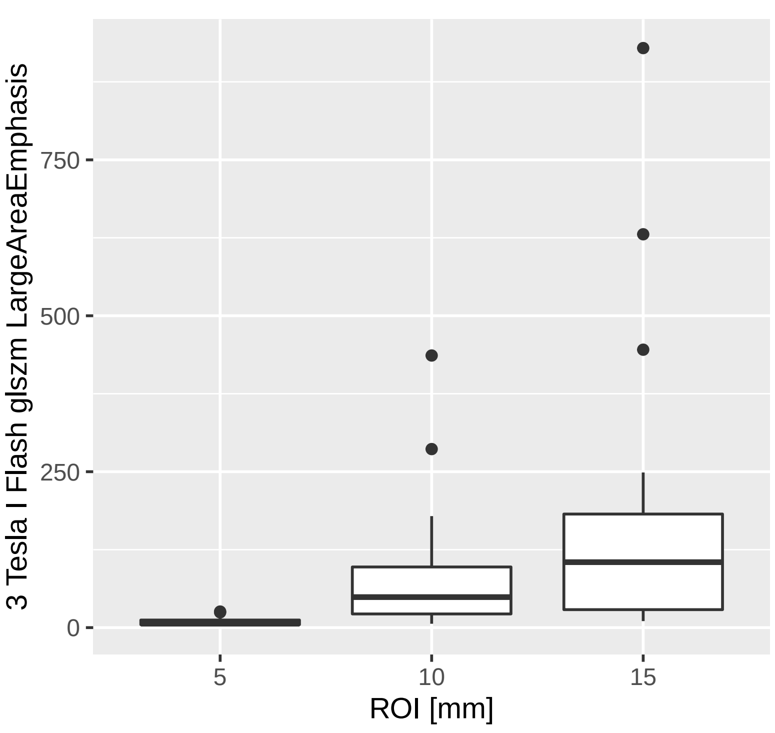
3 Tesla I Flash glszm LargeAreaEmphasis
750
500
250
0
5
10
15
ROI [mm]

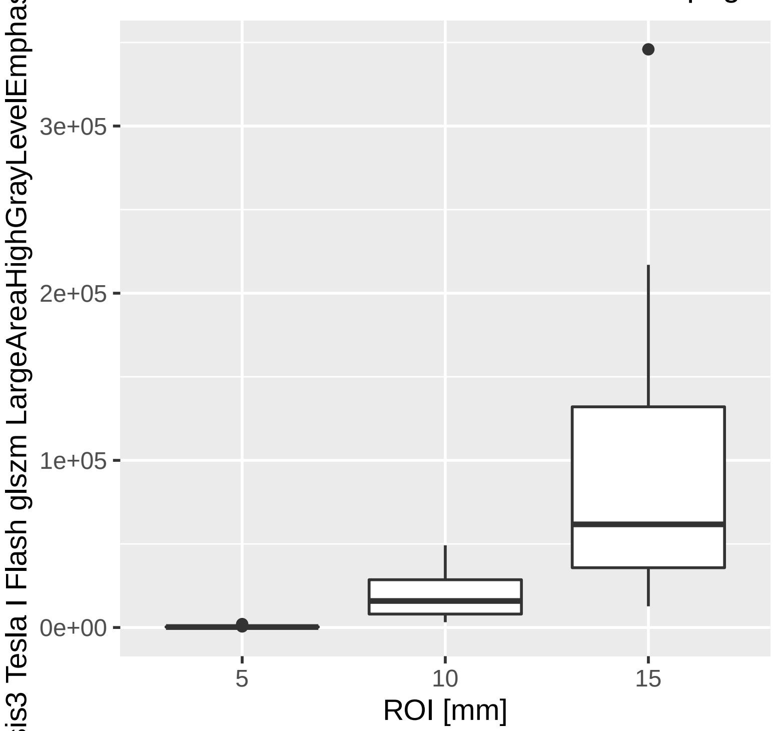
3 Tesla I Flash glszm LargeAreaHighGrayLevelEmphasis
3e+05
2e+05
1e+05
0e+00
5
10
15
ROI [mm]

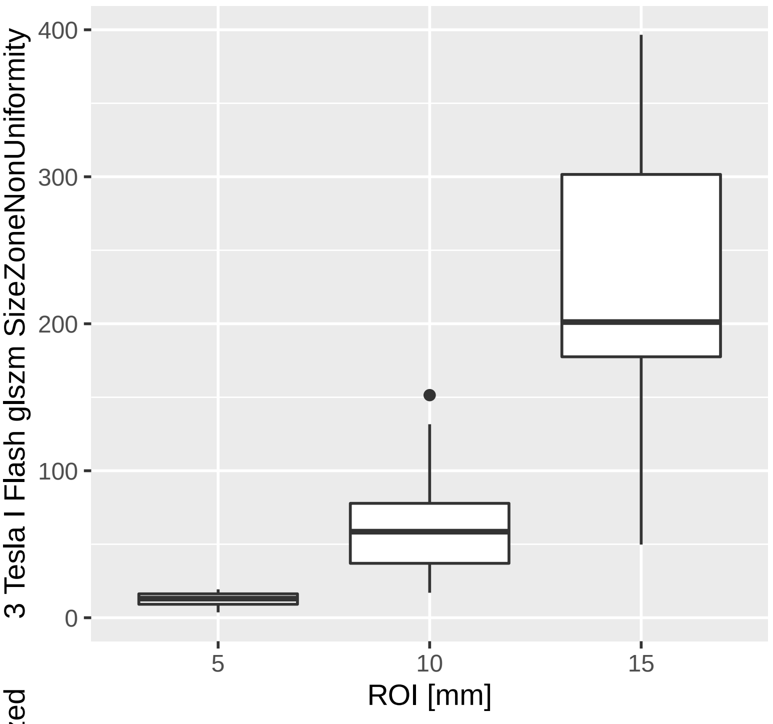
3 Tesla I Flash glszm SizeZoneNonUniformity
400
300
200
100
0
5
10
15
ROI [mm]

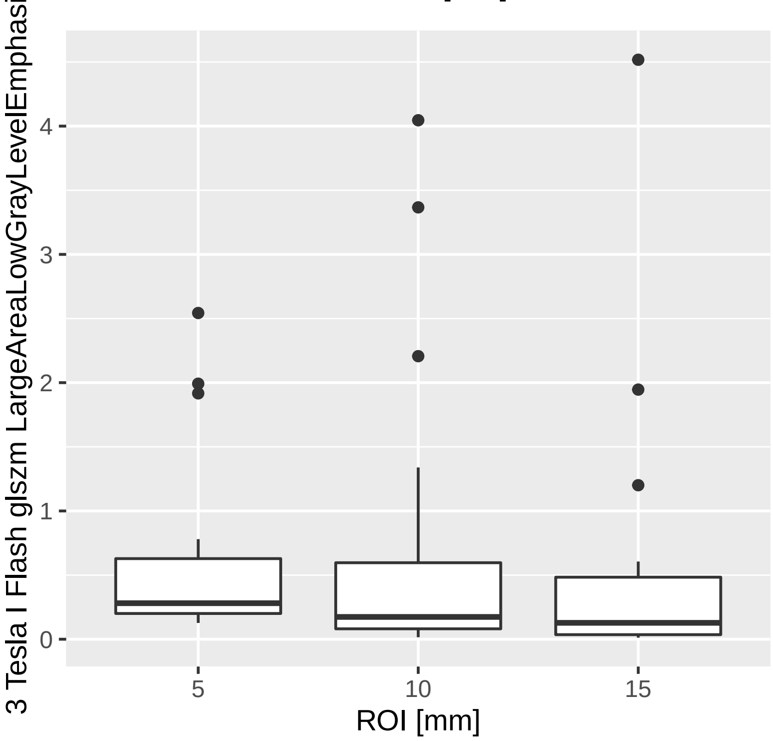
3 Tesla I Flash glszm LargeAreaLowGrayLevelEmphasis
4
3
2
1
0
5
10
15
ROI [mm]

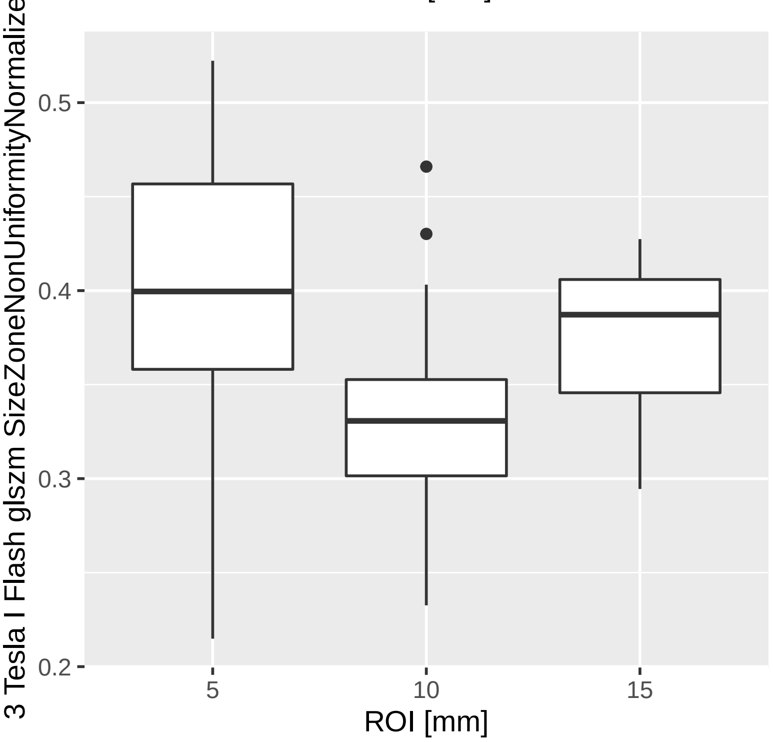
3 Tesla I Flash glszm SizeZoneNonUniformityNormalized
0.5
0.4
0.3
0.2
5
10
15
ROI [mm]

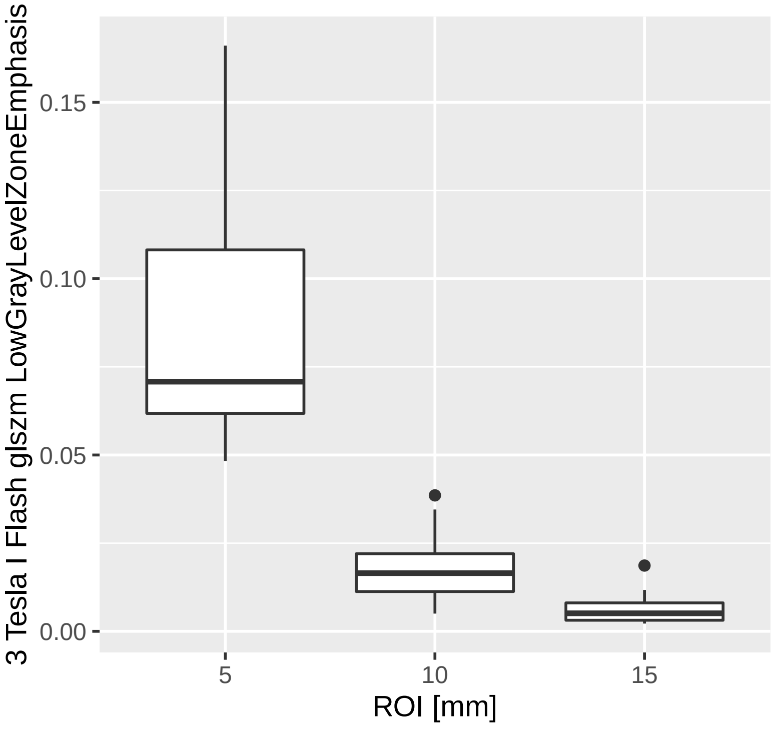
3 Tesla I Flash glszm LowGrayLevelZoneEmphasis
0.15
0.10
0.05
0.00
5
10
15
ROI [mm]

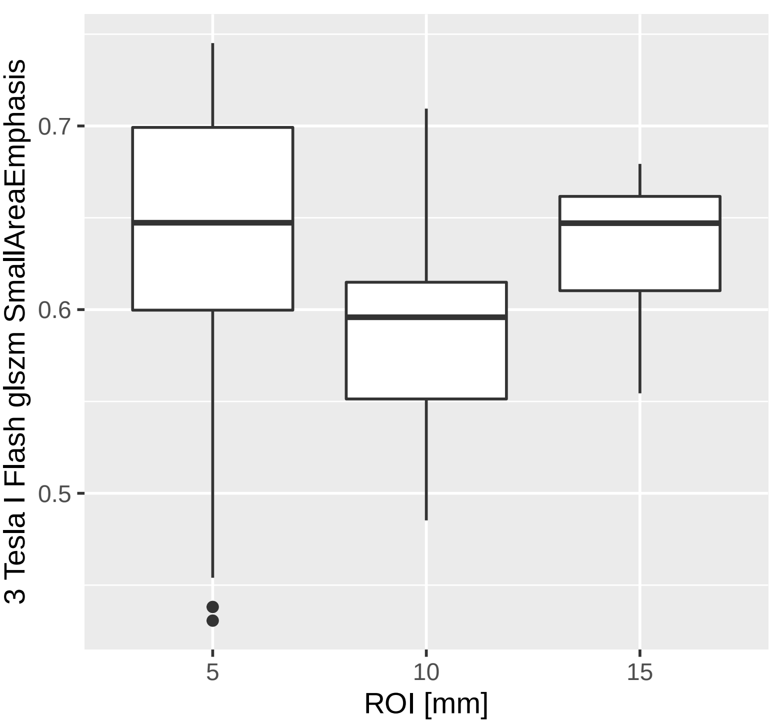
3 Tesla I Flash glszm SmallAreaEmphasis
0.7
0.6
0.5
5
10
15
ROI [mm]

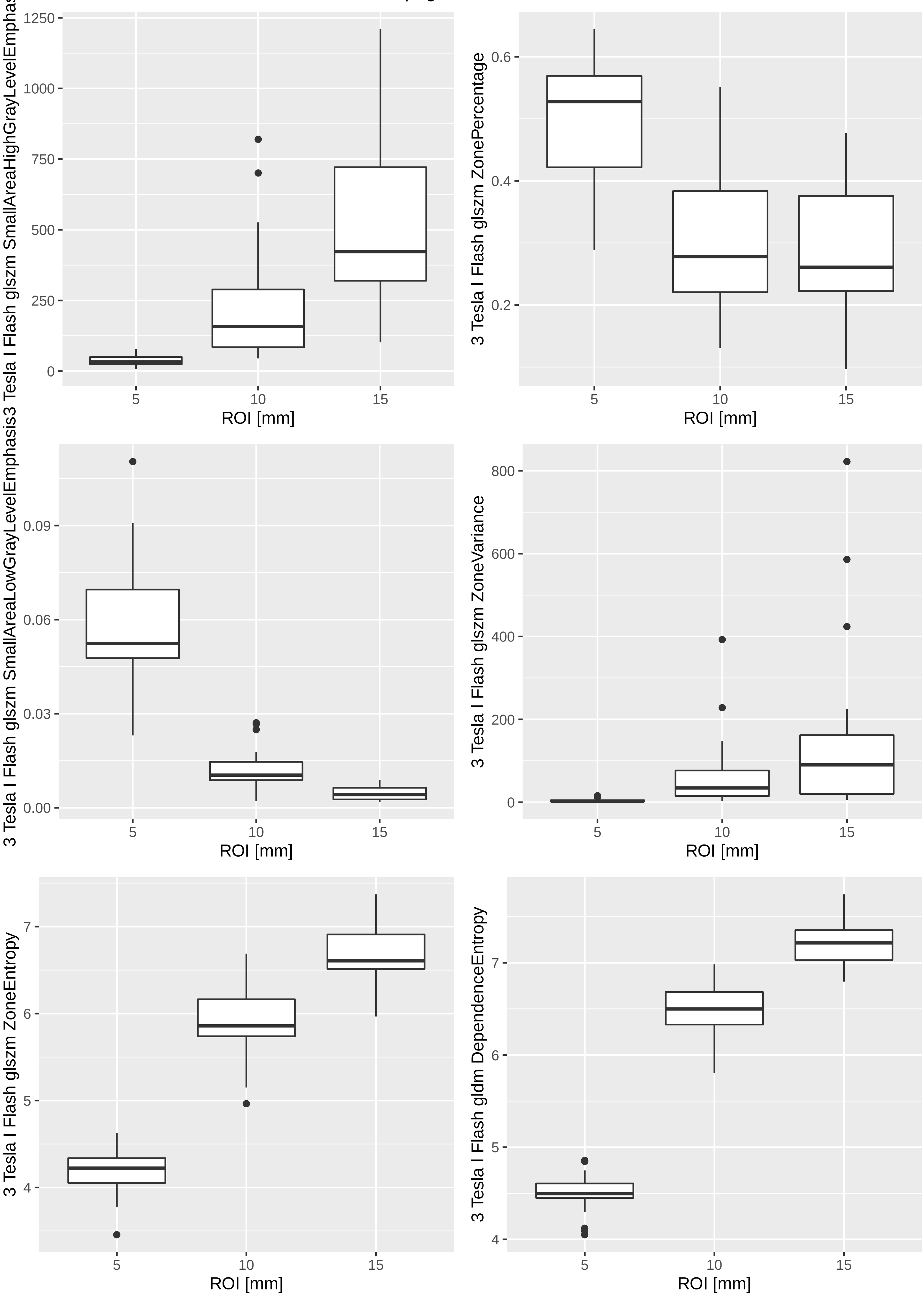
3 Tesla I Flash glszm SmallAreaHighGrayLevelEmphasis
0
250
500
750
1000
1250
5
10
15
ROI [mm]
3 Tesla I Flash glszm ZonePercentage
0.2
0.4
0.6
5
10
15
ROI [mm]
3 Tesla I Flash glszm SmallAreaLowGrayLevelEmphasis
0.00
0.03
0.06
0.09
5
10
15
ROI [mm]
3 Tesla I Flash glszm ZoneVariance
0
200
400
600
800
5
10
15
ROI [mm]
3 Tesla I Flash glszm ZoneEntropy
4
5
6
7
5
10
15
ROI [mm]
3 Tesla I Flash gldm DependenceEntropy
4
5
6
7
5
10
15
ROI [mm]

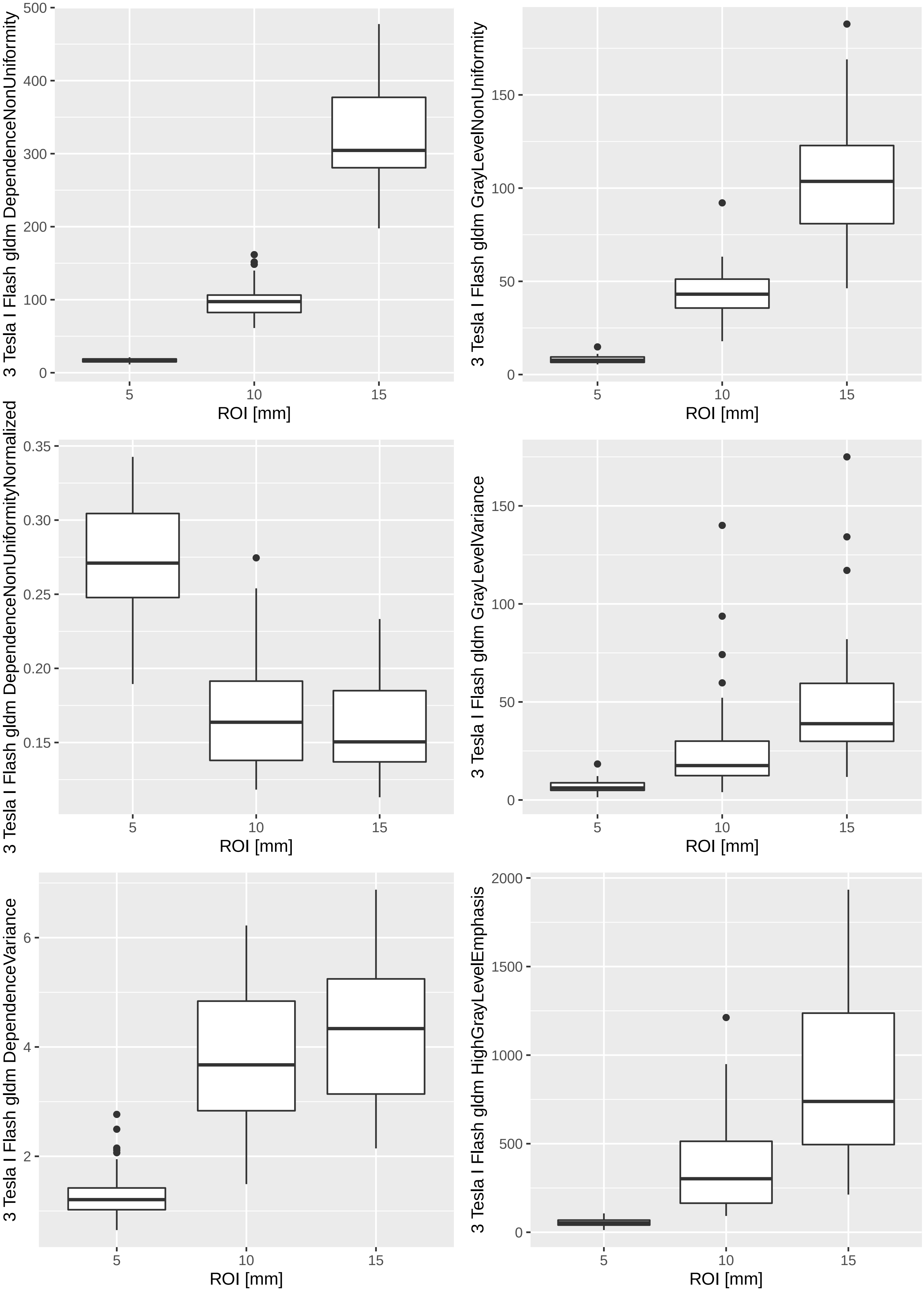
3 Tesla I Flash gldm DependenceNonUniformity
ROI [mm]
3 Tesla I Flash gldm GrayLevelNonUniformity
ROI [mm]
3 Tesla I Flash gldm DependenceNonUniformityNormalized
ROI [mm]
3 Tesla I Flash gldm GrayLevelVariance
ROI [mm]
3 Tesla I Flash gldm DependenceVariance
ROI [mm]
3 Tesla I Flash gldm HighGrayLevelEmphasis
ROI [mm]

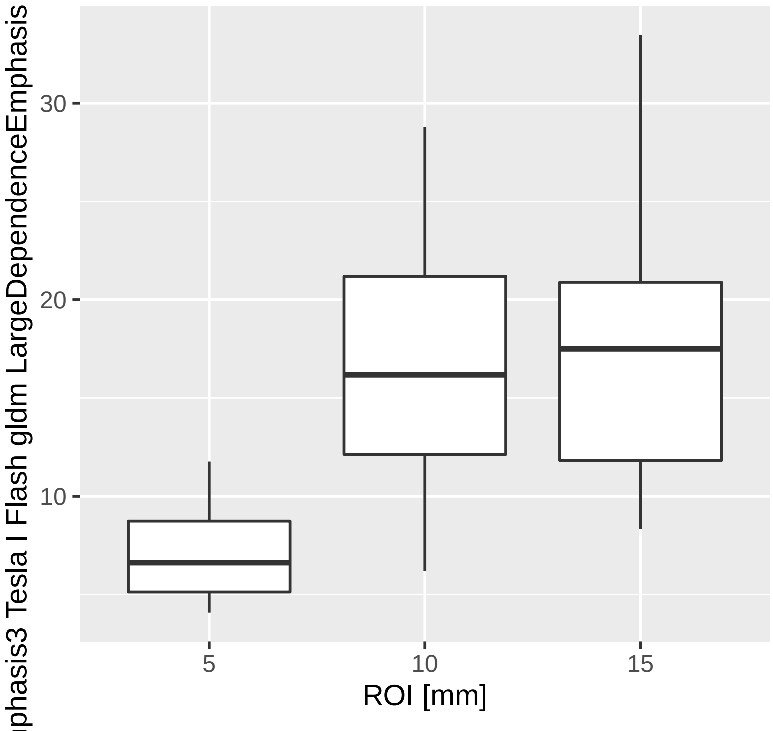
3 Tesla I Flash gldm LargeDependenceEmphasis
30
20
10
5
10
15
ROI [mm]

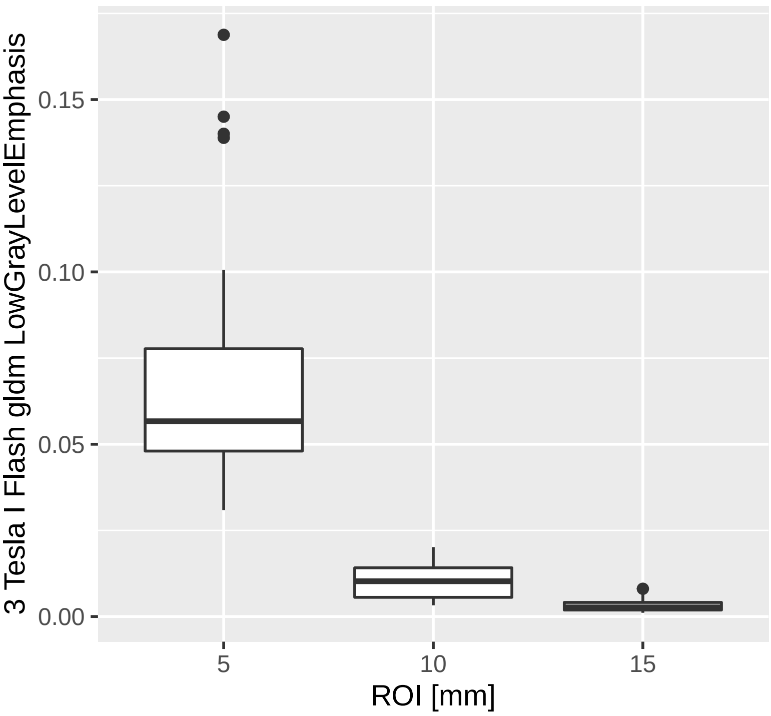
3 Tesla I Flash gldm LowGrayLevelEmphasis
0.15
0.10
0.05
0.00
5
10
15
ROI [mm]

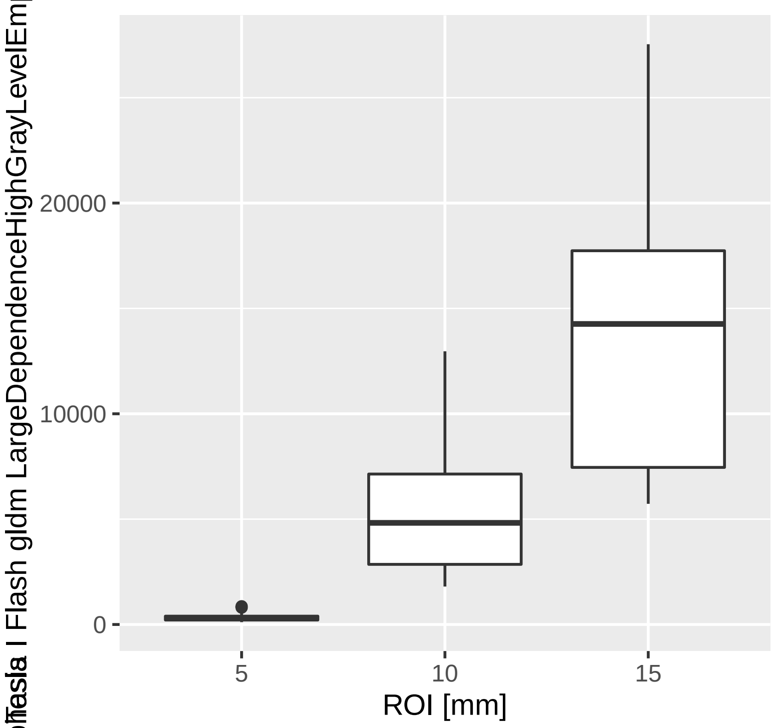
gldm LargeDependenceHighGrayLevelEmphasis
20000
10000
0
5
10
15
ROI [mm]

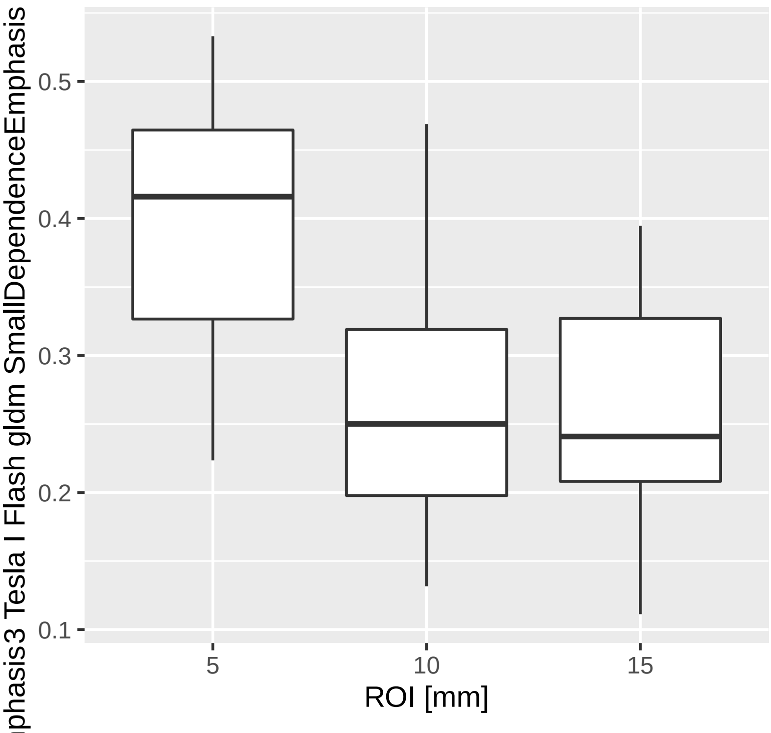
3 Tesla I Flash gldm SmallDependenceEmphasis
0.5
0.4
0.3
0.2
0.1
5
10
15
ROI [mm]

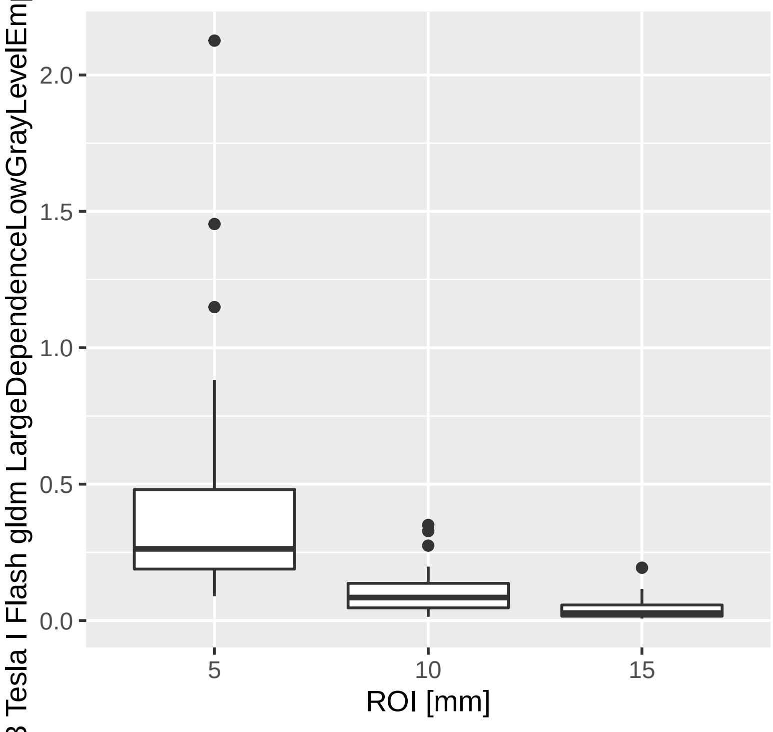
gldm LargeDependenceLowGrayLevelEmphasis
2.0
1.5
1.0
0.5
0.0
5
10
15
ROI [mm]

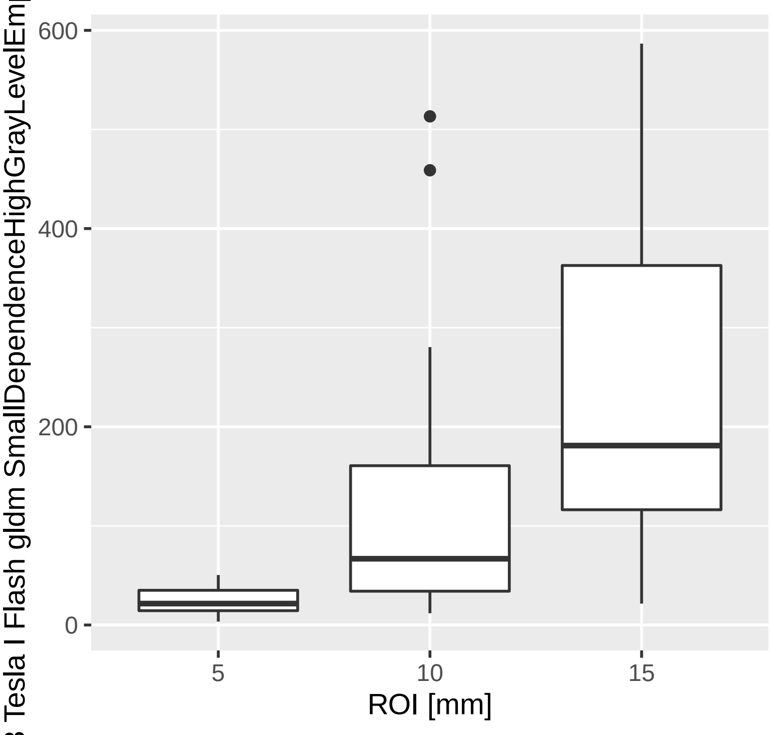
gldm SmallDependenceHighGrayLevelEmphasis
600
400
200
0
5
10
15
ROI [mm]

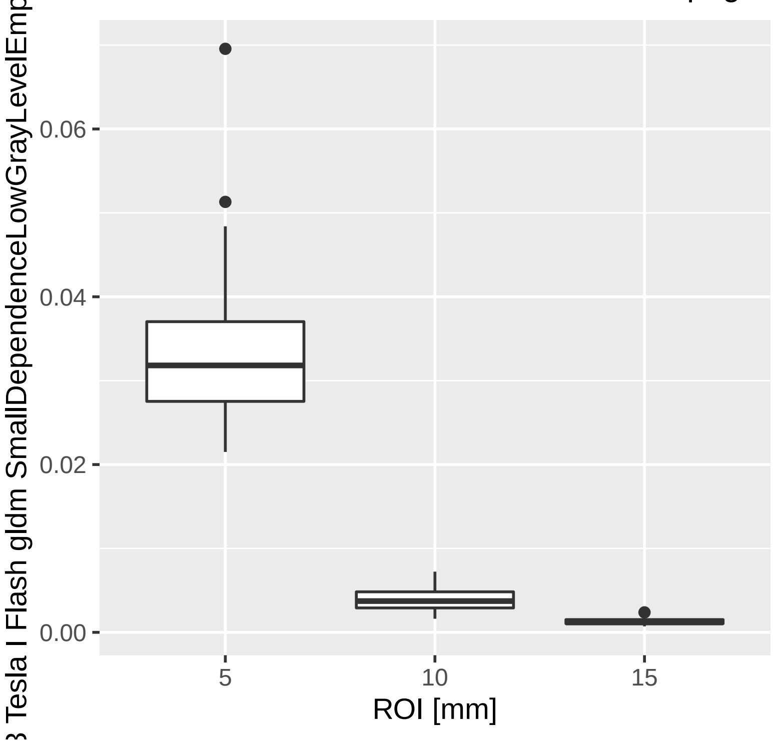
3 Tesla I Flash gldm SmallDependenceLowGrayLevelEmpha
0.00
0.02
0.04
0.06
5
10
15
ROI [mm]

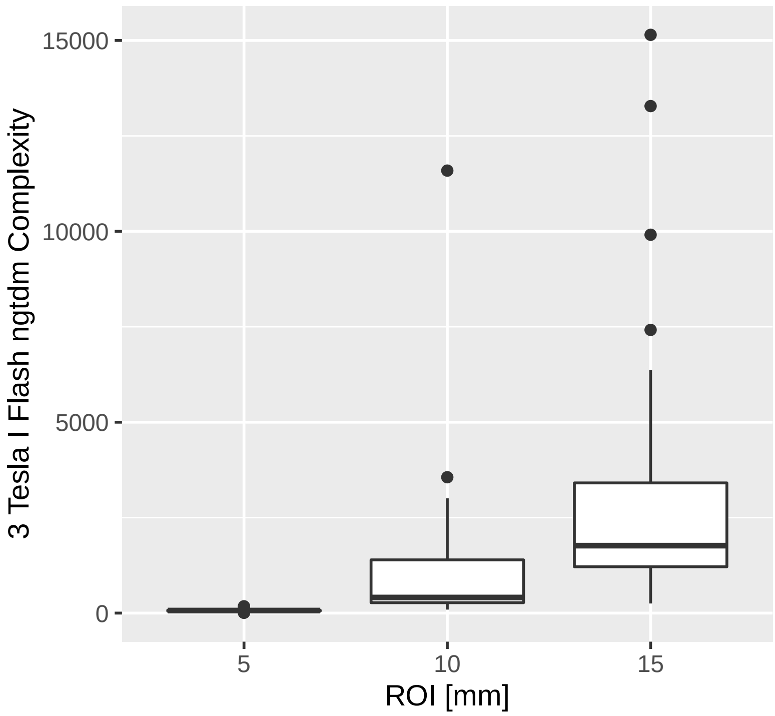
3 Tesla I Flash ngtdm Complexity
0
5000
10000
15000
5
10
15
ROI [mm]

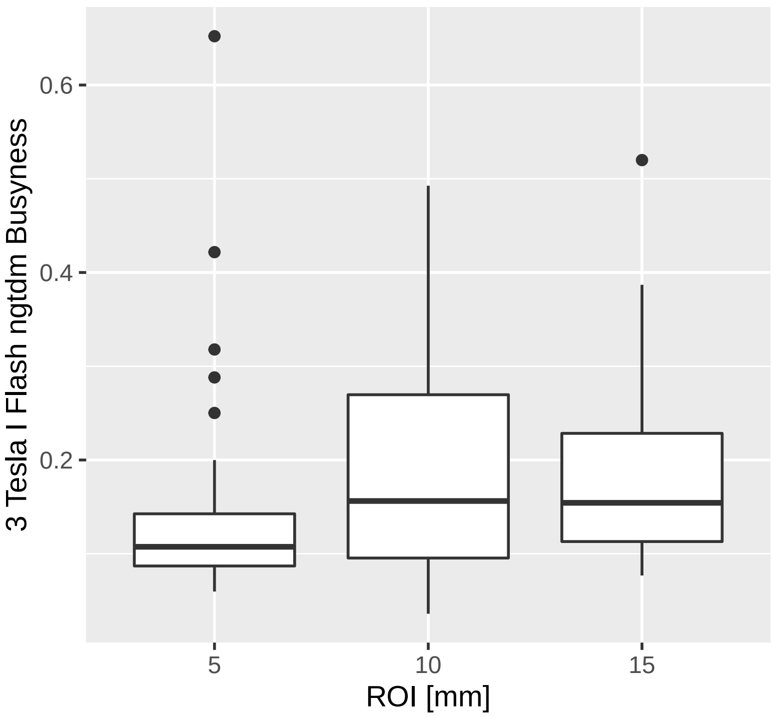
3 Tesla I Flash ngtdm Busyness
0.2
0.4
0.6
5
10
15
ROI [mm]

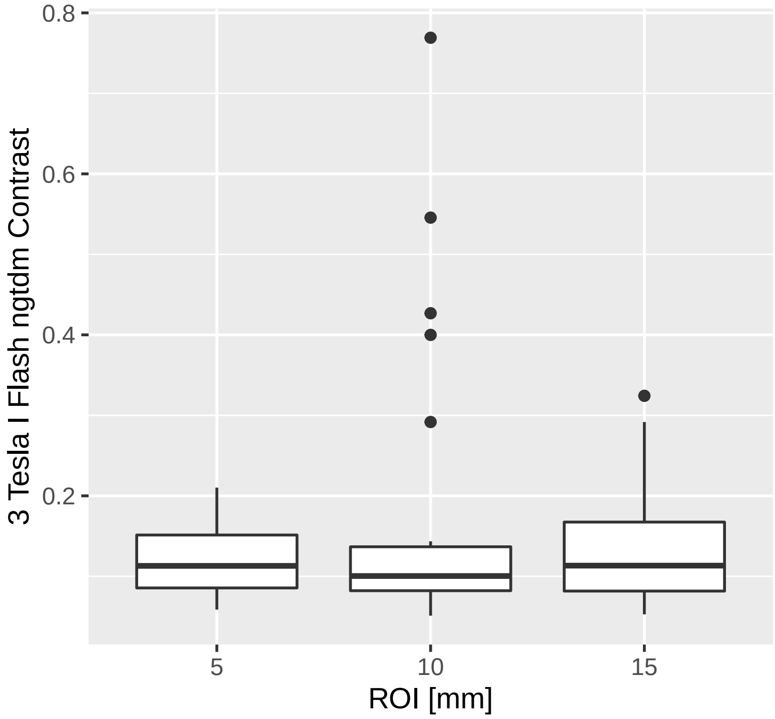
3 Tesla I Flash ngtdm Contrast
0.2
0.4
0.6
0.8
5
10
15
ROI [mm]

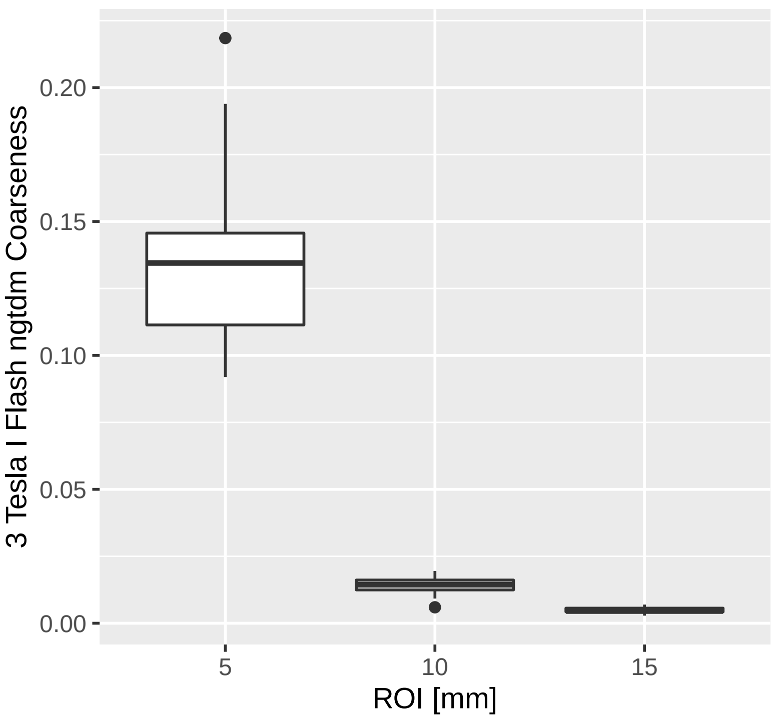
3 Tesla I Flash ngtdm Coarseness
0.00
0.05
0.10
0.15
0.20
5
10
15
ROI [mm]

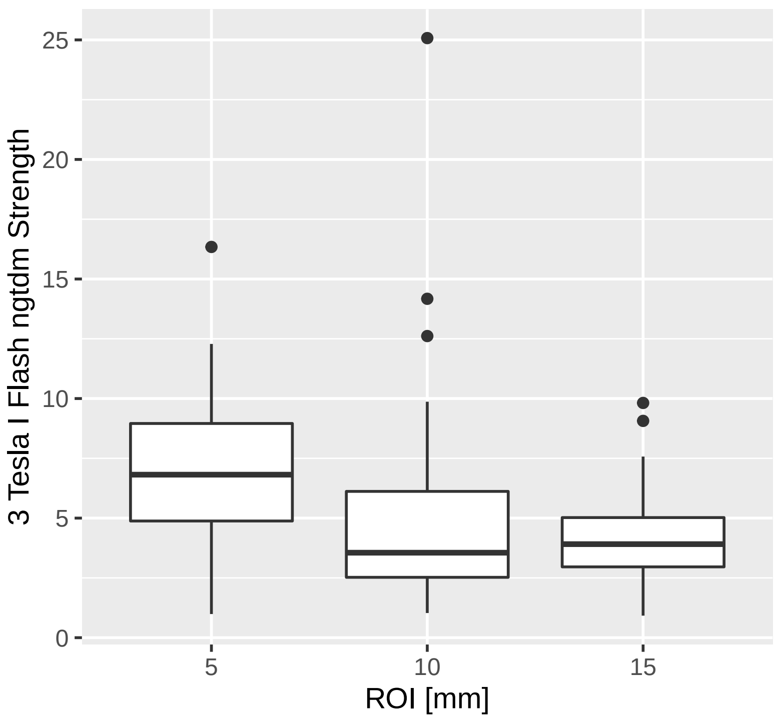
3 Tesla I Flash ngtdm Strength
0
5
10
15
20
25
5
10
15
ROI [mm]

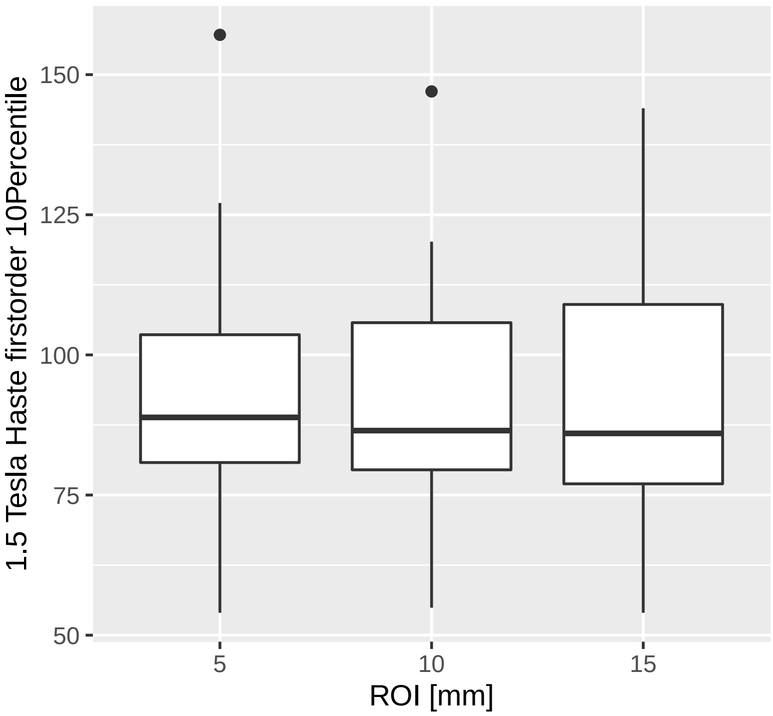
1.5 Tesla Haste firstorder 10Percentile
150
125
100
75
50
5
10
15
ROI [mm]

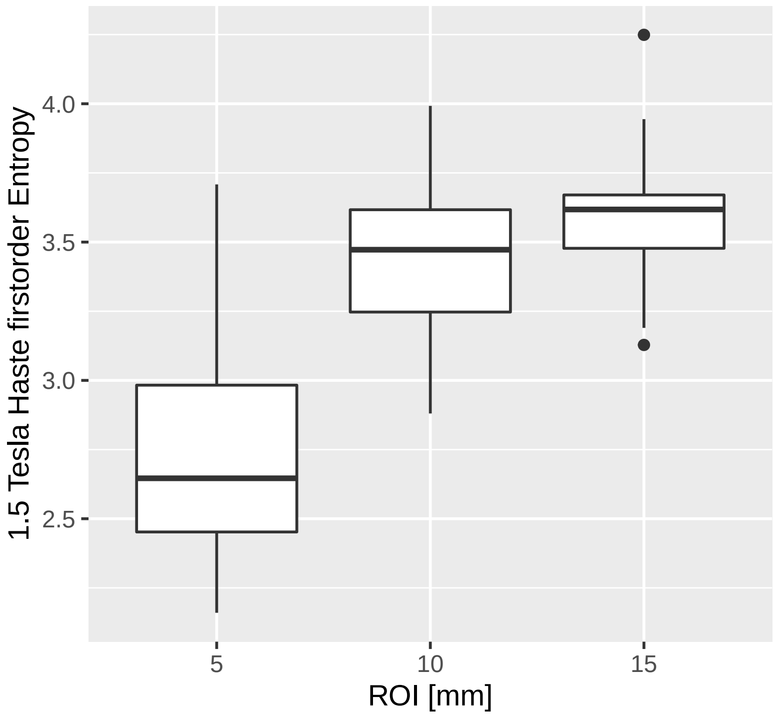
1.5 Tesla Haste firstorder Entropy
4.0
3.5
3.0
2.5
5
10
15
ROI [mm]

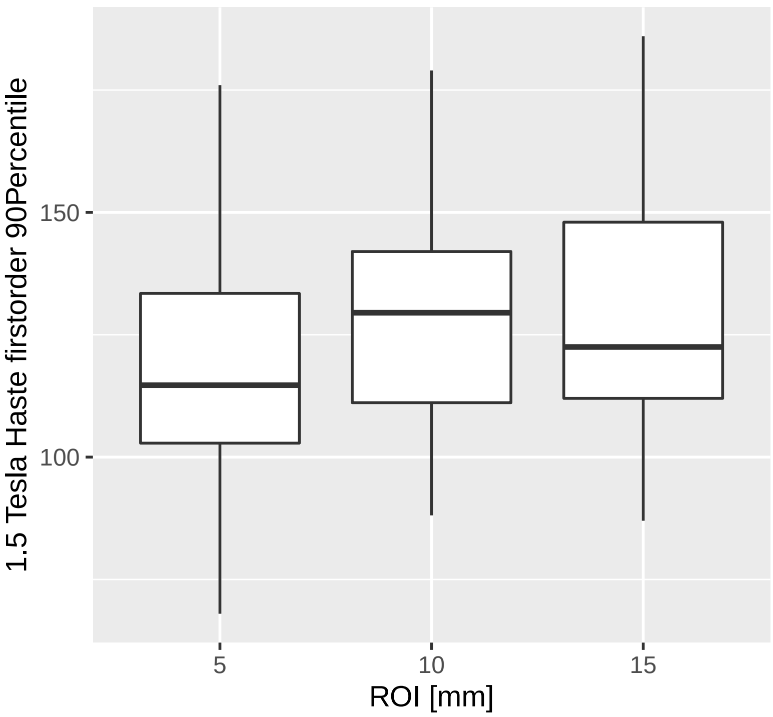
1.5 Tesla Haste firstorder 90Percentile
150
100
5
10
15
ROI [mm]

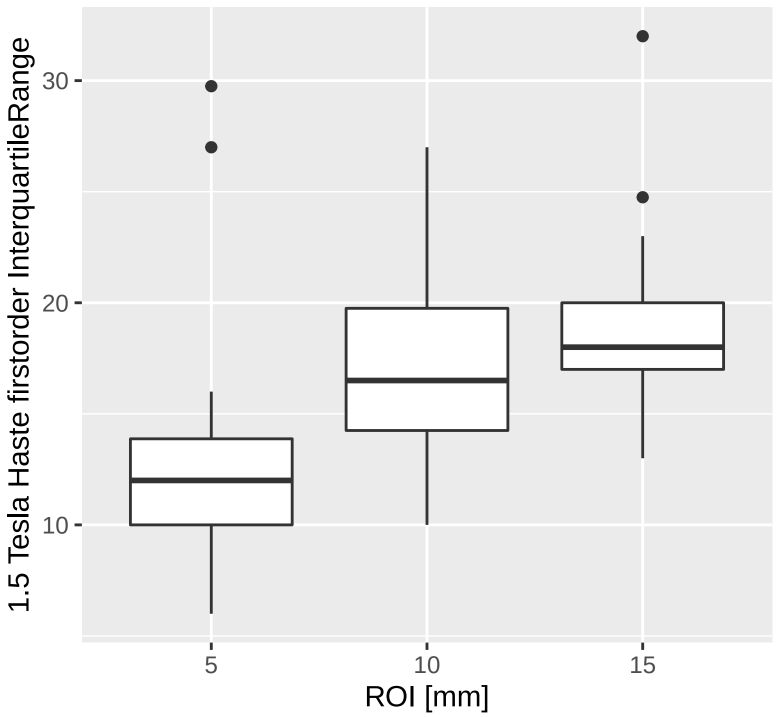
1.5 Tesla Haste firstorder InterquartileRange
30
20
10
5
10
15
ROI [mm]

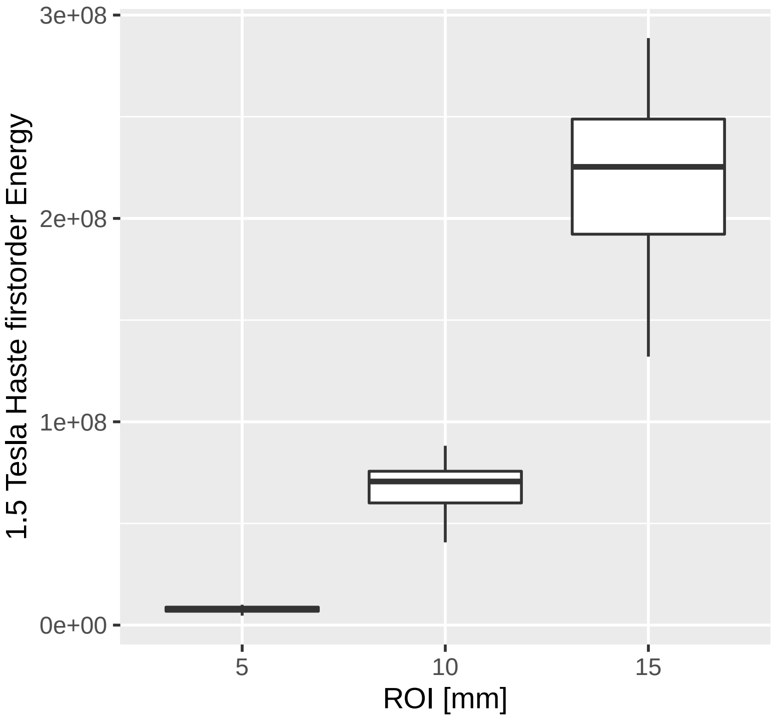
1.5 Tesla Haste firstorder Energy
3e+08
2e+08
1e+08
0e+00
5
10
15
ROI [mm]

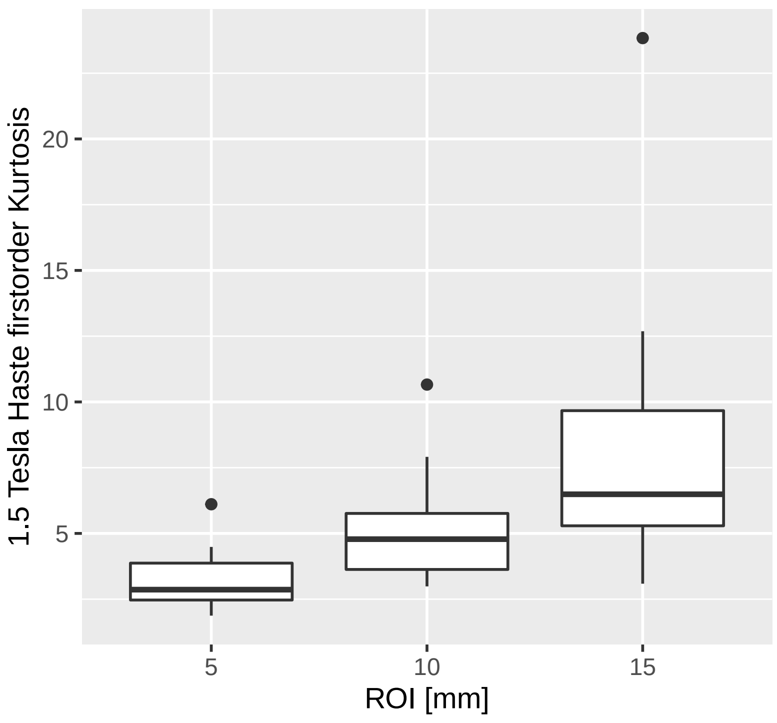
1.5 Tesla Haste firstorder Kurtosis
20
15
10
5
5
10
15
ROI [mm]

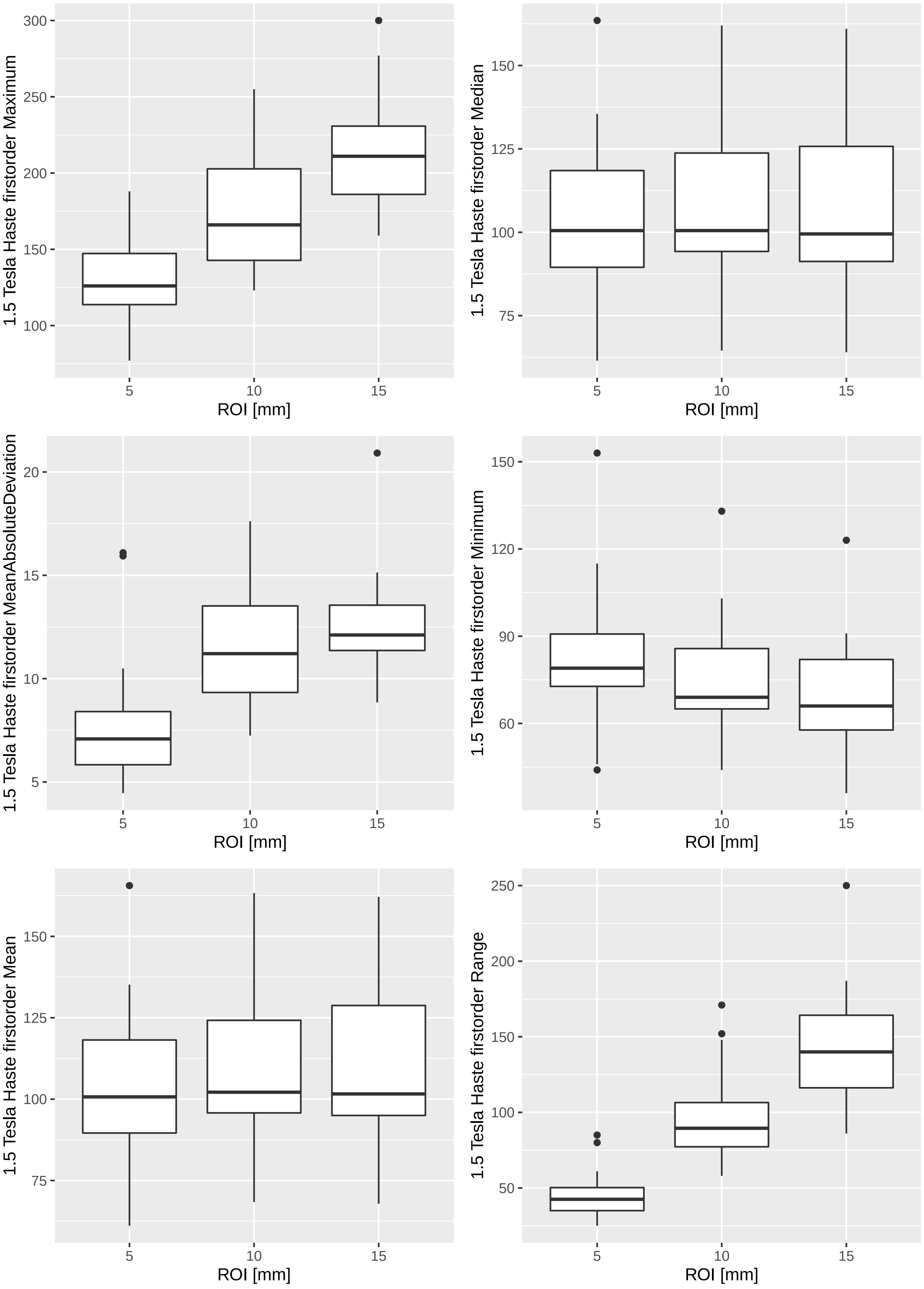

1.5 Tesla Haste firstorder Maximum
1.5 Tesla Haste firstorder Median
1.5 Tesla Haste firstorder MeanAbsoluteDeviation
1.5 Tesla Haste firstorder Minimum
1.5 Tesla Haste firstorder Mean
1.5 Tesla Haste firstorder Range
ROI [mm]
5
10
15

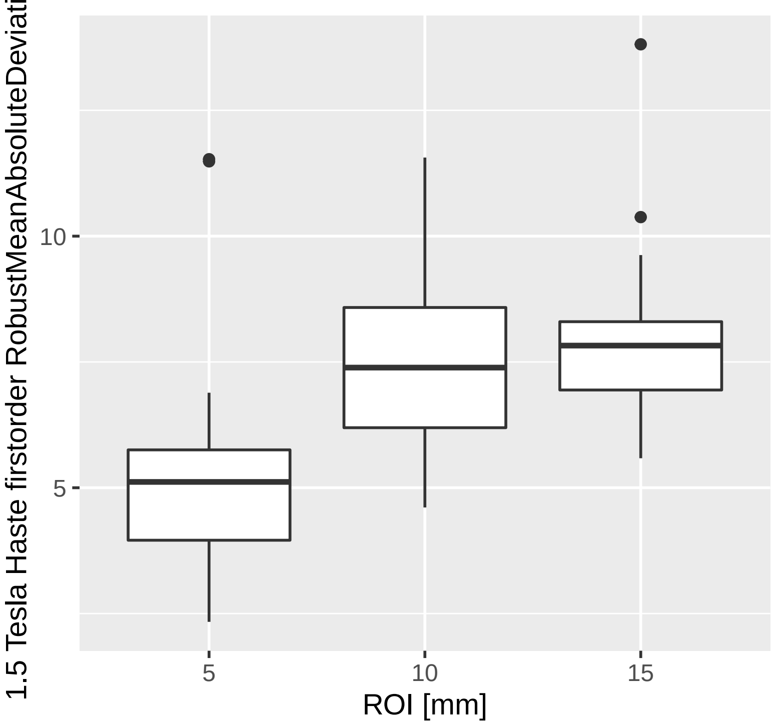
1.5 Tesla Haste firstorder RobustMeanAbsoluteDeviation
10
5
5
10
15
ROI [mm]

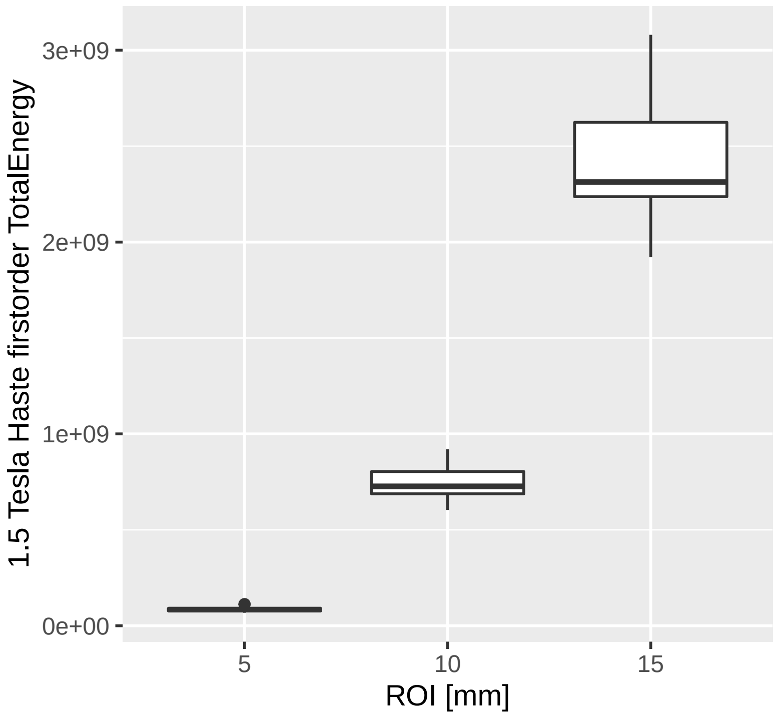
1.5 Tesla Haste firstorder TotalEnergy
3e+09
2e+09
1e+09
0e+00
5
10
15
ROI [mm]

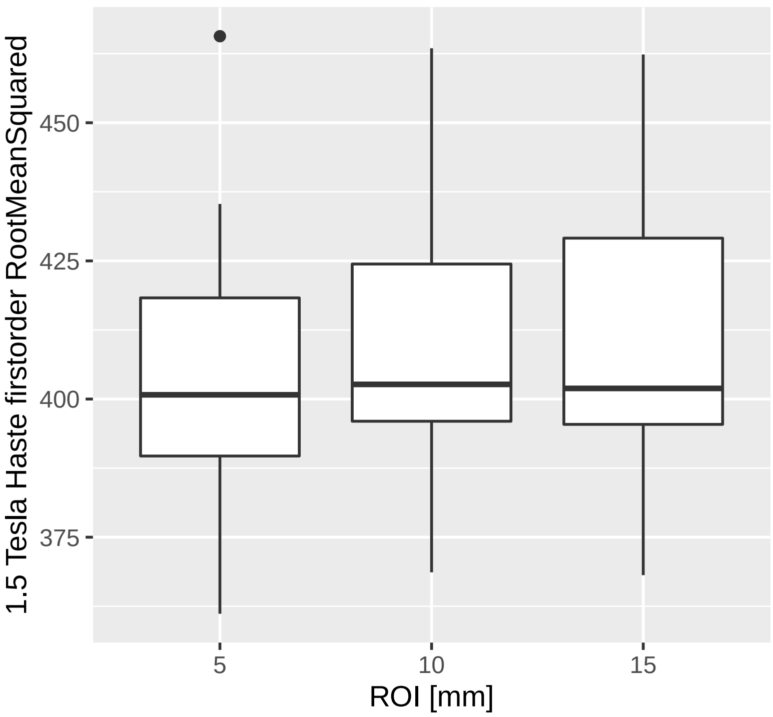
1.5 Tesla Haste firstorder RootMeanSquared
450
425
400
375
5
10
15
ROI [mm]

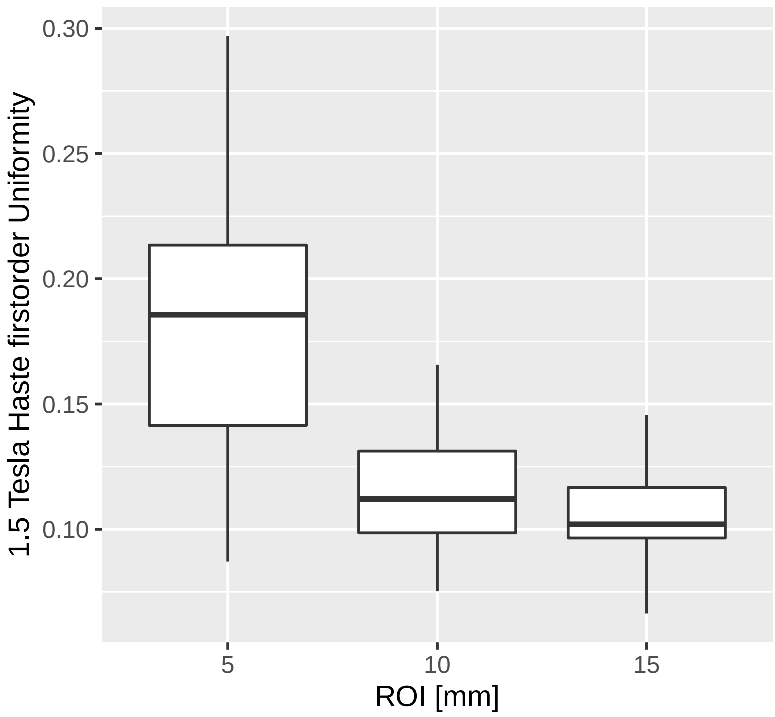
1.5 Tesla Haste firstorder Uniformity
0.30
0.25
0.20
0.15
0.10
5
10
15
ROI [mm]

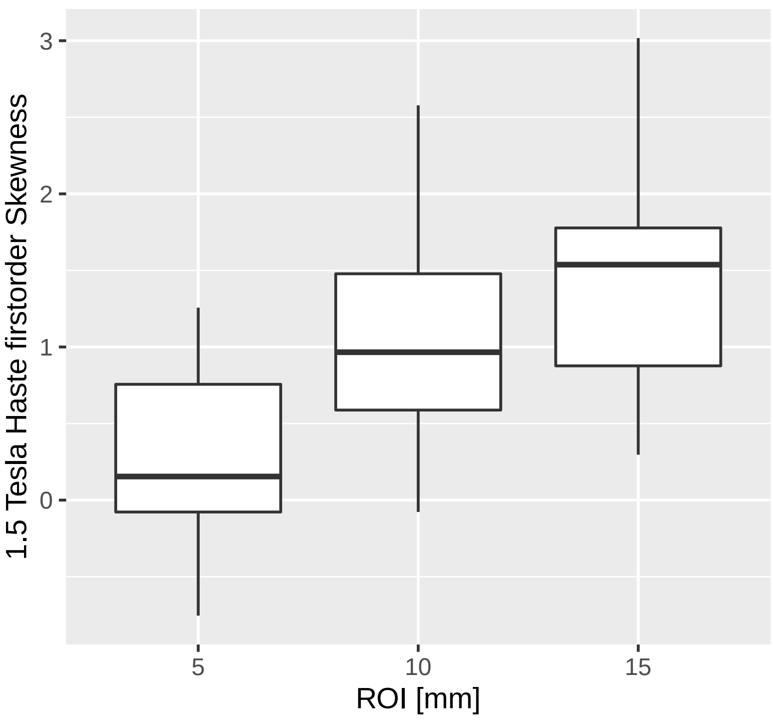
1.5 Tesla Haste firstorder Skewness
3
2
1
0
5
10
15
ROI [mm]

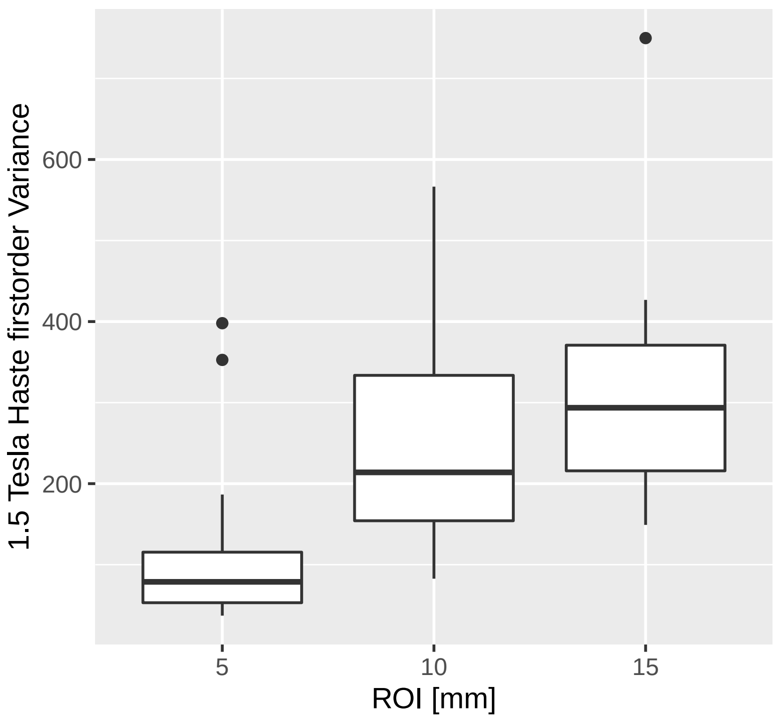
1.5 Tesla Haste firstorder Variance
600
400
200
5
10
15
ROI [mm]

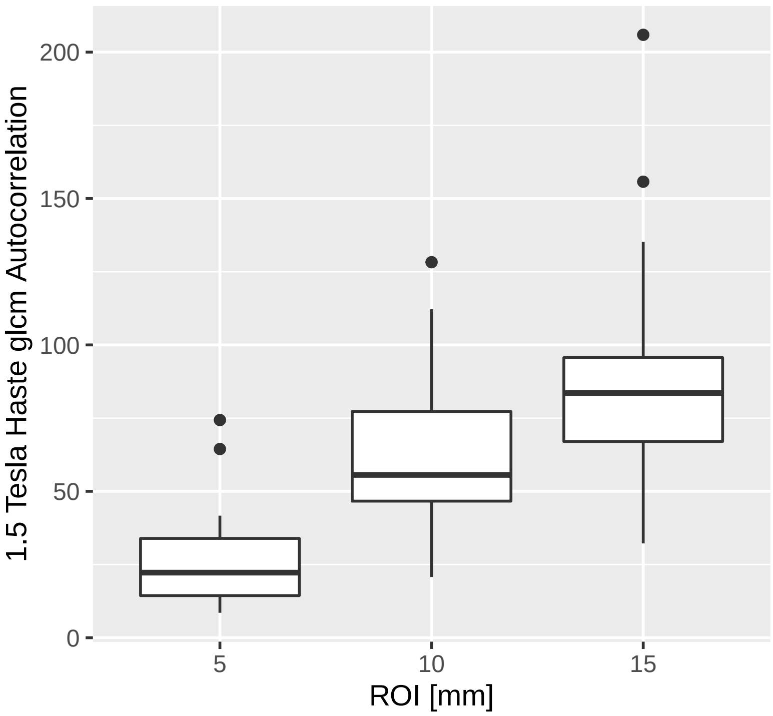

1.5 Tesla Haste glcm Autocorrelation
200
150
100
50
0
5
10
15
ROI [mm]

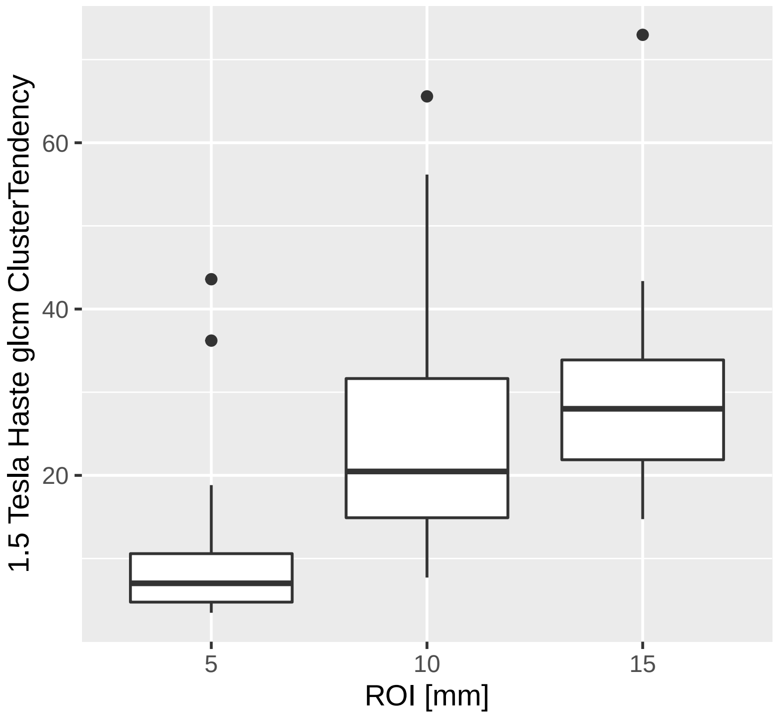

1.5 Tesla Haste glcm ClusterTendency
60
40
20
5
10
15
ROI [mm]

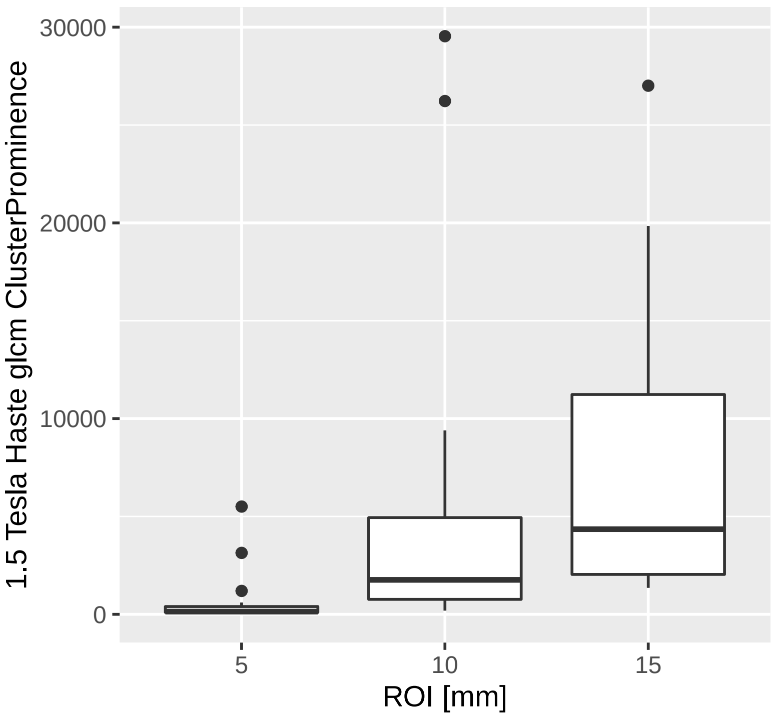

1.5 Tesla Haste glcm ClusterProminence
30000
20000
10000
0
5
10
15
ROI [mm]

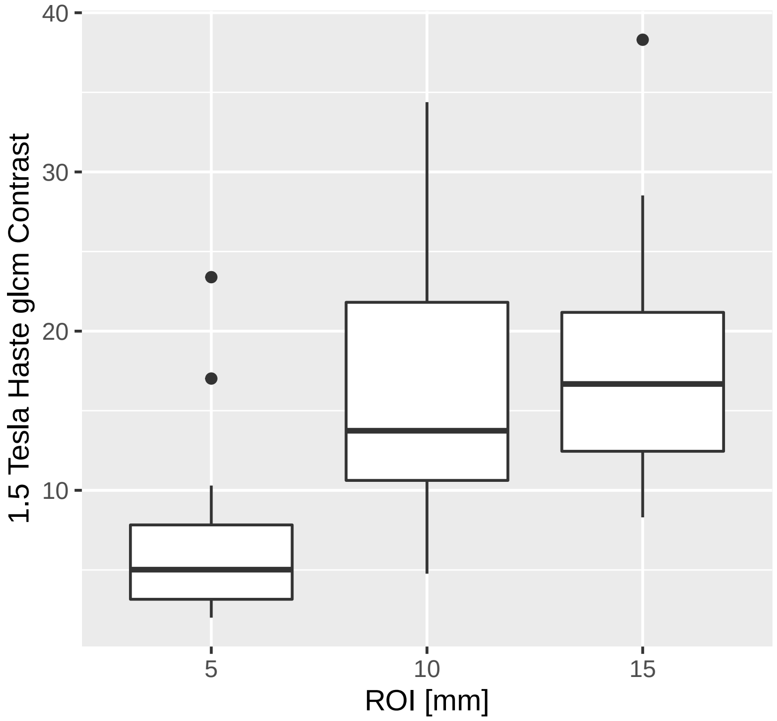

1.5 Tesla Haste glcm Contrast
40
30
20
10
5
10
15
ROI [mm]

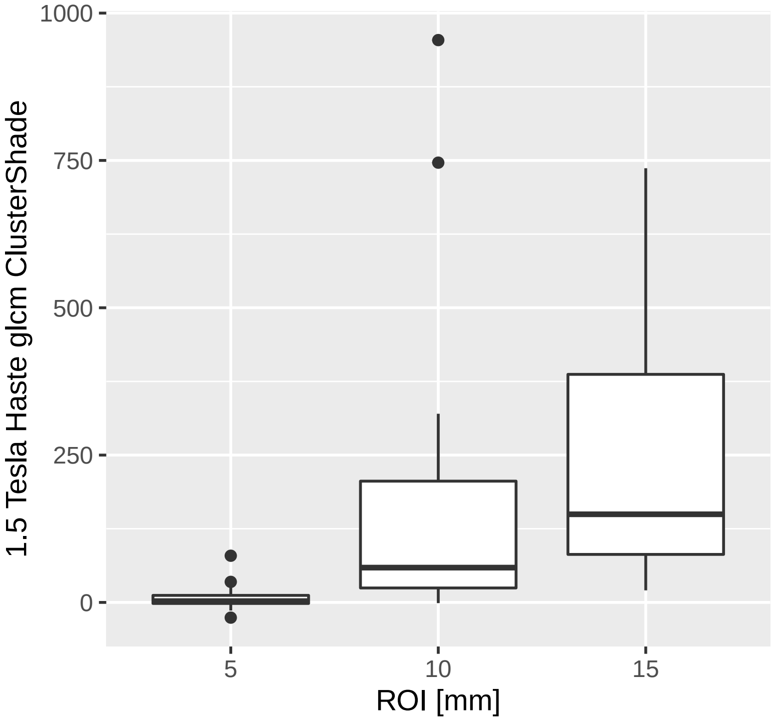

1.5 Tesla Haste glcm ClusterShade
1000
750
500
250
0
5
10
15
ROI [mm]

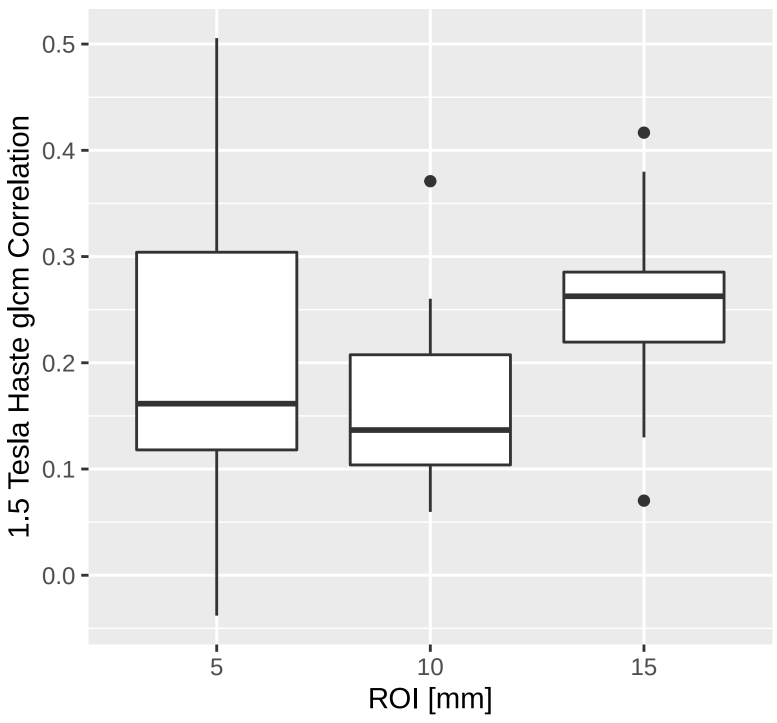

1.5 Tesla Haste glcm Correlation
0.5
0.4
0.3
0.2
0.1
0.0
5
10
15
ROI [mm]

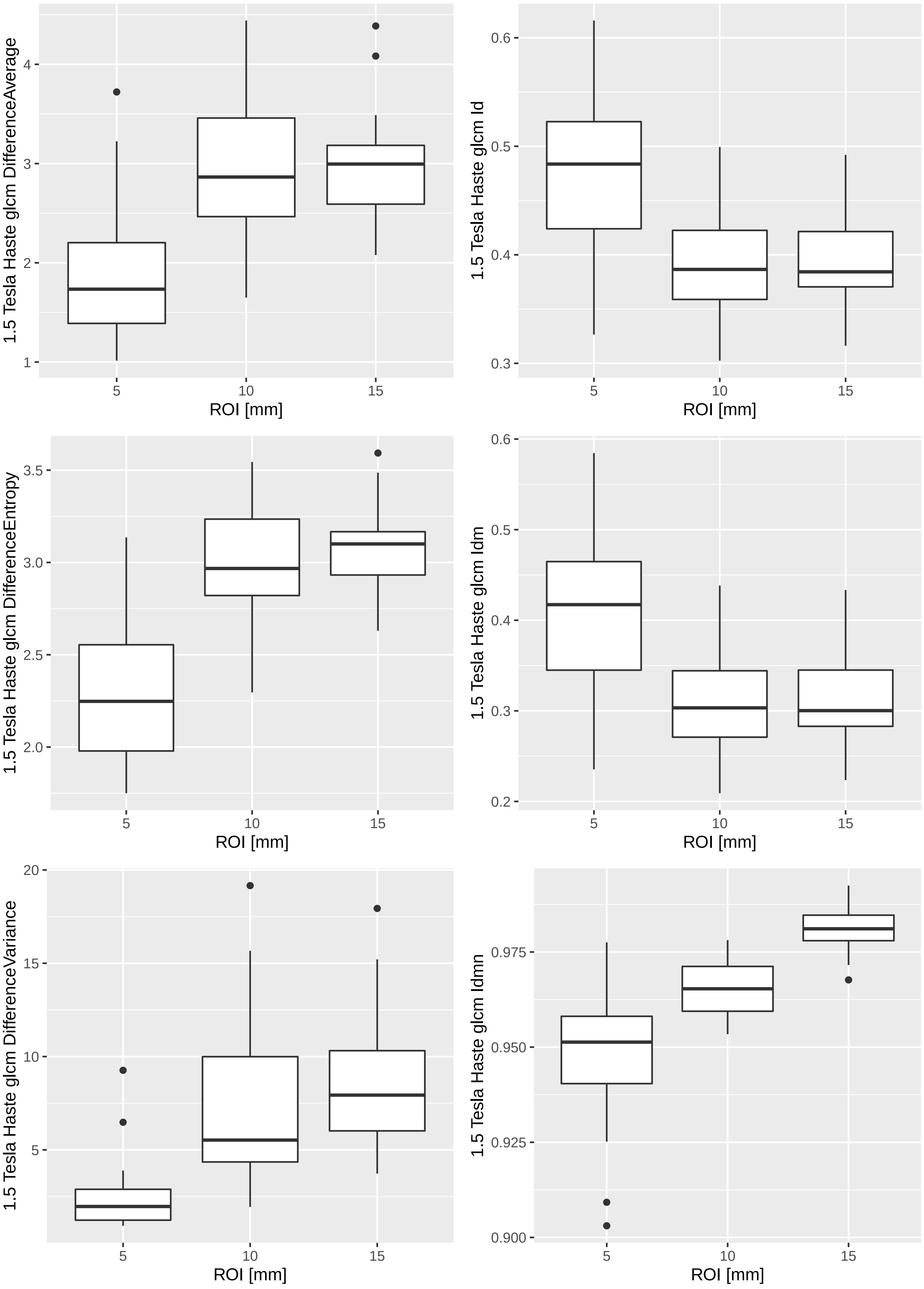
1.5 Tesla Haste glcm DifferenceAverage
1 2 3 4
ROI [mm]
5 10 15
1.5 Tesla Haste glcm Id
0.3 0.4 0.5 0.6
ROI [mm]
5 10 15
1.5 Tesla Haste glcm DifferenceEntropy
2.0 2.5 3.0 3.5
ROI [mm]
5 10 15
1.5 Tesla Haste glcm Idm
0.2 0.3 0.4 0.5 0.6
ROI [mm]
5 10 15
1.5 Tesla Haste glcm DifferenceVariance
5 10 15 20
ROI [mm]
5 10 15
1.5 Tesla Haste glcm Idmn
0.900 0.925 0.950 0.975
ROI [mm]
5 10 15

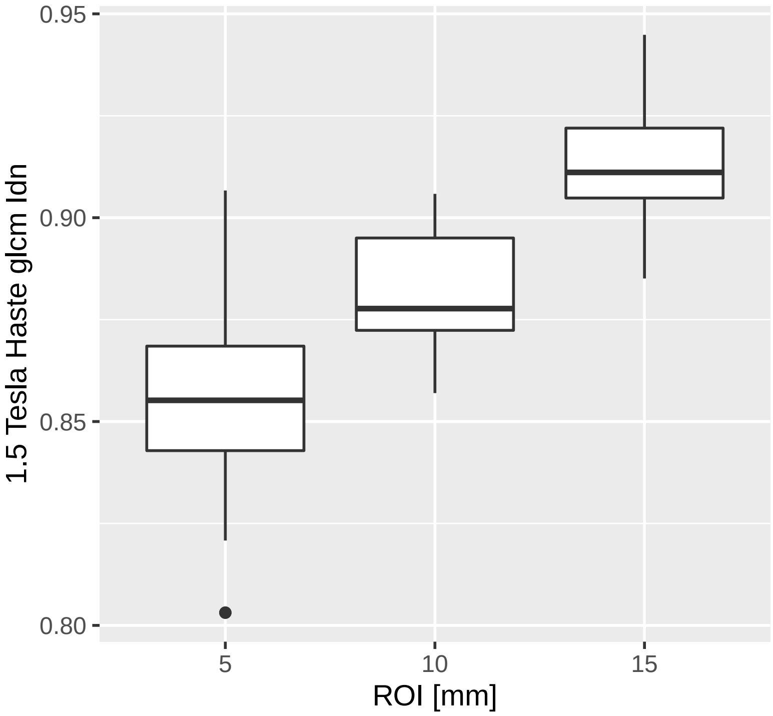
1.5 Tesla Haste glcm Idn
0.95
0.90
0.85
0.80
5
10
15
ROI [mm]

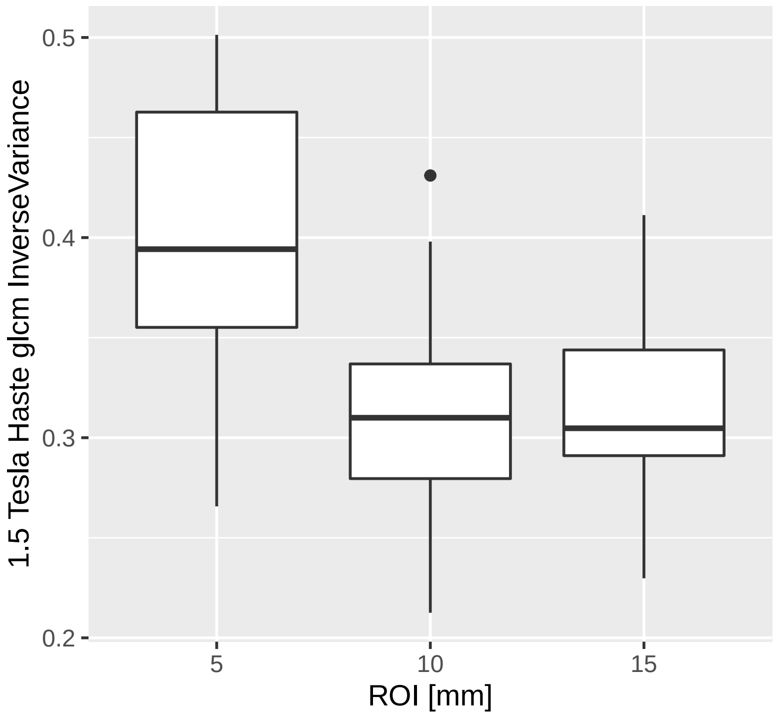
1.5 Tesla Haste glcm InverseVariance
0.5
0.4
0.3
0.2
5
10
15
ROI [mm]

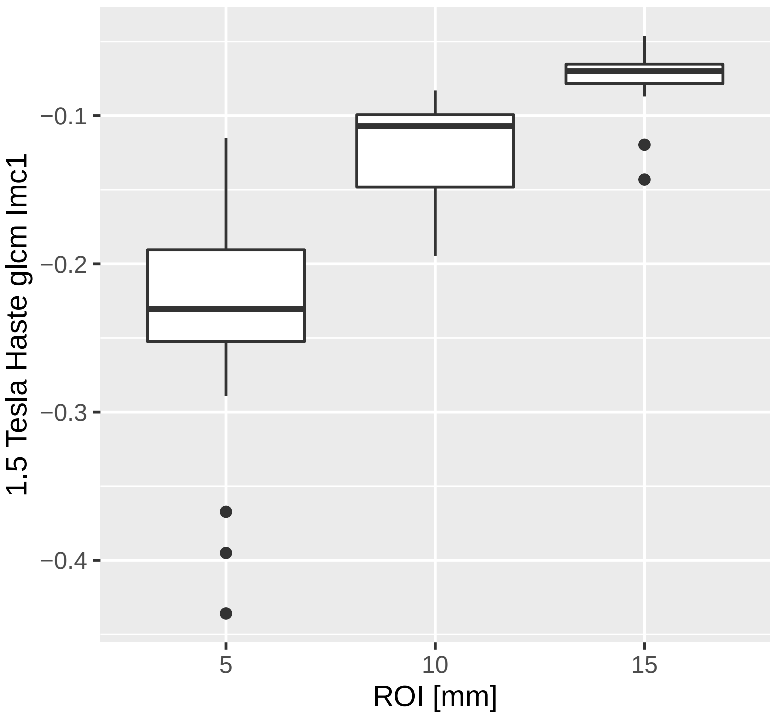
1.5 Tesla Haste glcm Imc1
−0.1
−0.2
−0.3
−0.4
5
10
15
ROI [mm]

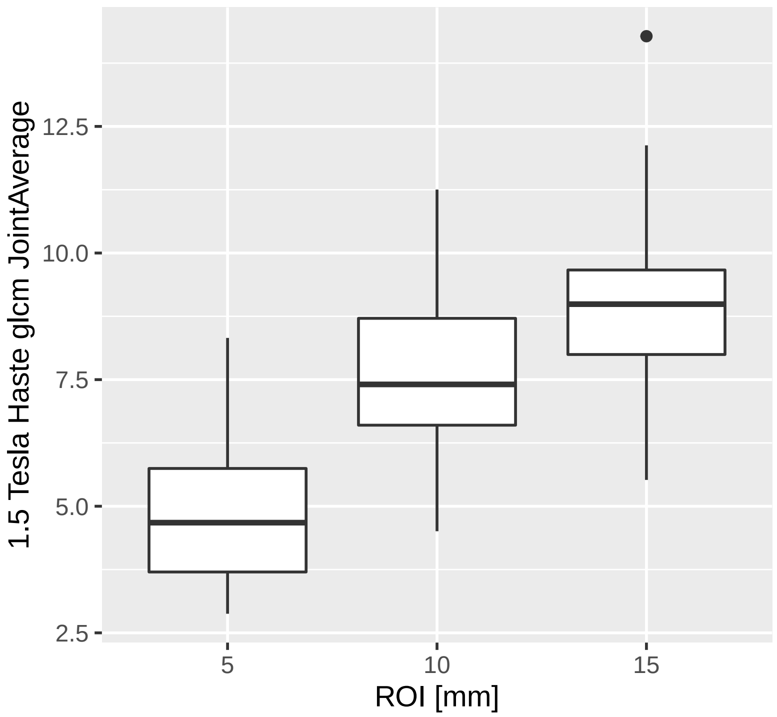
1.5 Tesla Haste glcm JointAverage
12.5
10.0
7.5
5.0
2.5
5
10
15
ROI [mm]

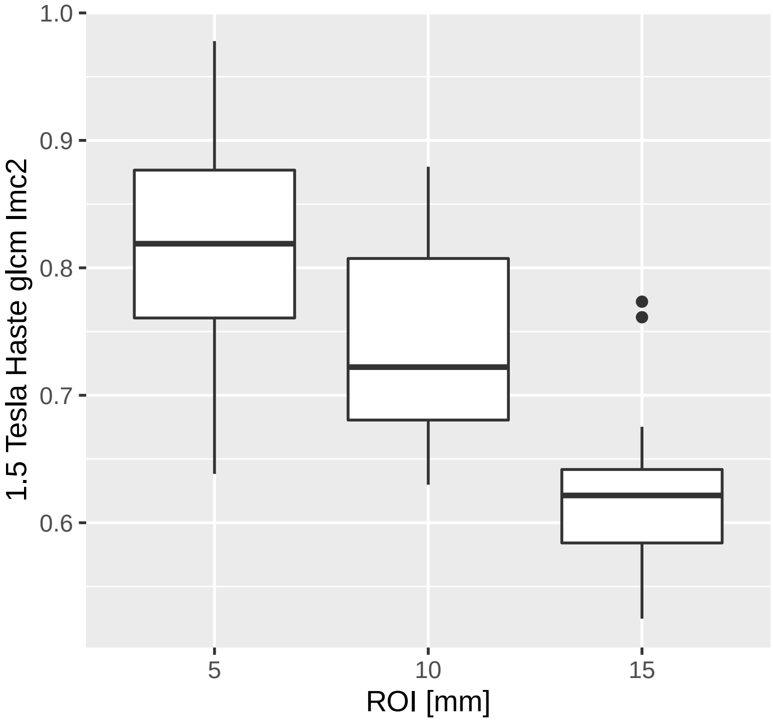
1.5 Tesla Haste glcm Imc2
1.0
0.9
0.8
0.7
0.6
5
10
15
ROI [mm]

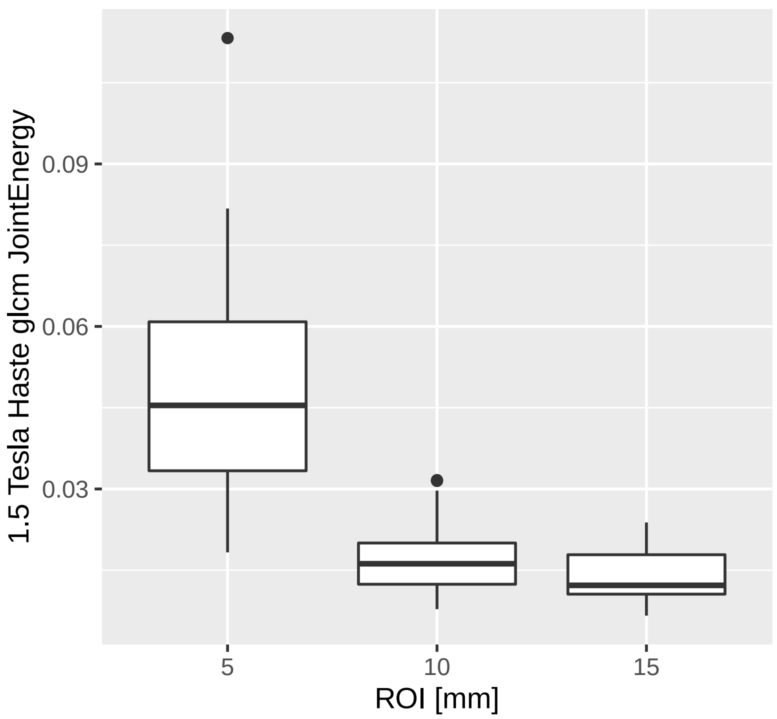
1.5 Tesla Haste glcm JointEnergy
0.09
0.06
0.03
5
10
15
ROI [mm]

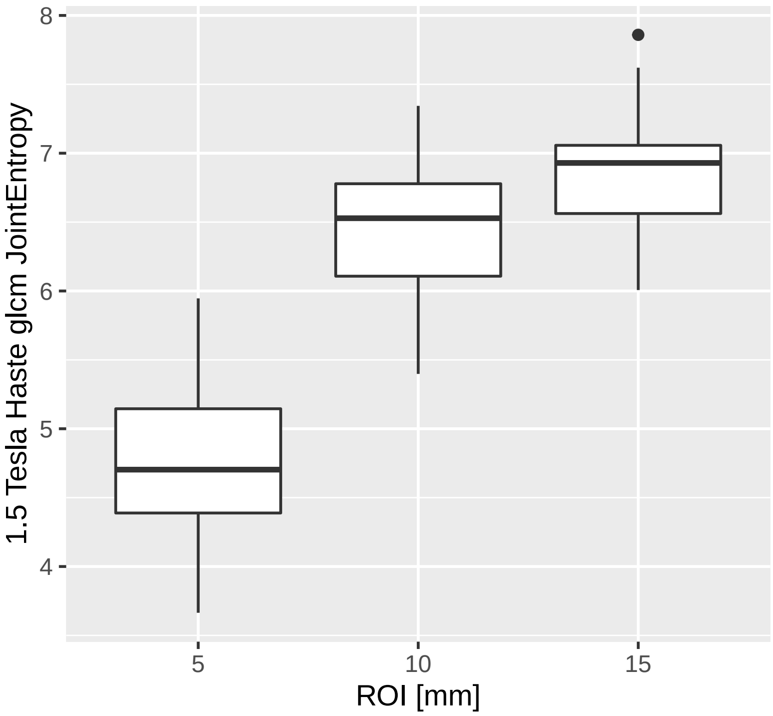
1.5 Tesla Haste glcm JointEntropy
8
7
6
5
4
5
10
15
ROI [mm]

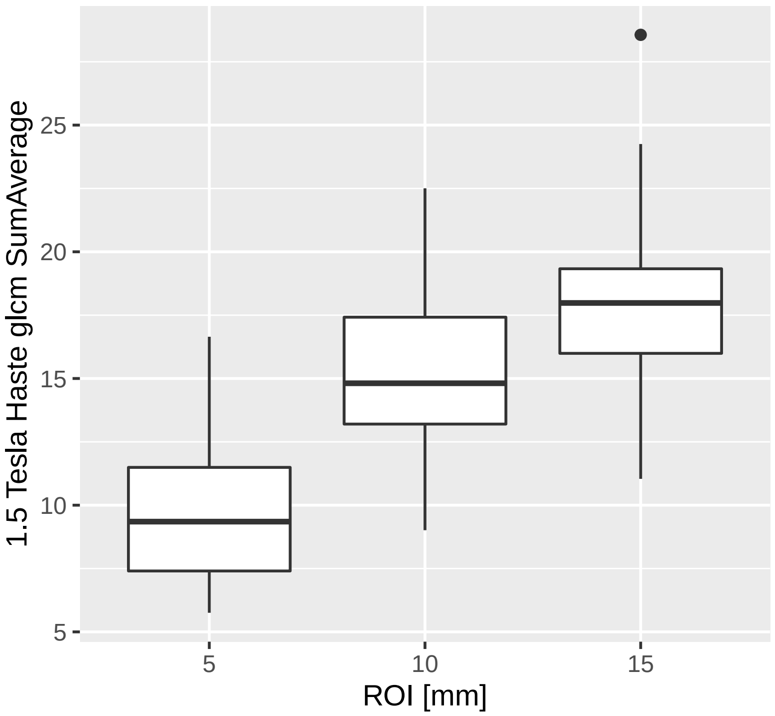
1.5 Tesla Haste glcm SumAverage
25
20
15
10
5
5
10
15
ROI [mm]

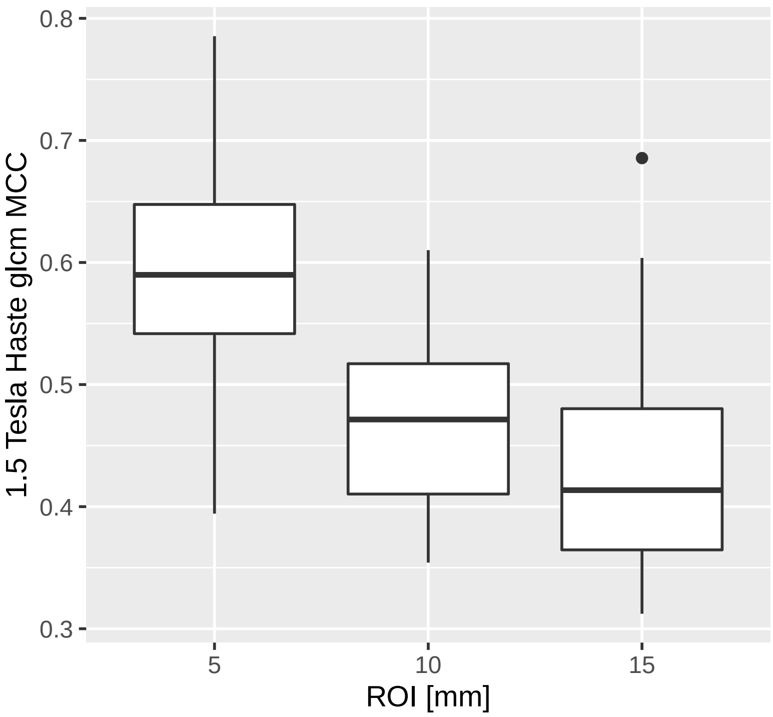
1.5 Tesla Haste glcm MCC
0.8
0.7
0.6
0.5
0.4
0.3
5
10
15
ROI [mm]

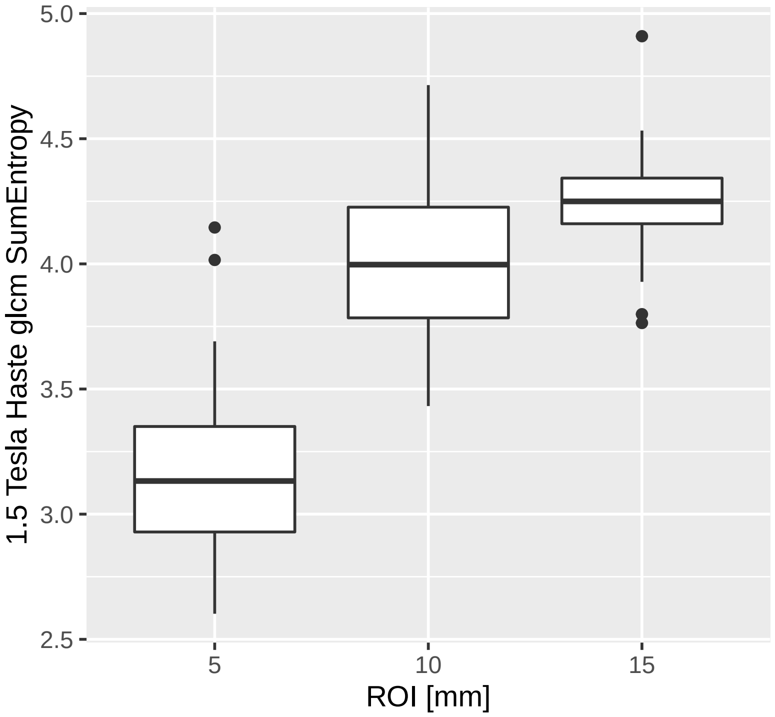
1.5 Tesla Haste glcm SumEntropy
5.0
4.5
4.0
3.5
3.0
2.5
5
10
15
ROI [mm]

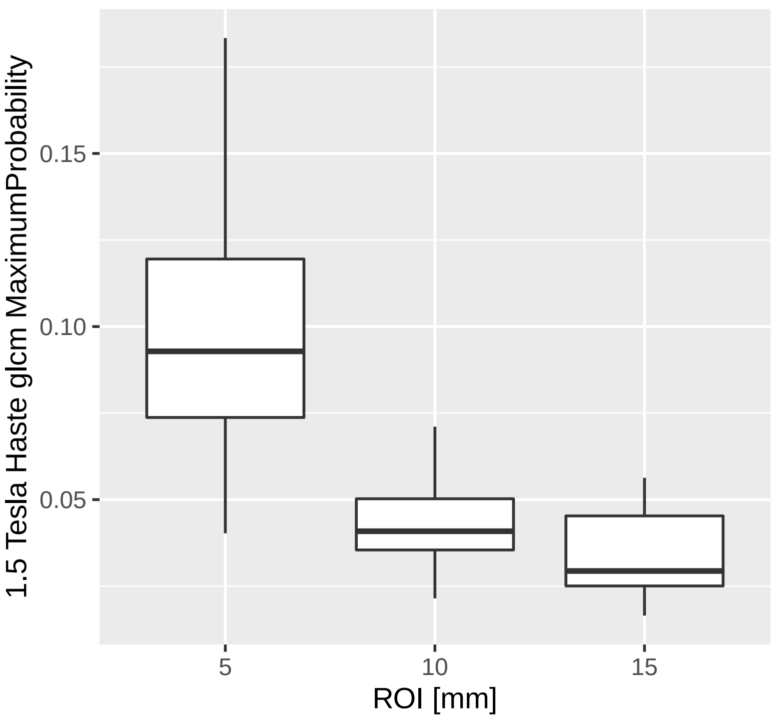
1.5 Tesla Haste glcm MaximumProbability
0.15
0.10
0.05
5
10
15
ROI [mm]

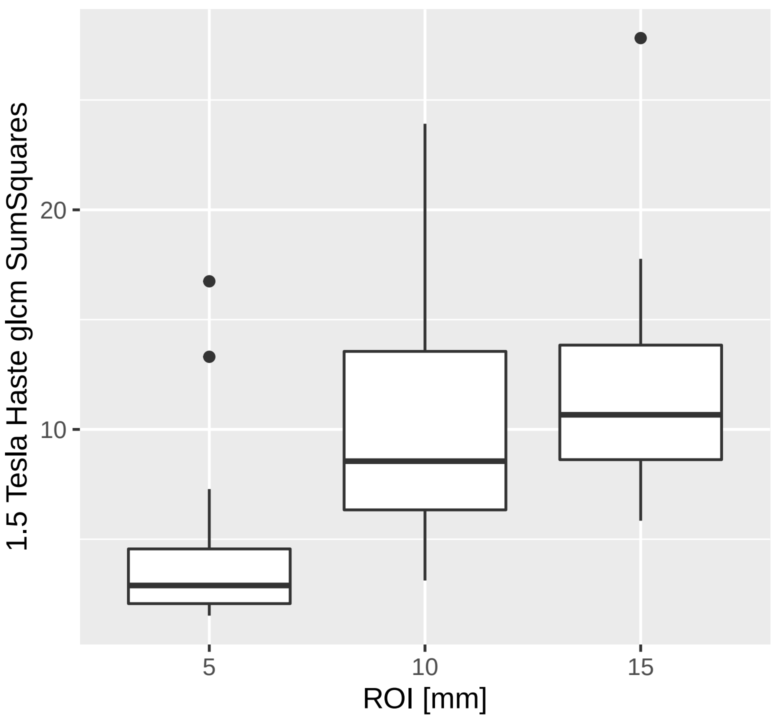
1.5 Tesla Haste glcm SumSquares
20
10
5
10
15
ROI [mm]

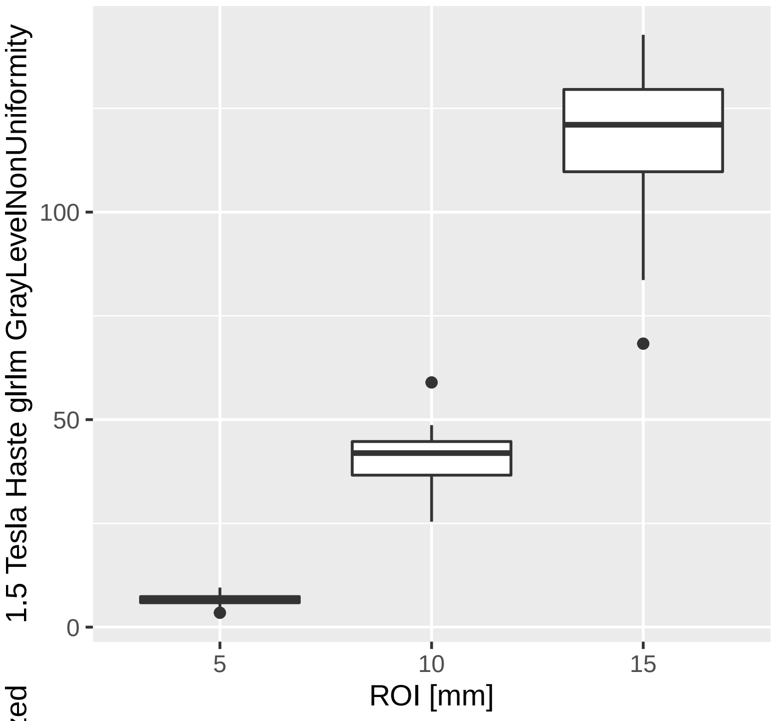
1.5 Tesla Haste glrlm GrayLevelNonUniformity
0
50
100
5
10
15
ROI [mm]

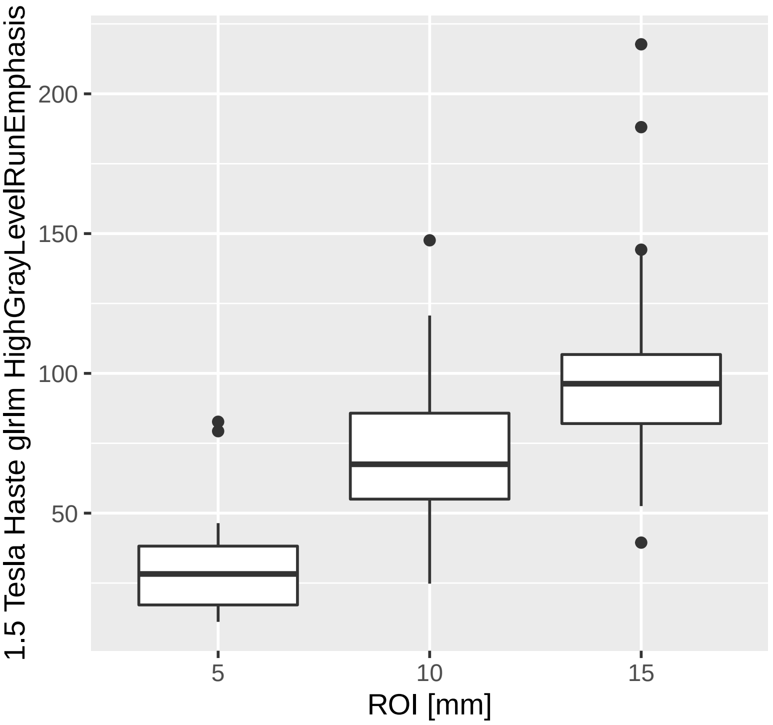
1.5 Tesla Haste glrlm HighGrayLevelRunEmphasis
50
100
150
200
5
10
15
ROI [mm]

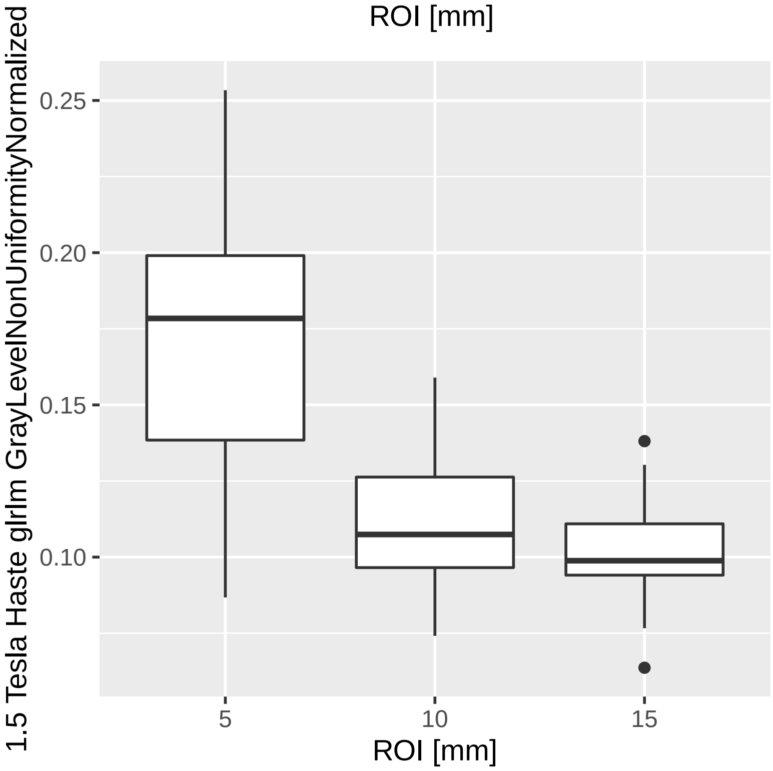
1.5 Tesla Haste glrlm GrayLevelNonUniformityNormalized
0.10
0.15
0.20
0.25
5
10
15
ROI [mm]

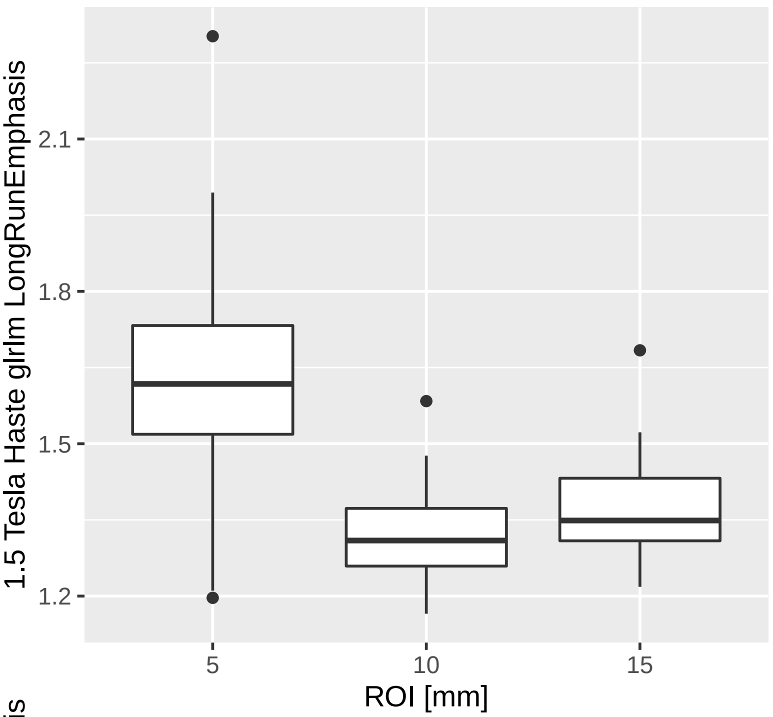
1.5 Tesla Haste glrlm LongRunEmphasis
1.2
1.5
1.8
2.1
5
10
15
ROI [mm]

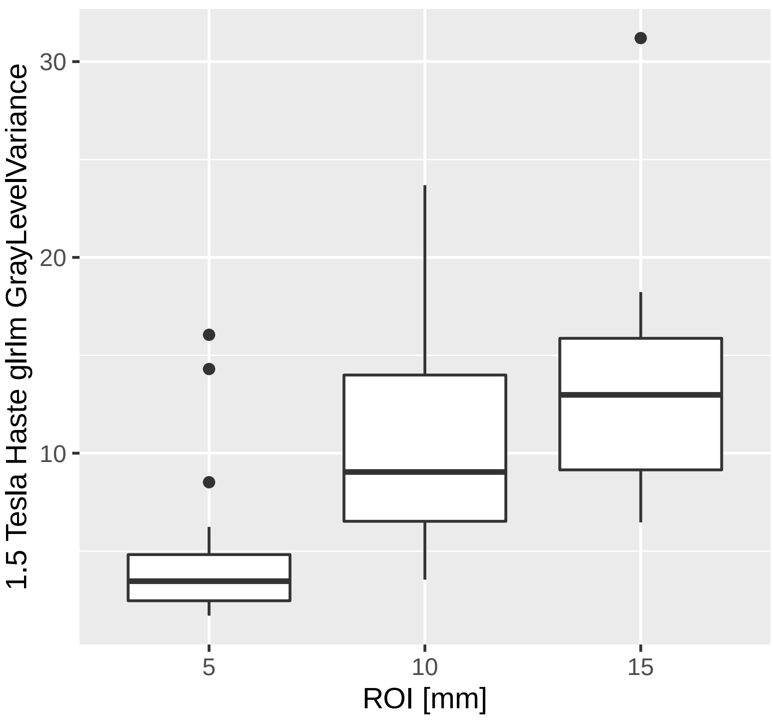
1.5 Tesla Haste glrlm GrayLevelVariance
10
20
30
5
10
15
ROI [mm]

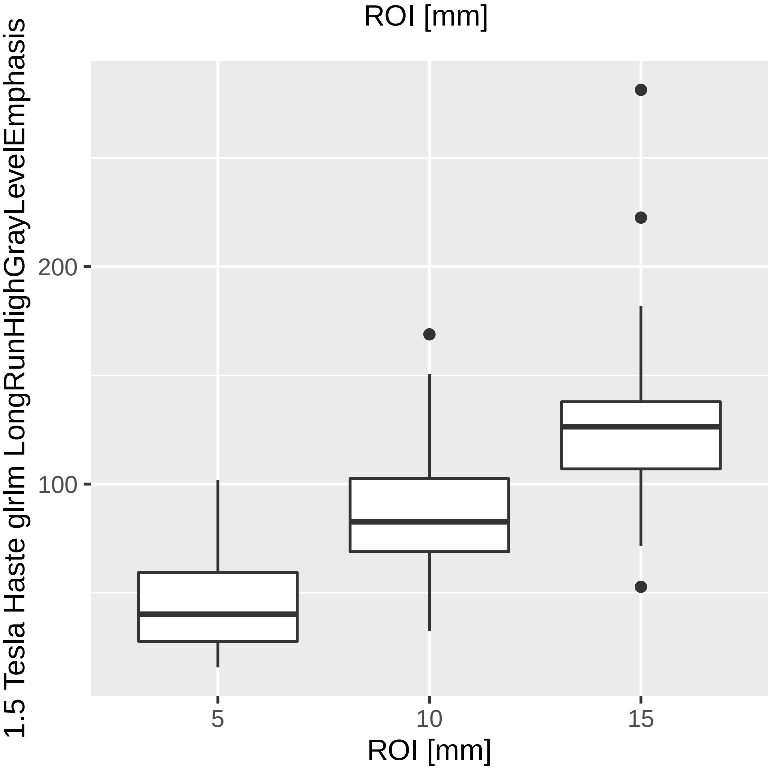
1.5 Tesla Haste glrlm LongRunHighGrayLevelEmphasis
100
200
5
10
15
ROI [mm]

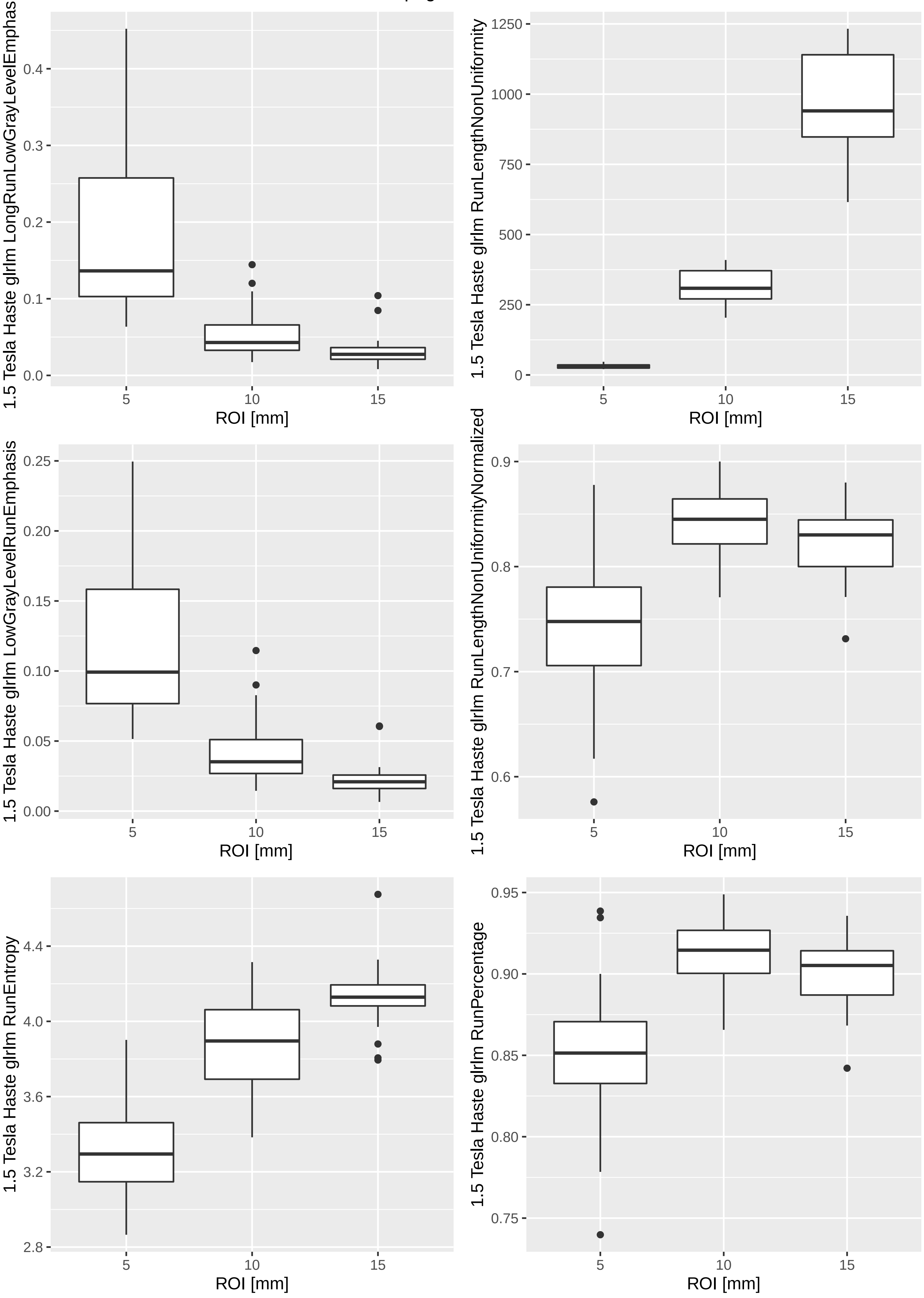
1.5 Tesla Haste glrlm LongRunLowGrayLevelEmphasis
0.0
0.1
0.2
0.3
0.4
5
10
15
ROI [mm]
1.5 Tesla Haste glrlm RunLengthNonUniformity
0
250
500
750
1000
1250
5
10
15
ROI [mm]
1.5 Tesla Haste glrlm LowGrayLevelRunEmphasis
0.00
0.05
0.10
0.15
0.20
0.25
5
10
15
ROI [mm]
1.5 Tesla Haste glrlm RunLengthNonUniformityNormalized
0.6
0.7
0.8
0.9
5
10
15
ROI [mm]
1.5 Tesla Haste glrlm RunEntropy
2.8
3.2
3.6
4.0
4.4
5
10
15
ROI [mm]
1.5 Tesla Haste glrlm RunPercentage
0.75
0.80
0.85
0.90
0.95
5
10
15
ROI [mm]

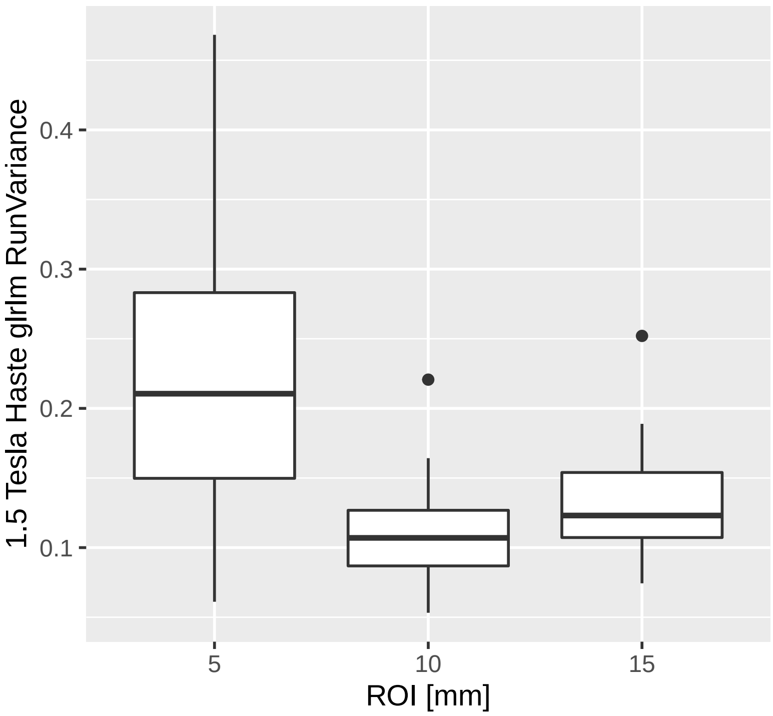
1.5 Tesla Haste glrlm RunVariance
0.1
0.2
0.3
0.4
5
10
15
ROI [mm]

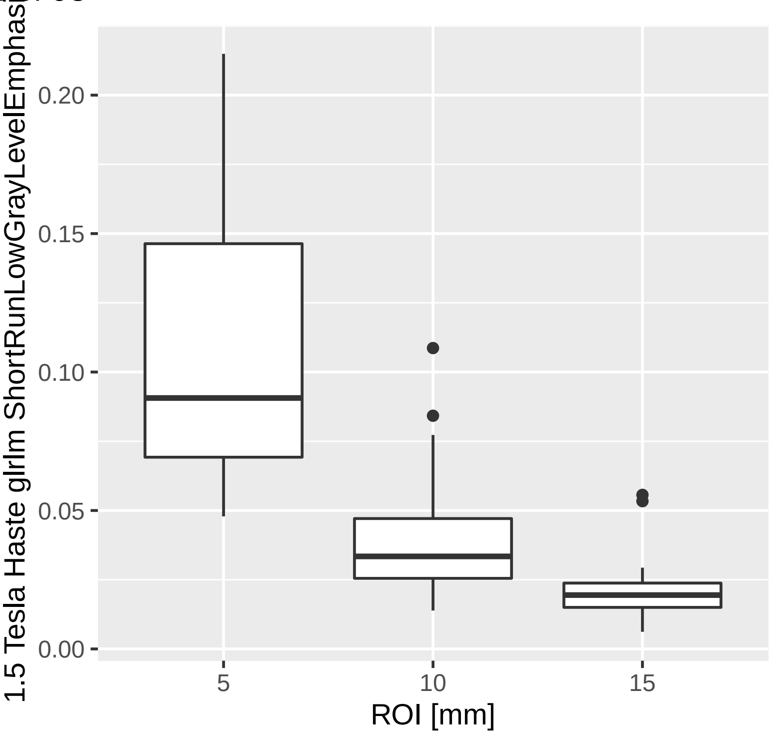
1.5 Tesla Haste glrlm ShortRunLowGrayLevelEmphasis
0.00
0.05
0.10
0.15
0.20
5
10
15
ROI [mm]

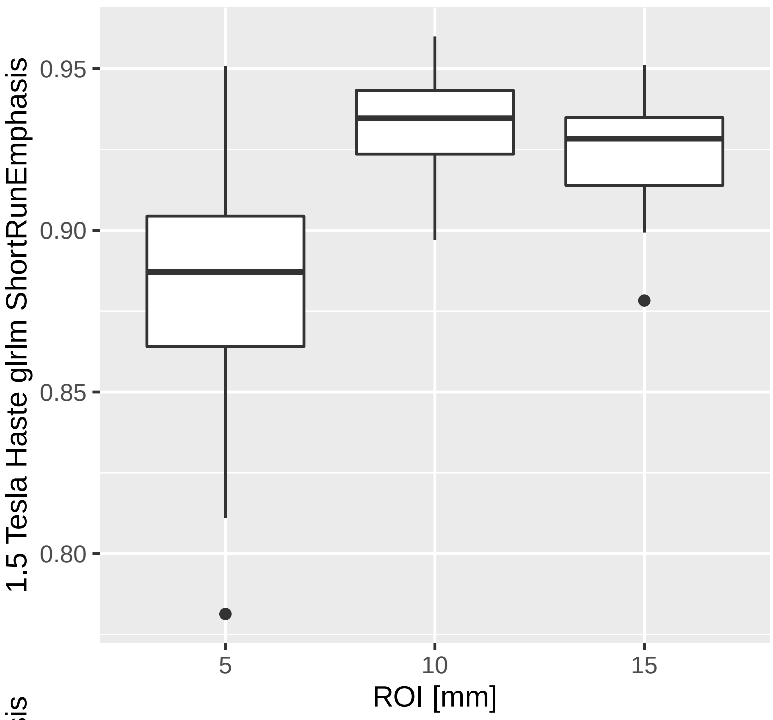
1.5 Tesla Haste glrlm ShortRunEmphasis
0.80
0.85
0.90
0.95
5
10
15
ROI [mm]

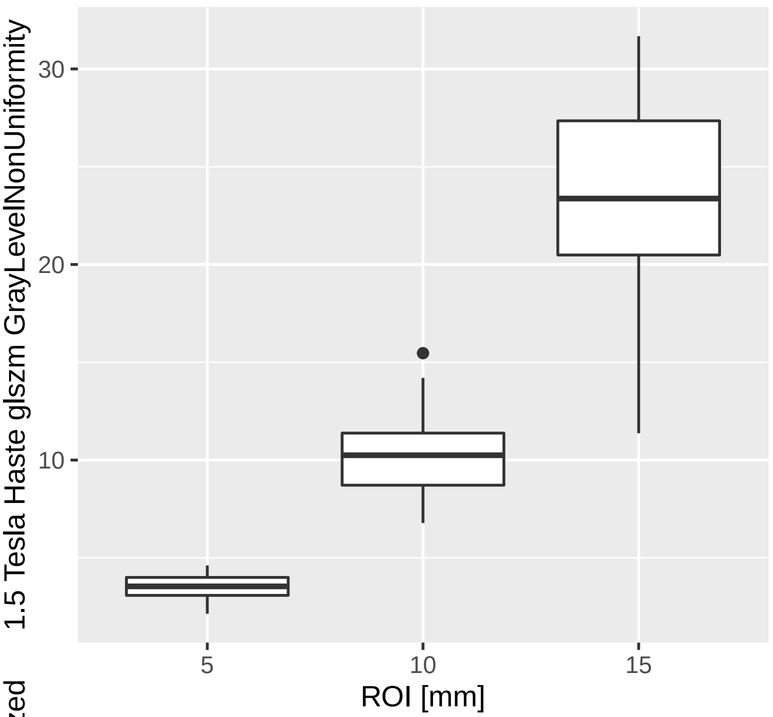
1.5 Tesla Haste glszm GrayLevelNonUniformity
10
20
30
5
10
15
ROI [mm]

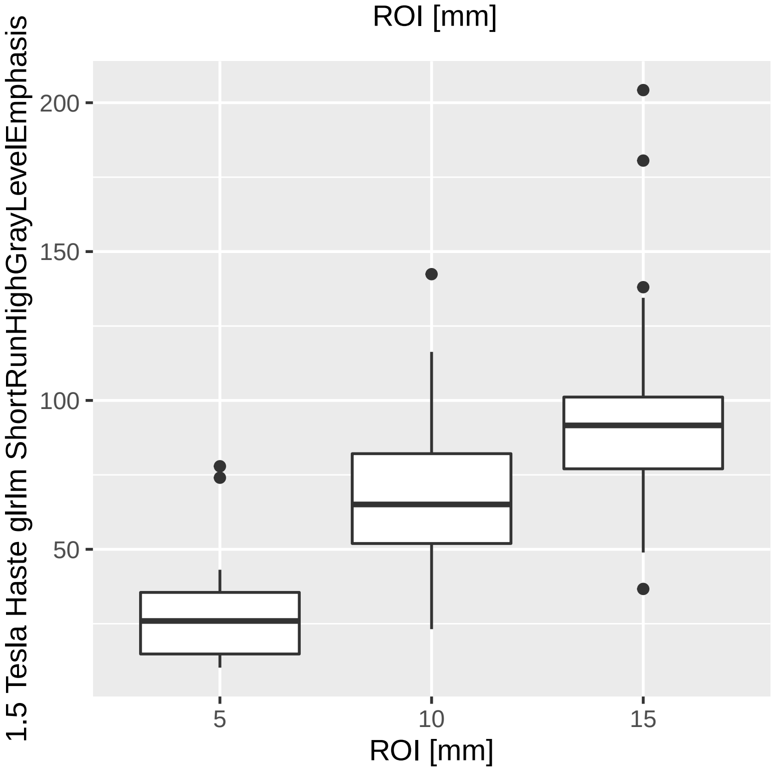
1.5 Tesla Haste glrlm ShortRunHighGrayLevelEmphasis
50
100
150
200
5
10
15
ROI [mm]

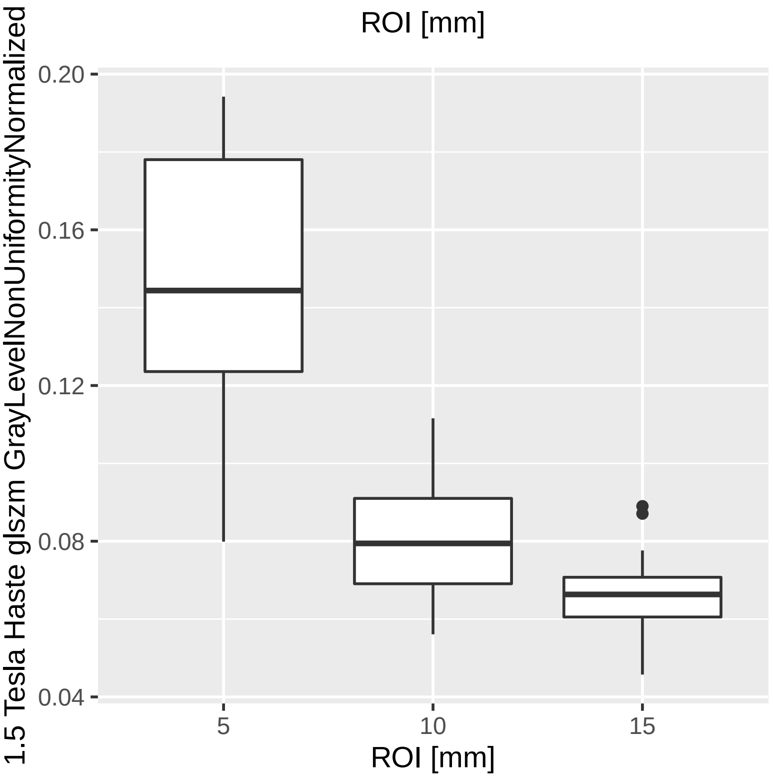
1.5 Tesla Haste glszm GrayLevelNonUniformityNormalized
0.04
0.08
0.12
0.16
0.20
5
10
15
ROI [mm]

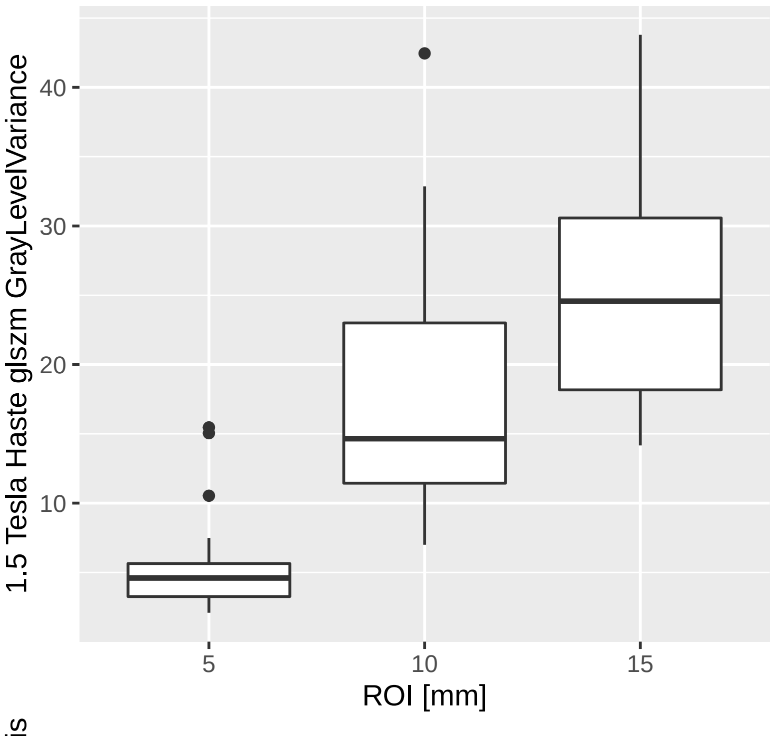
1.5 Tesla Haste glszm GrayLevelVariance
40
30
20
10
5
10
15
ROI [mm]

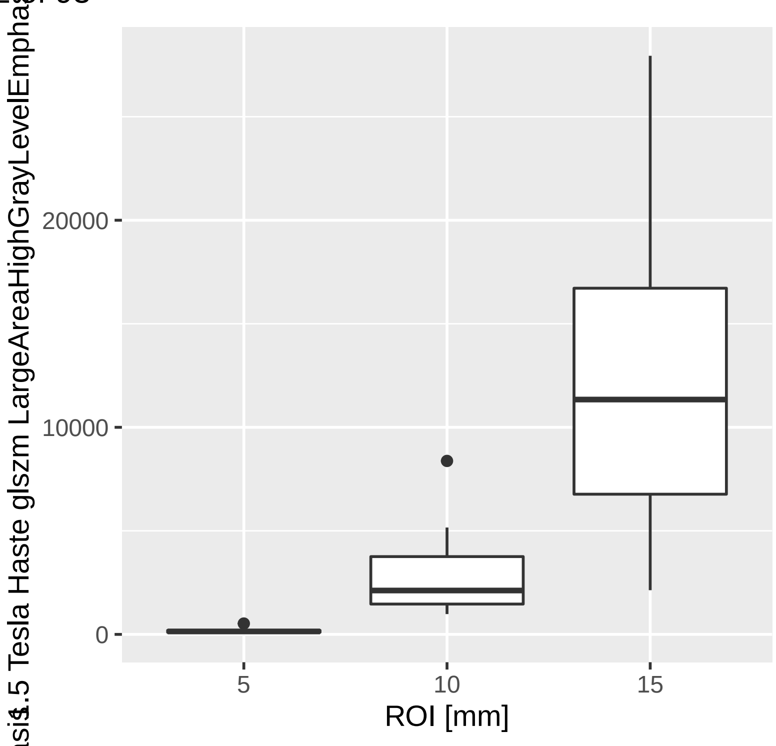
1.5 Tesla Haste glszm LargeAreaHighGrayLevelEmphasis
20000
10000
0
5
10
15
ROI [mm]

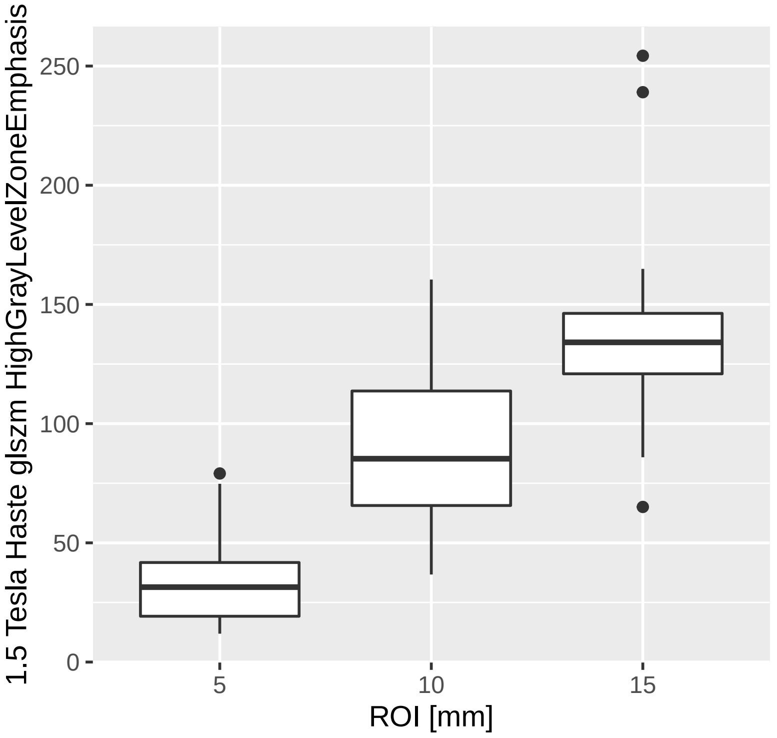
1.5 Tesla Haste glszm HighGrayLevelZoneEmphasis
250
200
150
100
50
0
5
10
15
ROI [mm]

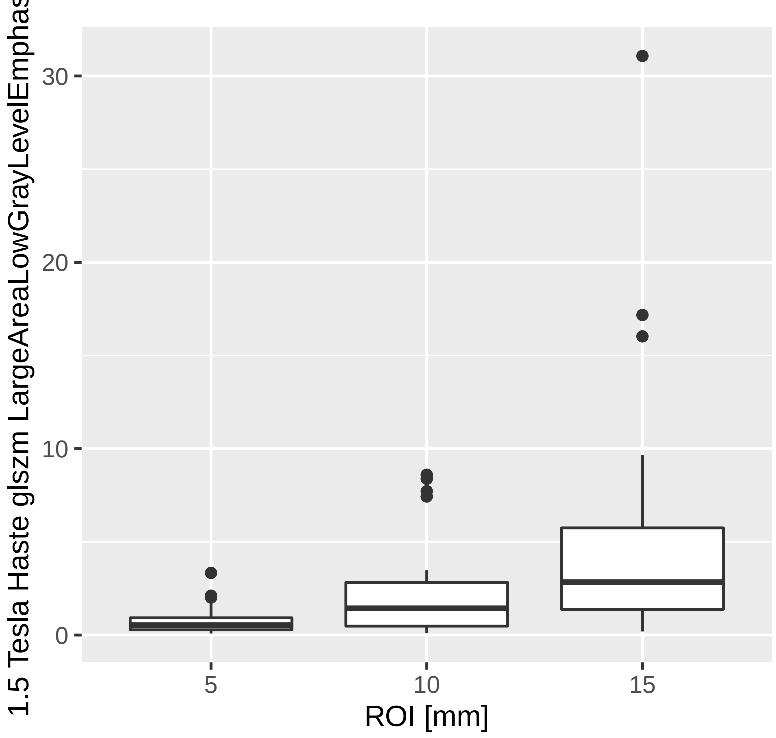
1.5 Tesla Haste glszm LargeAreaLowGrayLevelEmphasis
30
20
10
0
5
10
15
ROI [mm]

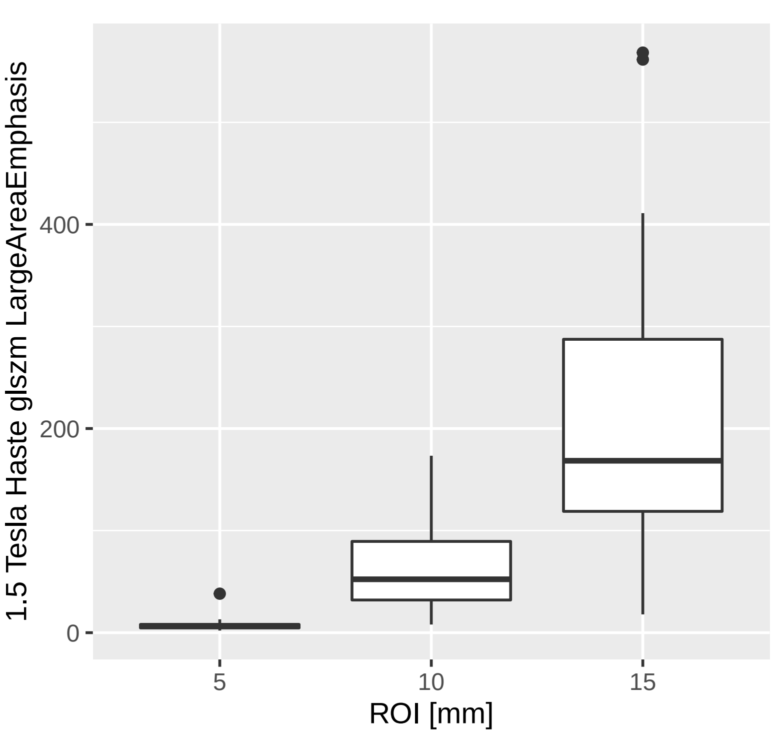
1.5 Tesla Haste glszm LargeAreaEmphasis
400
200
0
5
10
15
ROI [mm]

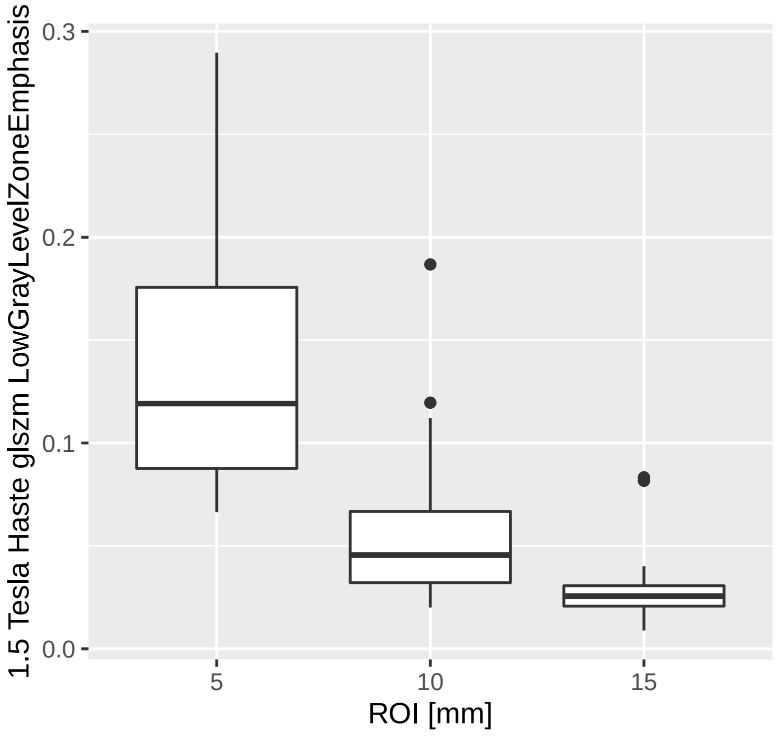
1.5 Tesla Haste glszm LowGrayLevelZoneEmphasis
0.3
0.2
0.1
0.0
5
10
15
ROI [mm]

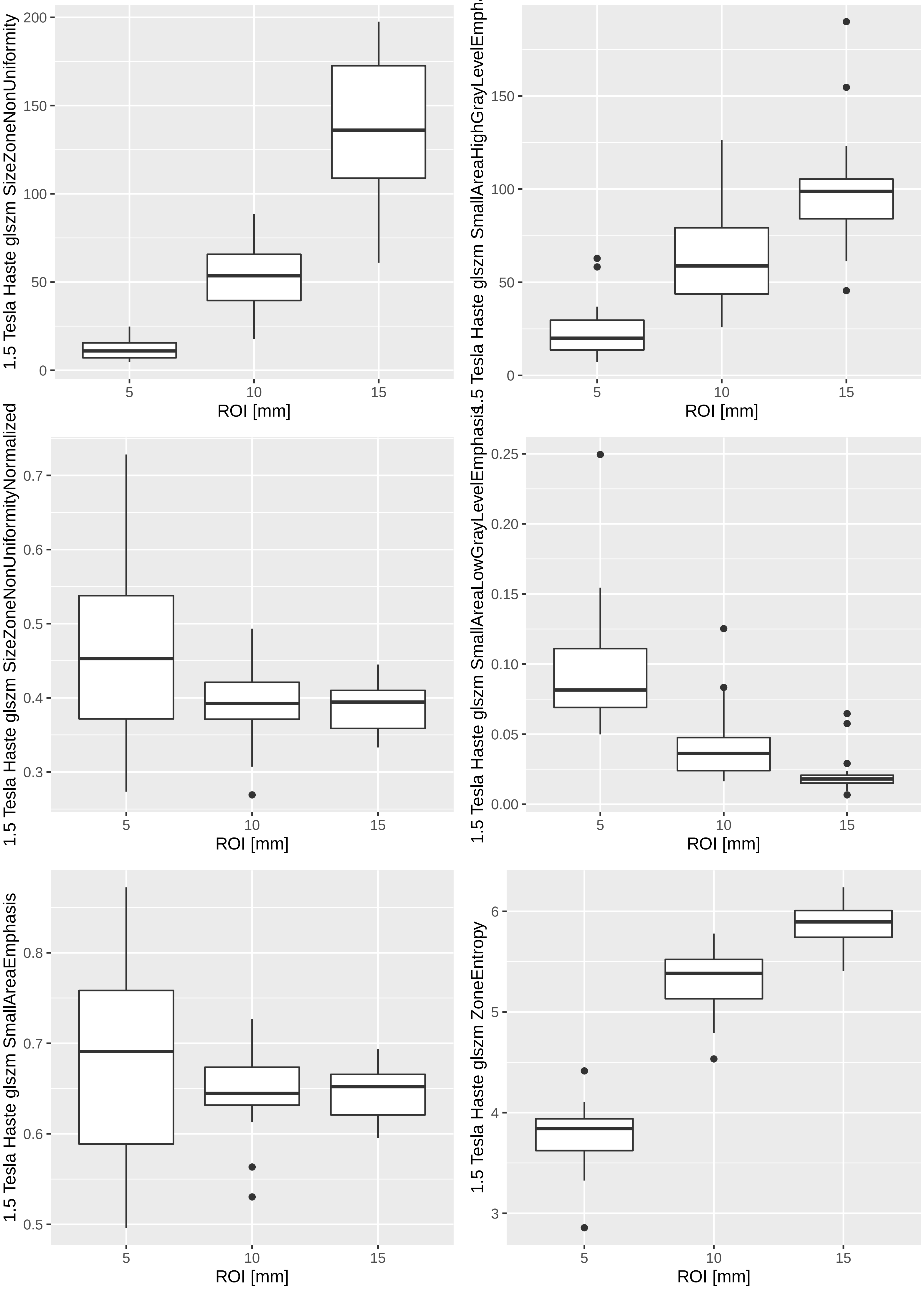
1.5 Tesla Haste glszm SizeZoneNonUniformity
ROI [mm]
1.5 Tesla Haste glszm SmallAreaHighGrayLevelEmphasis
ROI [mm]
1.5 Tesla Haste glszm SizeZoneNonUniformityNormalized
ROI [mm]
1.5 Tesla Haste glszm SmallAreaLowGrayLevelEmphasis
ROI [mm]
1.5 Tesla Haste glszm SmallAreaEmphasis
ROI [mm]
1.5 Tesla Haste glszm ZoneEntropy
ROI [mm]

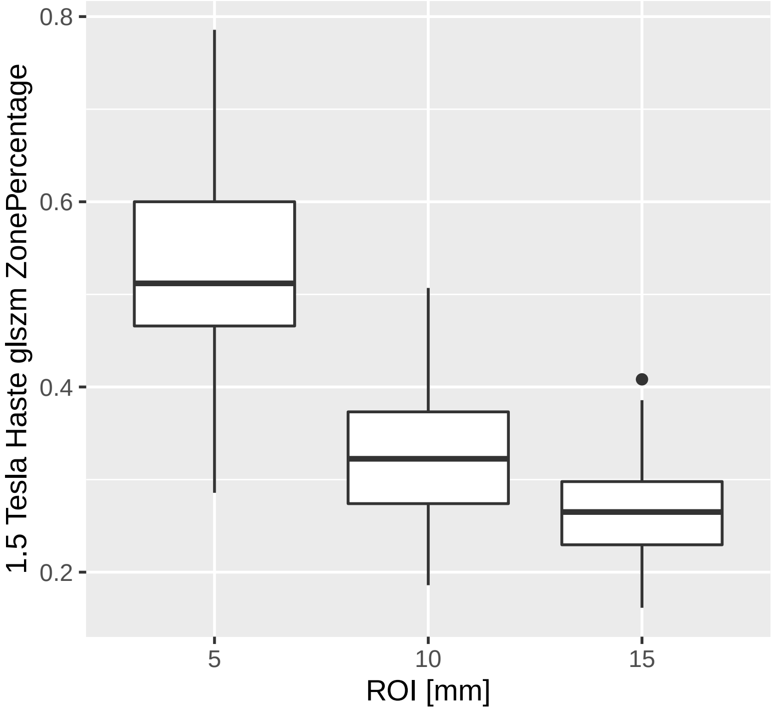
1.5 Tesla Haste glszm ZonePercentage
0.8
0.6
0.4
0.2
5
10
15
ROI [mm]

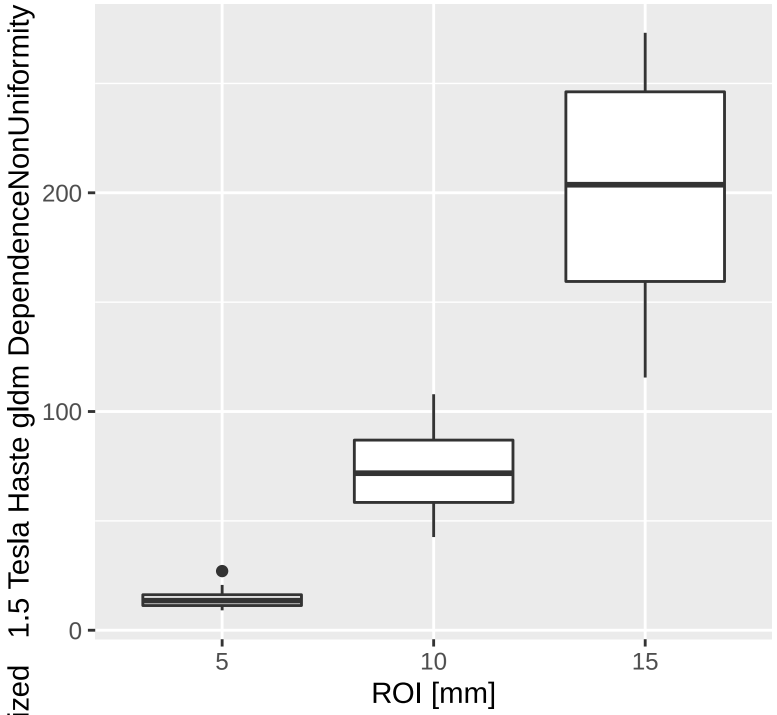
1.5 Tesla Haste gldm DependenceNonUniformity
200
100
0
5
10
15
ROI [mm]

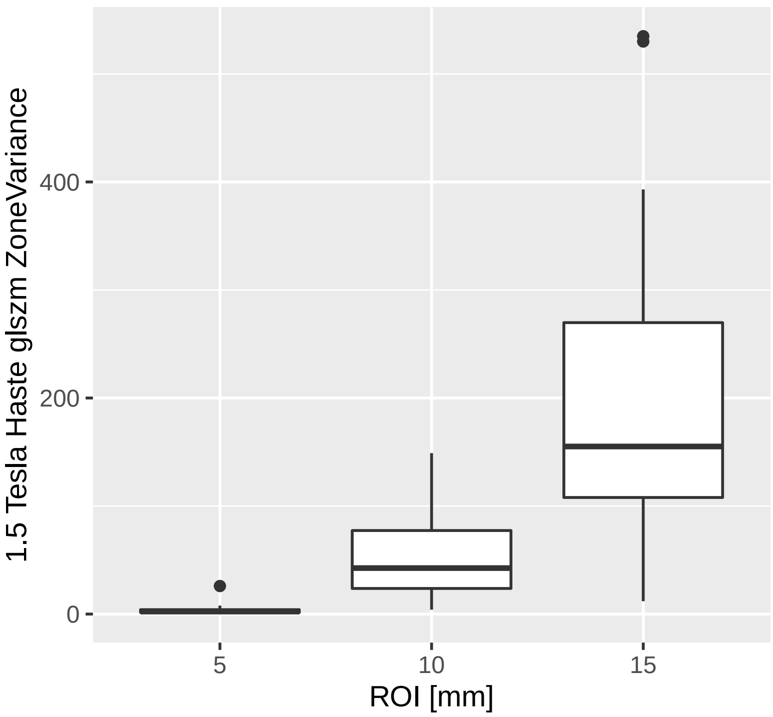
1.5 Tesla Haste glszm ZoneVariance
400
200
0
5
10
15
ROI [mm]

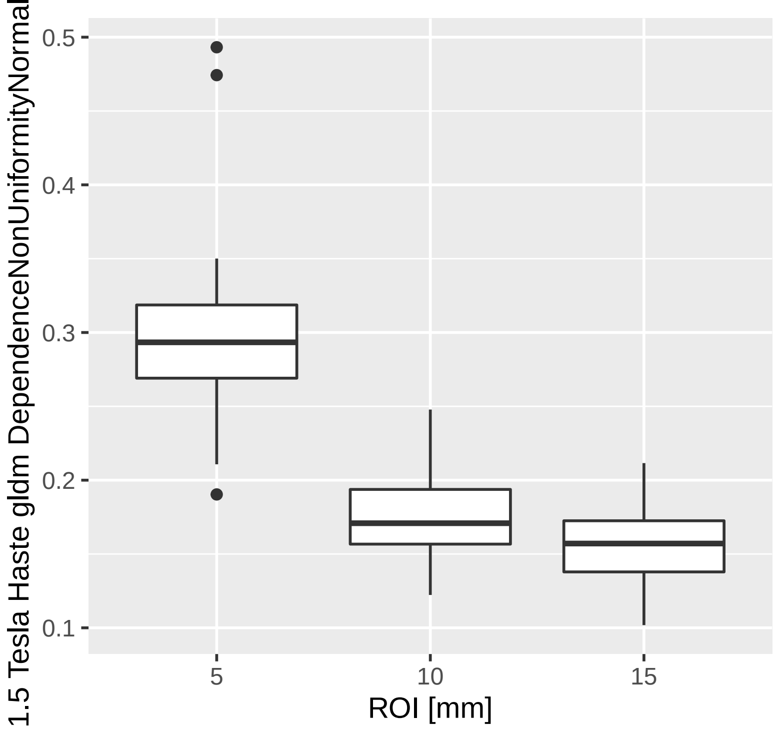
1.5 Tesla Haste gldm DependenceNonUniformityNormalized
0.5
0.4
0.3
0.2
0.1
5
10
15
ROI [mm]

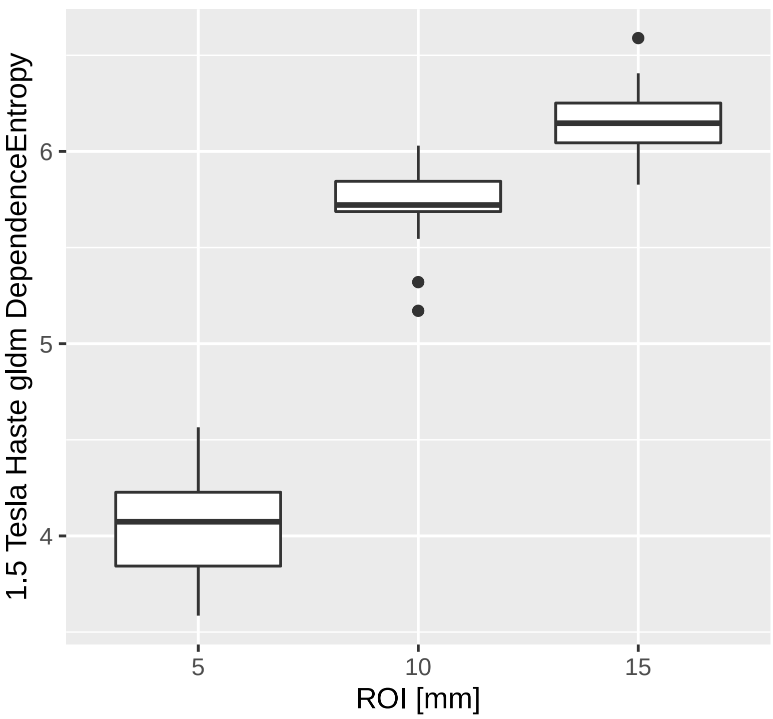
1.5 Tesla Haste gldm DependenceEntropy
6
5
4
5
10
15
ROI [mm]

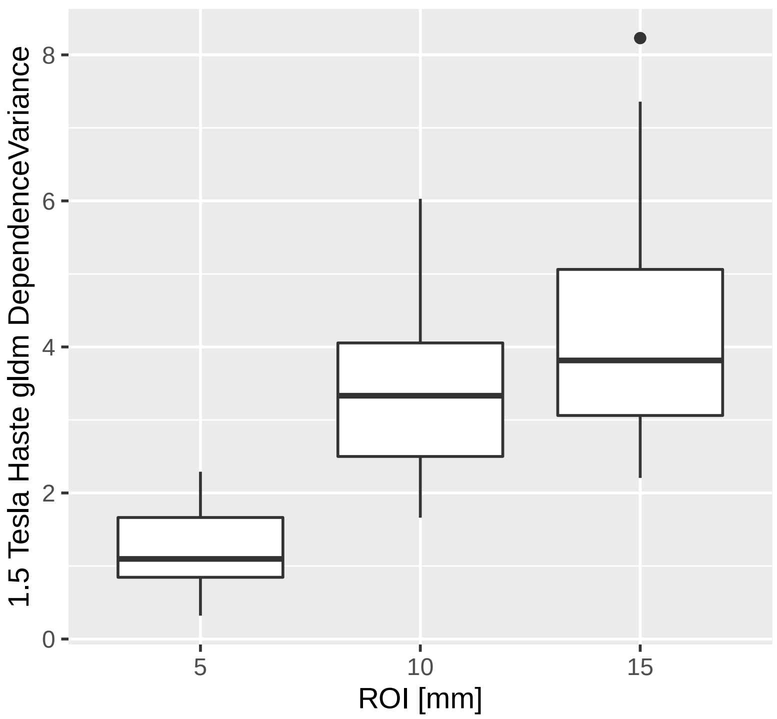
1.5 Tesla Haste gldm DependenceVariance
8
6
4
2
0
5
10
15
ROI [mm]

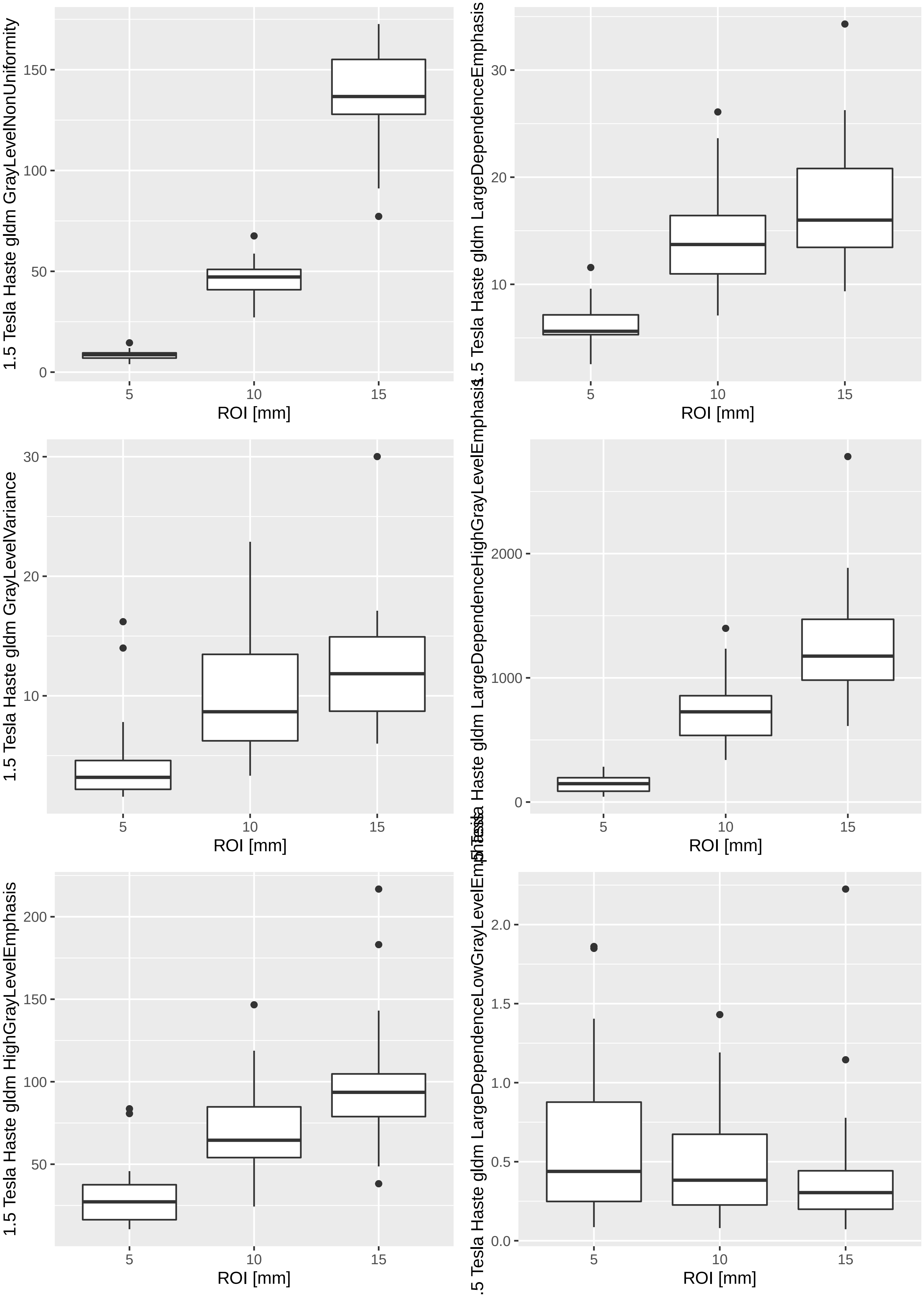
1.5 Tesla Haste gldm GrayLevelNonUniformity
ROI [mm]
1.5 Tesla Haste gldm LargeDependenceEmphasis
ROI [mm]
1.5 Tesla Haste gldm GrayLevelVariance
ROI [mm]
1.5 Tesla Haste gldm LargeDependenceHighGrayLevelEmphasis
ROI [mm]
1.5 Tesla Haste gldm HighGrayLevelEmphasis
ROI [mm]
1.5 Tesla Haste gldm LargeDependenceLowGrayLevelEmphasis
ROI [mm]

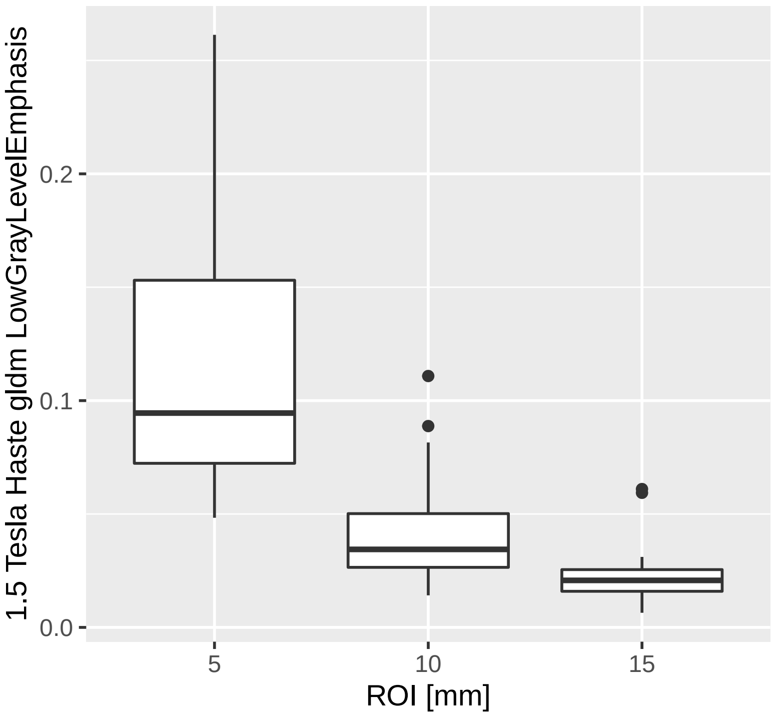
1.5 Tesla Haste gldm LowGrayLevelEmphasis
0.0
0.1
0.2
5
10
15
ROI [mm]

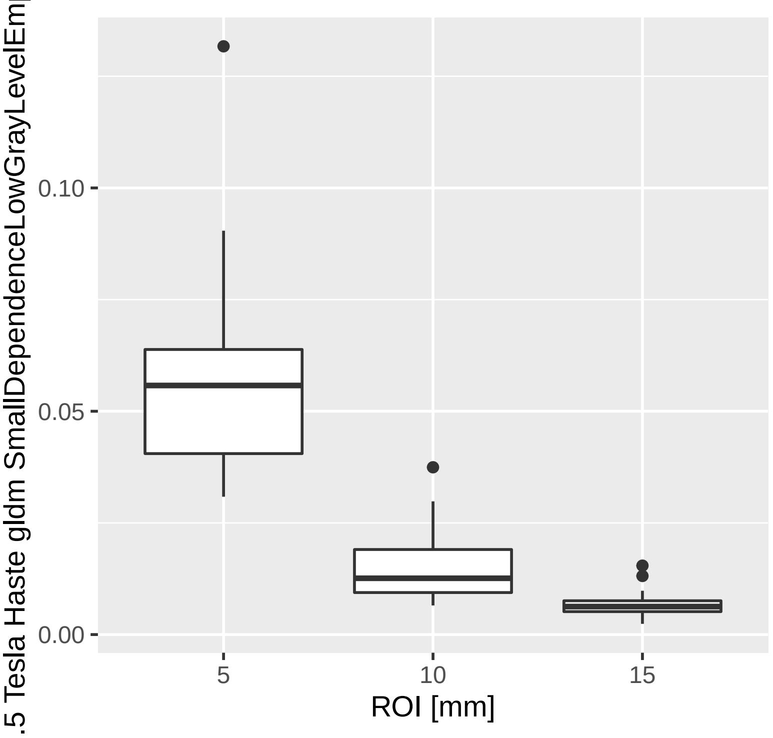
.5 Tesla Haste gldm SmallDependenceLowGrayLevelEmpha
0.00
0.05
0.10
5
10
15
ROI [mm]

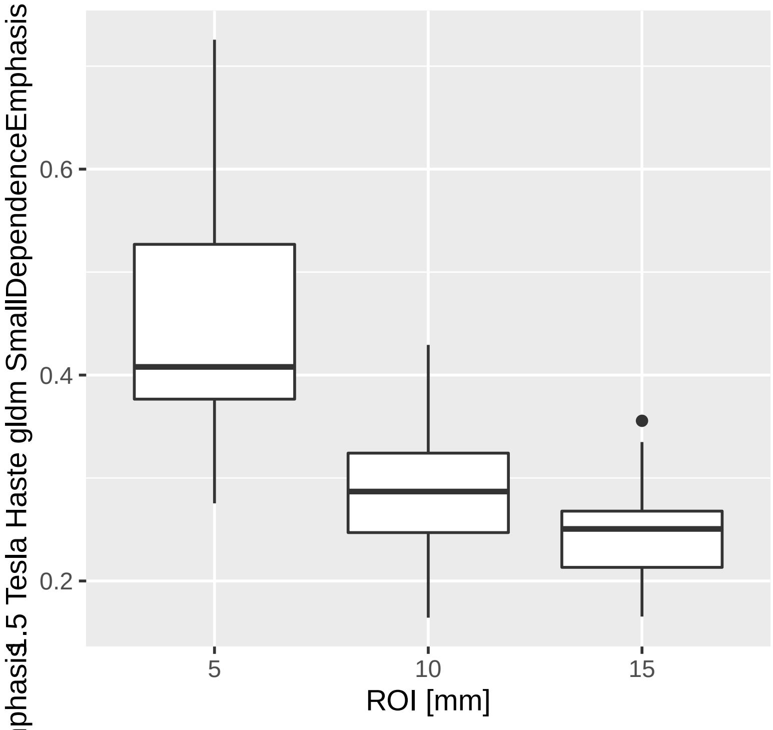
1.5 Tesla Haste gldm SmallDependenceEmphasis
0.2
0.4
0.6
5
10
15
ROI [mm]

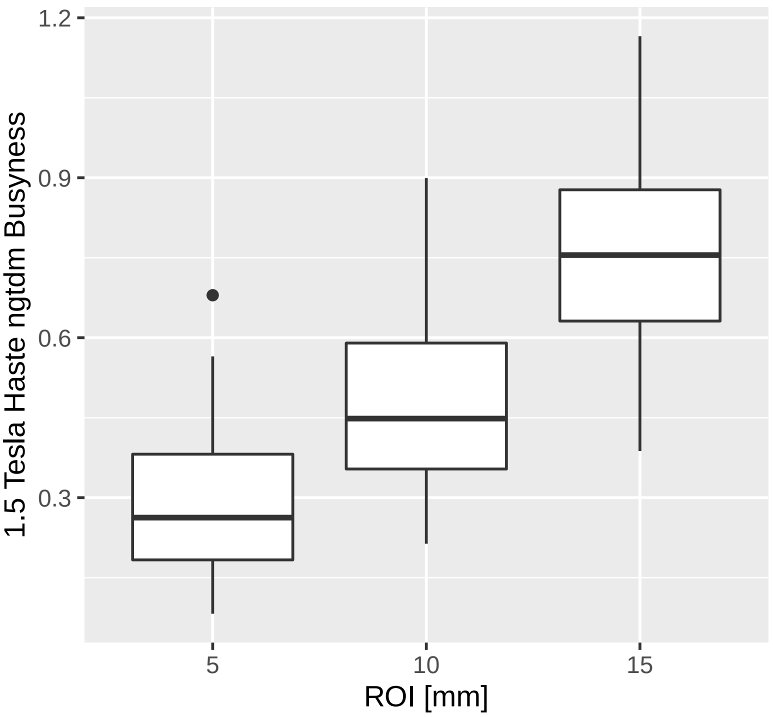
1.5 Tesla Haste ngtdm Busyness
0.3
0.6
0.9
1.2
5
10
15
ROI [mm]

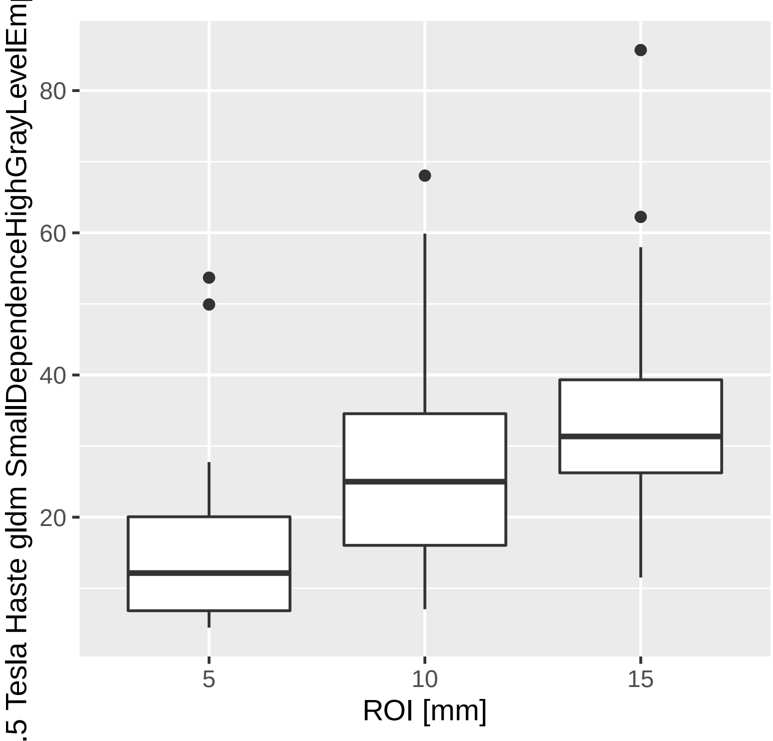
.5 Tesla Haste gldm SmallDependenceHighGrayLevelEmphasis
20
40
60
80
5
10
15
ROI [mm]

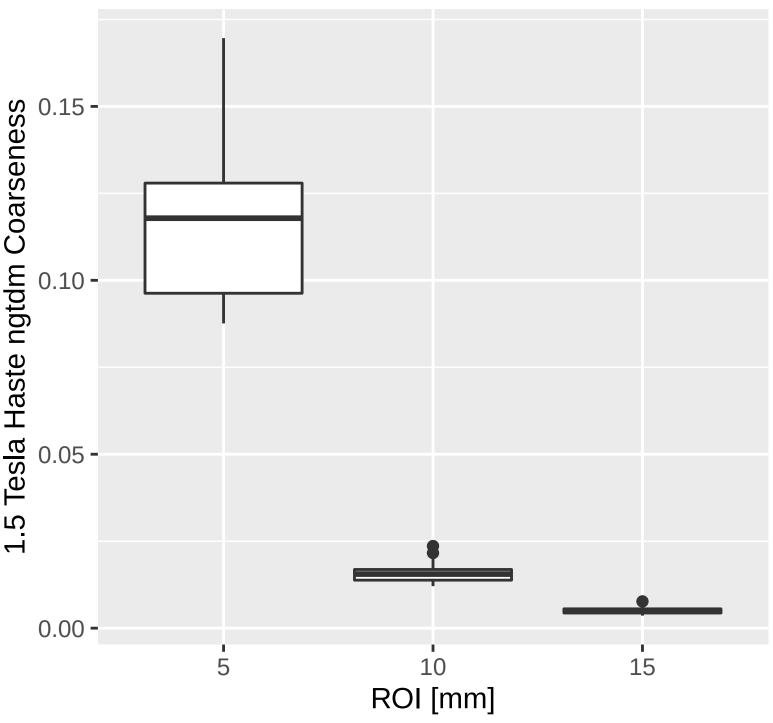
1.5 Tesla Haste ngtdm Coarseness
0.00
0.05
0.10
0.15
5
10
15
ROI [mm]

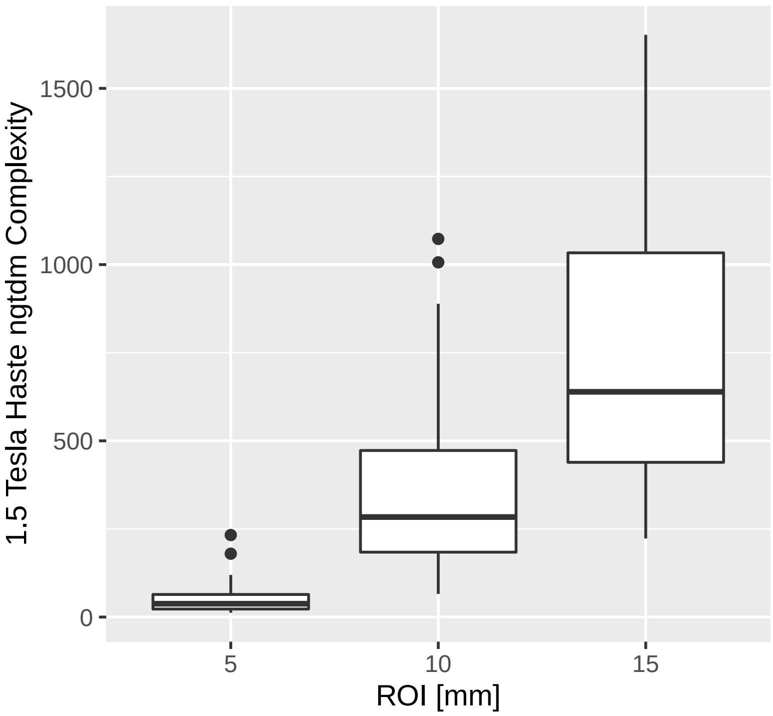
1.5 Tesla Haste ngtdm Complexity
0
500
1000
1500
5
10
15
ROI [mm]

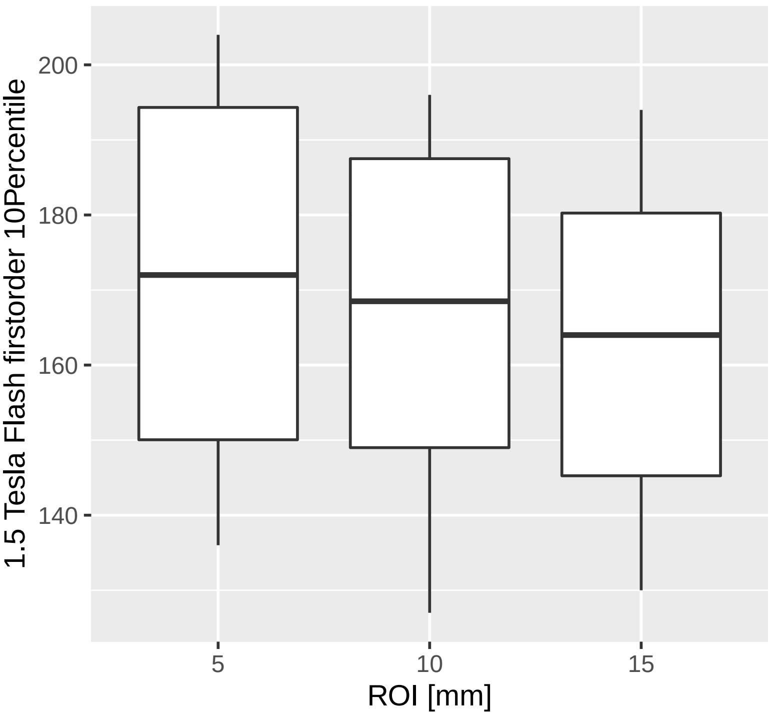
1.5 Tesla Flash firstorder 10Percentile
140
160
180
200
5
10
15
ROI [mm]

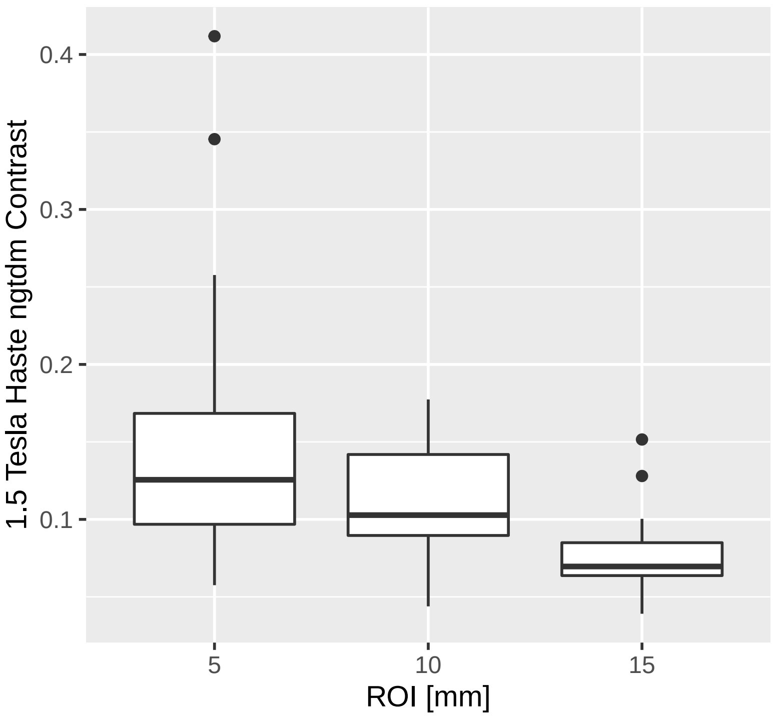
1.5 Tesla Haste ngtdm Contrast
0.1
0.2
0.3
0.4
5
10
15
ROI [mm]

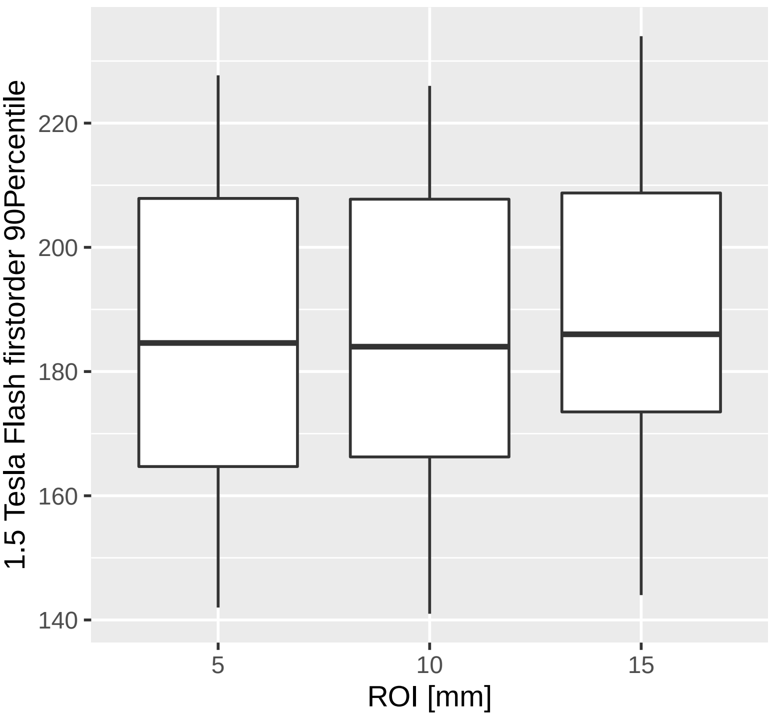
1.5 Tesla Flash firstorder 90Percentile
140
160
180
200
220
5
10
15
ROI [mm]

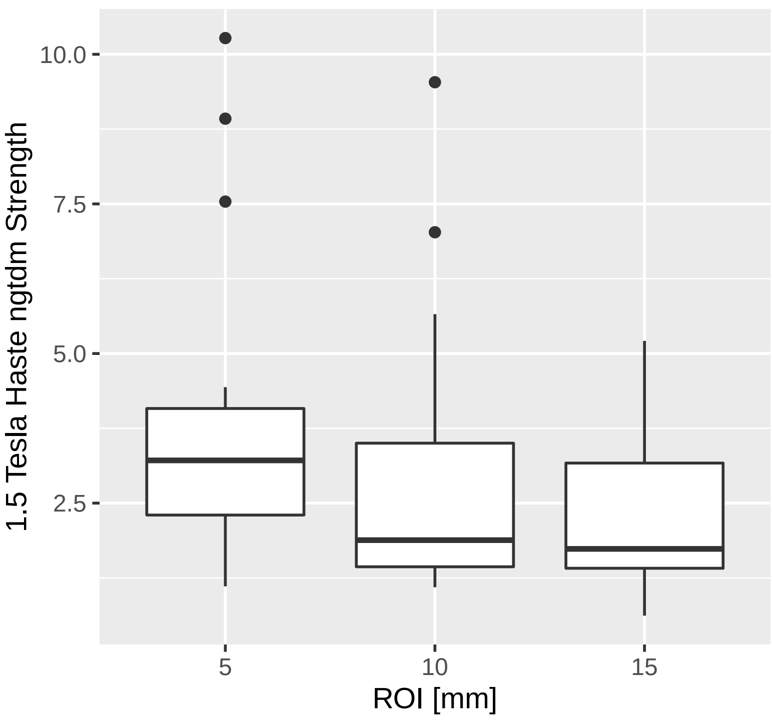
1.5 Tesla Haste ngtdm Strength
2.5
5.0
7.5
10.0
5
10
15
ROI [mm]

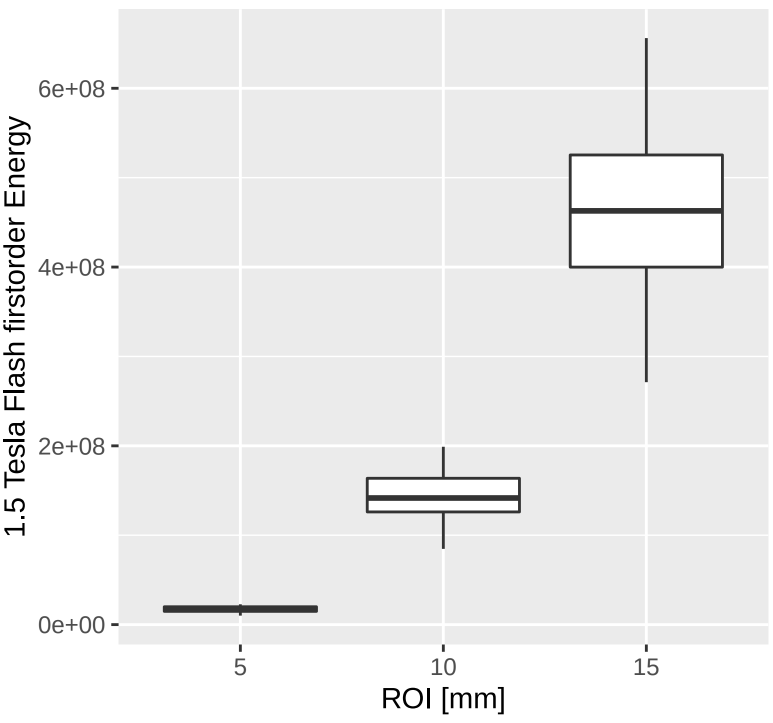
1.5 Tesla Flash firstorder Energy
0e+00
2e+08
4e+08
6e+08
5
10
15
ROI [mm]

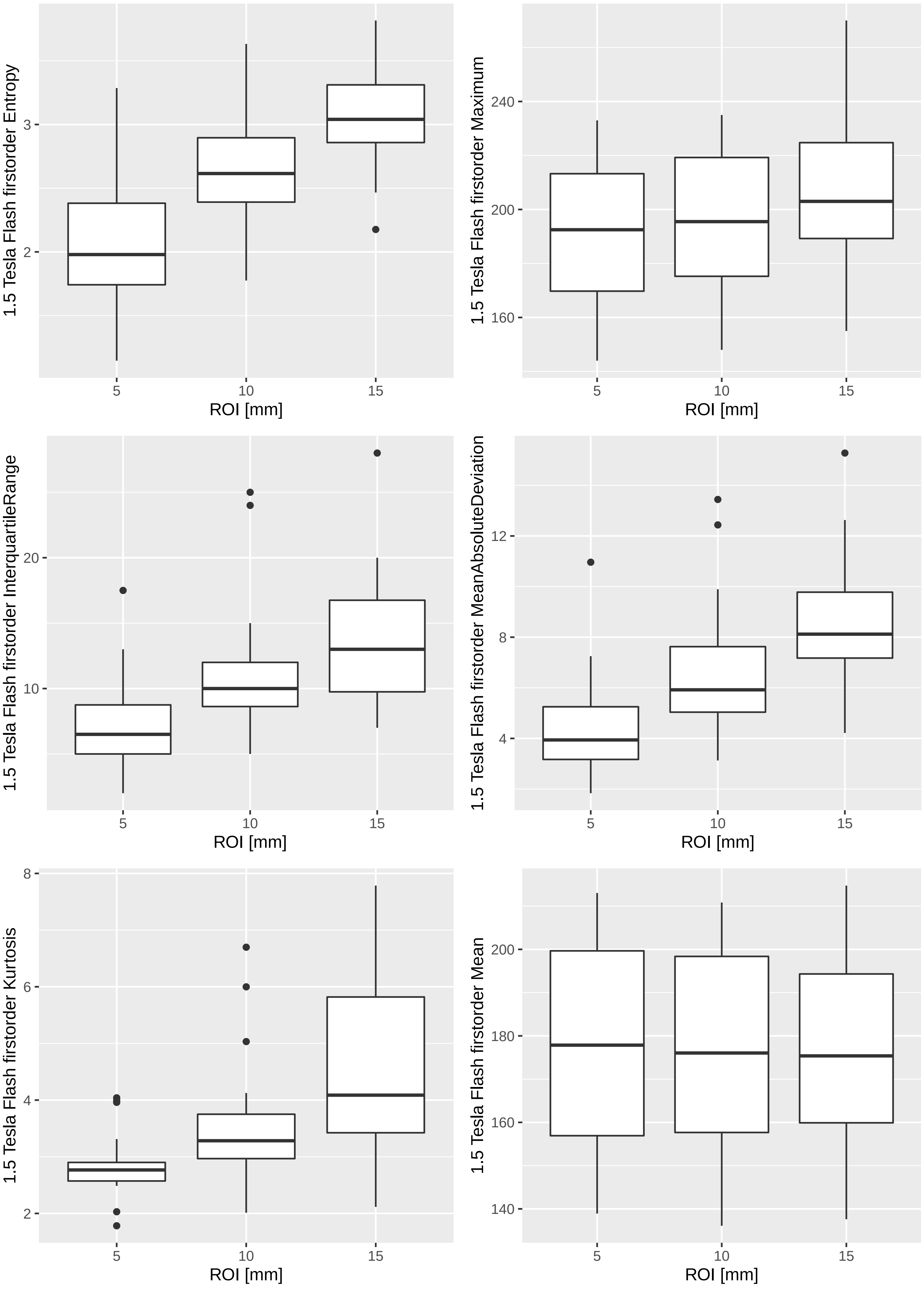
1.5 Tesla Flash firstorder Entropy
2
3
5
10
15
ROI [mm]
1.5 Tesla Flash firstorder Maximum
160
200
240
5
10
15
ROI [mm]
1.5 Tesla Flash firstorder InterquartileRange
10
20
5
10
15
ROI [mm]
1.5 Tesla Flash firstorder MeanAbsoluteDeviation
4
8
12
5
10
15
ROI [mm]
1.5 Tesla Flash firstorder Kurtosis
2
4
6
8
5
10
15
ROI [mm]
1.5 Tesla Flash firstorder Mean
140
160
180
200
5
10
15
ROI [mm]

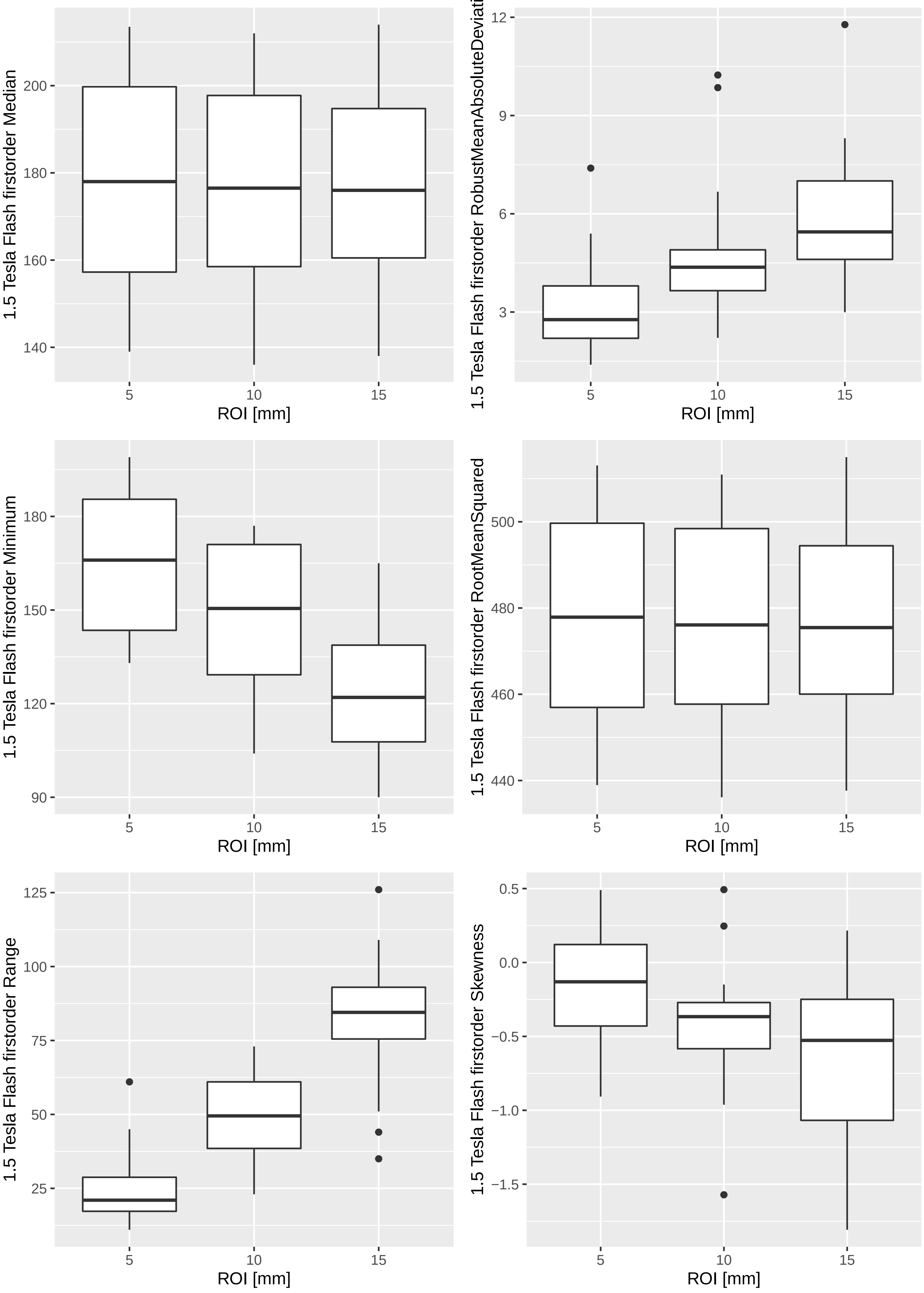
1.5 Tesla Flash firstorder Median
200
180
160
140
1.5 Tesla Flash firstorder RobustMeanAbsoluteDeviation
12
9
6
3
1.5 Tesla Flash firstorder Minimum
180
150
120
90
1.5 Tesla Flash firstorder RootMeanSquared
500
480
460
440
1.5 Tesla Flash firstorder Range
125
100
75
50
25
1.5 Tesla Flash firstorder Skewness
0.5
0.0
−0.5
−1.0
−1.5
5
10
15
ROI [mm]

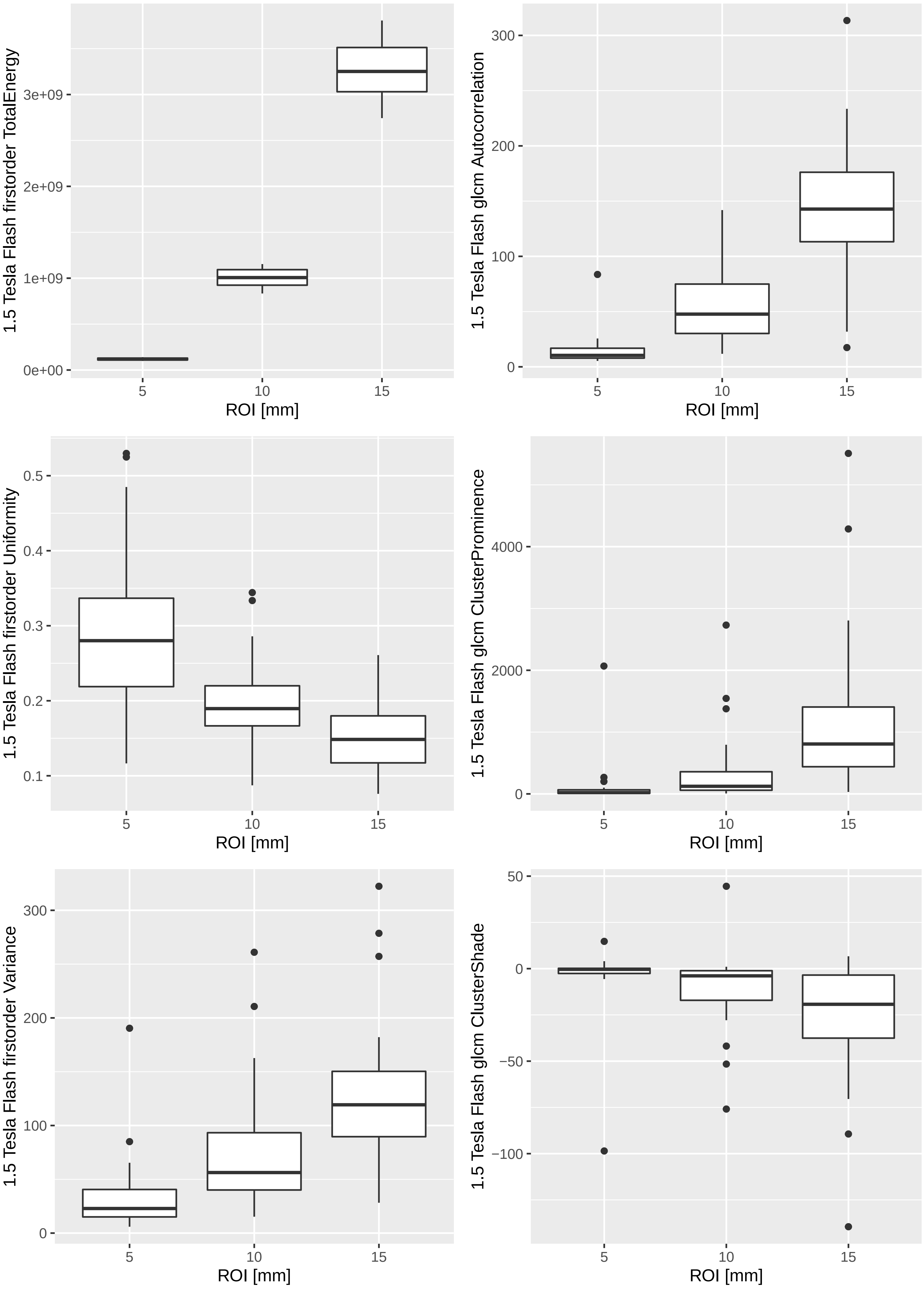
1.5 Tesla Flash firstorder TotalEnergy
0e+00
1e+09
2e+09
3e+09
1.5 Tesla Flash glcm Autocorrelation
0
100
200
300
1.5 Tesla Flash firstorder Uniformity
0.1
0.2
0.3
0.4
0.5
1.5 Tesla Flash glcm ClusterProminence
0
2000
4000
1.5 Tesla Flash firstorder Variance
0
100
200
300
1.5 Tesla Flash glcm ClusterShade
−100
−50
0
50
5
10
15
ROI [mm]

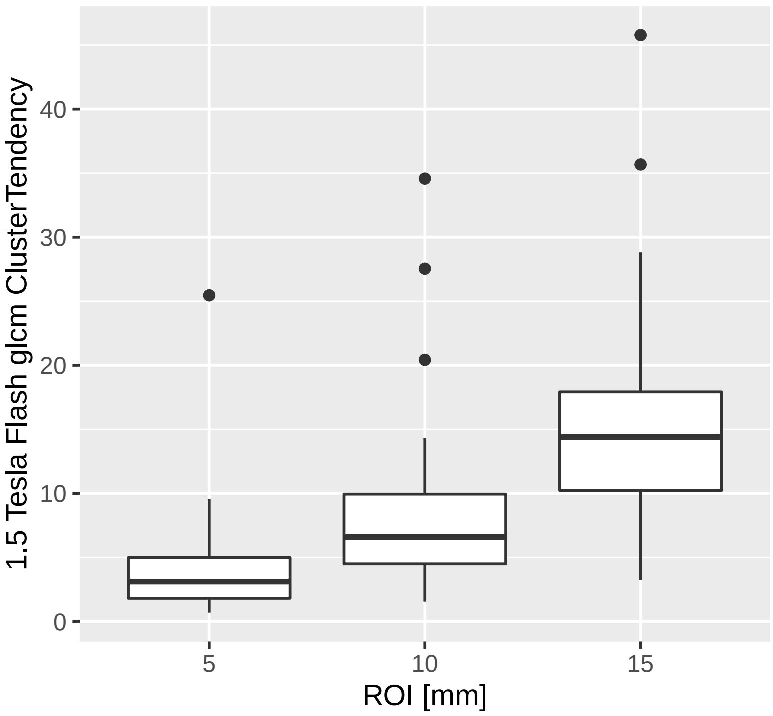

1.5 Tesla Flash glcm ClusterTendency
0
10
20
30
40
5
10
15
ROI [mm]

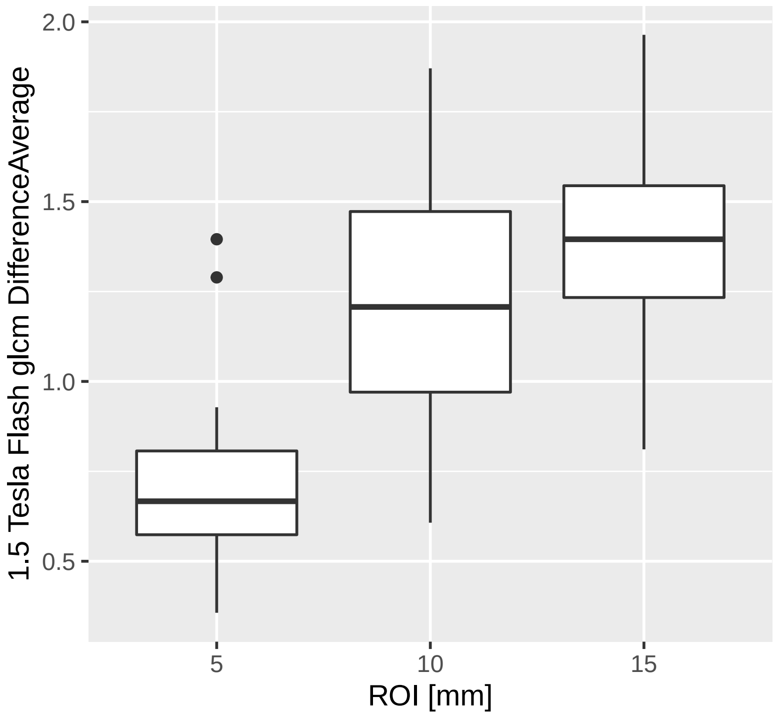

1.5 Tesla Flash glcm DifferenceAverage
0.5
1.0
1.5
2.0
5
10
15
ROI [mm]

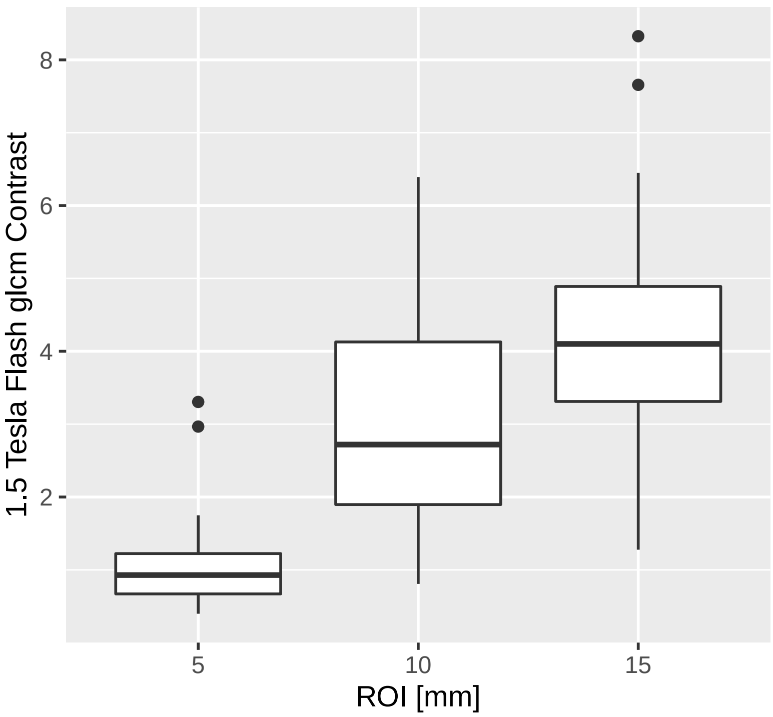

1.5 Tesla Flash glcm Contrast
2
4
6
8
5
10
15
ROI [mm]

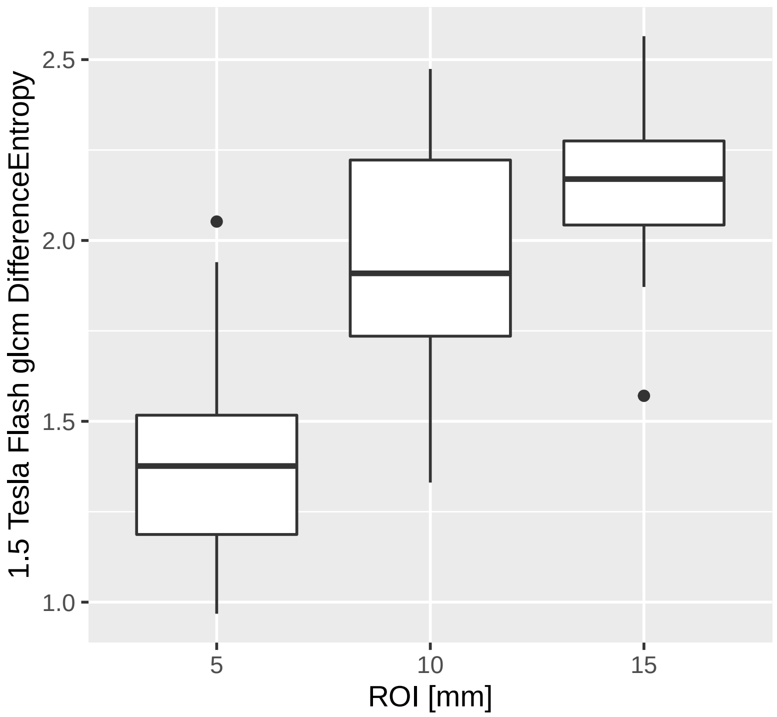

1.5 Tesla Flash glcm DifferenceEntropy
1.0
1.5
2.0
2.5
5
10
15
ROI [mm]

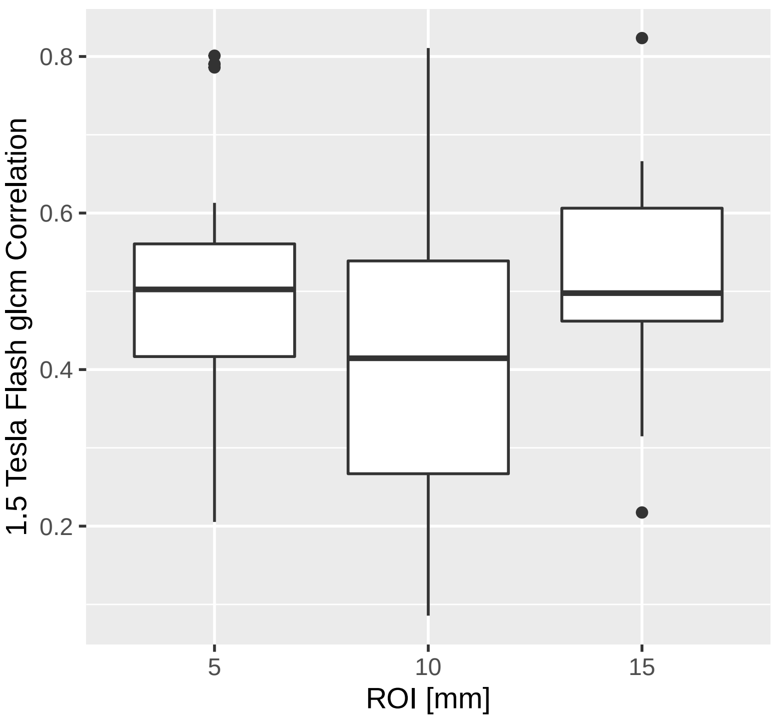

1.5 Tesla Flash glcm Correlation
0.2
0.4
0.6
0.8
5
10
15
ROI [mm]

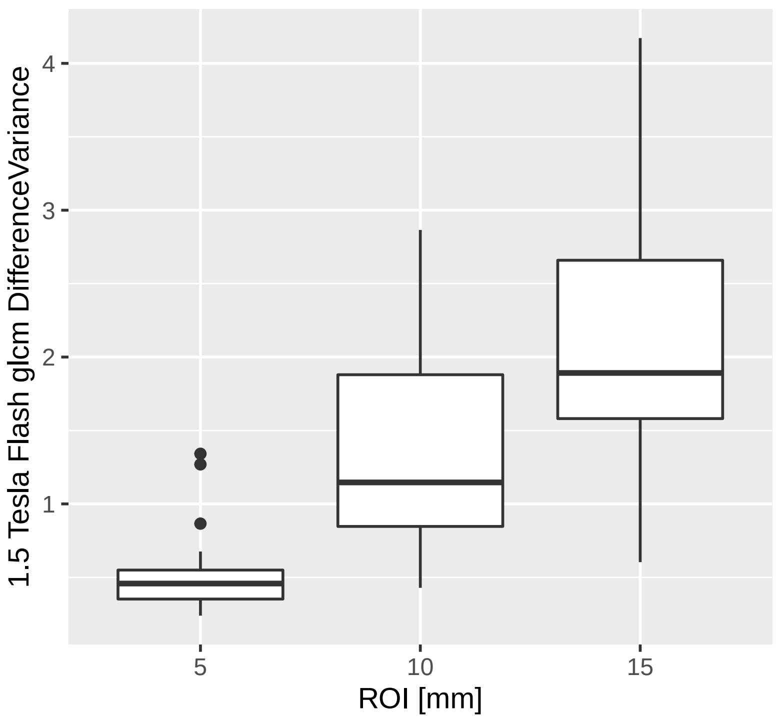

1.5 Tesla Flash glcm DifferenceVariance
1
2
3
4
5
10
15
ROI [mm]

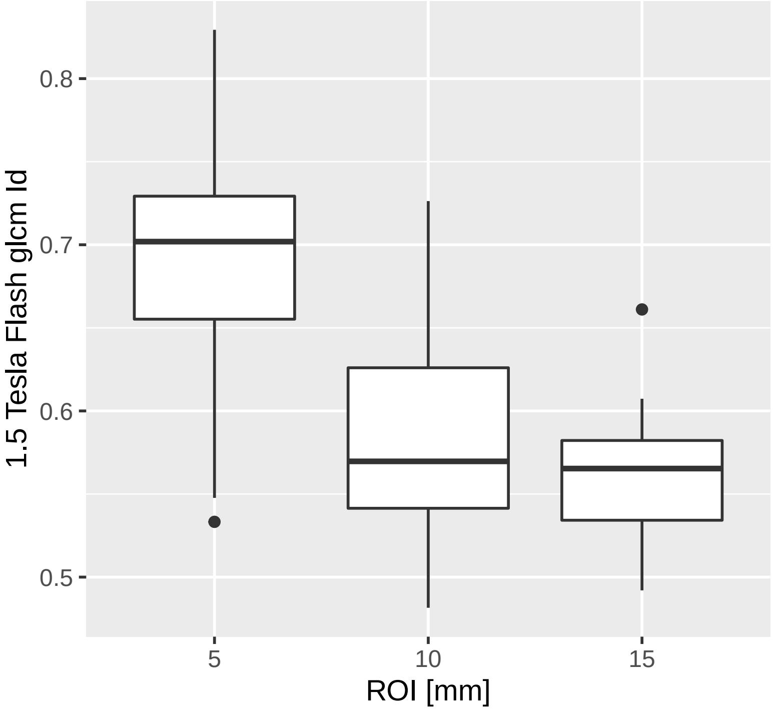
1.5 Tesla Flash glcm Id
0.8
0.7
0.6
0.5
5
10
15
ROI [mm]

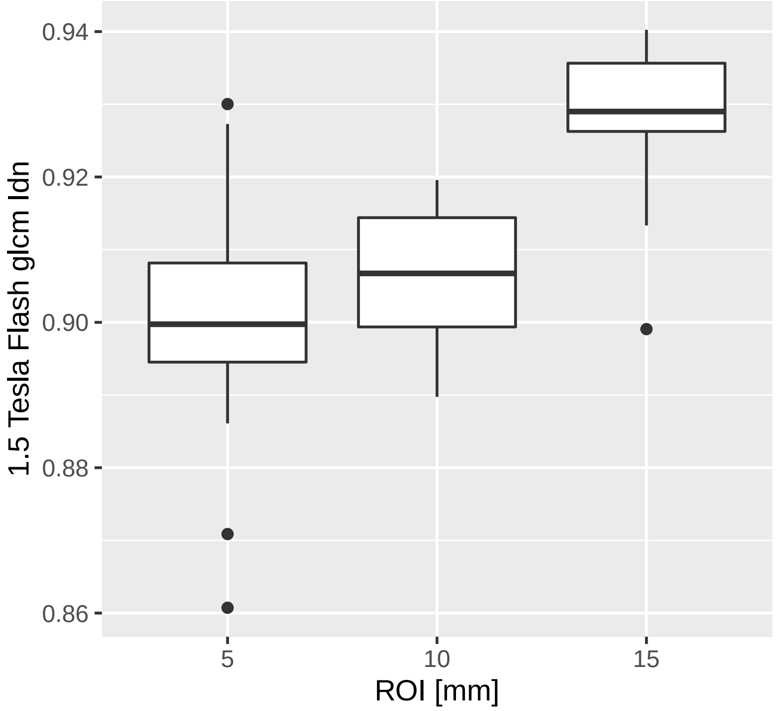
1.5 Tesla Flash glcm Idn
0.94
0.92
0.90
0.88
0.86
5
10
15
ROI [mm]

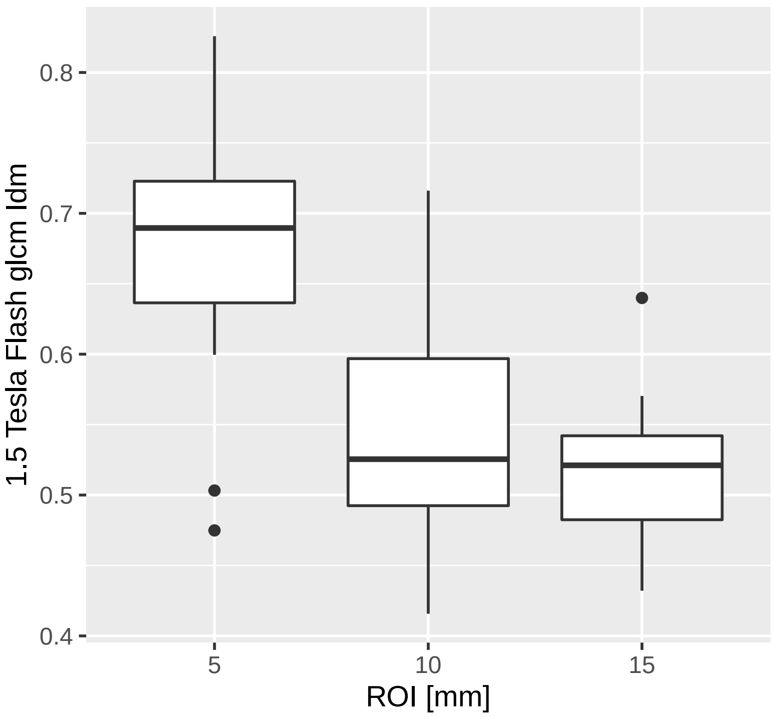
1.5 Tesla Flash glcm Idm
0.8
0.7
0.6
0.5
0.4
5
10
15
ROI [mm]

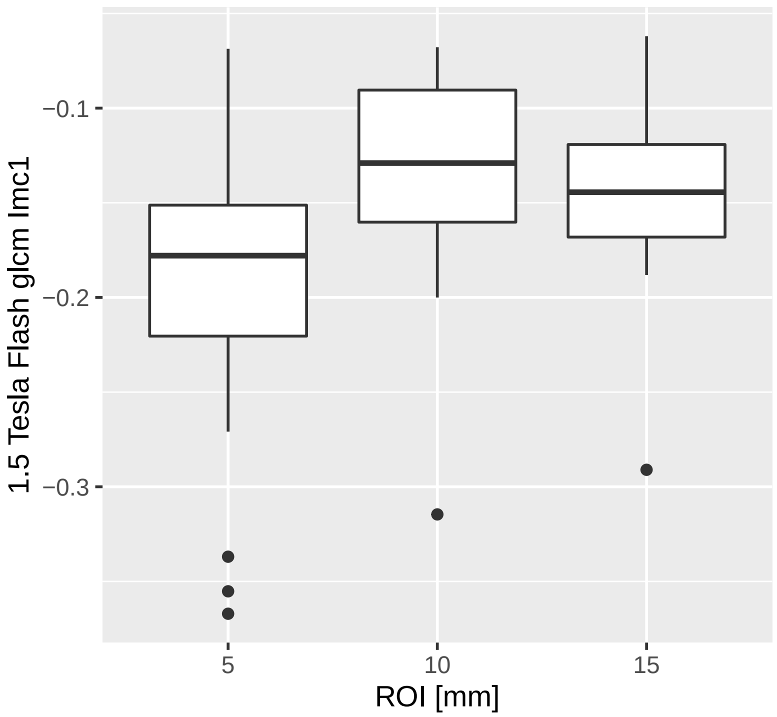
1.5 Tesla Flash glcm Imc1
−0.1
−0.2
−0.3
5
10
15
ROI [mm]

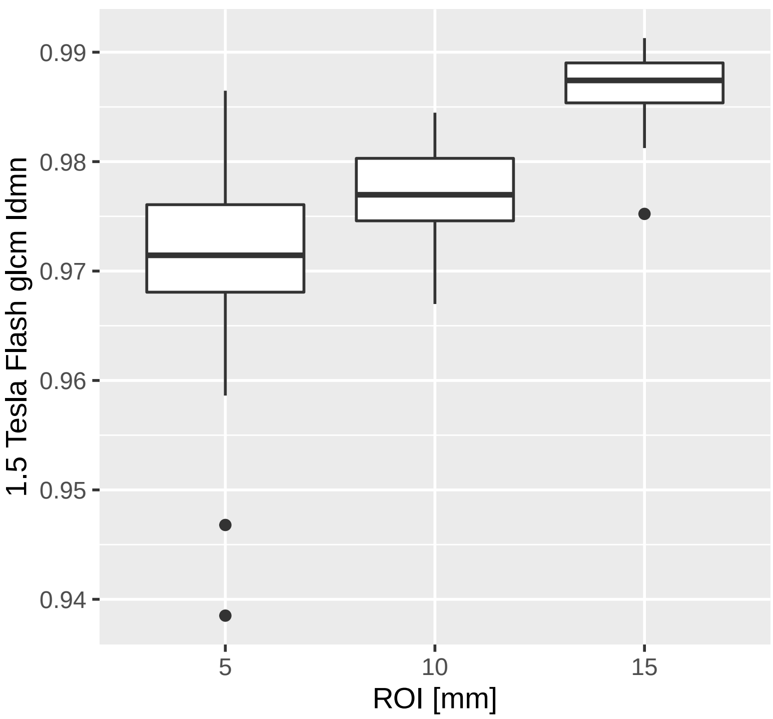
1.5 Tesla Flash glcm Idmn
0.99
0.98
0.97
0.96
0.95
0.94
5
10
15
ROI [mm]

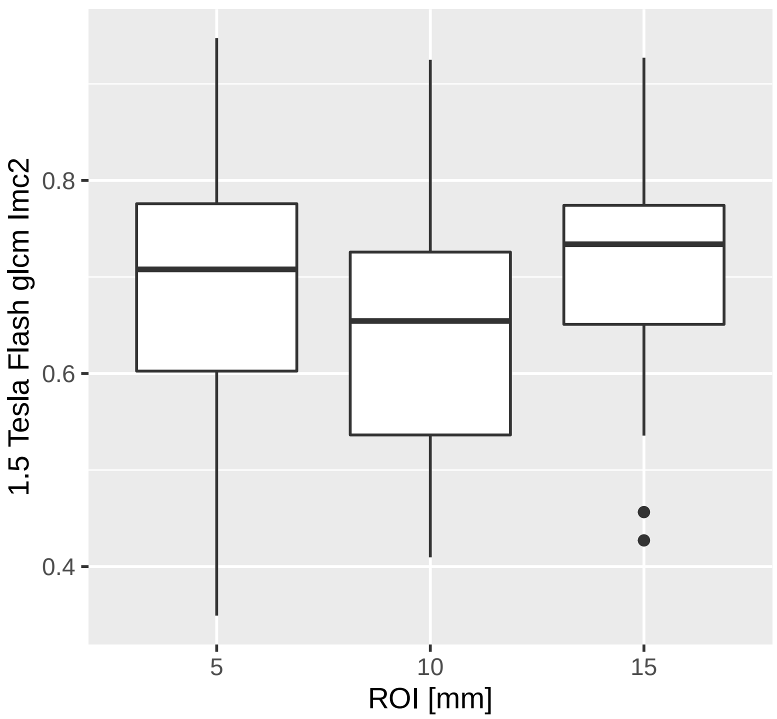
1.5 Tesla Flash glcm Imc2
0.8
0.6
0.4
5
10
15
ROI [mm]

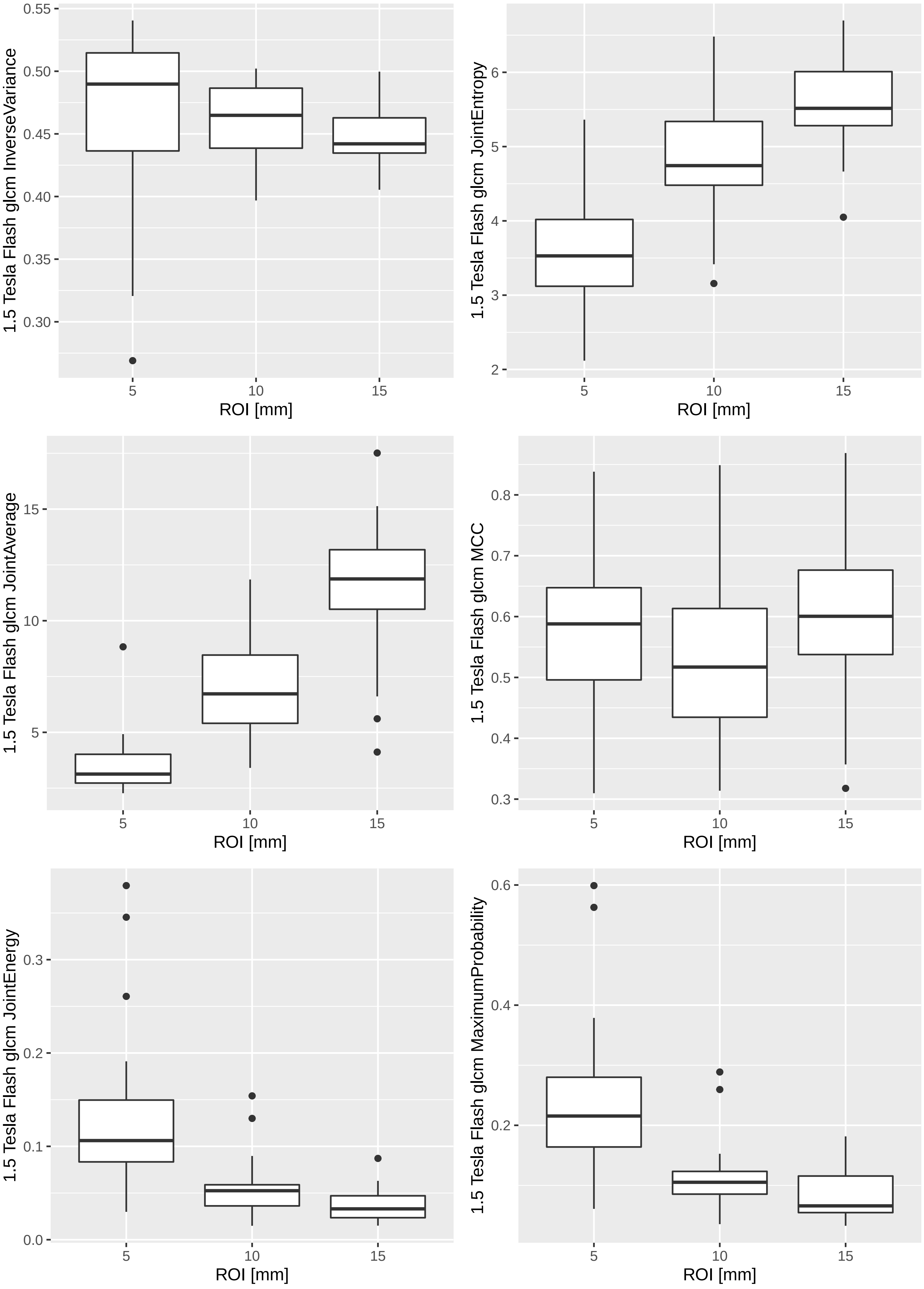
1.5 Tesla Flash glcm InverseVariance
0.55
0.50
0.45
0.40
0.35
0.30
5
10
15
ROI [mm]
1.5 Tesla Flash glcm JointEntropy
6
5
4
3
2
5
10
15
ROI [mm]
1.5 Tesla Flash glcm JointAverage
15
10
5
5
10
15
ROI [mm]
1.5 Tesla Flash glcm MCC
0.8
0.7
0.6
0.5
0.4
0.3
5
10
15
ROI [mm]
1.5 Tesla Flash glcm JointEnergy
0.3
0.2
0.1
0.0
5
10
15
ROI [mm]
1.5 Tesla Flash glcm MaximumProbability
0.6
0.4
0.2
5
10
15
ROI [mm]

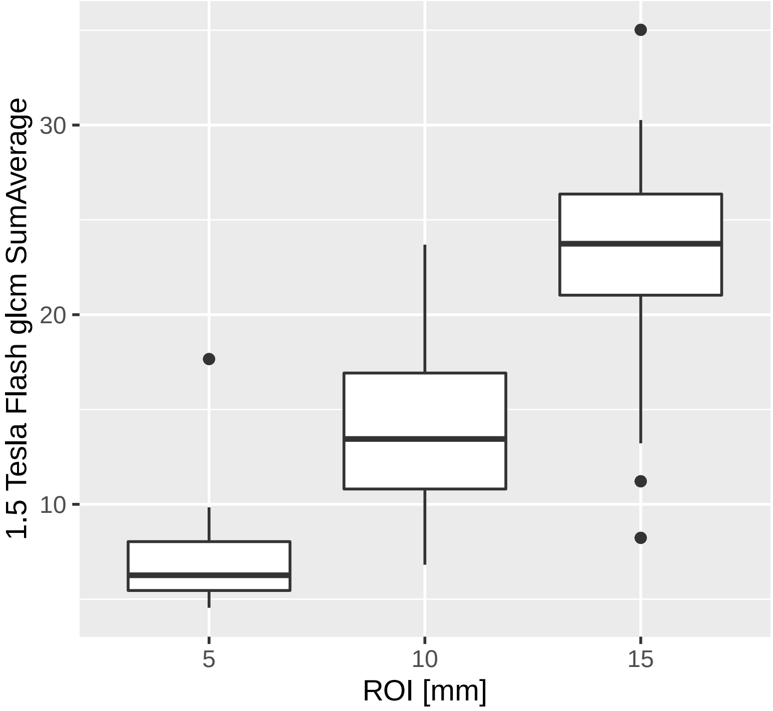
1.5 Tesla Flash glcm SumAverage
30
20
10
5
10
15
ROI [mm]

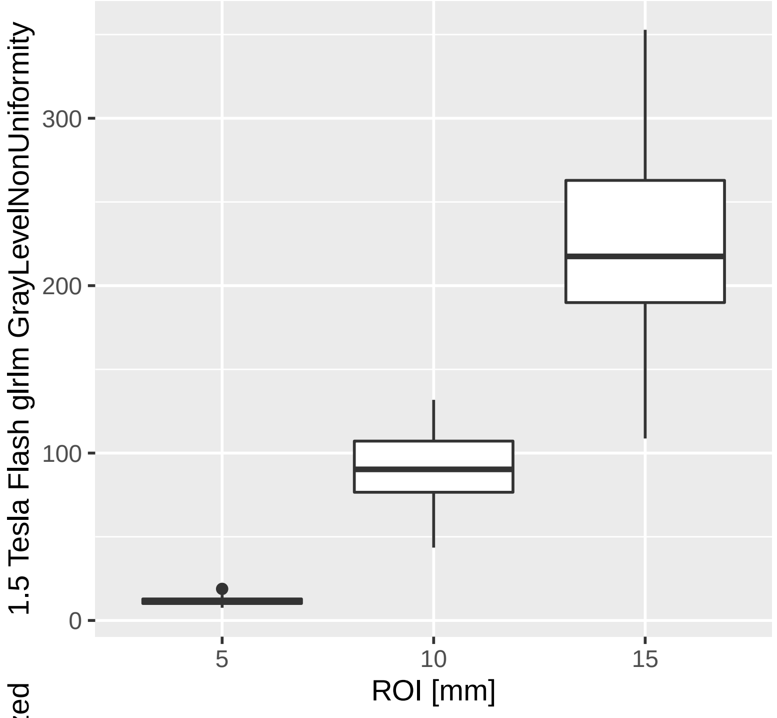
1.5 Tesla Flash glrlm GrayLevelNonUniformity
300
200
100
0
5
10
15
ROI [mm]

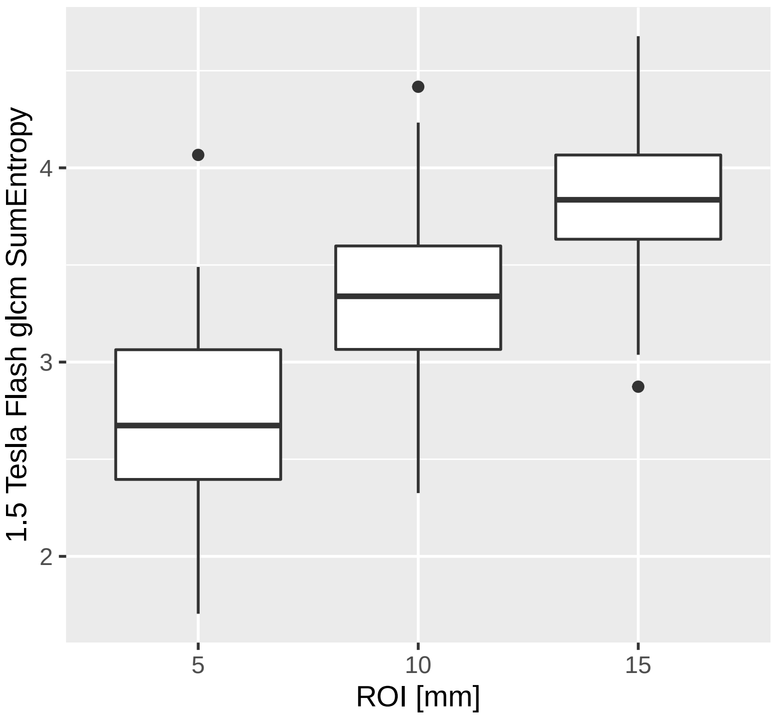
1.5 Tesla Flash glcm SumEntropy
4
3
2
5
10
15
ROI [mm]

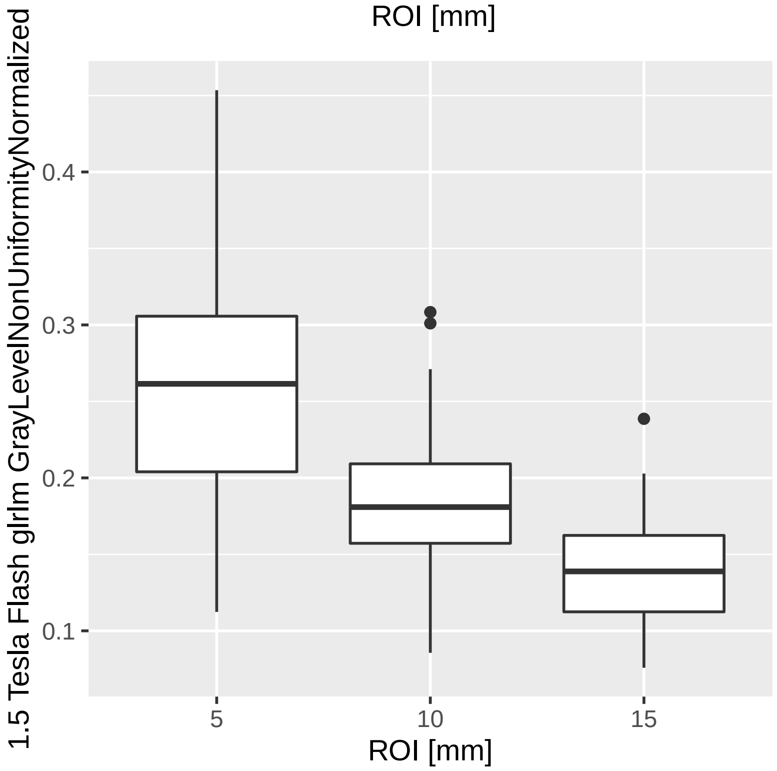
1.5 Tesla Flash glrlm GrayLevelNonUniformityNormalized
0.4
0.3
0.2
0.1
5
10
15
ROI [mm]

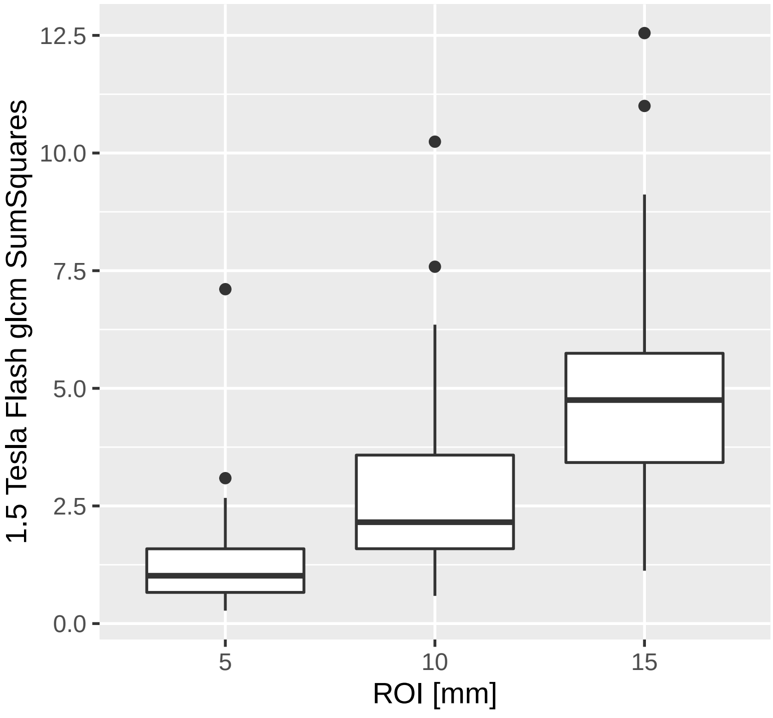
1.5 Tesla Flash glcm SumSquares
12.5
10.0
7.5
5.0
2.5
0.0
5
10
15
ROI [mm]

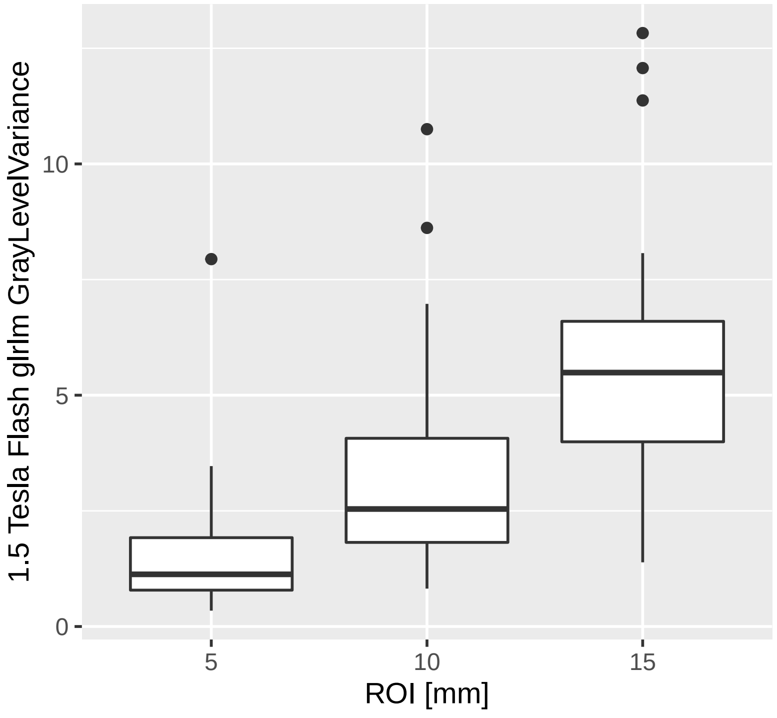
1.5 Tesla Flash glrlm GrayLevelVariance
10
5
0
5
10
15
ROI [mm]

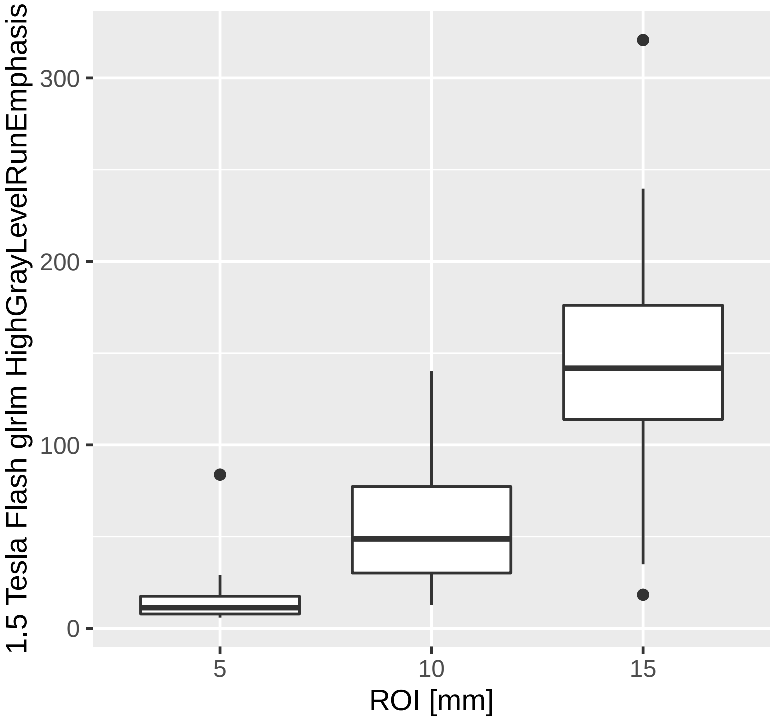
1.5 Tesla Flash glrlm HighGrayLevelRunEmphasis
0
100
200
300
5
10
15
ROI [mm]

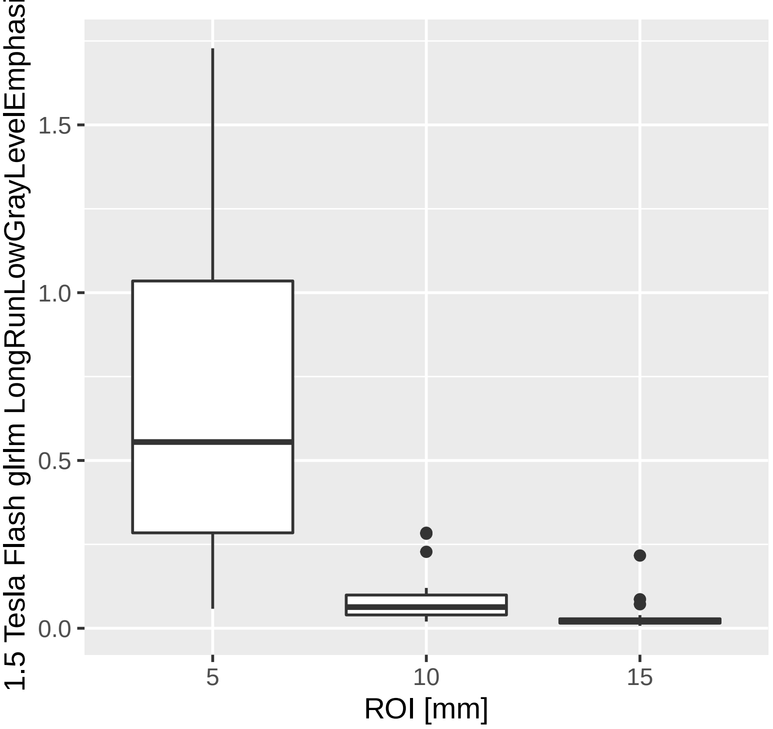
1.5 Tesla Flash glrlm LongRunLowGrayLevelEmphasis
0.0
0.5
1.0
1.5
5
10
15
ROI [mm]

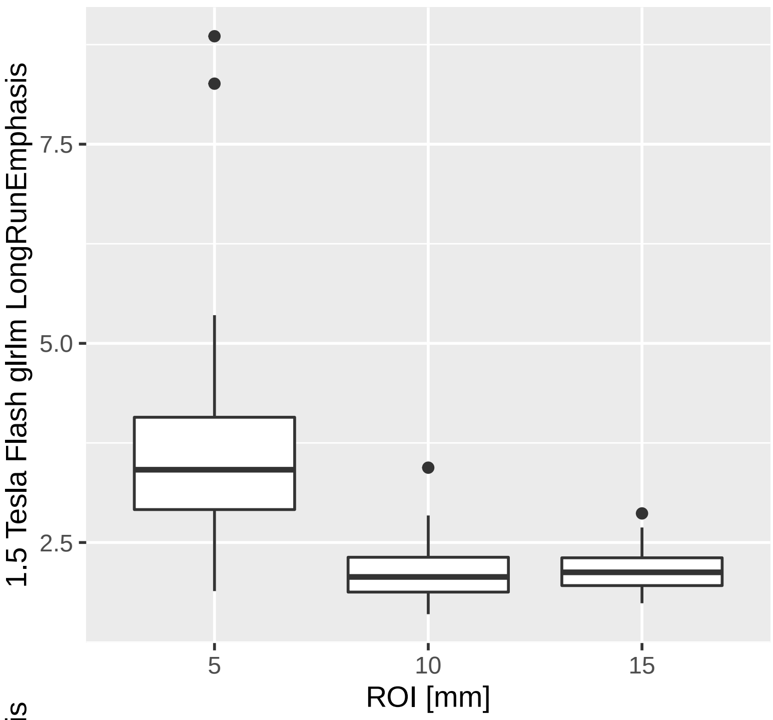
1.5 Tesla Flash glrlm LongRunEmphasis
2.5
5.0
7.5
5
10
15
ROI [mm]

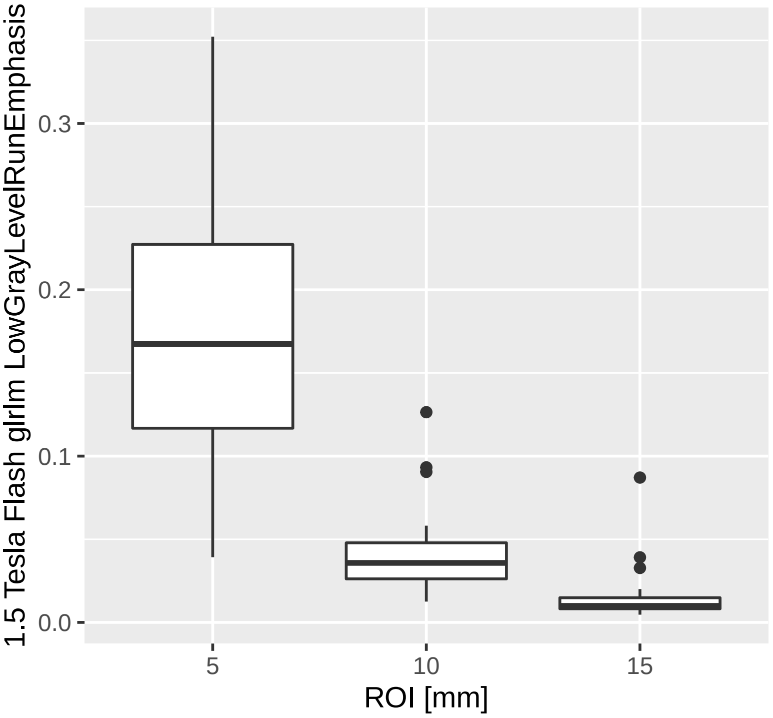
1.5 Tesla Flash glrlm LowGrayLevelRunEmphasis
0.0
0.1
0.2
0.3
5
10
15
ROI [mm]

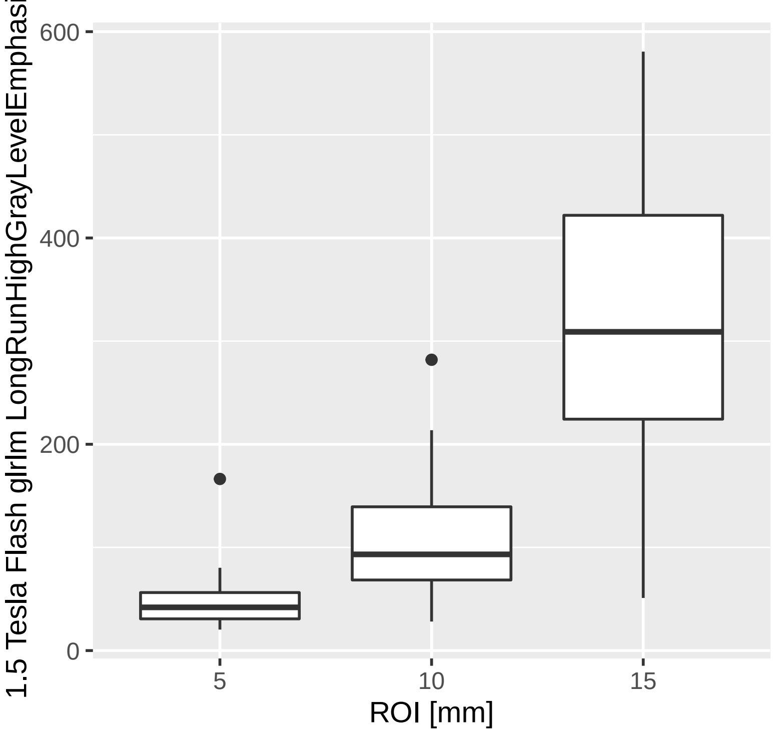
1.5 Tesla Flash glrlm LongRunHighGrayLevelEmphasis
0
200
400
600
5
10
15
ROI [mm]

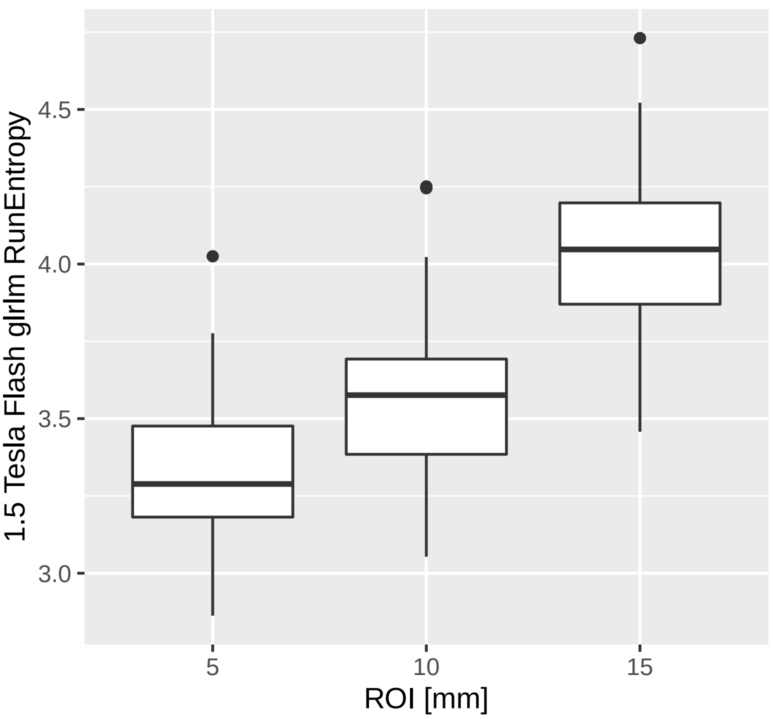
1.5 Tesla Flash glrlm RunEntropy
3.0
3.5
4.0
4.5
5
10
15
ROI [mm]

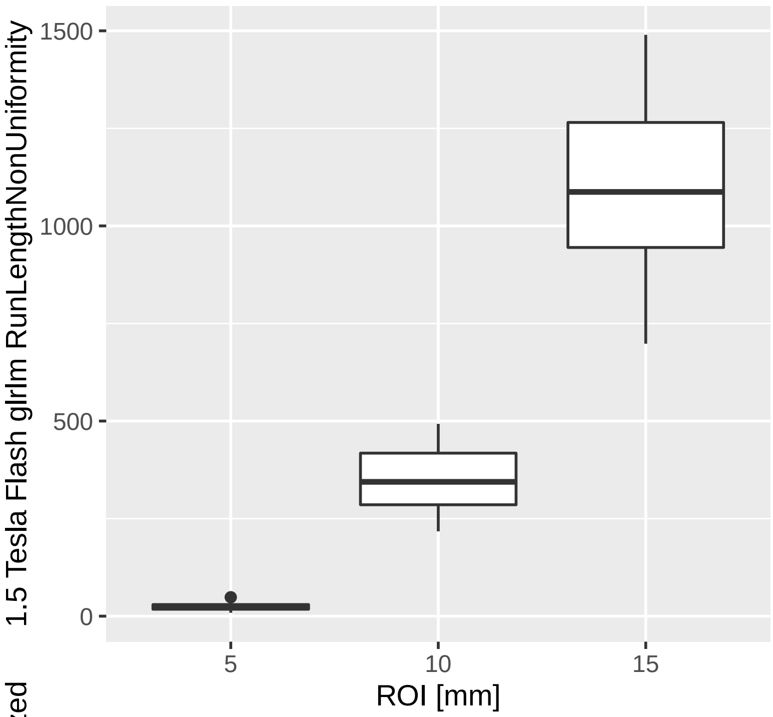
1.5 Tesla Flash glrlm RunLengthNonUniformity
1500
1000
500
0
5
10
15
ROI [mm]

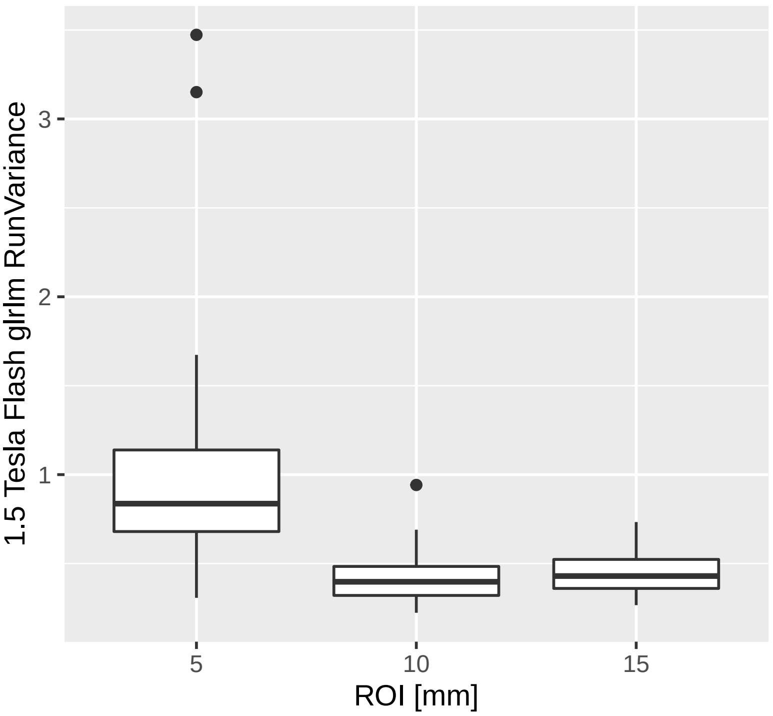
1.5 Tesla Flash glrlm RunVariance
3
2
1
5
10
15
ROI [mm]

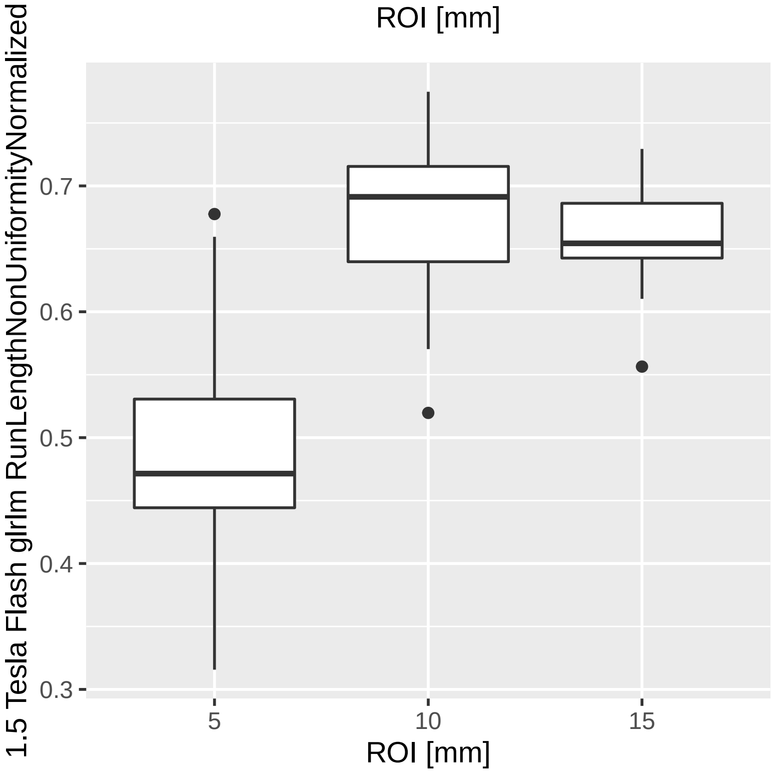
1.5 Tesla Flash glrlm RunLengthNonUniformityNormalized
0.7
0.6
0.5
0.4
0.3
5
10
15
ROI [mm]

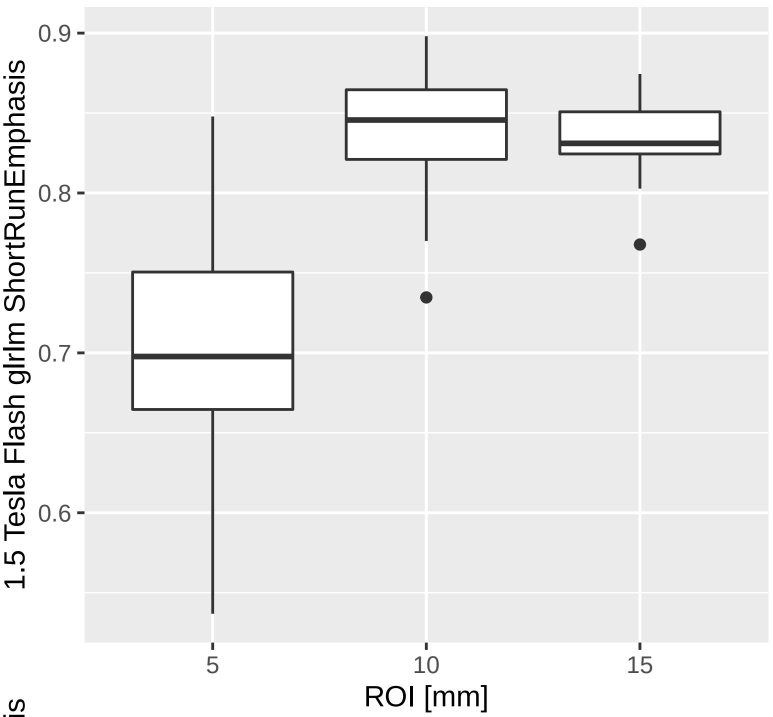
1.5 Tesla Flash glrlm ShortRunEmphasis
0.9
0.8
0.7
0.6
5
10
15
ROI [mm]

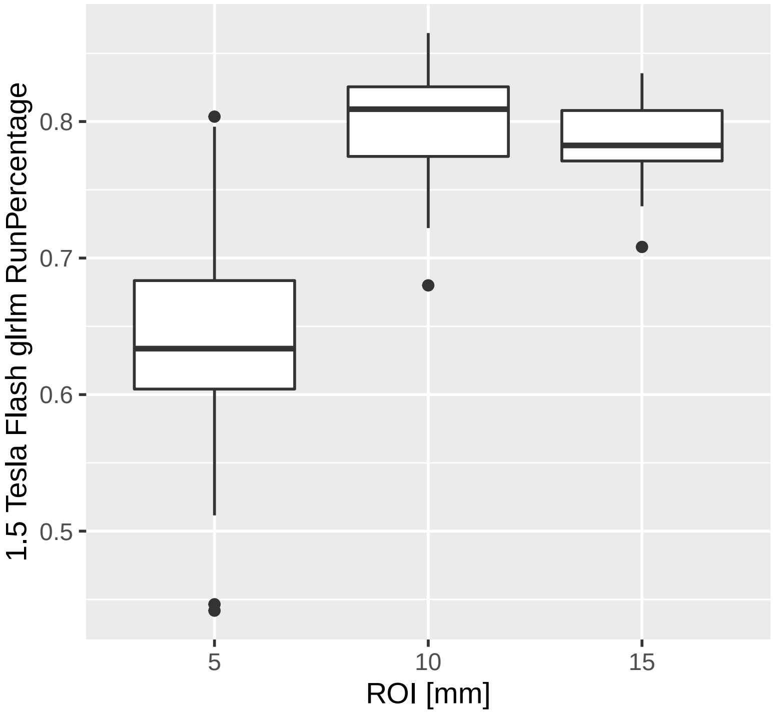
1.5 Tesla Flash glrlm RunPercentage
0.8
0.7
0.6
0.5
5
10
15
ROI [mm]

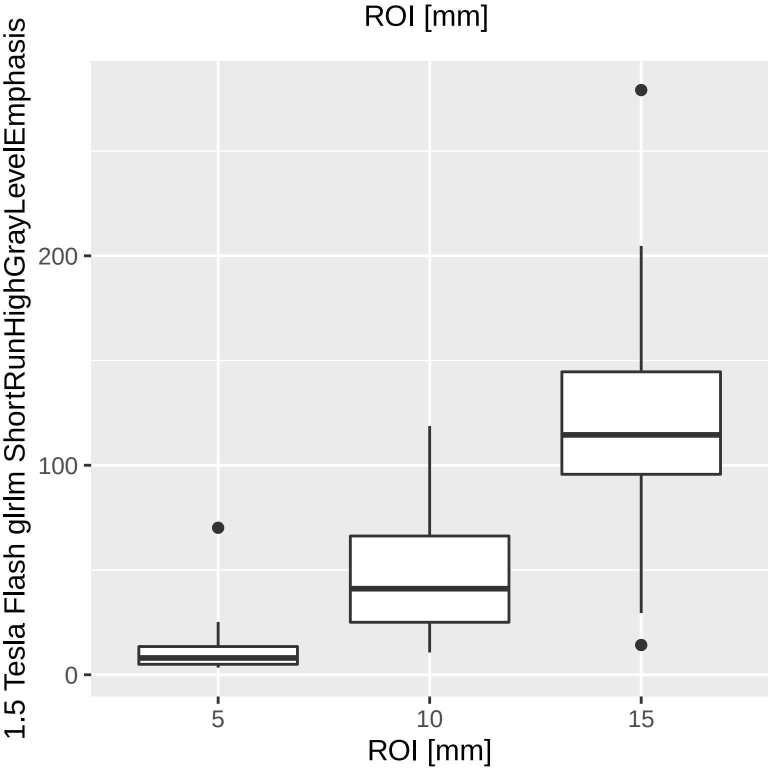
1.5 Tesla Flash glrlm ShortRunHighGrayLevelEmphasis
200
100
0
5
10
15
ROI [mm]

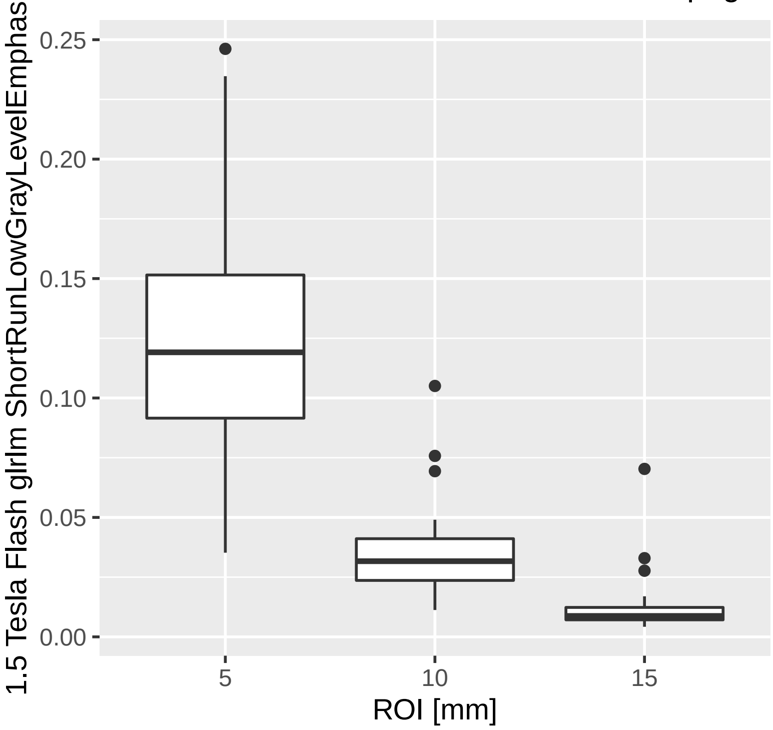
1.5 Tesla Flash glrlm ShortRunLowGrayLevelEmphasis
0.25
0.20
0.15
0.10
0.05
0.00
5
10
15
ROI [mm]

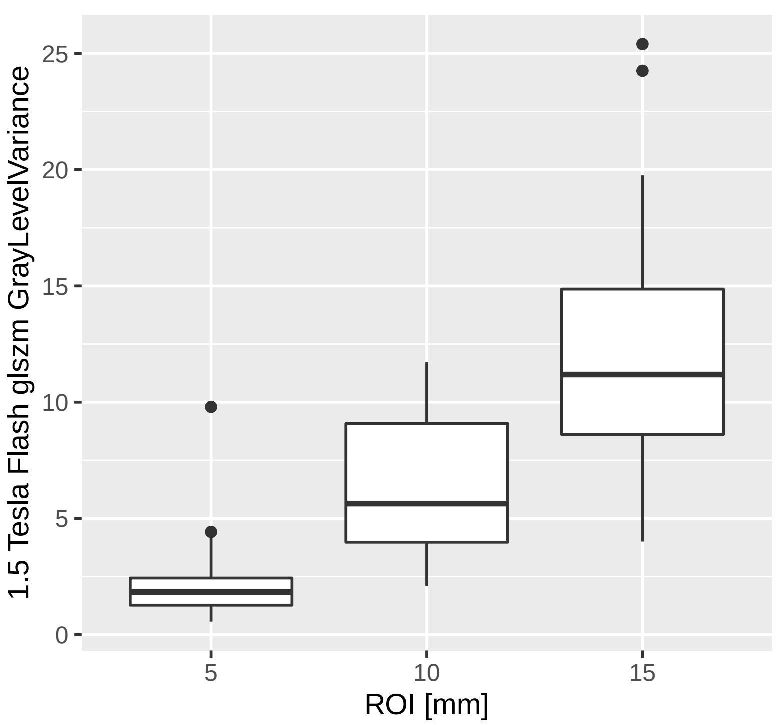
1.5 Tesla Flash glszm GrayLevelVariance
25
20
15
10
5
0
5
10
15
ROI [mm]

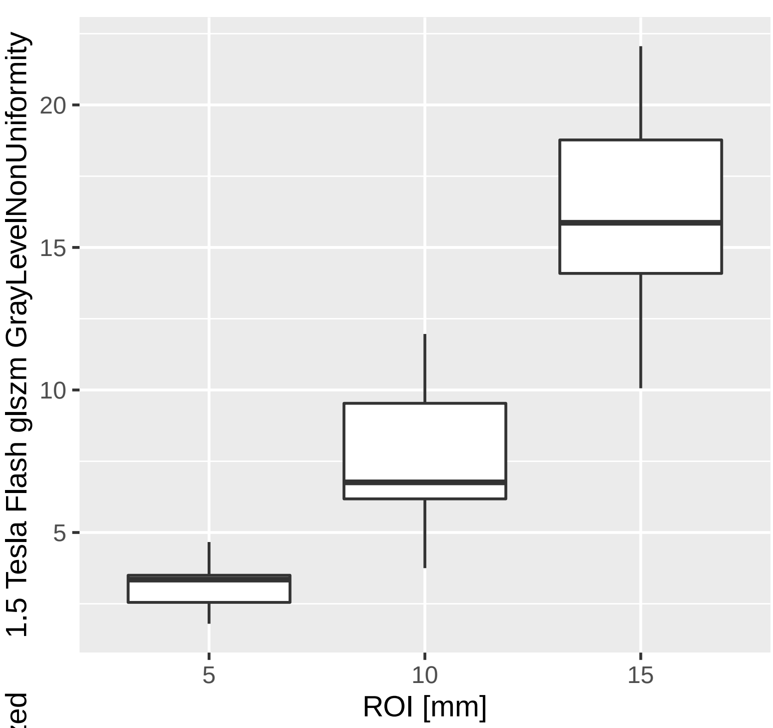
1.5 Tesla Flash glszm GrayLevelNonUniformity
20
15
10
5
5
10
15
ROI [mm]

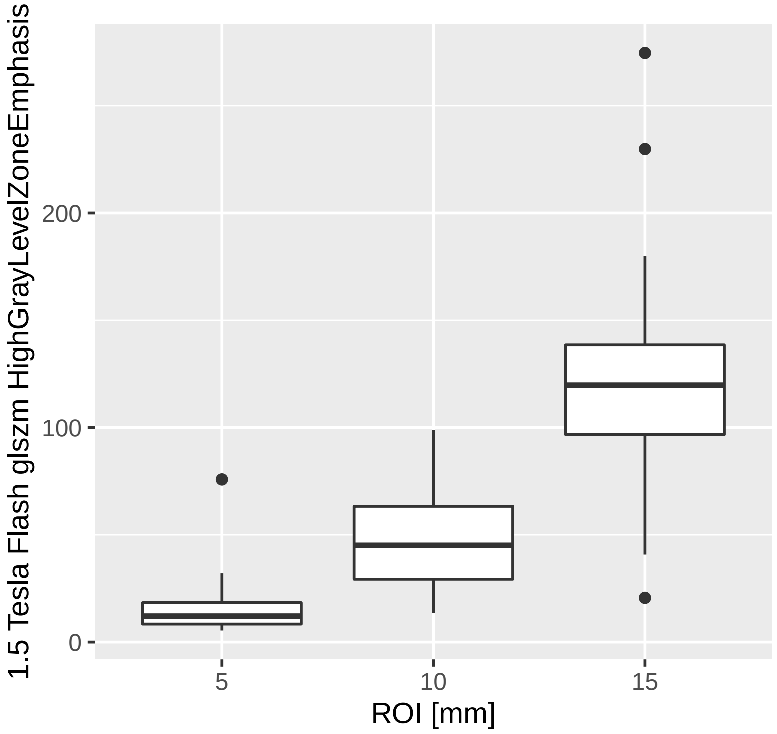
1.5 Tesla Flash glszm HighGrayLevelZoneEmphasis
200
100
0
5
10
15
ROI [mm]

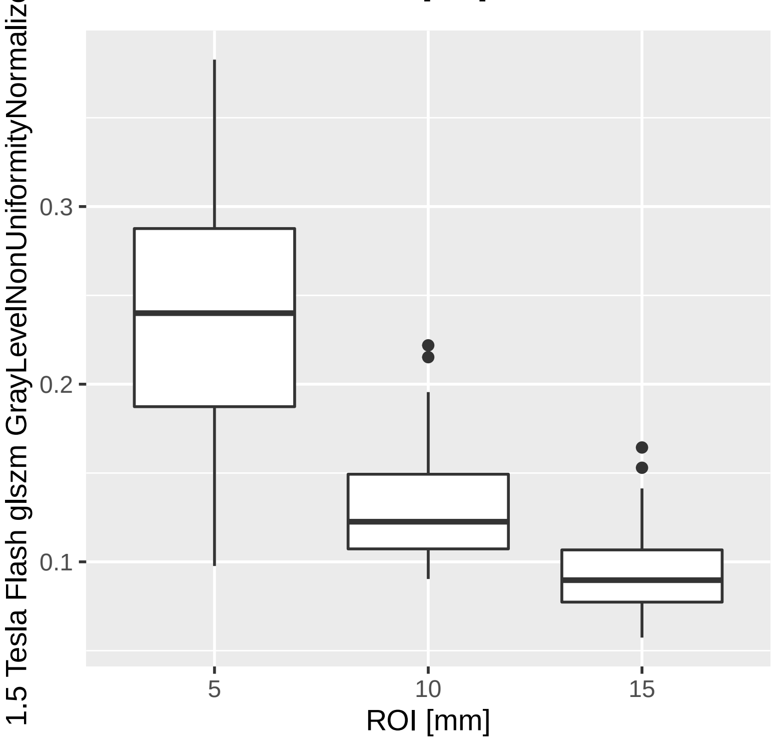
1.5 Tesla Flash glszm GrayLevelNonUniformityNormalized
0.3
0.2
0.1
5
10
15
ROI [mm]

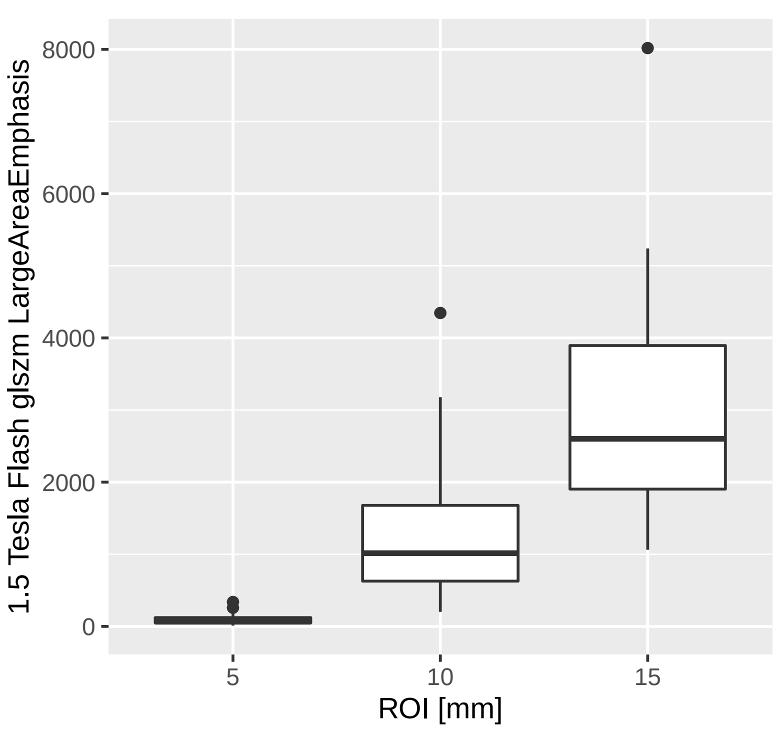
1.5 Tesla Flash glszm LargeAreaEmphasis
8000
6000
4000
2000
0
5
10
15
ROI [mm]

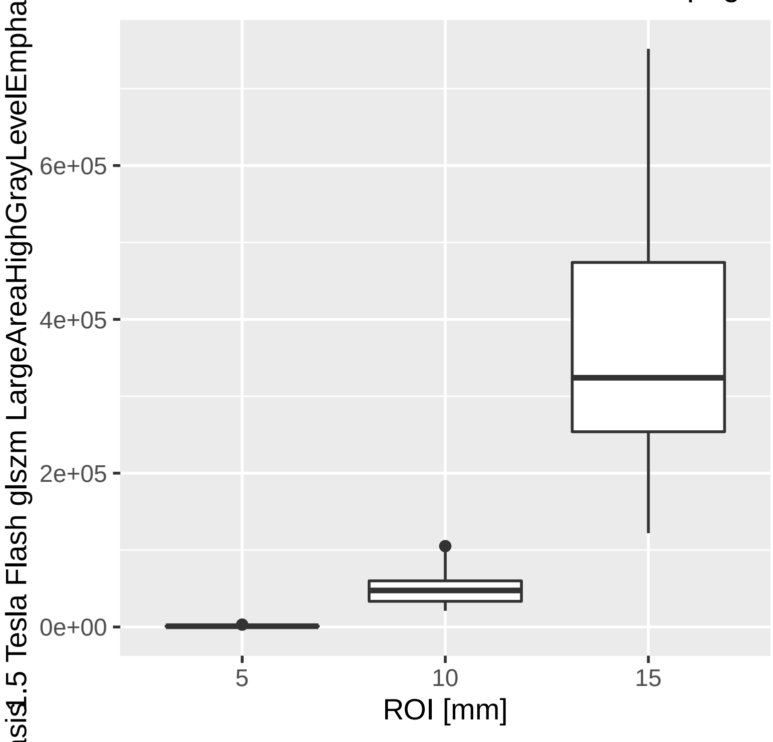
1.5 Tesla Flash glszm LargeAreaHighGrayLevelEmphasis
0e+00
2e+05
4e+05
6e+05
5
10
15
ROI [mm]

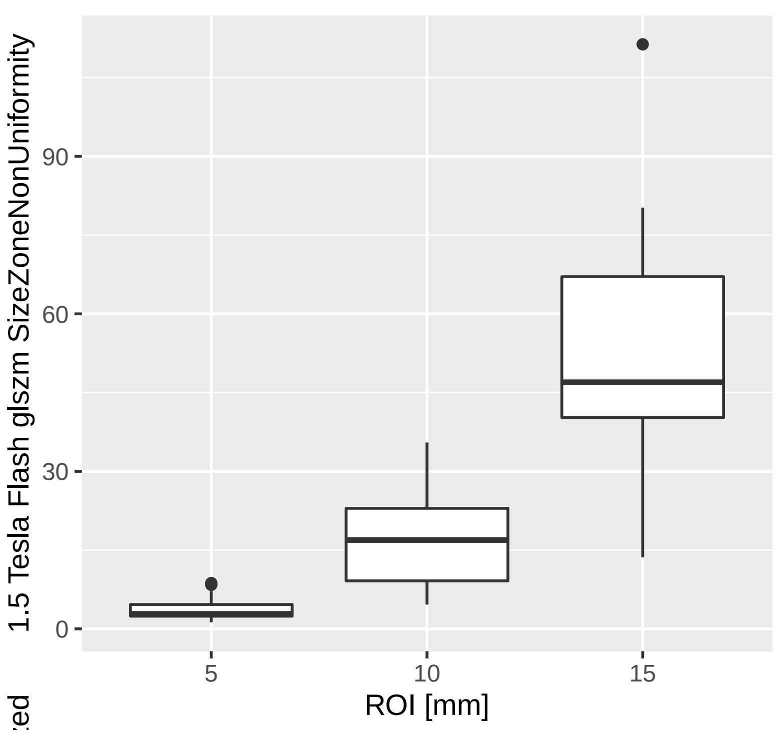
1.5 Tesla Flash glszm SizeZoneNonUniformity
0
30
60
90
5
10
15
ROI [mm]

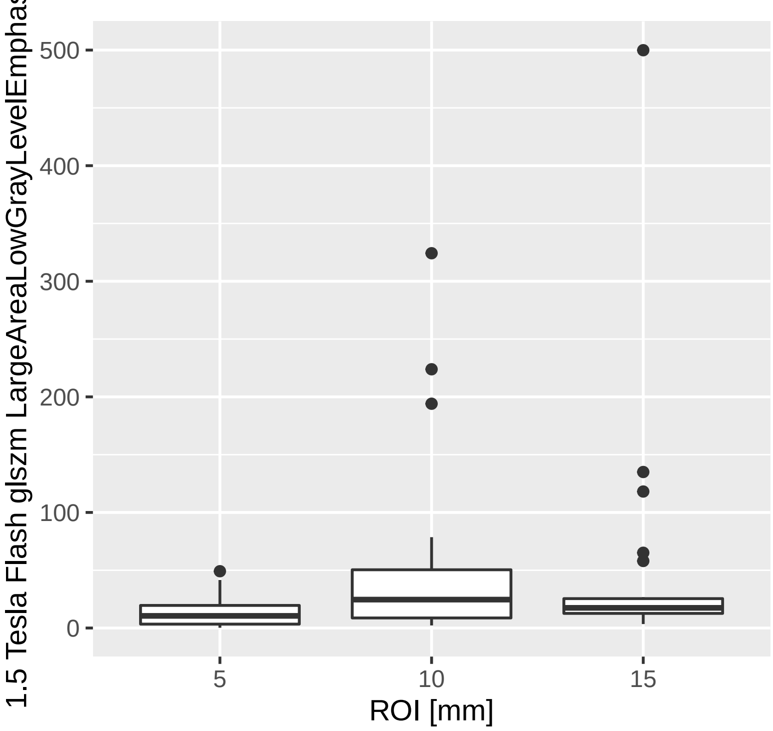
1.5 Tesla Flash glszm LargeAreaLowGrayLevelEmphasis
0
100
200
300
400
500
5
10
15
ROI [mm]

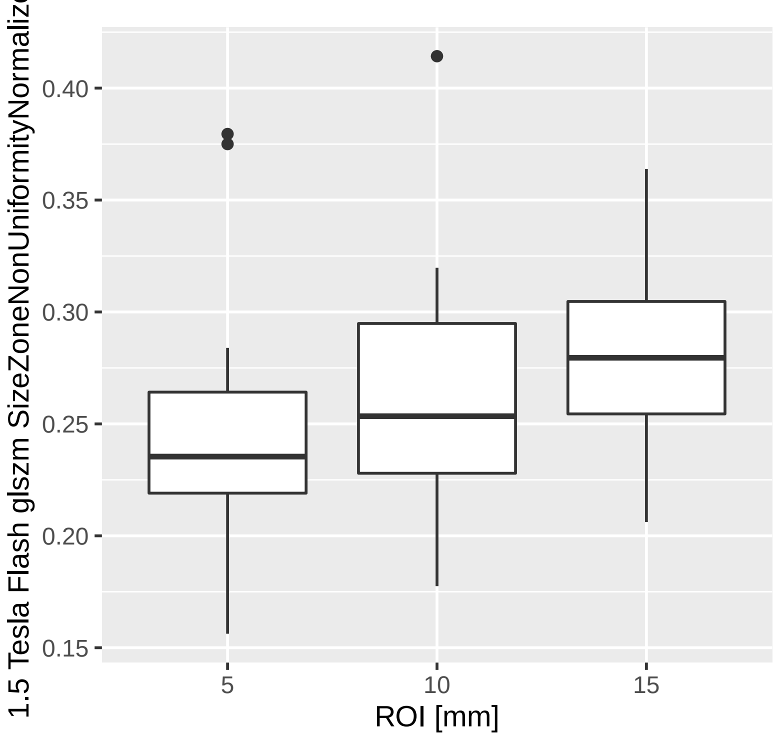
1.5 Tesla Flash glszm SizeZoneNonUniformityNormalized
0.15
0.20
0.25
0.30
0.35
0.40
5
10
15
ROI [mm]

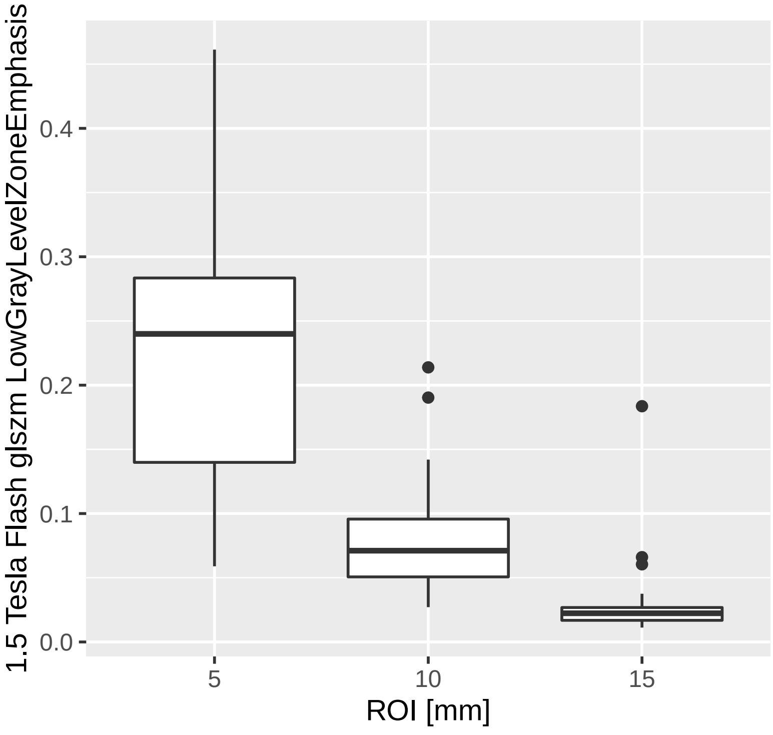
1.5 Tesla Flash glszm LowGrayLevelZoneEmphasis
0.0
0.1
0.2
0.3
0.4
5
10
15
ROI [mm]

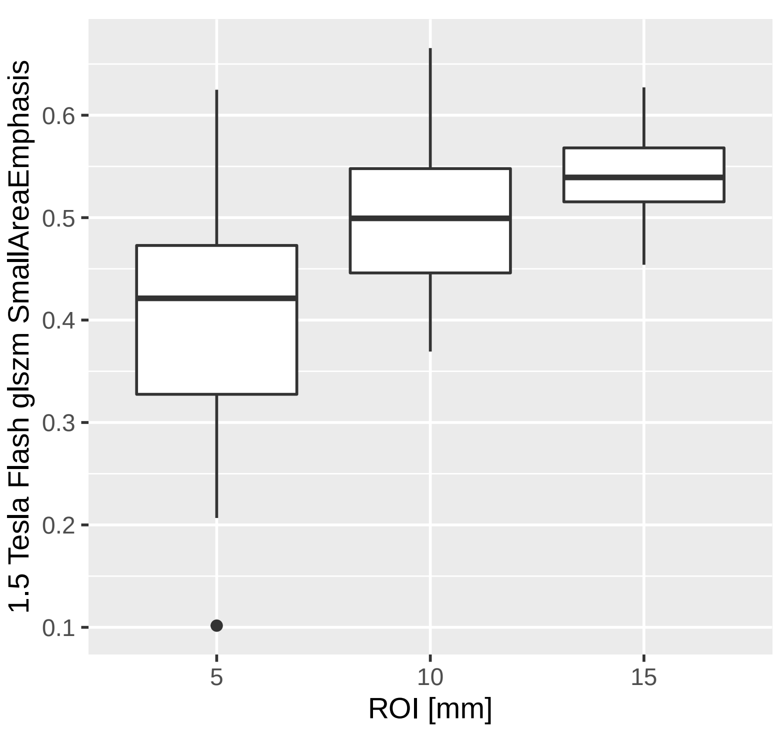
1.5 Tesla Flash glszm SmallAreaEmphasis
0.1
0.2
0.3
0.4
0.5
0.6
5
10
15
ROI [mm]

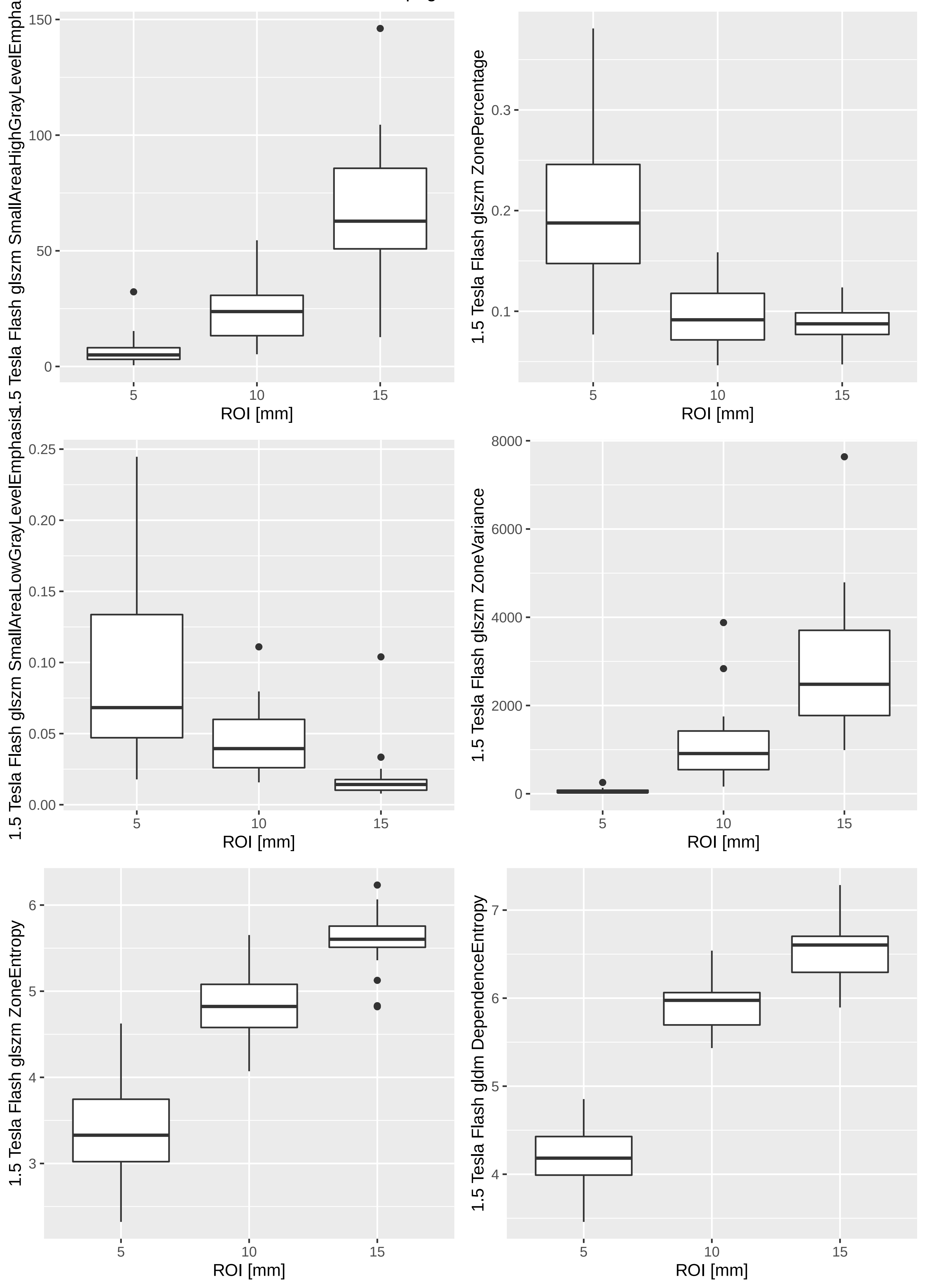
1.5 Tesla Flash glszm SmallAreaHighGrayLevelEmphasis
150
100
50
0
5
10
15
ROI [mm]
1.5 Tesla Flash glszm ZonePercentage
0.3
0.2
0.1
5
10
15
ROI [mm]
1.5 Tesla Flash glszm SmallAreaLowGrayLevelEmphasis
0.25
0.20
0.15
0.10
0.05
0.00
5
10
15
ROI [mm]
1.5 Tesla Flash glszm ZoneVariance
8000
6000
4000
2000
0
5
10
15
ROI [mm]
1.5 Tesla Flash glszm ZoneEntropy
6
5
4
3
5
10
15
ROI [mm]
1.5 Tesla Flash gldm DependenceEntropy
7
6
5
4
5
10
15
ROI [mm]

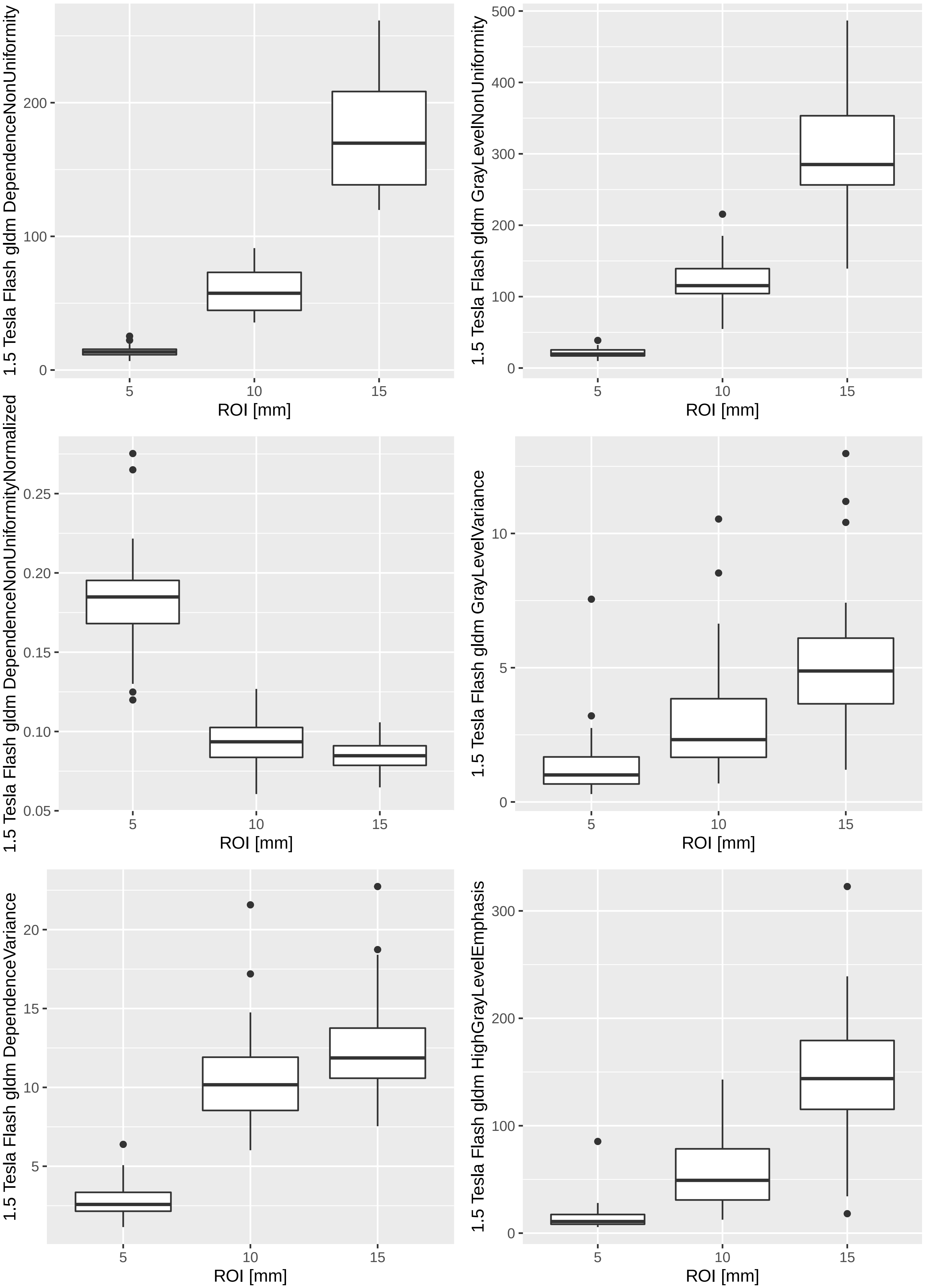
1.5 Tesla Flash gldm DependenceNonUniformity
0
100
200
5
10
15
ROI [mm]
1.5 Tesla Flash gldm GrayLevelNonUniformity
0
100
200
300
400
500
ROI [mm]
1.5 Tesla Flash gldm DependenceNonUniformityNormalized
0.05
0.10
0.15
0.20
0.25
ROI [mm]
1.5 Tesla Flash gldm GrayLevelVariance
0
5
10
ROI [mm]
1.5 Tesla Flash gldm DependenceVariance
5
10
15
20
ROI [mm]
1.5 Tesla Flash gldm HighGrayLevelEmphasis
0
100
200
300
ROI [mm]

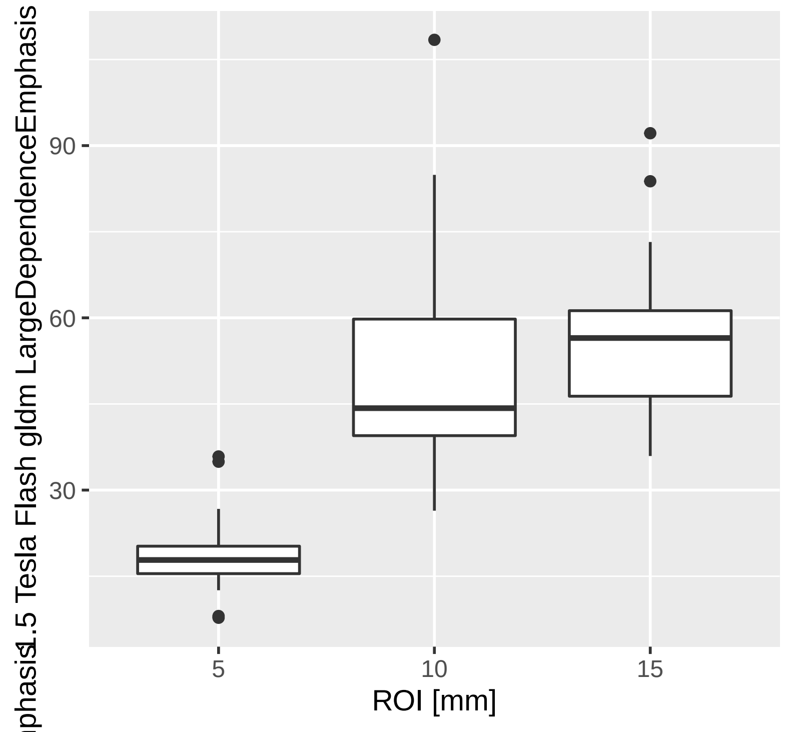
1.5 Tesla Flash gldm LargeDependenceEmphasis
90
60
30
5
10
15
ROI [mm]

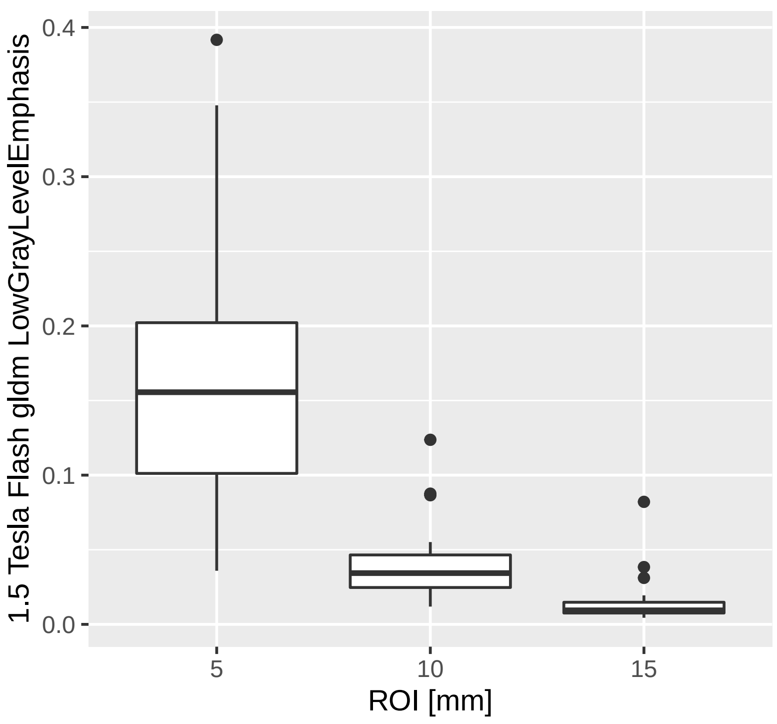
1.5 Tesla Flash gldm LowGrayLevelEmphasis
0.4
0.3
0.2
0.1
0.0
5
10
15
ROI [mm]

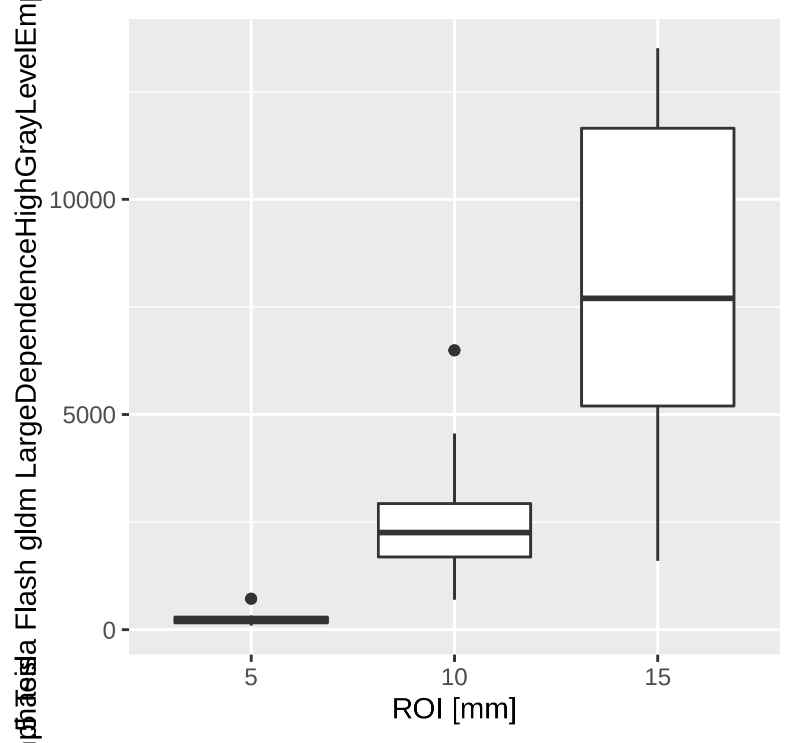
1.5 Tesla Flash gldm LargeDependenceHighGrayLevelEmphasis
10000
5000
0
5
10
15
ROI [mm]

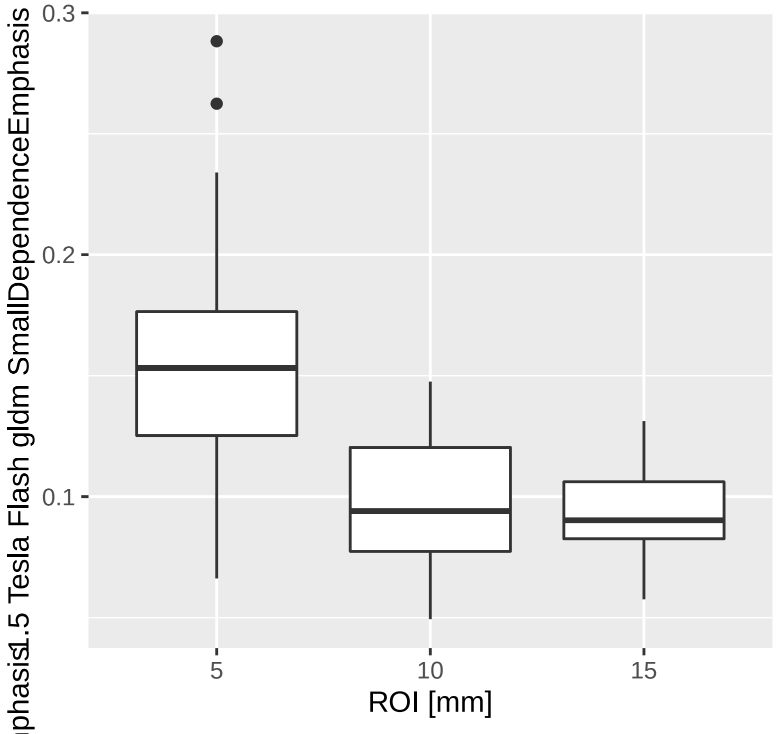
1.5 Tesla Flash gldm SmallDependenceEmphasis
0.3
0.2
0.1
5
10
15
ROI [mm]

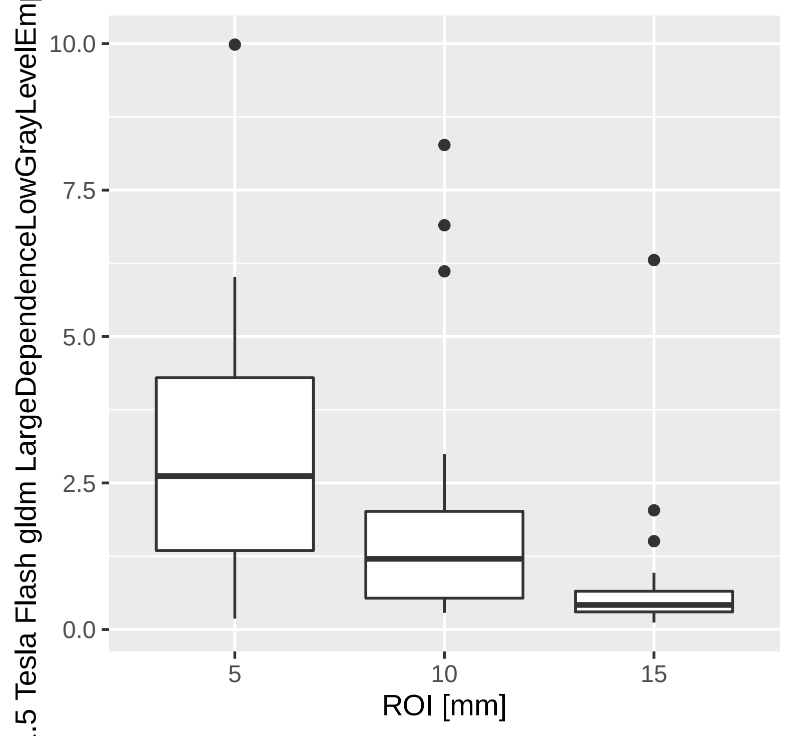
.5 Tesla Flash gldm LargeDependenceLowGrayLevelEmphasis
10.0
7.5
5.0
2.5
0.0
5
10
15
ROI [mm]

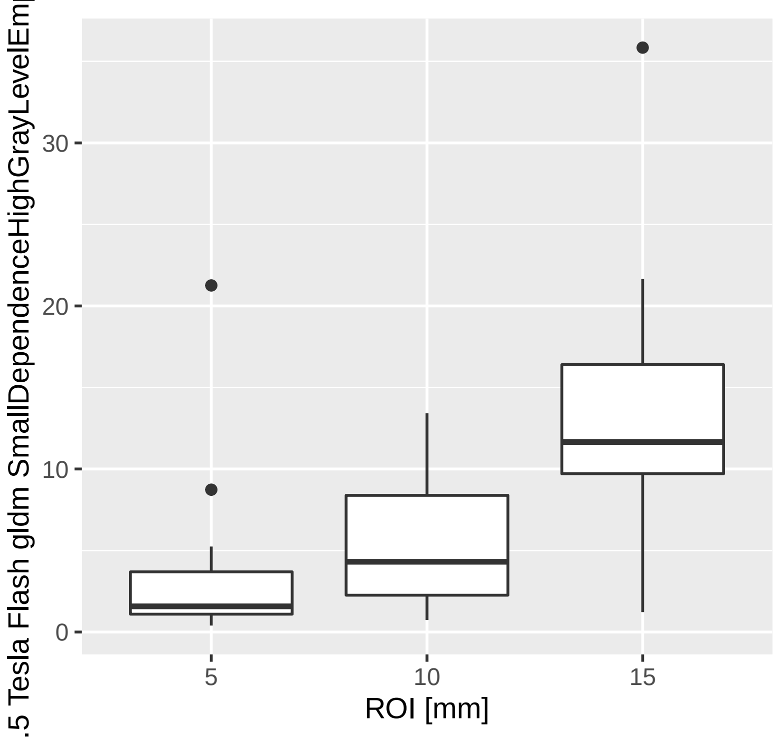
.5 Tesla Flash gldm SmallDependenceHighGrayLevelEmphasis
30
20
10
0
5
10
15
ROI [mm]

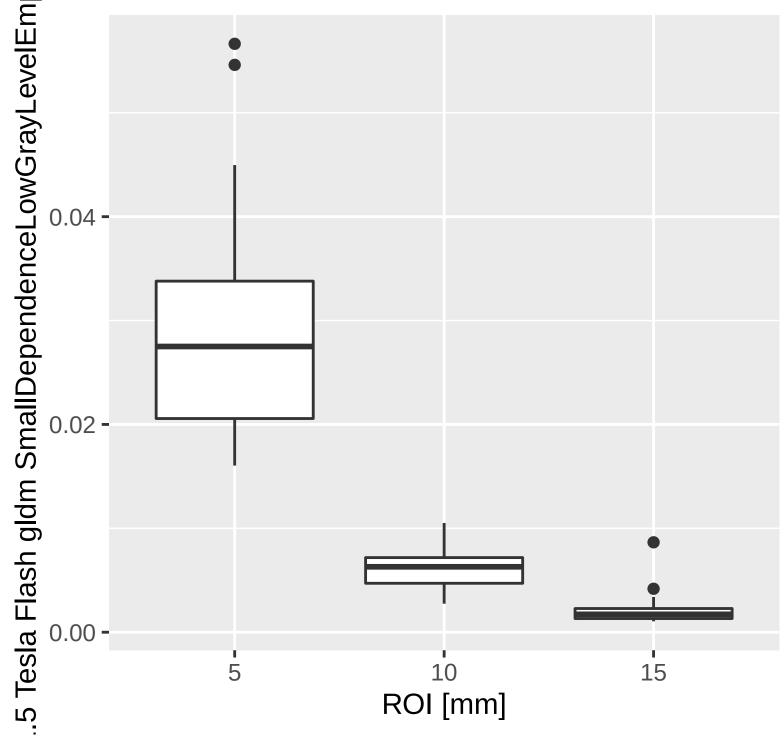
.5 Tesla Flash gldm SmallDependenceLowGrayLevelEmpha
0.00
0.02
0.04
5
10
15
ROI [mm]

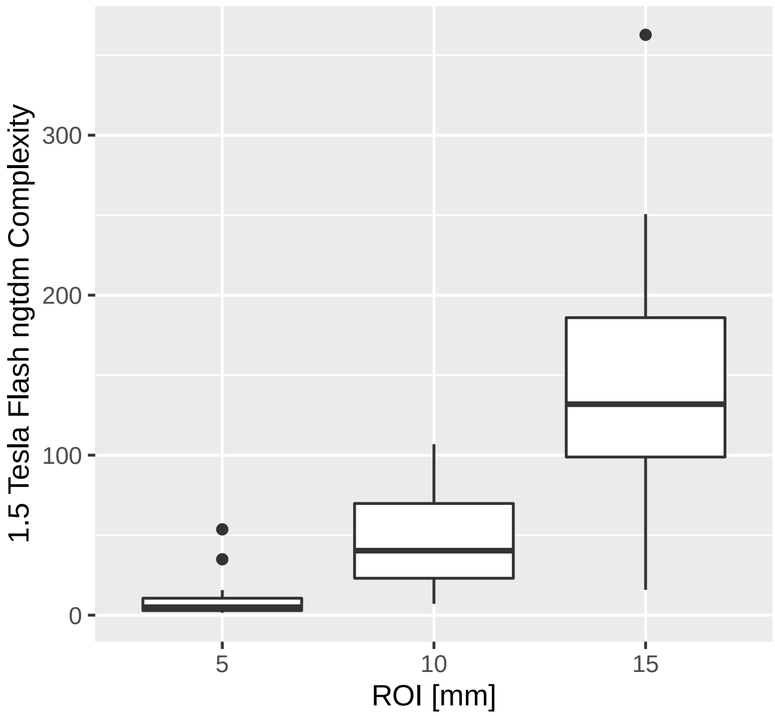
1.5 Tesla Flash ngtdm Complexity
0
100
200
300
5
10
15
ROI [mm]

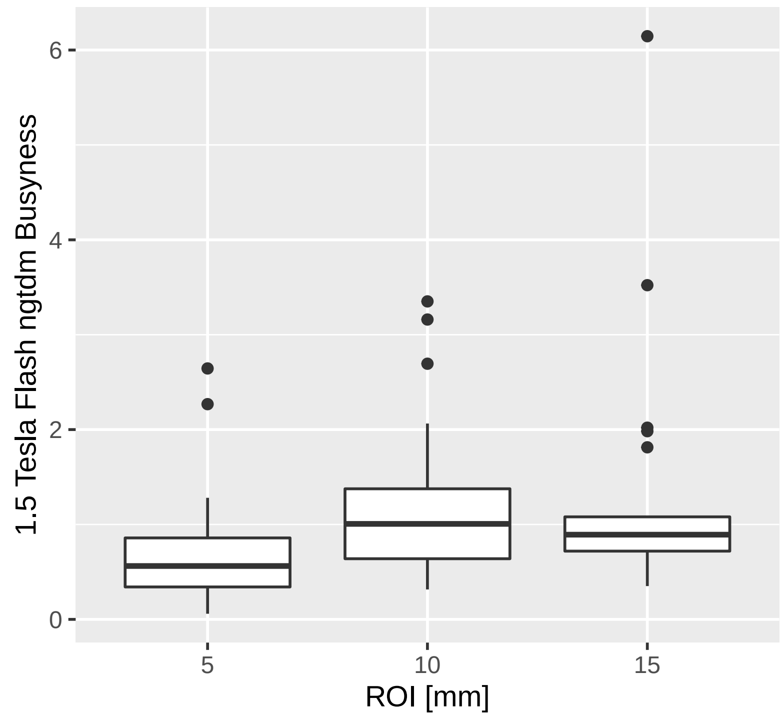
1.5 Tesla Flash ngtdm Busyness
0
2
4
6
5
10
15
ROI [mm]

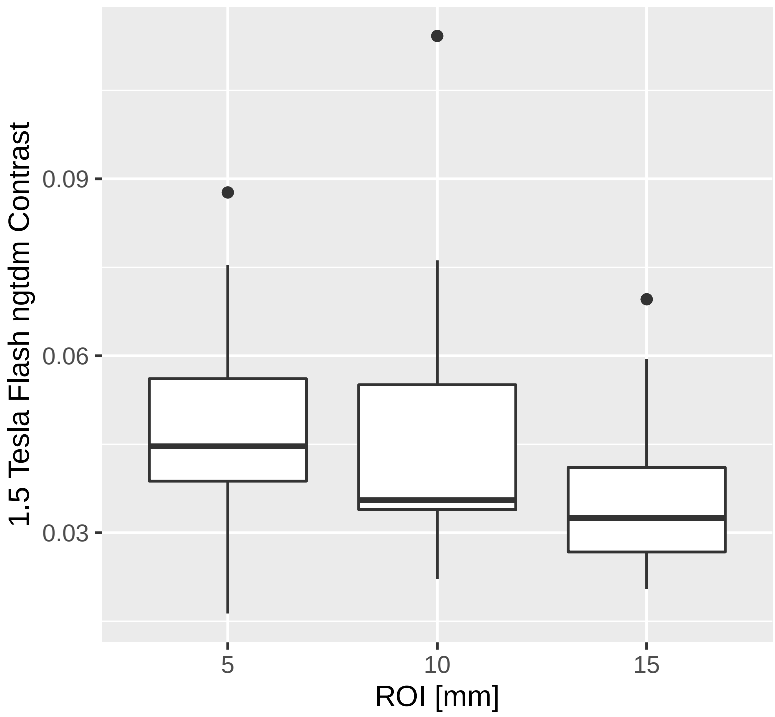
1.5 Tesla Flash ngtdm Contrast
0.03
0.06
0.09
5
10
15
ROI [mm]

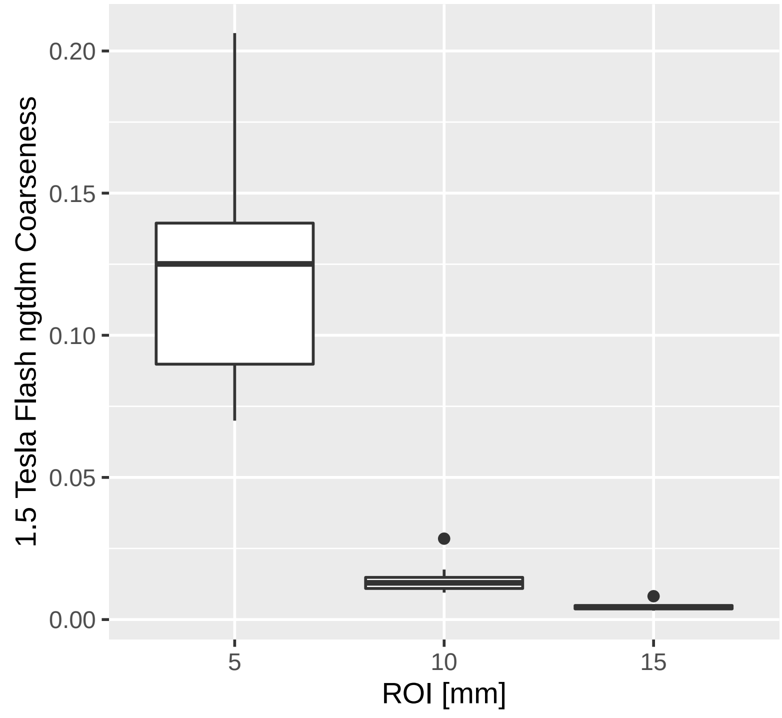
1.5 Tesla Flash ngtdm Coarseness
0.00
0.05
0.10
0.15
0.20
5
10
15
ROI [mm]

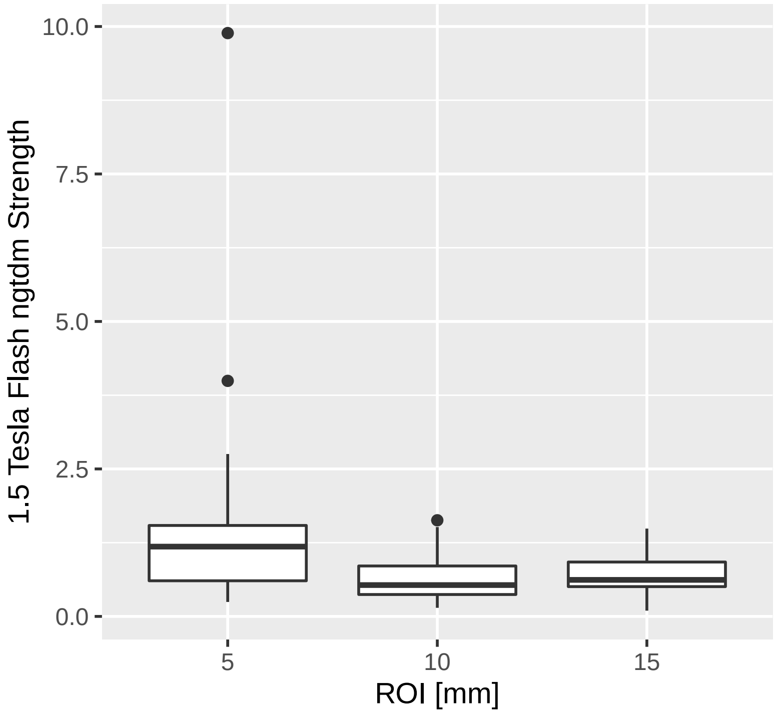
1.5 Tesla Flash ngtdm Strength
0.0
2.5
5.0
7.5
10.0
5
10
15
ROI [mm]

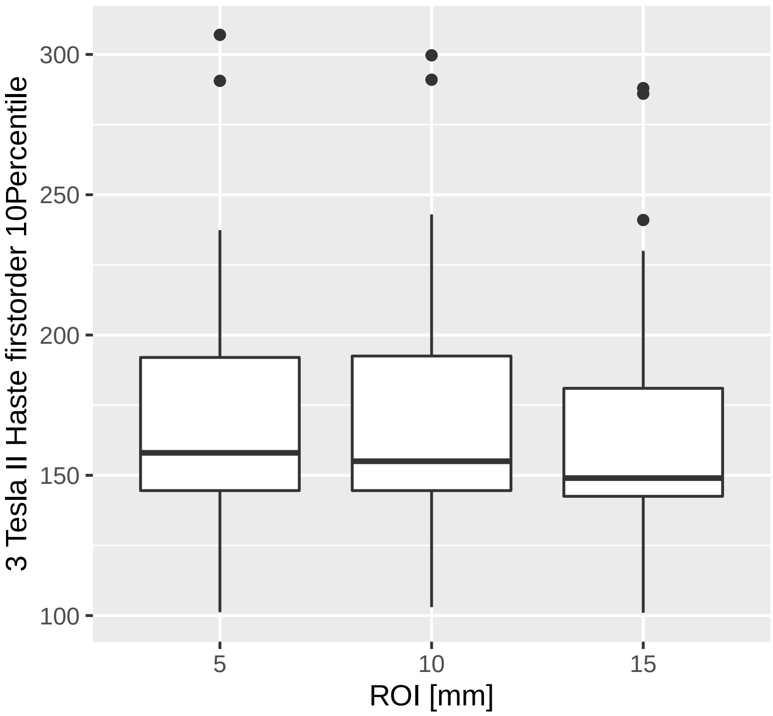

3 Tesla II Haste firstorder 10Percentile
300
250
200
150
100
5
10
15
ROI [mm]

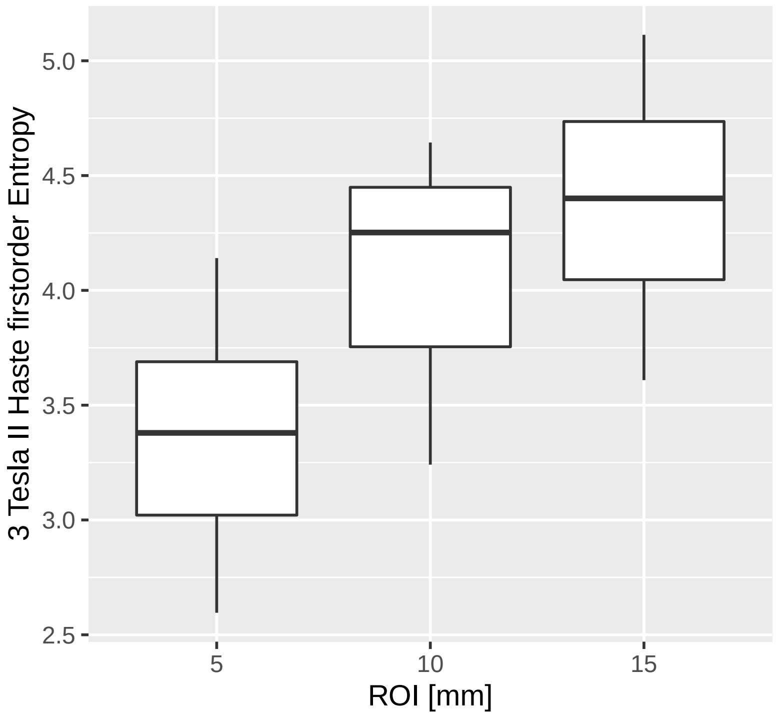

3 Tesla II Haste firstorder Entropy
5.0
4.5
4.0
3.5
3.0
2.5
5
10
15
ROI [mm]

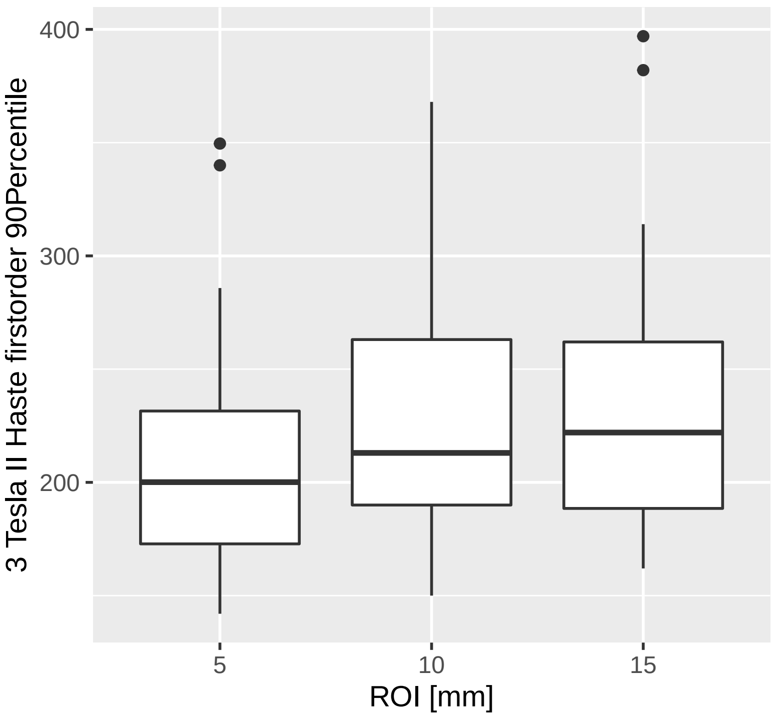

3 Tesla II Haste firstorder 90Percentile
400
300
200
5
10
15
ROI [mm]

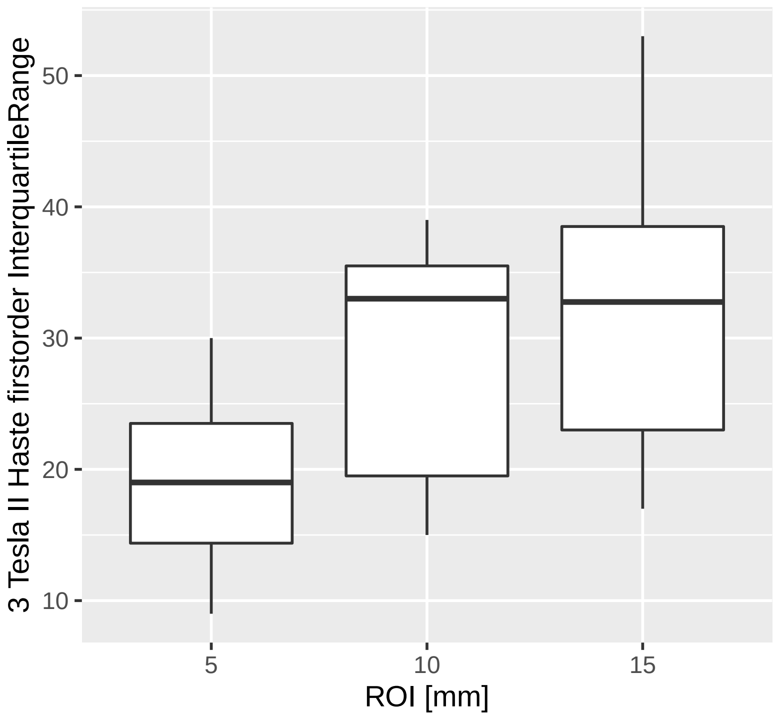

3 Tesla II Haste firstorder InterquartileRange
50
40
30
20
10
5
10
15
ROI [mm]

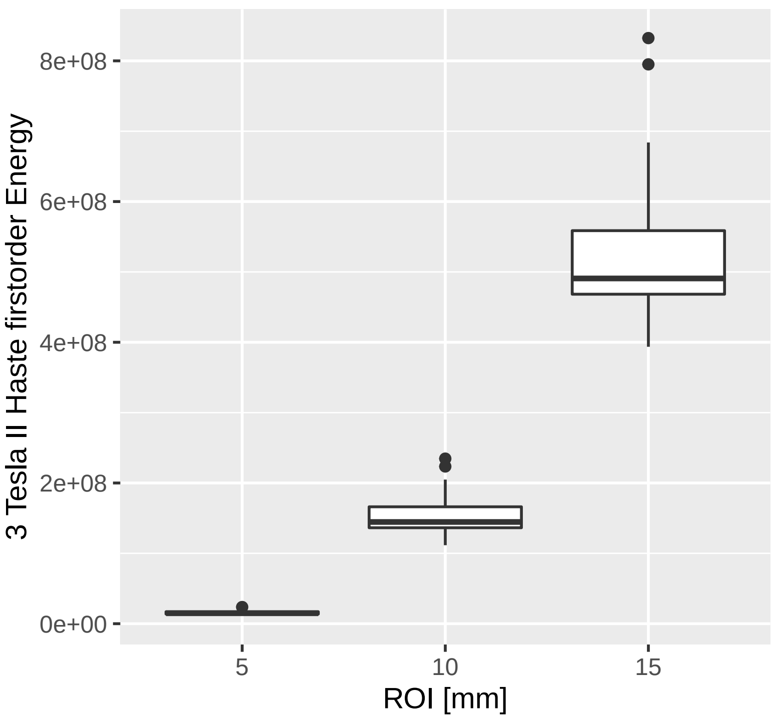

3 Tesla II Haste firstorder Energy
8e+08
6e+08
4e+08
2e+08
0e+00
5
10
15
ROI [mm]

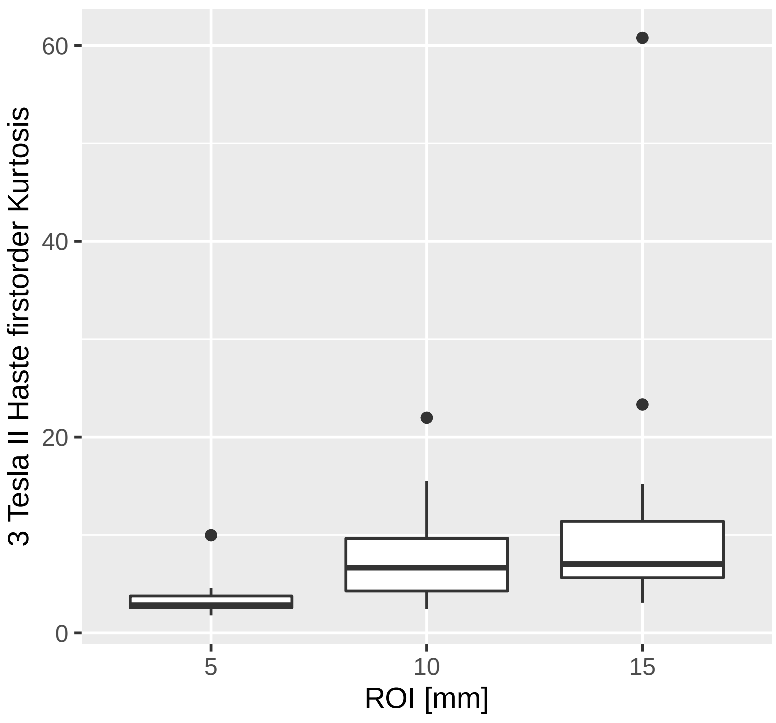

3 Tesla II Haste firstorder Kurtosis
60
40
20
0
5
10
15
ROI [mm]

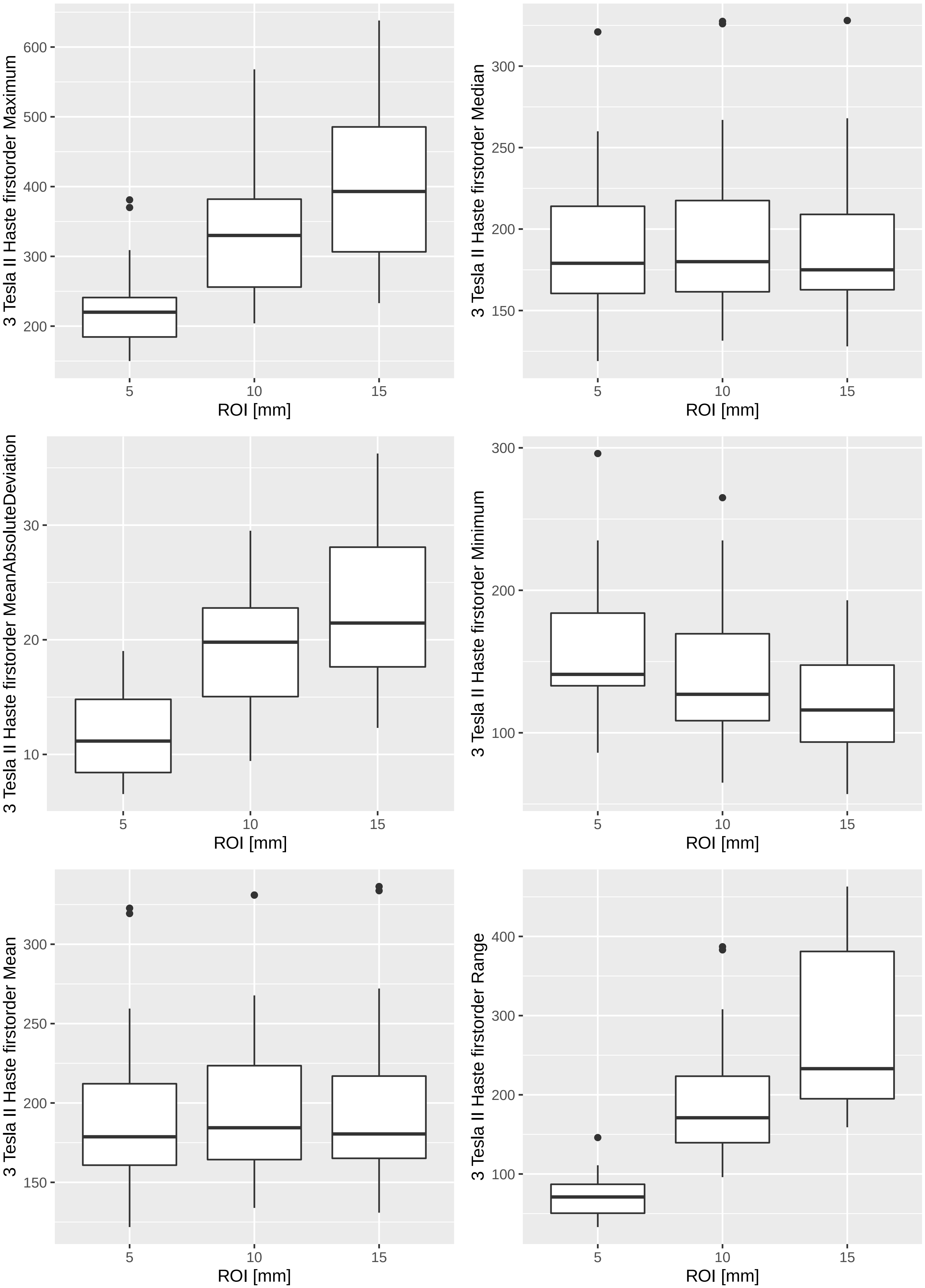
3 Tesla II Haste firstorder Maximum
3 Tesla II Haste firstorder Median
3 Tesla II Haste firstorder MeanAbsoluteDeviation
3 Tesla II Haste firstorder Minimum
3 Tesla II Haste firstorder Mean
3 Tesla II Haste firstorder Range
ROI [mm]
5
10
15

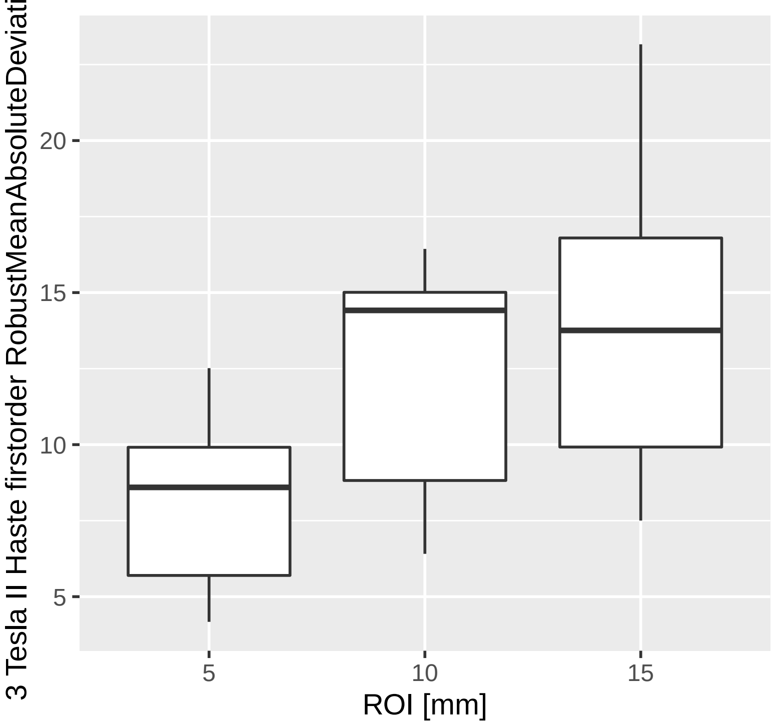
3 Tesla II Haste firstorder RobustMeanAbsoluteDeviation
5
10
15
20
ROI [mm]

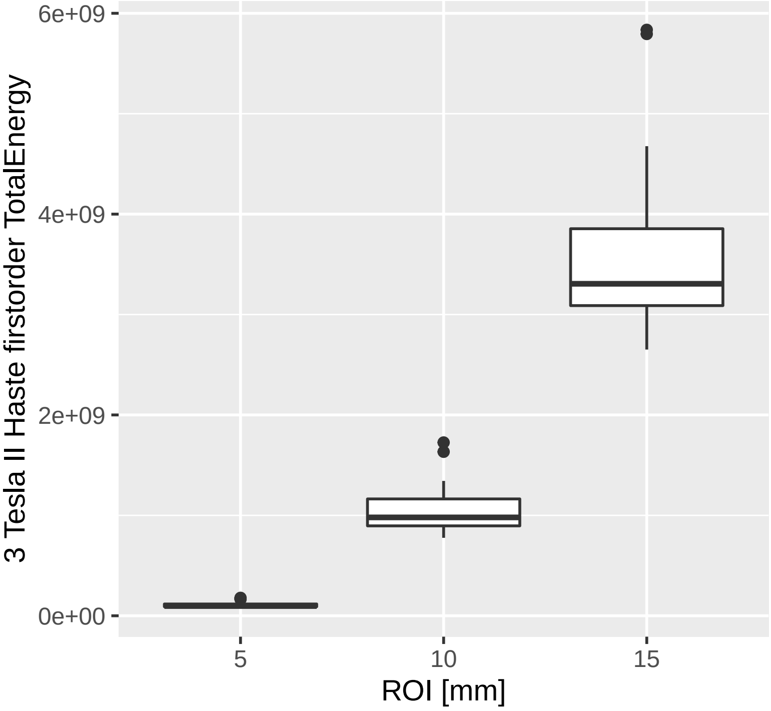
3 Tesla II Haste firstorder TotalEnergy
0e+00
2e+09
4e+09
6e+09
5
10
15
ROI [mm]

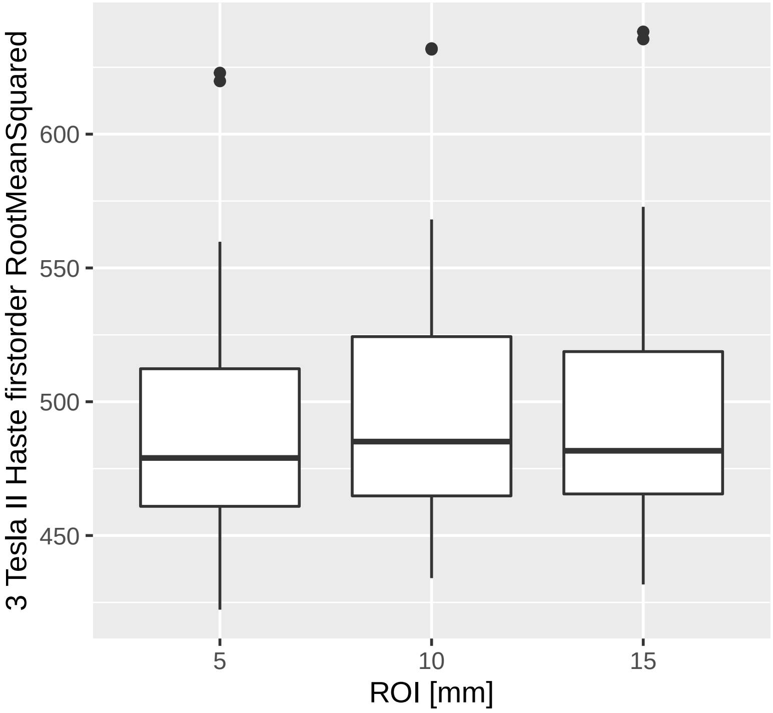
3 Tesla II Haste firstorder RootMeanSquared
450
500
550
600
5
10
15
ROI [mm]

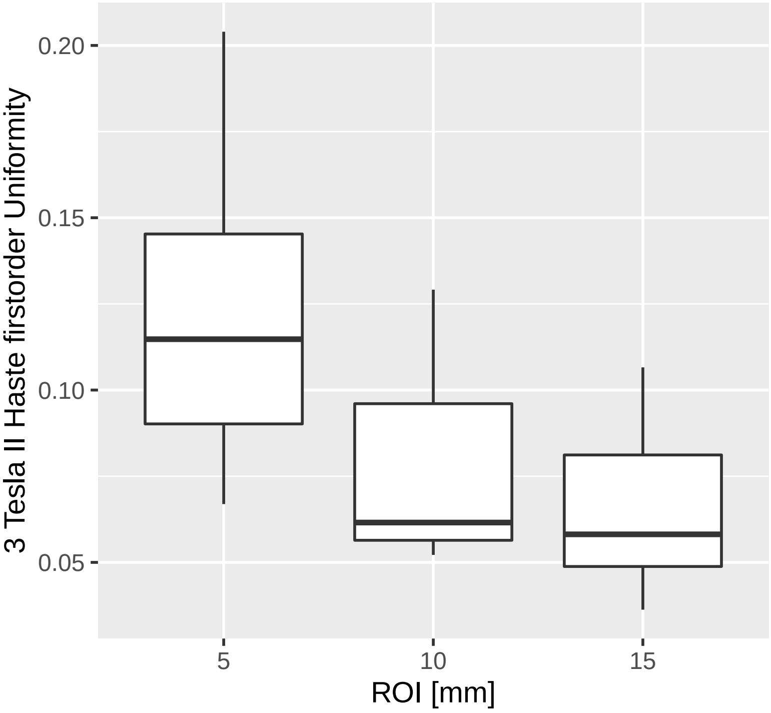
3 Tesla II Haste firstorder Uniformity
0.05
0.10
0.15
0.20
5
10
15
ROI [mm]

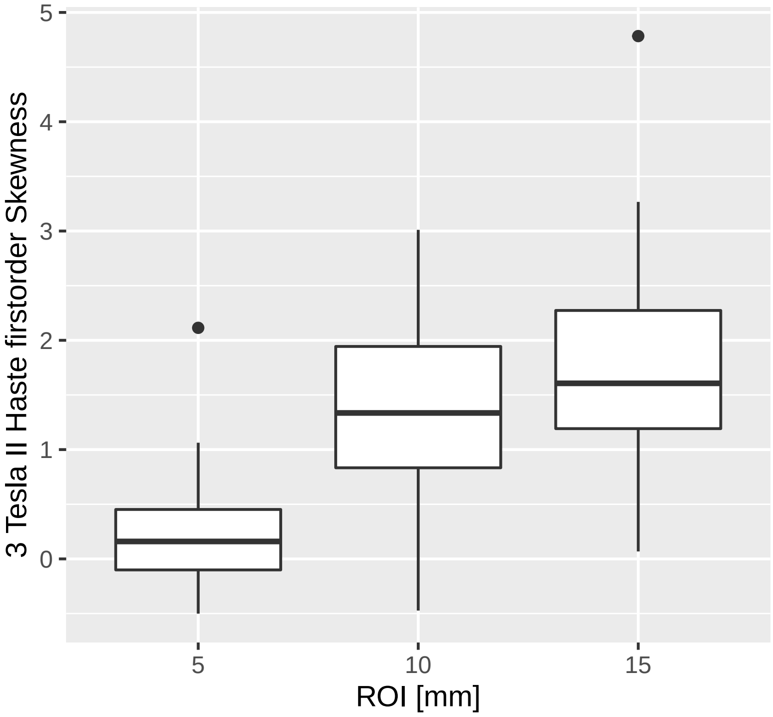
3 Tesla II Haste firstorder Skewness
0
1
2
3
4
5
10
15
ROI [mm]

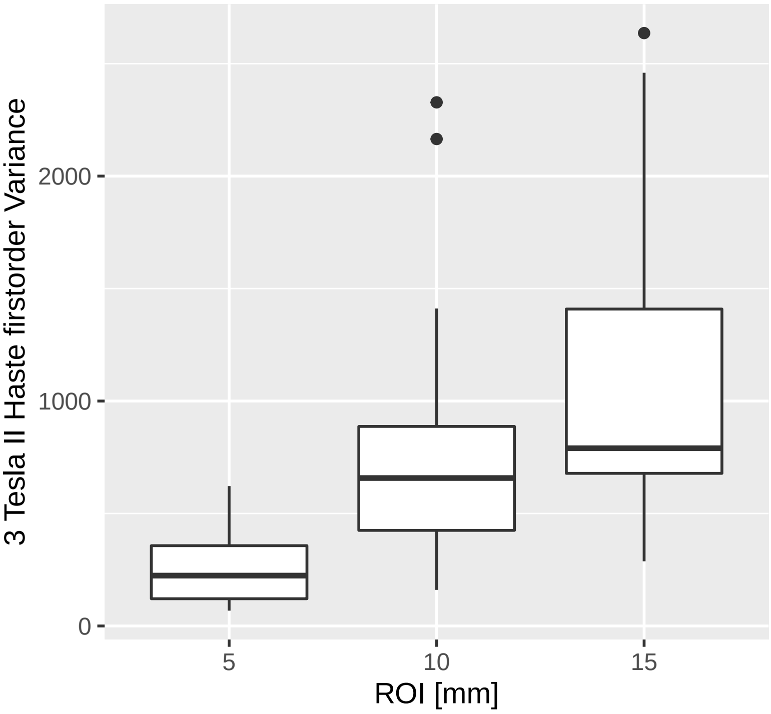
3 Tesla II Haste firstorder Variance
0
1000
2000
5
10
15
ROI [mm]

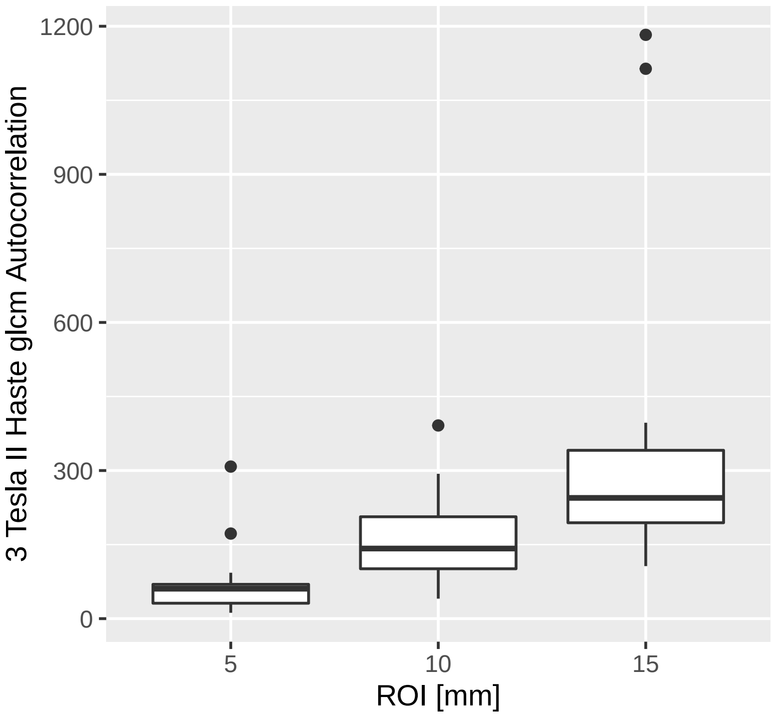
3 Tesla II Haste glcm Autocorrelation
1200
900
600
300
0
5
10
15
ROI [mm]

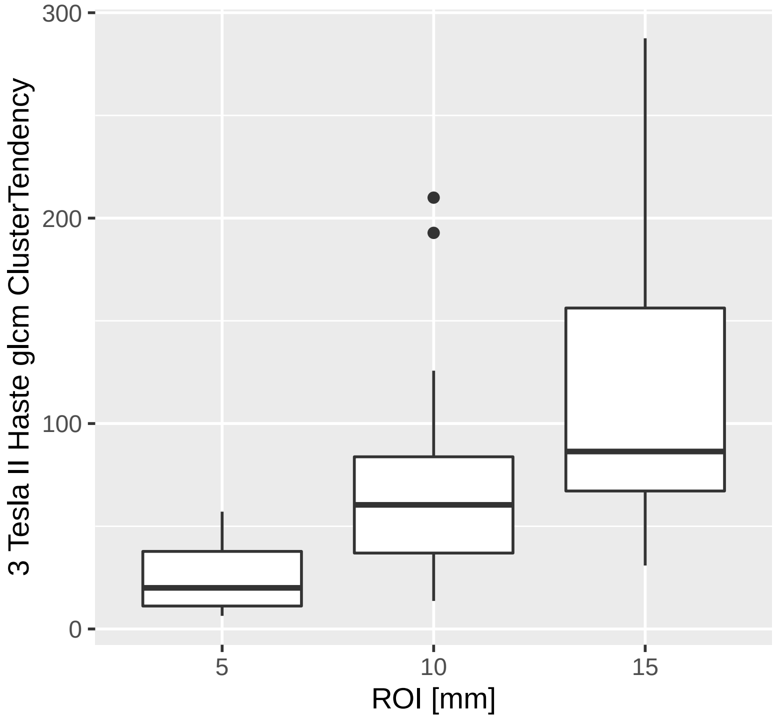
3 Tesla II Haste glcm ClusterTendency
300
200
100
0
5
10
15
ROI [mm]

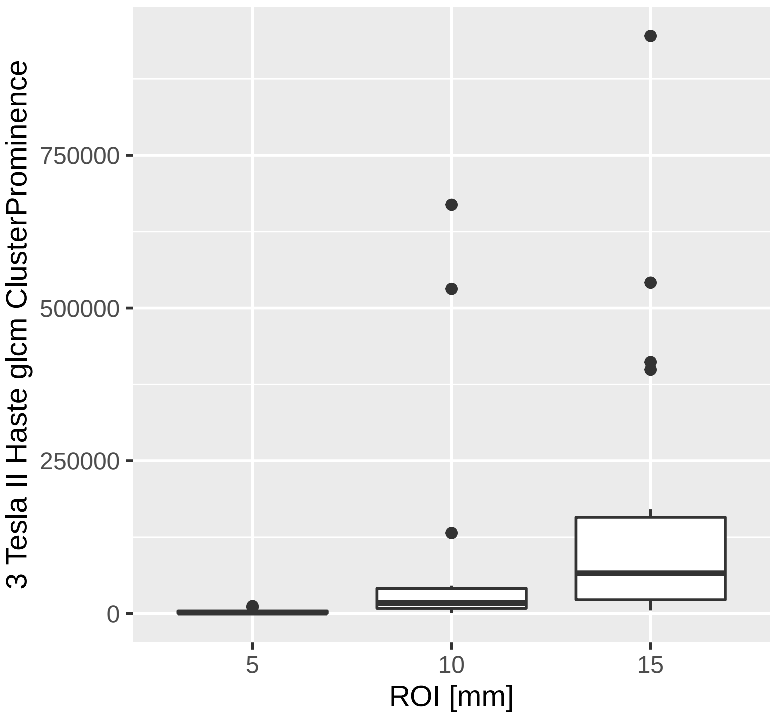
3 Tesla II Haste glcm ClusterProminence
750000
500000
250000
0
5
10
15
ROI [mm]

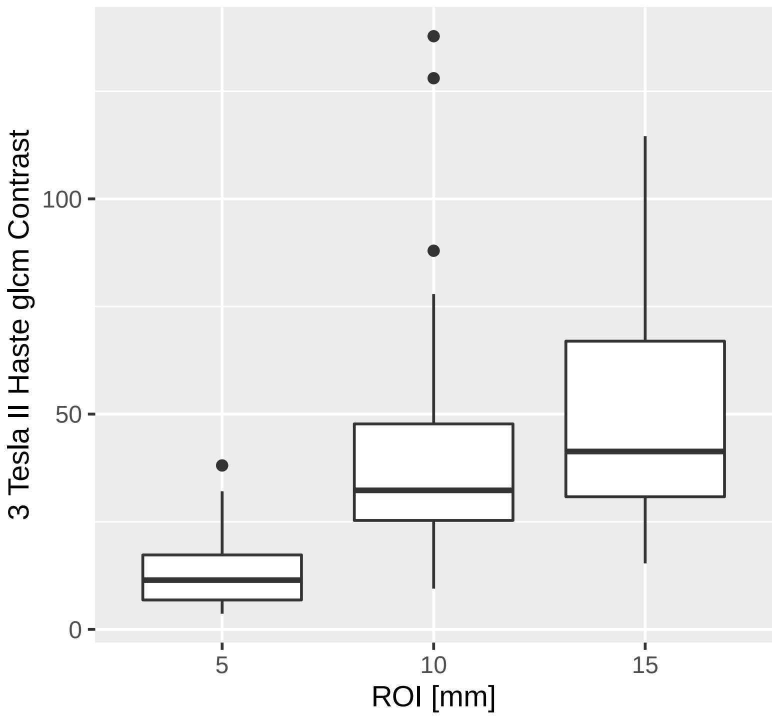
3 Tesla II Haste glcm Contrast
100
50
0
5
10
15
ROI [mm]

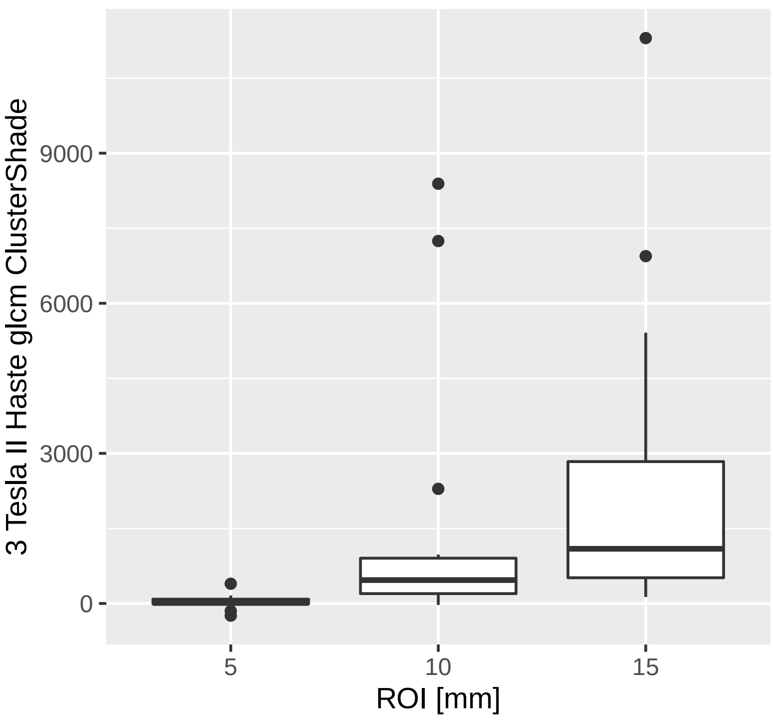
3 Tesla II Haste glcm ClusterShade
9000
6000
3000
0
5
10
15
ROI [mm]

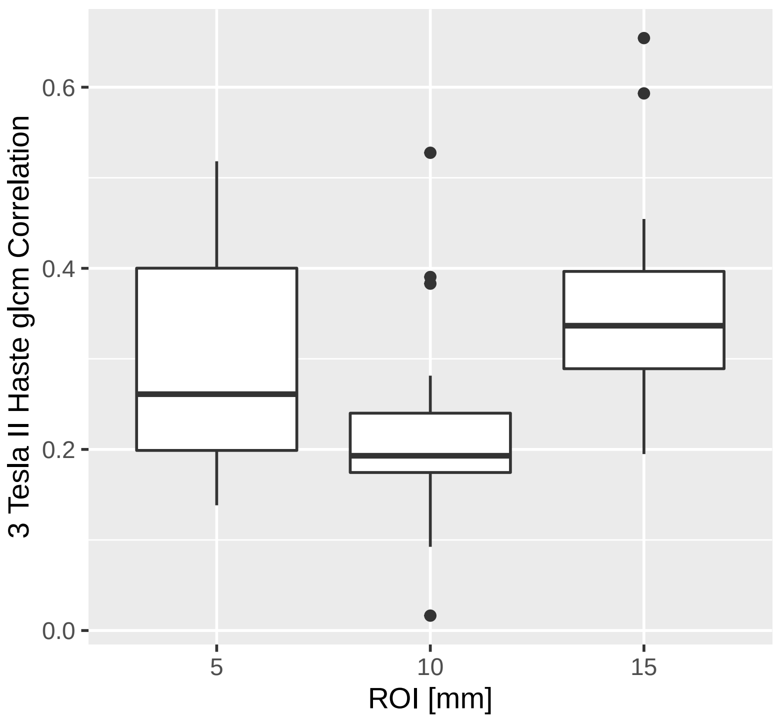
3 Tesla II Haste glcm Correlation
0.6
0.4
0.2
0.0
5
10
15
ROI [mm]

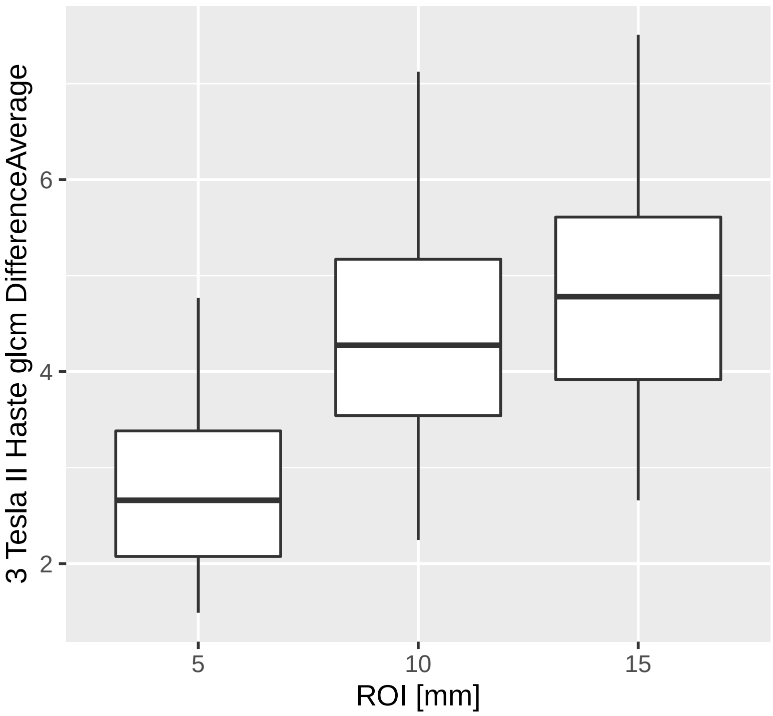
3 Tesla II Haste glcm DifferenceAverage
ROI [mm]
2
4
6
5
10
15

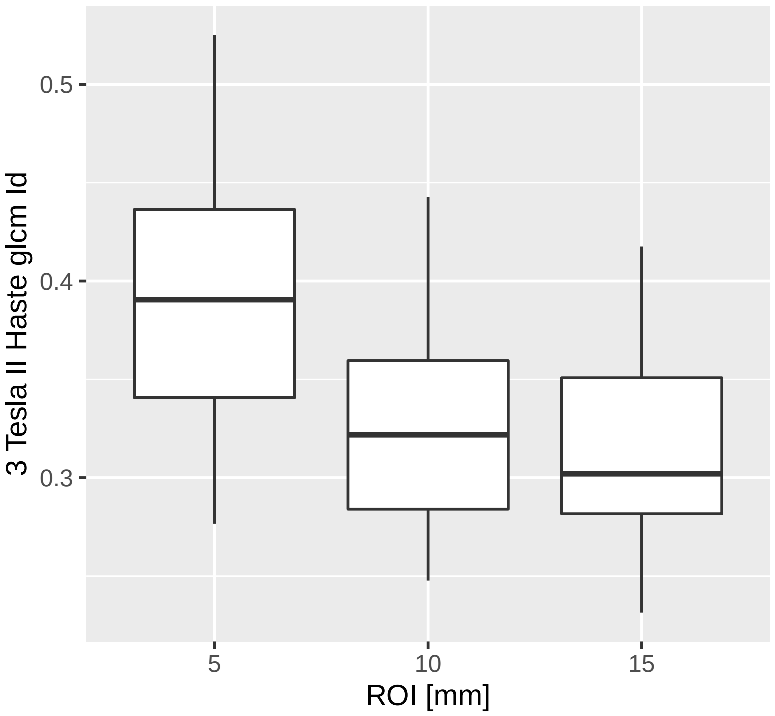
3 Tesla II Haste glcm Id
ROI [mm]
0.3
0.4
0.5
5
10
15

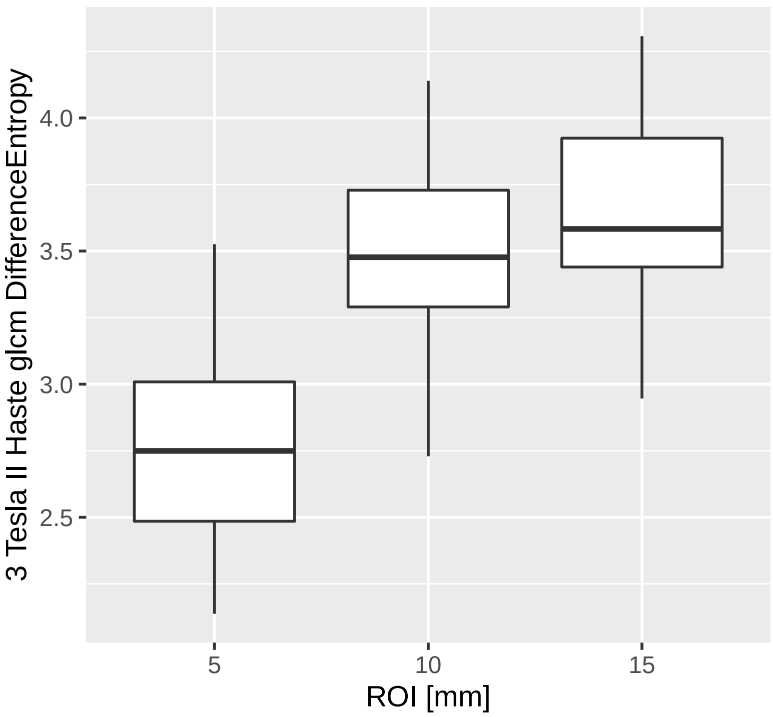
3 Tesla II Haste glcm DifferenceEntropy
ROI [mm]
2.5
3.0
3.5
4.0
5
10
15

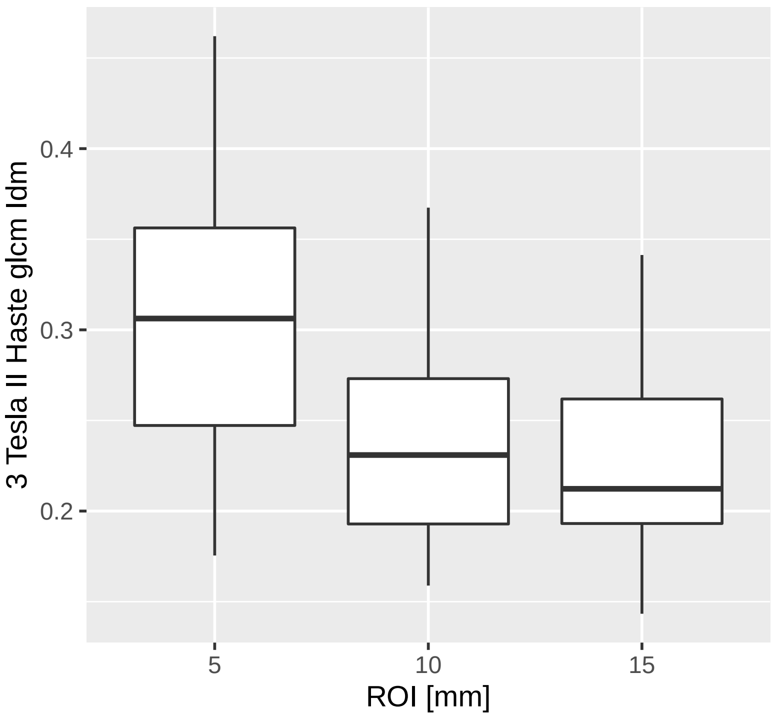
3 Tesla II Haste glcm Idm
ROI [mm]
0.2
0.3
0.4
5
10
15

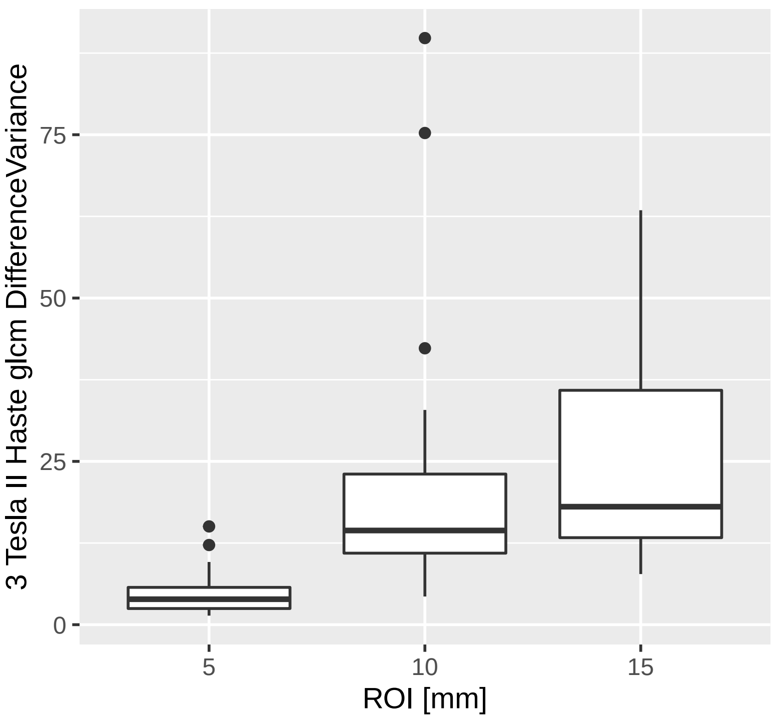
3 Tesla II Haste glcm DifferenceVariance
ROI [mm]
0
25
50
75
5
10
15

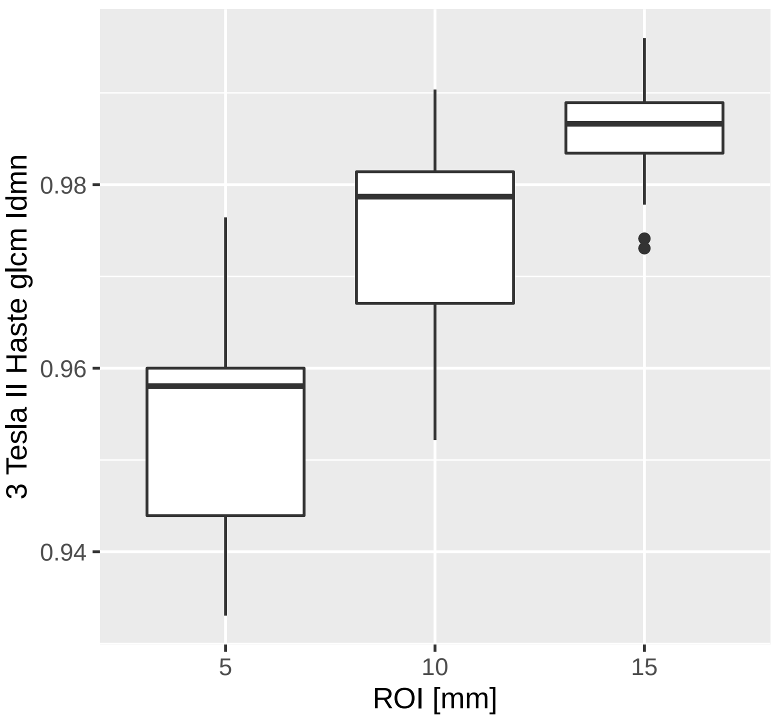
3 Tesla II Haste glcm Idmn
ROI [mm]
0.94
0.96
0.98
5
10
15

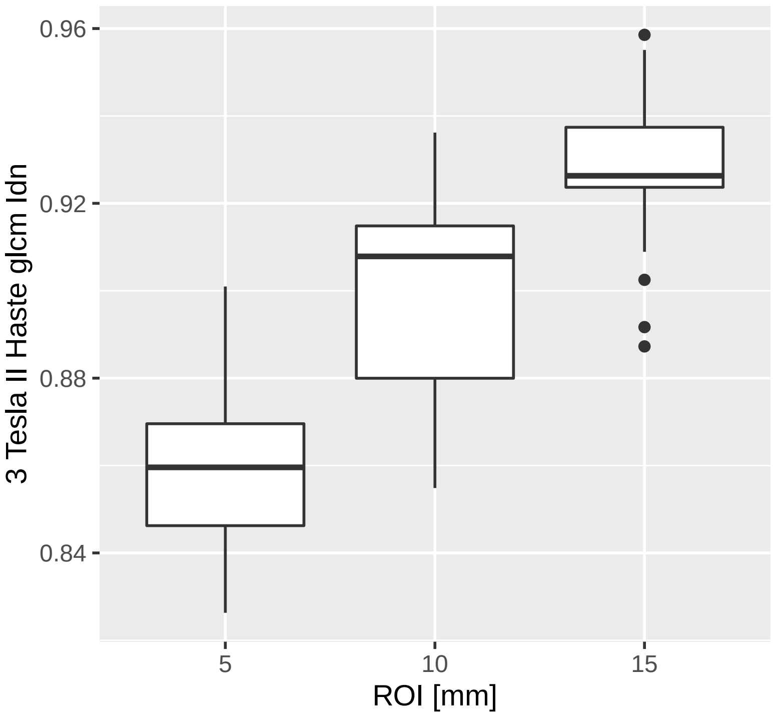
0.96
0.92
0.88
0.84
3 Tesla II Haste glcm Idn
5
10
15
ROI [mm]

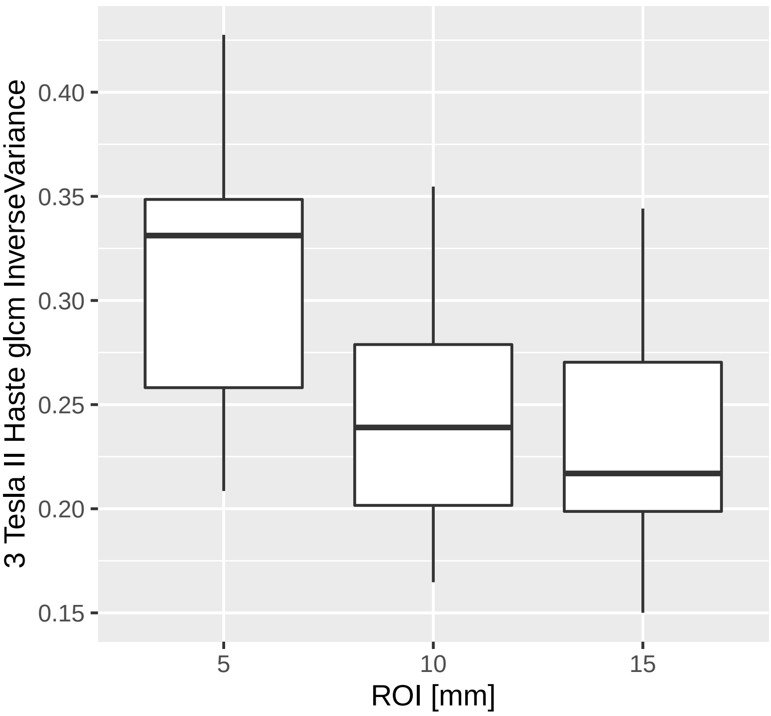
0.40
0.35
0.30
0.25
0.20
0.15
3 Tesla II Haste glcm InverseVariance
5
10
15
ROI [mm]

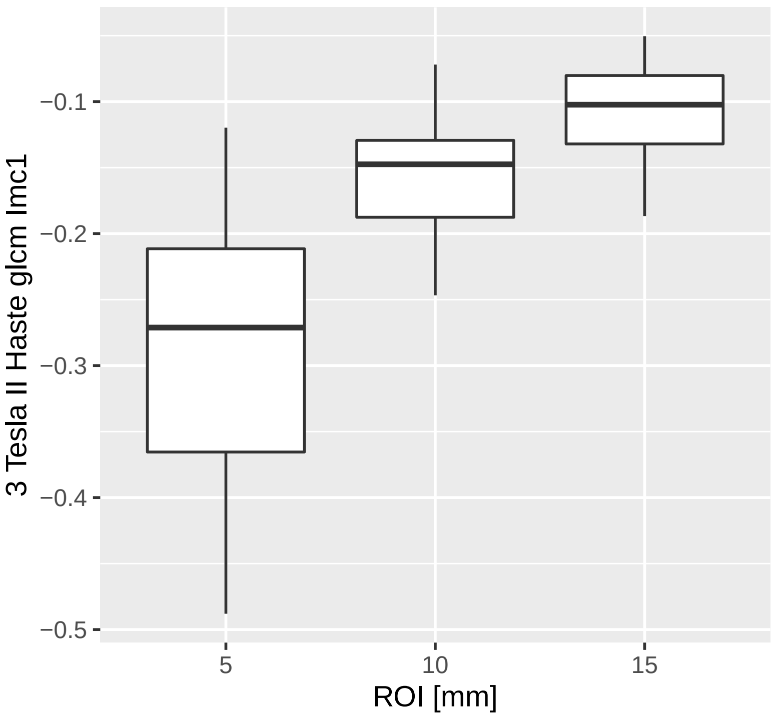
−0.1
−0.2
−0.3
−0.4
−0.5
3 Tesla II Haste glcm Imc1
5
10
15
ROI [mm]

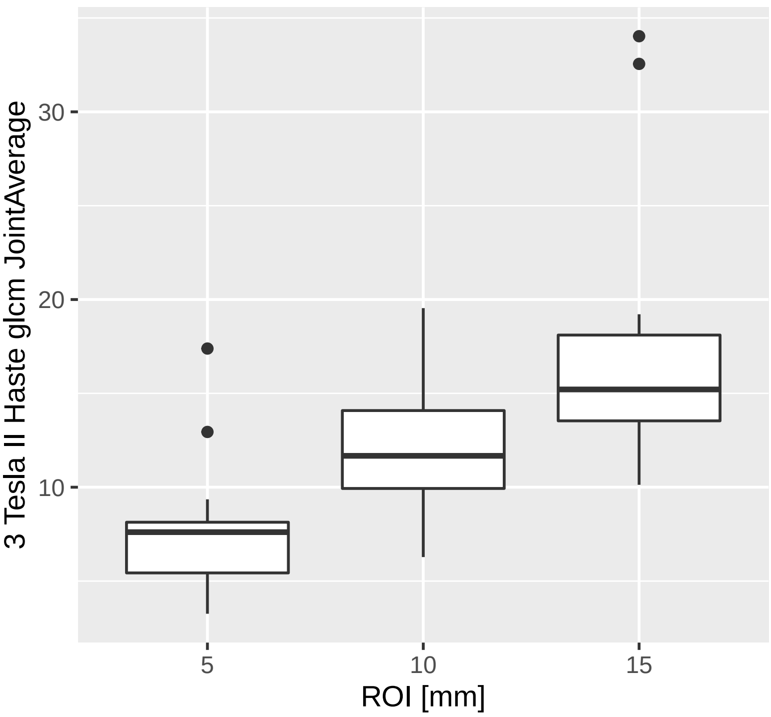
30
20
10
3 Tesla II Haste glcm JointAverage
5
10
15
ROI [mm]

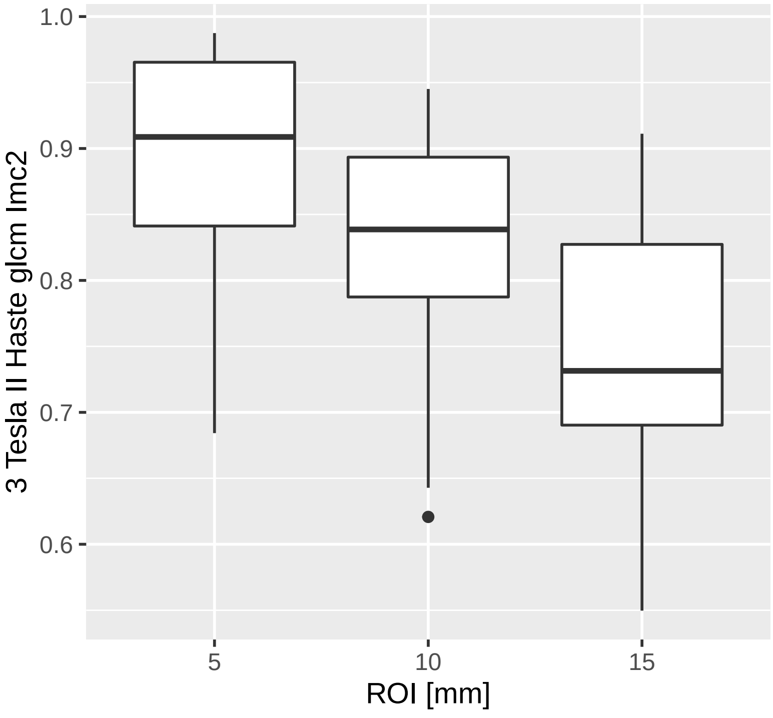
1.0
0.9
0.8
0.7
0.6
3 Tesla II Haste glcm Imc2
5
10
15
ROI [mm]

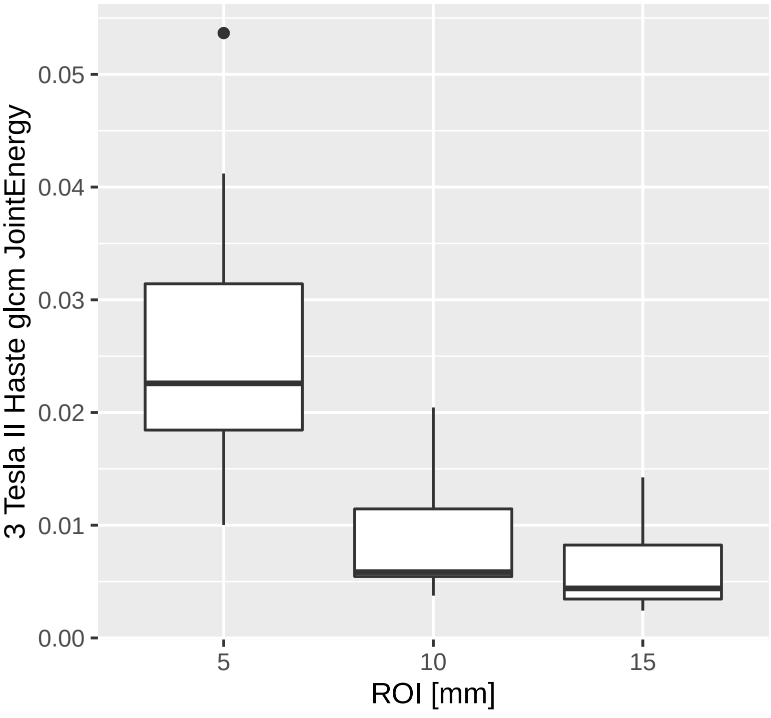
0.05
0.04
0.03
0.02
0.01
0.00
3 Tesla II Haste glcm JointEnergy
5
10
15
ROI [mm]

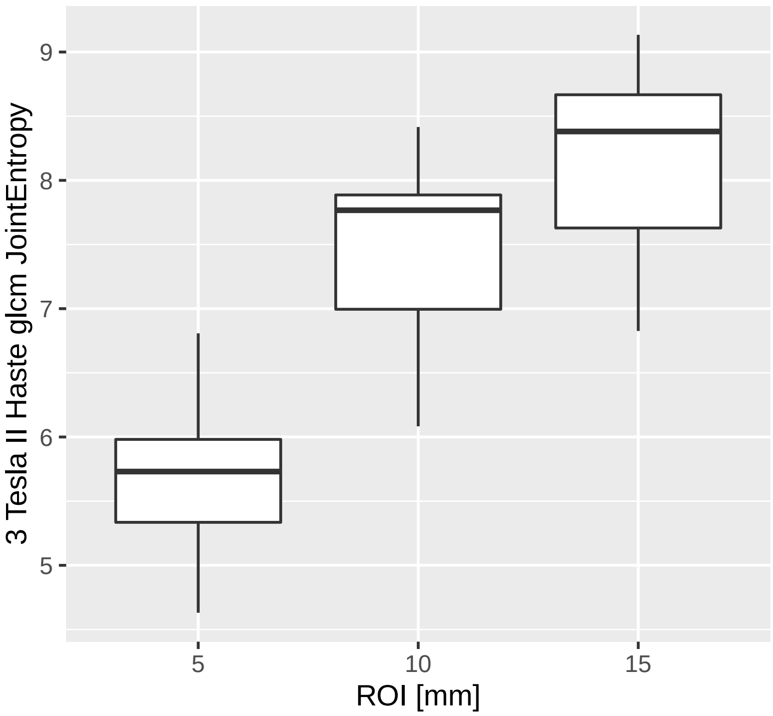
3 Tesla II Haste glcm JointEntropy
9
8
7
6
5
5
10
15
ROI [mm]

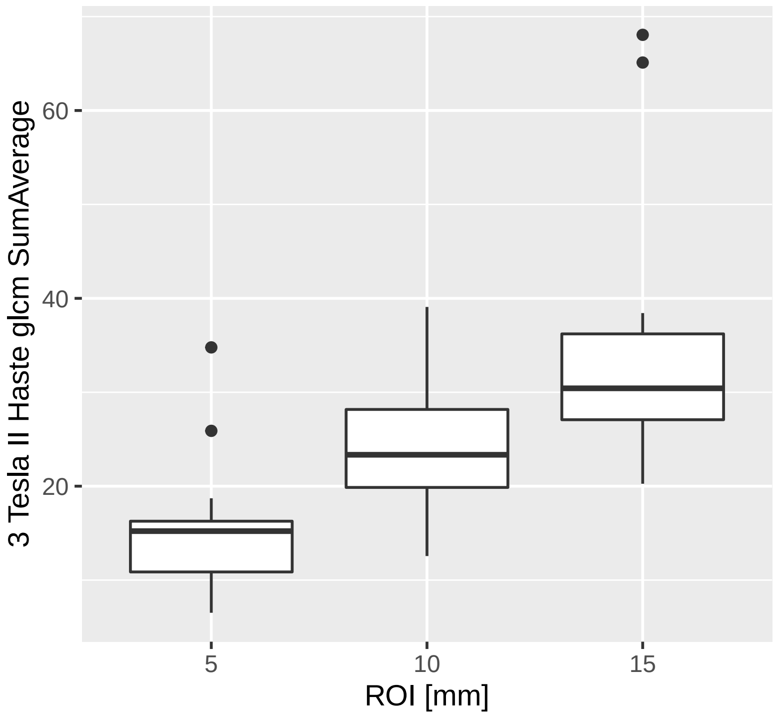
3 Tesla II Haste glcm SumAverage
60
40
20
5
10
15
ROI [mm]

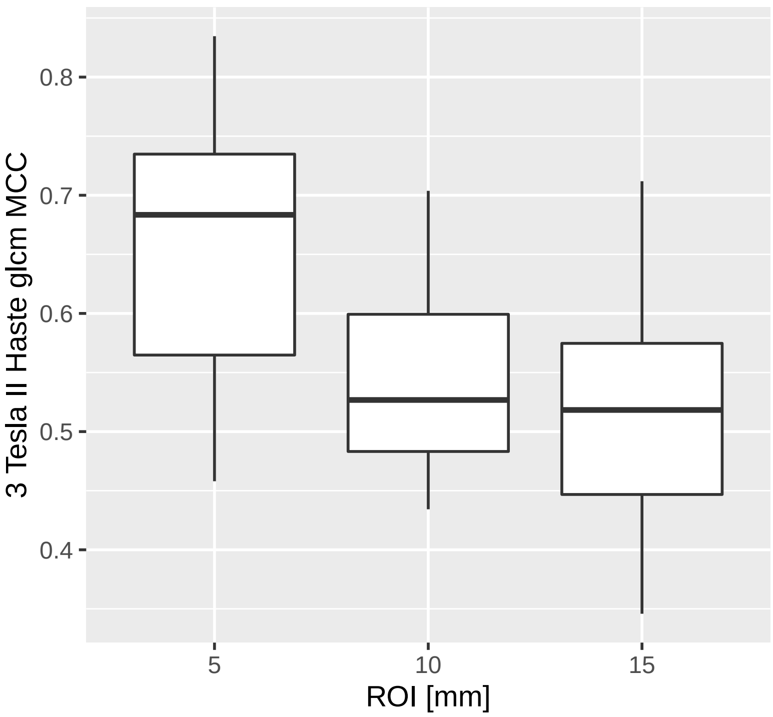
3 Tesla II Haste glcm MCC
0.8
0.7
0.6
0.5
0.4
5
10
15
ROI [mm]

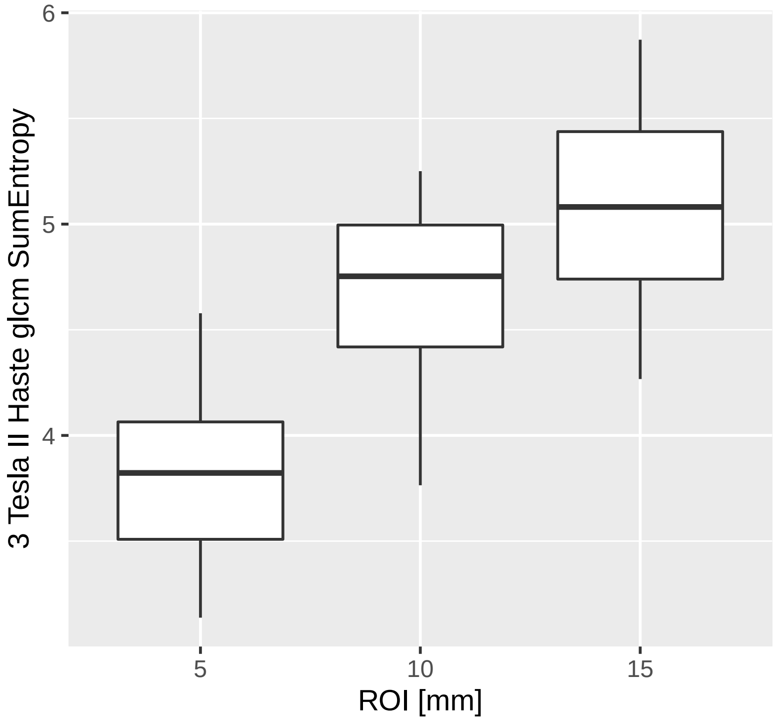
3 Tesla II Haste glcm SumEntropy
6
5
4
5
10
15
ROI [mm]

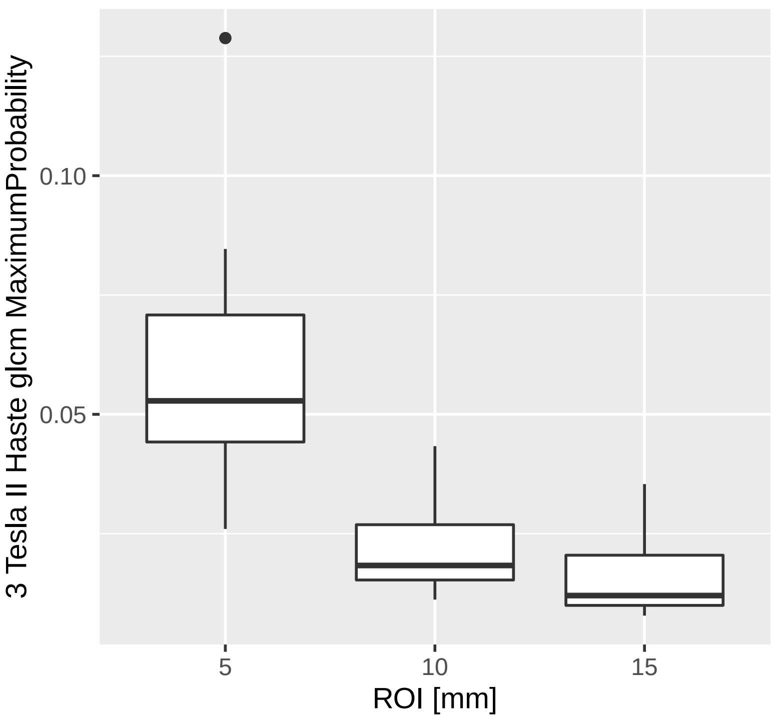
3 Tesla II Haste glcm MaximumProbability
0.10
0.05
5
10
15
ROI [mm]

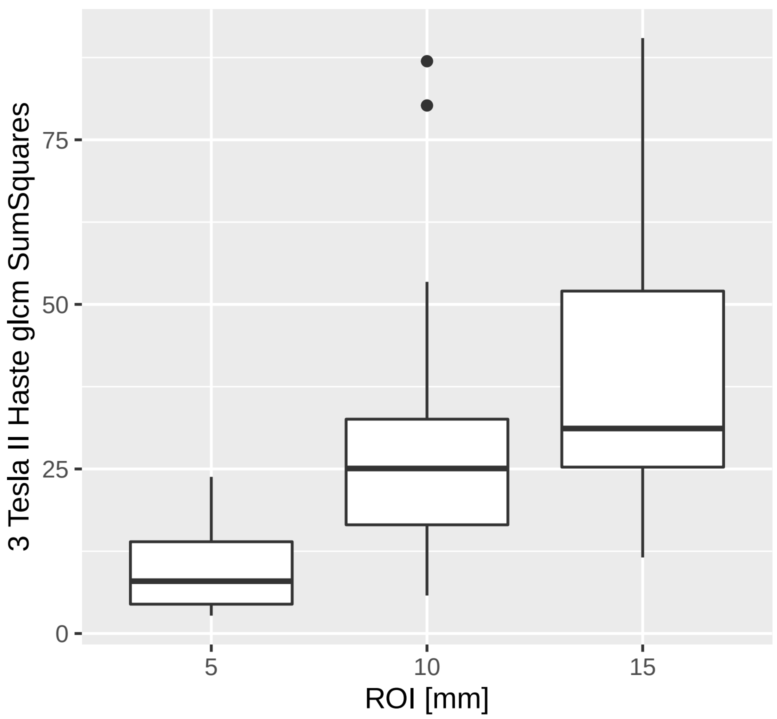
3 Tesla II Haste glcm SumSquares
75
50
25
0
5
10
15
ROI [mm]

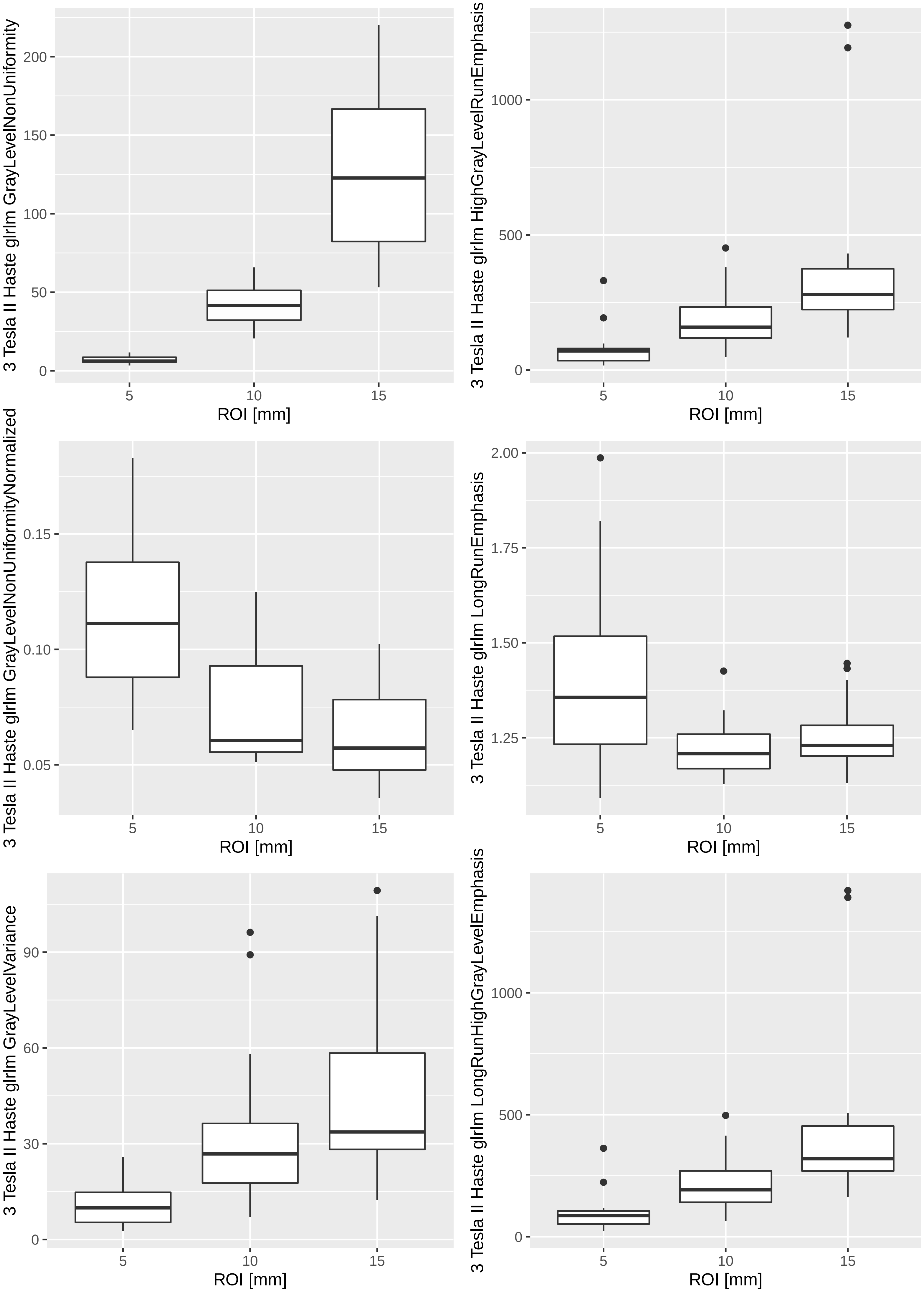
3 Tesla II Haste glrlm GrayLevelNonUniformity
0
50
100
150
200
5
10
15
ROI [mm]
3 Tesla II Haste glrlm HighGrayLevelRunEmphasis
0
500
1000
5
10
15
ROI [mm]
3 Tesla II Haste glrlm GrayLevelNonUniformityNormalized
0.05
0.10
0.15
5
10
15
ROI [mm]
3 Tesla II Haste glrlm LongRunEmphasis
1.25
1.50
1.75
2.00
5
10
15
ROI [mm]
3 Tesla II Haste glrlm GrayLevelVariance
0
30
60
90
5
10
15
ROI [mm]
3 Tesla II Haste glrlm LongRunHighGrayLevelEmphasis
0
500
1000
5
10
15
ROI [mm]

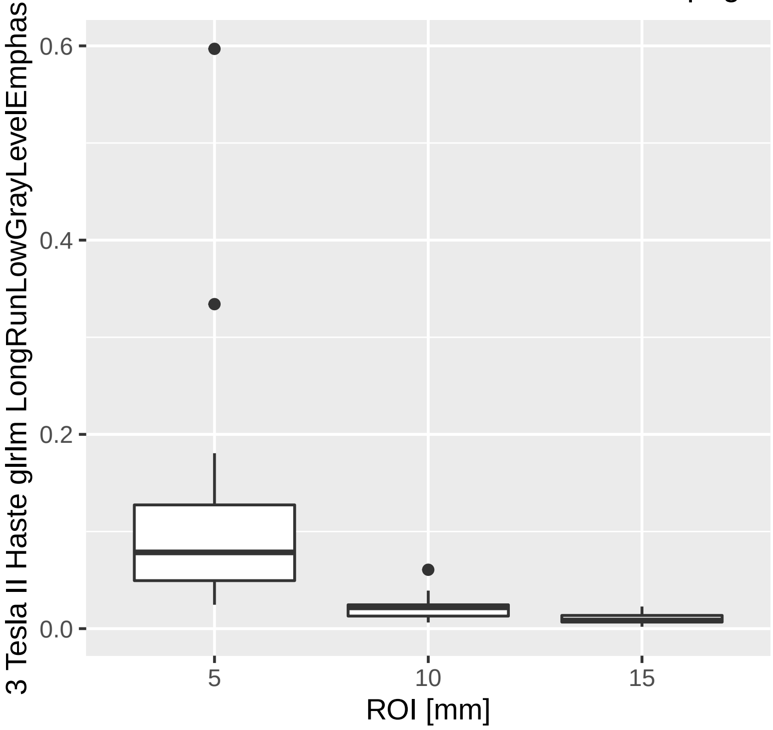
3 Tesla II Haste glrlm LongRunLowGrayLevelEmphasis
0.0
0.2
0.4
0.6
5
10
15
ROI [mm]

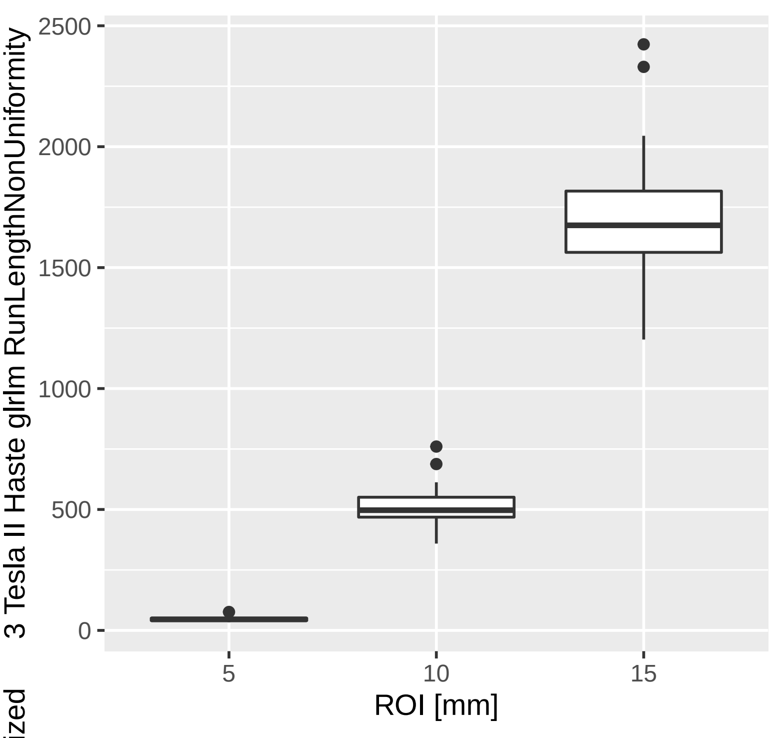
3 Tesla II Haste glrlm RunLengthNonUniformity
0
500
1000
1500
2000
2500
5
10
15
ROI [mm]

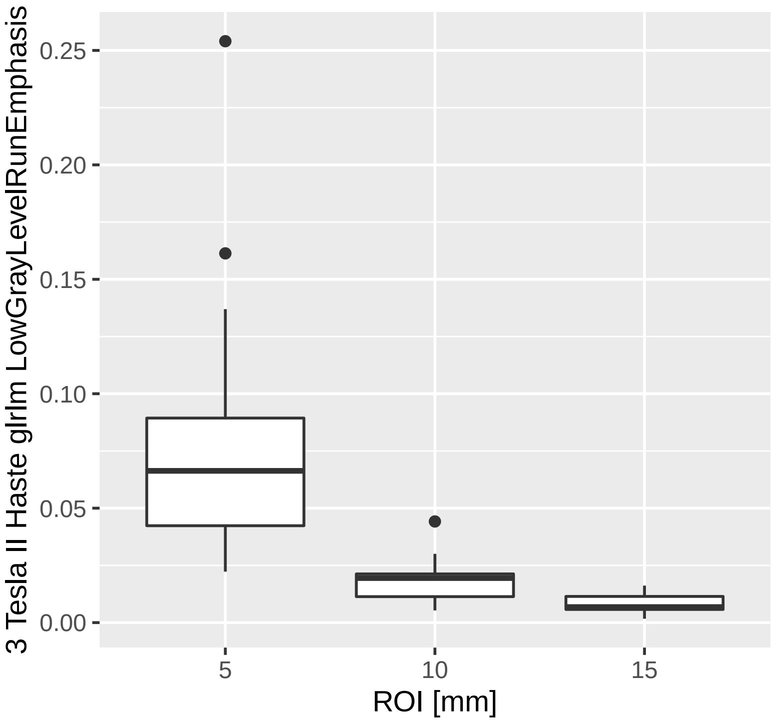
3 Tesla II Haste glrlm LowGrayLevelRunEmphasis
0.00
0.05
0.10
0.15
0.20
0.25
5
10
15
ROI [mm]

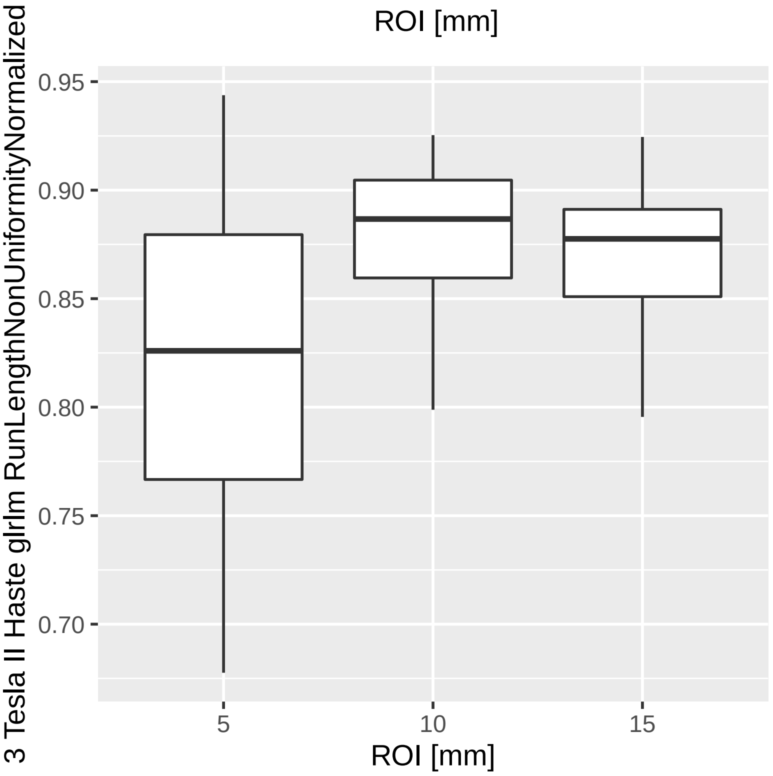
3 Tesla II Haste glrlm RunLengthNonUniformityNormalized
0.70
0.75
0.80
0.85
0.90
0.95
5
10
15
ROI [mm]

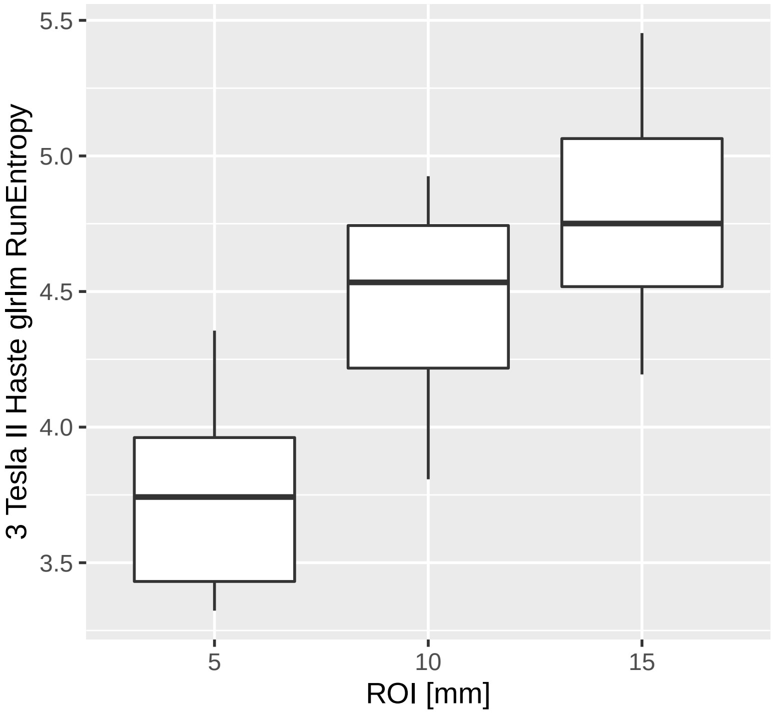
3 Tesla II Haste glrlm RunEntropy
3.5
4.0
4.5
5.0
5.5
5
10
15
ROI [mm]

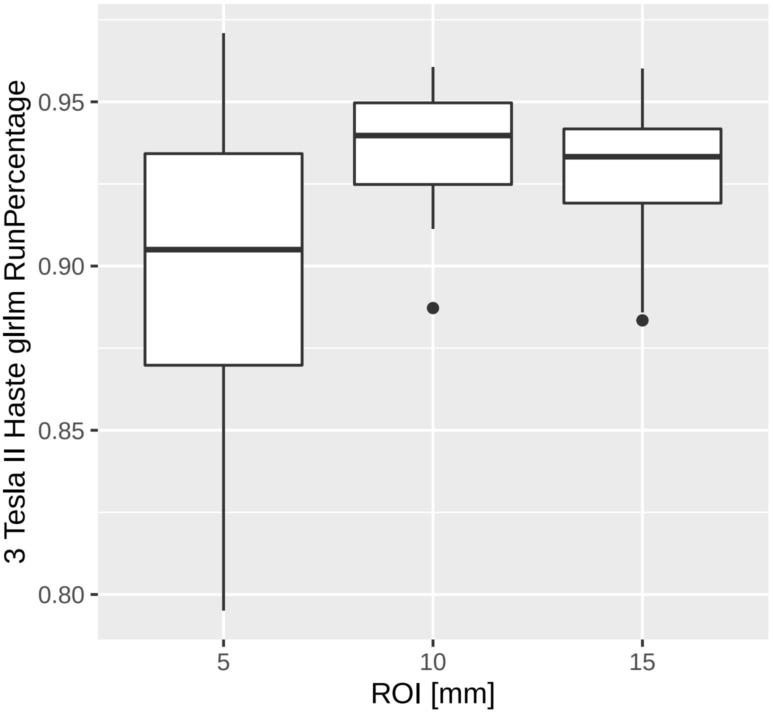
3 Tesla II Haste glrlm RunPercentage
0.80
0.85
0.90
0.95
5
10
15
ROI [mm]

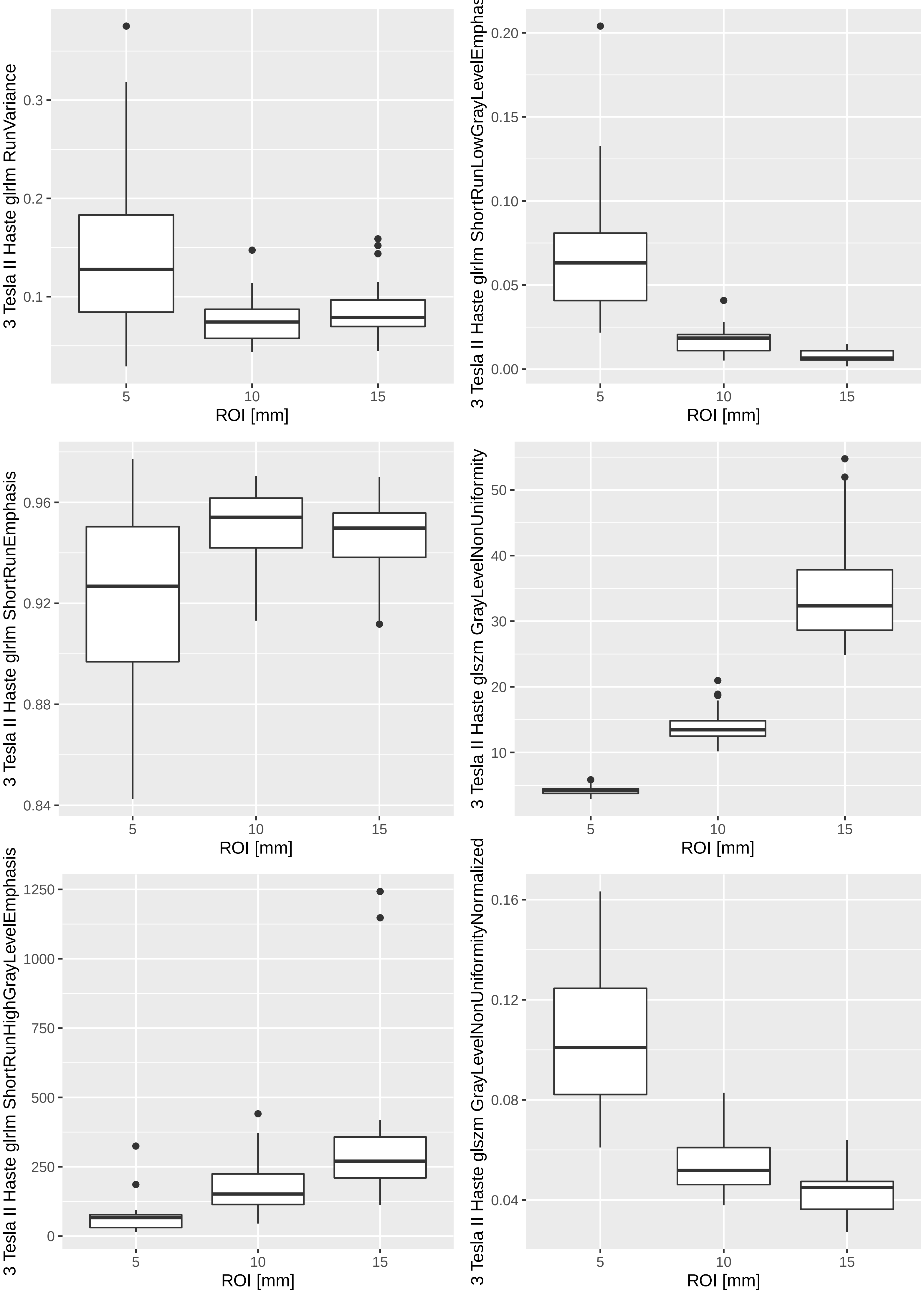
3 Tesla II Haste glrlm RunVariance
ROI [mm]
3 Tesla II Haste glrlm ShortRunLowGrayLevelEmphasis
ROI [mm]
3 Tesla II Haste glrlm ShortRunEmphasis
ROI [mm]
3 Tesla II Haste glszm GrayLevelNonUniformity
ROI [mm]
3 Tesla II Haste glrlm ShortRunHighGrayLevelEmphasis
ROI [mm]
3 Tesla II Haste glszm GrayLevelNonUniformityNormalized
ROI [mm]

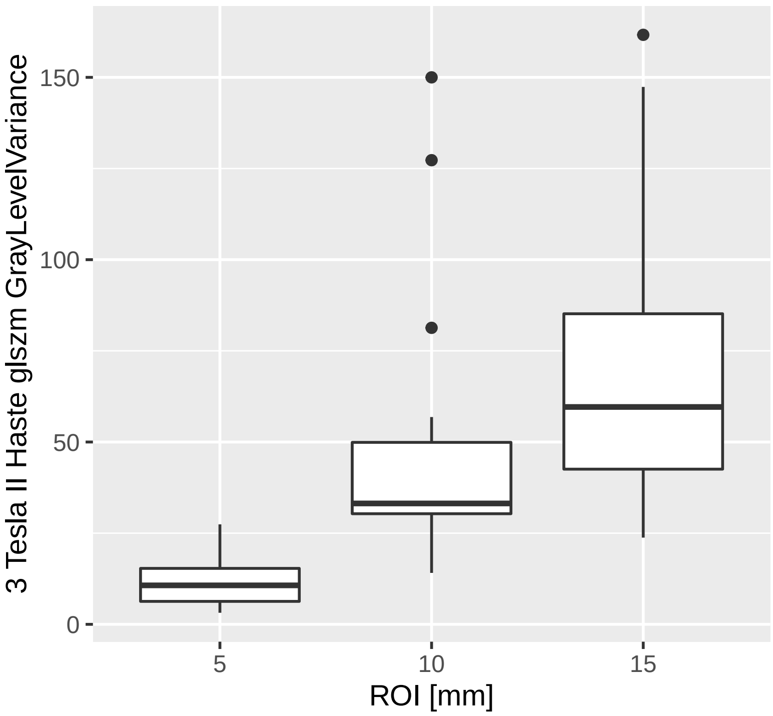

3 Tesla II Haste glszm GrayLevelVariance
150
100
50
0
5
10
15
ROI [mm]

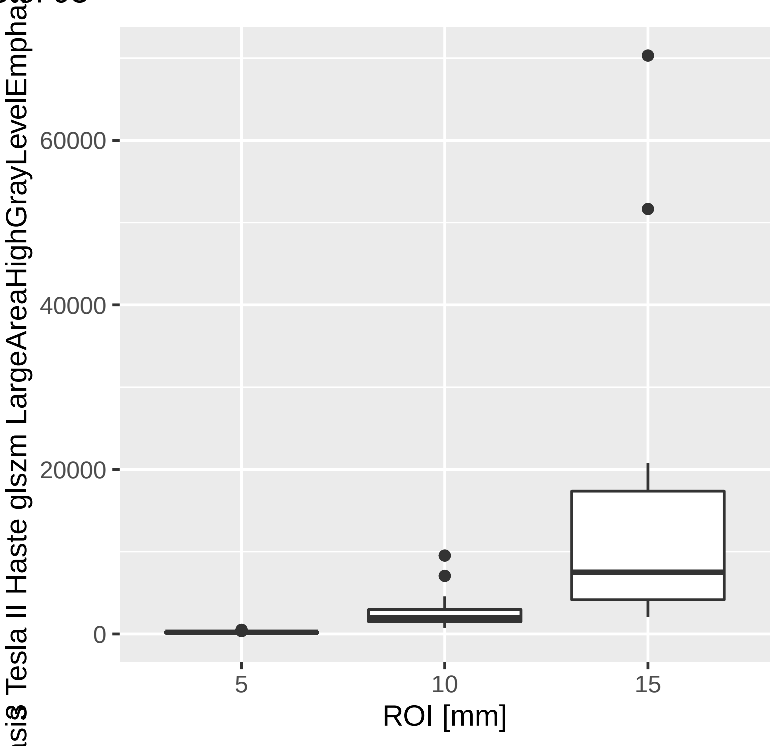

3 Tesla II Haste glszm LargeAreaHighGrayLevelEmphasis
60000
40000
20000
0
5
10
15
ROI [mm]

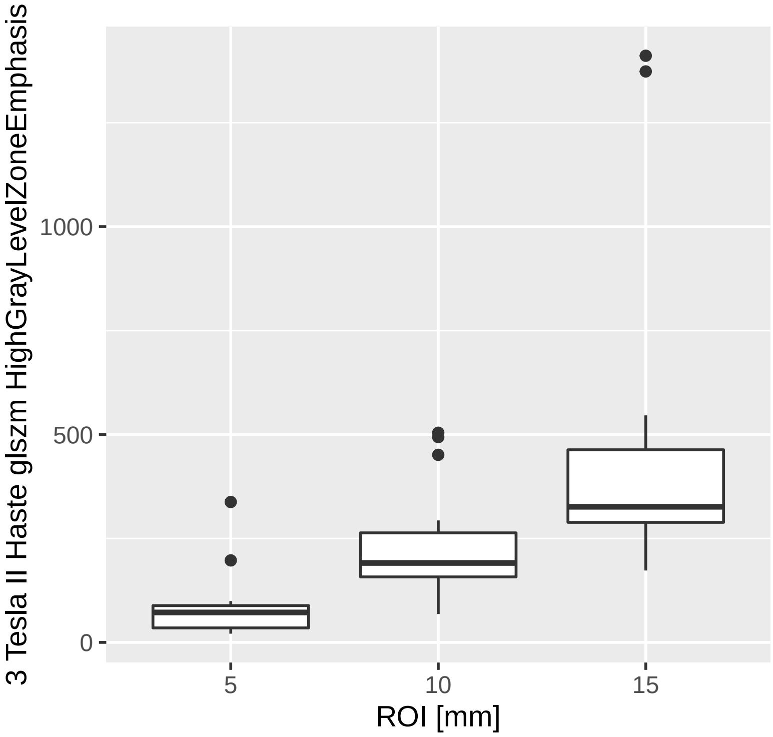

3 Tesla II Haste glszm HighGrayLevelZoneEmphasis
1000
500
0
5
10
15
ROI [mm]

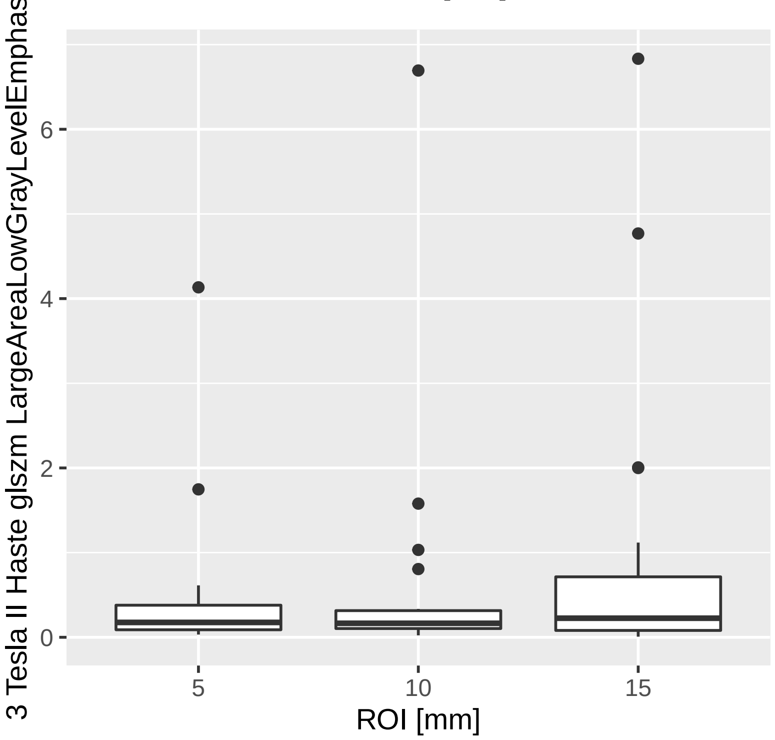

3 Tesla II Haste glszm LargeAreaLowGrayLevelEmphasis
6
4
2
0
5
10
15
ROI [mm]

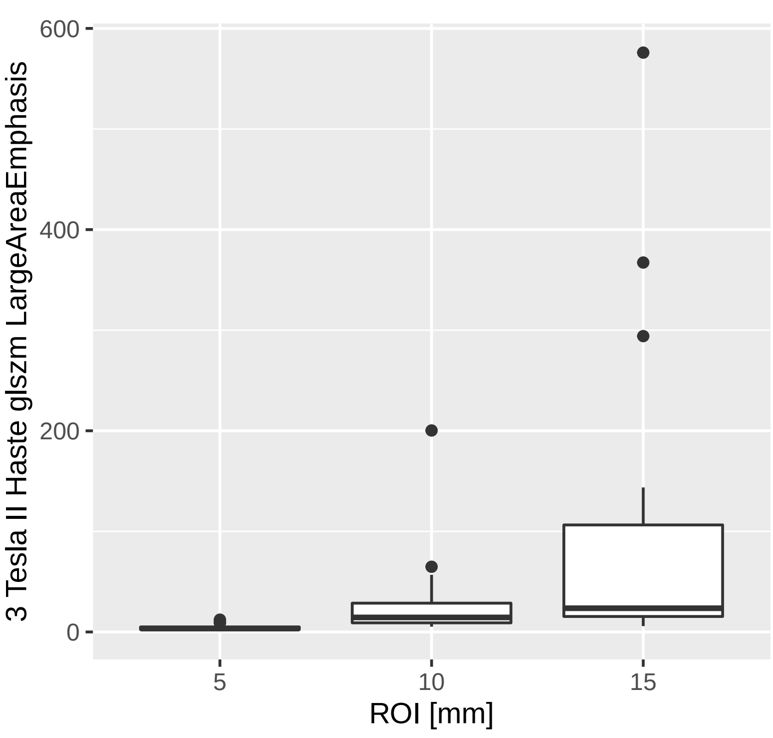

3 Tesla II Haste glszm LargeAreaEmphasis
600
400
200
0
5
10
15
ROI [mm]

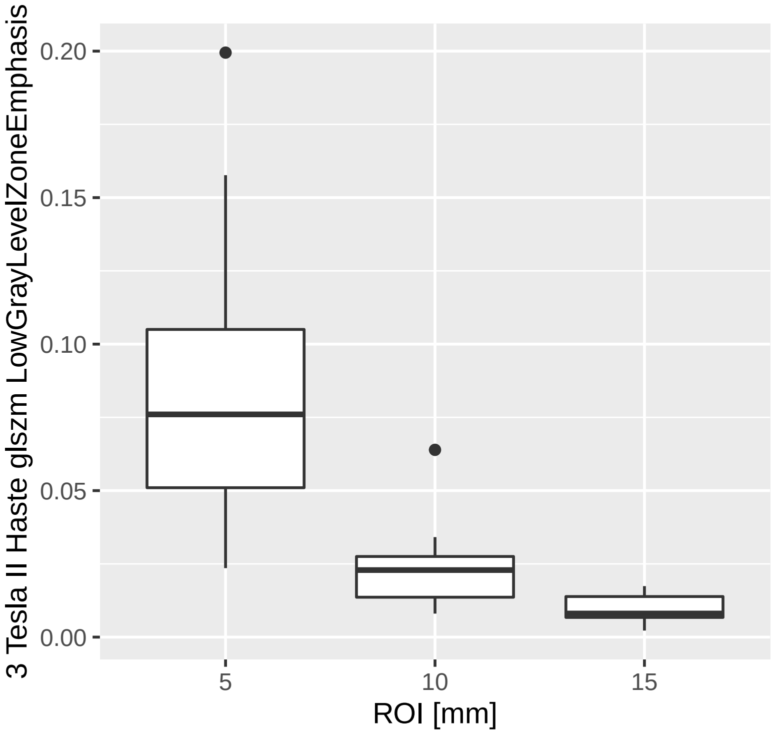

3 Tesla II Haste glszm LowGrayLevelZoneEmphasis
0.20
0.15
0.10
0.05
0.00
5
10
15
ROI [mm]

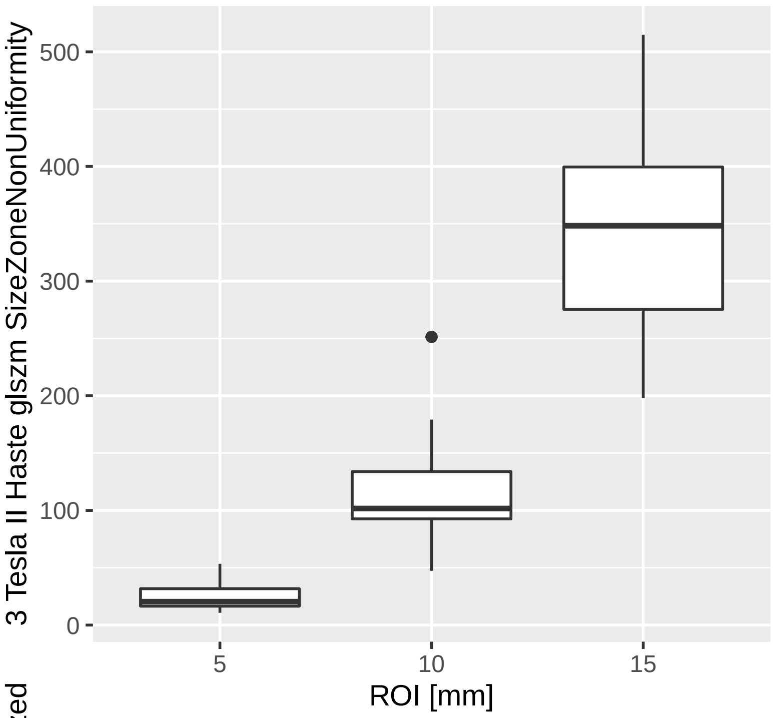
3 Tesla II Haste glszm SizeZoneNonUniformity
0
100
200
300
400
500
5
10
15
ROI [mm]

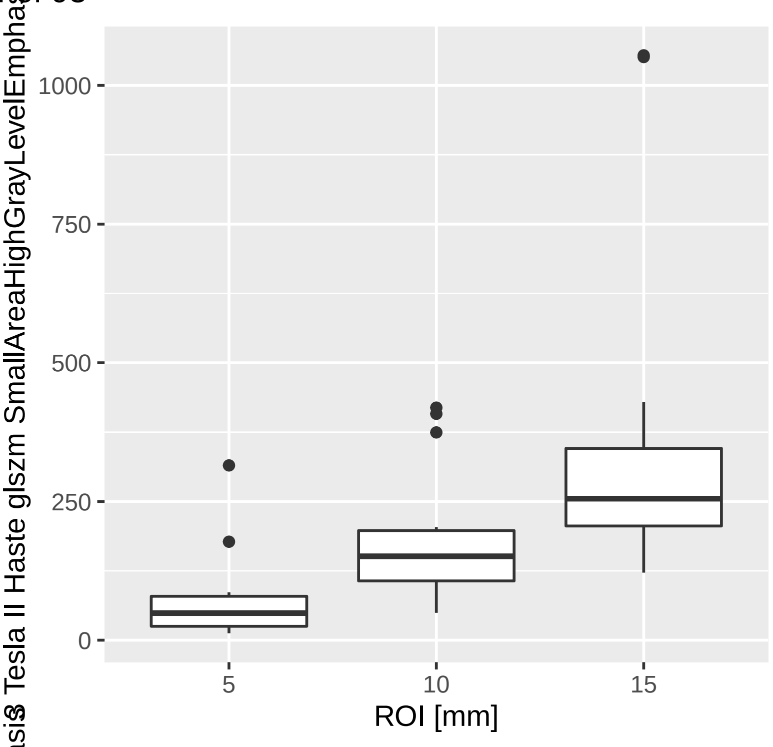
3 Tesla II Haste glszm SmallAreaHighGrayLevelEmphasis
0
250
500
750
1000
5
10
15
ROI [mm]

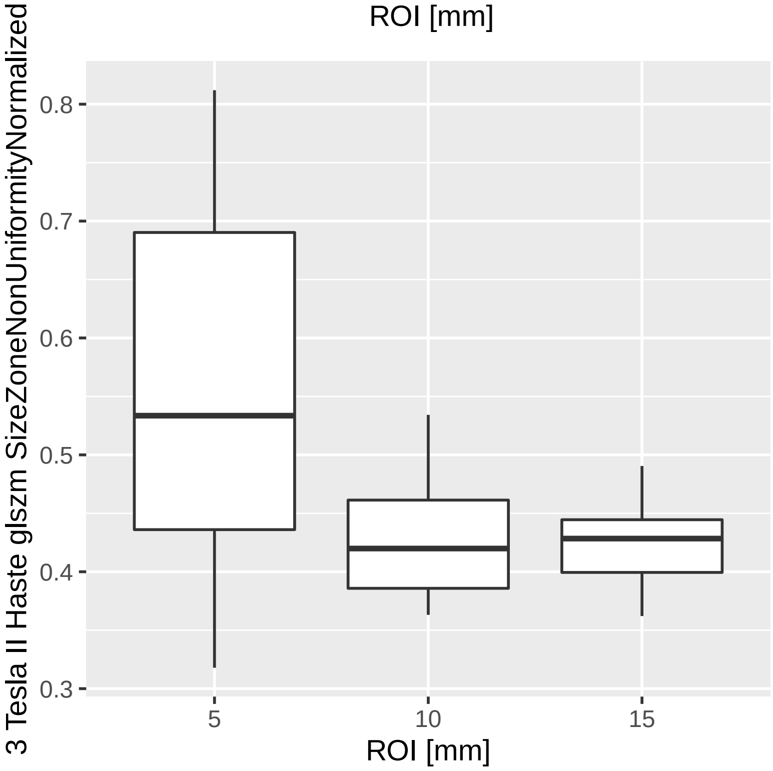
3 Tesla II Haste glszm SizeZoneNonUniformityNormalized
0.3
0.4
0.5
0.6
0.7
0.8
5
10
15
ROI [mm]

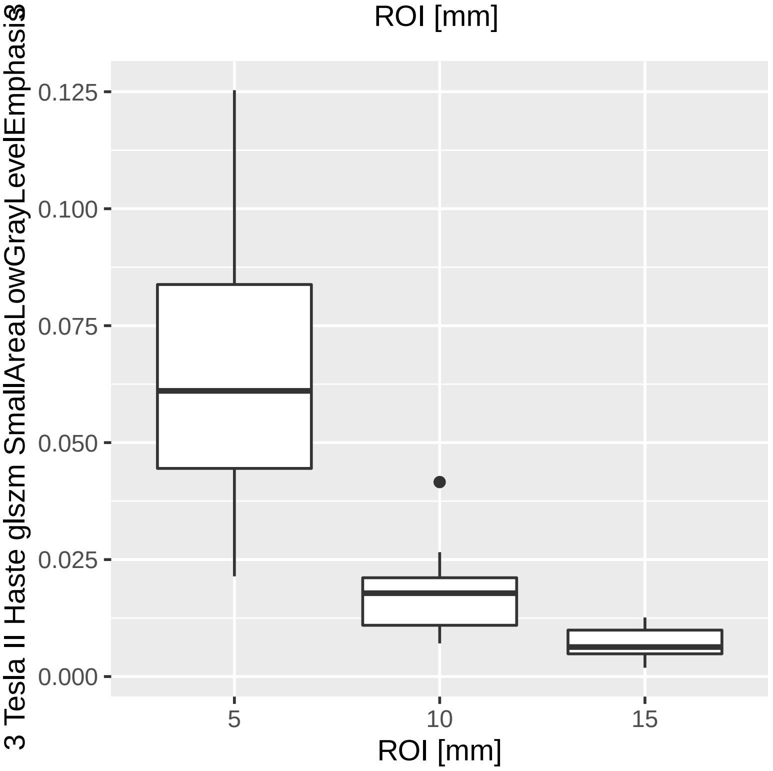
3 Tesla II Haste glszm SmallAreaLowGrayLevelEmphasis
0.000
0.025
0.050
0.075
0.100
0.125
5
10
15
ROI [mm]

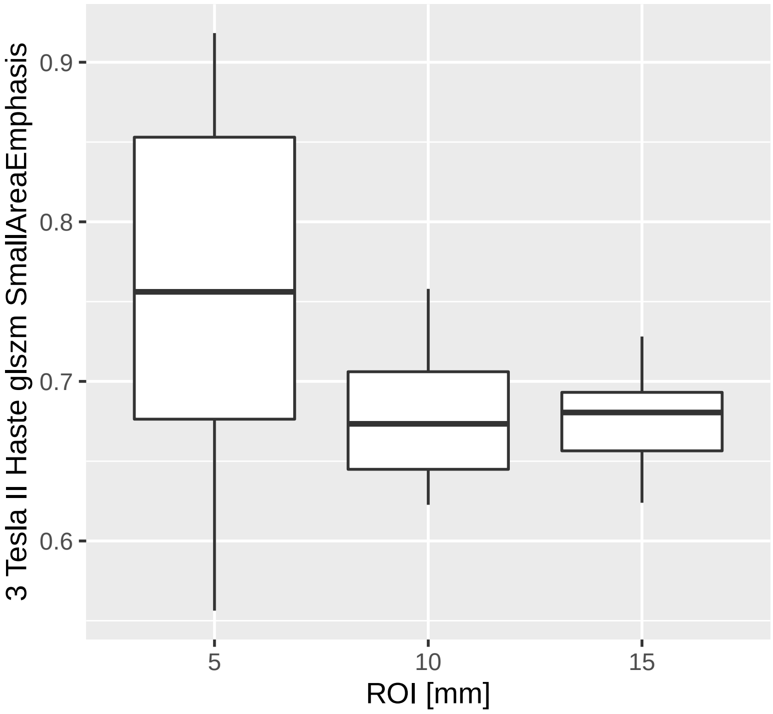
3 Tesla II Haste glszm SmallAreaEmphasis
0.6
0.7
0.8
0.9
5
10
15
ROI [mm]

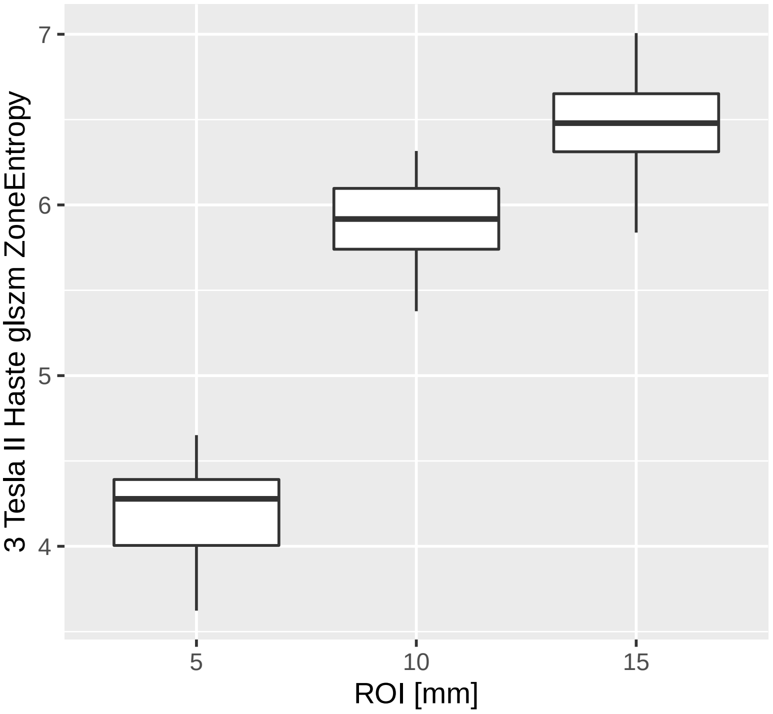
3 Tesla II Haste glszm ZoneEntropy
4
5
6
7
5
10
15
ROI [mm]

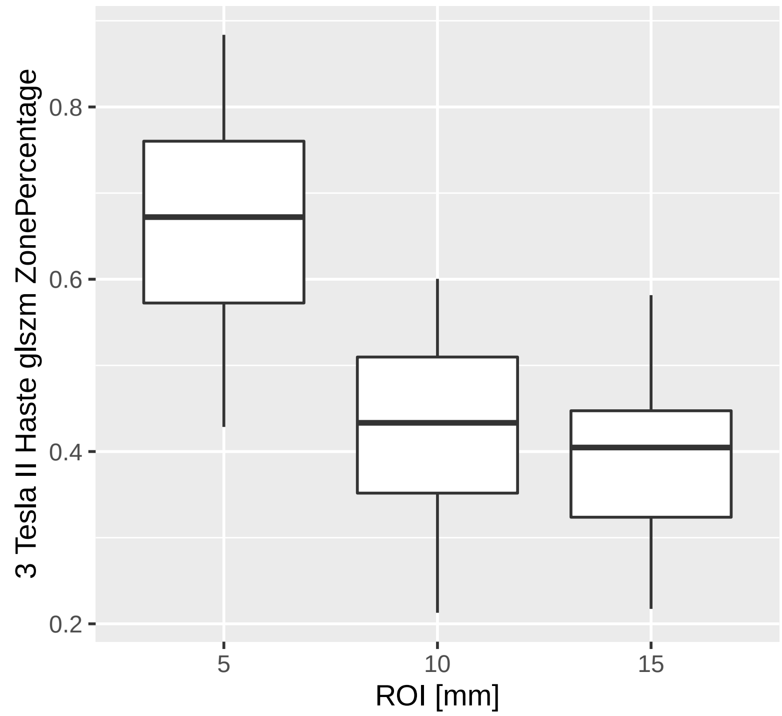
3 Tesla II Haste glszm ZonePercentage
0.2
0.4
0.6
0.8
5
10
15
ROI [mm]

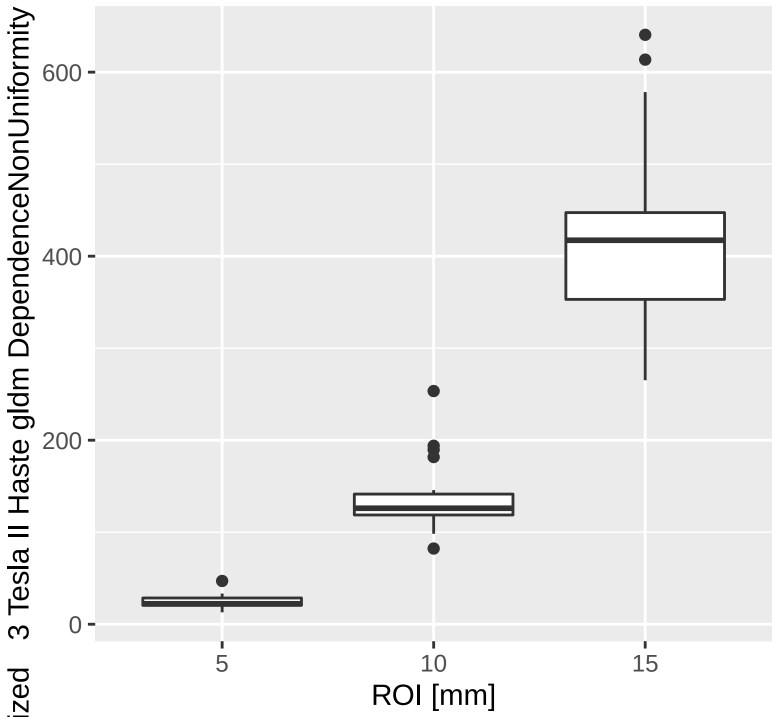
3 Tesla II Haste gldm DependenceNonUniformity
0
200
400
600
5
10
15
ROI [mm]

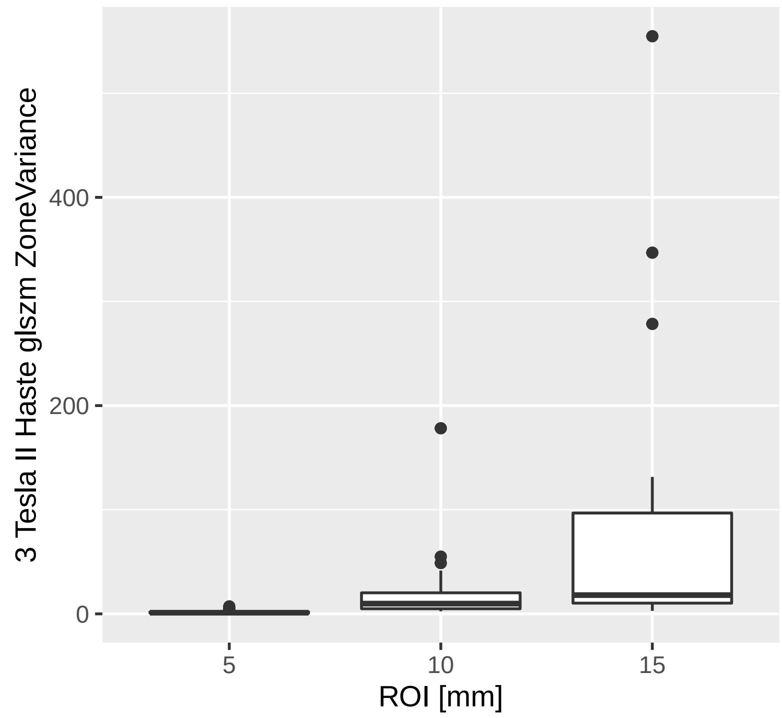
3 Tesla II Haste glszm ZoneVariance
0
200
400
5
10
15
ROI [mm]

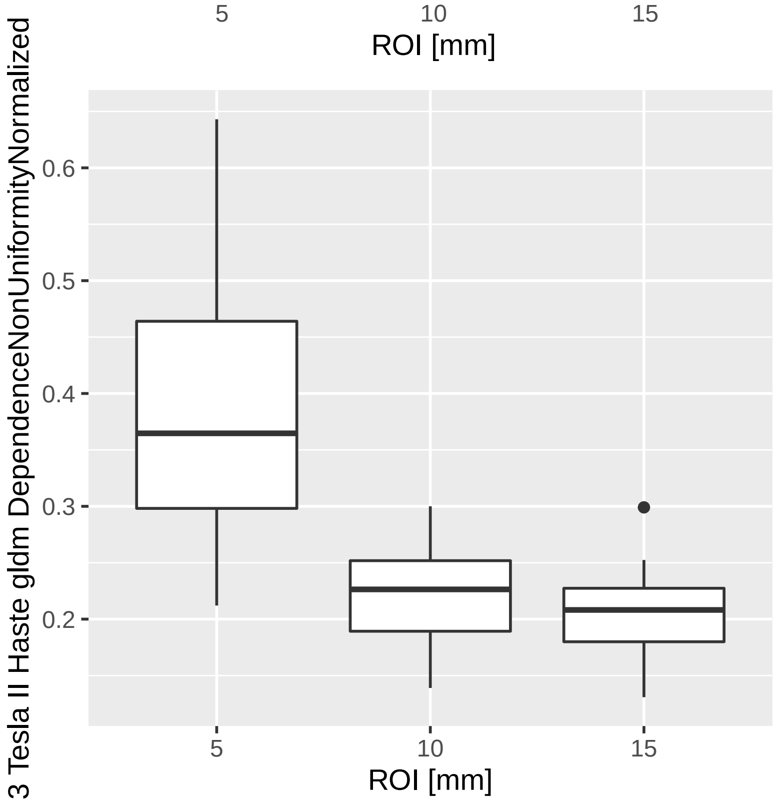
3 Tesla II Haste gldm DependenceNonUniformityNormalized
0.2
0.3
0.4
0.5
0.6
5
10
15
ROI [mm]

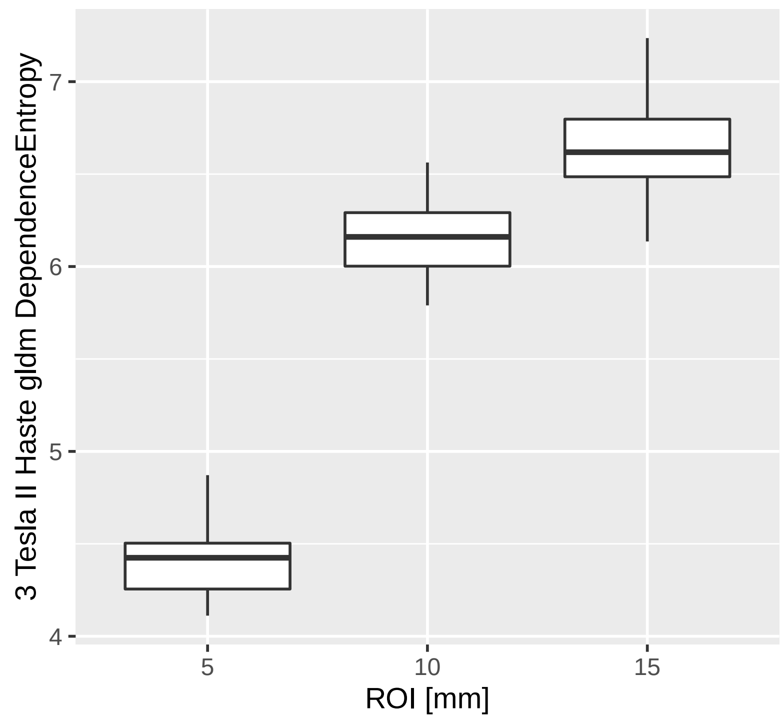
3 Tesla II Haste gldm DependenceEntropy
4
5
6
7
5
10
15
ROI [mm]

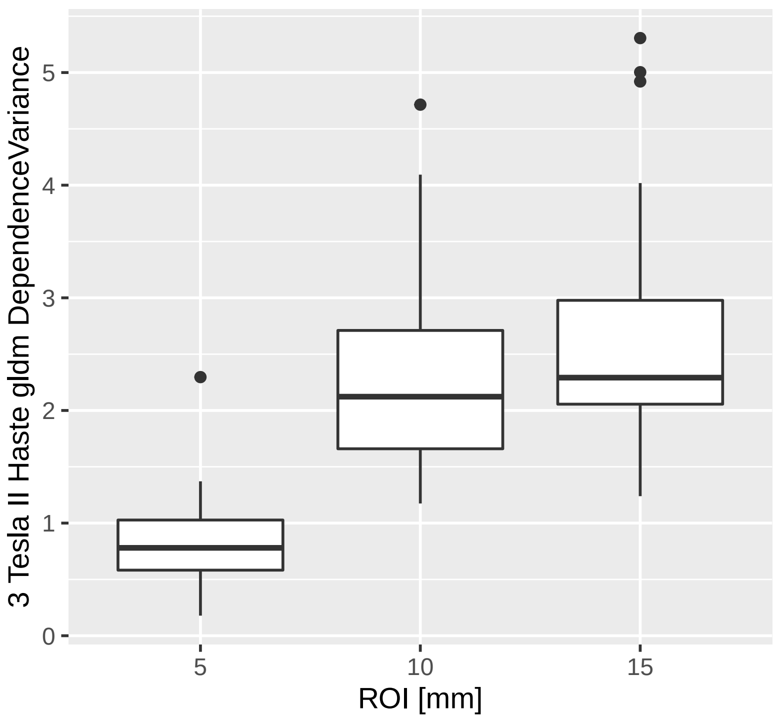
3 Tesla II Haste gldm DependenceVariance
0
1
2
3
4
5
5
10
15
ROI [mm]

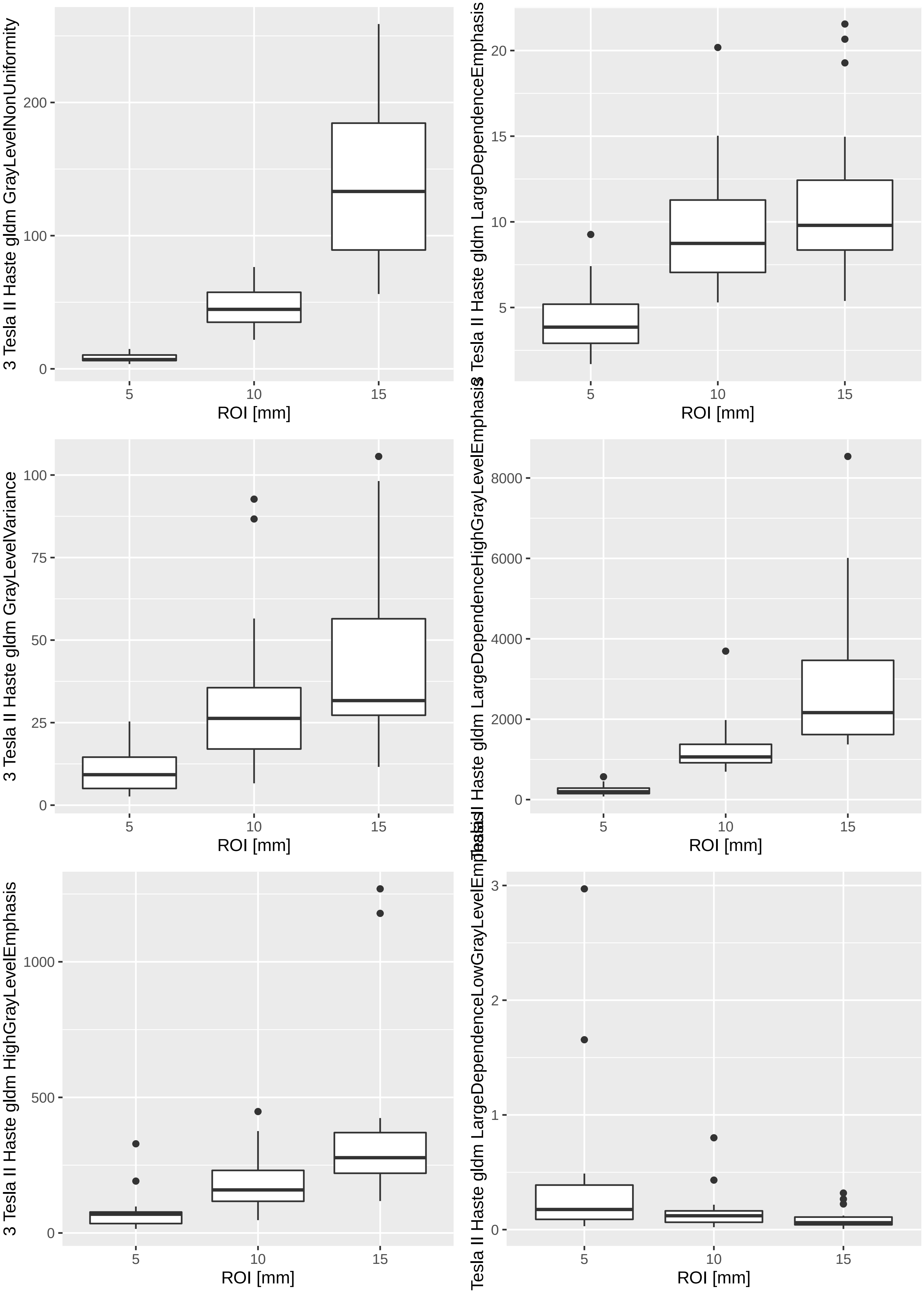
3 Tesla II Haste gldm GrayLevelNonUniformity
0
100
200
5
10
15
ROI [mm]
3 Tesla II Haste gldm LargeDependenceEmphasis
5
10
15
20
5
10
15
ROI [mm]
3 Tesla II Haste gldm GrayLevelVariance
0
25
50
75
100
5
10
15
ROI [mm]
3 Tesla II Haste gldm LargeDependenceHighGrayLevelEmphasis
0
2000
4000
6000
8000
5
10
15
ROI [mm]
3 Tesla II Haste gldm HighGrayLevelEmphasis
0
500
1000
5
10
15
ROI [mm]
3 Tesla II Haste gldm LargeDependenceLowGrayLevelEmphasis
0
1
2
3
5
10
15
ROI [mm]

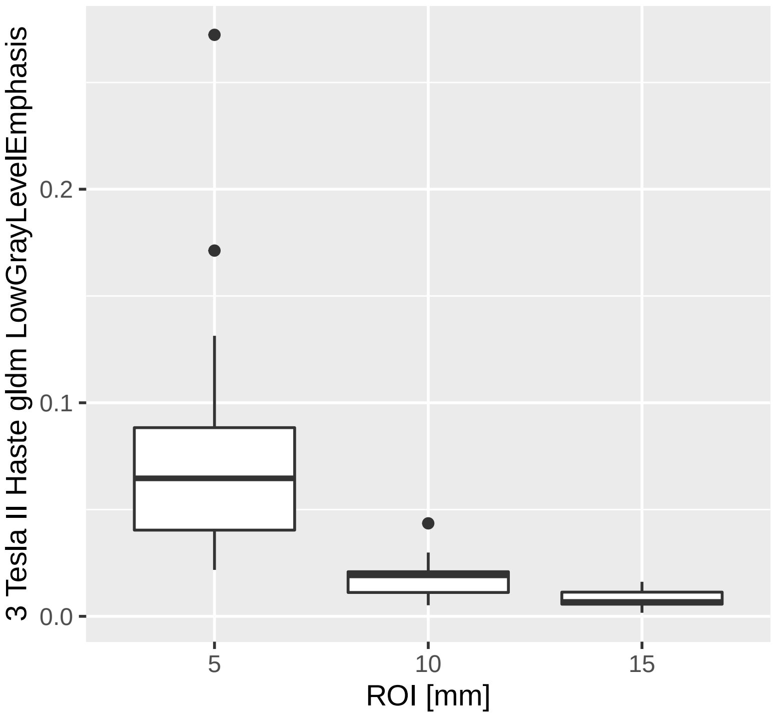
3 Tesla II Haste gldm LowGrayLevelEmphasis
0.0
0.1
0.2
5
10
15
ROI [mm]

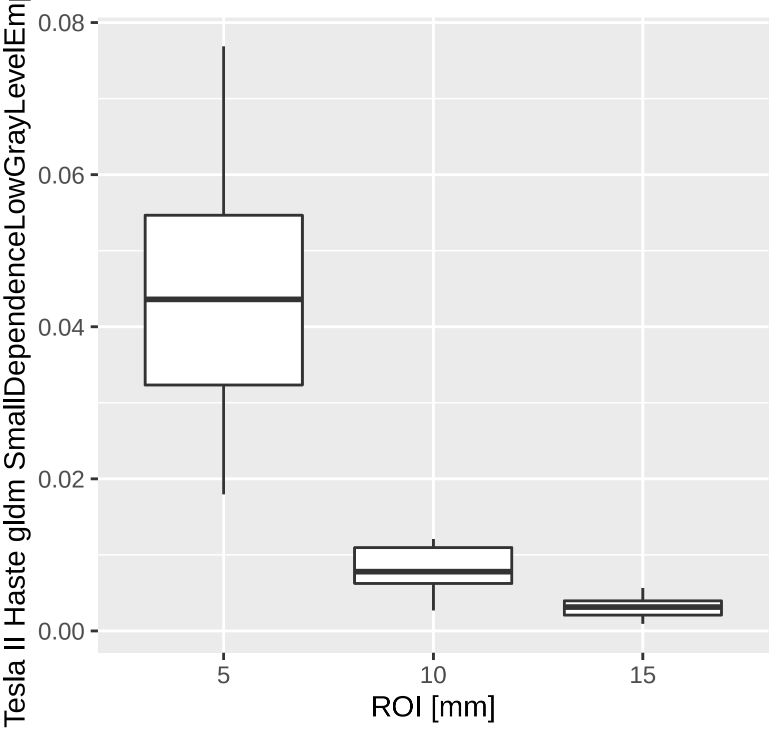
Tesla II Haste gldm SmallDependenceLowGrayLevelEmpha
0.00
0.02
0.04
0.06
0.08
5
10
15
ROI [mm]

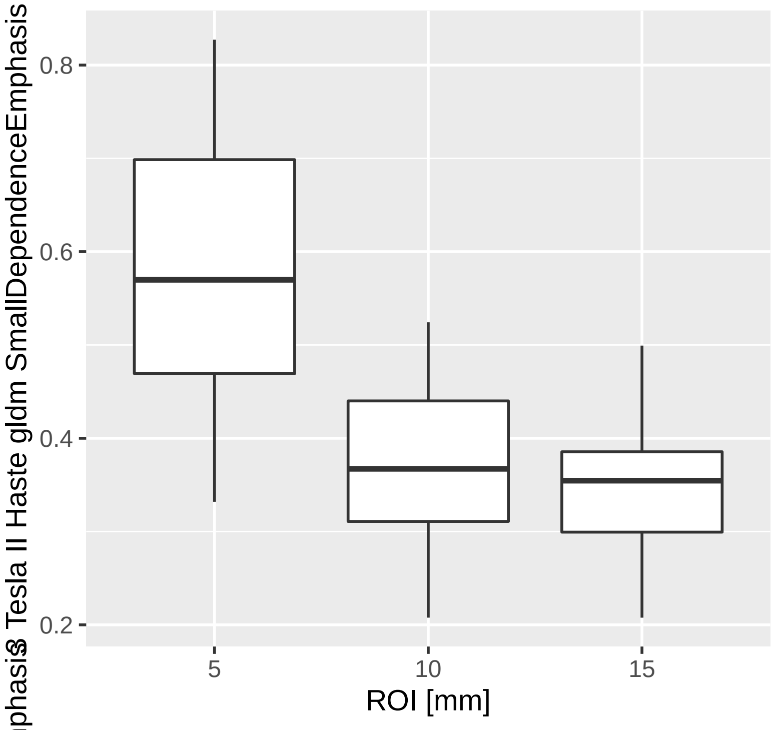
3 Tesla II Haste gldm SmallDependenceEmphasis
0.2
0.4
0.6
0.8
5
10
15
ROI [mm]

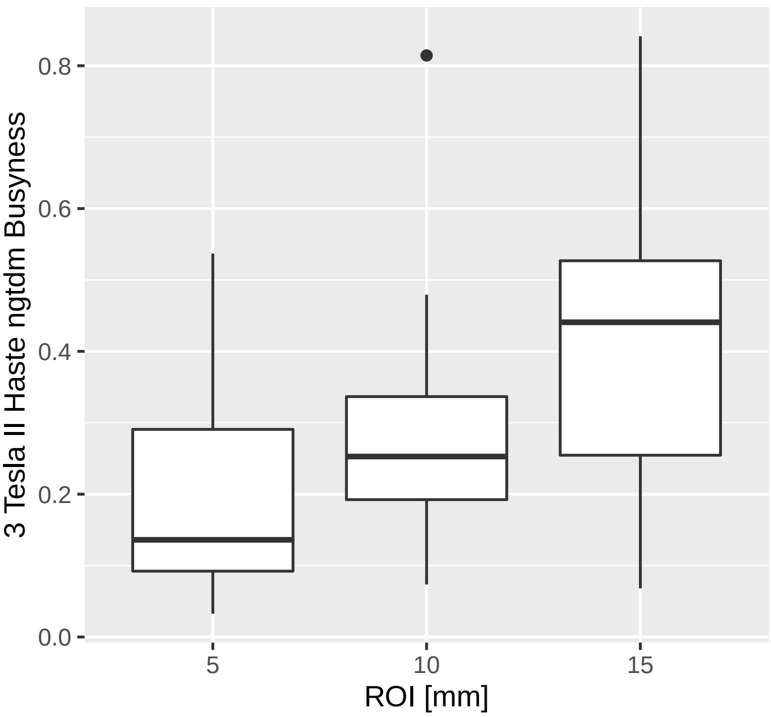
3 Tesla II Haste ngtdm Busyness
0.0
0.2
0.4
0.6
0.8
5
10
15
ROI [mm]

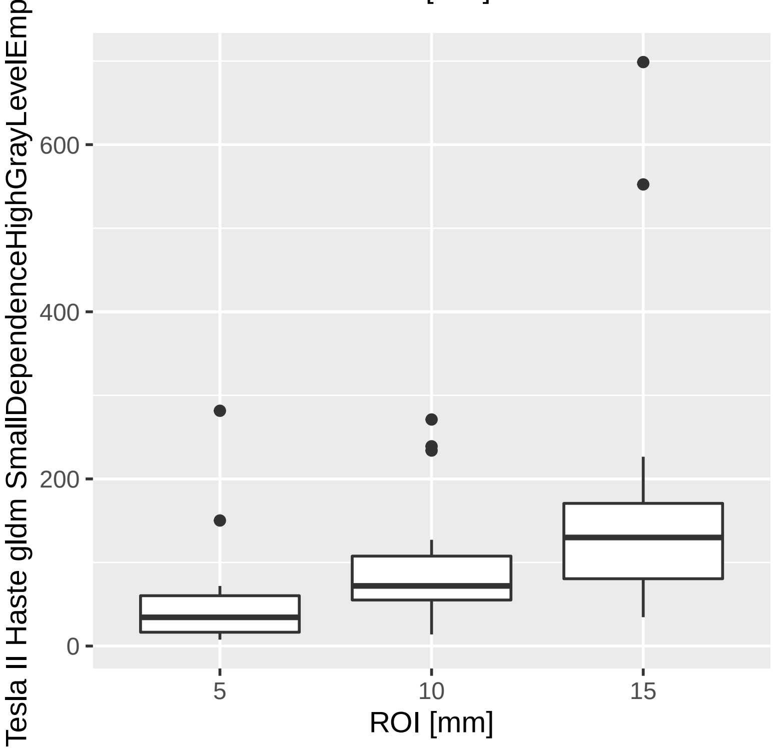
Tesla II Haste gldm SmallDependenceHighGrayLevelEmphasis
0
200
400
600
5
10
15
ROI [mm]

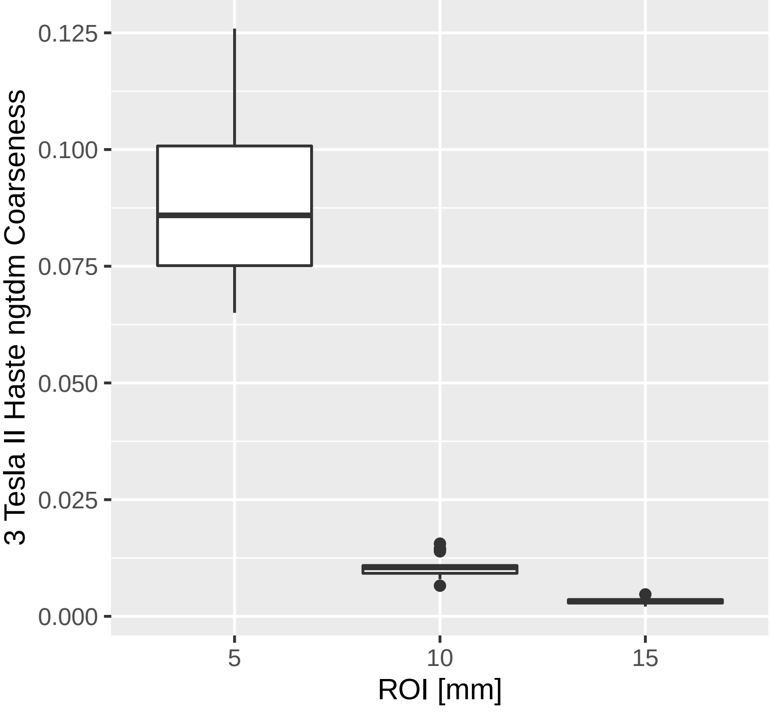
3 Tesla II Haste ngtdm Coarseness
0.000
0.025
0.050
0.075
0.100
0.125
5
10
15
ROI [mm]

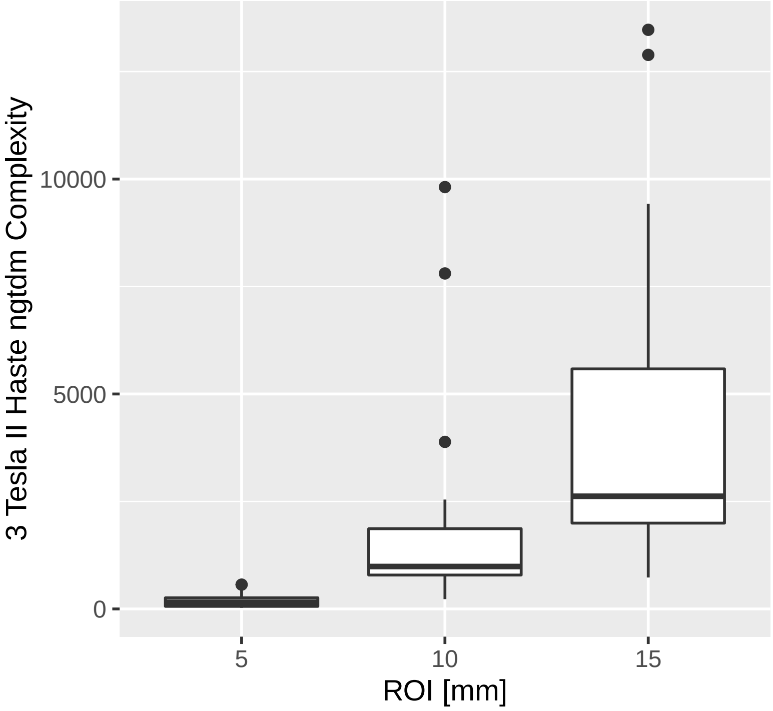
3 Tesla II Haste ngtdm Complexity
10000
5000
0
5
10
15
ROI [mm]

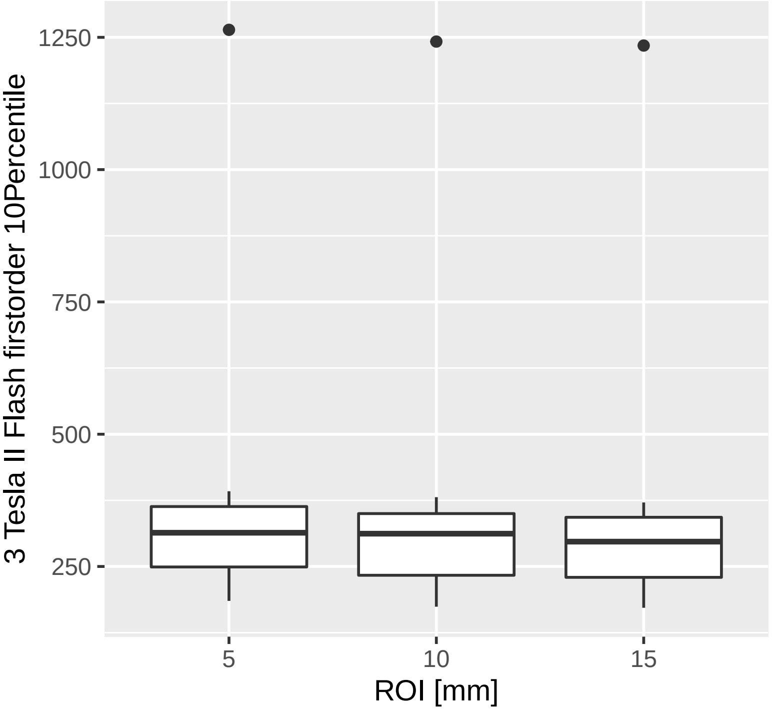
3 Tesla II Flash firstorder 10Percentile
1250
1000
750
500
250
5
10
15
ROI [mm]

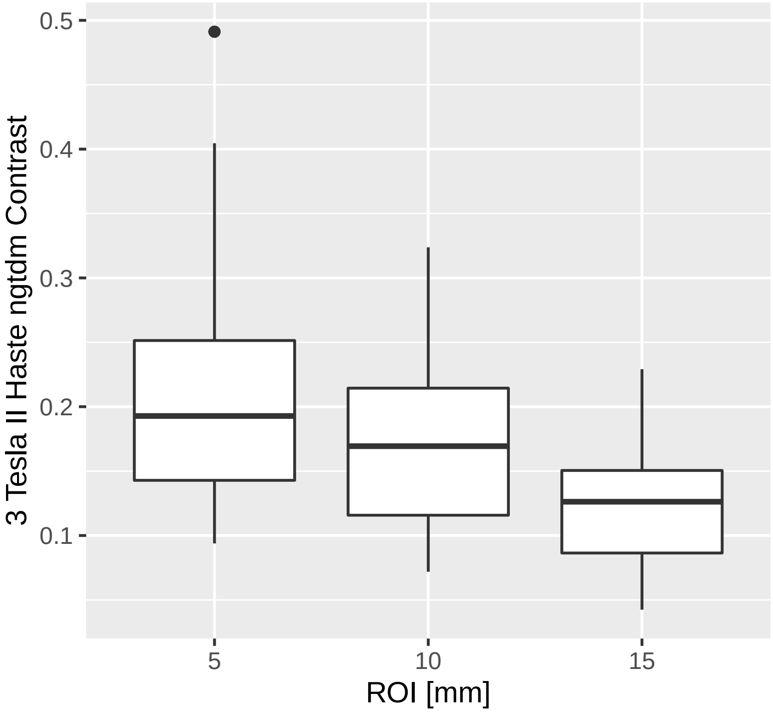
3 Tesla II Haste ngtdm Contrast
0.5
0.4
0.3
0.2
0.1
5
10
15
ROI [mm]

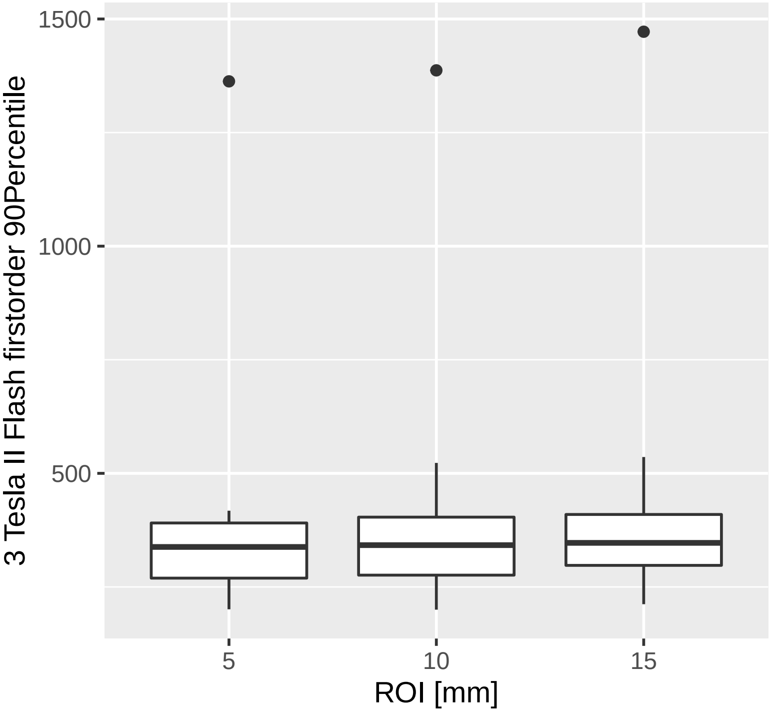
3 Tesla II Flash firstorder 90Percentile
1500
1000
500
5
10
15
ROI [mm]

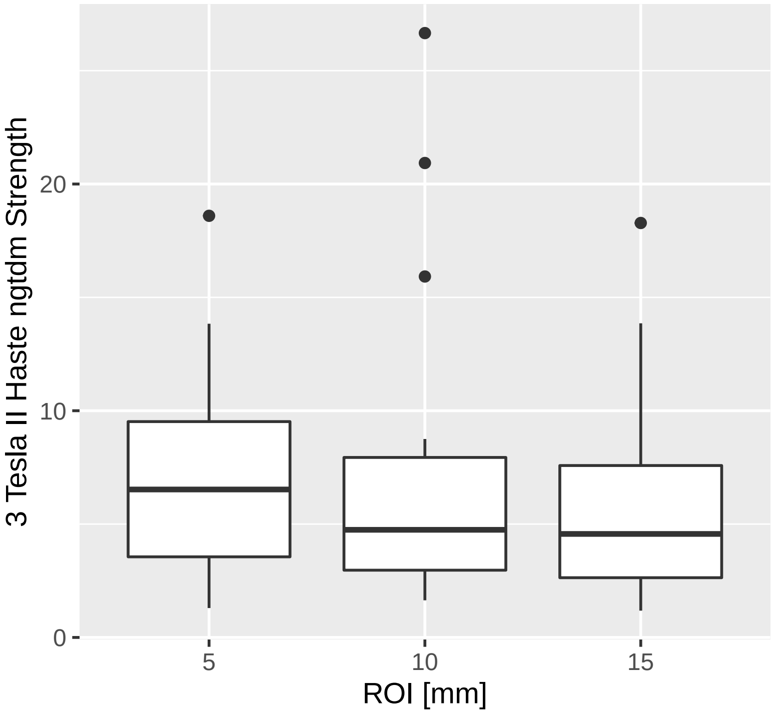
3 Tesla II Haste ngtdm Strength
20
10
0
5
10
15
ROI [mm]

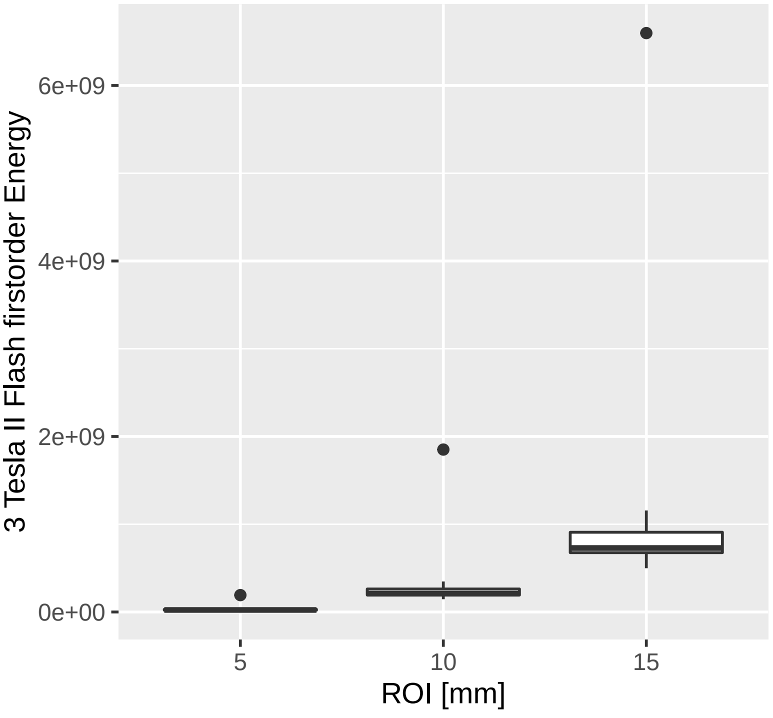
3 Tesla II Flash firstorder Energy
6e+09
4e+09
2e+09
0e+00
5
10
15
ROI [mm]

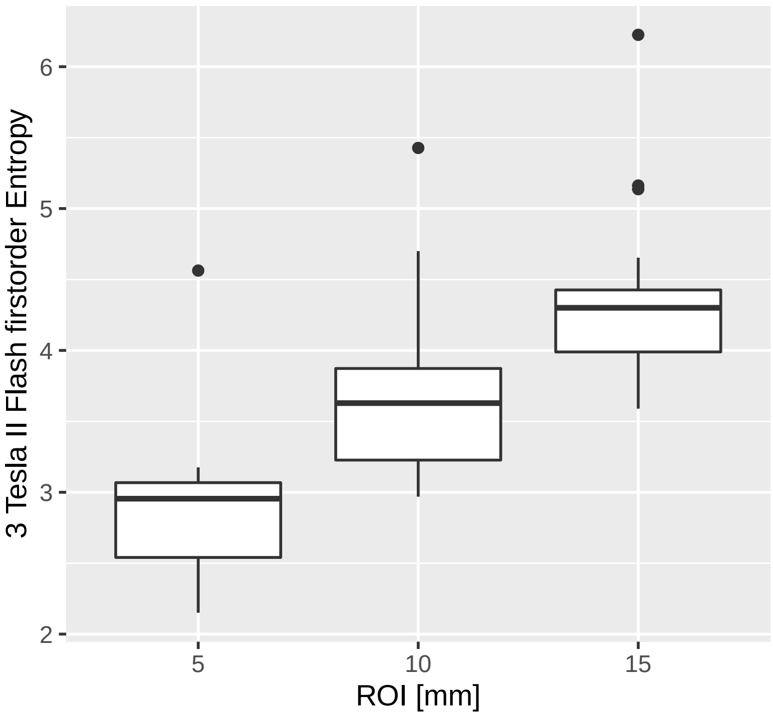
3 Tesla II Flash firstorder Entropy
2
3
4
5
6
5
10
15
ROI [mm]

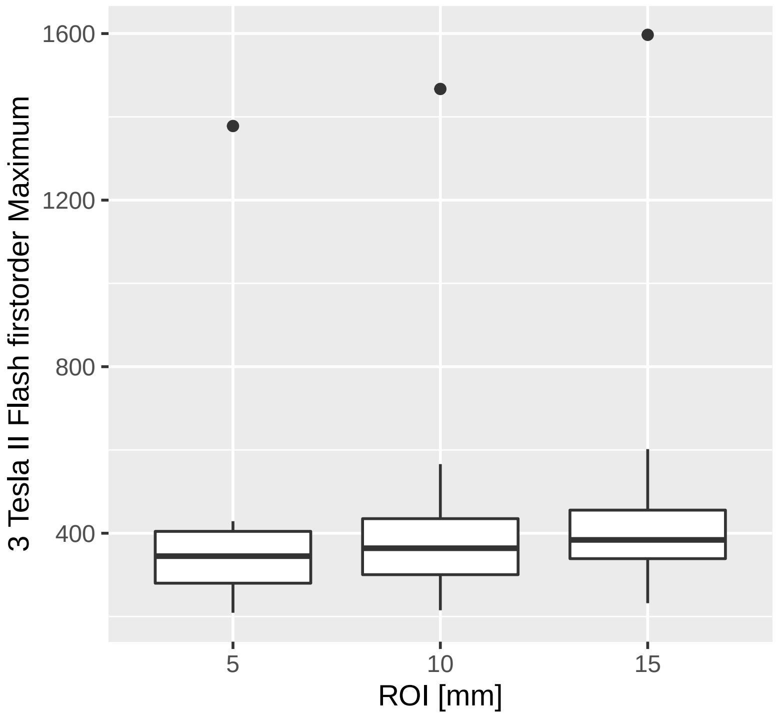
3 Tesla II Flash firstorder Maximum
400
800
1200
1600
5
10
15
ROI [mm]

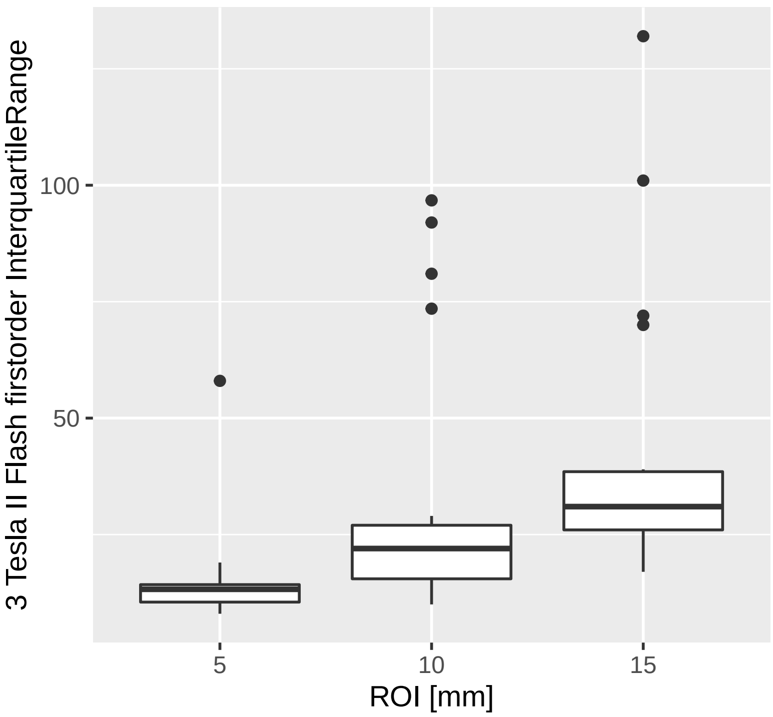
3 Tesla II Flash firstorder InterquartileRange
50
100
5
10
15
ROI [mm]

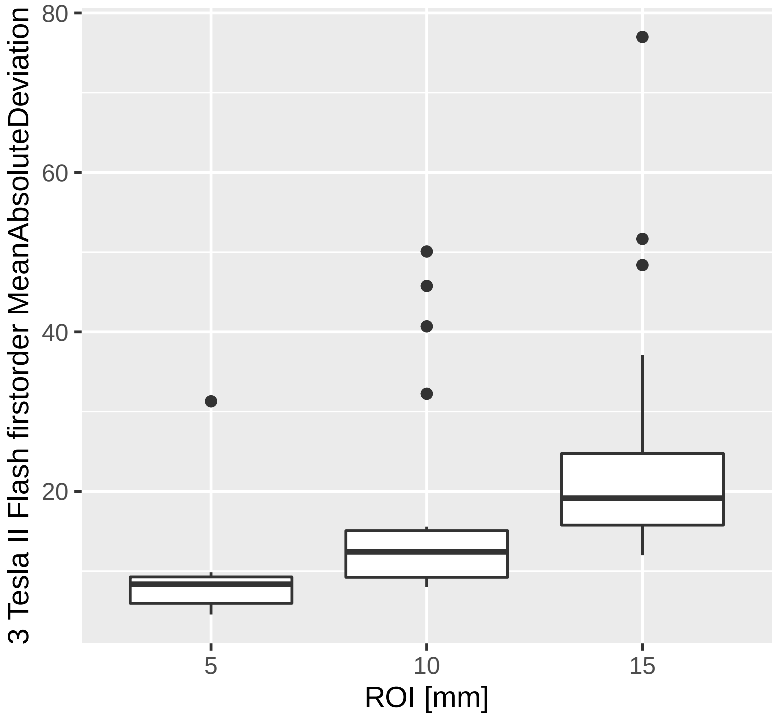
3 Tesla II Flash firstorder MeanAbsoluteDeviation
20
40
60
80
5
10
15
ROI [mm]

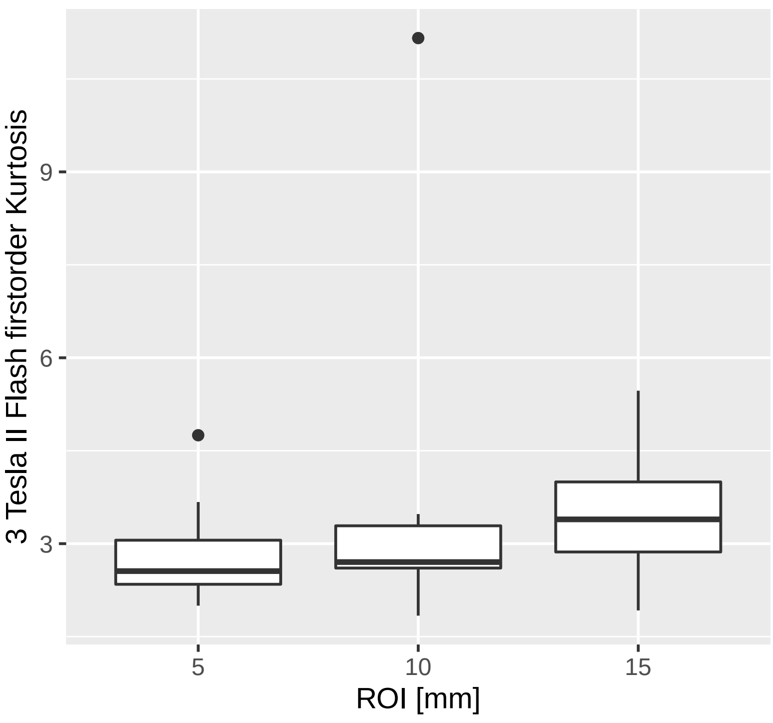
3 Tesla II Flash firstorder Kurtosis
3
6
9
5
10
15
ROI [mm]

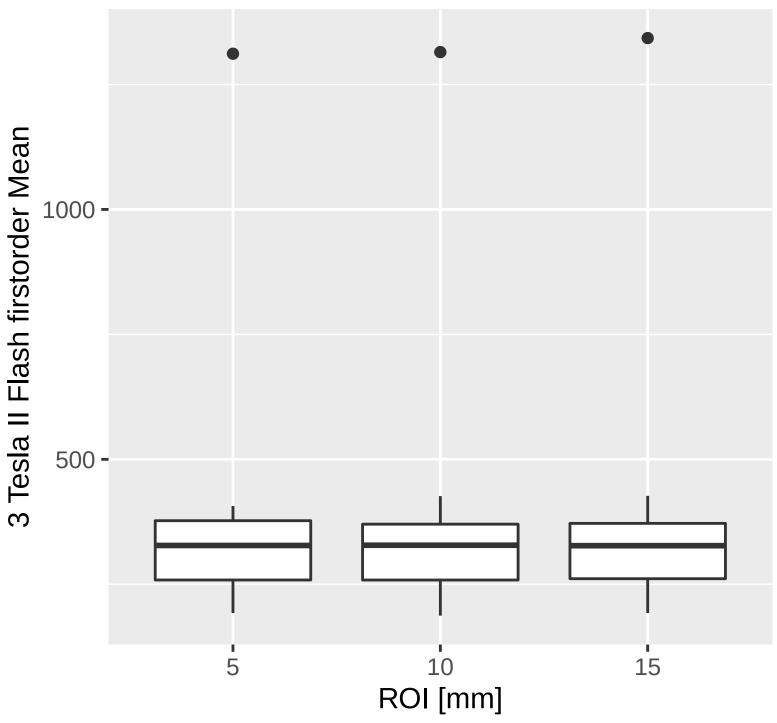
3 Tesla II Flash firstorder Mean
500
1000
5
10
15
ROI [mm]

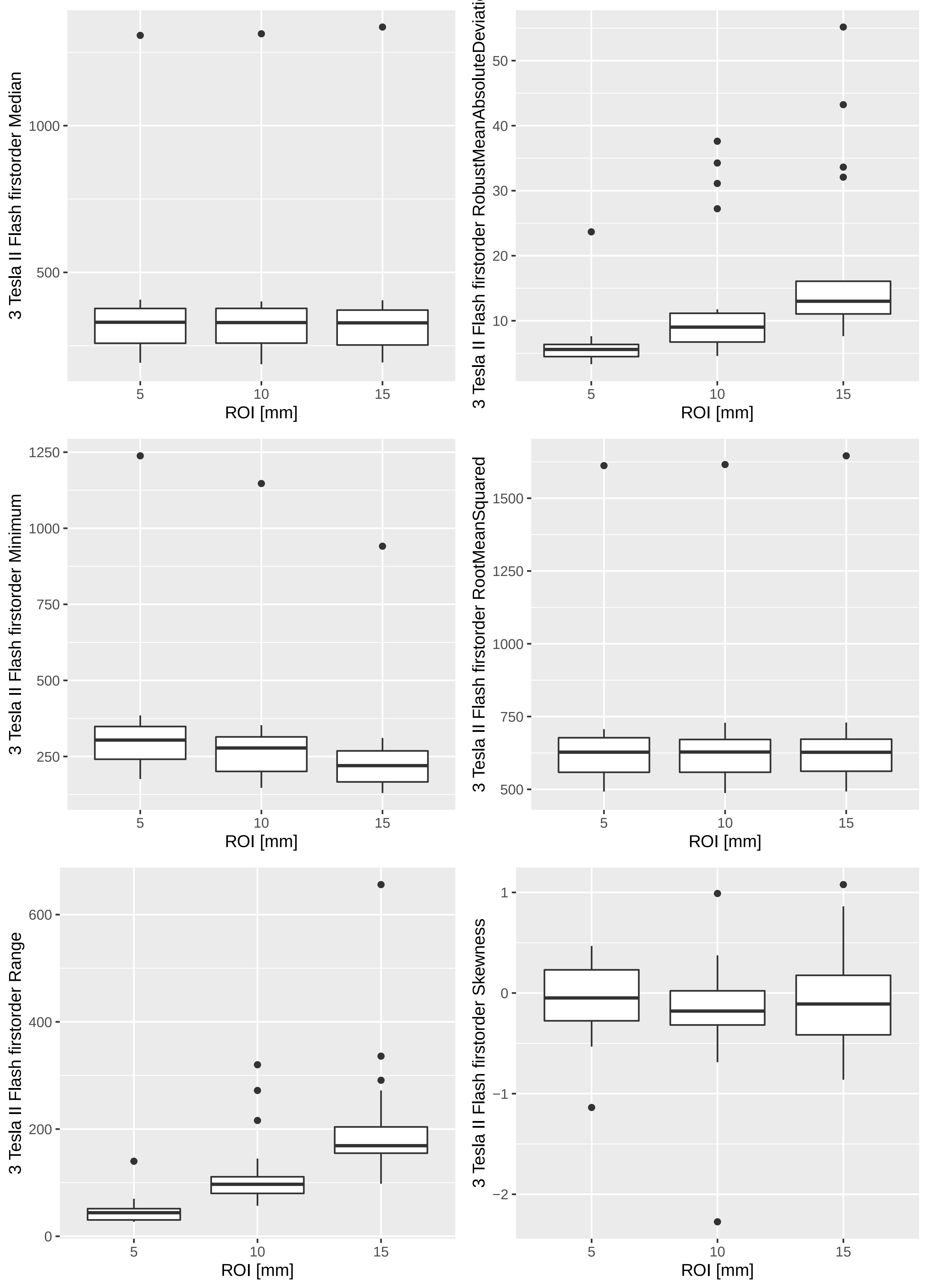
3 Tesla II Flash firstorder Median
ROI [mm]
3 Tesla II Flash firstorder RobustMeanAbsoluteDeviation
ROI [mm]
3 Tesla II Flash firstorder Minimum
ROI [mm]
3 Tesla II Flash firstorder RootMeanSquared
ROI [mm]
3 Tesla II Flash firstorder Range
ROI [mm]
3 Tesla II Flash firstorder Skewness
ROI [mm]

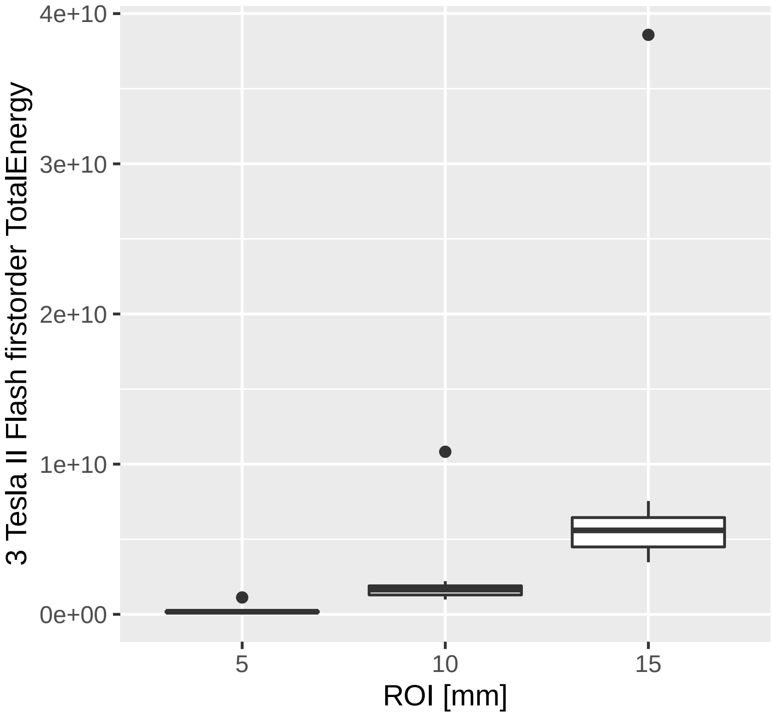
3 Tesla II Flash firstorder TotalEnergy
4e+10
3e+10
2e+10
1e+10
0e+00
5
10
15
ROI [mm]

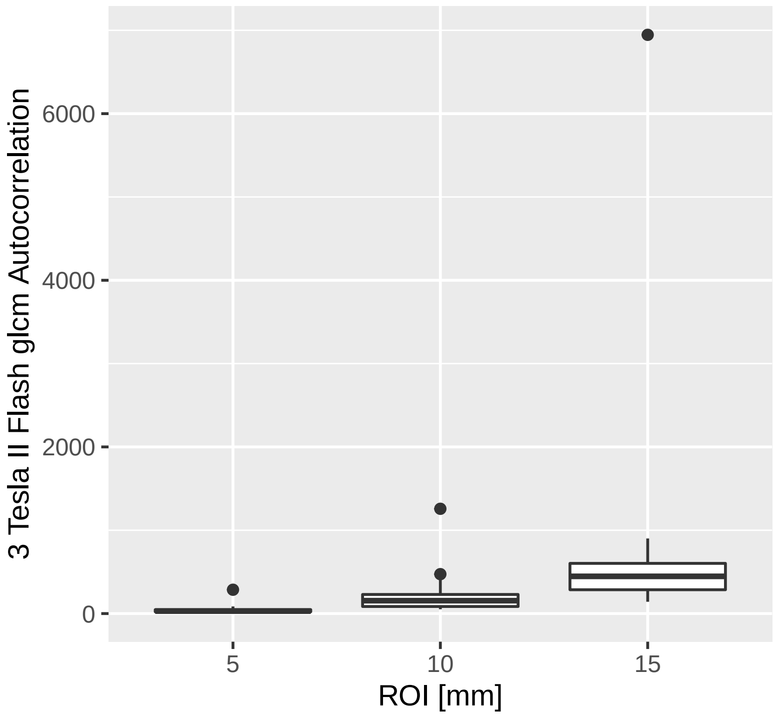
3 Tesla II Flash glcm Autocorrelation
6000
4000
2000
0
5
10
15
ROI [mm]

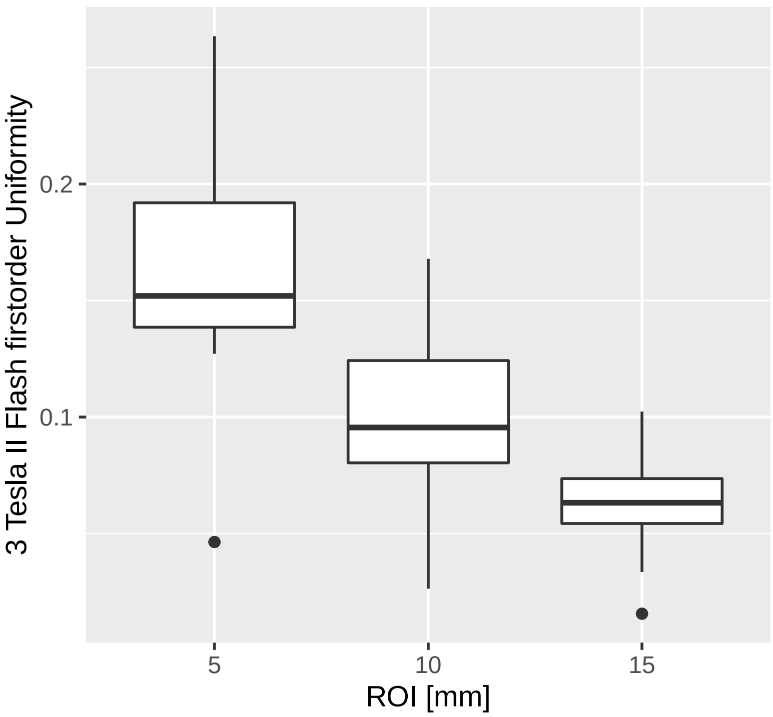
3 Tesla II Flash firstorder Uniformity
0.2
0.1
5
10
15
ROI [mm]

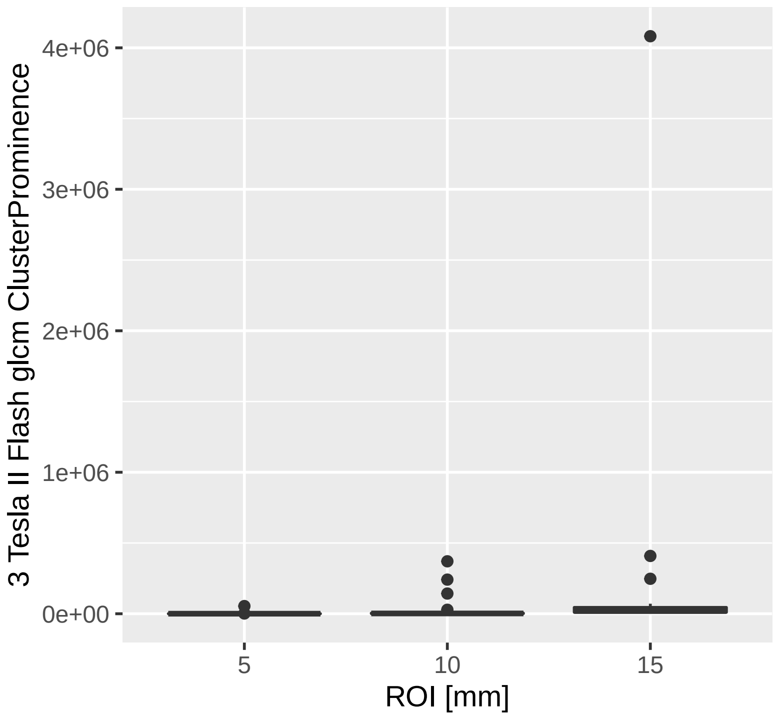
3 Tesla II Flash glcm ClusterProminence
4e+06
3e+06
2e+06
1e+06
0e+00
5
10
15
ROI [mm]

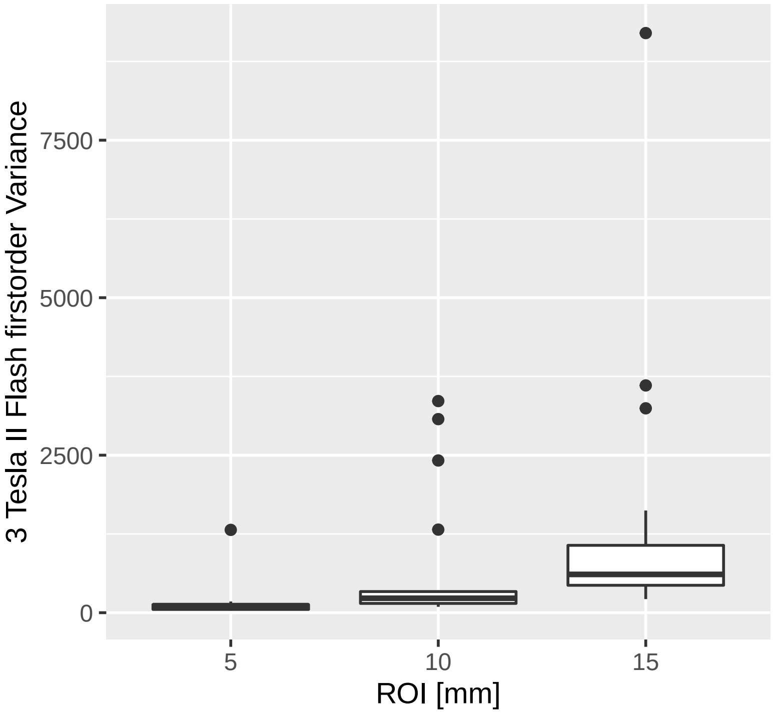
3 Tesla II Flash firstorder Variance
7500
5000
2500
0
5
10
15
ROI [mm]

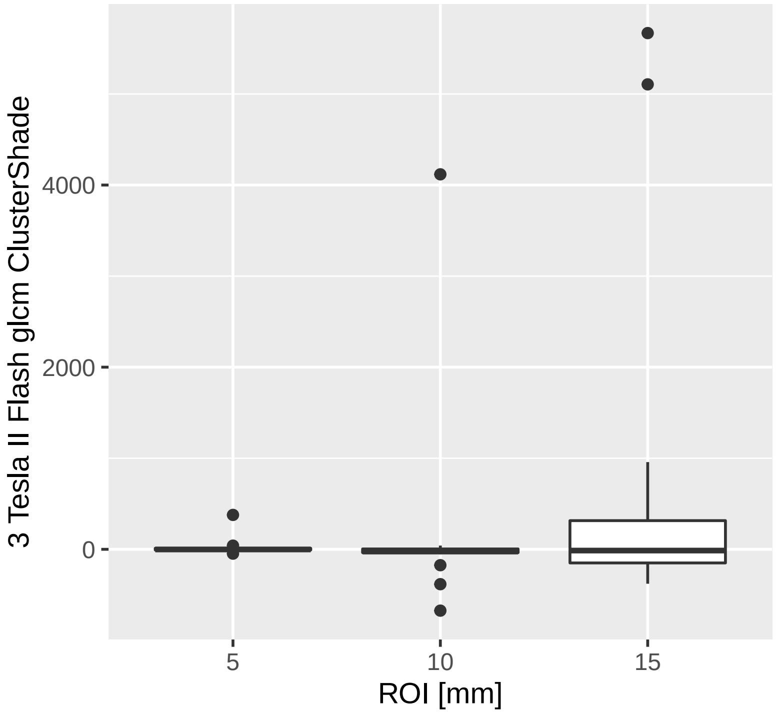
3 Tesla II Flash glcm ClusterShade
4000
2000
0
5
10
15
ROI [mm]

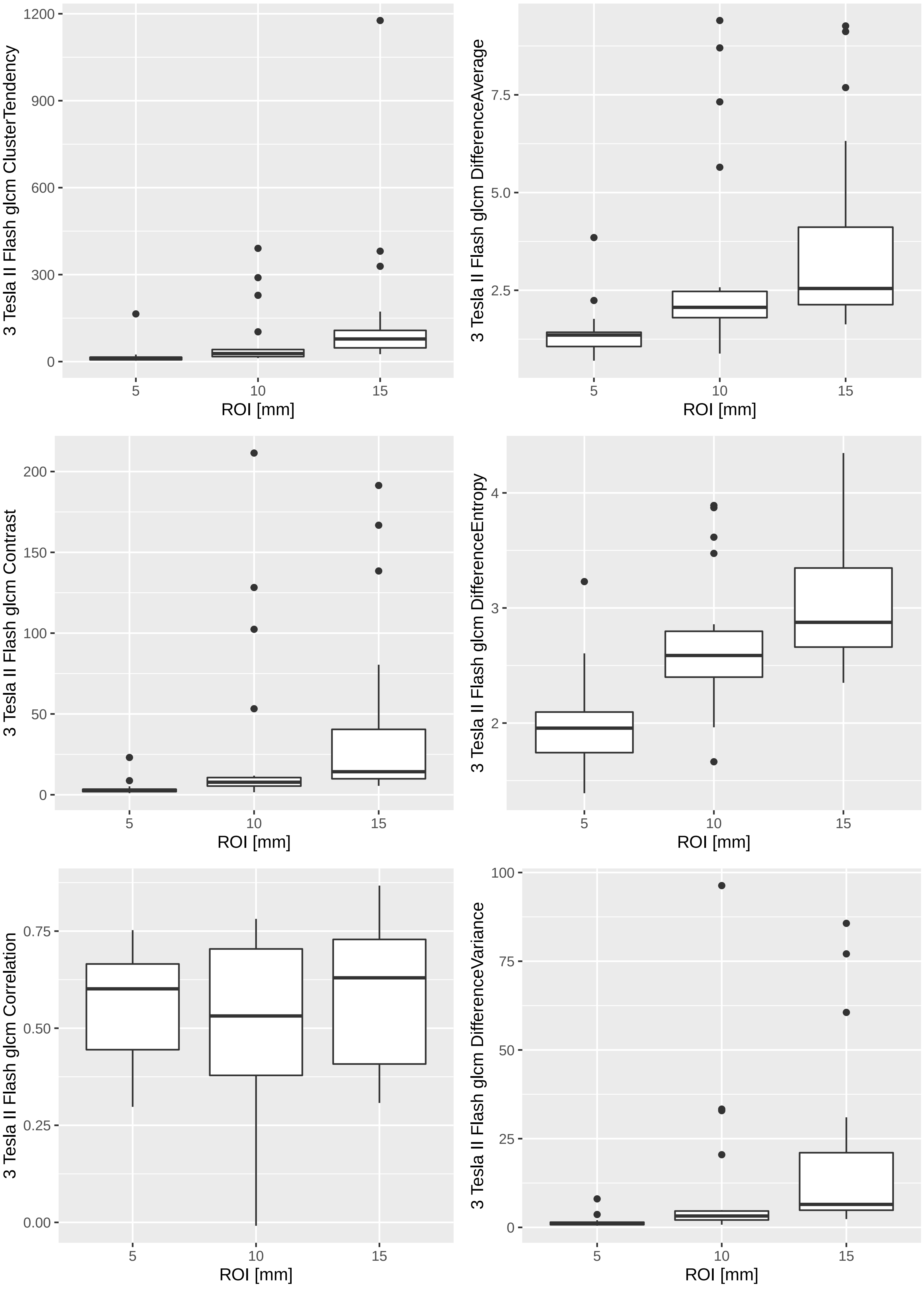

3 Tesla II Flash glcm ClusterTendency
0
300
600
900
1200
5
10
15
ROI [mm]
3 Tesla II Flash glcm DifferenceAverage
2.5
5.0
7.5
5
10
15
ROI [mm]
3 Tesla II Flash glcm Contrast
0
50
100
150
200
5
10
15
ROI [mm]
3 Tesla II Flash glcm DifferenceEntropy
2
3
4
5
10
15
ROI [mm]
3 Tesla II Flash glcm Correlation
0.00
0.25
0.50
0.75
5
10
15
ROI [mm]
3 Tesla II Flash glcm DifferenceVariance
0
25
50
75
100
5
10
15
ROI [mm]

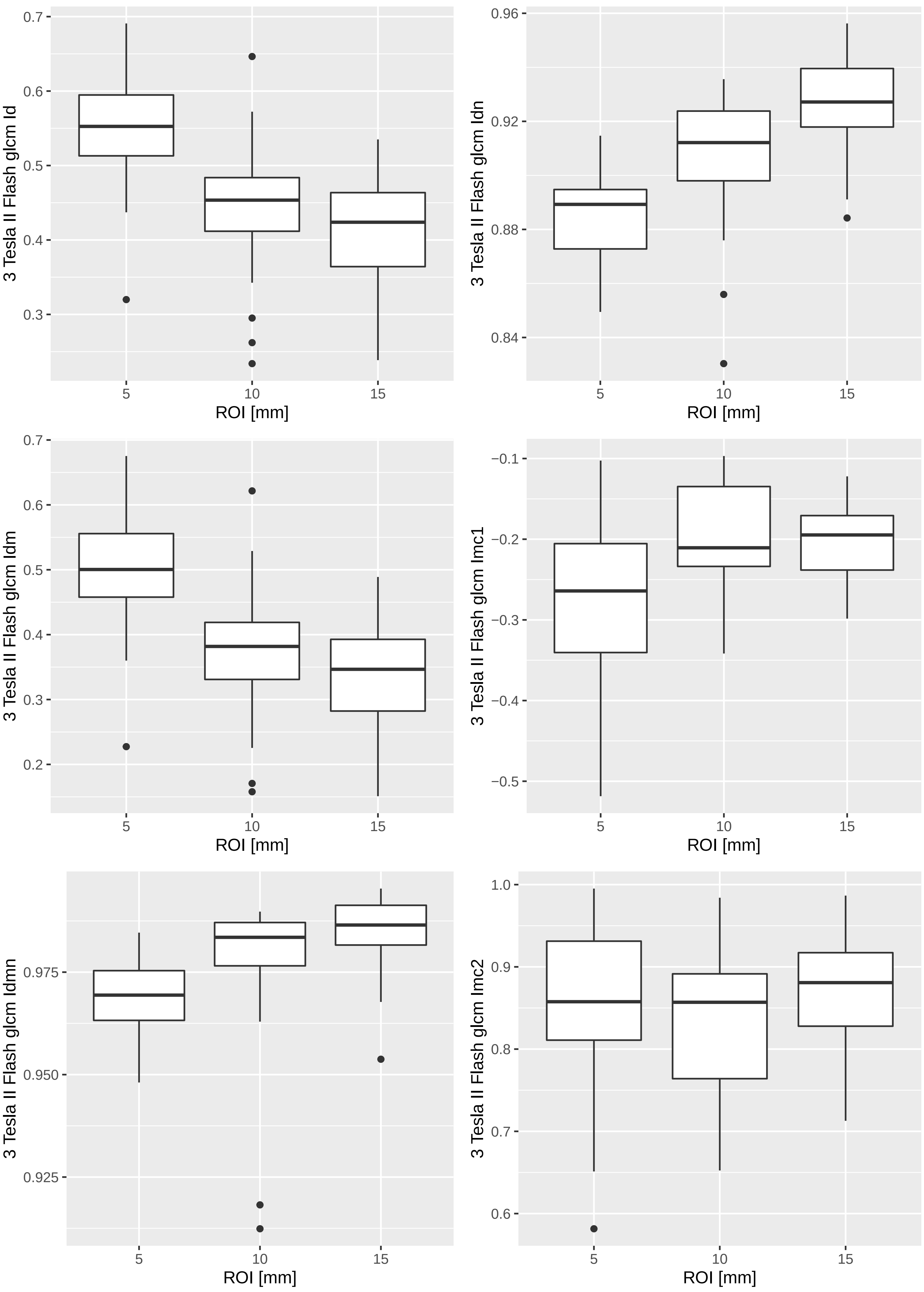

3 Tesla II Flash glcm Id
ROI [mm]
3 Tesla II Flash glcm Idn
ROI [mm]
3 Tesla II Flash glcm Idm
ROI [mm]
3 Tesla II Flash glcm Imc1
ROI [mm]
3 Tesla II Flash glcm Idmn
ROI [mm]
3 Tesla II Flash glcm Imc2
ROI [mm]

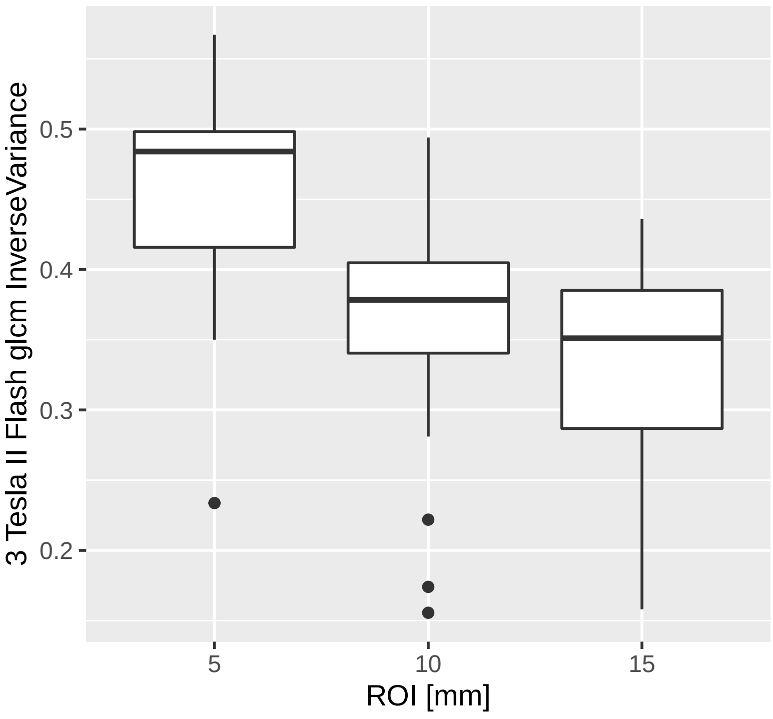

3 Tesla II Flash glcm InverseVariance
0.5
0.4
0.3
0.2
5
10
15
ROI [mm]

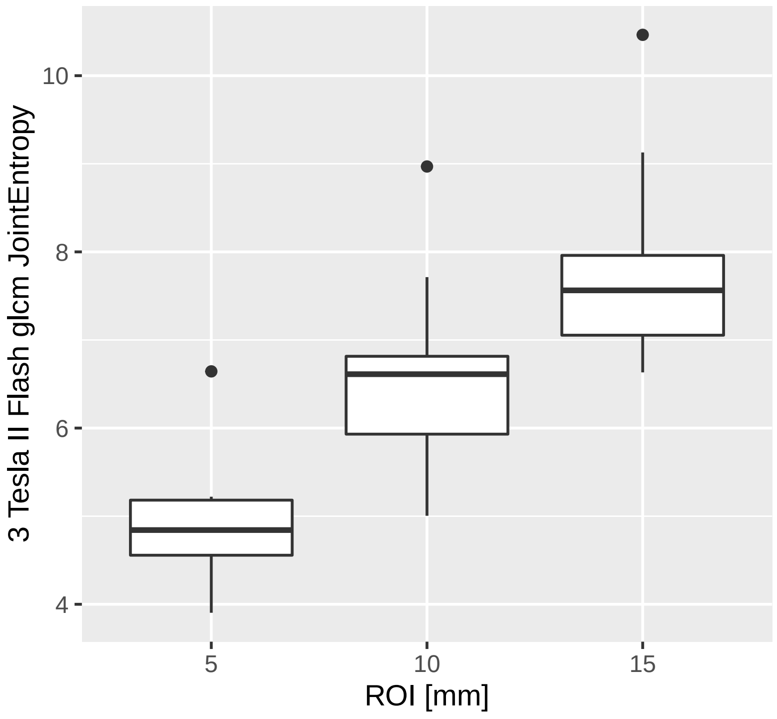

3 Tesla II Flash glcm JointEntropy
10
8
6
4
5
10
15
ROI [mm]

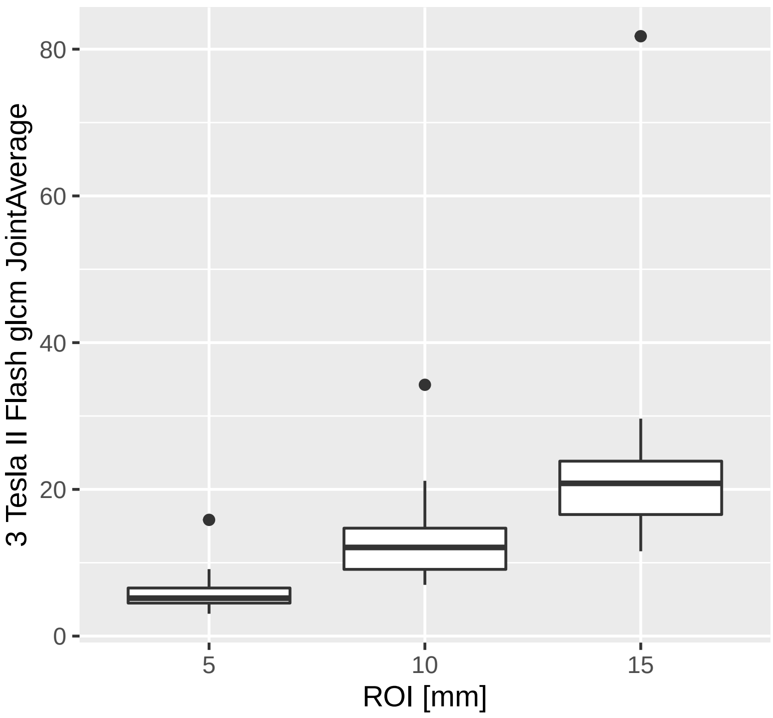

3 Tesla II Flash glcm JointAverage
80
60
40
20
0
5
10
15
ROI [mm]

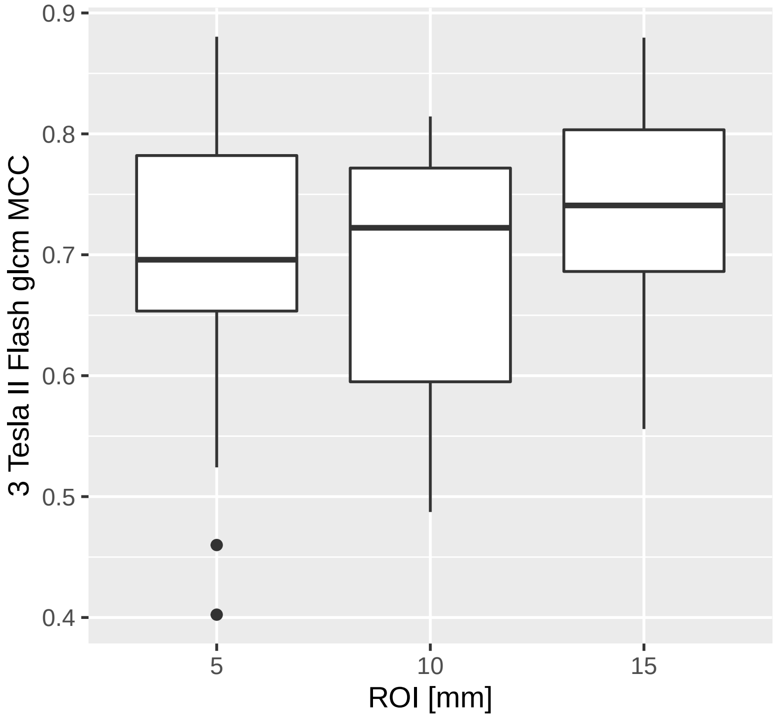

3 Tesla II Flash glcm MCC
0.9
0.8
0.7
0.6
0.5
0.4
5
10
15
ROI [mm]

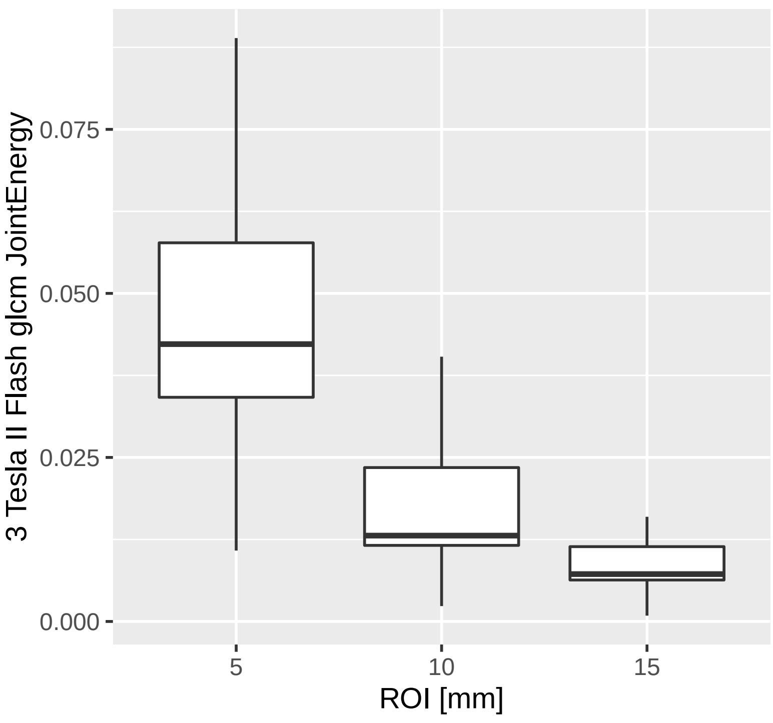

3 Tesla II Flash glcm JointEnergy
0.075
0.050
0.025
0.000
5
10
15
ROI [mm]

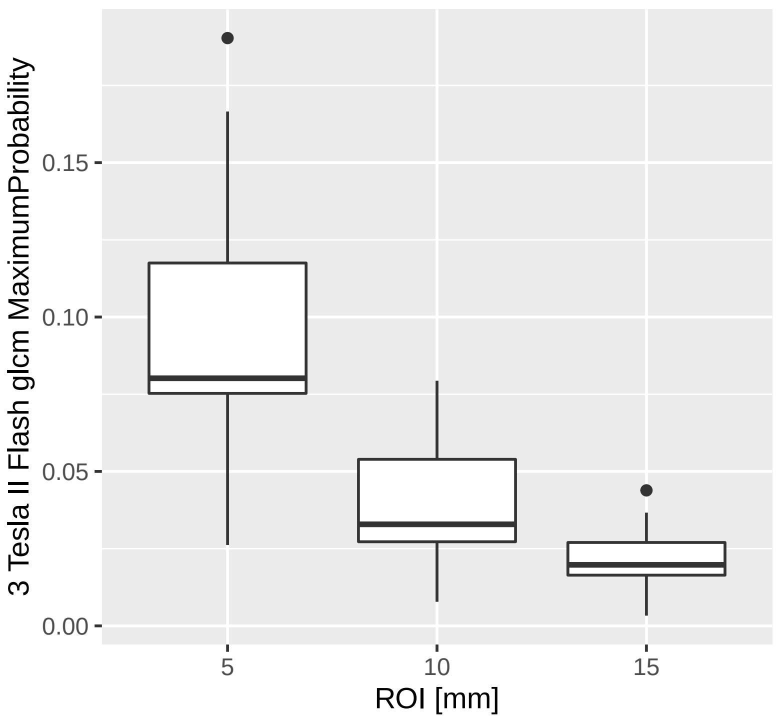

3 Tesla II Flash glcm MaximumProbability
0.15
0.10
0.05
0.00
5
10
15
ROI [mm]

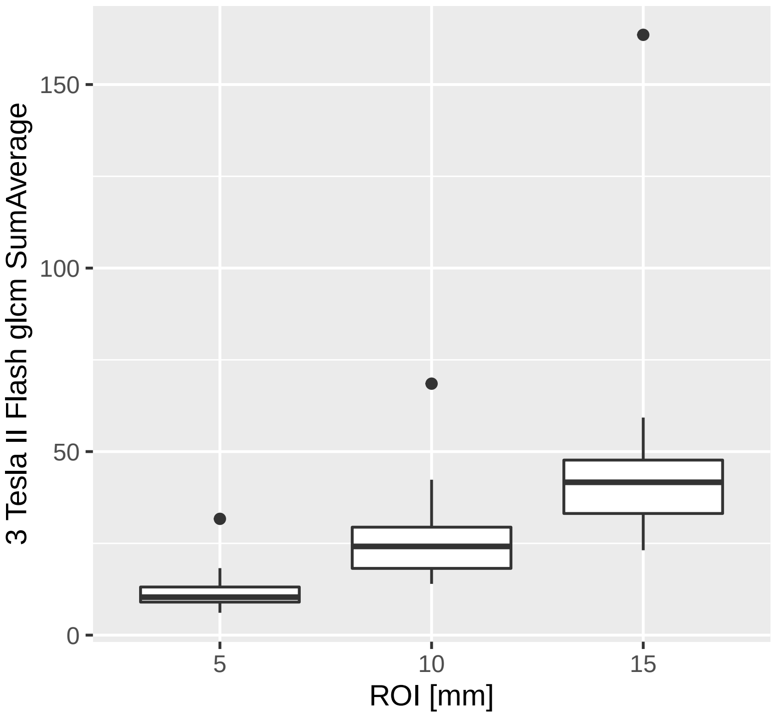
3 Tesla II Flash glcm SumAverage
0
50
100
150
5
10
15
ROI [mm]

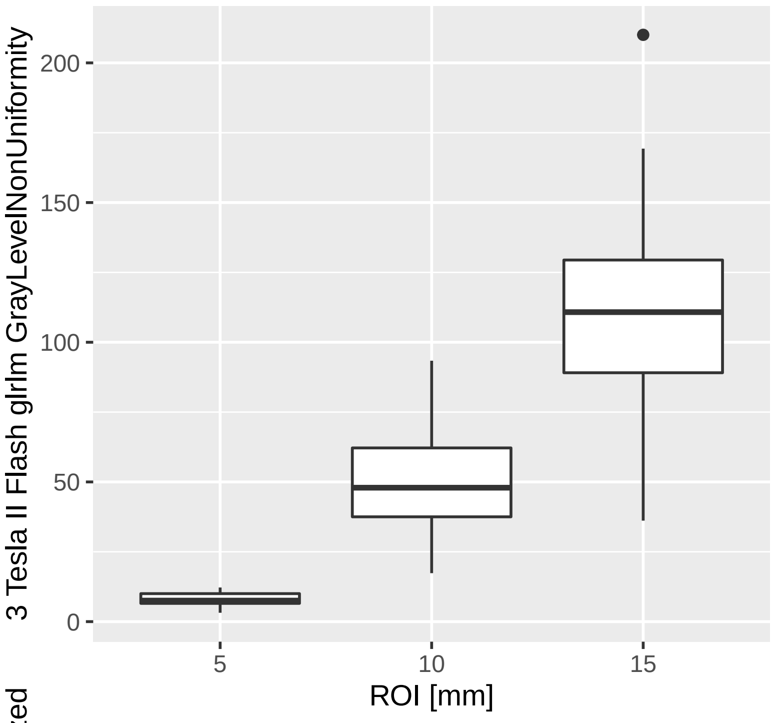
3 Tesla II Flash glrlm GrayLevelNonUniformity
0
50
100
150
200
5
10
15
ROI [mm]

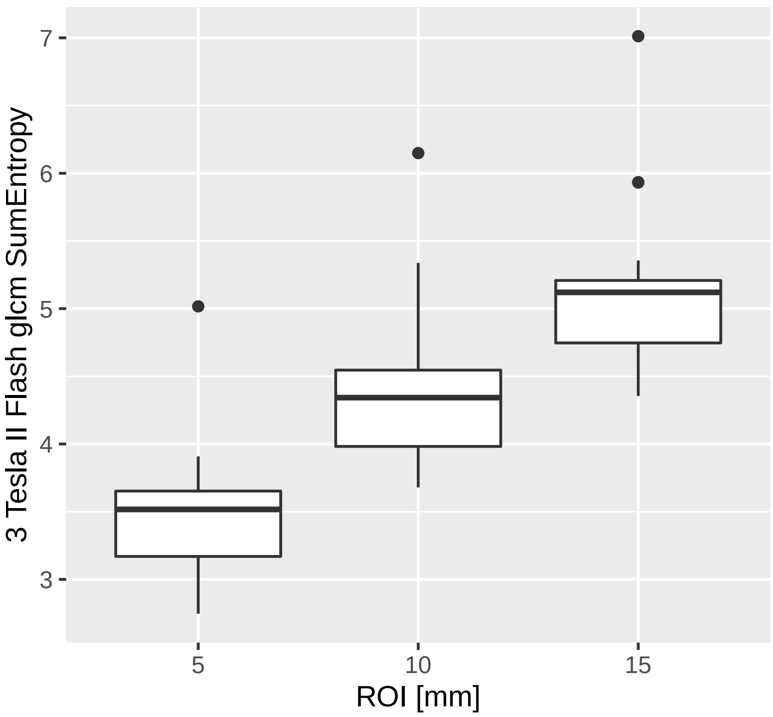
3 Tesla II Flash glcm SumEntropy
3
4
5
6
7
5
10
15
ROI [mm]

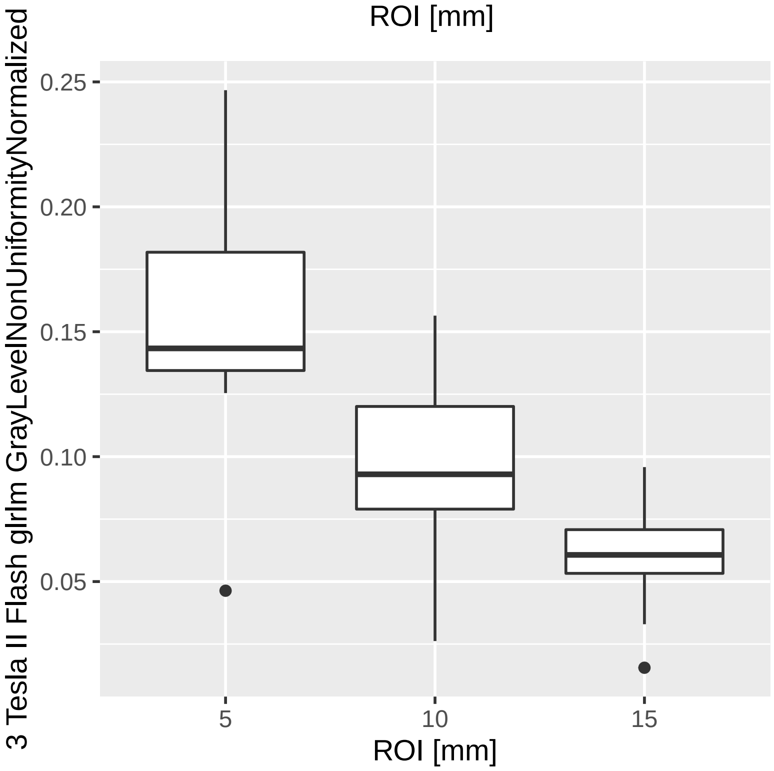
3 Tesla II Flash glrlm GrayLevelNonUniformityNormalized
0.05
0.10
0.15
0.20
0.25
5
10
15
ROI [mm]

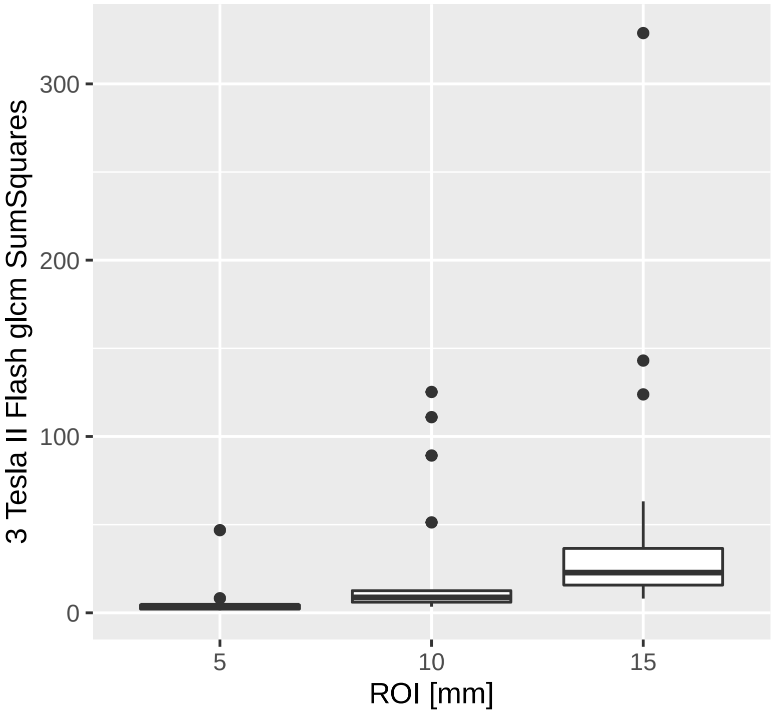
3 Tesla II Flash glcm SumSquares
0
100
200
300
5
10
15
ROI [mm]

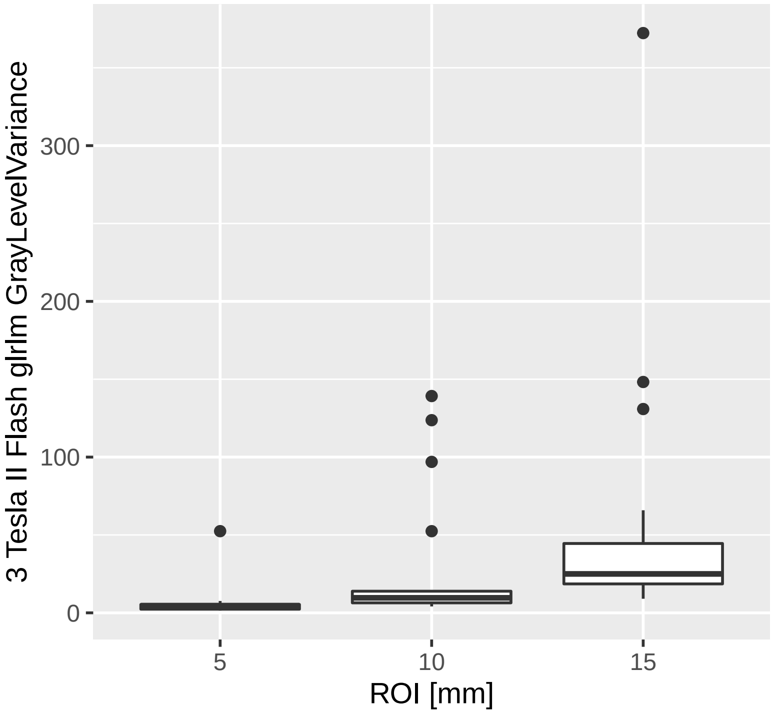
3 Tesla II Flash glrlm GrayLevelVariance
0
100
200
300
5
10
15
ROI [mm]

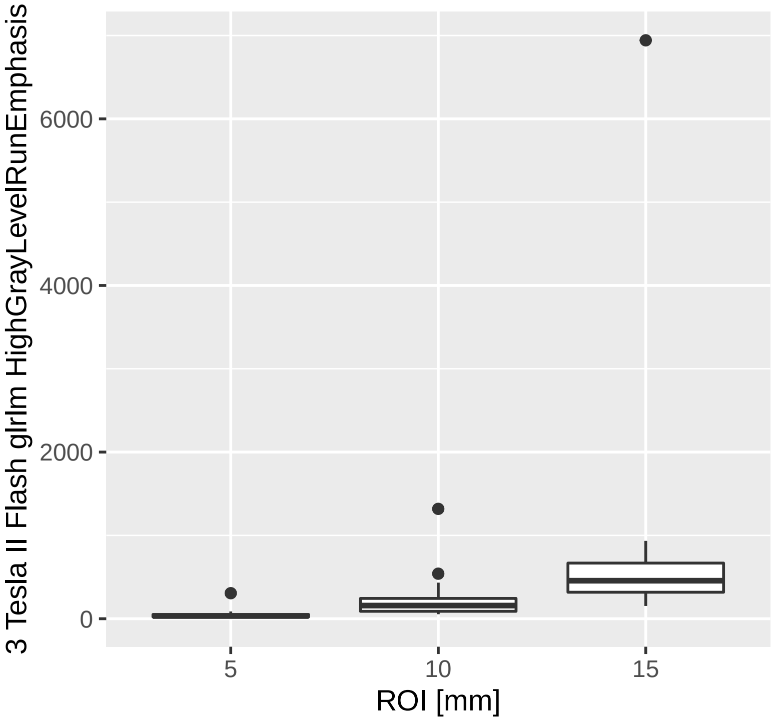
3 Tesla II Flash glrlm HighGrayLevelRunEmphasis
0
2000
4000
6000
5
10
15
ROI [mm]

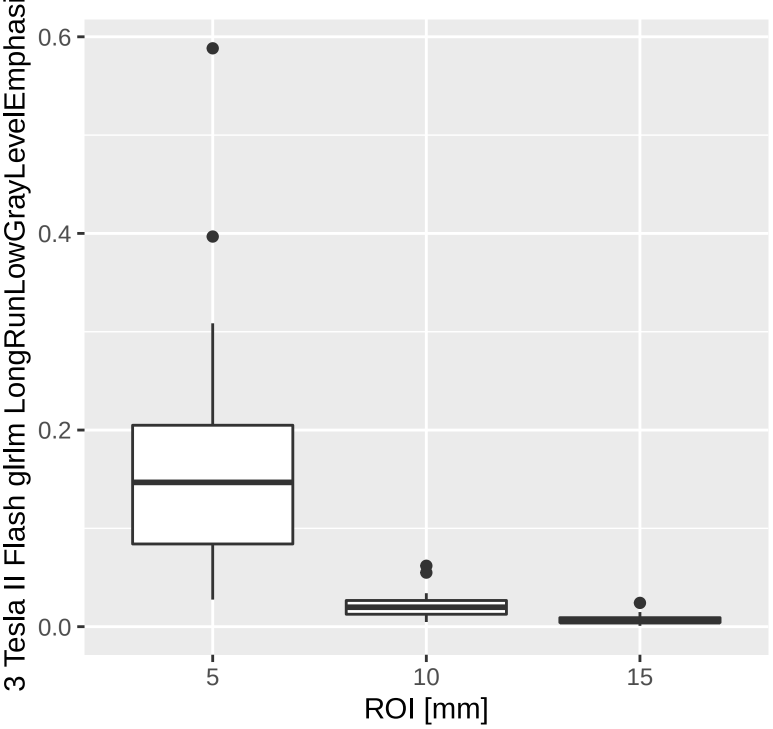
3 Tesla II Flash glrlm LongRunLowGrayLevelEmphasis
0.0
0.2
0.4
0.6
5
10
15
ROI [mm]

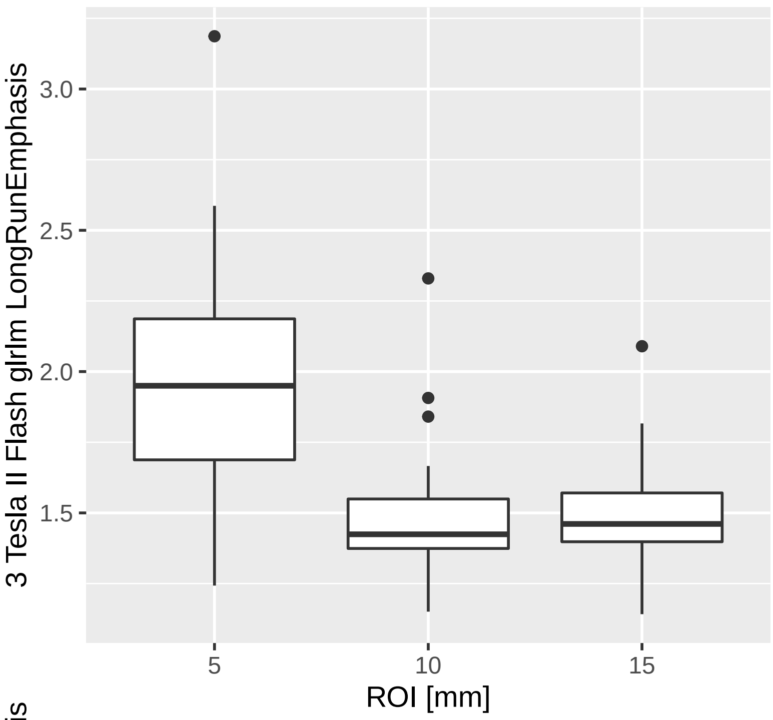
3 Tesla II Flash glrlm LongRunEmphasis
1.5
2.0
2.5
3.0
5
10
15
ROI [mm]

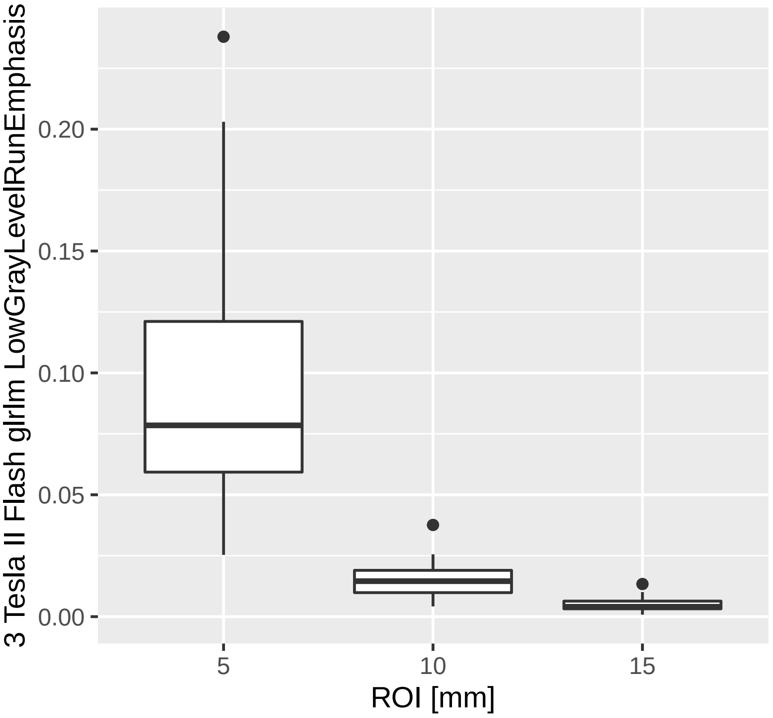
3 Tesla II Flash glrlm LowGrayLevelRunEmphasis
0.00
0.05
0.10
0.15
0.20
5
10
15
ROI [mm]

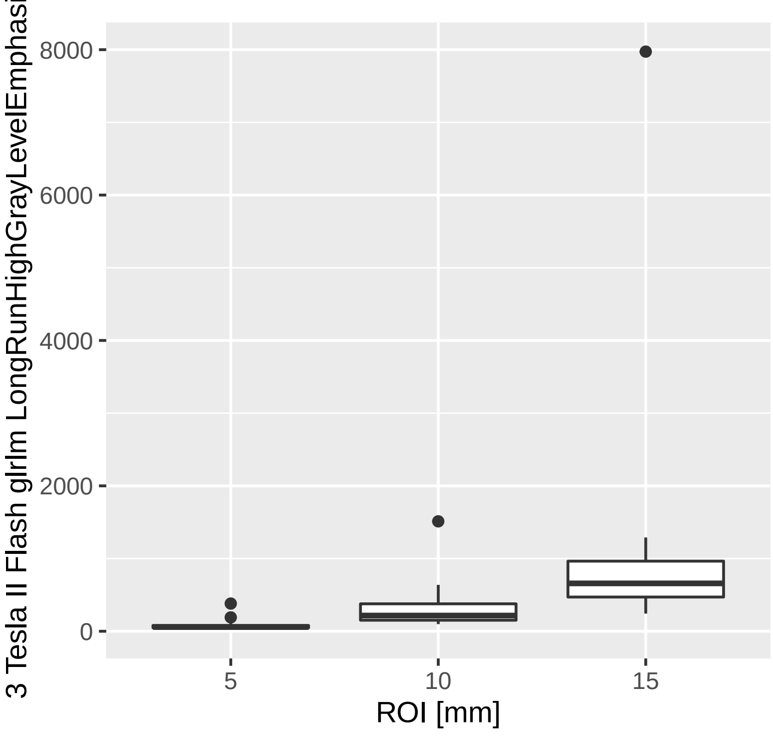
3 Tesla II Flash glrlm LongRunHighGrayLevelEmphasis
0
2000
4000
6000
8000
5
10
15
ROI [mm]

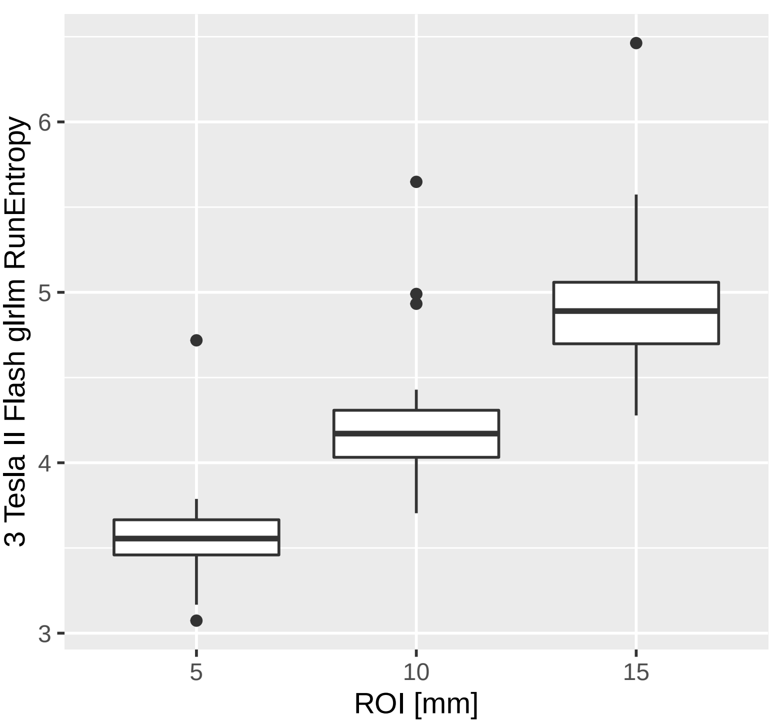
3 Tesla II Flash glrlm RunEntropy
3
4
5
6
5
10
15
ROI [mm]

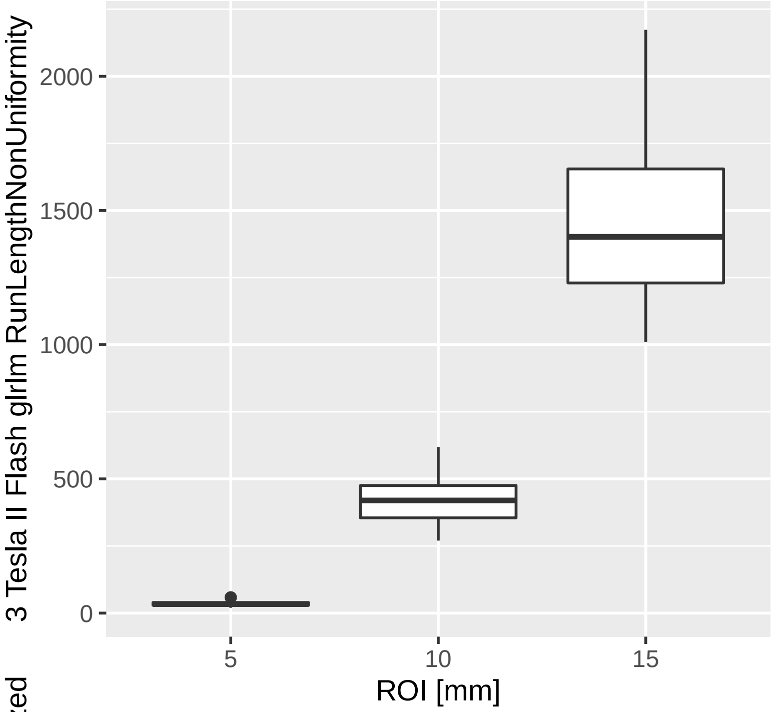
3 Tesla II Flash glrlm RunLengthNonUniformity
0
500
1000
1500
2000
5
10
15
ROI [mm]

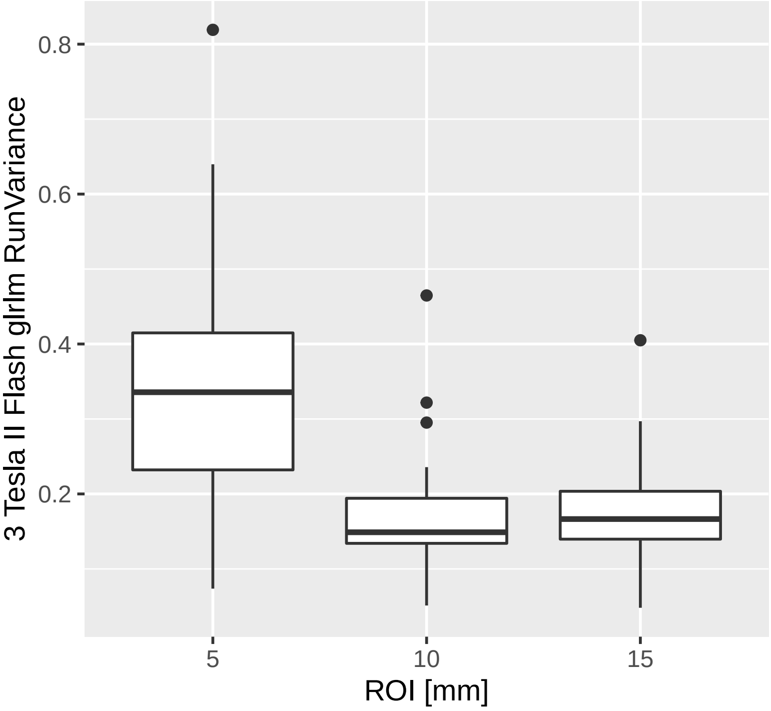
3 Tesla II Flash glrlm RunVariance
0.2
0.4
0.6
0.8
5
10
15
ROI [mm]

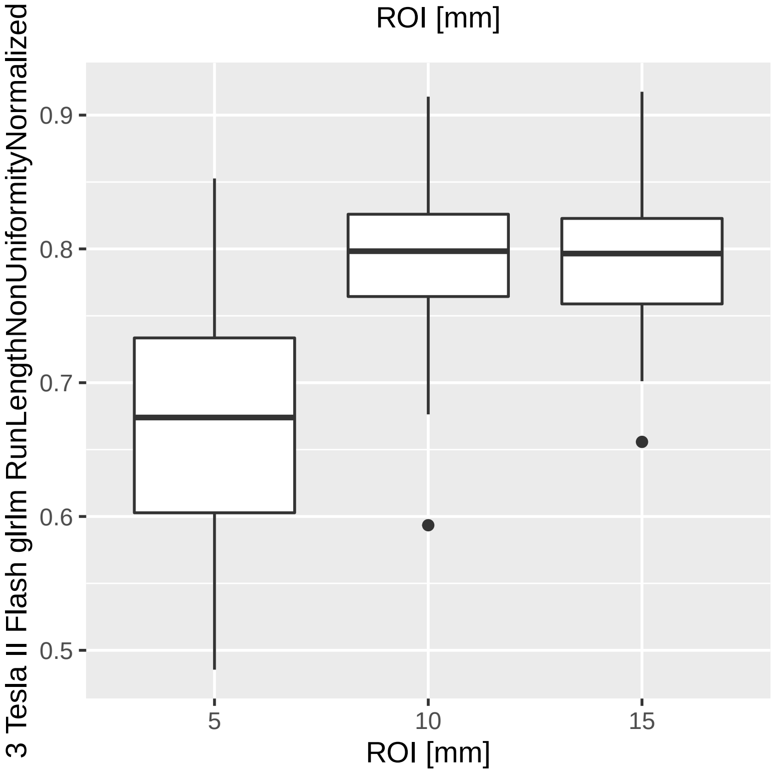
3 Tesla II Flash glrlm RunLengthNonUniformityNormalized
0.5
0.6
0.7
0.8
0.9
5
10
15
ROI [mm]

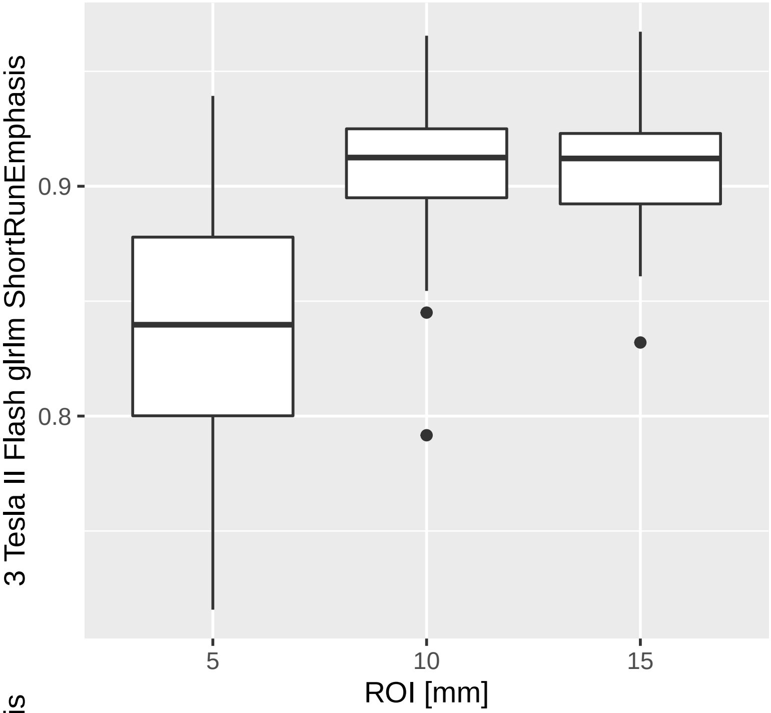
3 Tesla II Flash glrlm ShortRunEmphasis
0.8
0.9
5
10
15
ROI [mm]

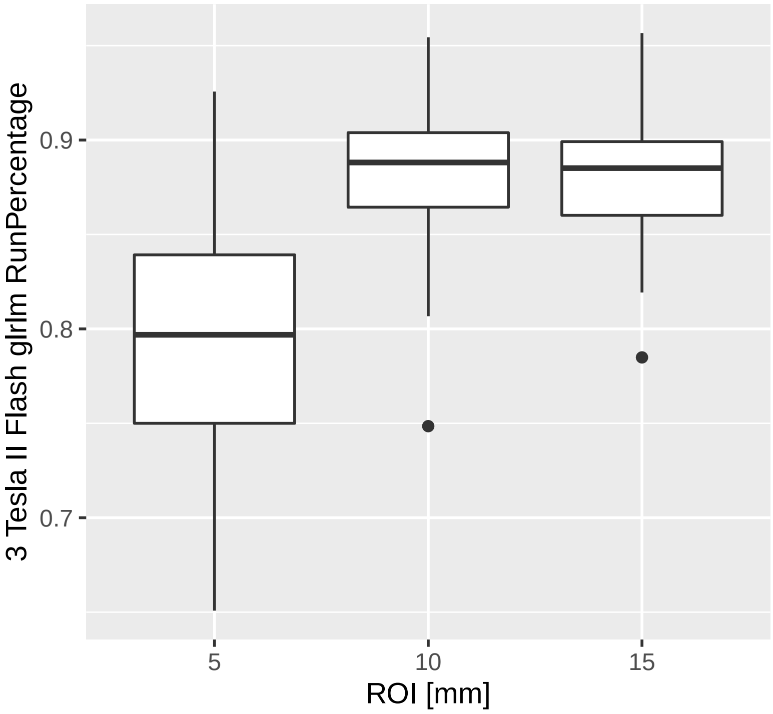
3 Tesla II Flash glrlm RunPercentage
0.7
0.8
0.9
5
10
15
ROI [mm]

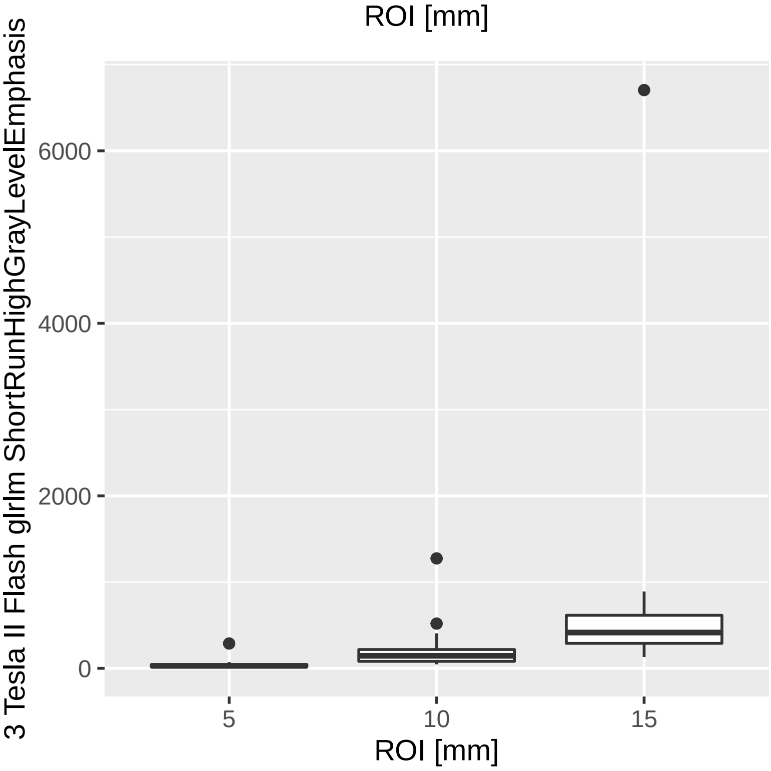
3 Tesla II Flash glrlm ShortRunHighGrayLevelEmphasis
0
2000
4000
6000
5
10
15
ROI [mm]

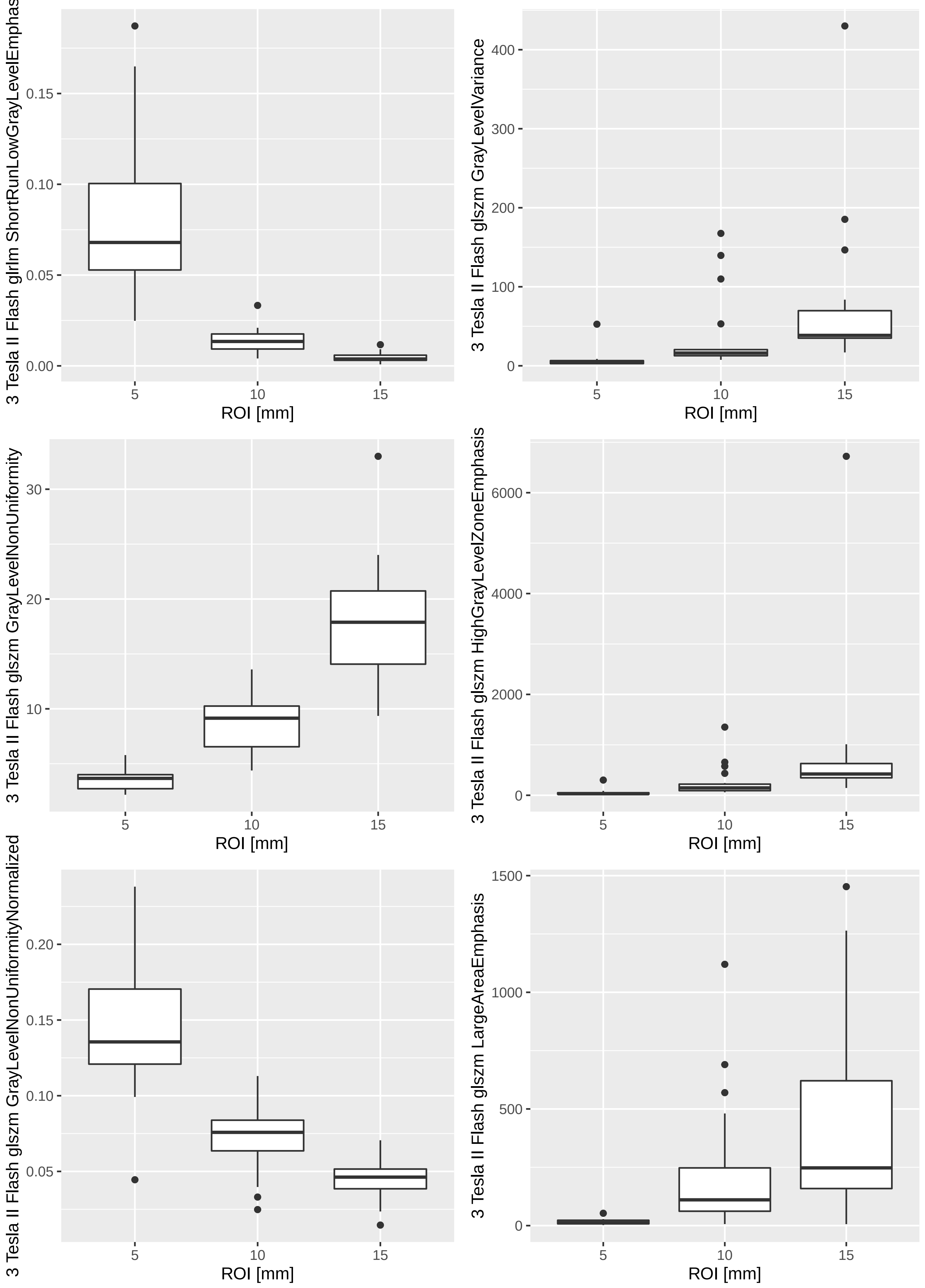

3 Tesla II Flash glrlm ShortRunLowGrayLevelEmphasis
0.00
0.05
0.10
0.15
5
10
15
ROI [mm]
3 Tesla II Flash glszm GrayLevelVariance
0
100
200
300
400
5
10
15
ROI [mm]
3 Tesla II Flash glszm GrayLevelNonUniformity
10
20
30
5
10
15
ROI [mm]
3 Tesla II Flash glszm HighGrayLevelZoneEmphasis
0
2000
4000
6000
5
10
15
ROI [mm]
3 Tesla II Flash glszm GrayLevelNonUniformityNormalized
0.05
0.10
0.15
0.20
5
10
15
ROI [mm]
3 Tesla II Flash glszm LargeAreaEmphasis
0
500
1000
1500
5
10
15
ROI [mm]

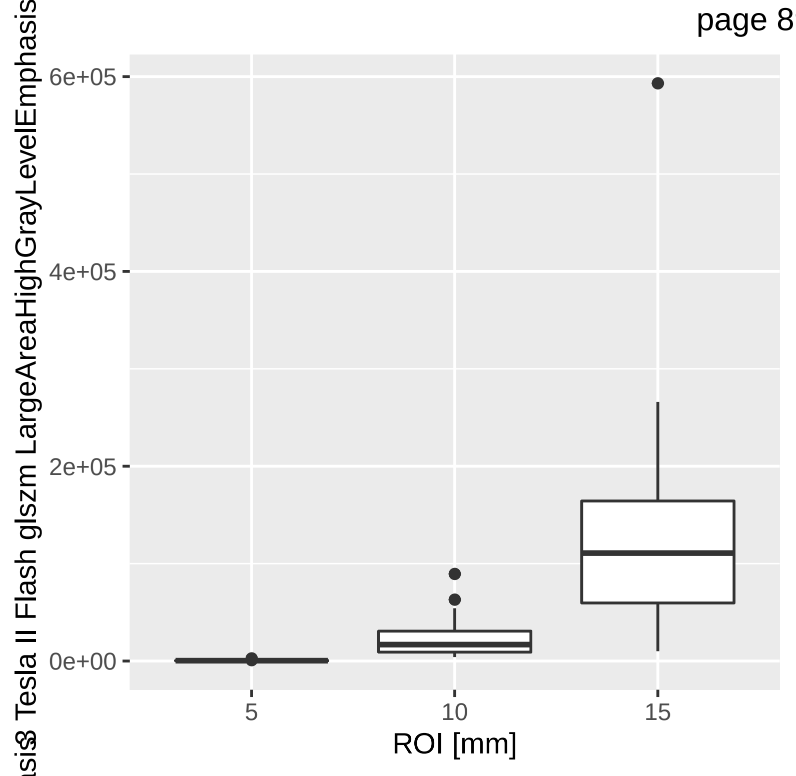
3 Tesla II Flash glszm LargeAreaHighGrayLevelEmphasis
6e+05
4e+05
2e+05
0e+00
5
10
15
ROI [mm]

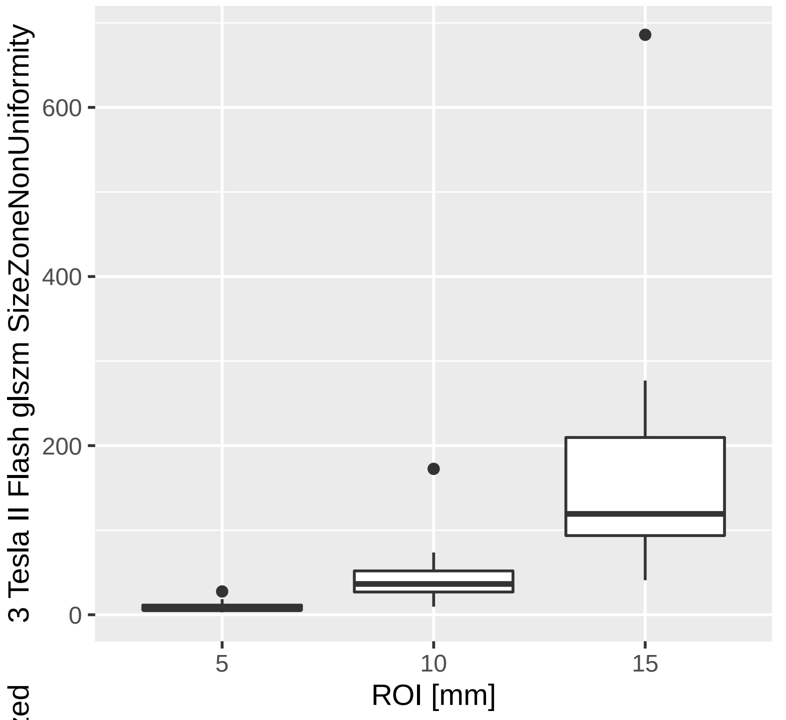
3 Tesla II Flash glszm SizeZoneNonUniformity
600
400
200
0
5
10
15
ROI [mm]

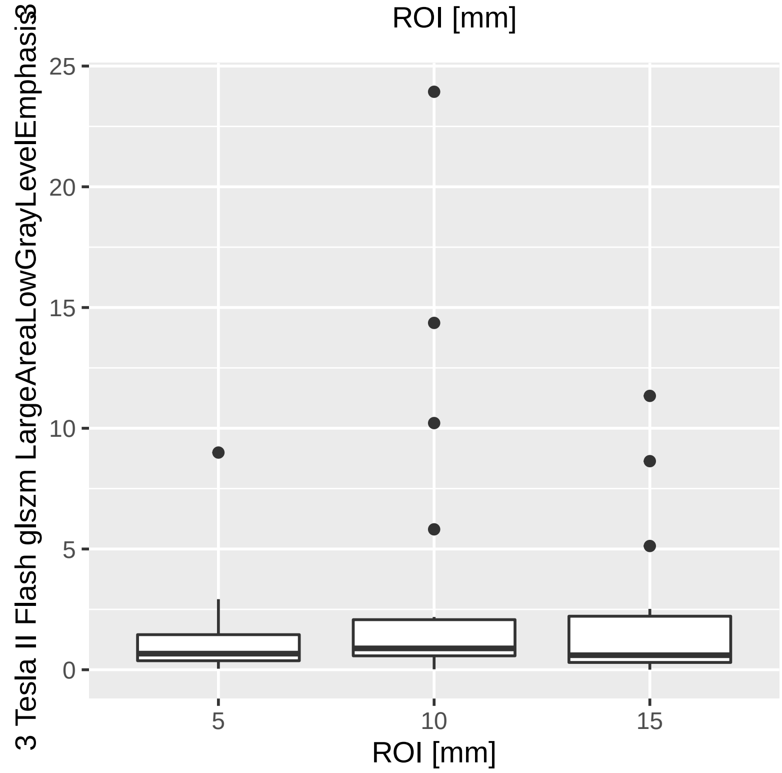
3 Tesla II Flash glszm LargeAreaLowGrayLevelEmphasis
25
20
15
10
5
0
5
10
15
ROI [mm]

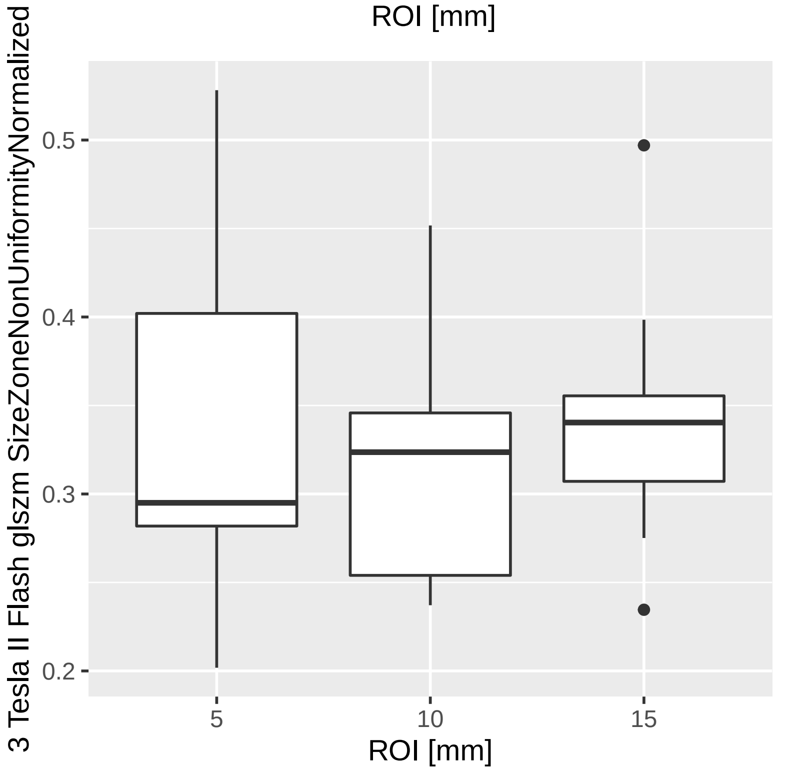
3 Tesla II Flash glszm SizeZoneNonUniformityNormalized
0.5
0.4
0.3
0.2
5
10
15
ROI [mm]

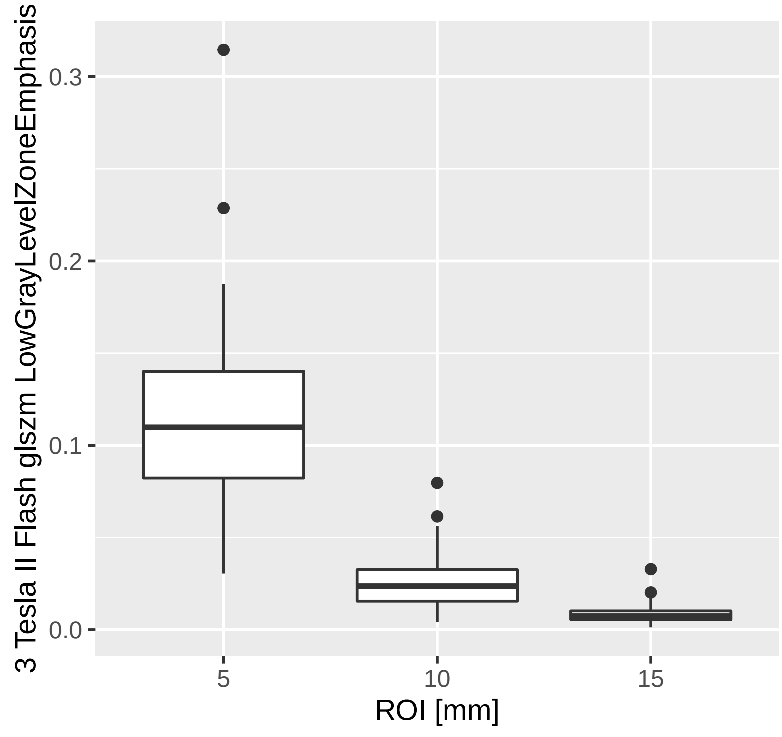
3 Tesla II Flash glszm LowGrayLevelZoneEmphasis
0.3
0.2
0.1
0.0
5
10
15
ROI [mm]

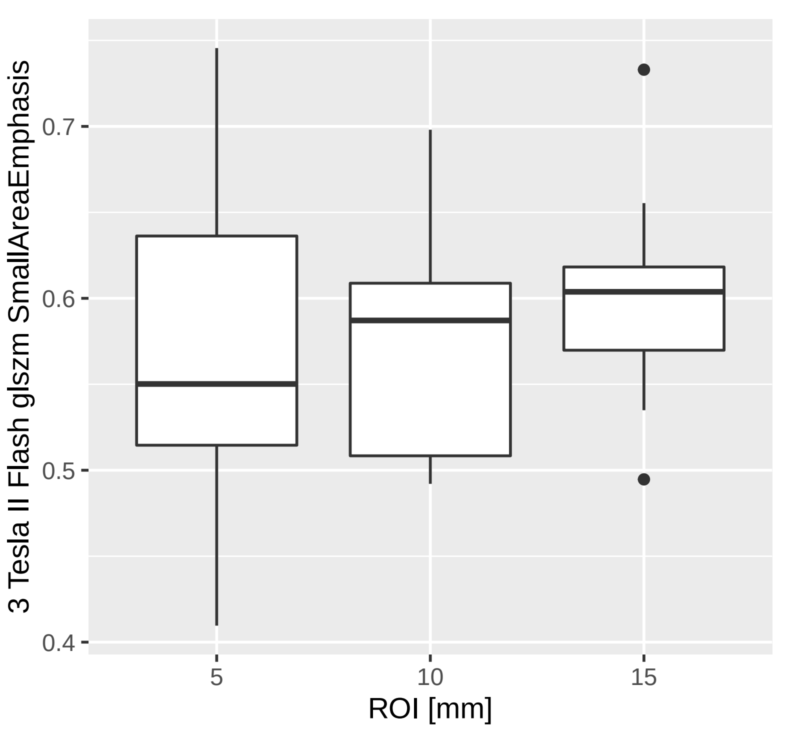
3 Tesla II Flash glszm SmallAreaEmphasis
0.7
0.6
0.5
0.4
5
10
15
ROI [mm]

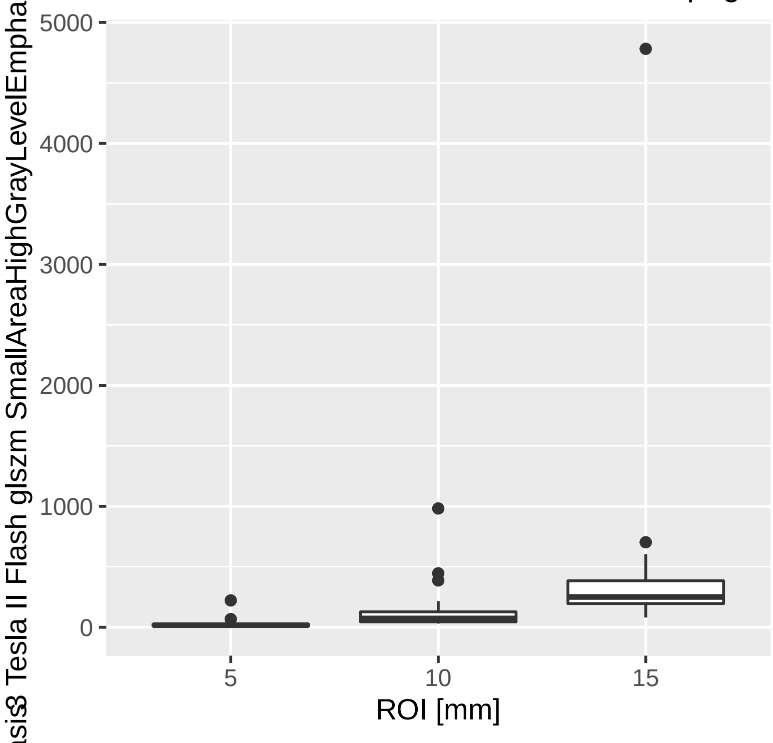
3 Tesla II Flash glszm SmallAreaHighGrayLevelEmphasis
5000
4000
3000
2000
1000
0
5
10
15
ROI [mm]

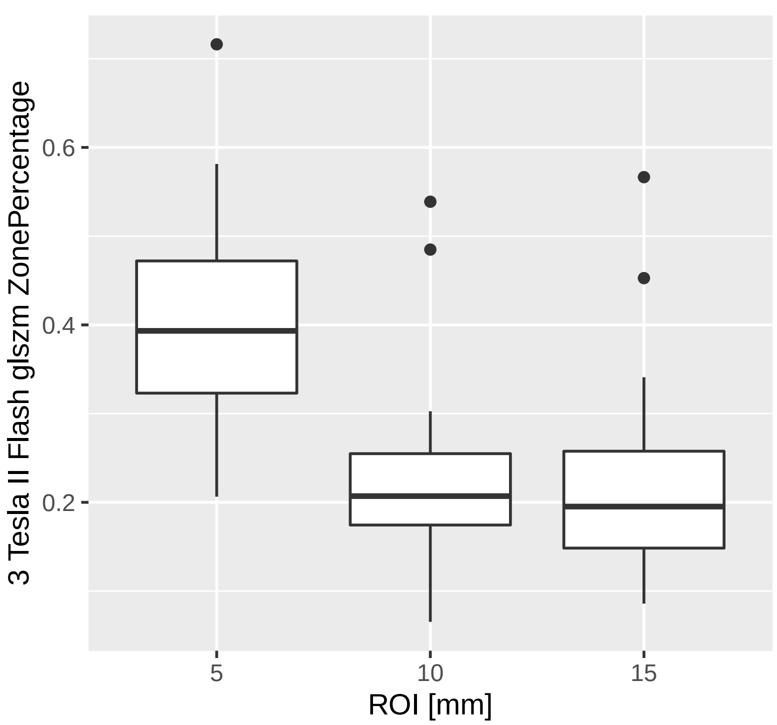
3 Tesla II Flash glszm ZonePercentage
0.6
0.4
0.2
5
10
15
ROI [mm]

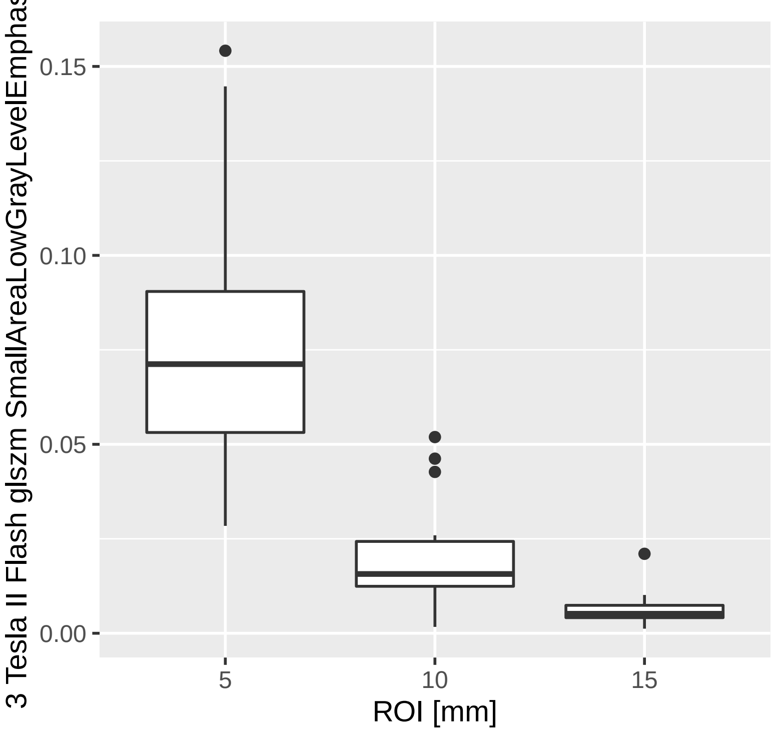
3 Tesla II Flash glszm SmallAreaLowGrayLevelEmphasis
0.15
0.10
0.05
0.00
5
10
15
ROI [mm]

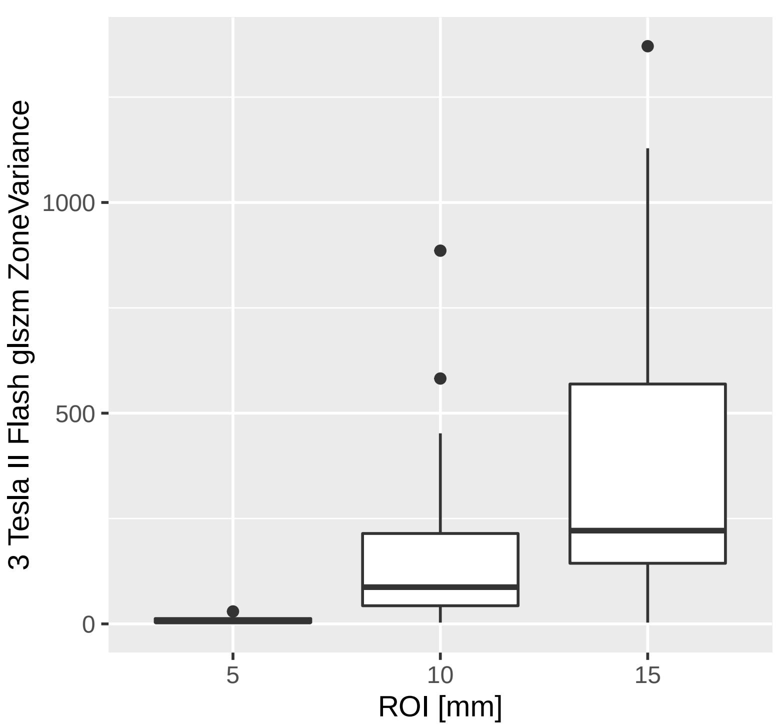
3 Tesla II Flash glszm ZoneVariance
1000
500
0
5
10
15
ROI [mm]

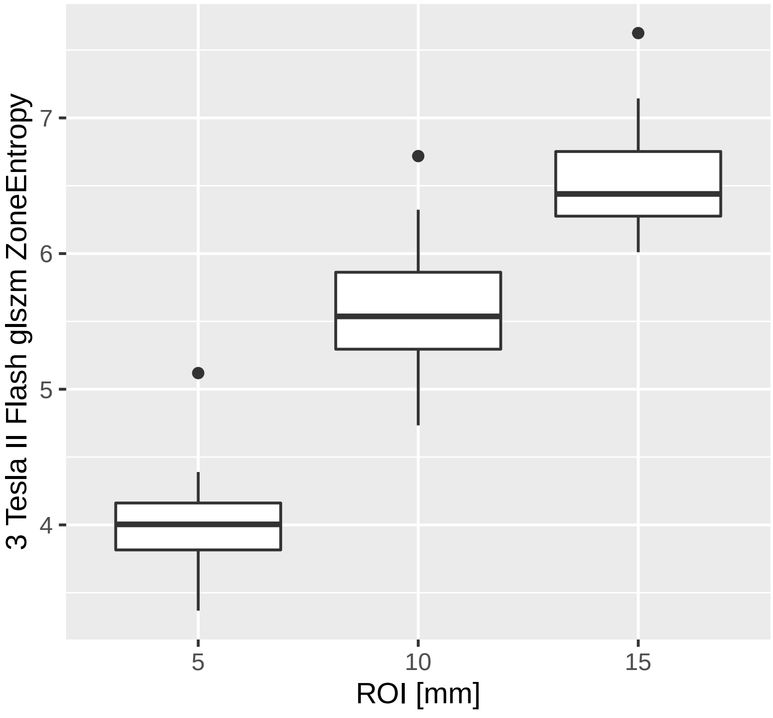
3 Tesla II Flash glszm ZoneEntropy
7
6
5
4
5
10
15
ROI [mm]

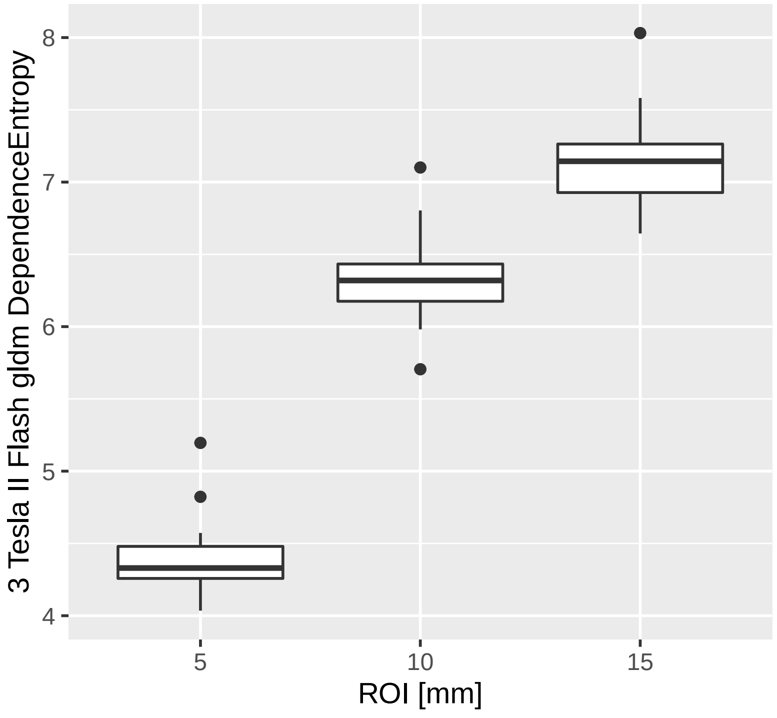
3 Tesla II Flash gldm DependenceEntropy
8
7
6
5
4
5
10
15
ROI [mm]

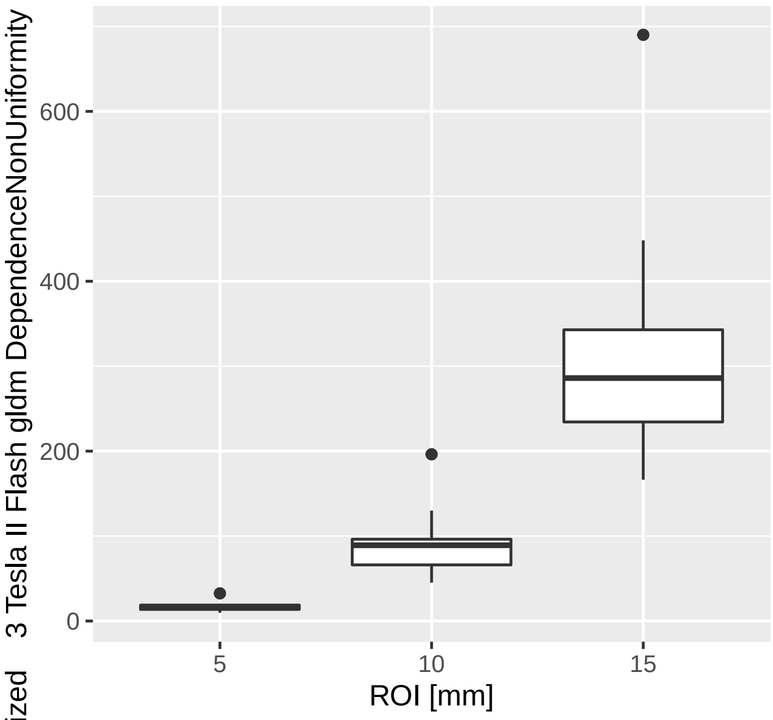

3 Tesla II Flash gldm DependenceNonUniformity
0
200
400
600
5
10
15
ROI [mm]

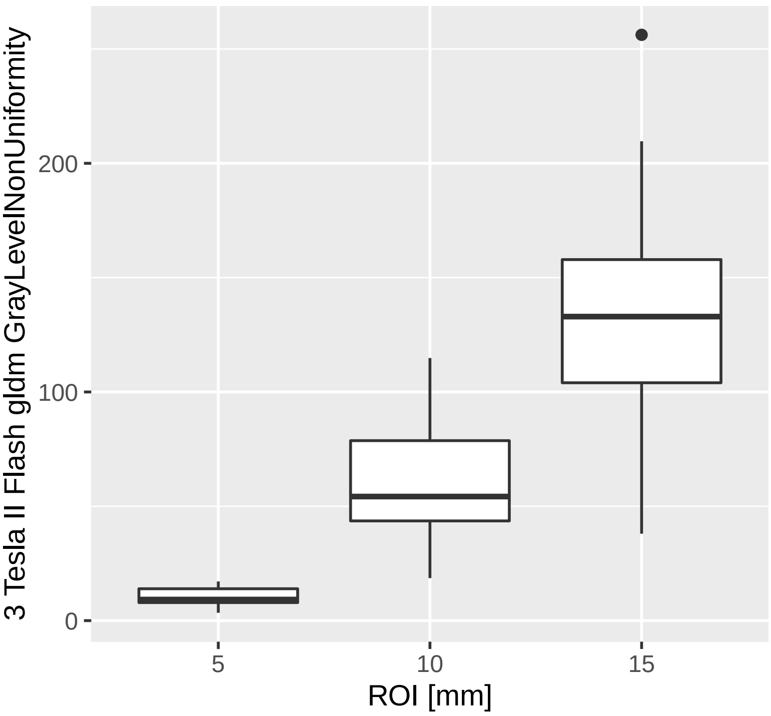

3 Tesla II Flash gldm GrayLevelNonUniformity
0
100
200
5
10
15
ROI [mm]

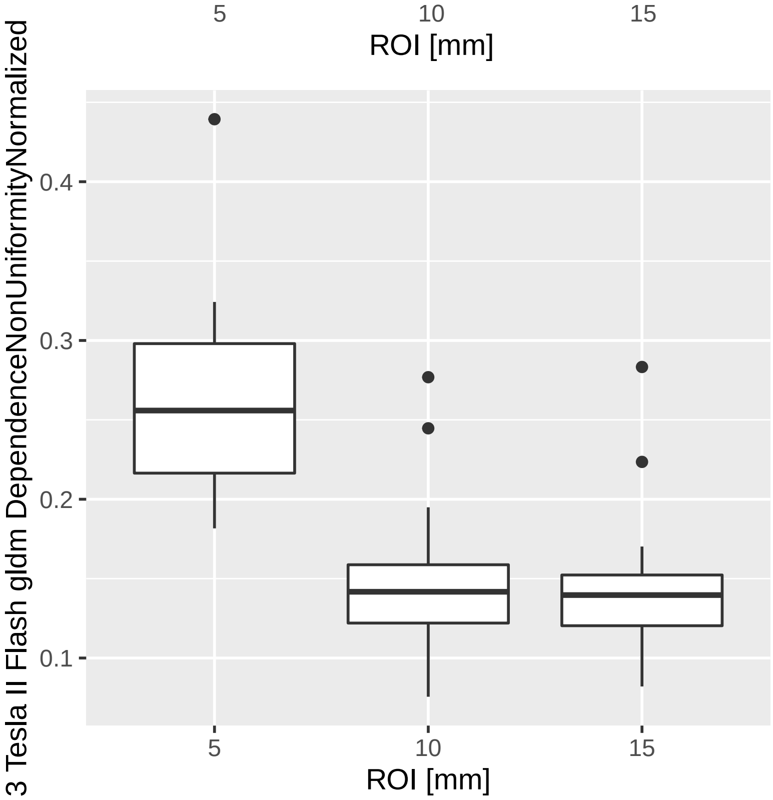

3 Tesla II Flash gldm DependenceNonUniformityNormalized
0.1
0.2
0.3
0.4
5
10
15
ROI [mm]

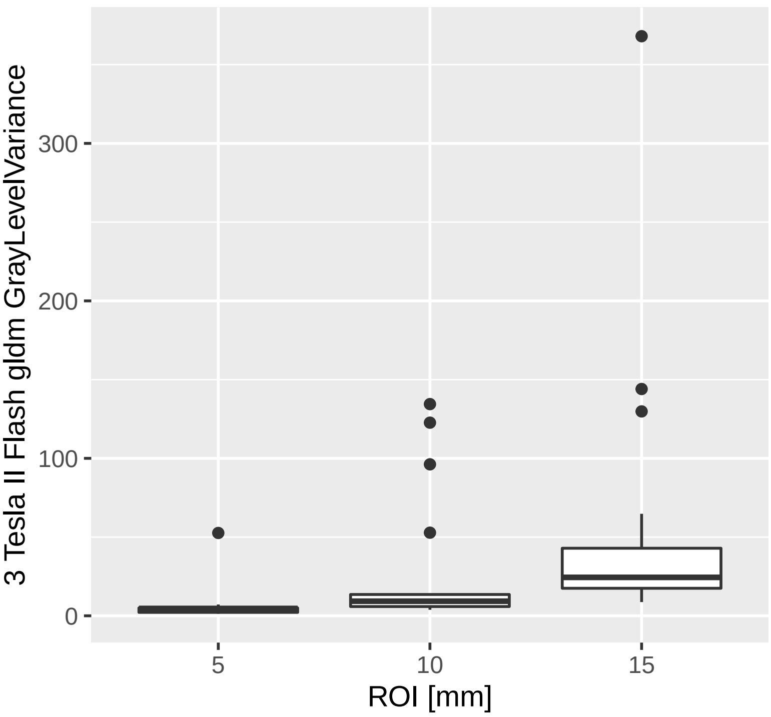

3 Tesla II Flash gldm GrayLevelVariance
0
100
200
300
5
10
15
ROI [mm]

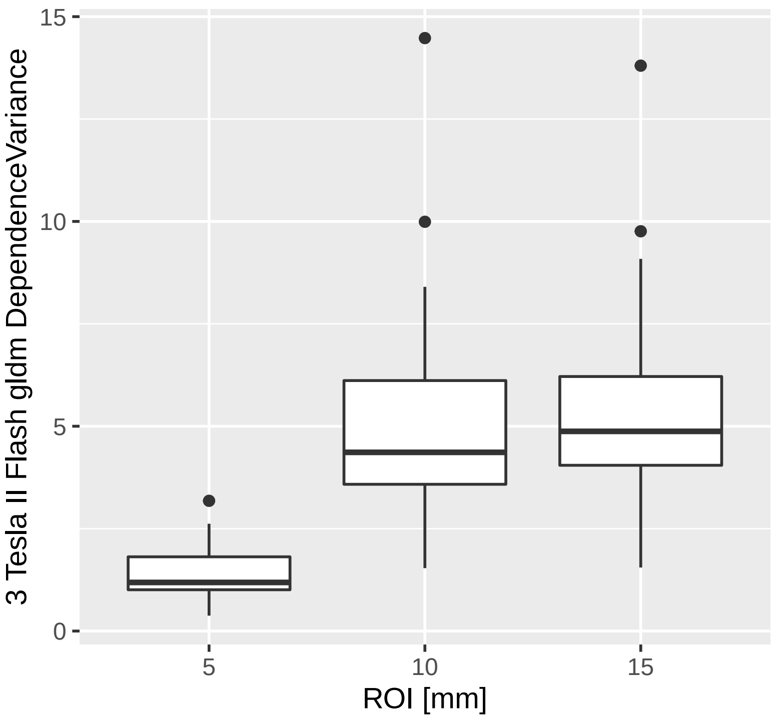

3 Tesla II Flash gldm DependenceVariance
0
5
10
15
5
10
15
ROI [mm]

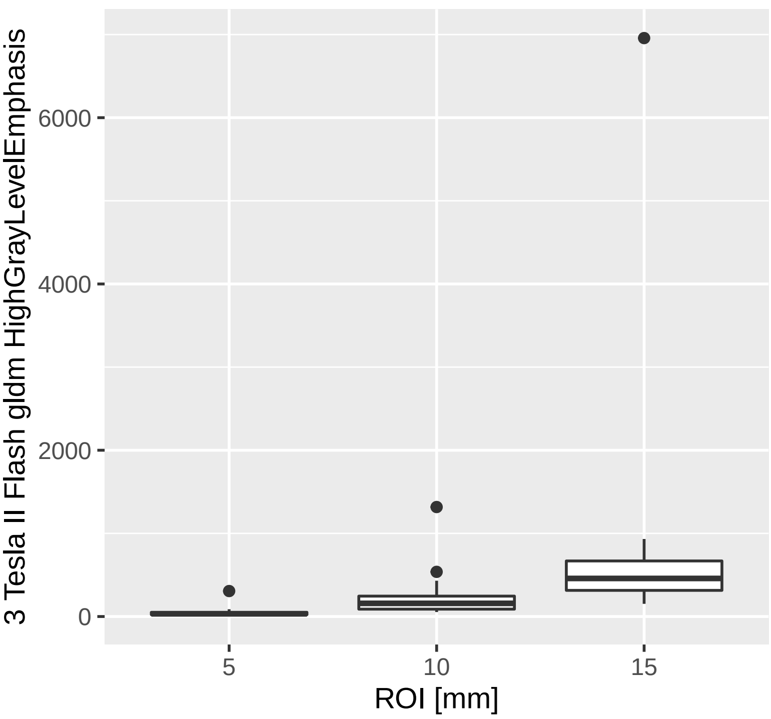

3 Tesla II Flash gldm HighGrayLevelEmphasis
0
2000
4000
6000
5
10
15
ROI [mm]

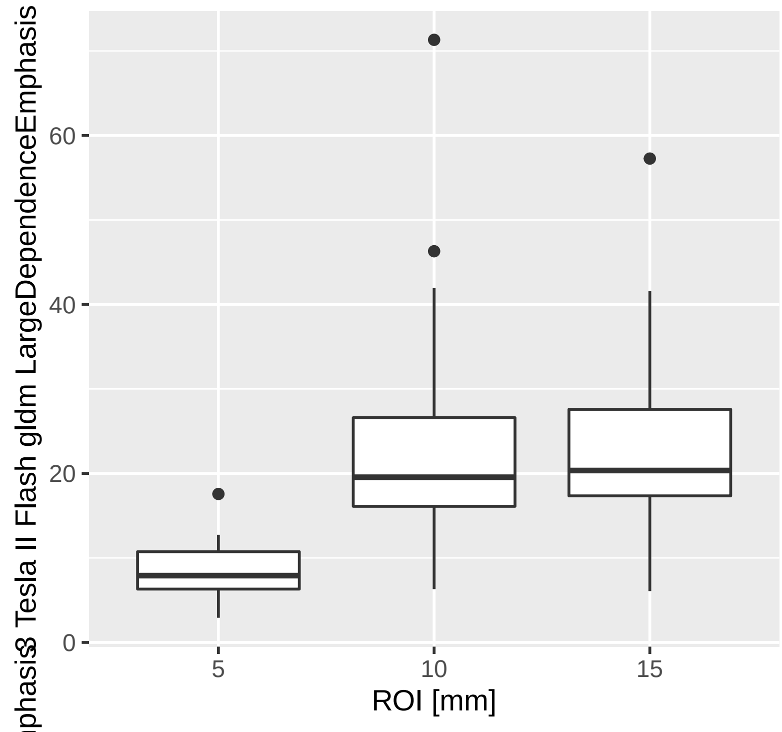
3 Tesla II Flash gldm LargeDependenceEmphasis
0
20
40
60
5
10
15
ROI [mm]

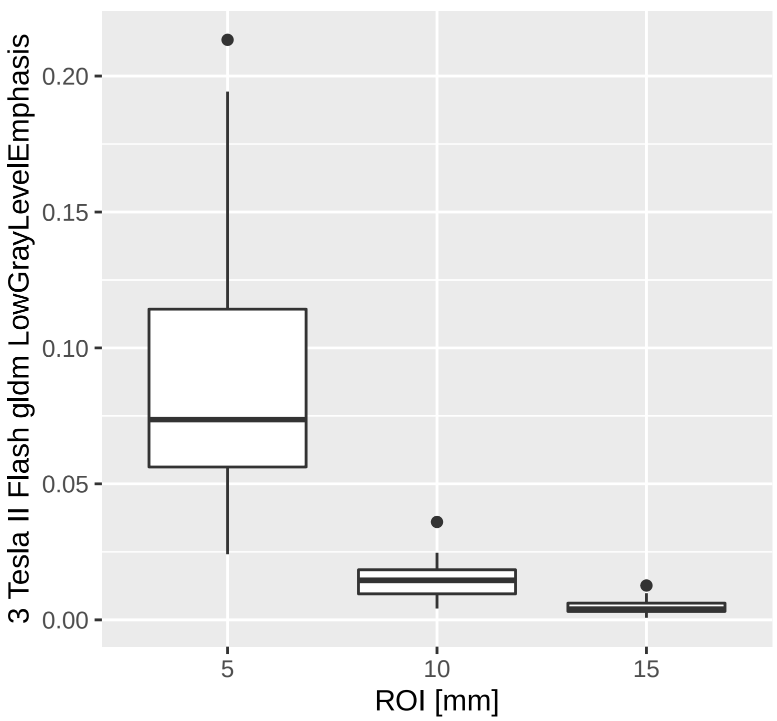
3 Tesla II Flash gldm LowGrayLevelEmphasis
0.00
0.05
0.10
0.15
0.20
5
10
15
ROI [mm]

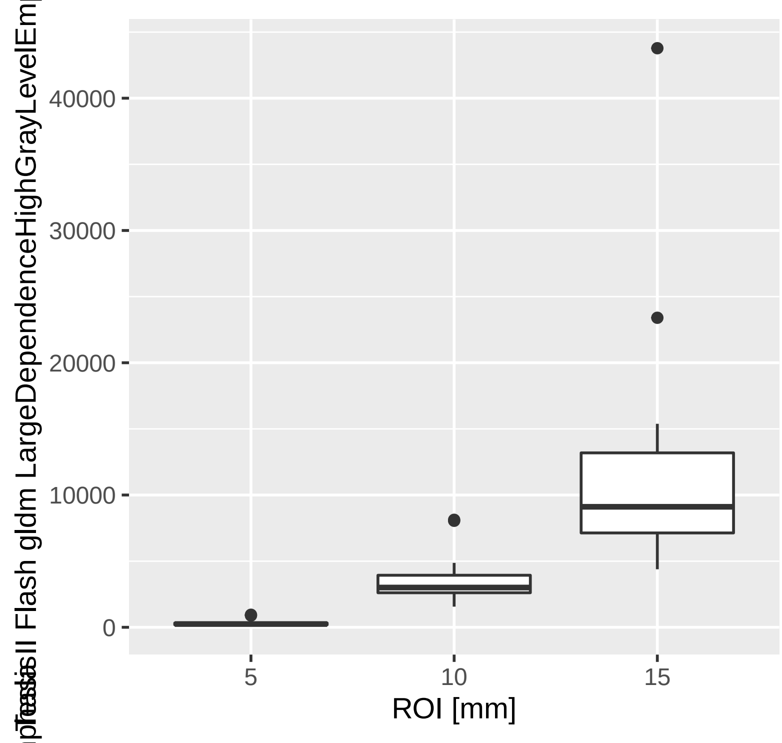
Tesla II Flash gldm LargeDependenceHighGrayLevelEmphasis
0
10000
20000
30000
40000
5
10
15
ROI [mm]

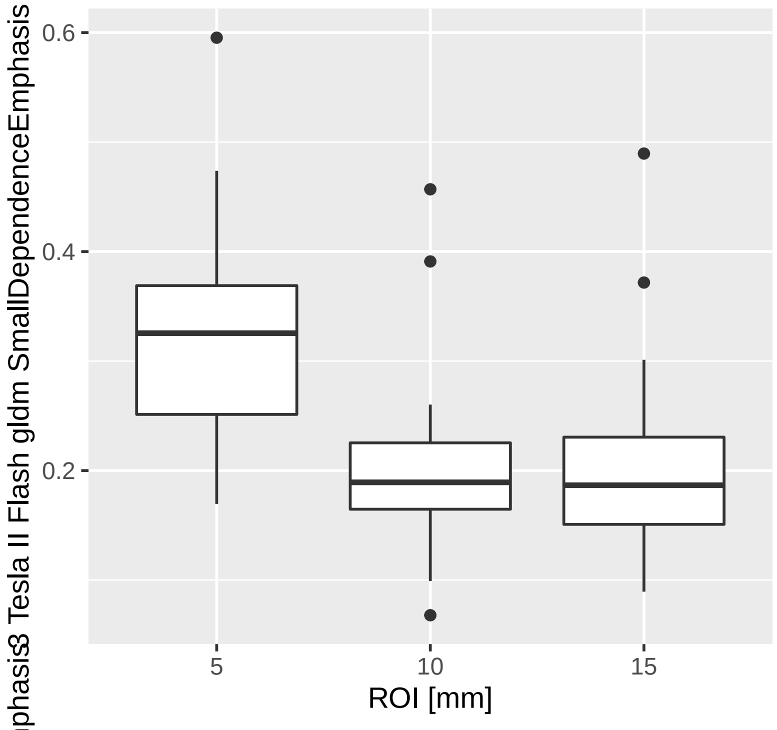
3 Tesla II Flash gldm SmallDependenceEmphasis
0.2
0.4
0.6
5
10
15
ROI [mm]

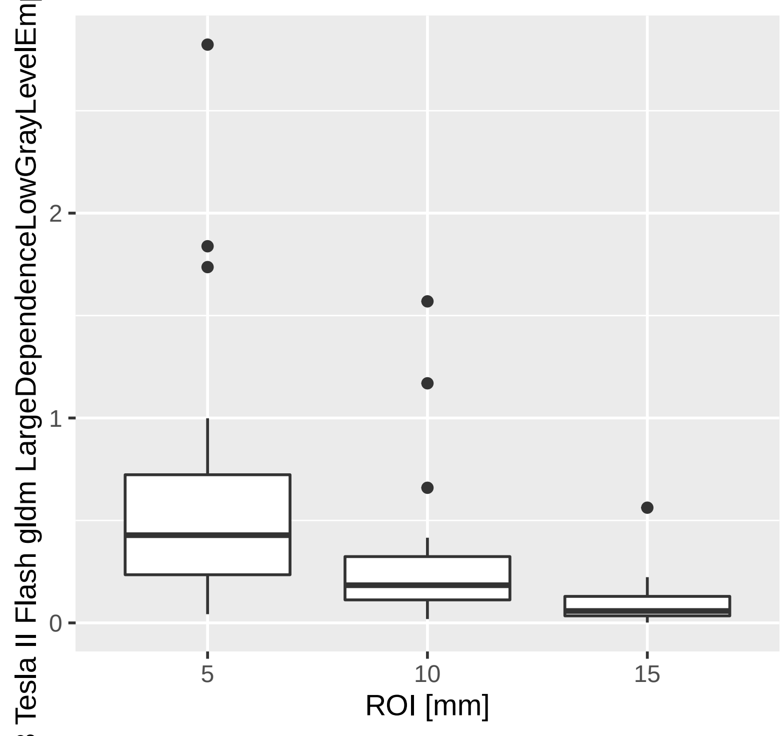
Tesla II Flash gldm LargeDependenceLowGrayLevelEmphasis
0
1
2
5
10
15
ROI [mm]

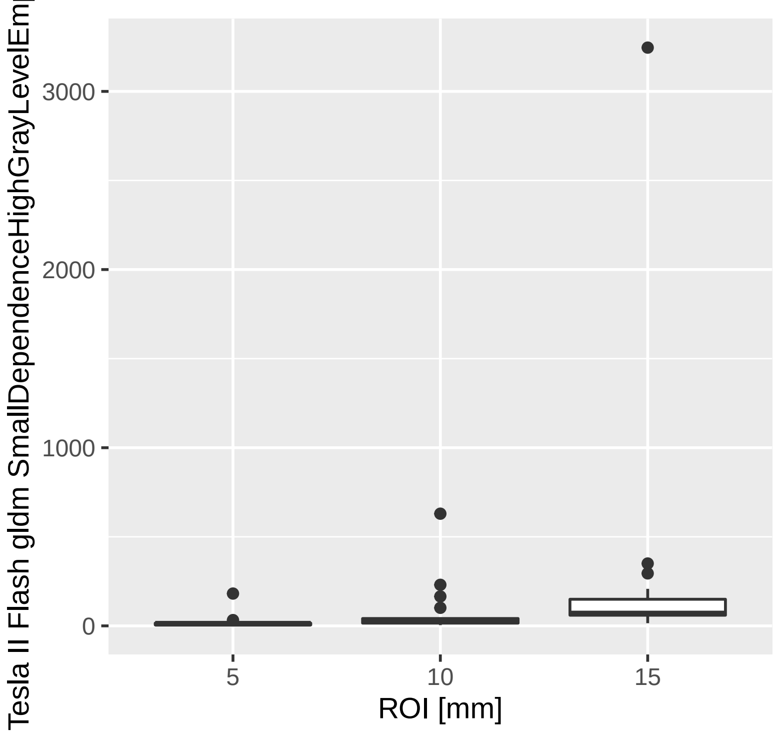
Tesla II Flash gldm SmallDependenceHighGrayLevelEmphasis
0
1000
2000
3000
5
10
15
ROI [mm]

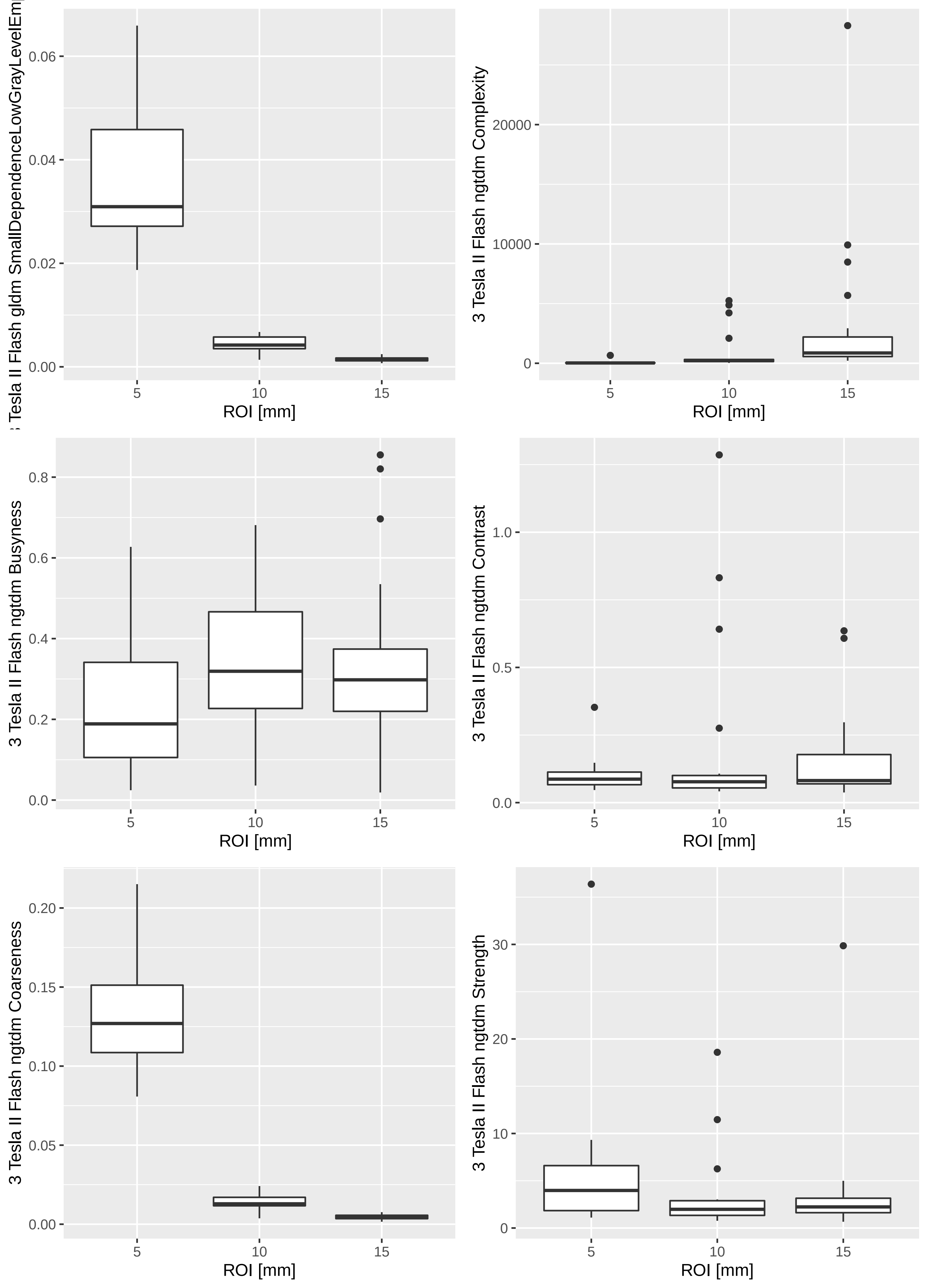
Tesla II Flash gldm SmallDependenceLowGrayLevelEmpha
0.00
0.02
0.04
0.06
5
10
15
ROI [mm]
3 Tesla II Flash ngtdm Complexity
0
10000
20000
5
10
15
ROI [mm]
3 Tesla II Flash ngtdm Busyness
0.0
0.2
0.4
0.6
0.8
5
10
15
ROI [mm]
3 Tesla II Flash ngtdm Contrast
0.0
0.5
1.0
5
10
15
ROI [mm]
3 Tesla II Flash ngtdm Coarseness
0.00
0.05
0.10
0.15
0.20
5
10
15
ROI [mm]
3 Tesla II Flash ngtdm Strength
0
10
20
30
5
10
15
ROI [mm]
