# Supplementary figures and images for: Stability of Liver Radiomics across Different 3D ROI Sizes—An MRI In Vivo Study
Source: Tomography. 2021 Dec 3;7(4):866–76. doi: 10.3390/tomography7040073 (PMC8706942; doi:10.3390/tomography7040073)

# 3 Tesla I – Flash – 10 / 20 / 30 mm

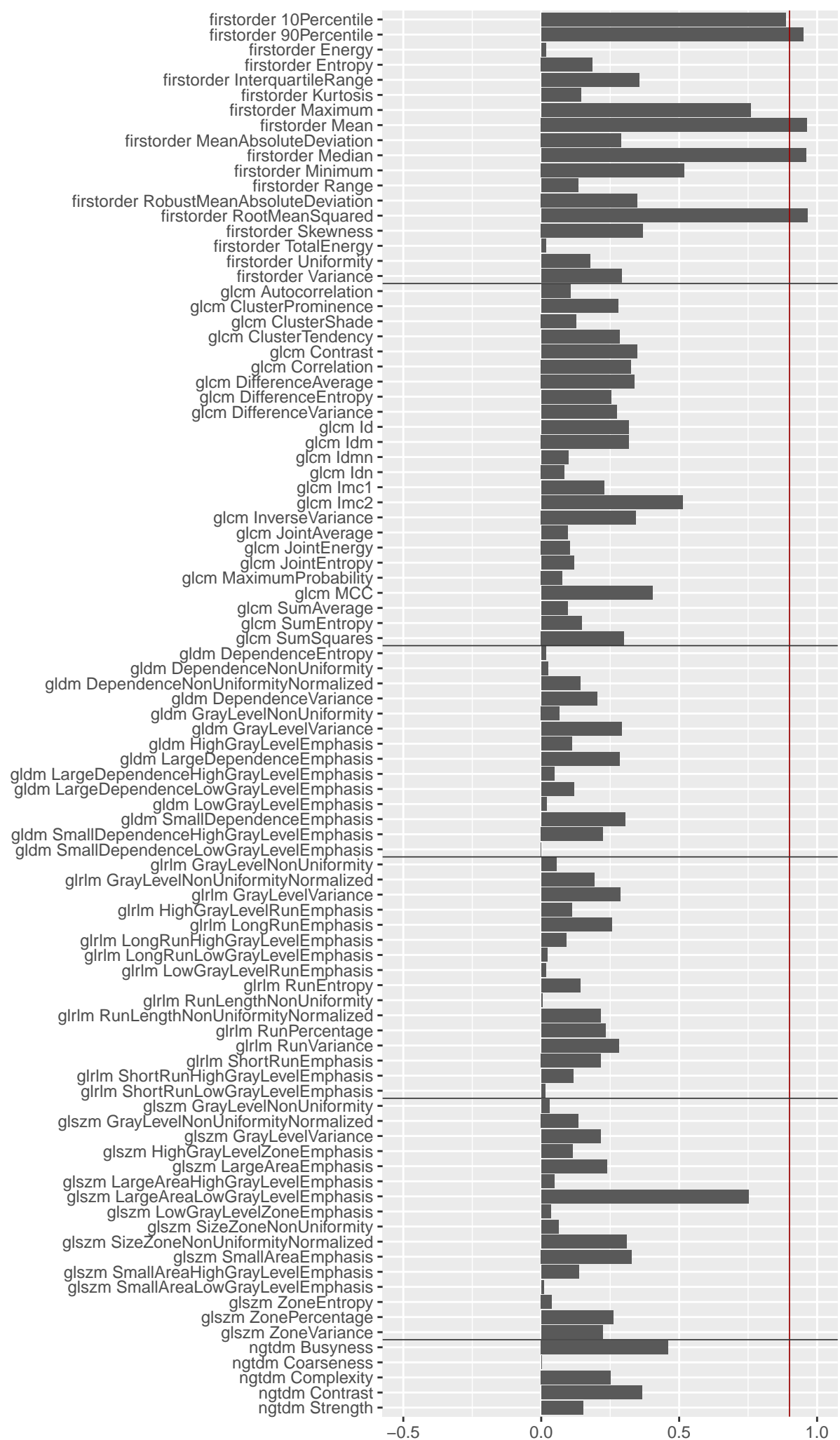

Supplement: Supplementary file 1 [file tomography-07-00073-s001.zip › SF6a_OCCCs_3_Tesla_I_T1_GRE_ROIs_10_20_30.pdf]

# 3 Tesla I – Flash – 10 / 20 mm

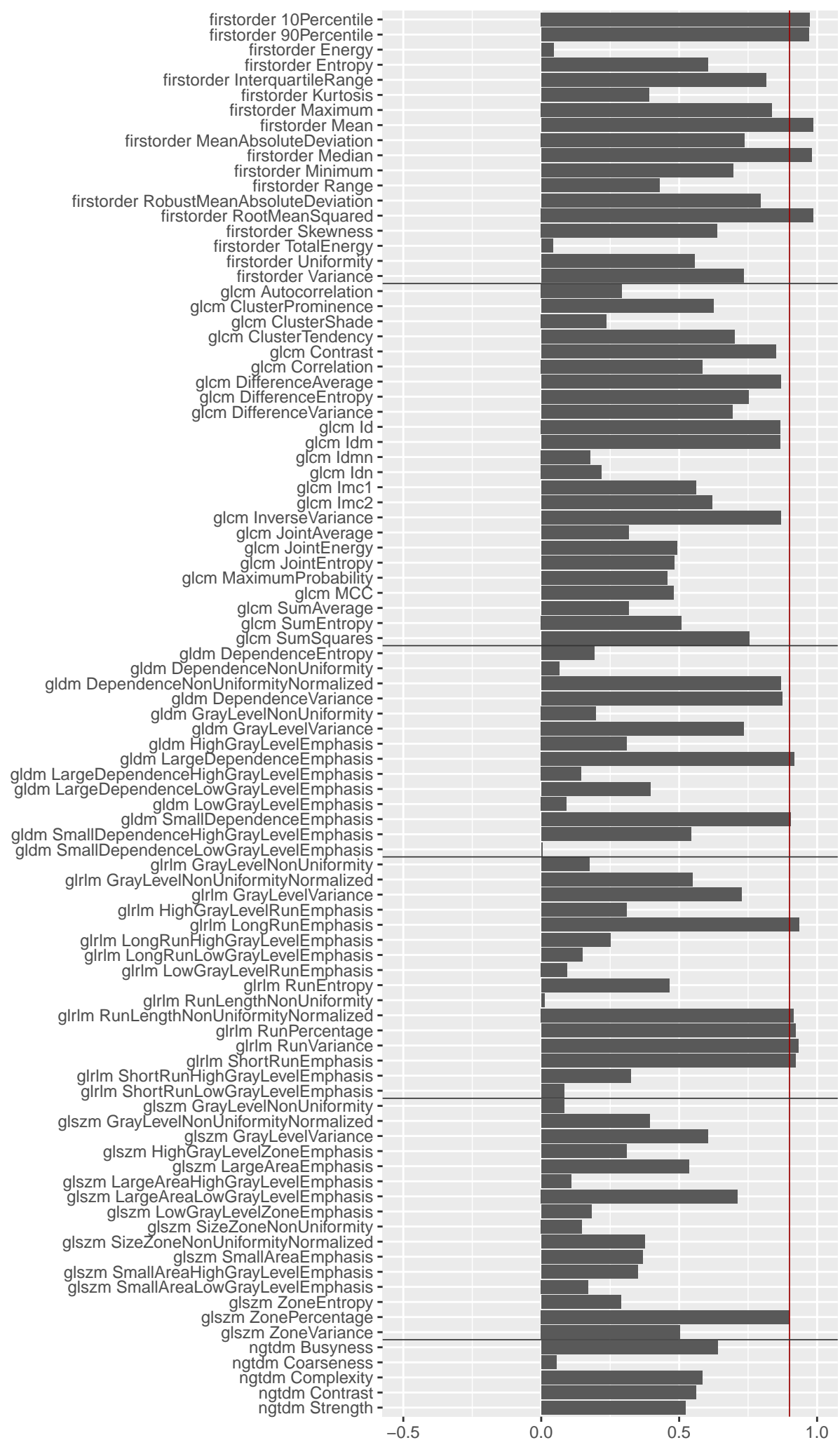

Supplement: Supplementary file 1 [file tomography-07-00073-s001.zip › SF6b_OCCCs_3_Tesla_I_T1_GRE_ROIs_20_30.pdf]

# 3 Tesla I – Haste – 10 / 20 / 30 mm

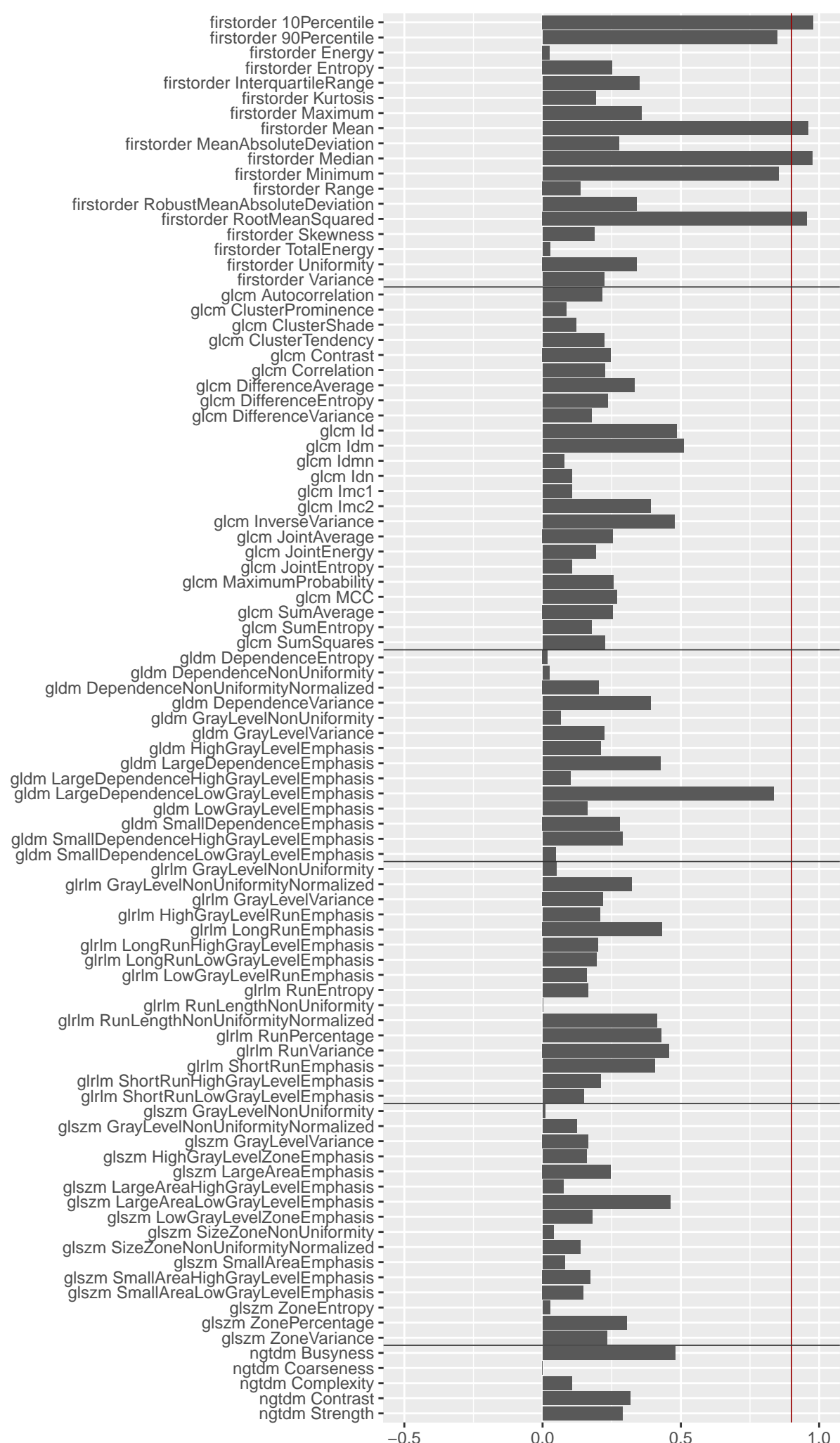

Supplement: Supplementary file 1 [file tomography-07-00073-s001.zip › SF6c_OCCCs_3_Tesla_I_T2_TSE_ROIs_10_20_30.pdf]

# 3 Tesla I – Haste – 10 / 20 mm

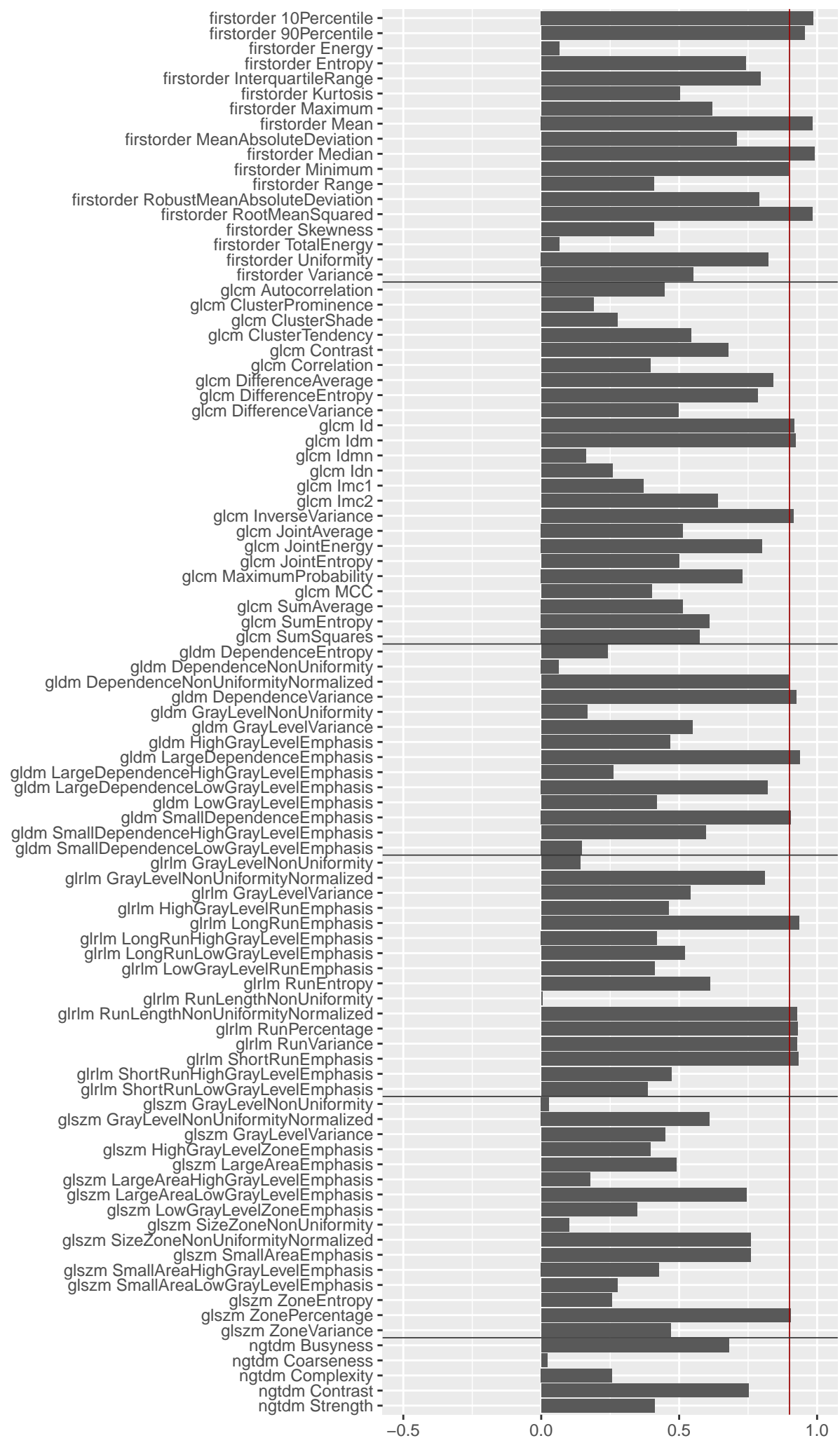

Supplement: Supplementary file 1 [file tomography-07-00073-s001.zip › SF6d_OCCCs_3_Tesla_I_T2_TSE_ROIs_20_30.pdf]

# 3 Tesla II – Flash – 10 / 20 / 30 mm

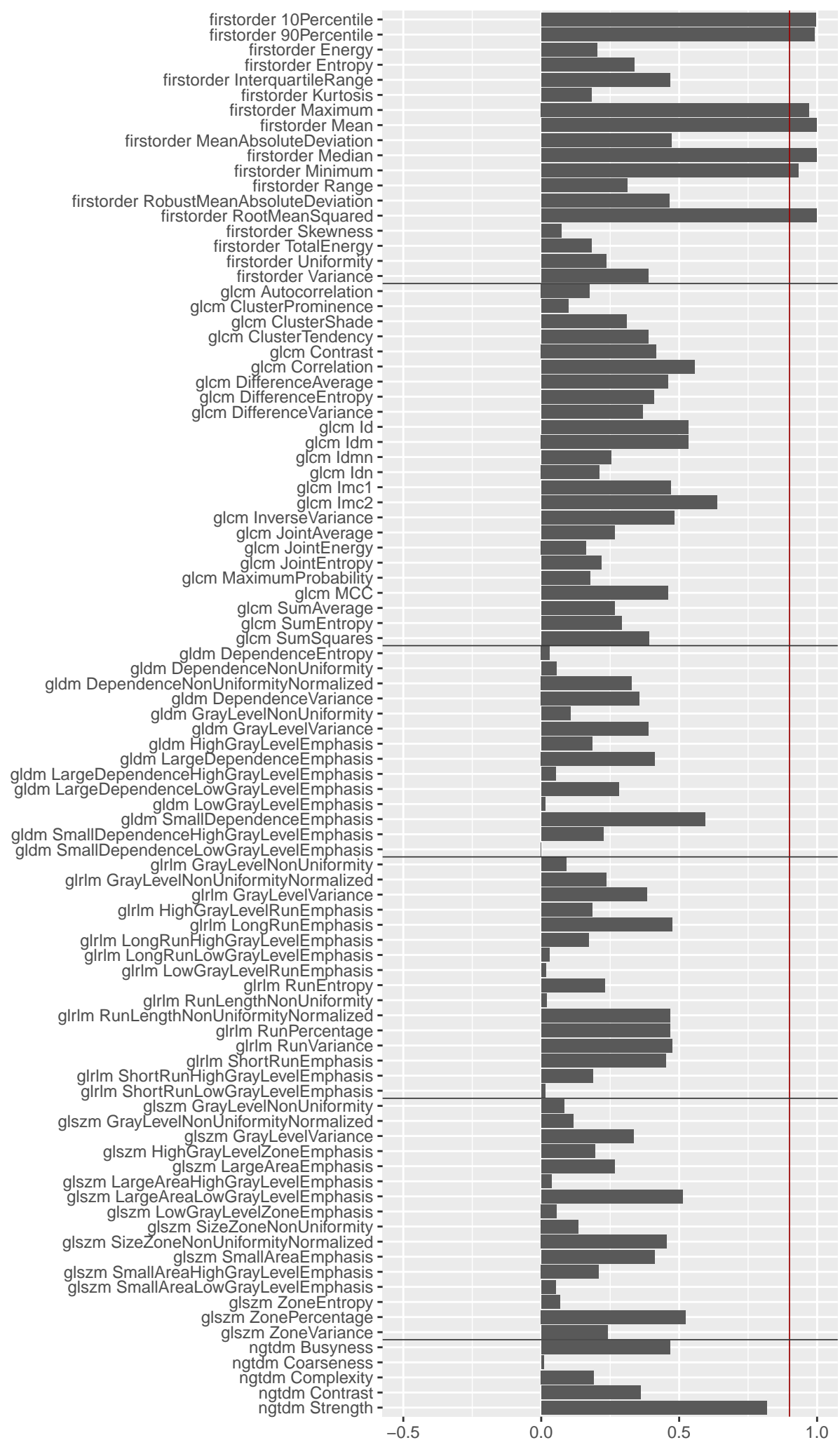

Supplement: Supplementary file 1 [file tomography-07-00073-s001.zip › SF7a_OCCCs_3_Tesla II_T1_GRE_ROIs_10_20_30.pdf]

### 3 Tesla II – Flash – 10 / 20 mm

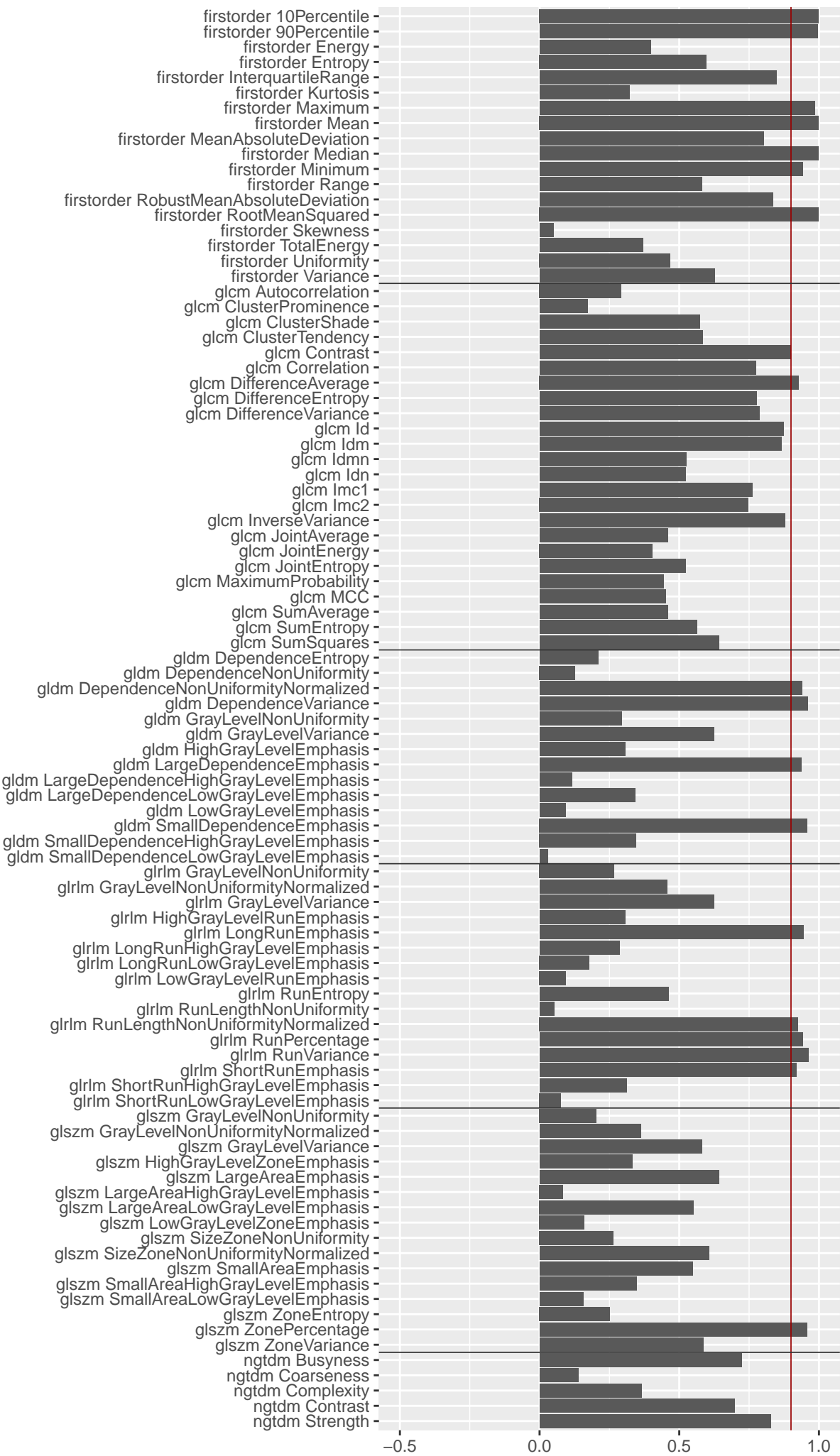

Supplement: Supplementary file 1 [file tomography-07-00073-s001.zip › SF7b_OCCCs_3_Tesla_II_T1_GRE_ROIs_20_30.pdf]

# 3 Tesla II – Haste – 10 / 20 / 30 mm

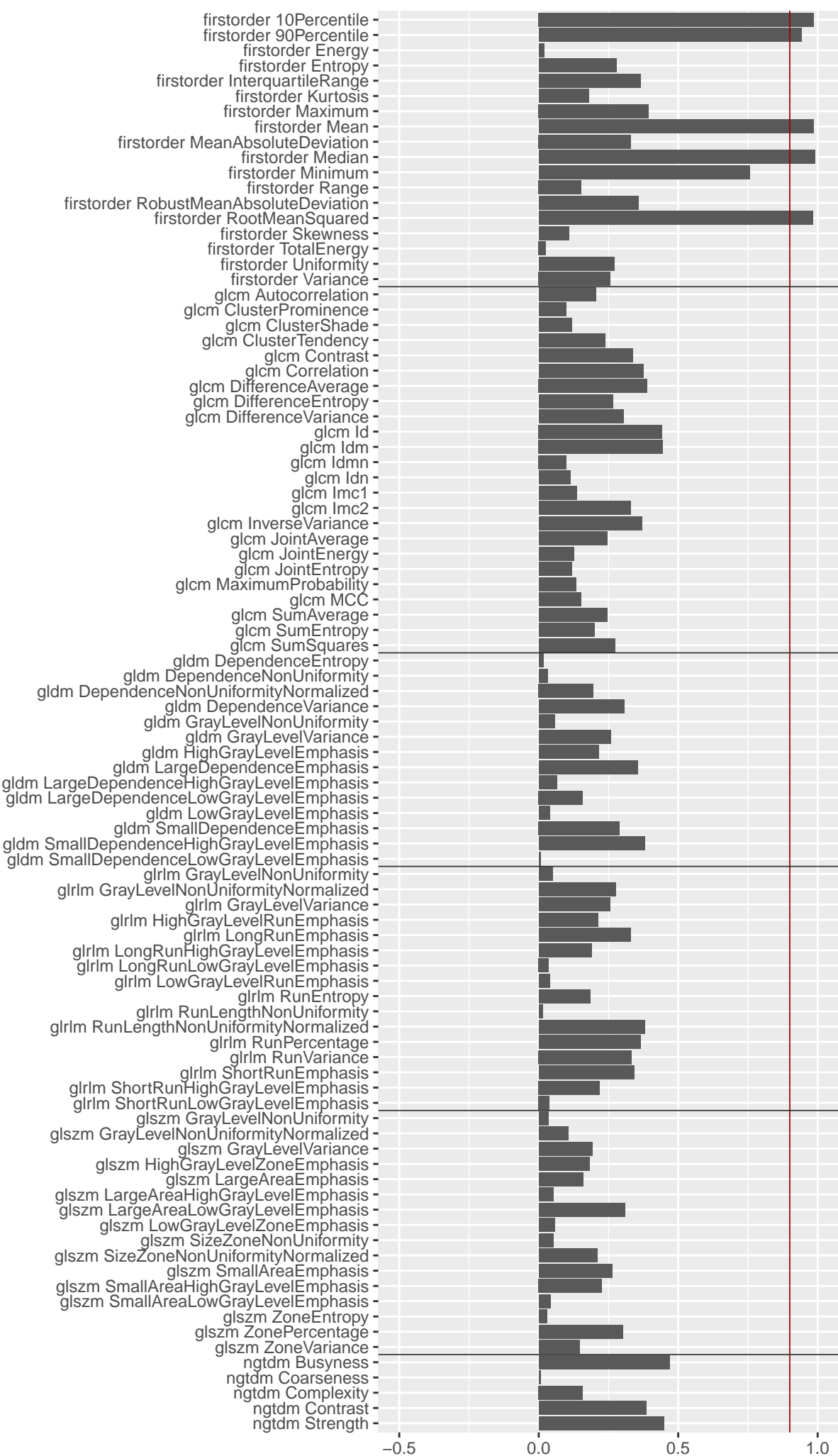

Supplement: Supplementary file 1 [file tomography-07-00073-s001.zip › SF7c_OCCCs_3_Tesla_II_T2_TSE_ROIs_10_20_30.pdf]

# 3 Tesla II – Haste – 10 / 20 mm

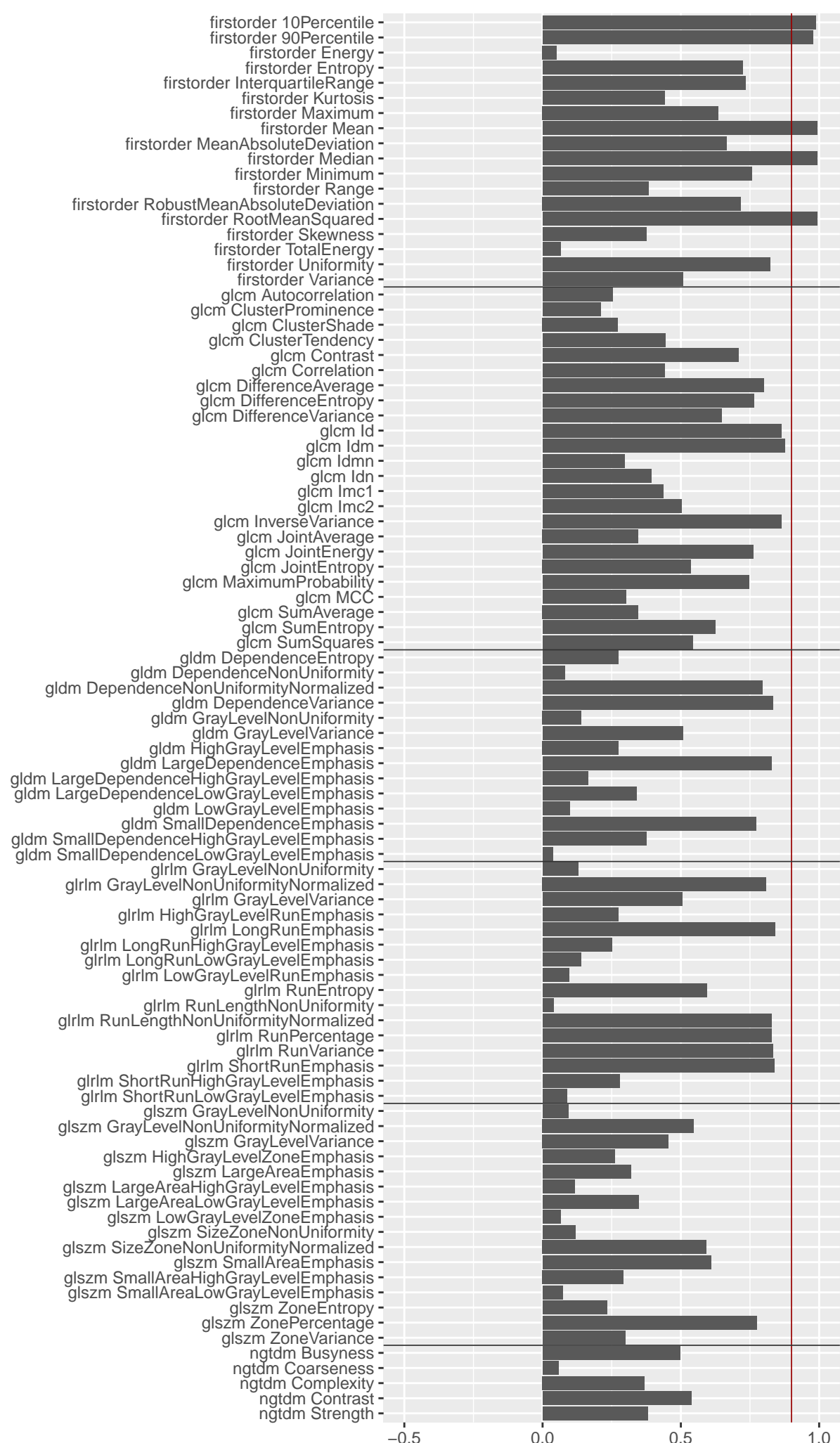

Supplement: Supplementary file 1 [file tomography-07-00073-s001.zip › SF7d_OCCCs_3_Tesla_II_T2_TSE_ROIs_20_30.pdf]

# 1.5 Tesla – Flash – 10 / 20 / 30 mm

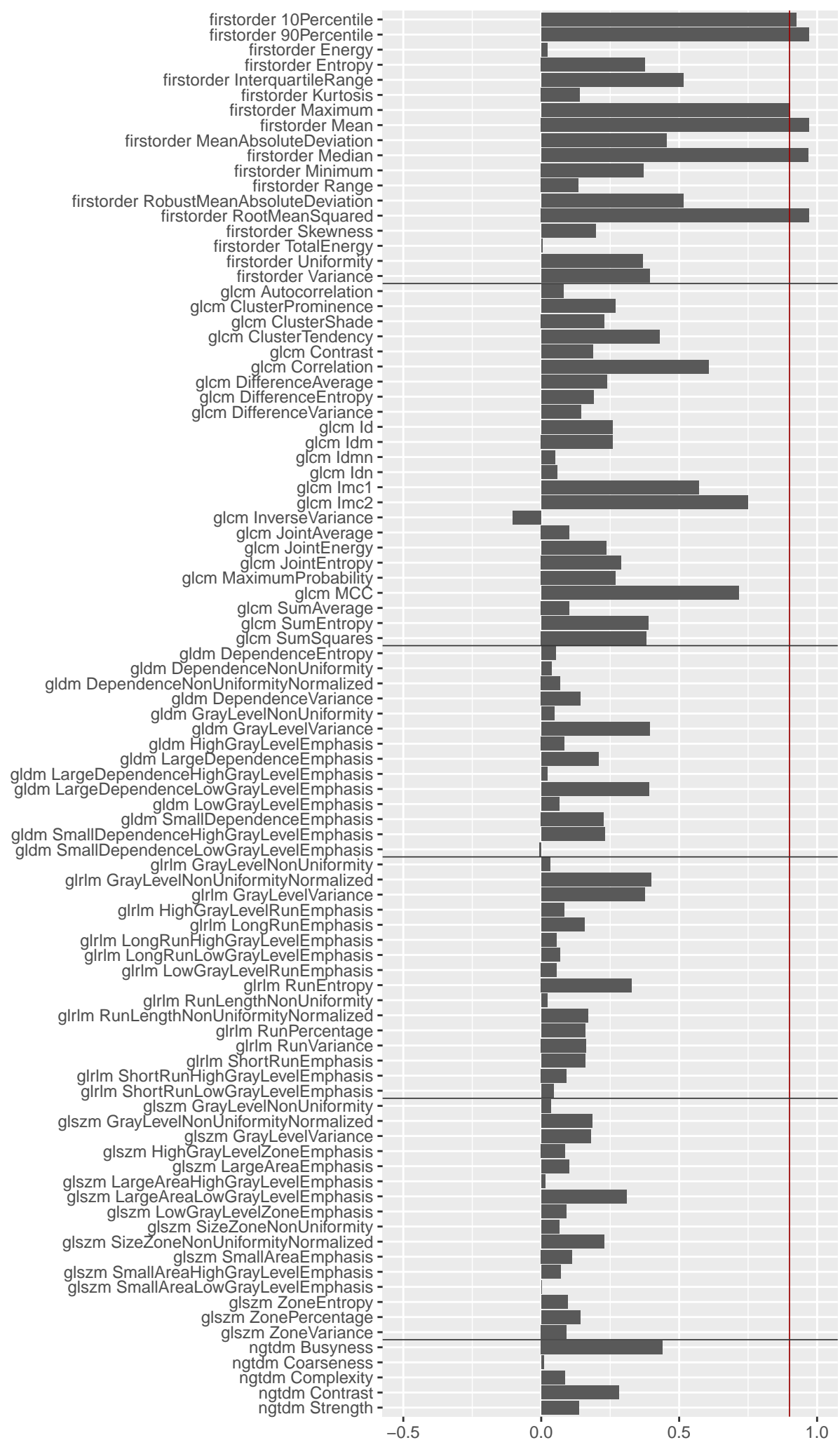

Supplement: Supplementary file 1 [file tomography-07-00073-s001.zip › SF8a_OCCCs_1.5_Tesla_T1_GRE_ROIs_10_20_30.pdf]

# 1.5 Tesla – Flash – 10 / 20 mm

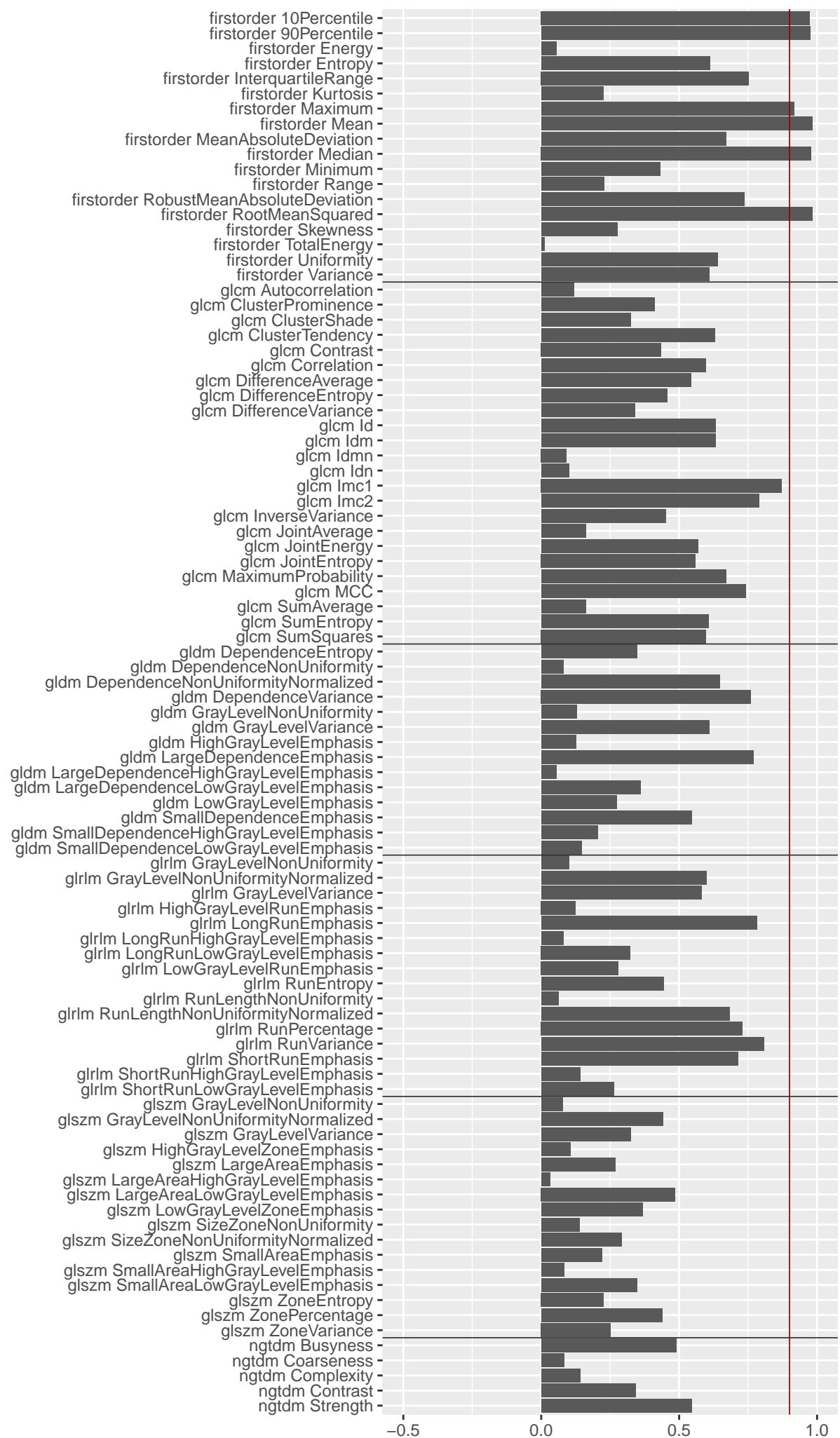

Supplement: Supplementary file 1 [file tomography-07-00073-s001.zip › SF8b_OCCCs_1.5_Tesla_T1_GRE_ROIs_20_30.pdf]

# 1.5 Tesla – Haste – 10 / 20 / 30 mm

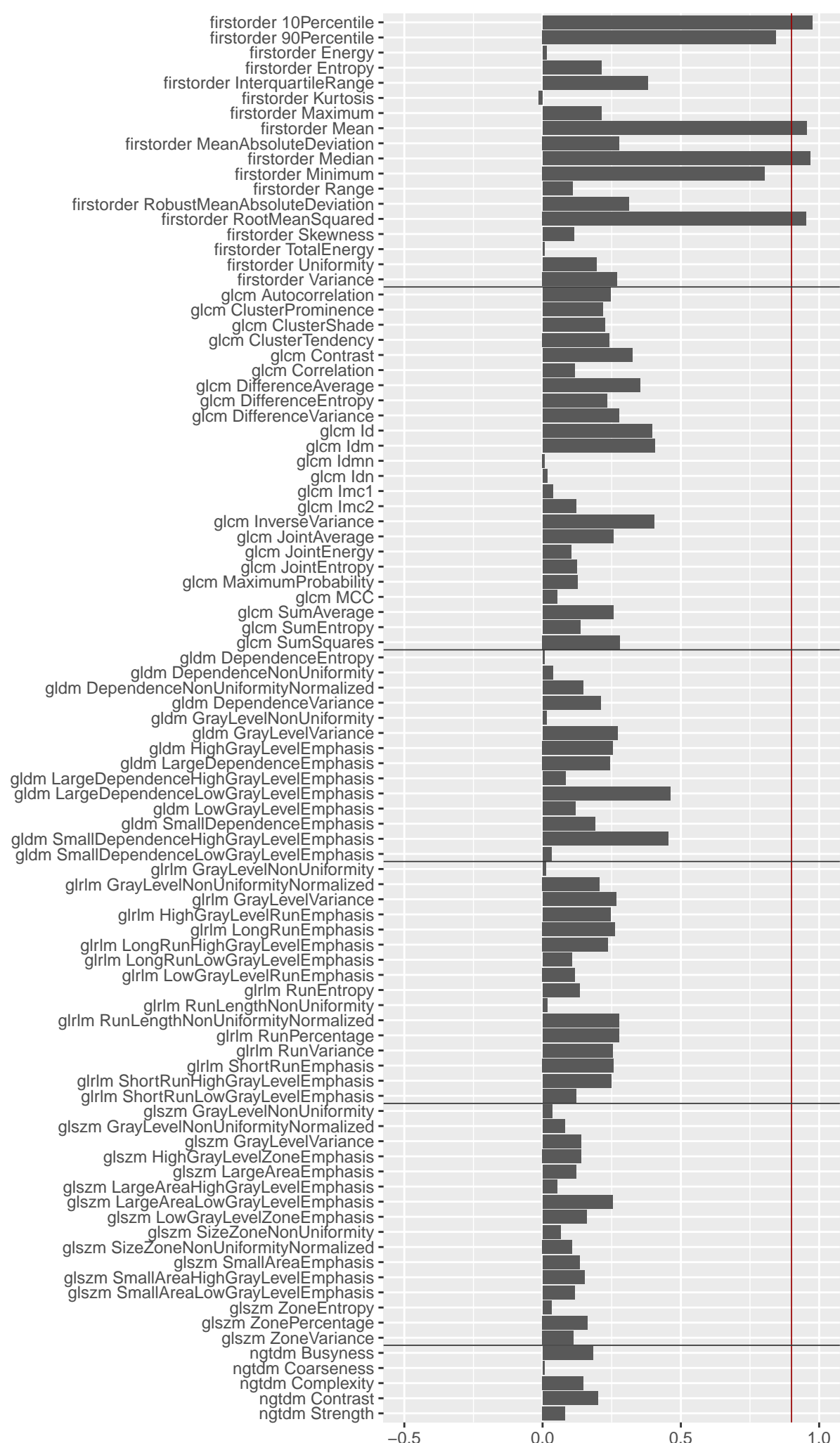

Supplement: Supplementary file 1 [file tomography-07-00073-s001.zip › SF8c_OCCCs_1.5_Tesla_T2_TSE_ROIs_10_20_30.pdf]

# 1.5 Tesla – Haste – 10 / 20 mm

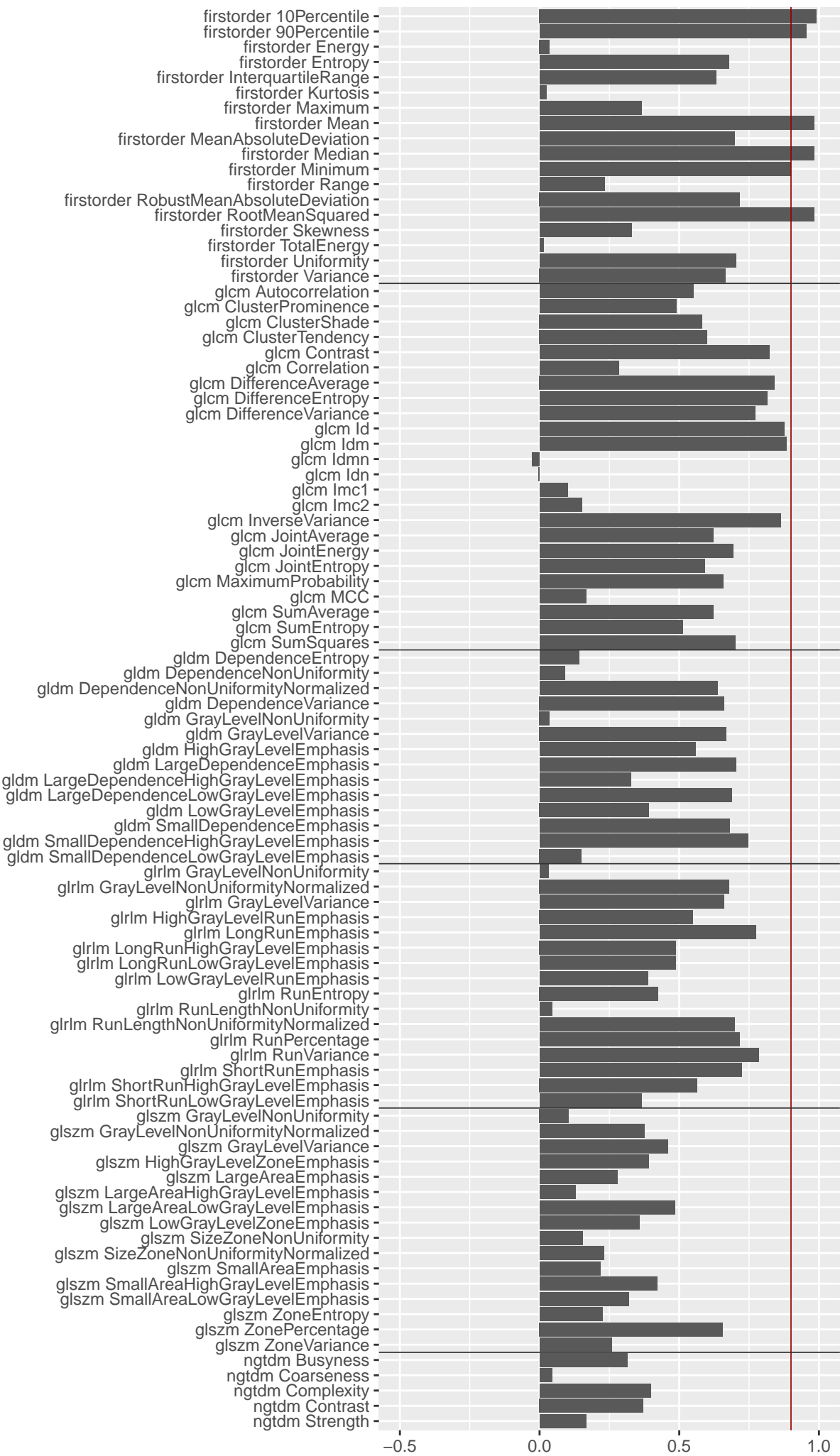

Supplement: Supplementary file 1 [file tomography-07-00073-s001.zip › SF8d_OCCCs_1.5_Tesla_T2_TSE_ROIs_20_30.pdf]
